# Formal synthesis of kibdelomycin and derivatisation of amycolose glycosides†

**DOI:** 10.1039/d3sc00595j

**Published:** 2023-03-03

**Authors:** Manuel G. Schriefer, Laura Treiber, Rainer Schobert

**Affiliations:** a Organic chemistry laboratory, University of Bayreuth Universitaetsstr. 30 95447 Bayreuth Germany Rainer.Schobert@uni-bayreuth.de

## Abstract

A convergent total synthesis of bacterial gyrase B/topoisomerase IV inhibitor kibdelomycin (a.k.a. amycolamicin) (1) was devised starting from inexpensive d-mannose and l-rhamnose, which were converted in new efficient ways to an *N*-acylated amycolose and an amykitanose derivative as late building blocks. For the former, we developed an expeditious, general method for the introduction of an α-aminoalkyl linkage into sugars *via* 3-Grignardation. The decalin core was built up in seven steps *via* an intramolecular Diels–Alder reaction. These building blocks could be assembled as published previously, making for a formal total synthesis of 1 in 2.8% overall yield. An alternative order of connecting the essential fragments was also made possible by the first protocol for the direct *N*-glycosylation of a 3-acyltetramic acid.

## Abstract 2

A new formal synthesis gave kibdelomycin with 2.8% yield over 19 steps, featuring a general method for introduction of a 3-(α-aminoalkyl) linkage into glycosides, and the first *N*-glycosylation of 3-acyltetramic acids.

## Graphical abstract

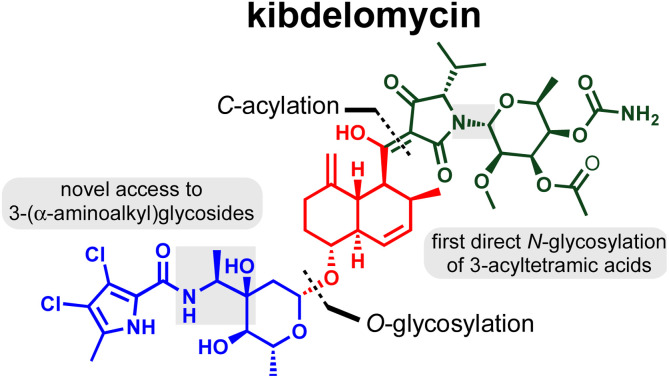

## Introduction

**Scheme 1:**
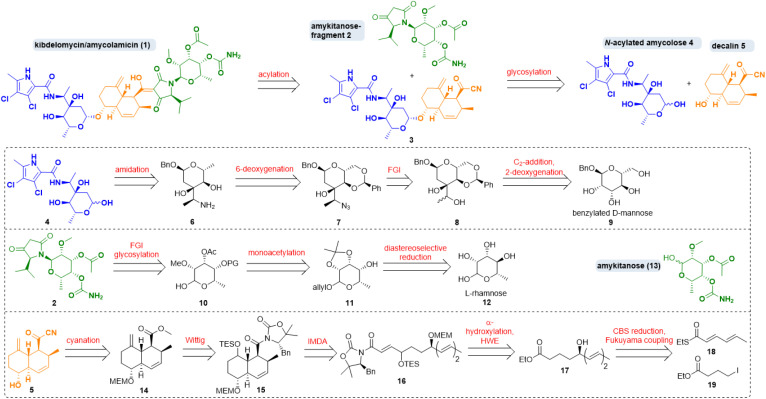
Retrosynthesis of kibdelomycin (1) and key fragments. FGI: functional group interconversion; IMDA: intramolecular Diels–Alder, HWE: Horner–Wadsworth–Emmons; CBS: Corey–Bakshi–Shibata.

Amycolamicin (1) (Scheme 1) was first mentioned in 2008/2009 in patents by Igarashi *et al.* who had isolated it from the bacterium *Amycolatopsis* sp. MK575-fF5.^1^ In 2010, proposals for its structure and for the biosynthesis of its *N*-acylated amycolose constituent 4, featuring an unusual α-aminoethyl branched sugar, were put forward.^2^ In 2011 Singh and coworkers isolated a compound from *Kibdelsporangium* sp. MA 7385 which they dubbed kibdelomycin and which they assumed to comprise a largely inverted amykitanose moiety when compared to the purported structure of amycolamicin.^3^ They recognised its extraordinary efficacy mainly against Gram-positive bacteria, including multidrug resistant pathogens from the ESKAPE panel. In 2012, a Japanese group disclosed a first crystal structure of the β-methyl anomer of amycolose and a revised structure of amycolamicin differing from the earlier one in the configuration of a stereogenic centre in the amykitanose.^4^ In 2014, Singh *et al.* settled the dispute over structure and stereochemistry with an X-ray diffraction analysis of crystals of kibdelomycin (1) bound to gyrase B/topoisomerase IV.^5^ They revised their original structure proposal and so proved that kibdelomycin and amycolamicin are one and the same.

Singh *et al.* also undertook extensive studies of structure activity relationships.^5^ Their crystal structure revealed a horseshoe-like conformation in which the dichlorinated pyrrole of amycolose amide 4 penetrates the ATP-binding pocket of gyrase B/topoisomerase IV which is the usual target of known topoisomerase IV inhibitory antibiotics. In contrast to gyrase-inhibiting antibiotics like novobiocin, the decalin, the tetramic acid and the amykitanose fragments of kibdelomycin protrude from the usual binding pocket, a possible explanation for it not showing cross resistance with established gyrase inhibitors.

Regarding its synthesis, kibdelomycin (1) can be dissected in three main parts, which are interesting synthetic targets in their own right. There is a decalinoyltetramic acid, a compound class known for its diverse biological activities.^6^ The decalin is *O*-glycosidically bound to a 3-α-aminoethyl-3,6-dideoxyhexopyra-nose. A 6-deoxygenated talose, carrying a methyl ether, an acetate and a carbamic acid, is attached to the tetramic acid by an *N*-glycosidic bond. The first synthetic foray towards kibdelomycin was the preparation of *N*-acyl amycolose 4 by Kuwahara *et al.* in 2019.^7^ Then, in quick succession, the groups of Li, Kuwahara and Baran published total syntheses of kibdelomycin within less than one year from December 2021 until 2022.^8,9^

## Results and discussion

Our retrosynthetic strategy for kibdelomycin (1) took advantage of a convergent route (Scheme 1). Disconnections were set (i) between *N*-acyl amycolose 4 and decalin fragment 5, requiring a challenging glycosylation of a 2-deoxy sugar in the forward direction, (ii) between decalin fragment 5 and *N*-amykitanosyltetramic acid 2, to be linked *via* a 3-acylation of the latter, and (iii) between amykitanose (13) and 5-isopropyltetramic acid as present in fragment 2. This strategy would harness our experience with decalinoyl- and *N*-glycosylated tetramic acids.^10^ While working on this project the three mentioned total syntheses were released, so that we decided not to frantically avoid a few of their obvious reaction steps but to concentrate on employing new and more efficient functional group interconversions for the sugar chemistry and to develop an expeditious formal total synthesis of 1.

The first synthesis by Yang *et al.* resembles ours most because of its convergence and the similarity of some retrosynthetic fragments.^9^ However, we chose distinctly different routes to decalin 5, *N*-acylated amycolose 4 and amykitanose 13. For the latter two we used a glycal approach with the advantage of not having to build up every single stereogenic centre by means of expensive catalysts and starting materials. For amycolose derivative 4 we decided to start from inexpensive benzylated d-mannose 9, which first had to be deoxygenated at 2-position, and in which it was necessary to instal an oxidised ethyl group at 3-position. After a second deoxygenation at 6-position and formation of the 3-(α-aminoethyl)sugar 6 the amidation with a dichlorinated pyrrole carboxylic acid should afford 4. For the synthesis of amykitanose fragment 2 we wanted to start from affordable l-rhamnose (12) instead of expensive l-fucose or l-talose. Key steps were the inversion at 4-position, the regioselective monoacetylation at 3-position, the *N*-glycosylation of 5-isopropyltetramic acid, and carbamate formation at C-4. For the synthesis of decalin fragment 5 any reaction other than an intramolecular Diels–Alder (IMDA) cycloaddition was out of the question. In a few steps, starting from thioester 18 and iodide 19, triene 16 should be accessible *via* Fukuyama coupling, stereoselective reduction of the resulting δ-ketoester to give hydroxyester 17, α-hydroxylation of the latter, and a chain-lengthening HWE-olefination. The following IMDA should afford mainly the *trans*-decalin scaffold, which had to be olefinated once more and converted to acyl cyanide 5. Due to the complexity of kibdelomycin (1) we had to pursue different synthetic routes to these key fragments. Foundered and abandoned attempts are detailed in the ESI.†

**Scheme 2:**
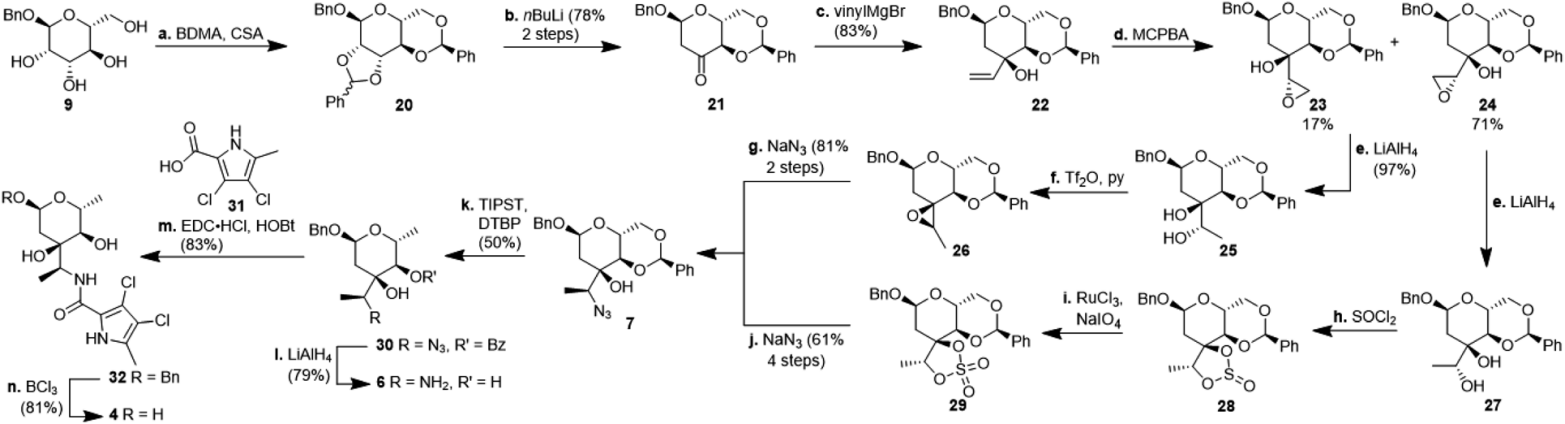
Synthesis of amycolose derivative 4. BDMA: benzaldehyde dimethyl acetal; CSA: camphorsulfonic acid; MCPBA: 3-chloroperbenzoic acid; Tf_2_O: triflic anhydride; TIPST: triisopropylsilanethiol; DTBP: di-*tert*-butylperoxide; EDC: *N*-(3-dimethylaminopropyl)-*N*′-ethylcarbodiimide; HOBt: 1-hydroxybenzotriazole.

**Fig. 1:**
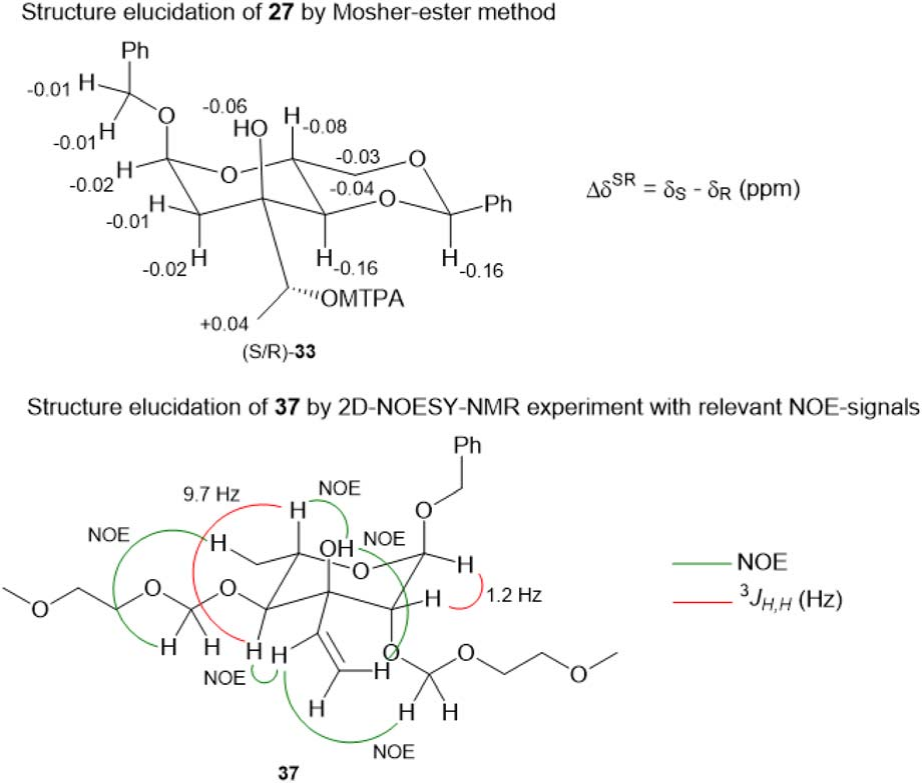
Structure elucidation of 27*via* Mosher ester method (top) and significant NOE-signals for the elucidation of the stereoconfiguration of 37 (bottom).

**Scheme 3:**
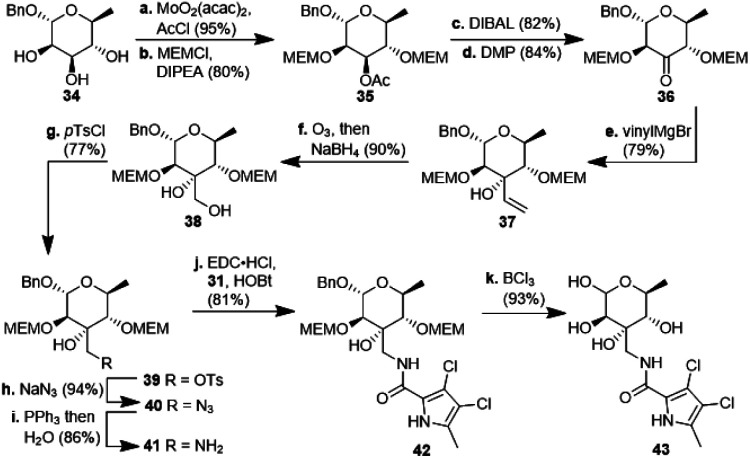
Synthesis of 3-aminomethyl-6-deoxyhexopyranose derivative 43 starting from benzylated l-rhamnose 34. MEM: methoxyethoxymethyl; DIPEA: diisopropylethylamine; DMP: Dess–Martin periodinane; Ts: tosyl.

For the synthesis of *N*-acylated amycolose 4, benzyl protected d-mannose 9 was reacted with benzaldehyde dimethyl acetal (BDMA) and camphorsulfonic acid (CSA) to give bisbenzylidene acetal 20. This was treated, without prior purification, with *n*BuLi at −78 °C to undergo a Klemer–Rodemeyer fragmentation upon warming to −35 °C, affording ketone 21 in 78% yield over two steps (Scheme 2).^11^ It is worthy of note that a *p*-methoxyphenyl (PMP) instead of a methyl, benzyl or propargyl protecting group at the anomeric position was cleaved under these conditions with release of PMPOH. The subsequent Grignard addition of vinyl magnesium bromide occurred exclusively from the side opposite to the neighbouring 4,6-benzylidene acetal. For the introduction of the amino group we intended an initial stereoselective formation of a secondary alcohol at the ethylene group, accessible *via* epoxidation and ensuing ring opening by a metal hydride, and its S_N_2-type substitution with sodium azide. The enantio- and diastereoselective Sharpless and VO(acac)_2_/TBHP epoxidations failed, whereas the Prilezhaev epoxidation gave the epoxides 24 and 23 in 88% yield as a separable 4 : 1 mixture of diastereomers which could both be used for the synthesis of 4. Epoxide opening by LiAlH_4_ afforded diols 25 (from 23) and 27 (from 24) quantitatively. Applying the Mosher ester method, alcohol 27 was found to be (*S*)-configured (Fig. 1, top).^12^ For the retention of its terminal stereogenic centre, diol 25 was submitted to two consecutive S_N_2-like reactions. Epoxide formation between the secondary and tertiary alcohol with Tf_2_O/pyridine afforded compound 26 which was treated immediately with NaN_3_ to furnish azide 7 in 81% over two steps. For the inversion of the terminal stereogenic centre of diol 27, it was first converted to the sulfite 28. This was oxidised with RuCl_3_/NaIO_4_ to sulfate 29 which was reactive enough to render azide 7 (61% over 4 steps) upon treatment with NaN_3_ and subsequent acidic hydrolysis of the intermediate sodium sulfate ester (*cf.* ESI† for details). While on small scale this hydrolysis was possible using aqueous H_2_SO_4_ (70% yield), at a larger scale aqueous H_2_SO_4_ led to cleavage of the benzylidene acetal and had to be replaced by a pH 4 citric acid buffer. For the 6-deoxygenation of 7 we followed the protocol of Dang *et al.* and employed a system of DTBP/TIPST for its radical-chain redox rearrangement to give benzoate 30.^13^ After an extensive optimisation this step proceeded with at least 50% yield, which spared us the use of the alternative Hanessian–Hullar reaction with subsequent dehalogenation.^14^ Treatment of benzoate 30 with LiAlH_4_ led to concomitant azide and benzoate reduction with 79% yield. The resulting amine 6 was selectively acylated with carboxylic acid 31 and EDC·HCl/HOBt to give amide 32 in 83% yield. Other amidation reagents such as BOP or HATU were less effective. Because of the potential hydrogenative dechlorination of the pyrrole we used BCl_3_ rather than Pd/C and H_2_ for the final debenzylation step. We obtained a mixture of α- and β-anomers of 4, the ratio of which was strongly dependent on the solvent and purification. Next, we checked the applicability of this synthesis to other sugars (Scheme 3). We chose l-rhamnose to test the introduction of an α-aminoalkyl residue. Benzylated l-rhamnose 34 was regioselectively 3-acetylated using a molybdenum catalyst.^15^ The hydroxyl groups at 2- and 4-position were MEM-protected (→ 35, 80%), because the downstream Grignard reaction would not work with bulky (TBS, Bn) or no protecting groups. After removal of the acetyl group by DIBAL (82%) and DMP-oxidation, ketone 36 was obtained with good yield. Its reaction with vinyl magnesium bromide gave the tertiary allyl alcohol 37 in 79% yield and *dr* > 30 : 1. A 2D-NOESY-experiment proved that the Grignard reagent had attacked from the site opposite to the C4-OMEM group (Fig. 1, bottom). This finding also shows that the group at C4, directing diastereoselective additions, need not be a large 4,6-benzylidene acetal. Next, alkene 37 was converted to primary alcohol 38 by ozonolysis which was tosylated to give 39 that was converted to azide 40. After Staudinger reaction, the resulting amine 41 was acylated with pyrrole carboxylic acid 31 to give amide 42 in 81% yield. Finally, the benzyl group at the anomeric position as well as both MEM protecting groups of 42 were removed by BCl_3_ in a single step to give the rhamnose derivative 43 in excellent 17% yield over 11 steps. With the synthesis of amycolose and a rhamnose derivative, we demonstrated that this method may be used in general to introduce an α-aminoalkyl linkage in sugars. Moreover, the vinyl group is amenable to a good many other functionalisations (*cf.* ESI†). This aspect might facilitate diversity-oriented syntheses of highly functionalised sugars, including even amycolose, given its known cell growth suppression and possible application as an anticancer medication.^16^

**Scheme 4:**
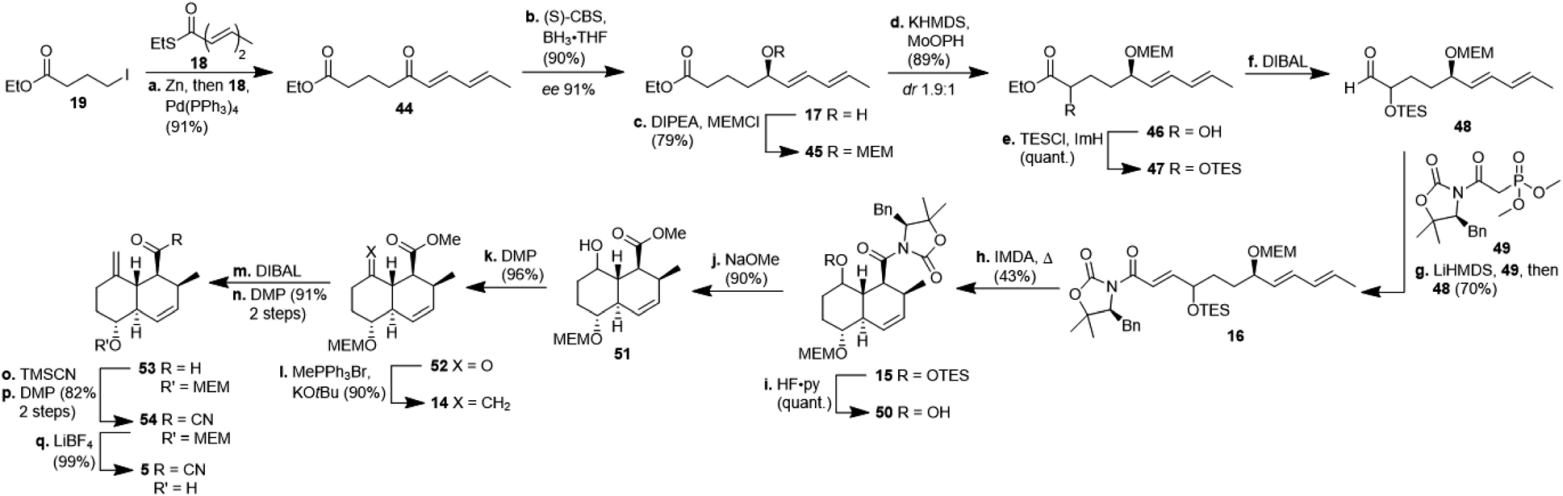
Synthesis of decalin fragment 5 starting from ethyl 4-iodobutyrate 19. CBS: Corey–Bakshi–Shibata catalyst; KHMDS: potassium hexamethyldisilazide; MoOPH: oxodiperoxymolybdenum(pyridine)(hexamethylphosphoric triamide); TES: triethylsilyl; ImH: imidazole; LiHMDS: lithium hexamethyldisilazide; TMSCN: trimethylsilyl cyanide.

The synthesis of decalin fragment 5 started with a Fukuyama coupling between ethyl 4-iodobutyrate 19 and ethyl (2*E*,4*E*)-hexa-2,4-dienethioate 18 to give δ-ketoester 44 in 91% yield (Scheme 4).^17^ The ketone was reduced with BH_3_ in the presence of (*S*)-CBS-catalyst affording alcohol 17 with 90% yield and an ee of 91%. This protocol is easier to use on a laboratory scale than a recently published asymmetric Noyori-type hydrogenation of α,β,γ,δ-unsaturated ketones.^18^ Unlike other groups who applied a more than quantitative amount of CBS-catalyst, we realised that the reduction proceeded with higher ee when using a merely catalytic amount of CBS-catalyst. After MEM-protection of the alcohol to give ether 45 with 79% yield, a non-trivial α-hydroxylation had to be done at this post-Fukuyama stage, since α-hydroxylated esters from the chiral pool failed to undergo the Fukuyama coupling due to not forming the respective zinc organyl (*cf.* ESI†). After quite a few failed attempts with sulfonyloxaziridines, we identified MoOPH/KHMDS as a viable α-hydroxylating agent affording α-hydroxyester 46 with 89% yield and 1.9 : 1 *dr*. The TES-protected ester 47 was reduced with DIBAL to aldehyde 48 and the latter was submitted to a HWE-olefination with phosphonate 49 to give the triene 16 comprising the SuperQuat auxiliary (70%, two steps). Because HWE-reactions with Evans/Davies auxiliary bearing phosphonates only worked with α-hydroxylated aldehydes but not so with α-methylene substituted aldehydes (*cf.* ESI†) we had to postpone the introduction of the methylene group until after the decalin formation. We opted for Davies' SuperQuat auxiliary for the following Diels–Alder reaction, after many attempts to remove an Evans auxiliary had failed after successful Diels–Alder reaction and in accordance with the results of Frossard *et al.*^19^ Unlike most who use AlMeCl_2_ as a catalyst for the IMDA, we had better results when heating triene 16 in toluene at 80 °C over 3 d which afforded octalin 15 with 43% yield besides some separable undesired *cis*-octalin. Quantitative removal of the TES-protecting group with HF pyridine complex left the alcohol 50 which had its auxiliary cleaved off with sodium methoxide to give hydroxyester 51 with 90% yield. The introduction of the methylene unit was achieved by oxidising alcohol 51 with DMP (96%) and treating the resulting ketone 52 with methylenetriphenylphosphorane. The resulting ester 14 (90%) was reduced to aldehyde 53 in two steps, *i.e.* reduction to the corresponding alcohol with DIBAL and subsequent oxidation with DMP, because of overreduction issues. Reaction of aldehyde 53 with TMSCN led to a cyanohydrin, which was right away oxidised with DMP to acyl cyanide 54. Cleavage of the MEM-group, liberating decalin 5, proceeded best using LiBF_4_ compared to TiCl_4_ or TFA. This synthesis of the central decalin building block has an edge over those of the previous kibdelomycin syntheses due to its high yielding, simple steps and inexpensive starting materials. Most reactions were performed on a gram scale without yields decreasing.

**Scheme 5:**
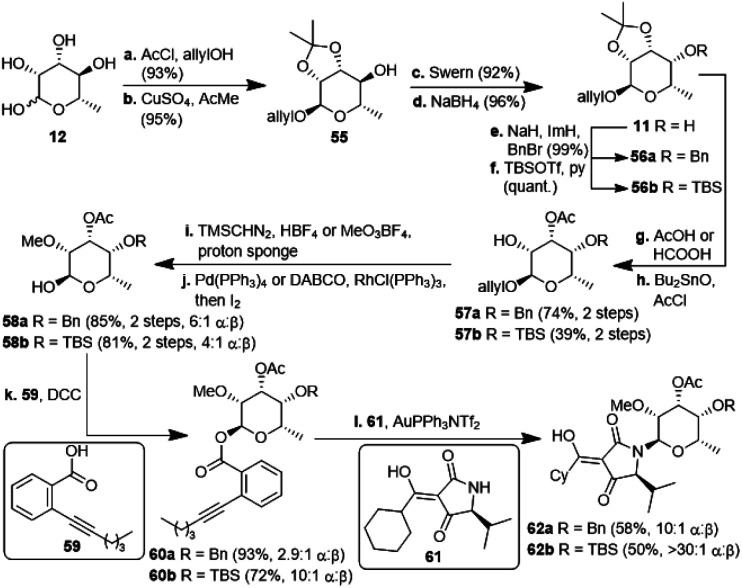
Synthesis of *N*-glycosylated 3-cyclohexanoylltetramic acids 62a/b. TBS: *tert*butyldimethylsilyl; Tf: triflyl; DABCO: 1,4-diazabicyclo[2.2.2]octane; DCC: dicyclohexylcarbodiimide.

**Scheme 6:**
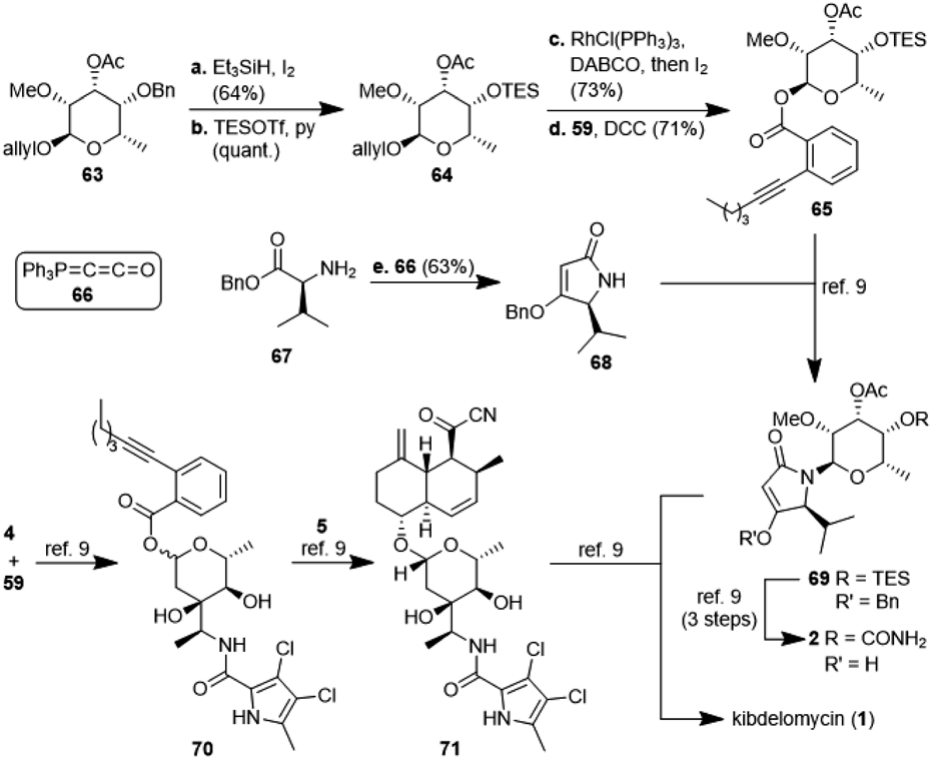
Synthesis of glycoside 65 and tetramate 68 as well as a formal synthesis of kibdelomycin (1) according to ref. 9.

The second, amykitanose-related sugar fragment was synthesised starting from l-rhamnose (12) (Scheme 5). It was allylated at the anomeric position in 93% yield and its *syn*-diol was protected as an isopropylidene acetal using anhydrous CuSO_4_ (95%). The allyl protecting group was chosen since the cleavage of the comparable methyl acetal later on in the synthesis had failed in the presence of other necessary functional groups, *e.g.* because of the instability of the acetyl group. The configuration at the 4-position of the resulting compound 55 was inverted by a sequence of Swern oxidation and ensuing reduction with NaBH_4_ to give a single diastereomer of 11 in 88% over two steps. Benzylation of the hydroxyl group led to fully protected sugar 56a. After deprotection of the *syn*-diol, the hydroxyl group at 3-position was acetylated selectively under optimised conditions to afford sugar 57a in 74% yield over two steps.^20^ Methylation at 2-positon was difficult due to the acetyl group getting easily removed under basic conditions, but was eventually achieved using TMSCHN_2_ and HBF_4_. Deprotection of the anomeric position in acidic milieu under Pd-catalysis gave sugar 58a. All attempts at coupling it with any kind of tetramic acid *via* different customary methods in order to establish analogues of amykitanose fragment 2, as well as Dieckmann cyclisation based sequences failed (*cf.* ESI†).^21^ As a last resort and based on the first total synthesis of kibdelomycin by Li *et al.*,^9^ sugar 58a was esterified with carboxylic acid 59 and the resulting ester 60a was coupled with 3-cyclohexanoyl-tetramic acid 61*via* Au-catalysis affording *N*-glycoside 62a in a decent 58% yield.^22^ The cyclohexyl residue was to mimic the octalin moiety. As far as we know, this is the first example of a direct *N*-glycosylation of a 3-acyltetramic acid. The anomeric ratio of 10 : 1 was inferior to the 20 : 1 ratio reported by Li *et al.*^9^ for the *N*-glycosylation of 3*H*-5-isopropylpyrrolidin-2,4-dione. The divergent results could only be attributed to the different protecting groups at 4-position of the sugar (Bn *vs.* TES). To verify this assumption, we introduced a silyl protection group as in compound 58b. The following steps were identical to those for the 4-OBn analogues, albeit with slightly different reaction conditions because of the instability of the TBS-group in an acidic milieu. Even the esterification of 58b with carboxylic acid 59 showed the influence of the protecting group, since the anomeric ratio of the resulting sugar 60b increased to 10 : 1 α : β. After coupling with the 3-acyl tetramic acid 61, the *N*-glycoside 62b was isolated with an α : β-ratio of >30 : 1. For a strict formal total synthesis, the TES-protected sugar 65 was required (Scheme 6). So, we removed the benzyl group of compound 63, obtained from methylation of glycoside 57a, with *in situ* generated HI, and replaced it with a triethylsilyl group to afford compound 64. De-allylation of the latter and glycosylation with acid 59 gave ester 65 in excellent 15% yield over 12 steps, comparable with the corresponding sequence of the first total synthesis by Li *et al.*^9^ Glycoside 65 can then be coupled with 4-*O*-benzyl 5-isopropyltetramate 68 as shown in the first total synthesis of kibdelomycin.^9^ Tetramate 68 is readily accessible in one step and 63% yield from reaction of ketenylidenetriphenylphosphorane (66) with l-valine benzyl ester (67).^23^ Removal of the benzyl group in glycoside 63 also opened the door for the synthesis of amykitanose (13) in three more steps (*cf.* ESI† for a not yet optimised protocol). The formal synthesis of kibdelomycin (1) can be completed by esterification of amycolose derivative 4 with acid 59 to give 70 and subsequent use of the latter for glycosylation of decalin fragment 5 to give compound 71. Glycoside 65 can be converted to tetramic acid fragment 2 in four steps. Acylation of tetramic acid 2 with ketonitrile 71 using 1-hydroxy-7-azabenzotriazole (HOAt) and triethylamine finally affords kibdelomycin (1). For the completion of an alternative total synthesis exploiting the novel *N*-glycosylation of 3-acyltetramic acids *cf.* the ESI.†

## Conclusion

In summary we developed an expeditious formal synthesis of kibdelomycin (1) starting from inexpensive compounds and employing simple and high-yielding standard protocols, even on a large scale. The stereochemical information stems from the chiral pool or from highly diastereoselective reactions. The longest linear sequence of the factual synthesis of the fragments amounts to a competitive 19 steps. With all fragments in hand, a formal synthesis following the protocol of Yang *et al.* leads to kibdelomycin (1, 2.8% overall yield).^9^ During our research, we developed a method for introduction of an α-aminoalkyl linkage into sugars *via* Grignard addition to C3 which also opens access to a range of other functionalities. It could be used to synthesise different derivatives of kibdelomycin (1) for structure–activity relationship studies or for an optimisation of its applicability and efficacy. As a side benefit, we also report the first *N*-glycosylation of a 3-acyltetramic acid.

## Data availability

The datasets and spectra supporting this article have been uploaded as part of the ESI† material.

## Author contributions

M. G. S. planned and carried out all reactions concerning amycolose, planned the synthesis of derivatives of amycolose, and wrote parts of the manuscript. L. T. planned and carried out all syntheses concerning amykitanose and rhamnose derivatives and wrote parts of the manuscript. L. T. and M. G. S. planned and realised the synthesis of decalin fragment 5. R. S. supervised the syntheses and assisted with manuscript preparation.

## Conflicts of interest

There are no conflicts to declare.

## Supplementary Material

SC-014-D3SC00595J-s001
