# Supplementary material for: Formal synthesis of kibdelomycin and derivatisation of amycolose glycosides
Source: Chem Sci. 2023 Mar 3;14(13):3562–8. doi: 10.1039/d3sc00595j (PMC10055692; doi:10.1039/d3sc00595j)
Supplement: SC-014-D3SC00595J-s001 [file SC-014-D3SC00595J-s001.pdf]

Electronic Supplementary Material (ESI) for Chemical Science.
This journal is © The Royal Society of Chemistry 2023

Formal synthesis of kibdelomycin and derivatisation of amycolose glycosides

Manuel Georg Schriefer [a], *Laura Treiber* [a], *Rainer Schobert* [a,*]

[a] *Organic chemistry laboratory, University of Bayreuth, Universitaetsstr. 30, 95447 Bayreuth, Germany.*

Supporting Information

Table of contents

1. General information 2
2. Experimental procedure 3
   2.1 Synthesis of amycolose fragment **4** 3
   2.2 Synthesis of pyrrole carboxylic acid **31** 15
   2.3 Failed routes to amycolose derivative **4** 18
   2.4 Synthesis of sugar **43** – derivatization of amycolose 20
   2.5 Synthesis of decalin fragment **5** 31
   2.6 Failed routes to the decalin 46
   2.7 Synthesis of reagents for the decalin fragment 47
   2.8 Synthesis of glycosides **62a** and **62b** 51
   2.9 Synthesis of 3-acyltetramic acid **61** 66
   2.10 Synthesis of acid **59** 68
   2.11 Synthesis of glycoside **65** for the formal synthesis 71
   2.12 Failed routes to amykitanose 75
   2.13 Synthesis amykitanose (**13**) 81
   2.14 Alternative formal synthesis of kibdelomycin (**1**) 82
3. References 83
4. NMR-Spectra 84

1. General information

Melting points were determined with a Büchi M-565 melting point apparatus and are uncorrected. IR spectra were recorded with a PerkinElmer Spectrum 100 FT-IR spectrophotometer (PerkinElmer, Rodgau, Germany) with ATR sampling unit. Optical rotations were measured at 589 nm (Na-D line) on a PerkinElmer 241 polarimeter (PerkinElmer, Rodgau, Germany); $[\alpha]_D^{20}$ (c g/100mL, solvent) values are given in 10^{-1} deg cm^2 g^{-1}. High resolution mass spectra were obtained with a UPLC/Orbitrap MS system in ESI mode (ThermoFisher Scientific, Bremen, Germany). NMR spectra were recorded with a Bruker Avance III HD 500 spectrometer (^{1}H NMR: 500 MHz and ^{13}C NMR: 125 MHz) (Bruker, Karlsruhe, Germany). Chemical shifts are given in parts per million, relative to the residual solvent peak as an internal standard and coupling constants (*J*) are quoted in Hz. Most tetramic acids were measured in $CDCl_3$ and in CD_3OD. In the latter they usually exist as a single (enol) tautomer. Quaternary C-atoms of tetramic acids were sometimes difficult to spot in JMOD or ^{13}C NMR spectra. For these, more signals cropped up in HMBC and/or HSQC correlation spectra and were considered for peak assignment. In $CDCl_3$ solution, signals of virtually all C-atoms of tetramic acids were visible yet split up in multiple, difficult to assign sets for individual tautomers both in ^{1}H and JMOD/^{13}C NMR spectra. In line with literature, we assume the tautomers with exocyclic C–C double bond as drawn for the 3-acyltetramic acids in scheme S10, to be the major tautomer.[1] For the purification of synthetic products, chromatography silica gel 60 (40–63 µm) or silica gel RP18 (40–63 µm) were used. Analytical thin layer chromatography (TLC) was carried out using Merck silica gel 60 F254 pre-coated aluminum-backed plates. Analytical HPLC was performed on a Shimadzu Nexera XR (Shimadzu GmbH, Duisburg, Germany) using a Knauer Eurospher II C18-column (150 × 4 mm) (Knauer GmbH, Berlin, Germany). Enantiomeric excess was determined by HPLC analysis (Waters Alliance HPLC; Waters 2695 Separation Module, Waters 2487 Dual λ Absorbance Detector) on chiral phase (Daicel Chiralpak OD3). All reagents were purchased from commercial sources and were used without further purification. All anhydrous solvents were used as supplied, except tetrahydrofuran, 1,4-dioxane and toluene which were freshly distilled over sodium/benzophenone, dichloromethane (CH_2Cl_2) which was freshly distilled over CaH_2, dimethylformamide (DMF) which was dried over molecular sieves (3 Å), and methanol (MeOH) which was freshly distilled over Mg. Moisture or air sensitive reactions were routinely carried out in oven-dried glassware under an argon atmosphere using standard Schlenk technique.

2. Experimental procedure

2.1 Synthesis of amycolose fragment **4**

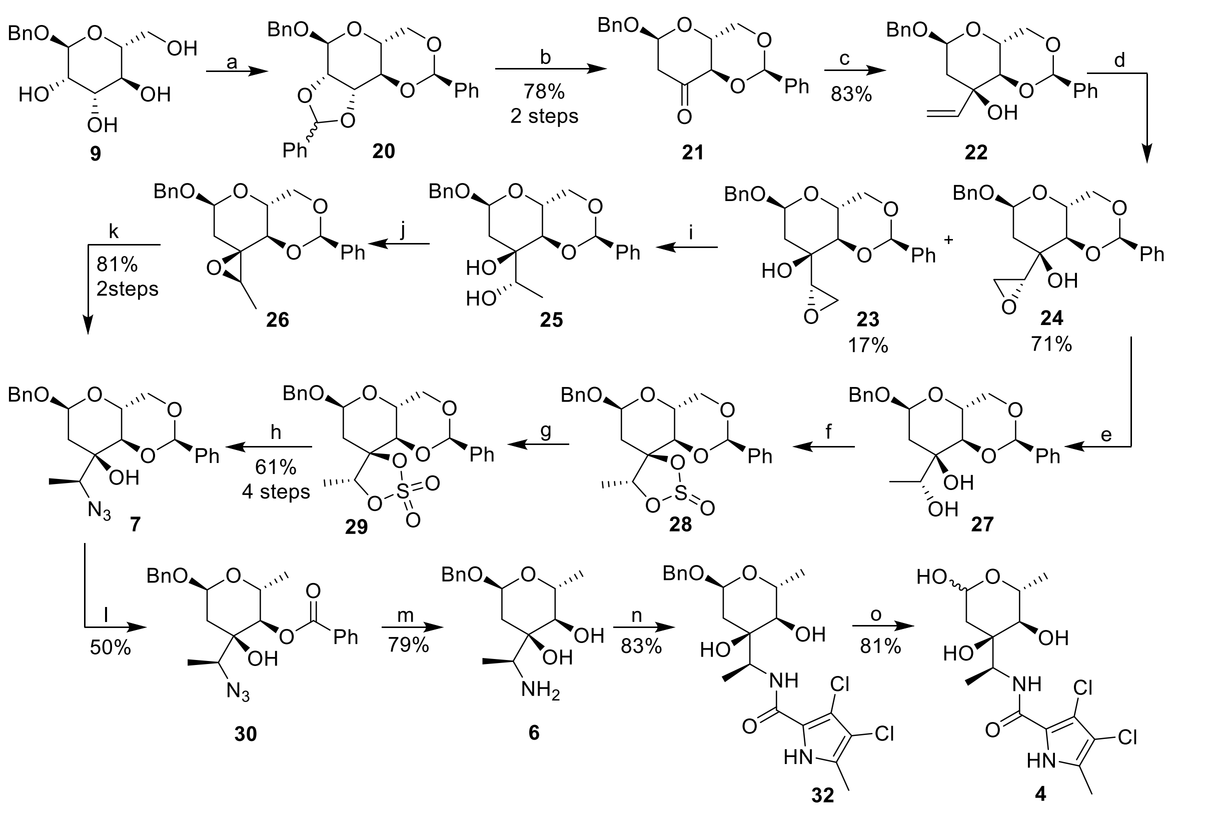

Scheme S1. Synthesis of amycolose derivate **4** starting from benzylated D-mannose **9**.
a) BDMA, CSA, $CHCl_3$, 80 °C, 6.5 h; b) *n*BuLi, THF, −78 °C→−35 °C, 3.75 h; c) VinylMgBr, THF, −78 °C, 3 h; d) *m*CPBA, CH_2Cl_2, rt, 22 h; e) $LiAlH_4$, THF, 0 °C→rt, 2.5 h; f) $SOCl_2$, NEt_3, CH_2Cl_2, 0 °C, 3 h, g) $NaIO_4$, $RuCl_3{\cdot}xH_2O$, MeCN, rt, 7 h; h) 1. NaN_3, DMF, 65 °C, 6.75 h, 2. Citric acid buffer, EtOAc, 45 °C, 15 h, 3. Citric acid, 3.5 h; i) $LiAlH_4$, THF, 0 °C→rt, 1.75 h; j) Tf_2O, pyridine, CH_2Cl_2, −78 °C→0 °C, 1.25 h; k) NaN_3, NH_4Cl, MeOH, 80 °C, 12 h; l) TIPST, DTBP, *n*-octane, 140 °C, 6.75 h; m) $LiAlH_4$, THF, 0 °C→rt, 24 h; n) **31**, HOBt, EDC·HCl, NEt_3, CH_2Cl_2, 0 °C→rt, 16 h; o) BCl_3, CH_2Cl_2, −80 °C, 40 min.

(2*R*,4a*R*,6*S*,8a*R*)-6-(Benzyloxy)-2-phenyltetrahydropyrano[3,2-*d*][1,3]dioxin-8(4*H*)-one (21)

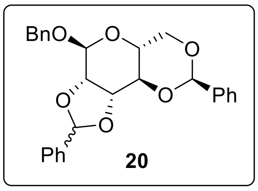

Benzylated mannose (**9**, 5.50 g, 20.3 mmol, 1.00 eq.) was solved in $CHCl_3$ (100 mL) and BDMA (7.02 mL, 46.8 mmol, 2.30 eq.) and CSA (709 mg, 3.05 mmol, 0.15 eq.) was added. The solution was heated at 80 °C and the vapor condensed in another flask. The reaction flask was refilled every hour with $CHCl_3$ (ca. 50 mL) and stirred at 80 °C for 6.5 h. The solution was poured into sat. aq. $NaHCO_3$ solution (200 mL) and extracted with CH_2Cl_2 (3×200 mL). The

combined organic phases were washed with sat. aq. $NaHCO_3$ solution (3×150 mL) and brine (150 mL), dried over Na_2SO_4 and evaporated. The bis-acetal **20** (7.97 g, quant.) was immediately used without further purification for the next step. It was isolated as a diastereomeric mixture. $\mathbf{R_f}$ = 0.38 (hexanes/EtOAc 6:1); **^{1}H-NMR** (500 MHz, $CDCl_3$) δ 7.56-7.29 (m, 15H), 6.29 (s, 0.60H), 5.96 (s, 0.31H), 5.65 (s, 0.61H), 5.53 (s, 0.32H), 5.28 (s, 0.31H), 5.22 (s, 0.60H), 4.78-4.49 (m, 3H), 4.38-4.19 (m, 2H), 3.94-3.72 (m, 3H) ppm; **HRMS** ESI *m/z* $[M + H]^+$ calcd. for $C_{27}H_{27}O_6$ 447.18022, found 447.17924.

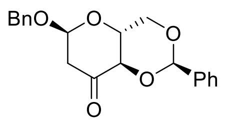

21

The raw bis-acetal **20** (7.97 g, 20.3 mmol, 1.00 eq) solved in dry THF (190 mL) at −78 °C under argon atmosphere and was treated with *n*BuLi (2.5M hexanes, 24.4 mL, 2.60 eq.) over 15 minutes. The solution was stirred at −78 °C for 3 h and at −35 °C for 30 min. Sat. aq. NH_4Cl-solution (100 mL) was added and the organic phase was removed by rotary evaporation. The resulting yellow solid was collected by filtration, washed with water (50 mL), crushed, and washed with *n*-pentane (50 mL). The pale yellow solid ketone **21** (5.48 g, 78% over two steps) was dried at the rotary evaporator and was pure enough for the next step. $\mathbf{R_f}$ = 0.47 (hexanes/EtOAc 3:2); **mp** 122 °C (decomposition); $[\alpha]_D^{20}$ +81.8° (c 1.0 in $CHCl_3$); **IR** ν_{max}/cm^{-1} 3069 (w), 3032 (w), 2932 (w), 2869 (w), 1733 (w), 1454 (m), 1379 (m), 1267 (m), 1214 (m), 1129 (s), 1093 (s), 1018 (s); **^{1}H-NMR** (500 MHz, $CDCl_3$) δ 7.51 (m, 2H), 7.39-7.30 (m, 8H), 5.59 (s, 1H), 5.33 (d, 1H, *J* = 4.8 Hz), 4.72 (d, 1H, *J* = 12.2 Hz), 4.55 (d, 1H, *J* = 12.2 Hz), 4.32 (m, 2H), 4.22 (dt, 1H, *J* = 4.8, 10.0 Hz), 3.91 (t, 1H, *J* = 10.1 Hz), 2.86 (ddd, 1H, *J* = 1.2, 4.9, 14.7 Hz), 2.72 (dd, 1H, *J* = 0.9, 14.7 Hz) ppm; **^{13}C-NMR** (125 MHz, $CDCl_3$) δ 197.7, 136.8, 136.6, 129.5, 128.7, 128.5, 128.2, 128.1, 126.6, 102.3, 98.8, 83.3, 69.6, 69.5, 65.5, 46.5 ppm; **HRMS** ESI *m/z* $[M + Na]^+$ calcd. for $C_{20}H_{20}O_5Na$ 363.12029, found 363.11918.

(2*R*,4a*R*,6*S*,8*R*,8a*R*)-6-(Benzyloxy)-2-phenyl-8-vinylhexahydropyrano[3,2-*d*][1,3]dioxin-8-ol (22)

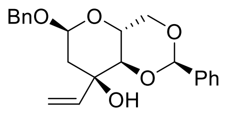

22

Ketone **21** (213 mg, 626 µmol, 1.00 eq.) was solved in dry THF (6.3 mL) under argon atmosphere at −78 °C. VinylMgBr (1M THF, 1.88 mL, 1.88 mmol, 3.00 eq.) was slowly dropped into the solution which was stirred for 3 h at −78 °C. Sat. aq. NH_4Cl solution (30 mL) and H_2O (30 mL) were added, and the aqueous phase was extracted with EtOAc (3×50 mL). The combined organic phases were washed with brine (50 mL), dried over Na_2SO_4, and evaporated. The crude allyl alcohol **22** was

purified by column chromatography (SiO_2, pentane/EtOAc 4:1). The alcohol **22** (192 mg, 83%) was obtained as colourless solid. **R$_f$** = 0.82 (hexanes/EtOAc 3:2); **mp** 109.6 °C; $[\alpha]_D^{20}$ +139.7° (c 1.0 in $CHCl_3$); **IR** ν_{max}/cm^{-1} 3518 (br. w), 3067 (w), 3033 (w), 2968 (w), 2933 (w), 2863 (w), 1455 (m), 1387 (m), 1116 (s), 1089 (s), 1017 (s), 905 (s); **^{1}H-NMR** (500 MHz, $CDCl_3$) δ 7.48 (m, 2H), 7.40-7.28 (m, 8H), 5.89 (dd, 1H, J = 10.8, 17.2 Hz), 5.59 (s, 1H), 5.45 (dd, 1H, J = 1.3, 17.2 Hz), 5.21 (dd, 1H, J = 1.3, 10.8 Hz), 5.00 (dd, 1H, J = 1.2, 3.7 Hz), 4.79 (d, 1H, J = 12.0 Hz), 4.56 (d, 1H, J = 12.0 Hz), 4.28 (m, 2H), 4.22 (dt, 1H, J = 4.8, 10.0 Hz), 3.78 (m, 1H), 3.59 (d, 1H, J = 9.3 Hz), 3.56 (s, 1H), 2.05 (dd, 1H, J = 1.3, 14.8 Hz), 2.01 (dd, 1H, J = 3.8, 14.8 Hz) ppm; **^{13}C-NMR** (125 MHz, $CDCl_3$) δ 140.5, 137.5, 137.0, 129.0, 128.7, 128.3, 128.2, 128.2, 126.3, 115.3, 102.0, 96.4, 82.3, 71.0, 69.7, 69.4, 60.0, 40.4 ppm; **HRMS** ESI m/z [M + Na]$^+$ calcd. for $C_{22}H_{24}O_5Na$ 391.15160, found 391.15074.

(2*R*,4a*R*,6*S*,8*R*,8a*R*)-6-(Benzyloxy)-8-((*S*)-oxiran-2-yl)-2-phenylhexahydropyrano[3,2-*d*][1,3]dioxin-8-ol (23) and (2*R*,4a*R*,6*S*,8*R*,8a*R*)-6-(benzyloxy)-8-((*R*)-oxiran-2-yl)-2-phenylhexahydropyrano[3,2-*d*][1,3]dioxin-8-ol (24)

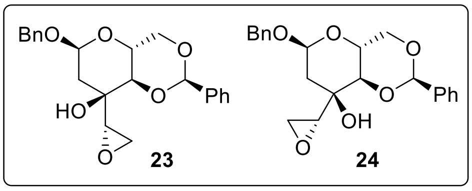

To a solution of allylalcohol **22** (50 mg, 136 µmol, 1.00 eq.) in CH_2Cl_2 at room temperature was added MCPBA (58.5 mg, 339 µmol, 2.50 eq.). The solution was stirred for 22 h and sat. aq. $Na_2S_2O_3$ solution (2 mL) and sat. aq. $NaHCO_3$ solution (2 mL) was added. The mixture was extracted with EtOAc (3×15 mL), the combined organic phases were washed with 10% K_2CO_3 solution (15 mL) and brine (15 mL), dried over Na_2SO_4 and evaporated. The diastereomeric mixture was separated by SiO_2 column chromatography (pentane/EtOAc 5:1 to 2:1). The optical pure epoxides **24** (37 mg, 71%) and **23** (9 mg, 17%) were isolated as colourless crystalline solids. **24**: **R$_f$** = 0.39 (hexanes/EtOAc 2:1); **mp** 113.9 °C; $[\alpha]_D^{20}$ +99.0° (c 1.0 in $CHCl_3$); **IR** ν_{max}/cm^{-1} 3507 (br. w), 3067 (w), 3035 (w), 2934 (w), 2864 (w), 1455 (m), 1388 (m), 1099 (s), 1018 (s), 905 (s); **^{1}H-NMR** (500 MHz, $CDCl_3$) δ 7.49 (m, 2H), 7.39-7.28 (m, 8H), 5.65 (s, 1H), 5.06 (d, 1H, J = 3.5 Hz), 4.76 (d, 1H, J = 11.9 Hz), 4.54 (d, 1H, J = 11.9 Hz), 4.33 (dd, 1H, J = 5.1, 10.2 Hz), 4.23 (dt, 1H, J = 5.1, 10.0 Hz), 3.82 (t, 1H, J = 10.0 Hz), 3.69 (d, 1H, J = 9.6 Hz), 3.63 (s, 1H), 3.16 (dd, 1H, J = 2.7, 4.1 Hz), 2.90 (dd, 1H, J = 2.7, 5.0 Hz), 2.78 (dd, 1H, J = 4.1, 5.0 Hz), 1.99 (dd, 1H, J = 1.3, 14.7 Hz), 1.91 (dd, 1H, J = 4.0, 14.7 Hz) ppm; **^{13}C-NMR** (125 MHz, $CDCl_3$) δ 137.4, 136.7, 129.1, 128.7, 128.3, 128.3, 128.2, 126.3, 102.0, 96.6, 80.6, 69.8, 69.4, 68.9, 59.6, 54.3, 43.8, 35.8 ppm; **HRMS** ESI m/z [M + Na]$^+$ calcd. for $C_{22}H_{24}O_6Na$ 407.14651,

found 407.14562. **23**: **R$_f$** = 0.32 (hexanes/EtOAc 2:1); **mp** 120.6 °C; $[\alpha]_D^{20}$ +58.7° (c 0.6 in $CHCl_3$); **IR** v_{max}/cm^{-1} 3506 (br. w), 3067 (w), 3035 (w), 2975 (w), 2931 (w), 2864 (w), 1455 (m), 1386 (w), 1119 (s), 1096 (s), 1025 (s) 911 (m); **^{1}H-NMR** (500 MHz, $CDCl_3$) δ 7.49 (m, 2H), 7.40-7.16 (m, 8H), 5.63 (s, 1H), 4.99 (d, 1H, J = 4.4 Hz), 4.77 (d, 1H, J = 12.2 Hz), 4.58 (d, 1H, J = 12.2 Hz), 4.28 (m, 2H), 3.77 (m, 1H), 3.62 (m, 1H), 3.23 (s, 1H), 3.02 (dd, 1H, J = 2.7, 4.1 Hz), 2.90 (dd, 1H, J = 2.7, 5.2 Hz), 2.69 (dd, 1H, J = 4.1, 5.2 Hz), 2.04 (dd, 1H, J = 1.1, 14.9 Hz), 1.97 (dd, 1H, J = 0.8, 14.9 Hz) ppm; **^{13}C-NMR** (125 MHz, $CDCl_3$) δ 137.3, 137.2, 129.1, 128.7, 128.4, 128.3, 128.1, 126.2, 101.7, 95.8, 80.5, 69.6, 69.3, 68.5, 59.2, 55.9, 43.7, 37.2 ppm; **HRMS** ESI m/z [M + Na]$^+$ calcd. for $C_{22}H_{24}O_6Na$ 407.14651, found 407.14557.

(2*R*,4a*R*,6*S*,8*R*,8a*R*)-8-((*S*)-1-Azidoethyl)-6-(benzyloxy)-2-phenylhexahydropyrano[3,2-*d*][1,3]dioxin-8-ol (7)

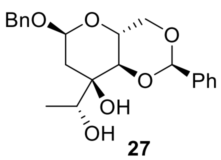

27

LiAlH$_4$ (128 mg, 3.38 mmol, 2.00 eq.) was suspended in dry THF (14 mL) at 0 °C under argon atmosphere and epoxide **24** (649 mg, 1.69 mmol, 1.00 eq) in dry THF (20 mL) was added dropwise. The solution was stirred at 0 °C for 30 min and at room temperature for 2 h. AcMe (1.7 mL) was added, the solution stirred for 5 min, poured into a mixture of EtOAc (20 mL) and sat. aq. Na,K-tartrate solution (300 mL) and stirred for 2 h. The aqueous phase was separated and extracted with EtOAc (3×100 mL). The organic phases were washed with brine (100 mL), dried over Na_2SO_4 and the solvent removed in vacuo. Alcohol **27** (669 mg, quant.) was obtained as colourless resin and used without further purification in the next step. **R$_f$** = 0.55 (hexanes/EtOAc 1:1); $[\alpha]_D^{20}$ +81.5° (c 0.9 in $CHCl_3$); **IR** v_{max}/cm^{-1} 3500 (br. w), 3067 (w), 3032 (w), 2971 (w), 2934 (w), 2873 (w), 1455 (m), 1397 (m), 1095 (s), 1078 (s), 1014 (s); **^{1}H-NMR** (500 MHz, $CDCl_3$) δ 7.49 (m, 2H), 7.40-7.12 (m, 8H), 5.62 (s, 1H), 5.06 (t, 1H, J = 2.7 Hz), 4.77 (d, 1H, J = 12.0 Hz), 4.55 (d, 1H, J = 12.0 Hz), 4.31 (dd, 1H, J = 5.1, 10.0 Hz), 4.24 (dt, 1H, J = 5.1, 9.8 Hz), 3.94 (qn, 1H, J = 6.4 Hz), 3.87 (d, 1H, J = 9.6 Hz), 3.79 (t, 1H, J = 10.1 Hz), 3.64 (s, 1H), 1.98 (m, 2H), 1.78 (m, 1H), 1.25 (d, 3H, J = 6.5 Hz) ppm; **^{13}C-NMR** (125 MHz, $CDCl_3$) δ 137.5, 137.0, 129.2, 128.7, 128.4, 128.3, 128.2, 126.3, 101.9, 97.0, 79.0, 72.3, 69.7, 69.5, 69.0, 59.6, 34.1, 17.5 ppm; **HRMS** ESI m/z [M + Na]$^+$ calcd. for $C_{22}H_{26}O_6Na$ 409.16216, found 409.16121.

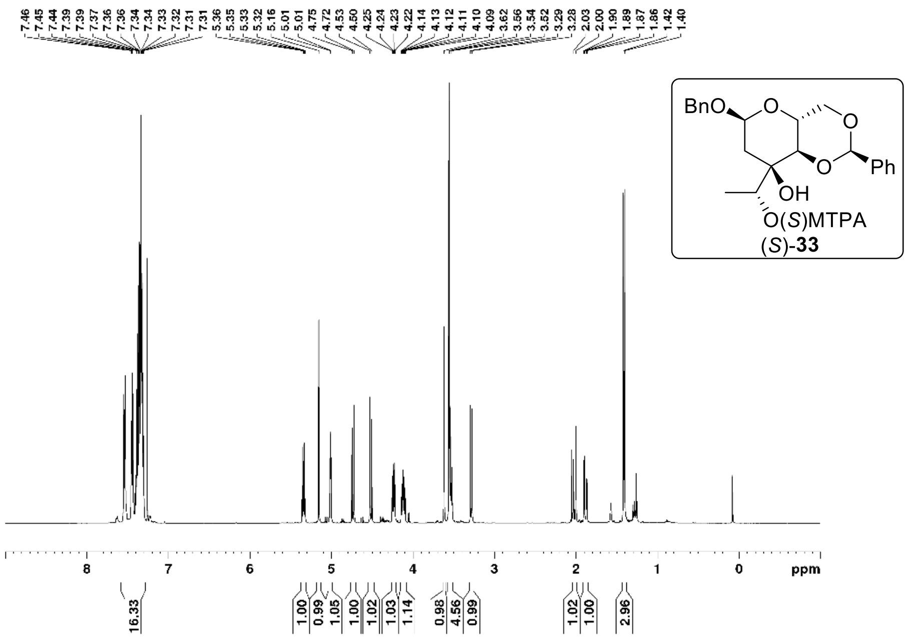

Fig. S1. ^{1}H-NMR-spectrum of (*S*)-**33**. (*S*)-Mosher ester of **27**.

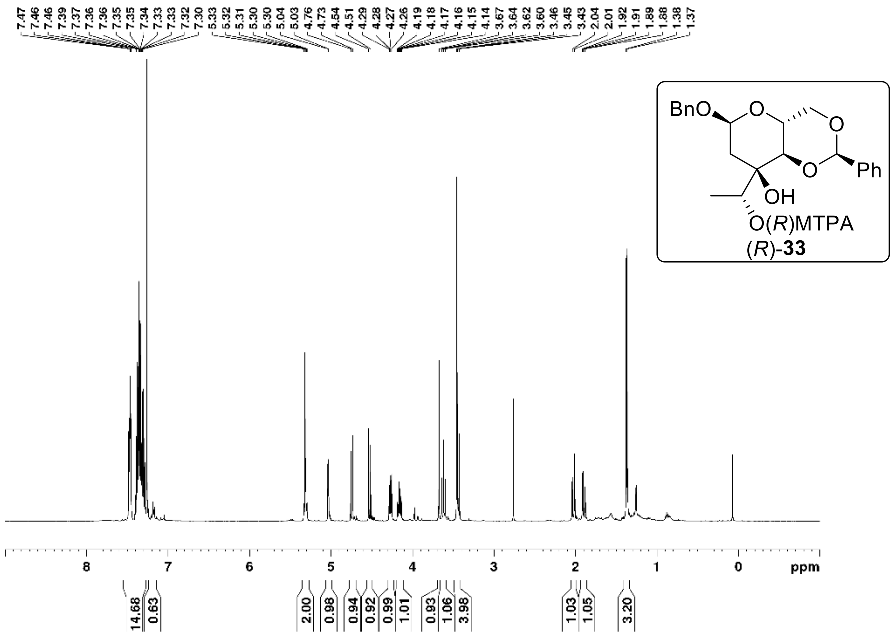

Fig. S2. ^{1}H-NMR-spectrum of (*R*)-**33**. (*R*)-Mosher ester of **27**.

The stereogenic centre of the secondary alcohol in **27** was determined by Mosher ester method. Comparison of the ^{1}H-NMR-spectra of (*S*)-**33** (fig. S1) and (*R*)-**33** (fig. S1) indicated the secondary alcohol to be (*R*)-configurated. Exact $\Delta\delta^{SR} = \delta^S - \delta^R$–values are shown in Figure 1 (main manuscript). The stereogenic determination was made by standard procedure.

To a solution of diol **27** (654 mg, 1.69 mmol, 1.00 eq.) and dest. dry NEt_3 (1.06 mL, 7.61 mmol, 4.50 eq.) in dry CH_2Cl_2 (16.9 mL) under argon atmosphere was added $SOCl_2$ (307 µL, 4.23 mmol, 2.50 eq.) at 0 °C. The solution was stirred at 0 °C for 3 h and sat aq. NH_4Cl solution (25 mL) was mixed by. The aqueous phase was extracted with EtOAc (4×25 mL) and the combined organic phases were washed with sat. aq. NH_4Cl solution (2×20 mL), sat. aq. $NaHCO_3$ solution (20 mL) and brine (20 mL). The solution was dried over Na_2SO_4, evaporated and the raw sulfite **28** (774 mg, quant.) used without purification. **R$_f$** = 0.50 (hexanes/EtOAc 1:1); $[\alpha]_D^{20}$ −10.8° (c 1.0 in $CHCl_3$); **IR** ν_{max}/cm^{-1} 3065 (w), 3030 (w), 2980 (w), 2932 (w), 2870 (w), 1455 (m), 1386 (m), 1207 (s) 1101 (s), 1026 (s), 911 (s), 878 (s); **^{1}H-NMR** (500 MHz, $CDCl_3$) *δ* 7.65-7.27 (m, 10H), 5.64 (s, 0.28H), 5.58 (s, 0.72H), 4.98 (m, 1H), 4.78 (m, 1H), 4.69 (q, 0.75H, *J* = 6.5 Hz) 4.56 (m, 1H), 4.37-4.27 (m, 1.30H), 4.23 (m, 1H), 3.80-3.66 (m, 1.58H), 3.58 (d, 0.73H, *J* = 9.4 Hz), 2.29 (d, 0.73H, *J* = 14.8 Hz), 2.10 (m, 1H), 1.95 (dd, 0.29H, *J* = 4.7, 14.8 Hz), 1.61 (d, 0.81H, *J* = 6.6 Hz), 1.55 (d, 2.13H, *J* = 6.5 Hz) ppm; major diastereomer: **^{13}C-NMR** (125 MHz, $CDCl_3$) *δ* 137.5, 137.0, 129.3, 128.6, 128.4, 127.8, 127.8, 126.1, 101.1, 94.8, 87.5, 80.9, 77.0, 69.4, 69.2, 58.7, 37.3, 13.7 ppm; minor diastereomer: **^{13}C-NMR** (125 MHz, $CDCl_3$) *δ* 137.4, 137.1, 129.1, 128.6, 128.4, 128.0, 128.0, 126.7, 101.9, 94.9, 85.0, 85.0, 77.1, 69.5, 69.3, 59.6, 39.4, 16.1 ppm; **HRMS** ESI *m/z* [M + Na]$^+$ calcd. for $C_{22}H_{24}O_7SNa$ 455.11349, found 455.11272.

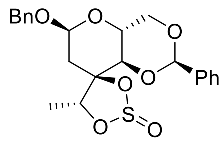

28

The sulfite **28** (724 mg, 1.58 mmol, 1.00 eq.) was solved in MeCN (9 mL)/H_2O (4.5 mL) at room temperature and $NaIO_4$ (355 mg, 1.66 mmol, 1.05 eq.) and $RuCl_3 \cdot xH_2O$ (16 mg, 79.0 µmol, 5 mol%) were added. The mixture was stirred at room temperature for 7 h, sat. aq. $Na_2S_2O_3$ solution (40 mL) was added and extracted with EtOAc (3×50 mL). The combined organic phases were washed with brine (50 mL), dried over Na_2SO_4, and evaporated. The crude sulfate **29** (678 mg, 96%) was pure enough for the next step without purification. **R$_f$** = 0.34 (hexanes/EtOAc 1:1); $[\alpha]_D^{20}$ +74.1° (c 1.0 in $CHCl_3$); **IR** ν_{max}/cm^{-1} 3069 (w), 3033 (w), 2926 (w), 2871 (w), 1455 (m), 1380 (s), 1208 (s), 1130 (m), 1105 (s), 1026 (s); **^{1}H-NMR** (500 MHz, $CDCl_3$) *δ* 7.55 (m, 2H), 7.41-7.27 (m, 8H), 5.62 (s, 1H), 4.97 (d, 1H, *J* = 4.7 Hz), 4.75 (d, 1H, *J* = 12.4 Hz), 4.71 (q, 1H, *J* = 6.5 Hz), 4.54 (d, 1H, *J* = 12.4 Hz), 4.30 (dt, 1H, *J* = 5.2, 9.9 Hz), 4.23 (dd, 1H, *J* = 5.2, 10.4 Hz), 3.74 (t, 1H, *J* = 10.4 Hz), 3.71 (d, 1H, *J* = 9.9 Hz), 2.33 (d, 1H, *J* = 15.1 Hz), 1.98 (dd, 1H, *J* = 4.7, 15.1 Hz), 1.25 (d, 3H, *J* = 6.5 Hz) ppm; **^{13}C-NMR** (125 MHz, $CDCl_3$) *δ* 137.2, 136.7, 129.4, 128.6, 128.5, 128.0, 127.9, 126.4, 101.7, 94.5, 88.6, 83.7,

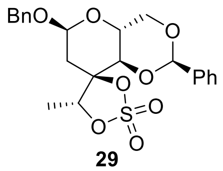

29

77.0, 69.5, 69.4, 69.1, 58.8, 37.5, 13.6 ppm; **HRMS** ESI *m/z* $[M + H]^+$ calcd. for $C_{22}H_{25}O_8S$ 449.12646, found 449.12551.

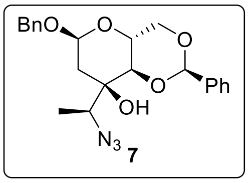

A solution of sulfate **29** (640 mg, 1.43 mmol, 1.00 eq.) in dry DMF (7.1 mL) under argon atmosphere was treated with NaN_3 (464 mg, 7.14 mmol, 5.00 eq.) and stirred at 65 °C for 6.75 h. The resulting sodium sulfate was hydrolyzed by adding pH 4.5 citrate-buffer (50 mL) and EtOAc (20 mL) and stirring at 45 °C for 15 h. Further citric acid (5 g) was added and stirring at 45 °C was continued for 3.5 h. The mixture was extracted with EtOAc (4×50 mL) and the combined organic phases were washed with sat. aq. $NaHCO_3$ solution (50 mL), H_2O (50 mL) and brine (50 mL), dried over Na_2SO_4 and concentrated. Column chromatography (SiO_2, pentane/EtOAc 6:1) led to azide **7** (370 mg, 63%; 61% over 4 steps) as colourless solid. $\mathbf{R_f}$ = 0.38 (hexanes/EtOAc 4:1); **mp** 86.3 °C; $[\alpha]_D^{20}$ +106.3° (c 1.0 in $CHCl_3$); **IR** ν_{max}/cm^{-1} 3504 (br. m), 3069 (w), 3037 (w), 2980 (w), 2934 (w), 2872 (w), 2092 (br. s), 1455 (m), 1402 (m), 1264 (m), 1117 (s), 1096 (s), 1019 (s); **^{1}H-NMR** (500 MHz, $CDCl_3$) δ 7.51 (m, 2H), 7.41-7.18 (m, 8H), 5.59 (s, 1H), 5.09 (d, 1H, *J* = 3.8 Hz), 4.78 (d, 1H, *J* = 11.9 Hz), 4.56 (d, 1H, *J* = 11.9 Hz), 4.34 (dd, 1H, *J* = 5.1, 10.2 Hz), 4.22 (dt, 1H, *J* = 5.1, 9.8 Hz), 4.08 (s, 1H), 3.84 (q, 1H, *J* = 6.9 Hz), 3.80 (t, 1H, *J* = 10.2 Hz), 3.64 (d, 1H, *J* = 9.5 Hz), 2.06 (d, 1H, *J* = 14.8 Hz), 1.94 (dd, 1H, *J* = 4.0, 14.8 Hz), 1.27 (d, 3H, *J* = 6.9 Hz) ppm; **^{13}C-NMR** (125 MHz, $CDCl_3$) δ 137.3, 136.6, 129.2, 128.8, 128.4 (2 signals), 128.3, 126.3, 101.9, 97.0, 79.9, 73.8, 69.9, 69.4, 62.4, 59.7, 35.0, 15.0 ppm; **HRMS** ESI *m/z* $[M + Na]^+$ calcd. for $C_{22}H_{25}O_5N_3Na$ 434.16864, found 434.16775.

(2*R*,4a*R*,6*S*,8*R*,8a*R*)-6-(Benzyloxy)-8-((*S*)-1-hydroxyethyl)-2-phenylhexahydropyrano-[3,2-*d*][1,3]dioxin-8-ol (25)

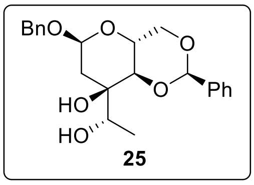

Epoxide **23** (475 mg, 1.24 mmol, 1.00 eq.) in dry THF (5 mL) was added to a suspension of $LiAlH_4$ (93.7 mg, 2.47 mmol, 2.00 eq.) in dry THF (20 mL) under argon atmosphere at 0 °C. The solution was stirred at 0 °C for 5 min and at room temperature for 1.75 h. EtOAc (15 mL) was added, the mixture stirred for 5 min and poured into Na,K-tartrate solution (150 mL). After stirring for 40 min the mixture was extracted with EtOAc (3×75 mL). The combined organic phases were washed with brine (75 mL), dried over Na_2SO_4 and evaporated. After column chromatography (SiO_2, pentane/EtOAc 4:1) the diol **25** (462 mg, 97%) was obtained as colourless resin. $\mathbf{R_f}$ =

0.46 (hexanes/EtOAc 1:1); $[\alpha]_D^{20}$ +121.8° (c 1.0 in $CHCl_3$); **IR** ν_{max}/cm^{-1} 3499 (br. m), 3033 (w), 2975 (w), 2934 (w), 2871 (w), 1455 (m), 1397 (m), 1101 (s), 1018 (s); **^{1}H-NMR** (500 MHz, $CDCl_3$) δ 7.47 (m, 2H), 7.39-7.16 (m, 8H), 5.59 (s, 1H), 5.07 (d, 1H, J = 3.8 Hz), 4.78 (d, 1H, J = 12.0 Hz), 4.56 (d, 1H, J = 12.0 Hz), 4.31 (dd, 1H, J = 5.1, 10.2 Hz), 4.24 (dt, 1H, J = 5.1, 9.8 Hz), 4.06 (q, 1H, J = 6.5 Hz), 3.92 (s, 1H), 3.78 (t, 1H, J = 10.1 Hz), 3.64 (d, 1H, J = 9.4 Hz), 2.74 (s, 1H), 2.08 (dd, 1H, J = 1.0, 14.7 Hz), 1.82 (dd, 1H, J = 4.2, 14.7 Hz), 1.25 (d, 3H, J = 6.5 Hz) ppm; **^{13}C-NMR** (125 MHz, $CDCl_3$) δ 137.2, 136.8, 129.3, 128.7, 128.4, 128.3 (2 signals), 126.3, 102.0, 96.9, 81.3, 72.9, 70.2, 69.8, 69.5, 59.6, 34.0, 16.0 ppm; **HRMS** ESI *m/z* [M + Na$^+$] calcd. for $C_{22}H_{26}O_6Na$ 409.16216, found 409.16120.

(2R,2'R,3R,4a'R,6'S,8a'R)-6'-(Benzyloxy)-3-methyl-2'-phenyltetrahydro-4'H-spiro-[oxirane-2,8'-pyrano[3,2-d][1,3]dioxine] (26)

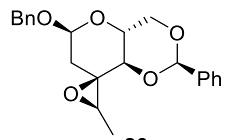

26

To a solution of diol **25** (100 mg, 259 µmol, 1.00 eq.) in dry CH_2Cl_2 (2 mL) and pyridine (200 µL) under argon atmosphere at −78 °C was added Tf_2O (87.1 µL, 518 µmol, 2.00 eq.). The solution was stirred at 0 °C for 1.25 h. Sat. aq. $NaHCO_3$ solution(20 mL) and $NaHCO_3$ (solid, 1 g) was mixed by and stirred for 30 min at room temperature. The emulsion was extracted with CH_2Cl_2 (3×20 mL). After washing the combined organic phases with H_2O (20 mL) and brine (20 mL), they were dried over Na_2SO_4 and solvent was removed in vacuo. The pinkish white solid (105 mg, quant.) was used without further purification. **R$_f$** = 0.85 (hexanes/EtOAc 1:1); **mp** 142 °C; $[\alpha]_D^{20}$ +96.0° (c 1.0 in $CHCl_3$); **IR** ν_{max}/cm^{-1} 3067 (w), 3032 (w), 2968 (w), 2927 (w), 2864 (w), 1454 (m), 1384 (m), 1126 (s), 1095 (s), 1022 (s); **^{1}H-NMR** (500 MHz, $CDCl_3$) δ 7.47-7.26 (m, 10H), 5.58 (s, 1H), 4.98 (d, 1H, J = 4.2 Hz), 4.78 (d, 1H, J = 12.3 Hz), 4.57 (d, 1H, J = 12.3 Hz), 4.30 (dt, 1H, J = 5.0, 9.9 Hz), 4.24 (d, 1H, J = 5.0, 10.3 Hz), 4.05 (d, 1H, J = 9.5 Hz), 3.77 (t, 1H, J = 10.3 Hz), 2.86 (q, 1H, J = 5.7 Hz), 2.37 (dd, 1H, J = 4.2, 14.8 Hz), 1.60 (dd, 1H, J = 0.7, 14.8 Hz), 1.54 (d, 3H, J = 5.7 Hz) ppm; **^{13}C-NMR** (125 MHz, $CDCl_3$) δ 137.6, 137.4, 129.0, 128.5, 128.3, 128.1, 127.8, 126.3, 101.7, 96.0, 69.8, 69.2, 61.8, 58.8, 58.3, 38.7, 14.1 ppm; **HRMS** ESI *m/z* [M + K$^+$] calcd. for $C_{22}H_{24}O_5K$ 407.12553, found 407.12479.

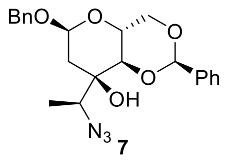

7

Half of the crude epoxide **26** (52.5 mg, 129 µmol, 1.00 eq.) was suspended in MeOH (1.2 mL)/H_2O (300 µL) and treated with NaN_3 (33.5 mg, 516 µmol, 4.00 eq.) and NH_4Cl (13.8 mg, 258 µmol, 2.00 eq.). The mixture was heated at 80 °C for 12 h. The volatile components were

removed by rotary evaporation and the remainder dissolved in EtOAc (15 mL)/H_2O (15 mL). The aqueous phase was separated and extracted with EtOAc (2×10 mL). The combined organic phases were washed with brine (15 mL), dried over Na_2SO_4 and evaporated. The crude azide **7** was chromatographed (SiO_2, pentane/EtOAc 3:1) and the pure compound (43 mg, 81%) was obtained as colourless solid. For analytical data see prior performed synthesis of azide **7**.

(2*R*,3*R*,4*R*,6*S*)-4-((*S*)-1-Azidoethyl)-6-(benzyloxy)-4-hydroxy-2-methyltetrahydro-2*H*-pyran-3-yl benzoate (30)

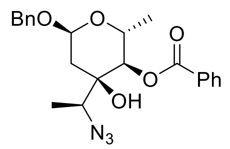

30

Azide **7** (360 mg, 875 µmol, 1.00 eq.) was placed in a sealed vessel with TIPST (187 µL, 875 µmol, 1.00 eq), DTBP (81.1 µL, 438 µmol, 0.50 eq.) and degassed *n*-octane (18 mL) under argon atmosphere. The solution was heated at 140 °C for 6.75 h, the solvent was removed in vacuo and the remainder was chromatographed (SiO_2, pentane/EtOAc 15:1 to 12:1). The ester **30** (179 mg, 50%) was obtained as colourless solid. **R$_f$** = 0.59 (hexanes/EtOAc 4:1); **mp** 90.1 °C; $[\alpha]_D^{20}$ +111.7° (c 1.0 in $CHCl_3$); **IR** ν_{max}/cm^{-1} 3492 (br. m), 2981 (w), 2937 (w), 2912 (w), 2093 (s), 1721 (s), 1453 (m), 1267 (s), 1113 (s), 1027 (w); **^{1}H-NMR** (500 MHz, $CDCl_3$) δ 8.11 (m, 2H), 7.60 (tt, 1H, *J* = 1.3, 7.4 Hz), 7.47 (m, 2H), 7.42-7.30 (m, 5H), 5.13 (d, 1H, *J* = 3.8 Hz), 5.01 (d, 1H, *J* = 9.7 Hz), 4.78 (d, 1H, *J* = 11.9 Hz), 4.57 (d, 1H, *J* = 11.9 Hz), 4.40 (s, 1H), 4.24 (dq, 1H, *J* = 6.4, 9.7 Hz), 3.60 (q, 1H, *J* = 6.9 Hz), 2.16 (dd, 1H, *J* = 1.0, 14.6 Hz), 1.87 (dd, 1H, *J* = 4.0, 14.6 Hz), 1.22 (d, 3H, *J* = 6.3 Hz), 1.15 (d, 3H, *J* = 6.9 Hz) ppm; **^{13}C-NMR** (125 MHz, $CDCl_3$) δ 166.1, 136.7, 133.7, 130.1, 129.5, 128.8, 128.7, 128.4, 128.3, 96.7, 75.7, 74.4, 69.9, 63.5, 62.1, 34.0, 17.5, 15.0 ppm; **HRMS** ESI *m/z* [M + Na]$^+$ calcd. for $C_{22}H_{25}O_6N_3Na$ 434.16864, found 434.16795.

(2*R*,3*R*,4*R*,6*S*)-4-((*S*)-1-Aminoethyl)-6-(benzyloxy)-2-methyltetrahydro-2*H*-pyran-3,4-diol (6)

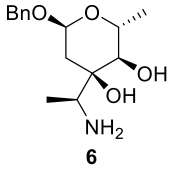

6

To a suspension of $LiAlH_4$ (22 mg, 583 µmol, 3.00 eq.) in dry THF (4 mL) under argon atmosphere at 0 °C was added dropwise ester **30** (80 mg, 194 µmol, 1.00 eq.). The solution was stirred at 0 °C for 7 h and further 17 h at room temperature. EtOAc (1 mL) was mixed by, stirred for 5 min and poured into sat. aq. Na,K-tartrate solution (10 mL). The suspension was stirred further 2 h and extracted with EtOAc (3×40 mL). The combined organic phases were washed with brine, dried

over Na_2SO_4, and evaporated. After column chromatography (SiO_2, CH_2Cl_2/MeOH+0.5% NEt_3 30:1 to 4:1) amine **6** (43 mg, 79%) was obtained as colourless resin. **R_f** = 0.11 (CH_2Cl_2/MeOH 4:1); $[\alpha]_D^{20}$ +108.3° (c 1.0 in $CHCl_3$); **IR** ν_{max}/cm^{-1} 3500-2500 (m), 3031 (m), 2970 (m), 2931 (m), 1735 (w), 1586 (m), 1455 (m), 1379 (m), 1258 (m), 1126 (s), 1064 (s), 1019 (s); **^{1}H-NMR** (500 MHz, $CDCl_3$) δ 7.36-7.26 (m, 5H), 4.97 (d, 1H, J = 3.8 Hz), 4.71 (d, 1H, J = 11.8 Hz), 4.46 (d, 1H, J = 11.9 Hz), 4.09 (br. s, 4H), 3.85 (dq, 1H, J = 6.2, 9.4 Hz), 3.32 (d, 1H, J = 9.5 Hz), 3.05 (q, 1H, J = 6.5 Hz), 1.96 (dd, 1H, J = 0.8, 14.5 Hz), 1.57 (dd, 1H, J = 4.0, 14.5 Hz), 1.31 (d, 3H, J = 6.2 Hz), 1.12 (d, 3H, J = 6.5 Hz) ppm; **^{1}H-NMR** (500 MHz, CD_3OD) δ 7.42-7.26 (m, 5H), 5.03 (d, 1H, J = 3.8 Hz), 4.71 (d, 1H, J = 11.8 Hz), 4.51 (d, 1H, J = 11.9 Hz), 3.88 (dq, 1H, J = 6.3, 9.5 Hz), 3.23 (d, 1H, J = 9.5 Hz), 3.18 (q, 1H, J = 6.7 Hz), 1.93 (dd, 1H, J = 1.1, 14.5 Hz), 1.70 (dd, 1H, J = 4.0, 14.5 Hz), 1.27 (d, 3H, J = 6.3 Hz), 1.08 (d, 3H, J = 6.8 Hz) ppm; **^{13}C-NMR** (125 MHz, $CDCl_3$) δ 137.0, 128.6, 128.2, 128.1, 96.4, 77.6, 71.7, 69.3, 65.0, 54.3, 36.0, 18.3, 17.8 ppm; **^{13}C-NMR** (125 MHz, CD_3OD) δ 138.7, 129.5, 129.2, 129.0, 98.0, 75.9, 74.5, 70.4, 66.1, 52.6, 34.4, 18.2, 16.4 ppm; **HRMS** ESI m/z [M + H]$^+$ calcd. for $C_{15}H_{24}O_4N$ 282.16998, found 282.16969.

N-((*S*)-1-((2*R*,3*R*,4*R*,6*S*)-6-(Benzyloxy)-3,4-dihydroxy-2-methyltetrahydro-2*H*-pyran-4-yl)ethyl)-3,4-dichloro-5-methyl-1*H*-pyrrole-2-carboxamide (32)

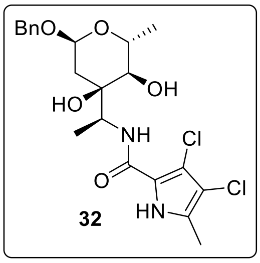

A solution of amine **6** (45 mg, 160 µmol, 1.00 eq.), carboxylic acid **31** (38.8 mg, 200 µmol, 1.25 eq.), HOBt (30.6 mg, 200 µmol, 1.25 eq.) and dry NEt_3 (55.8 µL, 400 µmol, 2.50 eq.) in dry CH_2Cl_2 (2 mL) was treated with EDC·HCl (61.3 mg, 320 µmol, 2.00 eq.) at 0 °C under argon atmosphere. The solution was slowly warmed to room temperature over 3 h and stirred further 13 h at room temperature. The reaction was quenched with 1M HCl (2 mL) and poured into a mixture of EtOAc (40 mL) and 1M HCl (40 mL). The organic phase was separated, and the aqueous phase extracted with EtOAc (2×40 mL). The combined organic phases were washed with 1M HCl (40 mL), sat. aq. $NaHCO_3$ solution (2×40 mL) and brine (40 mL). After drying over Na_2SO_4, the organic phase was evaporated and chromatographed (SiO_2, CH_2Cl_2/MeOH 100:1 to 40:1). The amide **32** (61 mg, 83%) was obtained as a reddish solid foam. **R_f** = 0.74 (CH_2Cl_2/MeOH 9:1); **mp** 68.6 °C; $[\alpha]_D^{20}$ +90.5° (c 1.0 in $CHCl_3$); **IR** ν_{max}/cm^{-1} 3412 (br. m), 3208 (br. m), 2976 (w), 2933 (m), 1629 (s), 1532 (s), 1455 (m), 1413 (m), 1272 (m), 1126 (m), 1047 (s), 1023 (m), 759 (m); **^{1}H-NMR** (500 MHz, $CDCl_3$) δ 11.00, (s, 1H), 7.40-7.28 (m, 5H), 6.93 (d, 1H, J = 8.8 Hz), 5.04 (d, 1H, J = 3.4 Hz), 4.72 (d, 1H, J =

11.8 Hz), 4.50 (d, 1H, J = 11.9 Hz), 4.46 (m, 1H), 4.16 (s, 1H), 3.76 (dq, 1H, J = 6.2, 9.4 Hz), 3.27 (d, 1H, J = 9.3 Hz), 2.47 (br. s, 1H), 2.25 (s, 3H), 2.02 (d, 1H, J = 14.4 Hz), 1.86 (dd, 1H, J = 3.9, 14.4 Hz), 1.34 (d, 3H, J = 6.2 Hz), 1.26 (d, 3H, J = 6.9 Hz) ppm; **^{13}C-NMR** (125 MHz, $CDCl_3$) δ 159.6, 136.7, 128.7, 128.5, 128.3, 128.2, 118.5, 111.0, 110.1, 96.3, 74.3, 73.5, 69.6, 65.7, 50.5, 35.2, 18.0, 16.4, 11.2 ppm; **HRMS** ESI m/z [M + Na]$^+$ calcd. for $C_{21}H_{26}O_5N_2Cl_2Na$ 479.11110, found 479.11029.

3,4-Dichloro-5-methyl-*N*-((1*S*)-1-((2*R*,3*R*,4*R*)-3,4,6-trihydroxy-2-methyltetrahydro-2*H*-pyran-4-yl)ethyl)-1*H*-pyrrole-2-carboxamide (4)

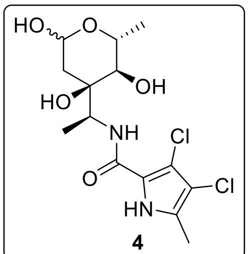

4

To a solution of amide **32** (20 mg, 43.7 µmol, 1.00 eq.) in dry CH_2Cl_2 (2 mL) under argon atmosphere was added BCl_3 (1 M CH_2Cl_2, 219 µL, 219 µmol, 5.00 eq.) at −80 °C. The solution was stirred at −80 °C for 40 min and a few drops of H_2O were added. The emulsion was evaporated to dryness and chromatographed (SiO_2, CH_2Cl_2/MeOH 40:1 to 15:1). The anomeric mixture of amycolose derivative **4** (13 mg, 81%) was obtained as colourless resin. **R_f** = 0.35, 0.42 (CH_2Cl_2/MeOH 9:1); **IR** ν_{max}/cm^{-1} 3668-3028 (br. m), 2976 (w), 2932 (m), 1758 (w), 1706 (m), 1627 (s), 1536 (s), 1416 (m), 1377 (m), 1269 (m), 1067 (s), 1001 (m), 803 (w), 764 (w); **^{1}H-NMR** (500 MHz, $CDCl_3$) δ 9.62 (s, 0.83H), 9.56 (s, 0.19H), 6.65 (d, 1H, J = 6.5 Hz), 6.21 (br. s, 0.72H), 5.64 (br. s, 0.64H), 5.23 (d, 0.81H, J = 3.5 Hz), 5.15 (dd, 0.19H, J = 2.1, 9.3 Hz), 4.41 (qn, 1H, J = 6.8 Hz), 4.00 (dq, 0.82H, J = 6.2, 9.3 Hz), 3.69 (dq, 0.19H, J = 6.3, 9.2 Hz), 3.19 (d, 0.74H, J = 9.3 Hz), 3.17 (d, 0.26H, J = 9.1 Hz), 2.94-1.53 (m, 6.78H), 2.29 (s, 2.23H), 2.28 (s, 0.78H), 1.99 (dd, 0.24H, J = 2.3, 13.3 Hz), 1.95 (d, 0.95H, J = 1.0, 13.9 Hz), 1.70 (dd, 0.87H, J = 3.9, 13.9 Hz), 1.46 (dd, 0.26H, J = 9.3, 13.0 Hz), 1.34 (d, 3H, J = 6.2 Hz), 1.31 (d, 3H, J = 7.0 Hz) ppm; **^{1}H-NMR** (500 MHz, CD_3OD) δ 5.21 (m, 0.75H), 5.05 (d, 0.31H, J = 2.1, 9.5 Hz), 4.37 (m, 1H), 4.05 (dq, 0.68H, J = 6.2, 9.4 Hz), 3.73 (dq, 0.30H, J = 6.2, 9.2 Hz), 3.21 (d, 0.73H, J = 9.4 Hz), 3.17 (d, 0.33H, J = 9.3 Hz), 2.23 (s, 3H), 1.90 (dd, 0.73H, J = 1.4, 14.1 Hz), 1.88 (dd, 0.27H, J = 2.1, 13.3 Hz), 1.80 (dd, 0.73H, J = 3.9, 14.1 Hz), 1.53 (dd, 0.31H, J = 9.5, 13.3 Hz), 1.26 (m, 6H) ppm; **^{13}C-NMR** (125 MHz, $CDCl_3$) δ 161.7, 161.5, 129.3, 129.1, 117.4, 117.3, 112.6, 112.4, 111.2, 111.1, 92.9, 92.2, 77.2, 76.3, 74.1, 74.0, 70.8, 64.7, 52.6, 52.5, 37.5, 33.6, 18.2, 18.1, 16.3, 11.5, 11.5 ppm; **^{13}C-NMR** (125 MHz, CD_3OD) δ 161.7, 161.6, 129.4, 129.4, 120.0, 120.0, 112.3, 112.2, 110.6, 110.6, 93.5, 92.9, 76.3, 76.1, 75.0, 74.7, 71.6, 65.8, 52.3, 52.0, 39.2, 35.5, 18.6, 18.5, 16.2, 10.8 ppm; **HRMS** ESI m/z [M + Na]$^+$ calcd. for $C_{14}H_{20}O_5N_2Cl_2Na$ 389.06415, found 389.06320.

α-/β-Anomeric ratio and signal form of O*H*-groups in ^{1}H-NMR-spectra depends on the purification method as well as solvent and pH.

Spectroscopic data corresponded to those reported in the literature.[2]

Trace impurities in the NMR-spectra of the compounds in the amycolose-sequence can result from the formation of different α-/β-anomers best observed in the ^{1}H-NMR at the anomeric and benzylic position as shown below (fig. S3). The amount of the wrong anomer in the synthesis sequence depends on the purity of the benzyl α-D-mannopyranoside (**9**) as starting material but has no influence on the (diastereoselective) reactions.

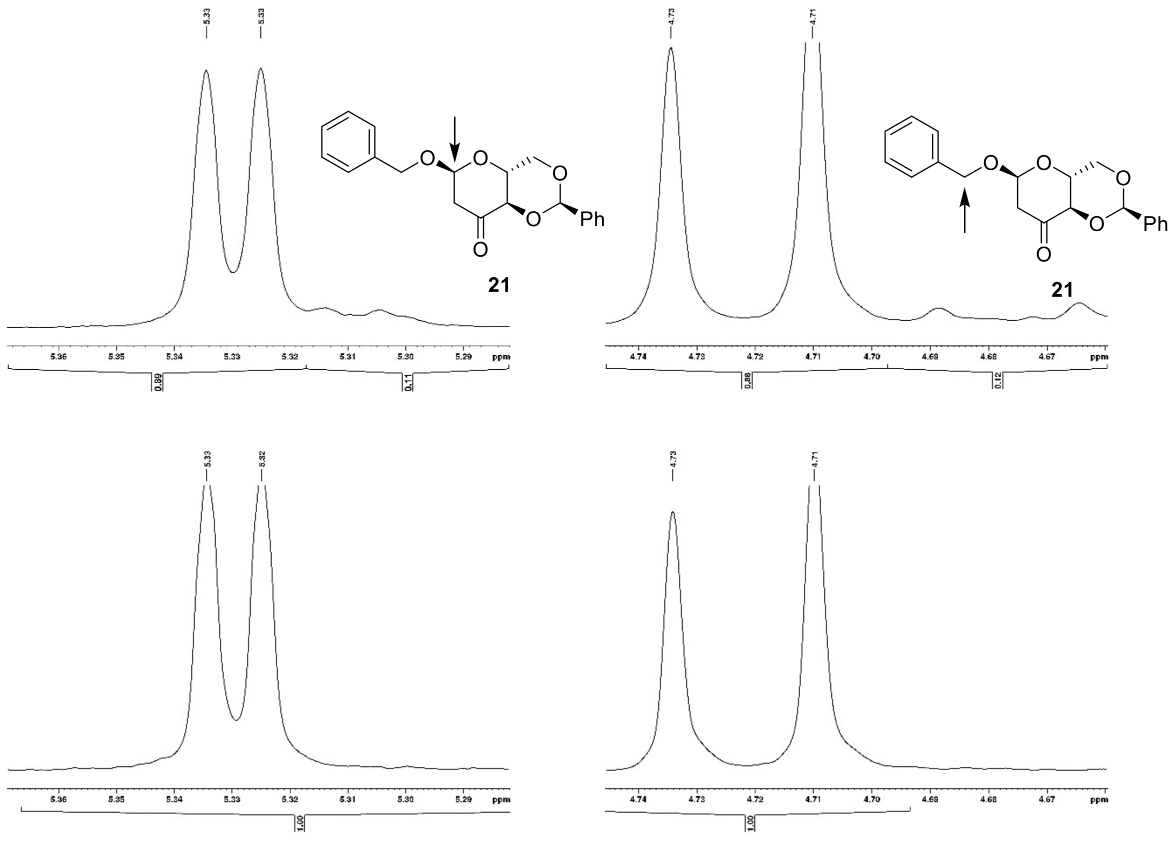

Fig. S3. Comparison of the anomeric (left) and benzylic (right) position of ketone **21** in the ^{1}H-NMR-spectra with different pure starting materials. The upper spectra show a α/β-ratio of ca. 9:1, while the others show 100% α.

2.2 Synthesis of pyrrole carboxylic acid **31**

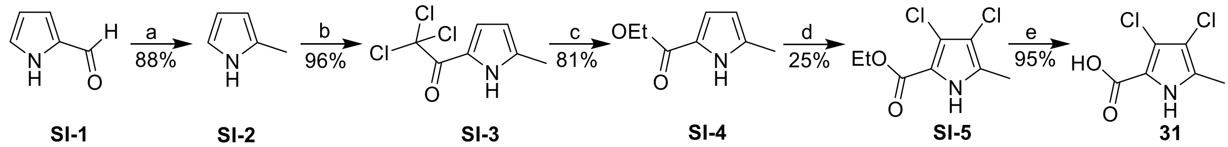

Scheme S2. Synthesis of pyrrole carbonic acid **31**.
Reagents and conditions: a) NaOH, ethylene glycol, N_2H_4·xH_2O, 210 °C, 2.5 h; b) trichloroacetyl chloride, THF, 0 °C, 16 h; c) Na, EtOH, rt, 35 min; d) SO_2Cl_2, CH_2Cl_2, 0 °C, 3.5 h; e) NaOH, H_2O/MeOH, rt, 22 h.

The route is also possible with a methyl ester (Methyl esterification by K_2CO_3/MeOH, 79%).

2,2,2-Trichloro-1-(5-methyl-1*H*-pyrrol-2-yl)ethan-1-one (SI-3)

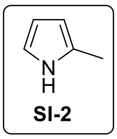

Pyrrole-2-carbaldehyde (**SI-1**, 5.71 g, 60.0 mmol, 1.00 eq.) and NaOH (12.5 g, 312 mmol, 5.20 eq.) were solved in ethylene glycol (80 mL) under argon atmosphere and hydrazine hydrate (18.1 mL, 372 mmol, 6.20 eq.) was added. The flask was equipped with a Dean-Stark apparatus and heated at 210 °C for 2.5 h. An azeotrope of glycol and 2-methyl pyrrole (**SI-2**) was condensed at the reflux condenser and collected in the Dean-Stark trap as biphasic mixture which was added to Et_2O (200 mL). The organic phase was washed with H_2O (100 mL, 2×50 mL), dried over Na_2SO_4 and evaporated. The raw methyl pyrrole (**SI-2**, 4.28 g, 88%) was used without further purification. **^{1}H-NMR** (500 MHz, $CDCl_3$) δ 7.88 (br. s, 1H), 6.67 (q, 1H, J = 2.2 Hz), 6.15 (q, 1H, J = 2.8 Hz), 5.93 (m, 1H), 2.30 (s, 3H) ppm.
Spectroscopic data corresponded to those reported in the literature.[3]

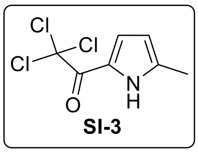

To a solution of trichloro acetylchloride (2.47 mL, 22.0 mmol, 1.10 eq.) in dry THF (10 mL) was slowly added 2-methyl pyrrole (**SI-2**, 1.72 mL, 20.0 mmol, 1.00 eq.) under argon atmosphere at 0 °C. The red solution was stirred at room temperature for 16 h and sat. aq. $NaHCO_3$ solution (100 mL) and 10% aq. K_2CO_3 solution (50 mL) were added. The mixture was extracted with EtOAc (4×50 mL) and the combined organic phases were washed with 10% aq. K_2CO_3 solution (50 mL) as well as brine (50 mL), dried over $NaSO_4$ and evaporated. The pyrrole **SI-3** (4.35 g, 96%) was obtained as

shiny black solid and was pure enough for the next step. **R_f** = 0.85 (hexanes/EtOAc 1:1); **IR** ν_{max}/cm^{-1}3315 (s), 3141 (w), 3102 (w), 2957 (w), 2920 (w), 1764 (w), 1636 (s), 1493 (m), 1399 (m), 1365 (s), 1262 (s), 1218 (s), 1054 (s), 842 (s), 808 (s), 784 (s), 743 (s), 726 (s), 681 (s); **^{1}H-NMR** (500 MHz, $CDCl_3$) δ 9.47 (br. s, 1H), 7.32 (dd, 1H, *J* = 2.6, 3.7 Hz), 6.11 (t, 1H, *J* = 3.7 Hz), 2.40 (s, 3H) ppm; **^{13}C-NMR** (125 MHz, $CDCl_3$) δ 172.7, 139.5, 122.8, 122.0, 111.3, 68.7, 13.6 ppm.

Spectroscopic data corresponded to those reported in the literature.[4]

Ethyl 5-methyl-1*H*-pyrrole-2-carboxylate (SI-4)

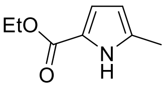

SI-4

Sodium (924 mg, 40.2 mmol, 1.30 eq.) was added to absolute EtOH (33 mL) and stirred until full dilution. Trichloro acetate **SI-3** (7.00 g, 30.9 mmol, 1.00 eq.) was added at room temperature and the solution was stirred for 35 min. It was concentrated at the rotary evaporator and 3M HCl (25 mL) was added. The solution was extracted with Et_2O (3×50 mL) and the organic phases were washed with sat. aq. $NaHCO_3$ solution (50 mL) and brine (50 mL). After drying over Na_2SO_4, the solvent was removed by rotary evaporation. The pale brown pyrrole **SI-4** (3.81 g, 81%) was used without purification. **mp** 97.2 °C, **R_f** = 0.87 (hexanes/EtOAc 2:1); **IR** ν_{max}/cm^{-1} 3288 (s), 3143 (w), 2987 (w), 2913 (w), 1667 (s), 1494 (m), 1321 (s), 1220 (s), 1152 (s), 1025 (s), 801 (s), 774 (s); **^{1}H-NMR** (500 MHz, $CDCl_3$) δ 8.97 (s, 1H), 6.81 (m, 1H), 5.95 (m, 1H), 4.30 (q, 2H, *J* = 7.1 Hz), 2.31 (s, 3H), 1.34 (t, 3H, *J* = 7.1 Hz) ppm; **^{13}C-NMR** (125 MHz, $CDCl_3$) δ 161.3, 133.7, 121.6, 116.1, 109.0, 60.2, 14.6, 13.3 ppm; **HRMS** ESI *m/z* $[M + H]^+$ calcd. for $C_8H_{12}NO_2$ 154.08626 found 154.08601.

Spectroscopic data corresponded to those reported in the literature.[4]

Ethyl 3,4-dichloro-5-methyl-1*H*-pyrrole-2-carboxylate (SI-5)

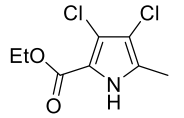

SI-5

Ester **SI-4** (2.06 g, 13.4 mmol, 1.00 eq.) was solved in CH_2Cl_2 (67 mL) at 0 °C and SO_2Cl_2 (2.17 mL, 26.9 mmol, 2.00 eq.) was slowly added. The solution was stirred for 3.5 h at 0 °C and sat. aq. $Na_2S_2O_3$ solution (80 mL) and sat. aq. $NaHCO_3$ solution (100 mL) were added. The mixture was extracted with EtOAc (2×100 mL), the combined organic phases were washed with brine (100 mL), dried over Na_2SO_4 and evaporated. The crude product was chromatographed (SiO_2, pentane/EtOAc 7:1 to 5:1). Pyrrole **SI-5** (741 mg, 25%) was obtained as colourless needles. **R_f** = 0.59

(hexanes/EtOAc 2:1); **IR** v_{max}/cm^{-1} 3315 (s), 3141 (w), 3102 (w), 2957 (w), 2920 (w), 1764 (m), 1636 (s), 1558 (m), 1493 (m), 1399 (m), 1365 (s), 1262 (s), 1218 (s), 1054 (s), 943 (w), 880 (w), 842 (s), 808 (s), 784 (s), 743 (s), 726 (s), 681 (s); **^{1}H-NMR** (500 MHz, $CDCl_3$) δ 9.02 (s, 1H), 4.35 (q, 2H, J = 7.1 Hz), 2.29 (s, 3H), 1.38 (t, 3H, J = 7.1 Hz) ppm; **^{13}C-NMR** (125 MHz, $CDCl_3$) δ 160.0, 129.1, 117.6, 116.2, 111.9, 61.1, 14.5, 11.7 ppm (quaternary C-atoms indicated by HMBC-correlations); **HRMS** ESI m/z [M + H]$^+$ calcd. for $C_8H_{10}Cl_2NO_2$ 222.00831, found 222.00833.

3,4-Dichloro-5-methyl-1*H*-pyrrole-2-carboxylic acid (31)

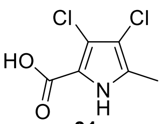

31

Ester **SI-5** (732 mg, 3.30 mmol, 1.00 eq.) was suspended in MeOH (33 mL) and H_2O (8.8 mL) at room temperature and 3M NaOH (4.40 mL, 13.2 mmol, 4.00 eq.) was added. The mixture was stirred for 22 h and further 3M NaOH (20 mL) was added. The mixture was extracted once with EtOAc (20 mL) and the aqueous phase was acidified to pH 1-2 with 1M HCl. The aqueous phase was extracted with EtOAc (3×50 mL). These organic phases were dried over Na_2SO_4 and evaporated. The carboxylic acid **31** (608 mg, 95%) was obtained as red solid. **mp** 102 °C (decomposition).**R_f** = 0.49 (hexanes/EtOAc 2:1); **IR** v_{max}/cm^{-1} 3113 (s), 2924 (s), 2590 (m), 2325 (s),1646 (s), 1544 (m), 1572 (m), 1498 (s), 1466 (m), 1360 (m), 1326 (m), 1283 (m), 1249 (m), 1102 (m), 1036 (m), 763 (m), 711 (m); **^{1}H-NMR** (500 MHz, CD_3OD) δ 2.23 (s, 3H) ppm; **^{13}C-NMR** (125 MHz, CD_3OD) δ 162.2, 130.6, 117.9, 117.2, 111.6, 10.9 ppm; **HRMS** ESI m/z [M − H]$^-$ calcd. for $C_6H_4Cl_2NO_2$ 191.96246, found 191.96179.

2.3 Failed routes amycolose derivative **4**

Our first try to build up amycolose derivative **4** was starting from lactic acid ester **SI-6** and perform a *de novo* synthesis of the sugar scaffold. Formation of ketones **SI-9a/b** was accomplished using Weinreb amide method. α,β-unsaturated esters **SI-10a/b** were synthesised in a HWE-olefination of ketones **SI-9a/b** with phosphonate **SI-15** which was itself synthesised by semihydrogenation under Lindlar-conditions of the corresponding alkyne. A base mediated deconjugation formed terminal dienes **SI-11a/b** which led to only low yields in the following Grubbs metathesis reaction. The Sharpless dihydroxylation to diols **SI-13a/b** was not observed. The following steps towards amycolose derived carbohydrate **4** should have been introduction of an amine and reduction of the lactone.

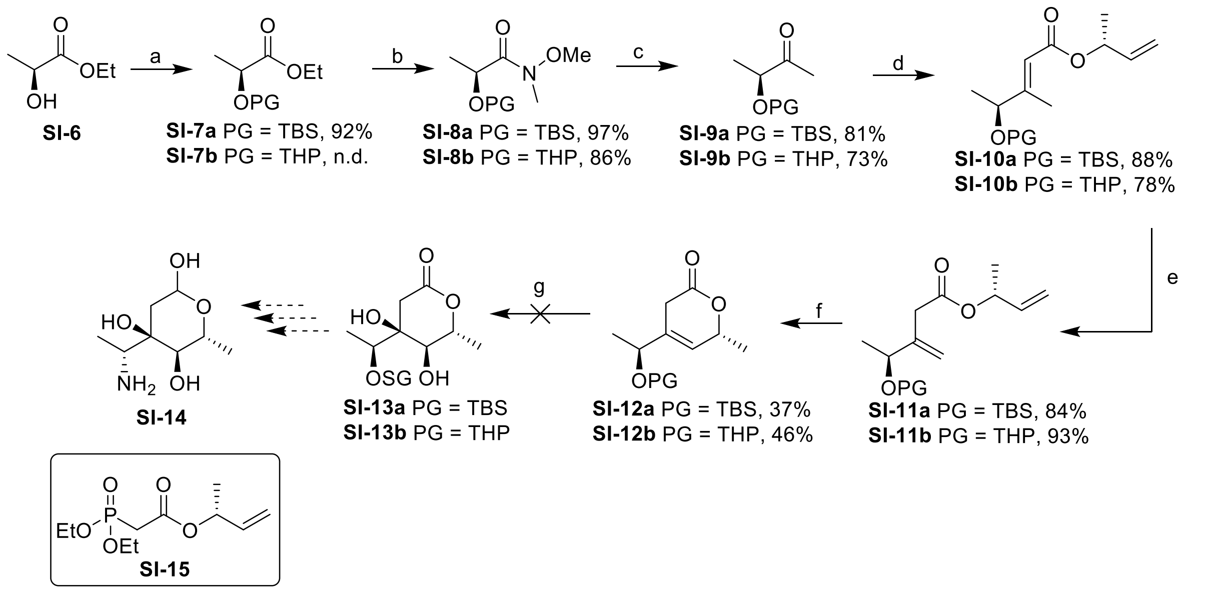

Scheme S3. Attempts to synthesise amycolose derivative **4** starting from lactic acid ester **SI-6**. Reagents and conditions: a) **SI-7a**: TBSCl, imidazole, DMAP, CH_2Cl_2, rt, 19 h; **SI-7b**: DHP, PPTS, CH_2Cl_2, rt; b) MeONHMe·HCl, *i*PrMgCl, LiCl/BuLi, THF, 0 °C, 19 h; c) MeMgBr/MeLi, THF; d) BuLi, LiHMDS, **SI-15**, THF; e) LDA/LiHMDS, HMPT, THF, −78 °C, then AcOH, Et_2O; f) Grubbs catalyst 2nd generation, Ti(O*i*Pr)$_4$, CH_2Cl_2, reflux, 21 h; g) AD-mix.

Another idea synthesising amycolose derivative **4** was starting from sugar based mannitol (**SI-16**) using a fully diastereoselective approach. After acetonide protection of both terminal diols a monobenzoylation was carried out (→**SI-18**). The free hydroxyl group was oxidised and ketone **SI-19** was treated with vinylMgBr. After protection of alcohol **SI-20**, the following acetonide deprotection was not possible. The next steps should have been the deoxygenation of

the primary position, protection of the vicinal hydroxy groups as well as amine and aldehyde formation and ultimate deprotection to carbohydrate **4**.

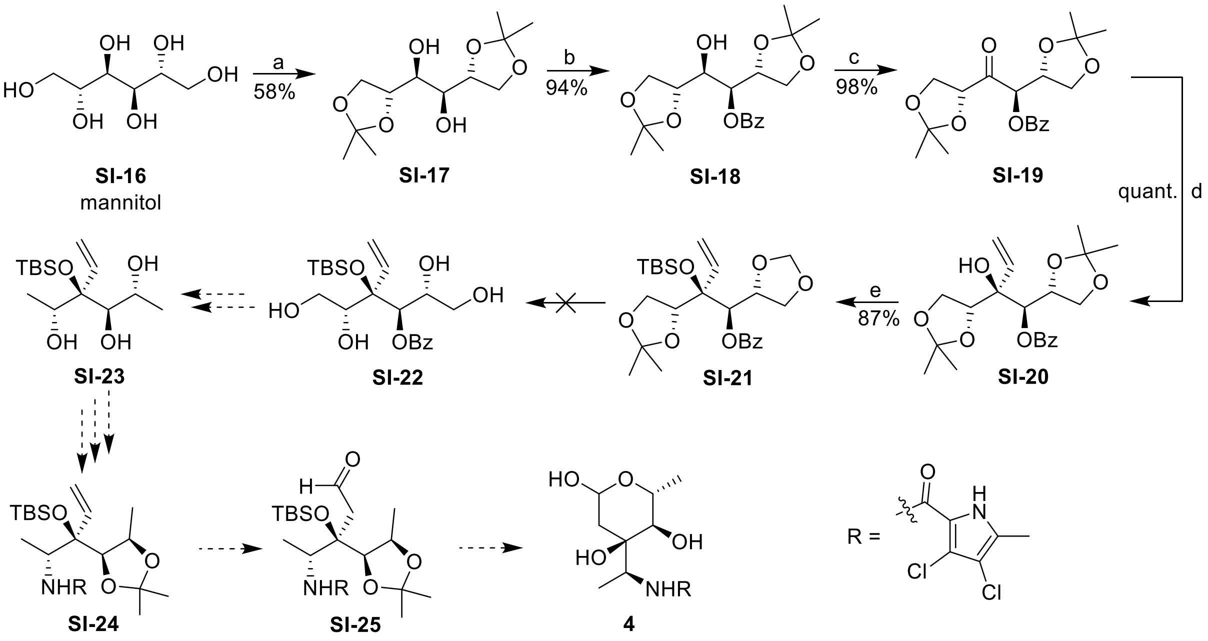

Scheme S4. Attempts to synthesise amycolose starting from mannitol (**SI-16**).
Reagents and conditions: a) $ZnCl_2$, acetone, rt, 15 h; b) Cu(bipy), DIPEA, BzCl, $CH_2Cl_2/CHCl_3$, 0 °C→rt, 5 h; c) DMP, $NaHCO_3$, CH_2Cl_2, rt, 3 h; d) vinylMgBr, THF, −78 °C, 40 min; e) 1. KH, THF, 0 °C, 10 min, 2. TBSCl, rt, 2 h.

2.4 Synthesis of sugar **43** – derivatization of amycolose

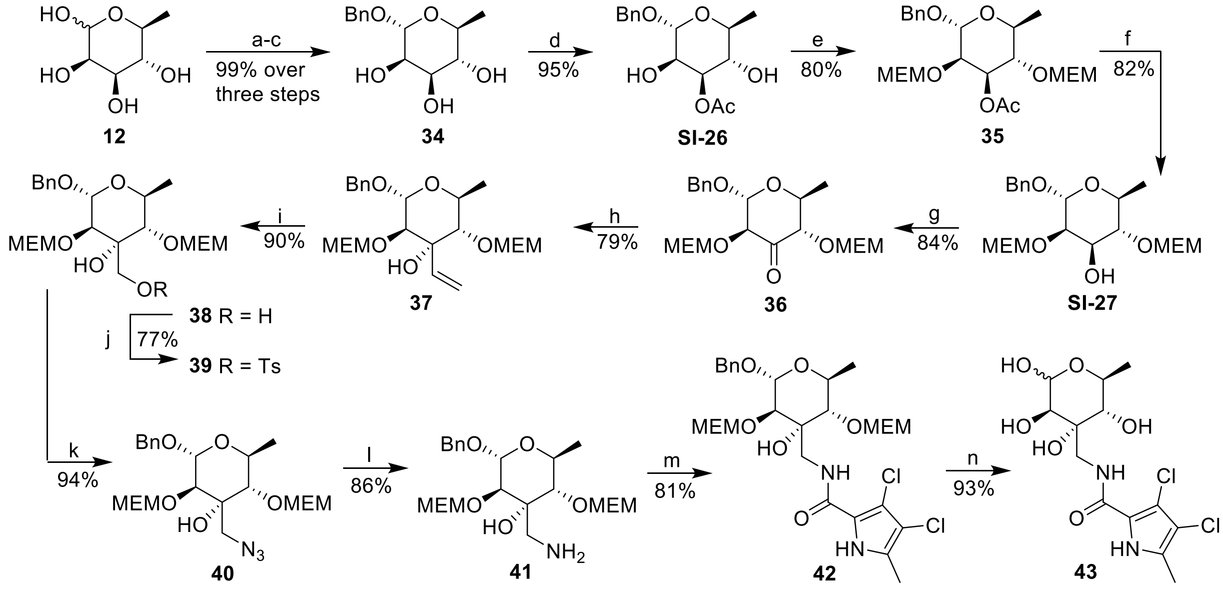

Scheme S5. Synthesis of amycolose derived carbohydrate **43**.
Reagents and conditions: a) Ac_2O, pyridine, rt, 22 h; b) BnOH, BF_3·OEt, 4 Å MS, CH_2Cl_2, 0 °C→rt, on; c) NaOMe, MeOH, rt, 4 d; d) $MoO_2(acac)_2$, collidine, AcCl, 1,4-dioxane, RT, 3 h; e) MEMCl, DIPEA, CH_2Cl_2, 0 °C→40 °C, 1 d; f) DIBAL, toluene, 0 °C, 3 h; g) DMP, CH_2Cl_2, 0 °C→rt, 5 h; h) vinylMgBr, THF, −78 °C, 5 h; i) 1. O_3, CH_2Cl_2/MeOH, −78 °C, 10 min; 2. $NaBH_4$, rt, 24 h; j) *p*TsCl, DMAP, NEt_3, CH_2Cl_2, rt, 21 h; k) NaN_3, DMF, 65 °C, 17 h; l) 1. PPh_3, THF, rt, 2 d; 2. H_2O, rt, 3 d; m) **31**, EDC·HCl, HOBt, DMAP, CH_2Cl_2, 0 °C→rt, on; n) BCl_3, CH_2Cl_2, −78 °C, 3.5 h.

(3*R*,4*R*,5*R*,6*S*)-2-(Benzyloxy)-6-methyltetrahydro-2*H*-pyran-3,4,5-triol (34)

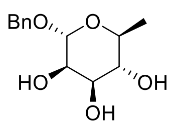
34

L-Rhamnose (**12**, 10.0 g, 54.9 mmol, 1.00 eq.) was dissolved in Ac_2O (57.0 mL) and pyridine (57.0 mL) at room temperature. The solution was stirred for 22 h and the volatiles were removed under reduced pressure. The crude product was diluted with CH_2Cl_2 and a sat. aq. Cu_2SO_4 solution. The aqueous phase was extracted thrice with CH_2Cl_2. The combined organic phases were dried over Na_2SO_4 and the solvents were removed under reduced pressure. After purification by column chromatography (SiO_2, pentane/EtOAc 5:1→3:1→2:1) the product (18.2 g, 54.9 mmol) was isolated in quantitative yield.

The peracetylated rhamnose (18.0 g, 54.3 mmol, 1.00 eq.) in dry CH_2Cl_2 (147 mL) was treated with BnOH (28.2 mL, 271 mmol, 5.00 eq.) and 4 Å molecular sieve (12 g) at room temperature. After stirring for 30 min BF_3·OEt_2 (55.0 mL, 434 mmol, 8.00 eq.) was added at 0 °C over a period of 45 min. The mixture was allowed to warm to room temperature overnight. After TLC showed complete conversion of the starting material, the reaction was quenched by slow

addition of H_2O. The mixture was diluted with CH_2Cl_2. The aqueous phase was extracted four times with CH_2Cl_2, and the combined organic phases were dried over Na_2SO_4. The volatiles were removed under reduced pressure and the crude product was used without further purification.

Fully protected rhamnose (20.7 g, 54.3 mmol, 1.00 eq.) was dissolved in dry MeOH (180 mL) and treated with NaOMe (25wt%, 3.72 mL, 16.3 mmol, 0.30 eq.) at room temperature. After 18 h of stirring, another portion of NaOMe (25wt%, 3.72 mL, 16.3 mmol, 0.30 eq.) was added. Stirring was continued for 3 d. The mixture was neutralised by addition of DOWEX. The solid was filtered off over celite® and the solvents were removed under reduced pressure. Purification by column chromatography (SiO_2, pentane/EtOAc 1:1→0:1) gave the product **34** (13.7 g, 99%, α:β >10:1) as a light yellow resin, minor impurities occurred due to β-anomer. $\mathbf{R_f}$ = 0.40 (CH_2Cl_2/MeOH 9:1); $[\alpha]_D^{20}$ −8.52° (c 1.0 in $CHCl_3$); **IR** ν_{max}/cm^{-1} 3392 (m), 2991 (w), 2906 (w), 1455 (w), 1276 (m), 1261 (m), 1131 (m), 1049 (m), 980 (m), 911 (w), 810 (w), 764 (s), 750 (s), 698 (m); **^{1}H-NMR** (500 MHz, CD_3OD) δ 7.37-7.22 (m, 5H), 4.75 (d, 1H, J = 1.6 Hz), 4.69 (d, 1H, J = 11.9 Hz), 4.51 (d, 1H, J = 11.9 Hz), 3.82 (dd, 1H, J = 1.6, 3.4 Hz), 3.68 (dd, 1H, J = 3.4, 9.5 Hz), 3.62 (dq, 1H, J = 6.2, 9.5 Hz), 3.39 (t, 1H, J = 9.5 Hz), 1.27 (d, 3H, J = 6.2 Hz) ppm; **^{13}C-NMR** (125 MHz, CD_3OD) δ 139.1, 129.4, 129.1, 128.8, 100.8, 74.0, 72.4, 72.3, 70.01, 70.00, 18.0 ppm.

Spectroscopic data corresponded to those reported in the literature.[5]

(3*R*,4*R*,5*S*,6*S*)-2-(Benzyloxy)-3,5-dihydroxy-6-methyltetrahydro-2*H*-pyran-4-yl acetate (SI-26)

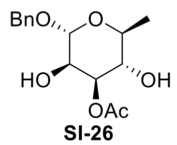

SI-26

Benzylated rhamnose **34** (900 mg, 3.54 mmol, 1.00 eq.) in dry 1,4-dioxane (29 mL) was treated with $MoO_2(acac)_2$ (57.7 mg, 177 µmol, 0.05 eq.), collidine (937 µL, 7.08 mmol, 2.00 eq.) and AcCl (379 µL, 5.31 mmol, 1.50 eq.) at room temperature. The mixture was stirred for 3 h and diluted with H_2O and CH_2Cl_2. The aqueous phase was extracted twice with CH_2Cl_2 and the combined organic phases were dried over Na_2SO_4. The volatiles were removed under reduced pressure. Purification by column chromatography (SiO_2, pentane/EtOAc 2:1→1:1) afforded the product **SI-26** (994 mg, 95%) as a colourless resin. The product was isolated as major isomer of a mixture of different regiosiomers (100:10:7). $\mathbf{R_f}$ = 0.64 (CH_2Cl_2/MeOH 9:1); $[\alpha]_D^{20}$ −74.5° (c 1.0 in $CHCl_3$); **IR** ν_{max}/cm^{-1} 3439 (m), 2980 (w), 2933 (w), 1717 (m), 1497 (w), 1455 (w), 1372

(m), 1275 (m), 1260 (s), 1128 (m), 1049 (s), 983 (m), 886 (w), 842 (w), 805 (w), 764 (s), 750 (s), 699 (m); major regioisomer **^{1}H-NMR** (500 MHz, $CDCl_3$) δ 7.38-7.27 (m, 5H), 5.08 (dd, 1H, J = 3.3, 9.8 Hz), 4.83 (d, 1H, J = 1.7 Hz), 4.72 (d, 1H, J = 12.0 Hz), 4.52 (d, 1H, J = 12.0 Hz), 4.05 (dd, 1H, J = 1.7, 3.3 Hz), 3.78 (dq, 1H, J = 6.2, 9.5 Hz), 3.64 (t, 1H, J = 9.8 Hz), 2.45 (br. s, 2H), 2.14 (s, 3H), 1.35 (d, 3H, J = 6.2 Hz) ppm; major regioisomer **^{13}C-NMR** (125 MHz, $CDCl_3$) δ 171.9, 137.0, 128.6, 128.2, 128.1, 98.5, 75.1, 71.7, 70.0, 69.3, 68.9, 21.3, 17.7 ppm; HRMS ESI *m/z* [M + Na]$^+$ calcd. for $C_{15}H_{20}O_6Na$ 315.11521 found 315.11417.

(3*R*,4*R*,5*S*,6*S*)-2-(Benzyloxy)-3,5-bis((2-methoxyethoxy)methoxy)-6-methyltetrahydro-2*H*-pyran-4-yl acetate (35)

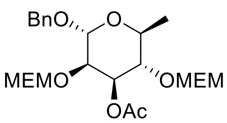

35

Carbohydrate **SI-26** (11.4 g, 38.3 mmol, 1.00 eq.) in dry CH_2Cl_2 (58 mL) was treated with DIPEA (20.0 mL, 115 mmol, 3.00 eq.) and MEMCl (13.1 mL, 115 mmol, 3.00 eq.) at 0 °C. After 30 min at 0 °C, the solution was allowed to warm to room temperature. DIPEA (6.67 mL, 38.3 mmol, 1.00 eq.) and MEMCl (4.37 mL, 38.3 mmol, 1.00 eq.) were added after 7 h at 0 °C. The solution was stirred at room temperature overnight and for 6 h at 40 °C. As soon as TLC showed complete conversion, the mixture was allowed to come to room temperature and EtOAc as well as sat. aq. K_2CO_3 solution were added. The organic phase was separated and washed with 1 M HCl. The combined aqueous phases were extracted thrice with EtOAc. All organic phases were washed with brine and dried over Na_2SO_4. Removal of the solvent under reduced pressure and purification of the crude product by column chromatography (SiO_2, pentane/EtOAc 4:1→3:1→2:1→1:1) gave the product **35** (14.4 g, 80%) as a colourless resin and as a mixture of regioisomers. **R$_f$** = 0.38 (hexanes/EtOAc 1:1); $[\alpha]_D^{20}$ −79.9° (c 1.0 in $CHCl_3$); **IR** ν_{max}/cm^{-1} 2935 (m), 2888 (m), 2816 (w), 1743 (m), 1456 (m), 1367 (m), 1237 (s), 1111 (m), 1035 (s), 750 (m) 700 (m); major regioisomer **^{1}H-NMR** (500 MHz, $CDCl_3$) δ 7.36-7.26 (m, 5H), 5.23 (dd, 1H, J = 3.3, 9.5 Hz), 4.86 (d, 1H, J = 6.7 Hz), 4.85 (d, 1H, J = 2.0 Hz), 4.73 (d, 1H, J = 6.7 Hz), 4.73 (d, 1H, J = 6.7 Hz), 4.72 (d, 1H, J = 6.7 Hz), 4.70 (d, 1H, J = 12.0 Hz), 4.51 (d, 1H, J = 12.0 Hz), 4.05 (dd, 1H, J = 2.0, 3.2 Hz), 3.80-3.63 (m, 6H), 3.53 (m, 2H), 3.45 (m, 2H), 3.38 (s, 3H), 3.35 (s, 3H), 2.08 (s, 3H), 1.31 (d, 3H, J = 6.2 Hz) ppm; major regioisomer **^{13}C-NMR** (125 MHz, $CDCl_3$) δ 170.2, 137.3, 128.5, 128.0, 127.9, 97.7, 96.9, 95.8, 77.7, 75.0, 73.2, 71.8, 71.6, 69.2, 68.0, 67.8, 67.2, 59.22, 59.17, 21.3, 18.1 ppm; **HRMS** ESI *m/z* [M + Na]$^+$ calcd. for $C_{23}H_{36}O_{10}$ 495.21925 found 495.22007.

(3*R*,4*R*,5*R*,6*S*)-2-(Benzyloxy)-3,5-bis((2-methoxyethoxy)methoxy)-6-methyltetrahydro-2*H*-pyran-4-ol (SI-27)

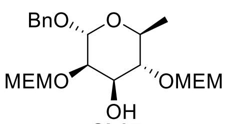

SI-27

The fully protected sugar **35** (859 mg, 1.82 mmol, 1.00 eq.) in dry toluene (25.0 mL) was treated with DIBAL (3.49 mL, 3.49 mmol, 1.90 eq.) at 0 °C. After stirring for 3 h at this temperature, sat. aq. Na,K-tartrate solution, Na,K-tartrate and acetone were added. The mixture was stirred for 40 min at room temperature. The organic phase was separated, and the aqueous phase was extracted thrice with CH_2Cl_2. The combined organic phases were washed with brine and dried over Na_2SO_4. Removal of the solvents and purification by column chromatography (SiO_2, pentane/EtOAc 2:1→1:1→0:1) afforded product **SI-27** (640 mg, 82%) as a colourless oil and as a mixture of regioisomers. **R$_f$** = 0.71 (CH_2Cl_2/MeOH 95:5); $[\boldsymbol{\alpha}]_D^{20}$ −61.9° (c 1.0 in $CHCl_3$); **IR** ν_{max}/cm^{-1} 3463 (m), 2980 (m), 2924 (m), 2889 (m), 2826 (w), 1455 (m), 1366 (w), 1276 (m), 1261 (m), 1112 (m), 1024 (s), 984 (m), 845 (m), 800 (w), 764 (s), 750 (s), 700 (m); major regioisomer **^{1}H-NMR** (500 MHz, $CDCl_3$) δ 7.37-7.26 (m, 5H), 4.93 (d, 1H, J = 6.8 Hz), 4.91 (d, 1H, J = 1.5 Hz), 4.91 (d, 1H, J = 6.8 Hz), 4.80 (d, 1H, J = 7.1 Hz), 4.78 (d, 1H, J = 7.1 Hz), 4.70 (d, 1H, J = 11.9 Hz), 4.48 (d, 1H, J = 11.9 Hz), 3.96 (m, 1H), 3.89 (m, 2H), 3.78 (m, 2H), 3.70 (m, 3H), 3.56 (m, 2H), 3.50 (m, 2H), 3.41 (t, 1H, J = 8.5 Hz), 3.38 (s, 3H), 3.36 (s, 3H), 1.29 (d, 3H, J = 6.3 Hz) ppm; major regioisomer **^{13}C-NMR** (125 MHz, $CDCl_3$) δ 137.6, 128.6, 127.90, 127.89, 98.1, 97.1, 96.6, 83.0, 77.9, 71.8, 71.7, 70.2, 69.2, 67.8, 67.4, 67.3, 59.2, 59.1, 17.9 ppm; **HRMS** ESI *m/z* [M + Na]$^+$ calcd. for $C_{21}H_{34}O_9Na$ 453.20890 found 453.20950.

(3*S*,5*S*,6*S*)-2-(Benzyloxy)-3,5-bis((2-methoxyethoxy)methoxy)-6-methyltetrahydro-4*H*-pyran-4-one (36)

Partially protected rhamnose **SI-27** (5.53 g, 12.8 mmol, 1.00 eq.) was dissolved in CH_2Cl_2 *p.a.* (51.0 mL) and DMP (6.53 g, 15.4 mmol, 1.20 eq.) was added at 0 °C. The suspension was allowed to warm to room temperature after 30 min. The reaction was quenched by addition of sat. aq. $Na_2S_2O_3$ solution and sat. aq. $NaHCO_3$ solution after 5 h. The aqueous phase was extracted thrice with EtOAc, combined organic phases were washed with sat. aq. $Na_2S_2O_3$ solution, sat. aq. $NaHCO_3$ solution, brine and dried over Na_2SO_4. Solvents were removed under reduced pressure. The crude product was purified by column chromatography (SiO_2, pentane/EtOAc 2:1) to give a mixture of product and residues of DMP. It was diluted in EtOAc and washed

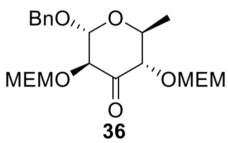

36

twice with sat. aq. $Na_2S_2O_3$ solution and sat. aq. $NaHCO_3$ solution alternately. The product **36** (4.58 g, 84%) was obtained as a colourless oil and as a mixture of regioisomers. **R$_f$** = 0.67 (hexanes/EtOAc 1:1); $[\alpha]_D^{20}$ −143.9° (c 1.0 in $CHCl_3$); **IR** ν_{max}/cm^{-1} 2938 (m), 2896 (m), 2826 (w), 1745 (m), 1137 (s), 1123 (s), 1052 (s), 997 (m), 751 (m); major regioisomer **^{1}H-NMR** (500 MHz, $CDCl_3$) δ 7.35-7.23 (m, 5H), 5.06 (d, 1H, *J* = 1.6 Hz), 4.82 (d, 1H, *J* = 7.1 Hz), 4.76 (d, 1H, *J* = 7.1 Hz), 4.74 (s, 2H), 4.68 (d, 1H, *J* = 12.2 Hz), 4.51 (d, 1H, *J* = 12.2 Hz), 4.40 (d, 1H, *J* = 9.4 Hz), 4.02 (d, 1H, *J* = 1.6 Hz), 3.98 (dq, 1H, *J* = 6.1, 9.4 Hz), 3.76 (m, 2H), 3.71-3.61 (m, 2H), 3.52 (m, 2H), 3.46 (m, 2H), 3.36 (s, 3H), 3.32 (s, 3H), 1.40 (d, 3H, *J* = 6.2 Hz) ppm; major regioisomer **^{13}C-NMR** (125 MHz, $CDCl_3$) δ 202.2, 136.6, 128.5, 128.0, 127.9, 99.7, 95.5, 95.2, 81.2, 80.0, 71.7, 71.6, 70.5, 69.1, 67.8, 67.6, 59.1, 59.0, 18.7 ppm; **HRMS** ESI *m/z* [M + Na]$^+$ calcd. for $C_{21}H_{32}O_9Na$ 451.19321 found 451.19385.

(3*R*,5*S*,6*S*)-2-(Benzyloxy)-3,5-bis((2-methoxyethoxy)methoxy)-6-methyl-4-vinyltetra-hydro-2*H*-pyran-4-ol (37)

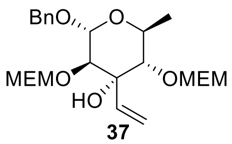

37

Ketone **36** (4.45 g, 10.4 mmol, 1.00 eq.) in dry THF (100 mL) was treated slowly with vinylMgBr solution (1 M in THF, 30.1 mL, 30.1 mmol, 3.00 eq., 1.00 mL per minute) at −78 °C. After 5 h at this temperature, the reaction was quenched by addition of sat. aq. NH_4Cl solution. The organic phase was separated, and the aqueous phase was extracted thrice with EtOAc, combined organic phases were washed with brine and dried over Na_2SO_4. Removal of the solvent under vacuum and purification by column chromatography (SiO_2, pentane/EtOAc 3:1→2:1→1:1) gave the product **37** (3.73 g, 79%, dr >30:1 determined by NMR) as a colourless oil and as a mixture of regioisomers. **R$_f$** = 0.52 (hexanes/EtOAc 1:1); $[\alpha]_D^{20}$ −90.5° (c 1.0 in $CHCl_3$); **IR** ν_{max}/cm^{-1} 3498 (m), 2942 (m), 2891 (m), 1455 (w), 1362 (w), 1200 (w), 1173 (m), 1135 (m), 1112 (m), 1024 (s), 958 (m), 847 (w), 739 (w), 700 (m); major regioisomer **^{1}H-NMR** (500 MHz, $CDCl_3$) δ 7.30-7.07 (m, 5H), 6.07 (ddd, 1H, *J* = 1.2, 10.7, 17.2 Hz), 5.61 (dd, 1H *J* = 2.0, 17.2 Hz), 5.22 (dd, 1H, *J* = 2.0, 10.7 Hz), 4.91 (d, 1H, *J* = 0.9 Hz), 4.77 (d, 1H, *J* = 11.7 Hz), 4.74 (s, 2H), 4.70 (s, 2H), 4.55 (d, 1H, *J* = 11.7 Hz), 4.09 (d, 1H, *J* = 1.2 Hz), 4.00 (dq, 1H, *J* = 6.3, 9.7 Hz), 3.72 (m, 2H), 3.66 (m, 1H), 3.60 (m, 2H), 3.53 (d, 1H, *J* = 9.7 Hz), 3.51 (m, 2H), 3.48-3.39 (m, 2H), 3.38 (s, 3H), 3.35 (s, 3H), 1.33 (d, 3H, *J* = 6.3 Hz) ppm; major regioisomer **^{13}C-NMR** (125 MHz, $CDCl_3$) δ 139.5, 136.7, 128.7, 128.32, 128.27, 116.3, 98.0, 97.1, 96.1, 79.71, 79.69, 74.4, 71.8, 71.6, 69.8, 67.9, 67.5, 64.4, 59.21, 59.15, 18.1 ppm; **HRMS** ESI *m/z* [M + Na]$^+$ calcd. for $C_{23}H_{36}O_9Na$ 479.22483 found 479.22515.

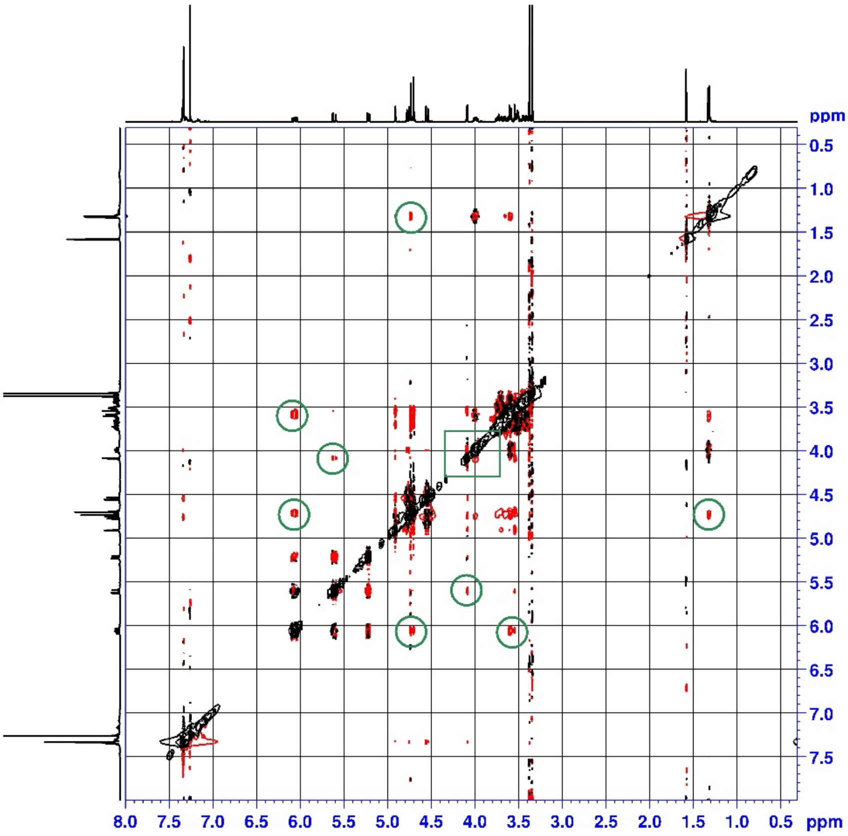

Fig. S4. Relevant NOE-signals for elucidation of stereoconfiguration of glycoside **37.**

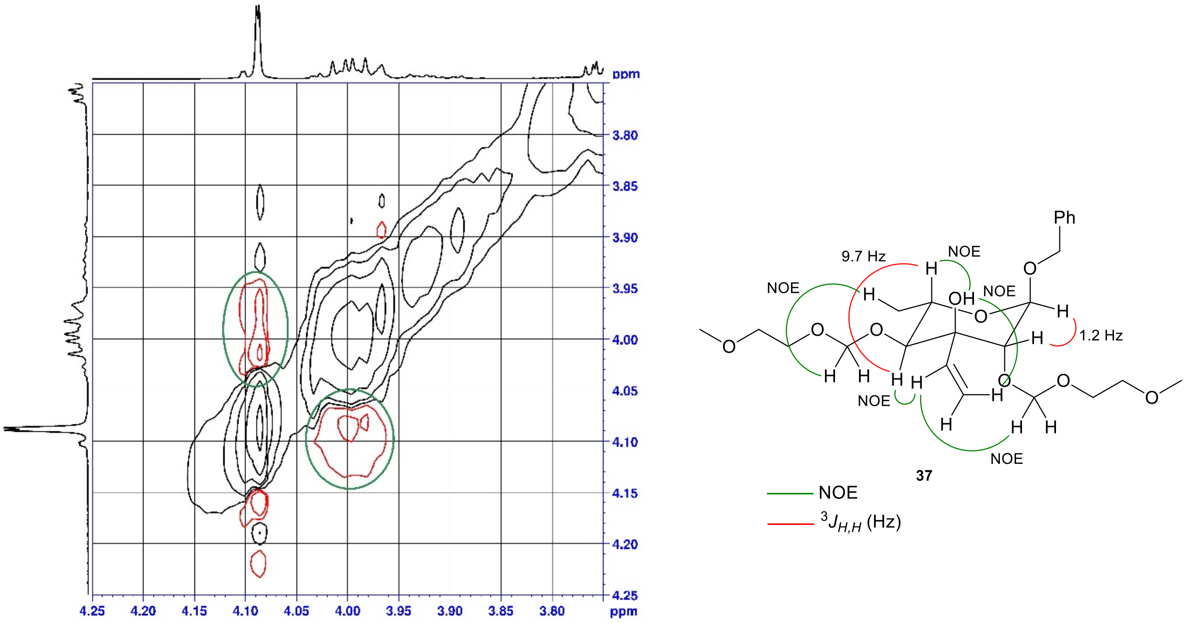

Fig. S5. Relevant NOE-signals for elucidation of stereoconfiguration of glycoside **37.**

(3*R*,5*S*,6*S*)-2-(Benzyloxy)-4-(hydroxymethyl)-3,5-bis((2-methoxyethoxy)methoxy)-6-methyltetrahydro-2*H*-pyran-4-ol (38)

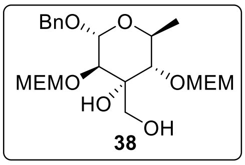

Carbohydrate **37** (3.61 g, 7.90 mmol, 1.00 eq.) was dissolved in MeOH *p.a.* (120 mL) and CH_2Cl_2 *p.a.* (120 mL) and cooled to −78 °C. O_3/O_2 was bubbled through the solution until it turned blue. This was followed by passing oxygen through the solution up to the blue colour disappeared. $NaBH_4$ (724 mg, 19.1 mmol, 2.40 eq.) was added and the solution was slowly allowed to come to room temperature. After stirring for 24 h, the residues were filtered off over celite® and the volatiles were removed under reduced pressure. Purification of the crude product by column chromatography (SiO_2, CH_2Cl_2/MeOH 19:1) gave the product **38** (3.28 g, 90%) as a colourless oil and as a mixture of regioisomers. $\mathbf{R_f}$ = 0.25 (hexanes/EtOAc 1:1); $[\alpha]_D^{20}$ −52.4° (c 1.0 in $CHCl_3$); **IR** ν_{max}/cm^{-1} 3486 (m), 2977 (m), 2935 (m), 2886 (m), 2819 (w), 1456 (m), 1363 (w), 1276 (m), 1261 (m), 1112 (m), 1024 (s), 847 (w), 764 (s), 750 (s), 701 (w); major regioisomer **^{1}H-NMR** (500 MHz, $CDCl_3$) δ 7.36-7.26 (m, 5H), 4.99 (s, 1H), 4.84 (d, 1H, *J* = 7.0 Hz), 4.80 (d, 1H, *J* = 7.0 Hz), 4.76 (d, 1H, *J* = 7.0 Hz), 4.75 (d, 1H, *J* = 11.5 Hz), 4.73 (d, 1H, *J* = 7.0 Hz), 4.54 (d, 1H, *J* = 11.5 Hz), 4.11 (d, 1H, *J* = 1.2 Hz), 3.98 (dq, 1H, *J* = 6.3, 9.7 Hz), 3.86 (d, 1H, *J* = 1.0 Hz), 3.79 (ddd, 1H, *J* = 3.8, 5.3, 9.1 Hz), 3.76-3.63 (m, 5H), 3.56-3.42 (m, 5H), 3.38 (s, 3H), 3.36 (s, 3H), 2.47 (dd, 1H, *J* = 3.8, 9.8 Hz), 1.31 (d, 3H, *J* = 6.3 Hz) ppm; major regioisomer **^{13}C-NMR** (125 MHz, $CDCl_3$) δ 136.5, 128.7, 128.32, 128.26, 98.1, 97.8, 96.0, 78.8, 75.11, 75.06, 71.7, 71.6, 69.9, 68.4, 67.6, 64.1, 63.9, 59.2, 59.1, 18.0 ppm; **HRMS** ESI *m/z* $[M + Na]^+$ calcd. for $C_{22}H_{36}O_{10}Na$ 483.21957 found 483.22007.

((3*R*,5*S*,6*S*)-2-(Benzyloxy)-4-hydroxy-3,5-bis((2-methoxyethoxy)methoxy)-6-methyl-tetrahydro-2*H*-pyran-4-yl)methyl-4-methylbenzenesulfonate (39)

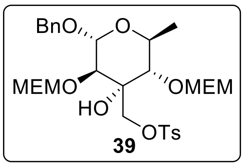

Carbohydrate **38** (36.0 mg, 78.2 µmol, 1.00 eq.) was dissolved in dry CH_2Cl_2 (550 µL) and treated with *p*TsCl (22.4 mg, 117 µmol, 1.50 eq.), dry NEt_3 (16.3 µL, 117 µmol, 1.50 eq.) and DMAP (478 µg, 3.91 µmol, 0.05 eq.) at room temperature. The solution was stirred for 21 h and H_2O was added. The organic phase was separated, and the aqueous phase was extracted thrice with EtOAc. The combined organic phases were washed with 1M HCl, H_2O as well as brine and dried over Na_2SO_4. The solvents were removed under vacuum and the crude product was purified by column chromatography (SiO_2, pentane/EtOAc 2:1). The tosylated sugar **39**

(37.1 mg, 77%) was isolated as a colourless oil. It was pure enough for next step. **R$_f$** = 0.41 (hexanes/EtOAc 1:1); $[\alpha]_D^{20}$ −58.8° (c 1.0 in $CHCl_3$); **IR** ν_{max}/cm^{-1} 3482 (m), 2931 (m), 2890 (m), 1600 (w), 1456 (m), 1362 (m), 1177 (s), 1114 (m), 1033 (s), 972 (m), 841 (m), 752 (w), 700 (m), 663 (w); major regioisomer **^{1}H-NMR** (500 MHz, $CDCl_3$) δ 7.79 (d, 2H, *J* = 8.3 Hz), 7.36-7.26 (m, 7H), 5.06 (s, 1H), 4.80 (d, 1H, *J* = 7.3 Hz), 4.73 (d, 1H, *J* = 7.2 Hz), 4.71 (d, 1H, *J* = 11.5 Hz), 4.69 (d, 1H, *J* = 7.2 Hz), 4.69 (d, 1H, *J* = 7.3 Hz), 4.51 (d, 1H, *J* = 11.5 Hz), 4.27 (dd, 1H, *J* = 2.2, 9.8 Hz), 4.10 (d, 1H, *J* = 9.8 Hz), 3.93 (dq, 1H, *J* = 6.1, 9.6 Hz), 3.78 (d, 1H, *J* = 1.2 Hz), 3.76 (ddd, 1H, *J* = 2.8, 6.3, 9.3 Hz), 3.69 (m, 1H), 3.61-3.47 (m, 5H), 3.44-3.39 (m, 2H), 3.40 (s, 3H), 3.36 (s, 3H), 2.41 (s, 3H), 1.31 (d, 3H, *J* = 6.1 Hz) ppm; major regioisomer **^{13}C-NMR** (125 MHz, $CDCl_3$) δ 144.8, 136.4, 133.1, 129.9, 128.7, 128.6, 128.4, 128.33, 128.27, 128.1, 98.0, 97.6, 96.8, 78.9, 75.2, 74.2, 71.6, 70.1, 68.6, 67.6, 63.8, 59.3, 59.1, 21.8, 17.7 ppm; **HRMS** ESI *m/z* $[M + Na]^+$ calcd. for $C_{29}H_{42}O_{12}SNa$ 637.22820 found 637.22892.

(3*R*,5*S*,6*S*)-4-(Azidomethyl)-2-(benzyloxy)-3,5-bis((2-methoxyethoxy)methoxy)-6-methyl-tetra-hydro-2*H*-pyran-4-ol (40)

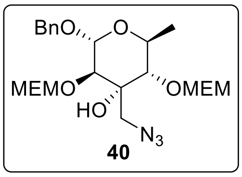

Tosylated sugar **39** (2.40 g, 3.90 mmol, 1.00 eq.) in dry DMF (15 mL) was treated with NaN_3 (760 mg, 11.7 mmol, 3.00 eq.) at room temperature. The mixture was stirred at 65 °C for 17 h and NaN_3 (760 mg, 11.7 mmol, 3.00 eq.) was added again. After stirring for a further 35 h at 70 °C, it was allowed to come to room temperature and H_2O was added. The aqueous phase was extracted thrice with EtOAc and the combined organic phases were washed with H_2O, brine and dried over Na_2SO_4. After removal of the solvents under vacuum, the crude product was purified by column chromatography (SiO_2, pentane/EtOAc 2:1→1.5:1) to give azide **40** (1.78 g, 94%) as a colourless oil, minor impurities occur due to regioisomers. **R$_f$** = 0.53 (hexanes/EtOAc 1:1); $[\alpha]_D^{20}$ −41.1° (c 1.0 in $CHCl_3$); **IR** ν_{max}/cm^{-1} 3484 (m), 2928 (m), 2880 (m), 2826 (w), 2099 (s), 1455 (m), 1364 (w), 1276 (m), 1261 (m), 1200 (w), 1134 (m), 1111 (s), 1022 (s), 977 (m), 919 (m), 847 (m), 764 (m), 750 (s), 700 (m); major regioisomer **^{1}H-NMR** (500 MHz, $CDCl_3$) δ 7.38-7.26 (m, 5H), 5.05 (d, 1H, *J* = 0.9 Hz), 4.85 (d, 1H, *J* = 7.1 Hz), 4.80 (d, 1H, *J* = 7.1 Hz), 4.77 (d, 1H, *J* = 7.1 Hz), 4.75 (d, 1H, *J* = 11.5 Hz), 4.73 (d, 1H, *J* = 7.1 Hz), 4.55 (d, 1H, *J* = 11.5 Hz), 4.18 (d, 1H, *J* = 2.2 Hz), 3.95 (dq, 1H, *J* = 6.5, 9.9 Hz), 3.86 (d, 1H, *J* = 1.4 Hz), 3.85 (ddd, 1H, *J* = 4.1, 4.9, 10.8 Hz), 3.75 (ddd, 1H, *J* = 2.9, 6.2, 10.8 Hz), 3.69 (ddd, 1H, *J* = 4.1, 4.9, 10.8 Hz), 3.65 (d, 1H, *J* = 12.6 Hz), 3.59 (ddd, 1H, *J* = 2.9, 6.4, 10.8 Hz), 3.55 (m, 2H), 3.49 (ddd, 1H, *J* = 2.9, 6.2, 10.8 Hz), 3.42 (ddd, 1H, *J* = 2.9, 6.4, 10.8 Hz), 3.39 (s, 3H), 3.36

(s, 3H), 3.37 (m, 1H), 3.23 (dd, 1H, J = 2.4, 12.5 Hz), 1.29 (d, 3H, J = 6.5 Hz) ppm; major regioisomer **^{13}C-NMR** (125 MHz, $CDCl_3$) δ 136.4, 128.8, 128.4 (2 signals), 98.2, 97.9, 96.4, 80.0, 75.9, 75.3, 71.8, 71.6, 70.1, 68.6, 67.6, 64.1, 59.22, 59.16, 54.5, 17.9 ppm; **HRMS** ESI *m/z* $[M + Na]^+$ calcd. for $C_{22}H_{35}N_3O_9Na$ 508.22636 found 508.22655.

(2*R*,3*R*,4*S*,5*S*,6*S*)-4-(Aminomethyl)-2-(benzyloxy)-3,5-bis((2-methoxyethoxy)methoxy)-6-methyltetrahydro-2*H*-pyran-4-ol (41)

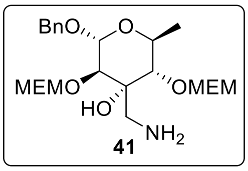

Azide **40** (952 mg, 1.96 mmol, 1.00 eq.) in dry THF (20 mL) was treated with PPh_3 (1.29 g, 4.90 mmol, 2.50 eq.) and stirred until TLC showed full consumption of starting material. H_2O (384 µL, 19.6 mmol, 10.0 eq.) was added and stirring was continued for 3 days. The volatiles were removed under reduced pressure and the crude product was purified by column chromatography (SiO_2, 15% MeOH in CH_2Cl_2 + 0.5% NEt_3→10 % MeOH in CH_2Cl_2+0.5% NEt_3). Amin **41** (772 mg, 86%) was isolated as a colourless oil, minor impurities occur due to regioisomers. **R_f** = 0.24 (CH_2Cl_2/MeOH 9:1); $[\alpha]_D^{20}$ −59.5° (c 1.0 in $CHCl_3$); **IR** ν_{max}/cm^{-1} 3495 (m), 2926 (m), 2882 (m), 1456 (m), 1363 (w), 1276 (m), 1261 (m), 1201 (w), 1111 (m), 1021 (s), 846 (m), 765 (s), 750 (s), 846 (m), 765 (s), 750 (s), 700 (m); major regioisomer **^{1}H-NMR** (500 MHz, $CDCl_3$) δ 7.39-7.26 (m, 5H), 4.97 (d, 1H, J = 0.9 Hz), 4.82 (d, 1H, J = 6.9 Hz), 4.81 (d, 1H, J = 7.0 Hz), 4.78 (d, 1H, J = 6.9 Hz), 4.76 (d, 1H, J = 11.8 Hz), 4.73 (d, 1H, J = 7.0 Hz), 4.55 (d, 1H, J = 11.8 Hz), 3.98 (dq, 1H, J = 6.4, 9.8 Hz), 3.82 (d, 1H, J = 1.4 Hz), 3.80 (ddd, 1H, J = 3.4, 5.6, 10.9 Hz), 3.76-3.69 (m, 3H), 3.59-3.46 (m, 4H), 3.41 (d, 1H, J = 9.8 Hz), 3.39 (s, 3H), 3.37 (s, 3H), 2.97 (d, 1H, J = 13.3 Hz), 2.82 (d, 1H, J = 13.3 Hz), 1.89 (br. s, 3H), 1.31 (d, 3H, J = 6.4 Hz) ppm; major regioisomer **^{13}C-NMR** (125 MHz, $CDCl_3$) δ 136.5, 128.8, 128.4, 128.3, 98.0, 97.9, 95.8, 80.3, 75.0, 74.6, 71.8, 71.7, 69.9, 68.5, 68.0, 64.3, 59.24, 59.21, 44.9, 18.1 ppm; **HRMS** ESI *m/z* $[M + H]^+$ calcd. for $C_{22}H_{38}NO_9$ 460.25411 found 460.25302.

N-(((2*R*,3*R*,4*S*,5*S*,6*S*)-2-(Benzyloxy)-4-hydroxy-3,5-bis((2-methoxyethoxy)methoxy)-6-methyltetrahydro-2*H*-pyran-4-yl)methyl)-3,4-dichloro-5-methyl-1*H*-pyrrole-2-carboxamide (42)

To a solution of amin **41** (42.0 mg, 91.4 µmol, 1.00 eq.) and carbonic acid **31** (21.3 mg, 110 µmol, 1.20 eq.) in dry CH_2Cl_2 (1 mL) was added dry NEt_3 (31.8 µL, 228 µmol, 2.50 eq.), EDC·HCl (26.3 mg, 137 µmol, 1.50 eq.) and HOBt (16.8 mg, 110 µmol, 1.20 eq.) at 0 °C. The

mixture was allowed to warm to room temperature overnight. Reaction was quenched by addition of sat. aq. $NaHCO_3$ solution. Aqueous phase was extracted with EtOAc thrice and combined organic phases were dried over Na_2SO_4. Removal of solvents under reduced pressure and purification by column chromatography (SiO_2, pentane/EtOAc 1:1→CH_2Cl_2/MeOH 50:1) gave amide **42** (47.2 mg, 81%) as a light red oil. Minor impurities occur due to regioisomers. **R$_f$** = 0.35 (hexanes/EtOAc 1:1); $[\alpha]_D^{20}$ −42.7° (c 1.0 in $CHCl_3$); **IR** ν_{max}/cm^{-1} 3407 (m), 3208 (m), 2924 (m), 2882 (m), 1629 (m), 1533 (m), 1455 (m), 1417 (w), 1379 (w), 1276 (m), 1262 (m), 1113 (m), 1024 (s9, 847 (m), 764 (s), 750 (s), 700 (m); major regioisomer **^{1}H-NMR** (500 MHz, $CDCl_3$) δ 9.41 (m, 1H), 7.38-7.27 (5H, m), 7.23 (m, 1H), 5.02 (d, 1H, J = 1.3 Hz), 4.83 (d, 1H, J = 7.0 Hz), 4.78 (d, 1H, J = 7.0 Hz), 4.75 (d, 1H, J = 11.8 Hz), 4.71 (d, 1H, J = 7.3 Hz), 4.64 (d, 1H, J = 7.3 Hz), 4.56 (d, 1H, J = 11.8 Hz), 4.34 (d, 1H, J = 1.6 Hz), 4.00 (m, 2H), 3.75 (t, 2H, J = 4.7 Hz), 3.71 (ddd, 1H, J = 2.9, 5.8, 10.9 Hz), 3.68 (m, 1H), 3.55 (m, 3H), 3.47 (d, 1H, J = 9.5 Hz), 3.46-3.36 (m, 2H), 3.34 (s, 3H), 3.32 (m, 1H), 3.29 (s, 3H), 2.29 (s, 3H), 1.58 (m, 1H), 1.33 (d, 3H, J = 6.3 Hz) ppm; major regioisomer **^{13}C-NMR** (125 MHz, $CDCl_3$) δ 159.8, 136.5, 128.7, 128.31, 128.27, 128.2, 118.6, 111.3, 110.2, 109.2, 98.4, 98.0, 96.8, 79.8, 76.3, 74.4, 71.7, 71.5, 70.0, 68.5, 67.4, 64.4, 59.2, 59.0, 41.9, 29.8, 18.1, 11.3 ppm; **HRMS** ESI m/z [M + H]$^+$ calcd. for $C_{28}H_{41}Cl_2N_2O_{10}$ 635.21328 found 635.21334.

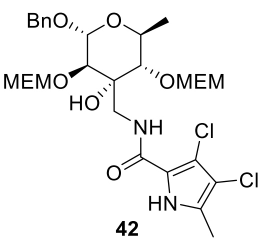

42

3,4-Dichloro-5-methyl-*N*-(((2*R*,3*R*,4*S*,5*S*,6*S*)-2,3,4,5-tetrahydroxy-6-methyltetrahydro-2*H*-pyran-4-yl)methyl)-1*H*-pyrrole-2-carboxamide (43)

Carbohydrate **42** (24.3 mg, 37.8 µmol, 1.00 eq.) in dry CH_2Cl_2 (1 mL) was treated dropwise with BCl_3 (1 M CH_2Cl_2, 453 µL, 12.0 eq.) at −78 °C. The solution was stirred at this temperature for 2 h, before BCl_3 (113 µL, 4.00 eq.) was added again. Stirring was continued for 1.5 h and H_2O was added to stop the reaction. All volatiles were removed at the rotary evaporator and the crude product was purified by column chromatography (SiO_2, CH_2Cl_2 19:1→10:1 MeOH in CH_2Cl_2). This yielded the product **43** (13.3 mg, 93%, α:β 1.7:1) as a colourless foam. **R$_f$** = 0.37 (CH_2Cl_2/MeOH 9:1); $[\alpha]_D^{20}$+7.17° (c 1.0 in $CHCl_3$); IR ν_{max}/cm^{-1} 3310 (s), 2925 (s), 2530 (m), 1606 (s), 1499 (s), 1450 (s), 1323 (m), 1272 (m), 1164 (m), 1071 (s), 761 (m); major regioisomer, α-anomer **^{1}H-NMR** (500 MHz, CD_3OD) δ 5.06 (d, 1H, J = 1.2 Hz), 3.87 (m, 1H), 3.71 (dq, 1H, J = 6.2, 9.5 Hz), 3.52 (m, 1H), 3.46 (d, 1H, J = 1.0 Hz),

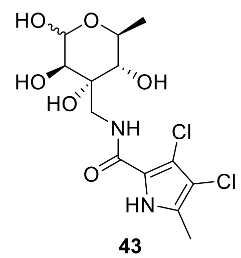

43

3.34 (d, 1H, J = 9.5 Hz), 2.23 (s, 3H), 1.27 (d, 3H, J = 6.2 Hz) ppm; major regioisomer, β-anomer **^{1}H-NMR** (500 MHz, CD_3OD) δ 4.96 (d, 1H, J = 1.2 Hz), 4.02 (dq, 1H, J = 6.2, 9.7 Hz), 3.89 (m, 1H), 3.57 (d, J = 1.5 Hz), 3.55 (m, 1H), 3.40 (d, 1H, J = 9.7 Hz), 2.23 (s, 3H), 1.29 (d, 3H, J = 6.2 Hz) ppm; major regioisomer **^{13}C-NMR** (125 MHz, CD_3OD) δ 162.3, 129.7, 119.6, 112.4, 96.3, 75.7, 72.5, 70.6, 65.6, 44.4, 18.2, 10.8 ppm; minor regioisomer **^{13}C-NMR** (125 MHz, CD_3OD) δ 162.4, 129.6, 119.6, 110.7, 93.6, 75.5, 73.2, 72.6, 71.3, 45.2, 18.3, 14.5 ppm; **HRMS** ESI *m/z* $[M + H]^+$ calcd. for $C_{13}H_{19}Cl_2N_2O_6$ 369.06147 found 369.06091.

The vinyl group in **37** is amenable to a good many other functionalisations, e.g., dihydroxylations affording vicinal diols such as **SI-28**, Wacker-type oxidations leading to methyl ketones such as **SI-29**, hydroborations to give primary alcohols like **SI-30**, or Grubbs-catalysed metathesis to non-terminal alkenes like **SI-31** (Scheme S6).

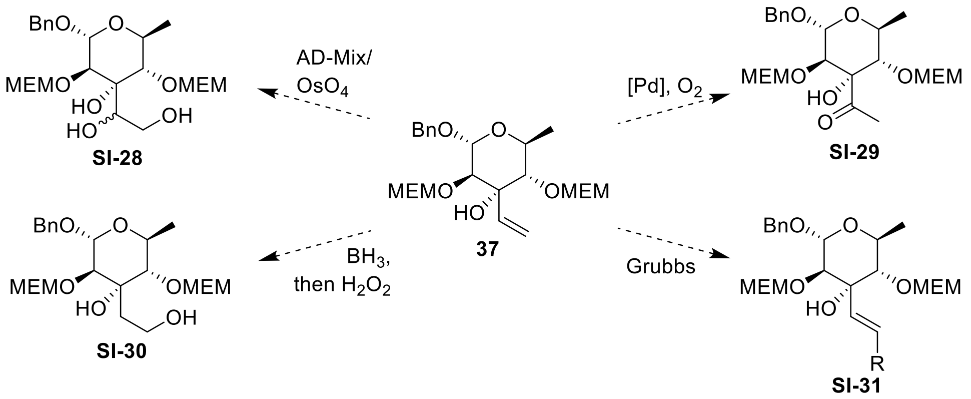

Scheme S6. Possible transformations of olefin **37** as a common intermediate

2.5 Synthesis of decalin fragment **5**

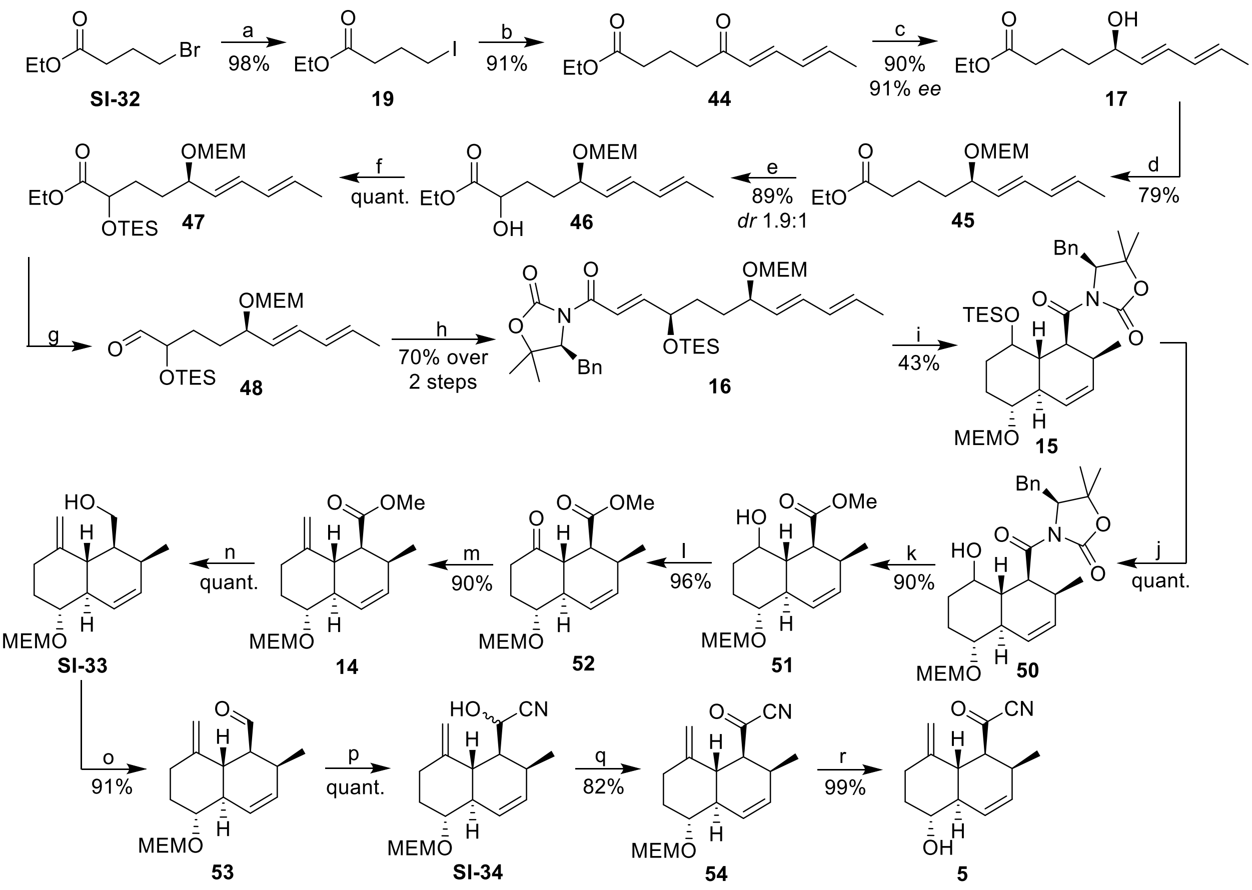

Scheme S7. Synthesis of decalin core **5**.

Reagents and conditions: a) NaI, acetone, reflux, 21 h; b) 1. **19**, Zn, THF, reflux, 3.5 h, 2. Thioester **18**, $Pd(PPh_3)_4$, toluene, rt, 23 h; c) 1. (*S*)-CBS-catalyst, BH_3·THF, rt, 1 h, 2. **44**, −35 °C, 3.5 h; d) MEMCl, DIPEA, CH_2Cl_2, 40 °C, 23 h; e) 1. KHMDS, THF, −78 °C, 30 min, 2. MoOPH, −78 °C, 4 h; f) TESCl, imidazole, DMAP, CH_2Cl_2, 0 °C→40 °C, 4.5 h; g) DIBAL, toluene, −78 °C, 5 h; h) 1. LiHMDS, phosphonate **49**, THF, 0 °C, 1 h, 2. **48**, 0 °C→rt, 17 h; i) toluene, 80 °C, 3 d; j) HF·py, THF, 0 °C, 15 h; k) NaOMe, CH_2Cl_2, 0 °C, 3 h; l) DMP, $NaHCO_3$, CH_2Cl_2, 0 °C→rt, 3 h; m) 1. $MePPh_3Br$, KO*t*Bu, THF, 0 °C, 45 min, 2. **52**, THF, 0 °C→rt, 3 h; n) DIBAL, CH_2Cl_2, 0 °C, 5 h; o) DMP, $NaHCO_3$, CH_2Cl_2, 0 °C→rt, 3 h; p) 1. TMSCN, NEt_3, CH_2Cl_2, 0 °C→rt, 4 h 20 min, 2. NH_4F, EtOH, 0 °C, 2 h; q) DMP, CH_2Cl_2, 0 °C, 1.5 h; r) $LiBF_4$, $MeCN/H_2O$, rt→55 °C, 4.5 h.

Ethyl 4-iodobutanoate (19)

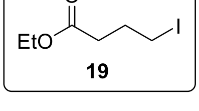

Bromo-butyric acid ester **SI-32** (20.0 mL, 133 mmol, 1.00 eq.) dissolved in acetone *p.a.* (1.3 L) was treated with NaI (100 g, 667 mmol, 5.00 eq.) at room temperature. The mixture was stirred under reflux for 21 h. The suspension was filtered off over celite® and washed with Et_2O. The filtrate was washed with H_2O. The aqueous phase was reextracted with Et_2O thrice and dried over Na_2SO_4. Removal of the solvent and purification by column chromatography (SiO_2, pentane→pentane/EtOAc 30:1) furnished

iodide **19** (31.6 g, 98%) as a yellow liquid. **R$_f$** = 0.61 (hexanes/EtOAc 98:2); **IR** ν_{max}/cm^{-1} 2981 (m), 2936 (w), 2908 (w), 1732 (s), 1444 (m), 1374 (m), 1352 (w), 1308 (w), 1226 (m), 1192 (s), 1163 (m), 1121 (m), 1097 (w), 1032 (m), 857 (w), 769 (w); **^{1}H-NMR** (500 MHz, $CDCl_3$) δ 4.13 (q, 2H, J = 7.1 Hz), 3.24 (t, 2H, J = 6.7 Hz), 2.44 (t, 2H, J = 7.1 Hz), 2.13 (qn, 2H, J = 7.0 Hz), 1.26 (t, 3H, J = 7.1 Hz) ppm.

Spectroscopic data corresponded to those reported in the literature.[6]

Ethyl (6*E*,8*E*)-5-oxodeca-6,8-dienoate (44)

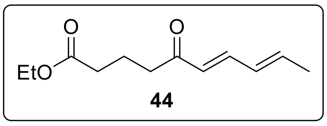

A solution of iodide **19** (26.7 g, 109 mmol, 3.00 eq.) in dry THF (120 mL) was treated with Zn (14.1 g, 215 mmol, 5.90 eq.) and stirred under reflux for 3.5 h. This mixture was added to a solution of thioester **18** (5.99 g, 36.3 mmol, 1.00 eq.) in dry. toluene (125 mL) at room temperature. The mixture was treated with $Pd(PPh_3)_4$ (2.10 g, 1.82 mmol, 0.05 eq.) and stirred for 23 h at room temperature. The solids were filtered off over celite® and the organic phases were washed with 1 M HCl, sat. aq. $NaHCO_3$ solution as well as brine and dried over Na_2SO_4. The solvents were removed under vacuum and the crude product was purified by column chromatography (SiO_2, pentane/EtOAc 9:1→8:1) to give product **44** (6.93 g, 91%) as a light-yellow oil. **R$_f$** = 0.68 (hexanes/EtOAc 8:1); IR ν_{max}/cm^{-1} 2979 (m), 2940 (m), 1732 (s), 1687 (m), 1664 (m), 1639 (m), 1596 (m), 1447 (w), 1418 (w), 1376 (m), 1323 (w), 1197 (m), 1100 (m), 1028 (m), 1000 (m), 949 (w), 858 (w); **^{1}H-NMR** (500 MHz, $CDCl_3$) δ 7.13 (m, 1H), 6.19 (m, 2H), 6.05 (d, 1H, J = 15.4 Hz), 4.12 (q, 2H, J = 7.2 Hz), 2.62 (t, 2H, J = 7.2 Hz), 2.35 (t, 2H, J = 7.2 Hz), 1.94 (qn, 2H, J = 7.3 Hz), 1.86 (d, 3H, J = 4.9 Hz), 1.25 (t, 3H, J = 7.3 Hz) ppm; **^{13}C-NMR** (125 MHz, $CDCl_3$) δ 200.1, 173.4, 143.2, 140.6, 130.4, 127.7, 60.5, 39.4, 33.6, 19.6, 19.0, 14.4 ppm; **HRMS** ESI m/z $[M + H]^+$ calcd. for $C_{12}H_{19}O_3$ 211.13287 found 211.13260.

Ethyl (*R*,6*E*,8*E*)-5-hydroxydeca-6,8-dienoate (17)

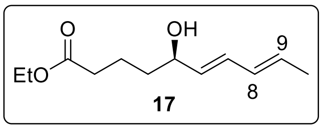

A solution of (*S*)-CBS-catalyst (3.95 g, 14.3 mmol, 1.50 eq.) in dry THF (90 mL) was treated with $BH_3 \cdot THF$ (10.5 mL, 10.5 mmol, 1.10 eq.) at room temperature. After stirring for 1 h, ketone **44** (2.00 g, 9.51 mmol, 1.00 eq.) was added dissolved in dry THF (22 mL) at -35 °C over 1.5 h. The reaction was stirred for a further 2h and quenched with sat. aq. NH_4Cl solution. The phases

were separated, and the organic phase was washed with sat. aq. NH_4Cl solution again. The combined aqueous phases were reextracted with Et_2O twice, the combined organic phases were washed with brine and dried over Na_2SO_4. The volatiles were removed under reduced pressure. Column chromatography (SiO_2, pentane/EtOAc 8:1→6:1→5:1→4:1→3:1) gave product **17** (1.82 g, 90%, 91% *ee*, E/Z 11:1) as a light-yellow liquid. E/Z isomerization occurred at double bond between position 8 and 9. **R$_f$** = 0.30 (hexanes/EtOAc 4:1); $[\alpha]_D^{20}$ −6.97° (c 1.0 in $CHCl_3$); **IR** ν_{max}/cm^{-1} 3439 (m), 2985 (m), 2935 (m), 2875 (w), 1732 (s), 1448 (m), 1374 (m), 1276 (s), 1261 (s), 1163 (m), 1099 (m), 1030 (m), 990 (m), 860 (w), 765 (s), 750 (s); *E,E*-isomer **^{1}H-NMR** (500 MHz, $CDCl_3$) δ 6.18 (dd, 1H, *J* = 10.5, 15.2 Hz), 6.03 (ddq, 1H, *J* = 1.4, 10.5, 15.0 Hz), 5.71 (dq, 1H, *J* = 6.7, 15.0 Hz), 5.55 (dd, 1H, *J* = 7.1, 15.2 Hz), 4.12 (q, 2H, *J* = 7.2 Hz), 4.12 (m, 1H), 2.33 (t, 2H, *J* = 7.3 Hz), 1.75 (dd, 3H, *J* = 1.4, 6.7 Hz), 1.74-1.52 (m, 4H), 1.25 (t, 3H, *J* = 7.1 Hz) ppm; significant signals *E,Z*-isomer **^{1}H-NMR** (500 MHz, $CDCl_3$) δ 6.53 (ddt, 1H, *J* = 0.9, 11.1, 15.2 Hz), 6.00 (m, 1H), 5.66 (m, 1H), 5.52 (m, 1H), 4.19 (m, 1H), 1.25 (t, 3H, *J* = 7.1 Hz) ppm; *E,E*-isomer **^{13}C-NMR** (125 MHz, $CDCl_3$) δ 173.7, 132.9, 131.2, 130.7, 130.2, 72.4, 60.3, 36.6, 34.1, 20.9, 18.1, 14.3 ppm; significant signals *E,Z*-isomer **^{13}C-NMR** (125 MHz, $CDCl_3$) δ 135.2, 128.5, 127.2, 125.9, 72.5 ppm; **HRMS** ESI *m/z* [M − OH]$^+$ calcd. for $C_{12}H_{19}O_2$ 195.13796 found 195.13789.

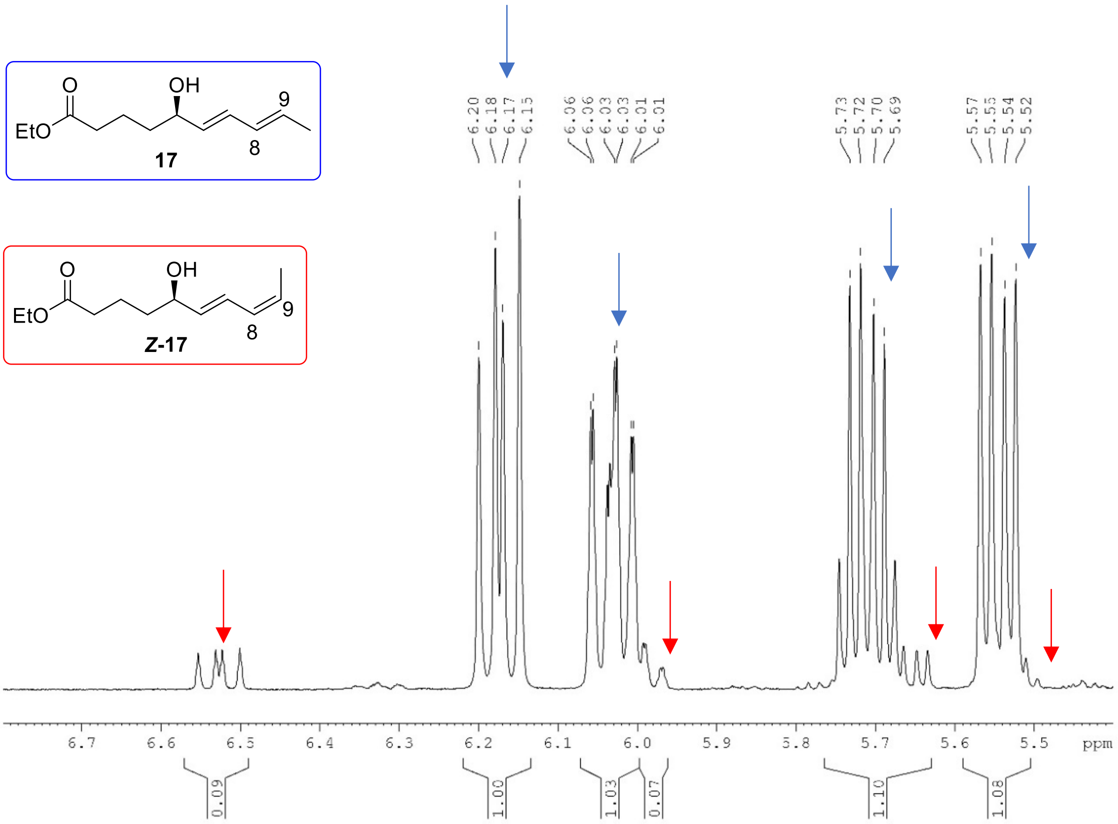

Fig. S6. Differentiation of **17** and *Z*-**17** in ^{1}H-NMR-spectrum.

Ethyl (*R*,6*E*,8*E*)-5-((2-methoxyethoxy)methoxy)deca-6,8-dienoate (45)

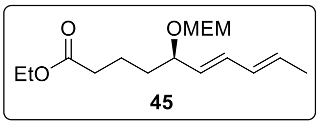

Alcohol **17** (2.28 g, 10.7 mmol, 1.00 eq.) in dry CH_2Cl_2 (100 mL) was treated with MEMCl (2.46 mL, 21.5 mmol, 2.00 eq.) and DIPEA (5.48 mL, 32.2 mmol, 3.00 eq.) at room temperature. The solution was stirred for 23 h at 40 °C. 0.5M HCl was added, and the aqueous phase was extracted with EtOAc thrice. The combined organic phases were washed with brine and dried over Na_2SO_4. Removal of the solvent under vacuum and purification by column chromatography (SiO_2, pentane/EtOAc 7:1→5:1) gave MEM-protected alcohol **45** (2.55 g, 79%) as a colourless liquid in 79% yield. **R_f** = 0.43 (hexanes/EtOAc 4:1); $[\alpha]_D^{20}$ −96.0° (c 1.0 in $CHCl_3$); **IR** ν_{max}/cm^{-1} 2977 (m), 2931 (m), 2879 (m), 1733 (s), 1451 (m), 1372 (m), 1276 (m), 1260 (m), 1178 (m), 1135 (m), 1089 (m), 1023 (s), 990 (s), 931 (w), 852 (m), 765 (s), 750 (s); *E,E*-isomer **^{1}H-NMR** (500 MHz, $CDCl_3$) δ 6.15 (dd, 1H, *J* = 10.5, 15.3 Hz), 6.02 (ddq, 1H, *J* = 1.3, 10.5, 15.1 Hz), 5.70 (dq, 1H, *J* = 6.8, 15.1 Hz), 5.33 (dd, 1H, *J* = 8.2, 15.3 Hz), 4.76 (d, 1H, *J* = 7.1 Hz), 4.61 (d, 1H, *J* = 7.1 Hz), 4.11 (q, 2H, *J* = 7.1 Hz), 4.04 (m, 1H), 3.79 (ddd, 1H, *J* = 2.9, 4.9, 10.3 Hz), 3.60 (m, 1H), 3.55 (m, 2H), 3.39 (s, 3H), 2.30 (t, 2H, *J* = 7.4 Hz), 1.74 (dd, 3H, *J* = 1.3, 6.8 Hz), 1.73-1.48 (m, 4H), 1.24 (t, 3H, *J* = 7.1 Hz) ppm; significant signals *E,Z*-isomer **^{1}H-NMR** (500 MHz, $CDCl_3$) δ 6.49 (ddt, 1H, *J* = 0.9, 11.1, 15.3 Hz), 5.98 (m, 1H), 5.51 (dqu, 1H, *J* = 7.0, 10.7 Hz), 5.33 (dd, 1H, *J* = 8.0, 15.3 Hz), 4.79 (d, 1H, *J* = 7.1 Hz), 4.63 (d, 1H, *J* = 7.1 Hz), 4.12 (q, 1H, 7.1 Hz), 4.11 (m, 1H), 3.82 (m, 1H), 3.65 (m, 1H), 3.57 (m, 2H), 3.39 (s, 3H), 2.31 (m, 2H), 1.25 (t, 3H, *J* = 7.1 Hz) ppm; *E,E*-isomer **^{13}C-NMR** (125 MHz, $CDCl_3$) δ 173.7, 133.7, 130.8, 130.5, 130.0, 92.6, 76.2, 71.9, 67.0, 60.4, 59.2, 35.2, 34.3, 21.1, 18.3, 14.4 ppm; significant signals *E,Z*-isomer **^{13}C-NMR** (125 MHz, $CDCl_3$) δ 132.4, 128.6, 128.4, 127.4, 92.7, 76.4, 67.1 ppm; **HRMS** ESI *m/z* $[M + Na]^+$ calcd. for $C_{16}H_{28}O_5Na$ 323.18290 found 323.18275.

Ethyl (5*R*,6*E*,8*E*)-2-hydroxy-5-((2-methoxyethoxy)methoxy)deca-6,8-dienoate (46)

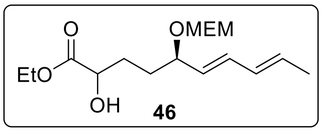

Ester **45** (2.50 g, 8.32 mmol, 1.00 eq.) was dissolved in dry THF (83 mL) and treated with KHMDS (12.5 mL, 12.5 mmol, 1.50 eq.) at −78 °C. The solution was stirred for 30 min, before MoOPH (4.04 g, 12.5 mmol, 1.50 eq.) was added. Another portion of MoOPH (1.35 g, 4.16 mmol, 0.5 eq.) was added after 2.5 h of stirring at −78 °C. Stirring was continued for 1.5 h and the reaction was quenched with sat. aq. NH_4Cl solution and sat. aq. $Na_2S_2O_3$ solution. The aqueous

phase was extracted thrice with EtOAc, organic phases were washed with H_2O, brine and dried over Na_2SO_4. Crude product was purified by column chromatography (SiO_2, pentane/EtOAc 4:1→3:1) to yield α-hydroxylated ester **46** (2.34 g, 89%, *dr* 1.6:1) as a colourless liquid. $\mathbf{R_f}$ = 0.24 (hexanes/EtOAc 4:1); $[\alpha]_D^{20}$ −93.9° (c 1.0 in $CHCl_3$); **IR** ν_{max}/cm^{-1} 3462 (w), 2980 (m), 2933 (m), 2884 (m), 1735 (m), 1449 (w), 1368 (w), 1261 (m), 1276 (m), 1199 (m), 1103 (m), 1024 (m), 991 (m), 853 (w), 764 (s), 750 (s); *E,E*-isomer major diastereomer **^{1}H-NMR** (500 MHz, $CDCl_3$) δ 6.16 (dd, 1H, *J* = 10.5, 15.2 Hz), 6.02 (dd, 1H, *J* = 10.4, 15.0 Hz), 5.72 (dq, 1H, *J* = 6.8, 15.0 Hz), 5.34 (m, 1H), 4.76 (d, 1H, *J* = 6.9 Hz), 4.62 (d, 1H, *J* = 6.9 Hz), 4.23 (m, 2H), 4.18 (m, 1H), 4.09 (m, 1H), 3.80 (m, 1H), 3.60 (m, 1H), 3.55 (m, 2H), 3.39 (s, 3H), 2.93 (m, 1H), 1.88 (m, 1H), 1.75 (d, 3H, *J* = 6.8 Hz), 1.78-1.58 (m, 3H), 1.29 (t, 3H, *J* = 7.1 Hz) ppm; significant signals *E,E*-isomer minor diastereomer **^{1}H-NMR** (500 MHz, $CDCl_3$) δ 4.62 (d, 1H, *J* = 7.0 Hz), 3.39 (s, 3H), 2.89 (m, 1H), 1.29 (t, 3H, *J* = 7.1 Hz) ppm; significant signals *E,Z*-isomer major diastereomer **^{1}H-NMR** (500 MHz, $CDCl_3$) δ 6.49 (dd, 1H, *J* = 11.0, 15.2 Hz), 5.98 (m, 1H), 5.52 (dq, 1H, *J* = 7.1, 10.6 Hz), 5.34 (m, 1H), 4.78 (d, 1H, *J* = 7.1 Hz), 4.64 (d, 1H, *J* = 7.1 Hz), 3.40 (s, 3H) ppm; *E,E*-isomer major diastereomer **^{13}C-NMR** (125 MHz, $CDCl_3$) δ 175.1, 133.6, 130.6, 130.4, 129.6, 92.5, 76.2, 71.8, 70.4, 67.0, 61.6, 59.1, 31.1, 30.4, 18.1, 14.2 ppm; significant signals *E,E*-isomer minor diastereomer **^{13}C-NMR** (125 MHz, $CDCl_3$) δ 175.1, 133.6, 130.6, 130.4, 129.6, 92.5, 76.0, 71.8, 70.1, 67.0, 61.7, 59.1, 30.7, 30.2 ppm; significant signals *E,Z*-isomer major diastereomer **^{13}C-NMR** (125 MHz, $CDCl_3$) δ 133.1, 128.5, 128.3, 127.3, 92.6, 76.4 ppm; **HRMS** ESI *m/z* $[M + Na]^+$ calcd. for $C_{16}H_{28}O_6Na$ 339.17781 found 339.17700.

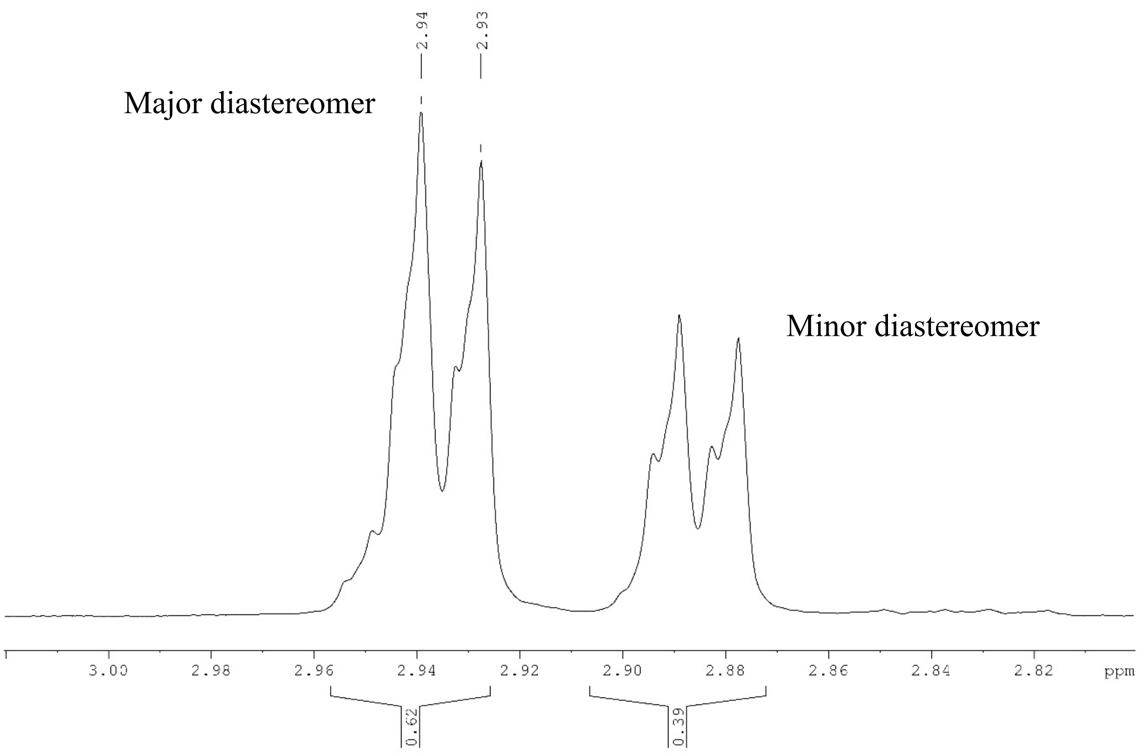

Fig. S7. Significant signals in ^{1}H-NMR-spectrum of ester **46**.

Ethyl (5*R*,6*E*,8*E*)-5-((2-methoxyethoxy)methoxy)-2-((triethylsilyl)oxy)deca-6,8-dienoate (47)

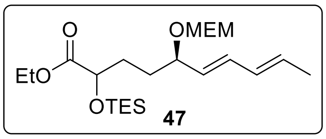

To a solution of α-hydroxylated ester **46** (2.29 g, 7.22 mmol, 1.00 eq.) in dry CH_2Cl_2 (72 mL) TESCl (2.42 mL, 14.4 mmol, 2.00 eq.), imidazole (1.47 g, 21.7 mmol, 3.00 eq.) and DMAP (88.2 mg, 722 µmol, 0.10 eq.) were added at 0 °C. The suspension was stirred at 40 °C for 4.5 h. Sat. aq. NH_4Cl solution was added. The aqueous phase was extracted with CH_2Cl_2 thrice and organic phases were dried over Na_2SO_4. The crude product was purified by column chromatography (SiO_2, pentane/EtOAc 8:1) to give TES-protected α-hydroxylated ester **47** (3.27 g, quant.) as a colourless liquid. **R**$_f$ = 0.24 (hexanes/EtOAc 4:1); $[\alpha]_D^{20}$ −61.7° (c 1.0 in $CHCl_3$); **IR** ν_{max}/cm^{-1} 2956 (m), 2914 (m), 2878 (m), 1752 (m), 1726 (m), 1458 (m), 1276 (m), 1261 (m), 1134 (m), 1023 (m), 990 (m), 764 (s), 750 (s); *E,E*-isomer major diastereomer **^{1}H-NMR** (500 MHz, $CDCl_3$) δ 6.14 (dd, 1H, *J* = 10.5, 15.2 Hz), 6.02 (ddq, 1H, *J* = 1.4, 10.4, 15.0 Hz), 5.69 (dq, 1H, *J* = 6.8, 15.0 Hz), 5.33 (dd, 1H, *J* = 8.2, 15.2 Hz), 4.76 (d, 1H, *J* = 6.9 Hz), 4.61 (d, 1H, *J* = 6.9 Hz), 4.17 (m, 3H), 4.04 (m, 1H), 3.77 (m, 1H), 3.59 (m, 1H), 3.55

(m, 2H), 3.38 (s, 3H), 1.89-1.76 (m, 1H), 1.75 (d, 3H, *J* = 6.5 Hz), 1.73-1.58 (m, 3H), 1.27 (t, 3H, *J* = 7.1 Hz), 0.95 (t, 9H, *J* = 8.0 Hz), 0.61 (q, 6H, *J* = 8.0 Hz) ppm; significant signals *E,E*-isomer minor diastereomer **^{1}H-NMR** (500 MHz, $CDCl_3$) δ 4.76 (d, 1H, *J* = 6.9 Hz), 3.39 (s, 3H), 1.75 (d, 3H, *J* = 6.7 Hz), 1.27 (t, 3H, *J* = 7.1 Hz) ppm; significant signals *E,Z*-isomer major diastereomer **^{1}H-NMR** (500 MHz, $CDCl_3$) δ 6.48 (dd, 1H, *J* = 11.0, 15.3 Hz), 5.98 (m, 1H), 5.51 (dq, 1H, *J* = 7.2, 10.8 Hz), 5.44 (dd, 1H, *J* = 8.2, 15.2), 4.77 (d, 1H, *J* = 7.1 Hz), 4.63 (d, 1H, *J* = 7.1 Hz), 4.10 (m, 1H), 3.39 (s, 3H) ppm; *E,E*-isomer major diastereomer **^{13}C-NMR** (125 MHz, $CDCl_3$) δ 173.8, 133.7, 130.8, 130.4, 129.9, 92.6, 76.6, 72.2, 71.9, 67.0, 60.9, 59.2, 31.5, 31.4, 18.3, 14.4, 6.86, 4.71 ppm; significant signals *E,E*-isomer minor diastereomer **^{13}C-NMR** (125 MHz, $CDCl_3$) δ 130.8, 130.4, 129.9, 92.5, 76.1, 71.8, 31.2, 31.1 ppm; significant signals *E,Z*-isomer major diastereomer **^{13}C-NMR** (125 MHz, $CDCl_3$) δ 132.34, 132.29, 128.7, 128.4 ppm; **HRMS** ESI *m/z* $[M + Na]^+$ calcd. for $C_{22}H_{42}O_6SiNa$ 453.26429 found 453.26346.

(S)-4-Benzyl-3-((2*E*,4*R*,7*R*,8*E*,10*E*)-7-((2-methoxyethoxy)methoxy)-4-((triethylsilyl)oxy)-dodeca-2,8,10-trienoyl)-5,5-dimethyloxazolidin-2-one (16)

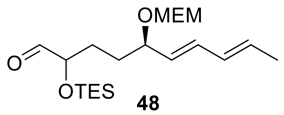
48

Ester **47** (1.20 g, 2.79 mmol, 1.00 eq.) in dry toluene (28 mL) was treated dropwise with DIBAL (4.18 mL, 4.18 mmol, 1.50 eq.) at −78 °C. The reaction was stirred at this temperature for 5 h, before it was stopped by addition of acetone (1 mL) and sat. aq. Na,K-tartrate solution. The two-phase mixture was stirred vigorously at room temperature for 2 h. The organic phase was separated, and the aqueous phase was extracted with EtOAc four times. The combined organic phases were washed with H_2O and dried over Na_2SO_4. Aldehyde **48** was used without further purification. **R_f** = 0.24 (hexanes/EtOAc 4:1); **IR** ν_{max}/cm^{-1} 3435 (w), 2954 (m), 2933 (m), 2908 (m), 2877 (m), 1731 (m), 1696 (w), 1457 (m), 1414 (m), 1367 (m), 1240 (m), 1199 (w), 1104 (s), 1042 (s), 1018 (s), 975 (s), 849 (m), 809 (m), 741 (s); *E,E*-isomer major diastereomer **^{1}H-NMR** (500 MHz, $CDCl_3$) δ 9.58 (t, 1H, *J* = 1.6 Hz), 6.14 (dd, 1H, *J* = 10.4, 15.8 Hz), 6.02 (ddq, 1H, *J* = 1.4, 10.5, 15.0 Hz), 5.70 (dq, 1H, *J* = 6.8, 15.0 Hz), 5.32 (dd, 1H, *J* = 8.1, 15.2 Hz), 4.76 (d, 1H, *J* = 7.0 Hz), 4.61 (d, 1H, *J* = 7.0 Hz), 4.03 (m, 1H), 3.97 (m, 1H), 3.77 (m, 1H), 3.59 (m, 1H), 3.55 (m, 2H), 3.38 (s, 3H), 1.80-1.57 (m, 4H), 1.75 (d, 3H, *J* = 6.5 Hz), 0.95 (t, 9H, *J* = 7.9 Hz), 0.61 (q, 6H, *J* = 7.9 Hz) ppm; significant signals *E,Z*-isomer major diastereomer **^{1}H-NMR** (500 MHz, $CDCl_3$) δ 9.59 (t, 1H, *J* = 1.4 Hz), 6.48 (dd, 1H, *J* = 11.2, 15.2 Hz), 5.99 (m, 1H), 5.52 (dq, 1H, *J* = 7.1, 10.8 Hz), 5.44 (dd, 1H, *J* = 8.2, 15.2 Hz), 4.10 (m, 1H), 3.40 (s, 3H) ppm.

A solution of phosphonate **49** (1.24 g, 3.49 mmol, 1.25 eq.) in dry THF (7 mL) was treated with LiHMDS (3.35 mL, 3.35 mmol, 1.20 eq.) at 0 °C. After stirring for 1 h, crude aldehyde **48** (1.08 g, 2.79 mmol, 1.00 eq.) dissolved in dry THF (3 mL) was added dropwise. The mixture was allowed to warm to room temperature overnight. Sat. aq. NH_4Cl solution stopped the reaction after 17 h of stirring. The aqueous phase was extracted with EtOAc thrice, combined organic phases were washed with H_2O, brine and dried over Na_2SO_4. Removal of the solvent under vacuum and purification by column chromatography (SiO_2, pentane/EtOAc 8:1→6:1→4:1→2:1) furnished trien **16** (1.18 g, 70% over two steps) as a colourless oil. **R_f** = 0.38 (hexanes/EtOAc 4:1); $[\alpha]_D^{20}$+27.4° (c 1.0 in $CHCl_3$); **IR** ν_{max}/cm^{-1} 2952 (m), 2936 (m), 2877 (m), 1778 (s), 1687 (m), 1640 (w), 1497 (w), 1456 (w), 1354 (m), 1329 (w), 1274 (w), 1242 (w), 1207 (w), 1180 (w), 1159 (w), 1100 (s), 1040 (s), 821 (w), 729 (m), 702 (w); *E,E*-isomer major diastereomer **^{1}H-NMR** (500 MHz, $CDCl_3$) δ 7.39 (dt, 1H, *J* = 1.3, 15.3 Hz), 7.32-7.20 (m, 5H), 7.04 (ddd, 1H, *J* = 2.3, 5.2, 15.3 Hz), 6.14 (dd, 1H, *J* = 10.6, 15.2 Hz), 6.03 (dd, 1H, *J* = 10.6, 14.8 Hz), 5.69 (dq, 1H, *J* = 6.8, 14.8 Hz), 5.32 (dd, 1H, *J* = 8.3, 15.2 Hz), 4.76 (d, 1H, *J* = 7.0 Hz), 4.61 (d, 1H, *J* = 7.0 Hz), 4.55 (dt, 1H, *J* = 3.6, 9.6 Hz), 4.38 (m, 1H), 4.02 (m, 1H), 3.79 (m, 1H), 3.60 (m, 1H), 3.55 (m, 2H), 3.38 (s, 3H), 3.21 (m, 1H), 2.89 (tt, 1H, *J* = 6.0, 9.7 Hz), 1.75 (d, 3H, *J* = 6,7 Hz), 1.71-1.53 (m, 4H), 1.38 (s, 3H), 1.35 (s, 3H), 0.95 (t, 9H, *J* = 8.0 Hz), 0.61 (q, 6H, *J* = 8.0 Hz) ppm; significant signals *E,E*-isomer minor diastereomer **^{1}H-NMR** (500 MHz, $CDCl_3$) δ 4.76 (d, 1H, *J* = 6.9 Hz), 3.39 (s, 3H), 1.37 (s, 3H), 1.36 (s, 3H), 0.96 (t, 3H, *J* = 7.9 Hz) ppm; significant signals *E,Z*-isomer major diastereomer **^{1}H-NMR** (500 MHz, $CDCl_3$) δ 7.40 (dt, 1H, *J* = 1.5, 15.3 Hz), 6.48 (dd, 1H, *J* = 10.7, 15.3 Hz), 5.98 (m, 1H), 5.51 (dq, 1H, *J* = 7.1, 10.4 Hz), 5.44 (dd, 1H, *J* = 8.3, 15.3 Hz), 4.09 (m, 1H), 3.39 (s, 3H) ppm; *E,E*-isomer major diastereomer **^{13}C-NMR** (125 MHz, $CDCl_3$) δ 165.4, 152.8, 152.6, 137.2, 133.7, 130.8, 130.4, 130.0, 129.2, 128.8, 126.9, 119.6, 92.5, 82.2, 76.7, 72.0, 71.9, 67.0, 63.9, 59.2, 35.4, 33.5, 31.2, 28.8, 22.5, 18.3, 6.99, 4.95 ppm; significant signals *E,E*-isomer minor diastereomer **^{13}C-NMR** (125 MHz, $CDCl_3$) δ 165.3, 152.7, 137.3, 130.4, 130.0, 119.5, 82.2, 76.4, 71.8, 63.9, 33.4, 31.0, 28.7, 22.5 ppm; **HRMS** ESI *m/z* [M + Na]$^+$ calcd. for $C_{34}H_{53}NO_7SiNa$ 638.34835 found 638.34784.

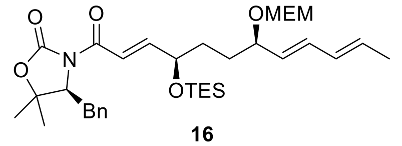

16

(4*S*)-4-Benzyl-3-((1*S*,2*S*,4a*R*,5*R*,8a*S*)-5-((2-methoxyethoxy)methoxy)-2-methyl-8-((triethylsilyl)oxy)-1,2,4a,5,6,7,8,8a-octahydronaphthalene-1-carbonyl)-5,5-dimethyl-oxazolidin-2-one (15)

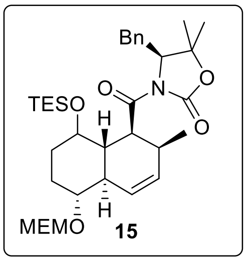

Trien **16** (513 mg, 833 µmol, 1.00 eq.) was dissolved in dry toluene (28 mL) and heated at 80 °C for 2 days. Temperature was raised to 100°C and stirring was continued for 1 d. The solvent was removed at the rotary evaporator. Crude product was purified by column chromatography (SiO_2, pentane/EtOAc 6:1→8:1) to give Diels-Alder-product **15** (219 mg, 43%, *de* >96%) as a colourless resin. **R$_f$** = 0.50 (hexanes/EtOAc 4:1); $[\boldsymbol{\alpha}]_D^{20}$+63.3° (c 1.0 in $CHCl_3$); **IR** ν_{max}/cm^{-1} 3030 (w), 2934 (m), 2876 (m), 1776 (s), 1690 (m), 1497 (w), 1456 (m), 1393 (w), 1374 (m), 1352 (m), 1301 (w), 1273 (m), 1242 (m), 1207 (w), 1221 (w), 1180 (w); 1159 (w), 1129 (w), 1101 (s), 1086 (s), 1039 (s), 1005 (s), 984 (m), 919 (m), 882 (w), 839 (w), 821 (m), 805 (w), 764 (w), 727 (s), 702 (s); **^{1}H-NMR** (500 MHz, $CDCl_3$) δ 7.32 (d, 4H, J = 4.4 Hz), 7.24 (sex, 1H, J = 4.4 Hz), 5.88 (d, 1H, J = 10.0 Hz), 5.61 (ddd, 1H, J = 2.6, 4.8, 10.0 Hz), 4.89 (d, 1H, J = 7.1 Hz), 4.75 (d, 1H, J = 7.1 Hz), 4.57 (dd, 1H, J = 2.3, 11.0 Hz), 4.30 (s, 1H), 4.05 (dd, 1H, J = 5.9, 11.2 Hz), 3.78 (dt, 1H, J = 4.6, 11.1 Hz), 3.73 (dt, 1H, J = 4.6, 11.1 Hz), 3.58 (t, 2H, J = 4.6 Hz), 3.39 (s, 3H), 3.33 (dd, 1H, J = 2.1, 14.3 Hz), 3.24 (dt, 1H, J = 4.4, 10.7 Hz), 2.84 (m, 1H), 2.79 (dd, 1H, J = 11.2, 14.3 Hz), 2.53 (tq, 1H, J = 2.0, 10.7 Hz), 1.93 (m, 1H), 1.81 (dq, 1H, J = 3.2, 14.0 Hz), 1.77-1.67 (m, 2H), 1.55 (m, 1H), 1.33 (d, 6H, J = 6.9 Hz), 0.93 (t, 9H, J = 7.9 Hz), 0.85 (d, 3H, J = 7.1 Hz), 0.60-0.46 (m, 6H) ppm; **^{13}C-NMR** (125 MHz, $CDCl_3$) δ 174.6, 151.8, 137.3, 131.1, 129.0, 128.9, 126.9, 126.2, 94.8, 81.5, 79.6, 71.9, 67.2, 66.0, 64.0, 59.2, 43.7, 39.0, 38.8, 35.2, 31.9, 31.0, 29.3, 27.0, 23.2, 17.7, 7.16, 5.43 ppm; **HRMS** ESI *m/z* $[M + Na]^+$ calcd. for $C_{34}H_{53}NO_7SiNa$ 638.34835 found 638.34778.

(4*S*)-4-Benzyl-3-((1*S*,2*S*,4a*R*,5*R*,8a*S*)-8-hydroxy-5-((2-methoxyethoxy)methoxy)-2-methyl-1,2,4a,5,6,7,8,8a-octahydronaphthalene-1-carbonyl)-5,5-dimethyloxazolidin-2-one (50)

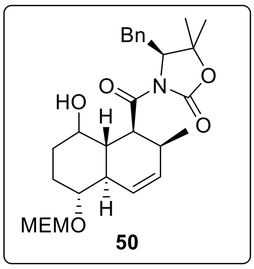

Diels-Alder product **15** (198 mg, 321 µmol, 1.00 eq.) was dissolved in THF *p.a.* (3.2 mL) and treated with HF·pyridine (459 µL, 17.7 mmol, 55.0 eq.) at 0 °C. The solution was stirred 15 h at this temperature and quenched with sat. aq. $NaHCO_3$ solution. The aqueous phase was extracted with EtOAc four times, combined organic phases were washed

with brine and dried over Na_2SO_4. The deprotected alcohol **50** (161 mg, quant.) was used without further purification. **R$_f$** = 0.42 (hexanes/EtOAc 1:1); $[\alpha]_D^{20}$+69.5° (c 1.0 in MeOH); **IR** ν_{max}/cm^{-1} 3485 (w), 2927 (m), 2880 (m), 1775 (s), 1692 (m), 1497 (w), 1455 (m), 1394 (m), 1373 (m), 1353 (m), 1297 (m), 1276 (m), 1230 (m), 1207 (m), 1176 (m), 1159 (m), 1101 (s), 1087 (s), 1036 (s), 956 (m), 921 (w), 844 (w), 822 (w), 766 (w), 730 (s), 700 (m); **^{1}H-NMR** (500 MHz, $CDCl_3$) δ 7.32-7.27 (m, 4H), 7.23 (m, 1H), 5.88 (d, 1H, *J* = 10.0 Hz), 5.63 (ddd, 1H, *J* = 2.6, 4.6, 10.0 Hz), 4.88 (d, 1H, *J* = 7.1 Hz), 4.74 (d, 1H, *J* = 7.1 Hz), 4.56 (dd, 1H, *J* = 4.0, 9.7 Hz), 4.10 (m, 1H), 4.07 (dd, 1H, *J* = 5.8, 11.2 Hz), 3.77 (dt, 1H, *J* = 4.6, 11.1 Hz), 3.71 (dt, 1H, *J* = 4.6, 11.1 Hz), 3.56 (t, 2H, *J* = 4.6 Hz), 3.39 (s, 3H), 3.24 (m, 1H), 3.14 (dd, 1H, *J* = 4.0, 14.3 Hz), 2.88 (dd, 1H, *J* = 9.7, 14.3 Hz), 2.78 (m, 1H), 2.31 (tq, 1H, *J* = 2.6, 11.2 Hz), 1.98 (m, 1H), 1.84 (m, 1H), 1.74 (dt, 1H, *J* = 2.2, 11.2 Hz), 1.55 (m, 2H), 1.35 (d, 6H, *J* = 7.7 Hz), 1.27 (d, 1H, *J* = 5.3 Hz), 0.80 (d, 3H, *J* = 7.1 Hz) ppm; **^{13}C-NMR** (125 MHz, $CDCl_3$) δ 174.0, 152.7, 137.0, 131.8, 129.2, 128.8, 127.0, 125.8, 94.7, 82.5, 79.0, 71.9, 67.2, 65.3, 63.8, 59.2, 43.9, 39.9, 38.4, 35.6, 31.4, 31.3, 28.3, 26.7, 22.3, 17.4 ppm; **HRMS** ESI *m/z* $[M + Na]^+$ calcd. for $C_{28}H_{39}NO_7Na$ 524.26187 found 524.26081.

Methyl(1*S*,2*S*,4a*R*,5*R*,8a*S*)-8-hydroxy-5-((2-methoxyethoxy)methoxy)-2-methyl-1,2,4a,5,6,7,8,8a-octahydronaphthalene-1-carboxylate (51)

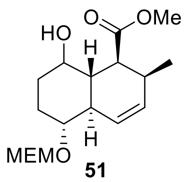

51

Alcohol **50** (554 mg, 1.10 mmol, 1.00 eq.) in dry CH_2Cl_2 (11 mL) was treated with NaOMe (50 wt%, 505 µL, 2.21 mmol, 2.00 eq.) at 0 °C. After stirring for 3 h, sat. aq. NH_4Cl solution was added, and the aqueous phase was extracted wit EtOAc four times. The combined organic phases were washed with sat. aq. $NaHCO_3$ solution as well as brine and dried over Na_2SO_4. Removal of the solvent under reduced pressure and purification by column chromatography (SiO_2, pentane/EtOAc 3:1→2:1→2:3→1:2) gave methylester **51** (325 mg, 90%) in 90% yield as a colourless oil. **R$_f$** = 0.35 (hexanes/EtOAc 1:1); $[\alpha]_D^{20}$+82.8° (c 1.0 in MeOH); **IR** ν_{max}/cm^{-1} 3484 (w), 3024 (w), 2932 (m), 2877 (m), 1732 (s), 1453 (w), 1436 (w), 1366 (w), 1296 (w), 1243 (w), 1199 (m), 1172 (m), 1127 (s), 1107 (s), 1032 (s), 1019 (s), 956 (m), 937 (m), 871 (m), 849 (w), 775 (w), 750 (m), 730 (m), 676 (w); **^{1}H-NMR** (500 MHz, $CDCl_3$) δ 5.89 (d, 1H, *J* = 10.0 Hz), 5.61 (ddd, 1H, *J* = 2.6, 4.4, 10.0 Hz), 4.88 (d, 1H, *J* = 7.1 Hz), 4.74 (d, 1H, *J* = 7.1 Hz), 4.21 (s, 1H), 3.77 (dt, 1H, *J* = 4.6, 11.1 Hz), 3.71 (dt, 1H, *J* = 4.6, 11.1 Hz), 3.69 (s, 3H), 3.56 (t, 2H, *J* = 4.6 Hz), 3.39 (s, 3H), 3.23 (dt, 1H, *J* = 3.9, 10.6 Hz), 2.90 (dd, 1H, *J* = 6.0, 11.6 Hz), 2.59 (m, 1H), 2.34 (tq, 1H, *J* = 2.6, 10.6 Hz), 1.99 (m, 1H), 1.84 (m, 1H), 1.72-1.56

(m, 3H), 1.28 (m, 1H), 0.90 (d, 3H, J = 7.1 Hz) ppm; **^{13}C-NMR** (125 MHz, $CDCl_3$) δ 174.2, 131.5, 126.2, 94.7, 79.2, 71.9, 67.2, 65.4, 59.2, 51.5, 45.1, 39.6, 38.1, 32.2, 31.5, 26.7, 17.6 ppm; **HRMS** ESI *m/z* [M + Na]$^+$ calcd. for $C_{17}H_{28}NO_6Na$ 351.17781 found 351.17722.

Methyl(1*S*,2*S*,4a*R*,5*R*,8a*S*)-5-((2-methoxyethoxy)methoxy)-2-methyl-8-methylene-1,2,4a,5,6,7,8,8a-octahydronaphthalene-1-carboxylate (14)

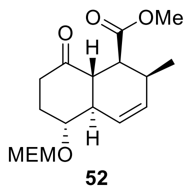

52

To a solution of alcohol **51** (305 mg, 928 µmol, 1.00 eq.) in CH_2Cl_2 *p.a.* (9.3 mL) was added DMP (590 mg, 1.39 mmol, 1.50 eq.) and $NaHCO_3$ (390 mg, 4.64 mmol, 5.00 eq.) at 0 °C. The suspension was allowed to warm to room temperature and stirred for 3 h. After addition of sat. aq. $Na_2S_2O_3$ solution and sat. aq. $NaHCO_3$ solution, the aqueous phase was extracted with EtOAc four times. The combined organic phases were washed with sat. aq. $NaHCO_3$ solution, sat. aq. $Na_2S_2O_3$ solution as well as brine and dried over Na_2SO_4. The crude product was purified by column chromatography (SiO_2, pentane/EtOAc 3:1→3:2→1:1) to give ketone **52** (290 mg, 96%) as a colourless resin in 96% yield. The product wasn´t further purified, but directly used in the next reaction. **R$_f$** = 0.53 (hexanes/EtOAc 1:1); $[\alpha]_D^{20}$+111.1° (c 1.0 in MeOH); **IR** v_{max}/cm^{-1} 3035 (w), 2958 (m), 2928 (m), 2877 (m), 1737 (s), 1720 (s), 1455 (m), 1436 (m), 1375 (w), 1326 (w), 1255 (m), 1197 (m), 1174 (m), 1145 (m), 1097 (s), 1034 (s), 927 (w), 854 (w), 814 (w), 742 (m); **^{1}H-NMR** (500 MHz, $CDCl_3$) δ 5.84 (d, 1H, J = 10.0 Hz), 5.70 (ddd, 1H, J = 2.6, 4.4, 10.0 Hz), 4.89 (d, 1H, J = 7.1 Hz), 4.79 (d, 1H, J = 7.1 Hz), 3.77 (dt, 1H, J = 4.7, 10.9 Hz), 3.71 (m, 1H), 3.69 (s, 3H), 3.57 (t, 2H, J = 4.6 Hz), 3.40 (s, 3H), 2.84 (dd, 1H, J = 6.4, 11.5 Hz), 2.71 (t, 1H, J = 12.0 Hz), 2.66-2.47 (m, 4H), 2.39 (m, 1H), 2.17 (m, 1H), 1.71 (dq, 1H, J = 5.7, 13.4 Hz), 0.86 (d, 3H, J = 7.2 Hz) ppm; **^{13}C-NMR** (125 MHz, $CDCl_3$) δ 209.6, 174.2, 132.7, 124.7, 95.2, 77.7, 71.8, 67.5, 59.2, 51.7, 46.8, 45.1, 42.6, 38.8, 32.9, 31.0, 17.8 ppm; **HRMS** ESI *m/z* [M + Na]$^+$ calcd. for $C_{17}H_{26}O_6Na$ 349.16216 found 349.16156.

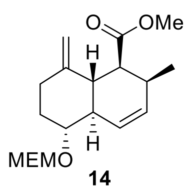

14

Methylphosphoniumbromide (2.14 g, 6.00 mmol, 1.20 eq.) in dry THF (10 mL) was treated with KO*t*Bu (561 mg, 5.00 mmol, 1.00 eq.) at 0 °C. The suspension was stirred for 45 min. A solution of ketone **52** (268 mg, 821 µmol, 1.00 eq.) in dry THF (4.3 mL) was treated with the suspension of ylide (0.5M, 3.28 mL, 1.64 mmol, 2.00 eq.) at 0 °C and stirred for 3 h at room temperature. Sat. aq. NH_4Cl solution was added, and the aqueous phase was extracted with EtOAc four times. The combined organic phases were dried over Na_2SO_4 and the solvents were removed *in vacuo*. Purification of the crude product by column chromatography (SiO_2, pentane/EtOAc 5:1)

delivered decalin **14** (240 mg, 90%) as a colourless liquid in 90% yield. $\mathbf{R_f}$ = 0.74 (hexanes/EtOAc 1:1); $[\boldsymbol{\alpha}]_D^{20}$+101.6° (c 1.0 in MeOH); **IR** ν_{max}/cm^{-1} 2934 (m), 2877 (m), 1742 (s), 1653 (w), 1455 (m), 1436 (m), 1365 (w), 1325 (m), 1300 (w), 1256 (m), 1192 (m), 1132 (s), 1109 (s), 1058 (m), 1032 (s), 931 (m), 892 (m), 852 (m), 818 (w), 775 (w), 745 (m), 730 (m), 670 (w); **^{1}H-NMR** (500 MHz, $CDCl_3$) δ 5.84 (dt, 1H, J = 1.4, 10.0 Hz), 5.68 (ddd, 1H, J = 2.6, 4.6, 10.0 Hz), 4.87 (d, 1H, J = 7.1 Hz), 4.75 (d, 1H, J = 7.1 Hz), 4.73 (s, 1H), 4.39 (s, 1H), 3.76 (dt, 1H, J = 4.7, 11.0 Hz), 3.70 (dt, 1H, J = 4.7, 11.0 Hz), 3.67 (s, 3H), 3.57 (t, 2H, J = 4.6 Hz), 3.40 (dt, 1H, J = 4.6, 10.8 Hz), 3.40 (s, 3H), 2.92 (dd, 1H, J = 6.3, 11.9 Hz), 2.62 (m, 1H), 2.38-2.28 (m, 2H), 2.23-2.13 (m, 2H), 1.87 (tq, 1H, J = 2.2, 10.8 Hz), 1.46-1.35 (m, 1H), 0.89 (d, 3H, J = 7.2 Hz) ppm; **^{13}C-NMR** (125 MHz, $CDCl_3$) δ 174.7, 150.3, 131.7, 125.4, 104.7, 95.0, 79.5, 71.9, 67.2, 59.2, 51.5, 48.5, 45.4, 38.5, 34.9, 34.7, 31.7, 18.3 ppm; **HRMS** ESI m/z [M + H]$^+$ calcd. for $C_{18}H_{29}O_5$ 325.20095 found 325.19994.

(1*S*,2*S*,4a*R*,5*R*,8a*S*)-5-((2-Methoxyethoxy)methoxy)-2-methyl-8-methylene-1,2,4a,5,6,7,8,8a-octahydronaphthalene-1-carbaldehyde (53)

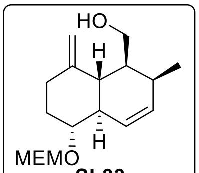

SI-33

Ester **14** (220 mg, 678 µmol, 1.00 eq.) dissolved in dry CH_2Cl_2 (6.8 mL) was treated with DIBAL (2.03 mL, 2.03 mmol, 3.00 eq.) at 0 °C. After stirring at this temperature for 4 h another portion of DIBAL (339 µL, 339 µmol, 0.50 eq.) was added. As soon as TLC showed complete conversion of the starting material, sat. aq. Na,K-tartrate solution was added and the two-phase mixture was stirred vigorously at room temperature for 45 min. The aqueous phase was extracted with EtOAc thrice, combined organic phases were washed with brine and dried over Na_2SO_4. Solvents were removed at the rotary evaporator. Crude product **SI-33** (211 mg, quant.) was used without further purification. $\mathbf{R_f}$ = 0.53 (hexanes/EtOAc 1:1); $[\boldsymbol{\alpha}]_D^{20}$+76.5° (c 0.9 in MeOH); **IR** ν_{max}/cm^{-1} 3424 (w), 3027 (w), 2930 (m), 2875 (m), 1649 (m), 1454 (m), 1394 (w), 1366 (m), 1296 (w), 1242 (w), 1200 (w), 1178 (w), 1155 (w), 1109 (m), 1086 (m), 1052 (s), 1037 (s), 1014 (s), 982 (m), 923 (m), 896 (m), 849 (w), 830 (w), 749 (m), 739 (m), 720 (w), 677 (w); **^{1}H-NMR** (500 MHz, $CDCl_3$) δ 5.80 (d, 1H, J = 10.1 Hz), 5.76 (ddd, 1H, J = 1.9, 4.4, 10.1 Hz), 4.88 (s, 1H), 4.85 (d, 1H, J = 7.1 Hz), 4.76 (s, 1H), 4.74 (d, 1H, J = 7.1 Hz), 4.15 (dt, 1H, J = 4.4, 11.3 Hz), 3.76 (dt, 1H, J = 4.7, 10.9 Hz), 3.70 (dt, 1H, J = 4.7, 10.9 Hz), 3.56 (t, 2H, J = 4.7 Hz), 3.55 (m, 1H), 3.40 (s, 3H), 3.37 (dt, 1H, J = 4.5, 10.7 Hz), 2.50 (m, 1H), 2.31 (m, 2H), 2.21 (m, 1H), 2.05 (dt, 1H, J = 4.6, 13.0 Hz), 1.93 (tq, 1H, J = 1.9, 10.7 Hz), 1.75 (t, 1H, J = 10.7 Hz), 1.43 (m, 1H), 1.20 (t, 1H, J = 5.3 Hz), 0.99 (d, 3H, J = 7.1 Hz) ppm; **^{13}C-NMR**

(125 MHz, $CDCl_3$) δ 150.3, 133.4, 125.2, 106.3, 95.0, 80.1, 71.9, 67.2, 62.1, 59.2, 50.6, 39.2, 38.5, 35.8, 35.6, 30.5, 16.4 ppm; **HRMS** ESI *m/z* $[M + H]^+$ calcd. for $C_{17}H_{29}O_4$ 297.20604 found 297.20509.

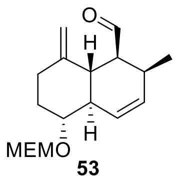

53

A solution of alcohol **SI-33** (180 mg, 607 µmol, 1.00 eq.) in CH_2Cl_2 *p.a.* (6 mL) was treated with $NaHCO_3$ (255 mg, 3.04 mmol, 5.00 eq.) and DMP (386 mg, 911 µmol, 1.50 eq.) at 0 °C. The suspension was stirred at this temperature for 1 h and at room temperature for 2 h. Sat. aq. $NaHCO_3$ solution and sat. aq. $Na_2S_2O_3$ solution were added. The aqueous phase was extracted with EtOAc thrice, the combined organic phases were washed with sat. aq. $NaHCO_3$ solution, sat. $Na_2S_2O_3$ aq. solution as well as brine and dried over Na_2SO_4. The crude product was purified by column chromatography (SiO_2, pentane/EtOAc 6:1→5:1) to give aldehyde **53** (163 mg, 91%) as a colourless liquid. **R_f** = 0.71 (hexanes/EtOAc 1:1); $[\alpha]_D^{20}$+43.0° (c 0.4 in MeOH); **IR** ν_{max}/cm^{-1} 2929 (m), 2878 (m), 1720 (m), 1652 (w), 1455 (m), 1366 (w), 1261 (m), 1199 (w), 1166 (w), 1102 (s), 1094 (s), 1032 (s), 895 (m), 849 (w), 803 (m), 741 (m); **^{1}H-NMR** (500 MHz, $CDCl_3$) δ 9.64 (d, 1H, J = 4.3 Hz), 5.87 (dt, 1H, J = 1.5, 10.1 Hz), 5.67 (ddd, 1H, J = 2.6, 4.5, 10.1 Hz), 4.88 (d, 1H, J = 7.1 Hz), 4.82 (s, 1H), 4.76 (d, 1H, J = 7.1 Hz), 4.38 (s, 1H), 3.77 (dt, 1H, J = 4.8, 10.8 Hz), 3.71 (dt, 1H, J = 4.8, 10.8 Hz), 3.57 (t, 2H, J = 4.8 Hz), 3.44 (dt, 1H, J = 4.6, 10.7 Hz), 3.40 (s, 3H), 2.74-2.62 (m, 2H), 2.42-2.29 (m, 3H), 2.18 (dt, 1H, J = 4.6, 13.5 Hz), 1.92 (tq, 1H, J = 2.1, 10.7 Hz), 1.44 (m, 1H), 1.01 (d, 3H, J = 6.9 Hz) ppm; **^{13}C-NMR** (125 MHz, $CDCl_3$) δ 207.5, 148.8, 132.0, 125.8, 107.5, 94.9, 79.3, 71.9, 67.3, 59.2, 50.3, 48.5, 37.4, 34.74, 34.67, 32.4, 16.9 ppm; **HRMS** ESI *m/z* $[M + H]^+$ calcd. for $C_{17}H_{27}O_4$ 295.19039 found 295.18976.

(1*S*,2*S*,4a*R*,5*R*,8a*S*)-5-((2-Methoxyethoxy)methoxy)-2-methyl-8-methylene-1,2,4a,5,6,7,8,8a-octahydronaphthalene-1-carbonyl cyanide (54)

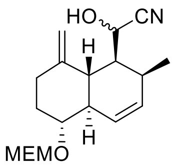

SI-34

Aldehyde **53** (86.2 mg, 292 µmol, 1.00 eq.) in dry CH_2Cl_2 (3 mL) was treated with TMSCN (110 µL, 876 µmol, 3.00 eq.) and dry NEt_3 (121 µL, 876 µmol, 3.00 eq.) at 0 °C. The solution was stirred at this temperature for 20 min and at room temperature for 4 h. The volatiles were removed at the rotary evaporator. Crude product was dissolved in EtOH *p.a.* and NH_4F (48.7 mg, 1.31 mmol, 4.50 eq.) was added at 0 °C. After 2 h of stirring TLC showed complete conversion of the starting material. H_2O was added and the aqueous phase was extracted with EtOAc thrice. Combined organic phases were washed with brine and dried over Na_2SO_4. The solvents were

removed under reduced pressure and the oily, colourless product **SI-34** (92.3 mg, quant., *dr* 1.1:1) was used without further purification. **R**$_f$ = 0.64 (hexanes/EtOAc 1:1); $[\alpha]_D^{20}$+61.1° (c 0.5 in MeOH); **IR** ν_{max}/cm^{-1} 3385 (m), 3076 (w), 3030 (w), 2933 (m), 2881 (m), 1651 (m), 1455 (m), 1395 (w), 1366 (w), 1296 (w), 1244 (w), 1170 (m), 1098 (s), 1036 (s), 894 (m), 848 (m), 754 (m), 737 (w), 677 (w); major diastereomer **^{1}H-NMR** (500 MHz, $CDCl_3$) δ 5.83 (d, 1H, J = 10.0 Hz), 5.74 (ddd, 1H, J = 2.5, 5.1, 10.0 Hz), 5.30 (m, 1H), 4.93 (s, 1H), 4.85 (d, 1H, J = 7.1 Hz), 4.74 (d, 1H, J = 7.1 Hz), 4.68 (s, 1H), 3.75 (dt, 1H, J = 4.7, 10.9 Hz), 3.69 (dt, 1H, J = 4.7, 10.9 Hz), 3.56 (t, 2H, J = 4.7 Hz), 3.43 (m, 1H), 3.39 (s, 3H), 2.73 (m, 1H), 2.55 (m, 1H), 2.40-2.25 (m, 3H), 2.22-1.95 (m, 3H), 1.44 (m, 1H), 1.19 (d, 3H, J = 7.1 Hz) ppm; minor diastereomer **^{1}H-NMR** (500 MHz, $CDCl_3$) δ 5.81 (d, 1H, J = 10.0 Hz), 5.72 (ddd, 1H, J = 2.5, 5.1, 10.0 Hz), 5.30 (m, 1H), 4.94 (s, 1H), 4.85 (d, 1H, J = 7.1 Hz), 4.74 (d, 1H, J = 7.1 Hz), 4.65 (s, 1H), 3.75 (dt, 1H, J = 4.7, 10.9 Hz), 3.69 (dt, 1H, J = 4.7, 10.9 Hz), 3.56 (t, 2H, J = 4.7 Hz), 3.43 (m, 1H), 3.40 (s, 3H), 2.63 (m, 1H), 2.35 (m, 3H), 2.22-1.95 (m, 4H), 1.44 (m, 1H), 1.21 (d, 3H, J = 7.1 Hz) ppm; major diastereomer **^{13}C-NMR** (125 MHz, $CDCl_3$) δ 150.7, 132.7, 125.4, 119.4, 106.2, 95.0, 79.8, 71.9, 67.2, 61.1, 59.2, 50.5, 40.0, 38.0, 35.8, 35.7, 31.7, 18.2 ppm; minor diastereomer **^{13}C-NMR** (125 MHz, $CDCl_3$) δ 150.1, 132.7, 125.4, 119.4, 105.7, 95.0, 79.9, 71.9, 67.2, 60.9, 59.2, 50.7, 40.0, 39.5, 35.65, 35.61, 29.3, 17.1 ppm; **HRMS** ESI *m/z* $[M + H]^+$ calcd. for $C_{18}H_{28}NO_4$ 322.20128 found 322.20044.

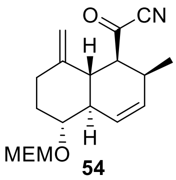

54

A solution of cyanohydrin **SI-34** (87.0 mg, 271 µmol, 1.00 eq.) in dry CH_2Cl_2 (2.7 mL) was treated with DMP (138 mg, 325 µmol, 1.20 eq.) at 0 °C. The suspension was stirred for 1.5 h at this temperature, before it was filtered off over celite®. The solvent was removed *in vacuo* and the crude product was purified by column chromatography (SiO_2, pentane/EtOAc 5:1) to give the acylcyanide **54** (71.3 mg, 82%) as a colourless liquid. **R**$_f$ = 0.77 (hexanes/EtOAc 1:1); $[\alpha]_D^{20}$+111.3° (c 1.0 in MeOH); **IR** ν_{max}/cm^{-1} 2934 (m), 2879 (m), 2217 (w), 1708 (m), 1653 (w), 1455 (w), 1177 (m), 1096 (s), 1054 (s), 1026 (s), 897 (m), 744 (m); **^{1}H-NMR** (500 MHz, $CDCl_3$) δ 5.91 (dt, 1H, J = 1.5, 10.1 Hz), 5.70 (ddd, 1H, J = 2.6, 4.6, 10.1 Hz), 4.87 (s, 1H), 4.87 (d, 1H, J = 7.1 Hz), 4.75 (d, 1H, J = 7.1 Hz), 4.29 (s, 1H), 3.76 (dt, 1H, J = 4.8, 10.9 Hz), 3.70 (dt, 1H, J = 4.8, 10.9 Hz), 3.56 (t, 2H, J = 4.8 Hz), 3.45 (dt, 1H, J = 4.6, 10.7 Hz), 3.39 (s, 3H), 3.18 (dd, 1H, J = 6.3, 12.1 Hz), 2.82 (m, 1H), 2.45-2.33 (m, 3H), 2.22 (dt, 1H, J = 4.7, 13.5), 1.94 (tq, 1H, J = 2.1, 10.7 Hz), 1.43 (m, 1H), 1.04 (d, 3H, J = 7.1 Hz) ppm; **^{13}C-NMR** (125 MHz, $CDCl_3$) δ 180.6, 148.8, 130.8, 125.9, 113.6, 107.2, 94.9, 79.0, 71.8, 67.4, 59.2, 52.7, 48.4, 37.8, 34.7, 34.3, 32.0, 17.4 ppm; **HRMS** ESI *m/z* $[M + Na]^+$ calcd. for $C_{18}H_{25}NO_4Na$ 342.16758 found 342.16726.

(1*S*,2*S*,4a*R*,5*R*,8a*S*)-5-Hydroxy-2-methyl-8-methylene-1,2,4a,5,6,7,8,8a-octahydronaphthalene-1-carbonyl cyanide (5)

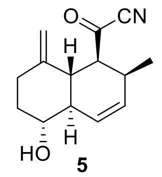

5

MEM-ether **54** (25.1 mg, 78.3 µmol, 1.00 eq.) in MeCN *p.a.* (1.5 mL) and H_2O (0.1 mL) was treated with $LiBF_4$ (183 mg, 1.96 mmol, 25.0 eq.) at room temperature. The mixture was stirred at 55 °C for 4.5 h. H_2O was added at 0 °C and the aqueous phase was extracted with EtOAc thrice. The combined organic phases were washed with brine and dried over Na_2SO_4. Purification of the crude product by column chromatography (SiO_2, pentane/EtOAc 4:1→3:1) gave product **5** (18.2 mg, 99%) as a colourless resin. **R$_f$** = 0.30 (hexanes/EtOAc 3:1); $[\boldsymbol{\alpha}]_{D}^{20}$+157.3° (c 1.0 in $CHCl_3$); **IR** ν_{max}/cm^{-1} 3375 (m), 3081 (w), 3032 (w), 2965 (w), 2939 (m), 2877 (m), 2217 (m), 1708 (s), 1652 (m), 1454 (m), 1377 (w), 1328 (w), 1260 (w), 1163 (m), 1062 (s), 1029 (s), 999 (w), 896 (m), 868 (w), 838 (w), 741 (s), 674 (m); **^{1}H-NMR** (500 MHz, $CDCl_3$) δ 6.00 (dt, 1H, J = 1.4, 10.1 Hz), 5.73 (ddd, 1H, J = 2.6, 4.6, 10.1 Hz), 4.89 (s, 1H), 4.30 (s, 1H), 3.52 (dt, 1H, J = 4.6, 10.5 Hz), 3.18 (dd, 1H, J = 6.3, 12.2 Hz), 2.83 (m, 1H), 2.43 (m, 1H), 2.36 (t, 1H, J = 11.6 Hz), 2.30-2.22 (m, 2H), 1.84 (tq, 1H, J = 2.2, 10.6 Hz), 1.65 (br. s, 1H), 1.52-1.43 (m, 1H), 1.05 (d, 3H, J = 7.2 Hz) ppm; **^{13}C-NMR** (125 MHz, $CDCl_3$) δ 180.7, 148.8, 130.9, 125.8, 113.6, 107.4, 73.3, 52.6, 49.9, 37.8, 37.5, 34.5, 32.1, 17.4 ppm.

2.6 Failed routes to the decalin

One promising and short route started from dibromide **SI-35** which was elongated by a tin mediated reaction to alcohol **SI-36**. After TBS-protection and formylation an HWE- or Wittig olefination with an auxiliary based phosphonate **SI-39** or ylide **SI-40** was not possible. The following steps should have been performed analogously to the actual route.

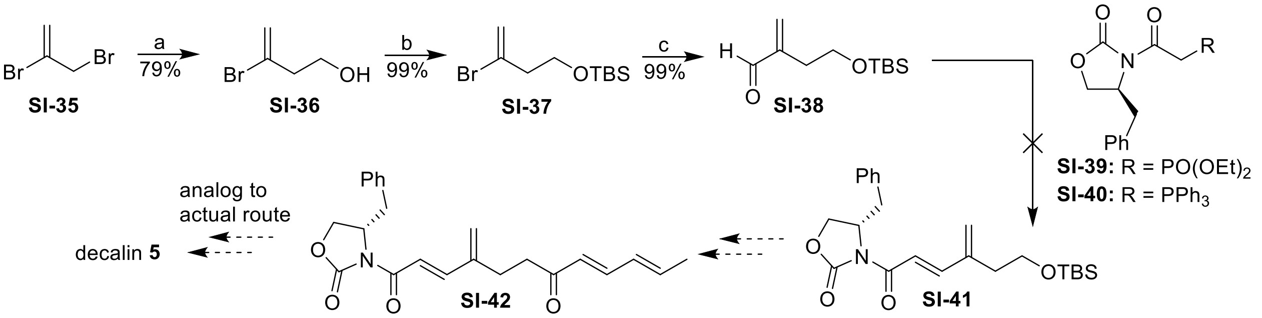

Scheme S8. Attempt to synthesise triene **SI-42** starting from vinylbromide **SI-35**.
Reagents and conditions: a) Sn, CH_2O, cat. HBr, Et_2O/H_2O, rt, 19 h; b) TBSCl, imidazole, DMAP, CH_2Cl_2, rt, 21 h, c) 1. *t*BuLi, Et_2O, −78 °C, 30 min, 2. DMF, 3.5 h.

After failure of the olefination of an α,β-unsaturated aldehyde we had the plan to introduce the terminal alkene after the olefination reaction. Starting with malic acid (**SI-43**) it was first chemoselectively esterified and TBS-protected (→ **SI-45**). The carboxyl group was reduced to alcohol **SI-46** which was iodinated in an Appel-reaction (→**SI-47**). The following Fukuyama coupling was not successful due to low formation of the zinc organyl.

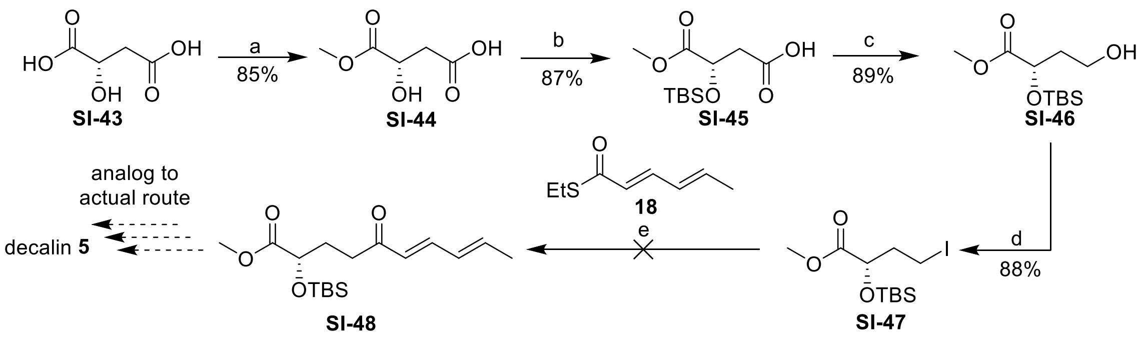

Scheme S9. Tested route to α-hydroxylated ester **SI-48**.
Reagents and conditions: a) 1. $(TFA)_2$, rt, 3 h, 2. MeOH, rt, 22.5 h, b) 1. TBSCl, imidazole, DMAP, CH_2Cl_2, rt, 23 h, 2. K_2CO_3, H_2O, MeOH, rt, 2.5 h, c) 1. EtOCOCl, NMM, THF, −10 °C, 1.2 h, 2. $NaBH_4$, H_2O, 1 h; d) PPh_3, imidazole, I_2, THF, 0 °C, 1 h.

2.7 Synthesis of reagents for the decalin fragment

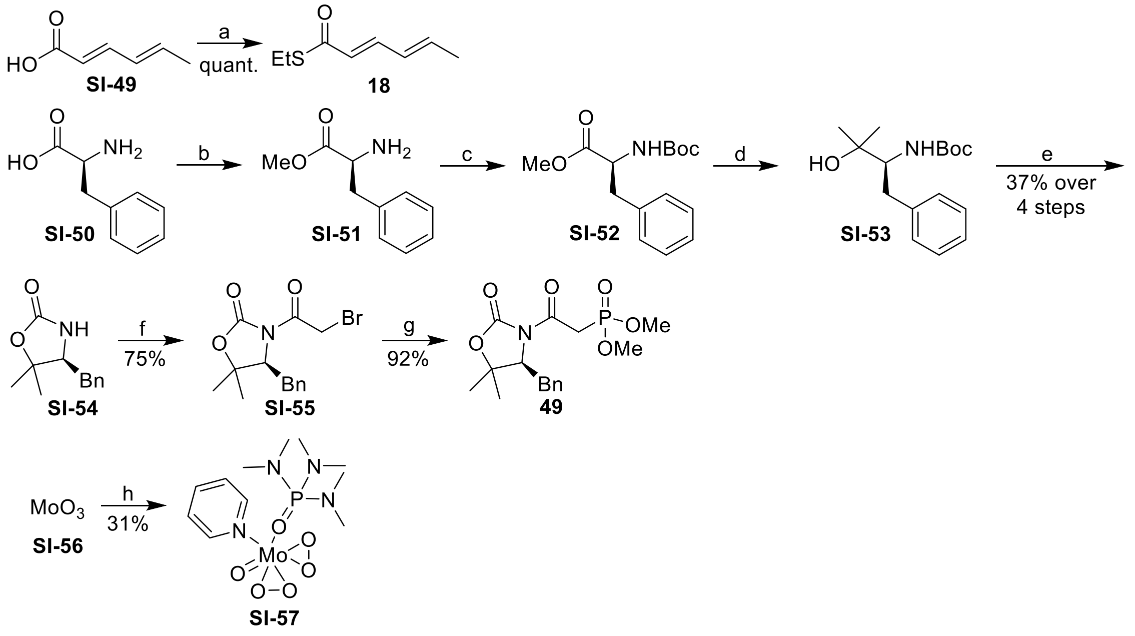

Scheme S10. Synthesis of thioester **18**, phosphonate **49** and molybdenum reagent **SI-57** needed for formation of decalin **5**.
Reagents and conditions: a) DCC, DMAP, EtSH, CH_2Cl_2, 0 °C→rt, 21 h; b) $SOCl_2$, MeOH, 0 °C→reflux, 20 h; c) Boc_2O, NEt_3, imidazole, CH_2Cl_2, 17 h; d) MeMgBr, THF, 0 °C→rt, 2 d; e) KO*t*Bu, THF, 0 °C, 30 min; f) 1. *n*BuLi, THF, −80 °C, 10 min, 2. Bromoacetylbromide, −80 °C→rt, 13.5 h; g) $P(OMe)_3$, 20.5 h, rt→60 °C; h) 1. H_2O_2, 40 °C, 4.25 h, 2. HMPA, rt, 5 min, 3. Pyridine, THF, rt, 15 min.

(*S*)-Ethyl (2*E*,4*E*)-hexa-2,4-dienethioate (18)

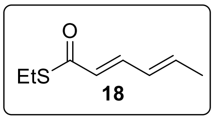

Sorbic acid (**SI-49**) (5.00 g, 44.6mmol, 1.00 eq.) was dissolved in dry CH_2Cl_2 (203 mL). DCC (9.66 g, 46.8 mmol, 1.05 eq.), DMAP (545 mg, 4.46 mmol, 0.10 eq.) were added at room temperature. At 0 °C EtSH (4.29 mL, 58.0 mmol, 1.30 eq.) was dripped to the mixture and it was stirred for 21 h at room temperature. The reaction mixture was filtered off over celite® and the solvents were partially removed. The organic phase was washed with sat. aq. $NaHCO_3$ solution and H_2O. The combined aqueous phases were reextracted with CH_2Cl_2 once and the organic phases were washed with brine. It was dried over Na_2SO_4. The solvent was removed *in vacuo* and the crude product was purified by column chromatography (SiO_2, CH_2Cl_2/MeOH 30:1→20:1) to give thioester **18** (6.97 g, quant.) as a light-yellow liquid. $\mathbf{R_f}$ = 0.92 (CH_2Cl_2/MeOH 25:1); **^{1}H-NMR** (500 MHz, $CDCl_3$) δ 7.17 (dd, 1H, J = 10.2, 15.2 Hz), 6.25-6.11 (m, 2H), 6.06 (d, 1H, J =

15.2 Hz), 2.95 (q, 2H, J = 7.4 Hz), 1.86 (d, 3H, J = 6.1 Hz), 1.28 (t, 3H, J = 7.4 Hz) ppm; **^{13}C-NMR** (125 MHz, $CDCl_3$) δ 190.3, 141.0, 140.8, 129.8, 126.3, 23.3, 19.0, 15.0 ppm.

Spectroscopic data corresponded to those reported in the literature.[7]

(*S*)-4-Benzyl-5,5-dimethyloxazolidin-2-one (SI-54)

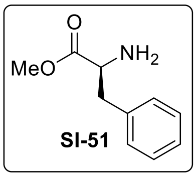

L-Phenylalanin (**SI-50**, 19.8 g, 120 mmol, 1.00 eq.) in MeOH *p.a.* (300 mL) was treated with $SOCl_2$ (26.1 mL, 360 mmol, 3.00 eq.) at 0 °C. The mixture was stirred at reflux for 20 h. The volatiles were removed under reduced pressure. The crude product was dissolved in MeOH *p.a.* and solvents were removed. This procedure was carried out multiple times. Methylesterhydrochlorid **SI-51** (25.7 g, quant.) was isolated as a colourless solid and used without further purification.

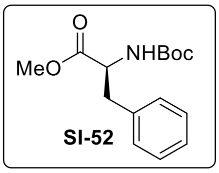

Methylester **SI-51** (25.7 g, 119 mmol, 1.00 eq.) in dry CH_2Cl_2 (300 mL) was treated with dry NEt_3 (18.3 mL) and Boc_2O (27.3 g, 125 mmol, 1.05 eq.) in dry CH_2Cl_2 (100 mL) at 0 °C. The suspension was stirred at this temperature for 20 min, dry NEt_3 (4.15 mL, 29.8 mmol, 0.25 eq.) was added again and stirring was continued at room temperature for 16 h. Imidazole (810 mg, 11.9 mmol, 0.10 eq.) was added and stirring was continued for 30 min. The mixture was poured into citric acid solution (1M). Organic phase was separated and washed with citric acid solution (1M) twice, with 1 vol% HCl twice and with brine once. They were dried over Na_2SO_4, and solvents were removed at the rotary evaporator. The Boc-protected phenylalanineester **SI-52** (33.1 g, 92%) was isolated as a clear brownish resin and was used without further purification.

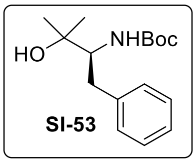

Ester **SI-52** (27.9 g, 100 mmol, 1.00 eq.) in dry THF (200 mL) was treated with MeMgBr (3M in Et_2O, 133 mL, 400 mmol) at 0 °C over 45 min. Solution was stirred at room temperature for 2 d. MeOH and H_2O was added, and the suspension was filtered off over celite®. The solvent was removed under reduced pressure and the crude product was suspended in Et_2O, filtered off over celite® and the solvent was again removed at the rotary evaporator. This procedure was repeated once. Alcohol **SI-53** (25.4 g, 91%) was isolated as a pale brown resin.

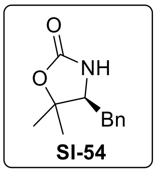

Alcohol **SI-53** (25.4 g, 90.9 mmol, 1.00 eq.) in dry THF (364 mL) was treated with KO*t*Bu (12.2 g, 109 mmol, 1.20 eq.) at 0 °C. After stirring for 30 min, sat. aq. NH_4Cl solution and EtOAc were added, and the aqueous phase was extracted with EtOAc twice. Combined organic phases were washed with brine and dried

over Na_2SO_4. After removal of the volatiles under reduced pressure, the crude product was recrystallised with pentane/Et_2O twice. Oxazolidinone **SI-54** (8.29 g, 44%) was obtained as colourless needles. $\mathbf{R_f}$ = 0.26 (hexanes/EtOAc 2:1); **mp** 66.5 °C; Lit.[8] **mp** 66-67 °C; **IR** ν_{max}/cm^{-1} 3263 (m), 3030 (w), 2980 (m), 2933 (w), 1739 (s), 1604 (w), 1496 (m), 1455 (m), 1374 (m), 1298 (m), 1271 (m), 1241 (w), 1218 (w), 1189 (w), 1143 (w), 1085 (m), 995 (m), 967 (w), 940 (w), 914 (w), 884 (w), 771 (m), 744 (m), 700 (s); **^{1}H-NMR** (500 MHz, $CDCl_3$) δ 7.34 (m, 2H), 7.27 (m, 1H), 7.18 (m, 2H), 4.87 (br. s , 1H), 3.69 (ddd, 1H, J = 0.6, 3.7, 10.8 Hz), 2.84 (dd, 1H, J = 3.7, 13.3 Hz), 2.67 (dd, 1H, J = 10.8, 13.3 Hz), 1.48 (s, 3H), 1.46 (s, 3H) ppm; **^{13}C-NMR** (125 MHz, $CDCl_3$) δ 158.0, 137.0, 129.2, 129.0, 127.4, 127.1, 83.3, 63.2, 37.2, 27.7, 22.1 ppm.

Spectroscopic data corresponded to those reported in the literature.[8]

(*S*)-4-Benzyl-3-(2-bromoacetyl)-5,5-dimethyloxazolidin-2-one (SI-55)

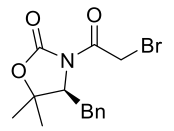

SI-55

A solution of oxazolidinone **SI-54** (6.00 g, 29.2 mmol, 1.00 eq.) in dry THF (73 mL) was treated with *n*BuLi (12.3 mL, 30.7 mmol, 1.05 eq.) at −80 °C. After 10 min, bromoacetylbromide (2.67 mL, 32.7 mmol, 1.12 eq.) was added at −80 °C and stirring was continued for 13.5 h at room temperature. Sat. aq. NH_4Cl solution and EtOAc were added and the aqueous phase was extracted with EtOAc thrice. Combined organic phases were washed with sat. aq. $NaHCO_3$ solution as well as brine and dried over Na_2SO_4. Crude product was purified by column chromatography (SiO_2, pentane/EtOAc 5:1) to yield bromide **SI-55** (7.14 g, 75%) as a light-yellow oil. $\mathbf{R_f}$ = 0.76 (hexanes/EtOAc 4:1); $[\boldsymbol{\alpha}]_D^{20}$−26.3° (c 1.0 in $CHCl_3$); **IR** ν_{max}/cm^{-1} 3060 (w), 3028 (w), 2983 (w), 2940 (w), 1773 (s), 1698 (s), 1605 (w), 1497 (w), 1455 (w), 1415 (w), 1393 (m), 1358 (s), 1327 (m), 1276 (s), 1234 (m), 1207 (m), 1184 (m), 1161 (m), 1142 (m), 1094 (s), 1024 (w), 962 (m), 920 (w), 902 (w), 849 (w), 761 (m), 731 (m), 700 (m), 653 (m); **^{1}H-NMR** (500 MHz, $CDCl_3$) δ 7.35-7.21 (m, 5H), 4.58 (d, 1H, J = 12.4 Hz), 4.51 (dd, 1H, J = 3.8, 9.7 Hz), 4.44 (d, 1H, J = 12.4 Hz), 3.19 (dd, 1H, J = 3.8, 14.6 Hz), 2.90 (dd, 1H, J = 9.7, 14.6 Hz), 1.42 (s, 3H), 1.41 (s, 3H) ppm; **^{13}C-NMR** (125 MHz, $CDCl_3$) δ 166.4, 152.3, 136.6, 129.2, 128.9, 127.1, 83.4, 64.2, 35.1, 28.8, 28.5, 22.4 ppm; **HRMS** ESI *m/z* [M + H]$^+$ calcd. for $C_{14}H_{17}NO_3Br$ 326.03863 found 326.03800.

Spectroscopic data corresponded to those reported in the literature.[9]

Dimethyl-(*S*)-(2-(4-benzyl-5,5-dimethyl-2-oxooxazolidin-3-yl)-2-oxoethyl)phosphonate (49)

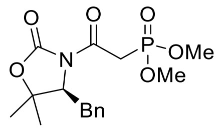

49

Bromide **SI-55** (5.17 g, 15.8 mmol, 1.00 eq.) was treated with $P(OMe)_3$ (9.36 mL, 79.2 mmol, 5.00 eq.) at room temperature. The mixture was stirred for 17 h at room temperature and for 3.5 h at 60 °C. The volatiles were removed under reduced pressure and the crude product was purified by column chromatography (SiO_2, EtOAc) to give phosphonate **49** (5.15 g, 92%) as a colourless resin. $\mathbf{R_f}$ = 0.59 (EtOAc); $[\alpha]_D^{20}$−12.3° (c 1.0 in $CHCl_3$); **IR** ν_{max}/cm^{-1} 3011 (w), 2957 (w), 2854 (w), 1771 (s), 1695 (s), 1605 (w), 1498 (w), 1456 (w), 1396 (m), 1357 (s), 1322 (m), 1265 (s), 1211 (m), 1185 (m), 1160 (m), 1094 (m), 1056 (m), 1020 (s), 964 (m), 926 (w), 901 (w), 882 (m), 846 (m), 806 (m), 764 (m), 731 (s), 700 (m), 677 (m); ^{1}**H-NMR** (500 MHz, $CDCl_3$) δ 7.33-7.21 (m, 5H), 4.53 (dd, 1H, J = 3.7, 9.8 Hz), 4.06 (dd, 1H, J = 14.1, 22.0 Hz), 3.82 (d, 3H, J = 4.9 Hz), 3.80 (d, 3H, J = 4.9 Hz), 3.56 (dd, 1H, J = 14.1, 22.2 Hz), 3.18 (dd, 1H, J = 3.7, 14.6 Hz), 2.89 (dd, 1H, J = 9.8, 14.6 Hz), 1.40 (s, 3H), 1.37 (s, 3H) ppm; ^{13}**C-NMR** (125 MHz, $CDCl_3$) δ 165.0 (d, J = 7.0 Hz), 152.8, 136.9, 129.2, 128.8, 127.0, 82.8, 64.1, 53.4 (d, J = 5.8 Hz), 53.3 (d, J = 5.8 Hz), 35.3, 34.4, 33.3, 28.5, 22.4 ppm; **HRMS** ESI *m/z* $[M + H]^+$ calcd. for $C_{17}H_{22}NO_6P$ 356.12575 found 356.12491.

Oxodiperoxymolybdenum(pyridine) (hexamethylphosphoric triamide) (SI-57)

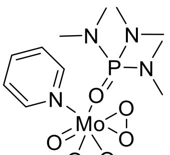

SI-57

MoO_3 (**SI-56**, 30.0 g, 208 mmol, 1.00 eq.) was dissolved in H_2O_2 (30 wt%, 150 mL) and stirred at 40 °C. Temperature was strictly kept at max. 40 °C, while stirring for 4.25 h. The suspension was filtered off over celite® and the mother liquor was treated with HMPA (36.2 mL, 208 mmol, 1.00 eq.) and stirred vigorously for 5 min. It was again filtered off and the solid was recrystallized in MeOH. The solid (27.6 g, 77.4 mmol, 1.00 eq.) was dried in the desiccator and dissolved in dry THF (115 mL). Pyridine (6.26 mL, 77.4 mmol, 1.00 eq.) was added at room temperature and the mixture was stirred for 15 min. The solid was filtered off, washed with dry THF as well as dry Et_2O and dried in a desiccator filled with P_2O_5. The Vedejs-reagent (**SI-57**, 27.8 g, 31%) was isolated as yellow crystals.

There is no convenient analytical method for characterization of this compound.[10]

2.8 Synthesis of glycosides **62a** and **62b**

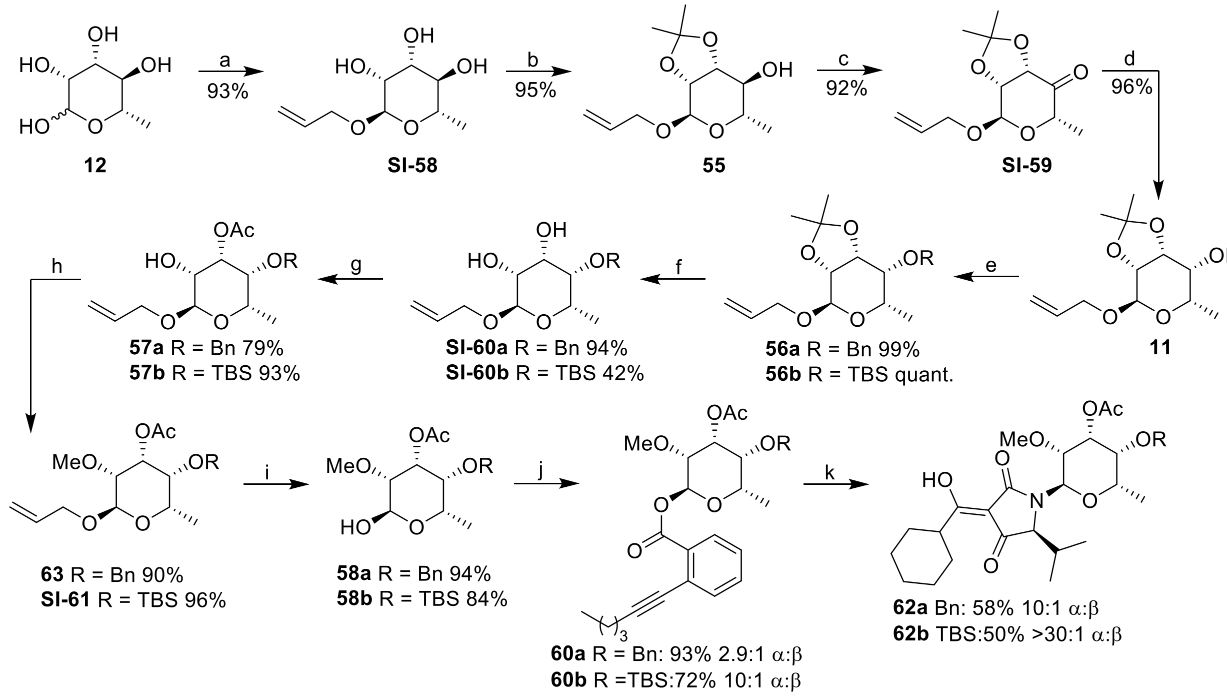

Scheme S11. Synthesis of glycosides **62a/b**.
Reagents and conditions: a) AcCl, allylOH, 0 °C→55 °C, 24 h; b) $CuSO_4$, AcMe, rt, 17 h; c) 1. $(ClCO)_2$, DMSO, −78 °C, 40 min, 2. **55**, 50 min, 3. DIPEA, −78 °C→rt, 16 h; d) $NaBH_4$, 0 °C, 1.5 h; e) 1. **56a**: NaH, imidazole, DMF, 0 °C, 35 min, 2. BnBr, TBAI, rt, 17 h; **56b**: TBSOTf, pyridine, CH_2Cl_2, 0 °C, 5 h; f) **SI-60a**: AcOH, H_2O, reflux, 1.5 h; **SI-60b**: HCOOH, EtOH, rt, 2.5 h; g) **57a**: 1. Bu_2SnO, toluene, reflux, 4 h, 2. AcCl, 0 °C, 30 min; **57b**: 1. Bu_2SnO, toluene, reflux, 3 h, 2. AcCl, rt, 1 h; h) **63**: $TMSCHN_2$, HBF_4, CH_2Cl_2, 0 °C, 5 h; **SI-61** MeO_3BF_4, proton sponge, CH_2Cl_2, 0 °C→40 °C, 21 h; i) **58a**: $Pd(PPh_3)_4$, AcOH, rt, 17 h; **58b**: 1. DABCO, Wilkinson´s catalyst, EtOH, Δ, 15 h, 2. I_2, phosphate buffer pH=7/H_2O/EtOAc, rt, 10 min; j) **60a/b**: acid **59**, DCC, DMAP, CH_2Cl_2, rt, 3-3.5 h; k) **62a/b**: tetramic acid **61**, $AuPPh_3NTf_2$, rt→40 °C, 17-20 h.

(3*R*,4*R*,5*R*,6*S*)-2-(Allyloxy)-6-methyltetrahydro-2*H*-pyran-3,4,5-triol (SI-58)

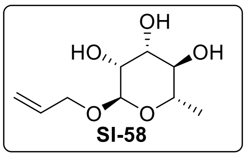

L-Rhamnose (**12**; 10.0 g, 54.9 mmol, 1.00 eq.) was added to a solution of AcCl (10.1 mL, 141 mmol, 1.10 eq.) and allylic alcohol (100 mL) at 0 °C. The mixture was stirred at 55 °C for 24 h. The reaction was quenched with $NaHCO_3$ and the solid was filtered off. The volatiles were removed in vacuo, toluene was added, and the solvent was concentrated under reduced pressure. This procedure was repeated twice. The crude product was purified by column chromatography (SiO_2, CH_2Cl_2/MeOH 10:1→9:1→8:1) to yield the allylated carbohydrate **SI-58** (10.3 g, 93%, α:β 9:1) as a colourless resin. **R$_f$** = 0.74 (CH_2Cl_2/MeOH 4:1); $[\alpha]_D^{20}$ −85.6° (c 1.0, $CHCl_3$); **IR** ν_{max}/cm^{-1} 3376 (s), 2978 (m), 2919 (m), 1451 (w), 1423 (w), 1384 (w), 1265 (w), 1130 (m), 1050 (s), 985 (m), 927 (w), 810 (w); α-anomer: **^{1}H-NMR** (500 MHz, $CDCl_3$) δ 5.89 (dddd, 1H, J = 5.1, 6.0, 10.7, 16.9 Hz), 5.29 (dq, 1H, J = 1.5, 16.9 Hz), 5.20 (dq, 1H, J = 1.5, 10.7 Hz),

4.83 (d, 1H, J = 1.0 Hz), 4.18 (ddt, 1H, J = 1.3, 5.1, 13.0 Hz), 3.99 (ddt, 1H, J = 1.3, 6.0, 13.0 Hz), 3.96 (m, 1H), 3.79 (m, 1H), 3.69 (m, 1H), 3.49 (d, 1H, J = 5.5 Hz), 3.46 (dt, 1H, J = 3.5, 9.4 Hz), 3.04-2.86 (br. s, 1H), 2.78-2.56 (br. s, 2H), 1.32 (d, 3H, J = 6.3 Hz) ppm; β-anomer: **^{1}H-NMR** (500 MHz, $CDCl_3$) δ 5.93 (m, 1H), 5.30 (m, 1H), 5.23 (m, 1H), 4.51 (s, 1H), 4.40 (ddt, 1H, J = 1.3, 5.2, 12.8 Hz), 4.13 (ddt, 1H, J = 1.3, 6.6, 12.8 Hz), 3.99 (m, 2H), 3.79 (m, 1H), 3.69 (m, 1H), 3.27 (m, 1H), 2.93 (br. s, 1H), 1.64 (br. s, 1H), 1.37 (d, 3H, J = 6.2 Hz), 0.99 (m, 1H) ppm. α-anomer: **^{13}C-NMR** (125 MHz, $CDCl_3$) δ 133.8, 117.7, 99.0, 73.1, 71.9, 71.1, 68.3, 68.1, 17.7 ppm; β-anomer: **^{13}C-NMR** (125 MHz, $CDCl_3$) δ 133.6, 118.6, 98.6, 74.2, 72.9, 72.2, 71.2, 70.1, 17.7 ppm.

Spectroscopic data corresponded to those reported in the literature.[11]

(3a*R*,6*S*,7*S*,7a*R*)-4-(Allyloxy)-2,2,6-trimethyltetrahydro-4*H*-[1,3]dioxolo[4,5-c]pyran-7-ol (55)

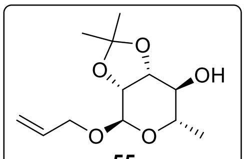

55

A solution of glycoside **SI-58** (7.74 g, 37.9 mmol, 1.00 eq.) in acetone (1.60 L) was treated with $CuSO_4$ (96.8 g, 606 mmol, 16.0 eq.) and stirred for 17 h at room temperature. The solid was removed by filtration over celite®. Removing of the solvent under reduced pressure gave the product **55** (8.68 g, 94%, α:β 16:1) as a colourless resin. **R_f** = 0.75 (CH_2Cl_2/MeOH 9:1); $[\alpha]_D^{20}$ −26.7° (c 1.0 in $CHCl_3$); **IR** ν_{max}/cm^{-1} 3470 (m), 2985 (m), 2937 (m), 2905 (m), 1456 (w), 1383 (m), 1244 (m), 1220 (m), 1141 (m), 1077 (s), 1053 (s), 1023 (s), 997 (m), 922 (w), 860 (m), 818 (w); α-Anomer: **^{1}H-NMR** (500 MHz, $CDCl_3$) δ 5.91 (dddd, 1H, J = 5.3, 6.2, 10.3, 17.0 Hz), 5.31 (dq, 1H, J = 1.4, 17.0 Hz), 5.22 (dq, 1H, J = 1.4, 10.3 Hz), 5.01 (s, 1H), 4.20 (ddt, 1H, J = 1.4, 2.8, 5.3 Hz), 4.17 (d, 1H, J = 5.8 Hz), 4.10 (dd, 1H, J = 5.8, 7.1 Hz), 4.01 (ddt, 1H, J = 1.4, 6.2, 12.8 Hz), 3.70 (dq, 1H, J = 6.3, 9.1 Hz), 3.42 (ddd, 1H, J = 4.6, 7.1, 9.1 Hz), 2.19 (m, 1H), 1.53 (s, 3H), 1.36 (s, 3H), 1.30 (d, 3H, J = 6.3 Hz) ppm; β-Anomer: **^{1}H-NMR** (500 MHz, $CDCl_3$) δ 5.96 (m, 1H), 5.31 (m, 1H), 5.23 (m, 1H), 4.78 (d, 1H, J = 2.2 Hz), 4.43 (ddt, 1H, J = 1.5, 4.9, 13.0 Hz), 4.25 (dd, 1H, J = 2.2, 5.7 Hz), 4.19 (m, 1H), 4.10 (m, 1H), 3.54 (m, 1H), 3.30 (m, 1H), 2.11 (m, 1H), 1.57 (s, 3H), 1.39 (s, 3H), 1.35 (m, 3H) ppm. α-Anomer: **^{13}C-NMR** (125 MHz, $CDCl_3$) δ 133.7, 118.0, 109.6, 96.4, 78.5, 75.9, 74.6, 68.1, 66.1, 28.1, 26.3, 17.6 ppm; β-Anomer: **^{13}C-NMR** (125 MHz, $CDCl_3$) δ 133.9, 118.5, 111.0, 97.0, 80.3, 75.1, 75.0, 71.1, 70.3, 28.2, 26.4, 17.9 ppm.

Spectroscopic data corresponded to those reported in the literature.[12]

(3a*R*,6*S*,7a*S*)-4-(Allyloxy)-2,2,6-trimethyldihydro-4*H*-[1,3]dioxolo[4,5-c]pyran-7(6*H*)-one (SI-59)

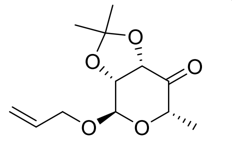
SI-59

Oxalyl chloride (7.90 mL, 92.1 mmol, 2.00 eq.) was dissolved in dry CH_2Cl_2 (38 mL) and treated with dry DMSO (13.1 mL, 184 mmol, 4.00 eq.) at -78 °C. After stirring for 40 min, glycoside **55** (11.3 g, 46.1 mmol, 1.00 eq.) was added. Stirring was continued for 50 min at −78 °C and DIPEA (31.5 mL, 184 mmol, 4.00 eq.) was dropped into the mixture. The solution was allowed to warm to room temperature and stirred for a further 16 h. Sat. aq. $Na_2S_2O_3$ solution was added, and the aqueous phase was extracted thrice with CH_2Cl_2. The combined organic phases were washed with brine, dried over Na_2SO_4 and the solvent was removed under reduced pressure. Purification by column chromatography (SiO_2, pentane/EtOAc 8:1) afforded ketone **SI-59** (9.52 g, 92%, only α) as a colourless oil. $\mathbf{R_f}$ = 0.79 (hexanes/EtOAc 7:3); $[\alpha]_D^{20}$ −125.1° (c 1.0 in $CHCl_3$); **IR** ν_{max}/cm^{-1} 2989 (m), 2938 (m), 2922 (m), 2876 (w), 1742 (s), 1456 (w), 1375 (m), 1228 (m), 1162 (m), 1107 (s), 1979 (s), 1012 (s), 932 (m), 857 (m); **^{1}H-NMR** (500 MHz, $CDCl_3$) δ 5.89 (m, 1H), 5.31 (m, 1H), 5.24 (m, 1H), 5.00 (s, 1H), 4.45 (q, 2H, J = 5.7 Hz), 4.28 (q, 1H, J = 6.8 Hz), 4.24 (m, 1H), 4.08 (m, 1H), 1.49 (s, 3H), 1.39 (d, 3H, J = 6.8 Hz), 1.36 (s, 3H), ppm; **^{13}C-NMR** (125 MHz, $CDCl_3$) δ 204.8, 133.1, 118.5, 111.5, 96.1, 78.9, 76.1, 70.2, 68.9, 26.9, 25.6, 16.0 ppm.

Spectroscopic data corresponded to those reported in the literature.[13]

(3a*R*,6*S*,7*R*,7a*R*)-4-(Allyloxy)-2,2,6-trimethyltetrahydro-4*H*-[1,3]dioxolo[4,5-c]pyran-7-ol (11)

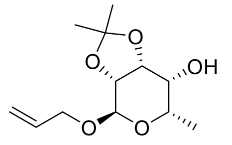
11

A solution of ketone **SI-59** (9.52 g, 39.3 mmol, 1.00 eq.) in EtOH *p.a.* (157 mL) was treated with $NaBH_4$ (1.64 g, 43.2 mmol, 1.10 eq.) at 0 °C. The suspension was stirred for 1.5h and the solid was filtered off over celite®. The solvent was removed under reduced pressure. Column chromatography (SiO_2, pentane/EtOAc, 7:1→6:1→4:1) gave alcohol **11** (9.19 g, 96%, only α) as a colourless liquid. $\mathbf{R_f}$ = 0.53 (hexanes/EtOAc 3:2); $[\alpha]_D^{20}$ −38.5° (c 1.0 in $CHCl_3$); **IR** ν_{max}/cm^{-1} 3528 (m), 2984 (m), 2936 (m), 1381 (m), 1255 (m), 1215 (m), 1152 (m), 1073 (s), 1019 (m), 991 (s), 852 (m); **^{1}H-NMR** (500 MHz, $CDCl_3$) δ 5.92 (m, 1H), 5.31 (d, 1H, J = 17.0 Hz), 5.22 (d, 1H, J = 10.3 Hz), 5.08 (s, 1H), 4.22 (q, 1H, J = 5.9 Hz), 4.20 (m, 1H), 4.07 (d, 1H, J = 6.2 Hz), 4.03 (dd, 1H, J = 6.2, 12.8 Hz), 3.89 (q, 1H, J = 6.7 Hz), 3.55 (t, 1H, J =

5.9 Hz), 2.18 (d, 1H, J = 6.7 Hz), 1.59 (s, 3H), 1.38 (s, 3H), 1.32 (d, 3H, J = 6.7 Hz) ppm; **^{13}C-NMR** (125 MHz, $CDCl_3$) δ 133.8, 118.0, 109.4, 96.8, 73.4, 73.1, 68.4, 67.0, 64.5, 26.0, 25.4, 16.8 ppm.

Spectroscopic data corresponded to those reported in the literature.[13]

(3a*R*,6*S*,7*R*,7a*R*)-4-(Allyloxy)-7-(benzyloxy)-2,2,6-trimethyltetrahydro-4*H*-[1,3]dioxolo-[4,5-c]pyran (56a)

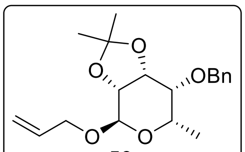

56a

A solution of alcohol **11** (8.98 g, 36.8 mmol, 1.00 eq.) in dry DMF (142 mL) was treated with NaH (2.82 g, 118 mmol, 3.20 eq.) and imidazole (225 mg, 3.31 mmol, 0.09 eq.) at 0 °C. The solution was stirred for 35 min, BnBr (6.33 mL, 53.3 mmol, 1.45 eq.) and TBAI (1.36 g, 3.68 g, 0.10 eq.) were added and stirring was continued for 17 h at room temperature. H_2O and EtOAc were added, the phases were separated, and the aqueous phase was extracted thrice with EtOAc. The combined organic phases were washed with H_2O and brine, dried over Na_2SO_4 and the solvent was removed under reduced pressure. Purification by column chromatography (SiO_2, pentane/EtOAc 7:1) afforded benzylated glycoside **56a** (12.2 g, quant., only α) as a colourless solid. **R_f** = 0.76 (hexanes/EtOAc 3:2); **mp** 27 °C; $[\boldsymbol{\alpha}]_{\boldsymbol{D}}^{\mathbf{20}}$ $-12.7°$ (c 1.0 in $CHCl_3$); **IR** ν_{max}/cm^{-1} 2984 (m), 2933 (m), 2910 (m), 1455 (m), 1380 (m), 1369 (m), 1252 (m), 1214 (m), 1161 (m), 1144 (m), 1055 (s), 1025 (s), 924 (w), 858 (m); **^{1}H-NMR** (500 MHz, $CDCl_3$) δ 7.40-7.26 (m, 5H), 5.89 (dddd, 1H, J = 5.6, 6.2, 10.5, 17.1 Hz), 5.27 (dq, 1H, J = 1.6, 17.1 Hz), 5.18 (d, 1H, J = 1.6, 10.5 Hz), 4.98 (d, 1H, J = 1.5 Hz), 4.85 (d, 1H, J = 12.0 Hz), 4.56 (d, 1H, J = 12.0 Hz), 4.40 (dd, 1H, J = 4.6, 6.6 Hz), 4.18 (ddt, 1H, J = 1.5, 5.1, 12.7 Hz), 4.07 (dd, 1H, J = 1.7, 6.7 Hz), 4.01 (ddt, 1H, J = 1.5, 6.3, 12.8 Hz), 3.88 (dq, 1H, J = 3.3, 6.7 Hz), 3.59 (dd, 1H, J = 3.3, 4.3 Hz), 1.56 (s, 3H), 1.37 (s, 3H), 1.20 (d, 3H, J = 6.7 Hz) ppm; **^{13}C-NMR** (125 MHz, $CDCl_3$) δ 138.1, 134.0, 128.7, 128.4, 127.9, 117.7, 110.1, 97.0, 74.5, 74.3, 73.8, 72.8, 68.6, 65.8, 26.4, 25.6, 16.9 ppm. **HRMS** ESI *m/z* $[M + Na]^+$ calcd. for $C_{19}H_{26}O_5Na$ 357.16685, found 357.16725.

(3*R*,4*S*,5*S*,6*S*)-2-(Allyloxy)-5-(benzyloxy)-6-methyltetrahydro-2*H*-pyran-3,4-diol (SI-60a)

Carbohydrate **56a** (12.2 g, 36.4 mmol, 1.00 eq.) was dissolved in H_2O (7 mL) and AcOH (64 mL). The solution was stirred at 110 °C for 1.5 h. Toluene was added and the volatiles were

removed under reduced pressure. This procedure was repeated twice. The crude product was purified by column chromatography (SiO_2, pentane/EtOAc 4:1→2:1) to give deprotected carbohydrate **SI-60a** (9.94 g, 93%, only α) as a colourless oil in 93% yield. **R$_f$** = 0.65 (hexanes/EtOAc 3:2); $[\alpha]_D^{20}$ −103.3° (c 1.0 in $CHCl_3$); **IR** ν_{max}/cm^{-1} 3475 (m), 2932 (m), 1736 (w), 1455 (w), 1383 (w), 1360 (w), 1103 (s), 1052 (s), 1008 (s), 928 (w), 813 (m), 737 (m); **^{1}H-NMR** (500 MHz, $CDCl_3$) δ 7.38-7.29 (m, 5H), 5.89 (m, 1H), 5.28 (dq, 1H, J = 1.4, 17.2 Hz), 5.19 (dq, 1H, J = 1.4, 10.4 Hz), 4.90 (d, 1H, J = 1.1 Hz), 4.78 (d, 1H, J = 11.0 Hz), 4.70 (d, 1H, J = 11.0 Hz), 4.15 (ddt, 1H, J = 1.4, 5.1, 13.0 Hz), 3.99 (ddt, 1H, J = 1.4, 6.0, 13.0 Hz), 3.92 (q, 1H, J = 6.6 Hz), 3.88 (dt, 1H, J = 3.4, 10.3 Hz), 3.69 (m, 1H), 3.64 (m, 1H), 3.39 (m, 1H), 2.79 (m, 1H), 1.27 (d, 3H, J = 6.6 Hz) ppm; **^{13}C-NMR** (125 MHz, $CDCl_3$) δ 137.6, 133.9, 128.7, 128.3, 128.2, 117.4, 100.2, 81.5, 76.8, 70.9, 68.3, 66.9, 66.1, 17.1 ppm; **HRMS** ESI *m/z* $[M + Na]^+$ calcd. for $C_{16}H_{22}O_5Na$ 317.13568, found 317.13594.

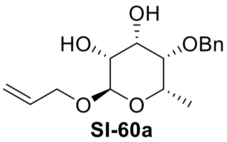

SI-60a

(3*R*,4*S*,5*R*,6*S*)-2-(Allyloxy)-5-(benzyloxy)-3-hydroxy-6-methyltetrahydro-2*H*-pyran-4-yl acetate (57a)

A solution of diol **SI-60a** (6.12 g, 21.0 mmol, 1.00 eq.) in toluene *p.a.* (1.00 L) was treated with Bu_2SnO (6.27 g, 25.2 mmol, 1.20 eq.) and stirred for 4 h under reflux with a water separator. AcCl (1.60 mL, 22.1 mmol, 1.05 eq.) was added at 0 °C and stirred for a further 30 min. The volatiles were removed under reduced pressure and the crude product was purified by column chromatography (SiO_2, pentane/EtOAc 5:1) to give product **57a** (5.56 g, 79%) as a colourless oil. **R$_f$** = 0.56 (hexanes/EtOAc 3:2); $[\alpha]_D^{20}$ −128.0° (c 1.0 in $CHCl_3$); **IR** ν_{max}/cm^{-1} 3487 (m), 2937 (w), 1740 (s), 1432 (w), 1455 (w), 1362 (m), 1229 (s), 1150 (m), 1116 (s), 1045 (s), 1011 (s), 919 (m), 752 (m), 731 (m); **^{1}H-NMR** (500 MHz, $CDCl_3$) δ 7.39-7.29 (m, 5H), 5.88 (m, 1H), 5.28 (dq, 1H, J = 1.4, 17.2 Hz), 5.19 (d, 1H, J = 1.4, 10.4 Hz), 5.08 (t, 1H, J = 3.1 Hz), 4.90 (d, 1H, J = 1.5 Hz), 4.77 (d, 1H, J = 11.3 Hz), 4.61 (d, 1H, J = 11.3 Hz), 4.16 (ddt, 1H, J = 1.3, 5.3, 13.0 Hz), 4.11, (d, 1H, J = 11.1 Hz), 4.00 (m, 2H), 3.83 (m, 1H), 3.77 (m, 1H), 2.11 (s, 3H), 1.25 (d, 3H, J = 6.5 Hz) ppm; **^{13}C-NMR** (125 MHz, $CDCl_3$) δ 170.6, 137.3, 133.9, 128.7, 128.4, 128.4, 117.6, 100.7, 79.0, 76.1, 70.1, 69.3, 68.4, 66.4, 21.3, 16.9 ppm; **HRMS** ESI *m/z* $[M + Na]^+$ calcd. for $C_{18}H_{24}O_6Na$ 359.14651, found 359.14602.

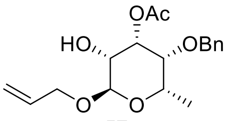

57a

(3*R*,4*R*,5*R*,6*S*)-2-(Allyloxy)-5-(benzyloxy)-3-methoxy-6-methyltetrahydro-2*H*-pyran-4-yl acetate (63)

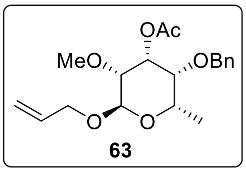

A solution of carbohydrate **57a** (2.75 g, 8.20 mmol, 1.00 eq.) in dry CH_2Cl_2 (33 mL) was treated with $TMSCHN_2$ (1.8-2.4M in hexanes, 20.5 mL, 40.9 mmol, 5.00 eq.) and HBF_4 (50 wt% in H_2O, 2.00 mL, 16.4 mmol, 2.00 eq.) at 0 °C. The reaction mixture was stirred for 3 h at 0 °C, $TMSCHN_2$ (1.8-2.4M in hexanes, 20.5 mL, 40.9 mmol, 5.00 eq.) and HBF_4 (50 wt% in H_2O, 2.00 mL, 16.4 mmol, 2.00 eq.) were added again and stirring was continued for 1 h. This was repeated a second time. The reaction was quenched by addition of sat. aq. $NaHCO_3$ solution. The aqueous phase was extracted thrice with CH_2Cl_2, the combined organic phases were washed with brine and dried over Na_2SO_4. The volatiles were removed under reduced pressure. Purification by column chromatography (SiO_2, pentane/EtOAc 4:1→2:1) gave product **63** (2.58 g, 90%) as a colourless resin. **R$_f$** = 0.61 (hexanes/EtOAc 3:2); $[\boldsymbol{\alpha}]_D^{20}$ −79.1° (c 1.0 in $CHCl_3$); **IR** ν_{max}/cm^{-1} 3004 (w), 2989 (w), 1744 (w), 1276 (m), 1261 (m), 1092 (w), 1051 (w), 764 (s), 750 (s); **^{1}H-NMR** (500 MHz, $CDCl_3$) δ 7.39-7.25 (m, 5H), 5.89 (m, 1H), 5.28 (dq, 1H, J = 11.6, 7.2 Hz), 5.18 (d, 1H, J = 1.4, 10.4 Hz), 5.17 (t, 1H, J = 3.5 Hz), 4.95 (d, 1H, J = 2.1 Hz), 4.71 (d, 1H, J = 12.2 Hz), 4.65 (d, 1H, J = 12.2 Hz), 4.17 (ddt, 1H, J = 1.5, 5.1, 13.0 Hz), 4.02-3.94, (m, 2H), 3.61 (m, 1H), 3.51 (s, 3H), 3.43 (m, 1H), 2.03 (s, 3H), 1.24 (d, 3H, J = 6.7 Hz) ppm; **^{13}C-NMR** (125 MHz, $CDCl_3$) δ 170.5, 138.6, 134.0, 128.4, 128.3, 127.7, 127.6, 117.4, 97.5, 77.5, 76.1, 74.7, 71.3, 68.2, 67.0, 59.9, 21.3, 16.6 ppm; **HRMS** ESI *m/z* [M + Na]$^+$ calcd. for $C_{19}H_{26}O_6Na$ 373.16216, found 373.16129.

(2*S*,3*R*,4*R*,5*R*)-3-(Benzyloxy)-6-hydroxy-5-methoxy-2-methyltetrahydro-2*H*-pyran-4-yl acetate (58a)

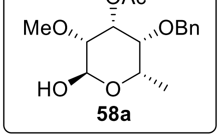

Glycoside **63** (1.00 g, 2.85 mmol, 1.00 eq.) was dissolved in AcOH (29 mL) and $Pd(PPh_3)_4$ (989 mg, 856 µmol, 0.30 eq.) was added at room temperature. The mixture was stirred for 17 h and quenched with sat. aq. $NaHCO_3$ solution as well as solid $NaHCO_3$. The aqueous phase was extracted thrice with EtOAc, the combined organic phases were washed with brine and dried over Na_2SO_4. The solvent was removed under reduced pressure and crude product was purified by column chromatography (SiO_2, pentane/EtOAc 1.5:1→1:1) to afford hemi-acetal **58a** (784 mg, 89%, α:β 6:1) as a light yellow resin. **R$_f$** = 0.52 (CH_2Cl_2/MeOH 9:1); $[\boldsymbol{\alpha}]_D^{20}$ −41.1° (c

1.0 in $CHCl_3$); IR v_{max}/cm^{-1} 3438 (m), 2977 (w), 2934 (m), 2896 (m), 2837 (w), 1739 (s), 1497 (w), 1455 (m), 1372 (m), 1236 (s), 1157 (m), 1132 (m), 1096 (s), 1044 (s), 968 (m), 913 (m), 817 (w), 750 (s), 699 (m), 677 (m); α-anomer **^{1}H-NMR** (500 MHz, $CDCl_3$) δ 7.39-7.26 (m, 5H), 5.31 (t, 2H, J = 3.2 Hz), 4.71 (d, 1H, J = 12.1 Hz), 4.64 (d, 1H, J = 12.1 Hz), 4.22 (dq, 1H, J = 2.6, 6.6 Hz), 3.63 (t, 1H, J = 2.8 Hz), 3.51 (s, 3H), 3.87 (t, 1H, J = 3.2 Hz), 2.70 (d, 1H, J = 3.7 Hz), 2.06 (s, 3H), 1.28 (d, 3H, J = 6.7 Hz) ppm; β-anomer **^{1}H-NMR** (500 MHz, $CDCl_3$) δ 7.39-7.26 (m, 5H), 4.85 (t, 1H, J = 3.3 Hz), 4.75 (d, 1H, J = 12.3 Hz), 4.68 (dd, 1H, J = 1.8, 12.8 Hz), 4.63 (d, 1H, J = 12.3 Hz), 4.04 (d, 1H, J = 12.8 Hz), 3.67 (s, 3H), 3.56 (m, 2H), 3.53 (m, 1H), 1.99 (s, 3H), 1.30 (m, 3H) ppm; α-anomer **^{13}C-NMR** (125 MHz, $CDCl_3$) δ 170.5, 138.4, 128.4, 128.3, 127.8, 92.7, 77.9, 75.7, 74.2, 70.3, 67.8, 59.7, 21.3, 16.4, ppm; β-anomer **^{13}C-NMR** (125 MHz, $CDCl_3$) δ 170.3, 138.3, 128.8, 128.4, 128.0, 93.8, 77.9, 75.6, 75.4, 74.4, 71.4, 61.6, 21.1, 16.9 ppm; **HRMS** ESI m/z [M + Na]$^+$ calcd. for $C_{16}H_{22}O_6Na$ 333.13033, found 333.213086.

(3*R*,4*R*,5*R*,6*S*)-4-Acetoxy-5-(benzyloxy)-3-methoxy-6-methyltetrahydro-2*H*-pyran-2-yl-2-(hex-1-yn-1-yl)benzoate (60a)

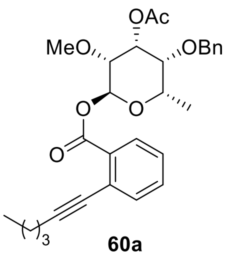

60a

A solution of hemi-acetal **58a** (784 mg, 2.53 mmol, 1.00 eq.) in dry CH_2Cl_2 (3.6 mL) was treated with acid **59** (656 mg, 3.03 mmol, 1.20 eq.), DCC (782 mg, 3.79 mmol, 1.50 eq.) and DMAP (463 mg, 3.79 mmol, 1.50 eq.) at room temperature. After stirring for 3 h, the solids were filtered off over celite®. The organic phase was washed with sat. aq. $NaHCO_3$ solution and the aqueous phase was extracted twice with CH_2Cl_2. The combined organic phases were dried over Na_2SO_4, and solvents were removed under reduced pressure. Purification by column chromatography (SiO_2, pentane/EtOAc 6:1→4:1→2:1) gave product **60a** (1.16 g, 93% mmol, α:β 2.9:1) as a colourless resin. **R_f** = 0.71 (hexanes/EtOAc 1:1); $[\boldsymbol{\alpha}]_D^{20}$ −9.6° (c 1.0 in $CHCl_3$); **IR** v_{max}/cm^{-1} 2934 (w), 2872 (w), 2229 (w), 1737 (s), 1596 (w), 1567 (w), 1484 (w), 1456 (w), 1366 (m), 1275 (m), 1233 (s), 1131 (m), 1069 (s), 1042 (s), 989 (m), 946 (m), 916 (m), 751 (s), 698 (m); α-anomer **^{1}H-NMR** (500 MHz, $CDCl_3$) δ 7.89 (dd, 1H, J = 1.2, 8.0 Hz), 7.53 (dd, 1H, J = 1.2, 8.0 Hz), 7.45 (dq, 1H, J = 2.3, 7.6 Hz), 7.40-7.27 (m, 6H), 6.51 (d, 1H, J = 2.0 Hz), 5.23 (t, 1H, J = 3.5 Hz), 4.75 (d, 1H, J = 12.2 Hz), 4.69 (d, 1H, J = 12.2 Hz), 4.24 (dq, 1H, J = 1.8, 6.5 Hz), 3.73 (m, 1H), 3.60 (s, 3H), 3.59 (s, 1H), 2.47 (m, 2H), 2.06 (s, 3H), 1.61 (m, 2H), 1.48 (m, 2H), 1.28 (d, 3H, J = 6.6 Hz), 0.94 (t, 3H, J = 7.4 Hz) ppm; β-anomer **^{1}H-NMR** (500 MHz, $CDCl_3$) δ 8.09 (dd, 1H, J = 1.2,

8.0 Hz), 7.53 (dd, 1H, *J* = 1.2, 8.0 Hz), 7.45 (dq, 1H, *J* = 2.3, 7.6 Hz), 7.40-7.27 (m, 6H), 6.07 (d, 1H, *J* = 2.2 Hz), 5.24 (t, 1H, *J* = 3.5 Hz), 4.72 (d, 1H, *J* = 12.2 Hz), 4.68 (d, 1H, *J* = 12.2 Hz), 3.89 (dq, 1H, *J* = 2.9, 6.7 Hz), 3.69 (dd, 1H, *J* = 1.8, 3.5 Hz), 3.65 (t, 1H, *J* = 3.5 Hz), 3.57 (s, 3H), 2.45 (m, 2H), 2.09 (s, 3H), 1.61 (m, 2H), 1.48 (m, 2H), 1.37 (d, 3H, *J* = 6.7 Hz), 0.94 (t, 3H, *J* = 7.3 Hz) ppm; α-anomer **^{13}C-NMR** (125 MHz, $CDCl_3$) *δ* 170.5, 164.4, 138.3, 135.0, 132.1, 130.7, 130.6, 128.4, 128.3, 127.8, 125.0, 96.6, 93.2, 79.6, 76.3, 75.5, 74.8, 70.9, 70.0, 60.1, 30.8, 22.2, 21.2, 19.6, 16.8, 13.8 ppm; significant signals β-anomer **^{13}C-NMR** (125 MHz, $CDCl_3$) *δ* 170.5, 164.0, 138.2, 134.6, 130.75, 130.73, 128.34, 128.27, 127.3, 127.0, 125.7, 97.1, 92.2, 79.1, 76.4, 74.2, 73.7, 72.2, 60.7, 30.8, 22.2, 21.1, 19.7, 17.0 ppm; **HRMS** ESI *m/z* $[M + Na]^+$ calcd. for $C_{29}H_{34}O_7Na$ 517.21967, found 517.21924.

(2*S*,3*R*,4*R*,5*R*)-3-(Benzyloxy)-6-((*S*,*Z*)-3-(cyclohexyl(hydroxy)methylene)-5-isopropyl-2,4-dioxopyrrolidin-1-yl)-5-methoxy-2-methyltetrahydro-2*H*-pyran-4-yl acetate (62a)

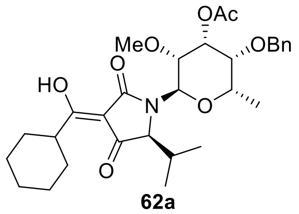

62a

Ester **60a** (200 mg, 404 µmol, 1.00 eq.) and 3-acyl tetramic acid **61** (152 mg, 607 µmol, 1.50 eq.) were dissolved in dry toluene (1.00 mL). $AuPPh_3NTf_2$ (59.8 mg, 80.9 µmol, 0.20 eq.) was added and the mixture was stirred at 40 °C for 17 h. All volatiles were removed in vacuo. The crude product was purified by column chromatography (SiO_2 C-18, 40% MeCN in H_2O + 0.1% HCO_2H→60% MeCN in H_2O + 0.1% HCO_2H→80% MeCN in H_2O + 0.1% HCO_2H→100% MeCN in H_2O + 0.1% HCO_2H) to give product **62a** as a light-yellow resin (127 mg, 58%, α:β 10:1). Anomers were separated by HPLC. Minor impurities occurred due to third tautomer $\mathbf{R_f}$ = 0.49 (hexanes/EtOAc 1:1); $[\alpha]_D^{20}$ −8.5° (c 1.0 in $CHCl_3$); **IR** ν_{max}/cm^{-1} 2932 (m), 2857 (w), 1744 (s), 1796 (s), 1647 (s), 1607 (s), 1453 (m), 1364 (w), 1312 (w), 1232 (s), 1089 (s), 1027 (w), 752 (m), 698 (w); α-anomer **^{1}H-NMR** (500 MHz, CD_3OD) *δ* 7.40-7.26 (m, 5H), 6.00 (t, 1H, *J* = 3.0 Hz), 5.09 (br. s, 1H), 4.67 (d, 1H, *J* = 11.6 Hz), 4.51 (d, 1H, *J* = 11.6 Hz), 4.26 (m, 2H), 3.84 (br. s, 1H), 3.83 (dd, 1H, *J* = 3.2, 6.5 Hz), 3.43 (tt, 1H, *J* = 3.3, 11.5 Hz), 3.32 (s, 3H), 2.24 (m, 1H), 2.13 (s, 3H), 1.86-1.70 (m, 5H), 1.51 (m, 2H), 1.43 (d, 3H, *J* = 7.1 Hz), 1.39 (m, 2H), 1.27 (m, 1H), 1.17 (d, 3H, *J* = 7.1 Hz), 0.89 (d, 3H, *J* = 7.1 Hz) ppm; α-anomer major tautomer **^{13}C-NMR** (125 MHz, $CDCl_3$) *δ* 193.9, 192.6, 175.9, 170.4, 137.8, 128.6, 128.0, 127.7, 101.2, 75.6, 74.2, 73.2, 71.9, 71.0, 66.4, 57.1, 41.0, 30.4, 29.0, 28.5, 25.8, 25.71, 25.70, 25.6, 21.4, 18.1, 16.1, 13.6 ppm; significant signals α-anomer minor tautomer **^{13}C-NMR** (125 MHz, $CDCl_3$) *δ* 199.7, 197.6, 170.5, 168.4, 137.8, 128.6, 128.2, 127.7, 104.8, 73.1, 71.7, 70.9, 66.6, 57.0, 41.8, 30.2,

29.1, 28.4, 25.8, 25.5, 21.4, 18.2, 15.7, 13.6 ppm; **HRMS** ESI *m/z* $[M + H]^+$ calcd. for $C_{30}H_{42}NO_8$ 544.29049, found 544.28949.

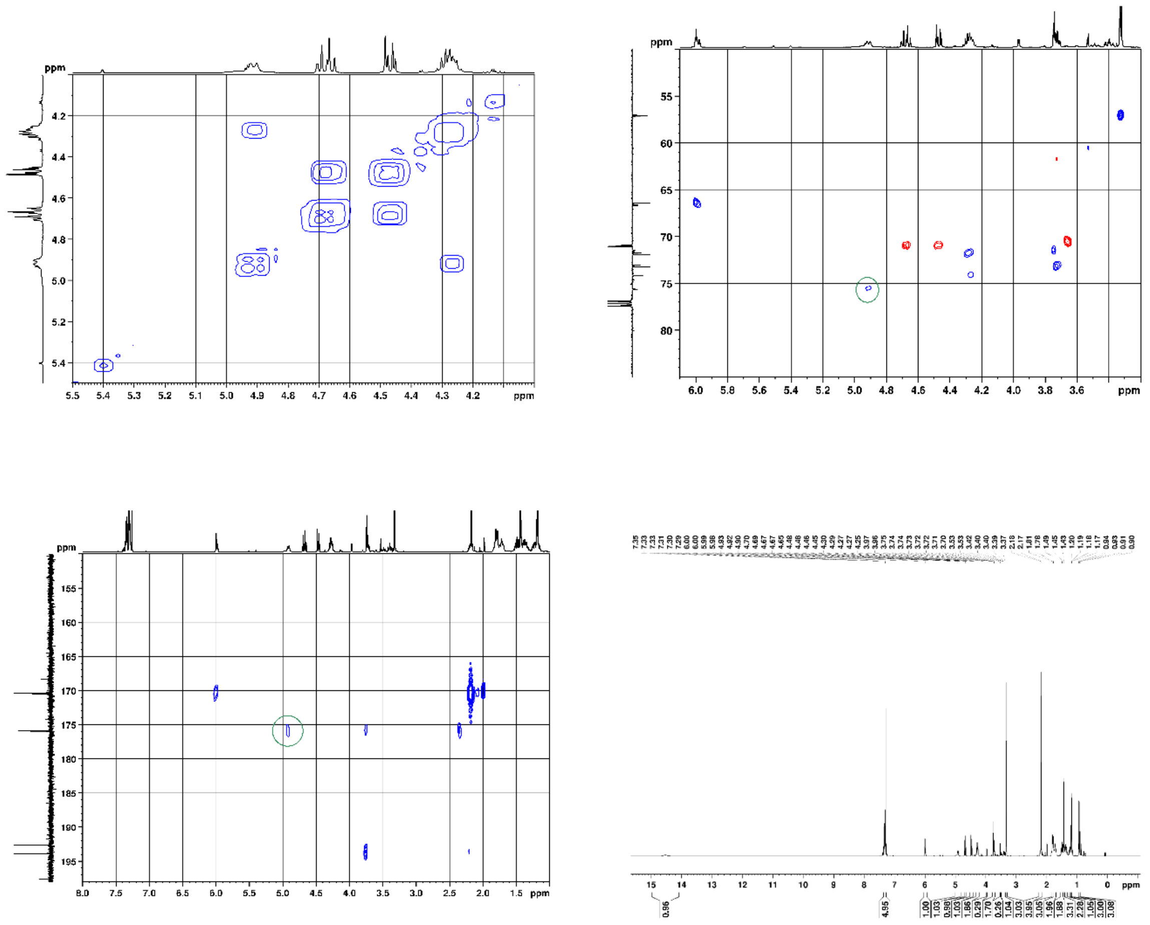

Fig. S8. 2D-NMR-spectra [^{1}H-^{1}H-COSY (top, left), ^{1}H-^{13}C-HSQC (top, right), ^{1}H-^{13}C-HMBC (bottom, left)] of **62a** for elucidation of *N*,*O*-acetal formation. ^{1}H-NMR-spectrum ($CDCl_3$) of **62a** (bottom, right).

2D-NMR-spectra (COSY, HSQC, HMBC) as well as 1D-NMR-spectra (^{1}H and ^{13}C, $CDCl_3$) clearly showed the exclusive formation of an *N*,O-acetal. An *O*-glycosylation with tautomers of 3-acyl-tetramic acids could is conceivable, yet was not observed.[1] Via COSY and HSQC the signal at 4.92 ppm was assigned to the anomeric proton (Fig. S8, top). The chemical shift of the anomeric C-atom (δ = 75.6 ppm) had a distinct high-field shift compared to an *O*,*O*-acetal ($\delta \approx$ 95 ppm). The chemical shifts of the anomeric position are in full accordance with the results of Yang *et al.*[2] As known from the literature the enolization of the amide is highly unfavoured and therefore an *O*-glycosylation with enolized amide is unlikely.[1] HMBC indicated a coupling of the anomeric proton of talose-derivative with amide-C-atom (Fig. S8, bottom left, green circle) confirming the spatial proximity to the amide-C. In the ^{1}H-NMR-spectrum a signal for an enolic

proton was found at 14.5 ppm while no signal for NH was observed. In an additional experiment for *N*-glycosylation of tetramic acid derivatives, the *O*-glycosylation took place (for synthesis see Scheme S19). For proof of *N*-glycosylation the spectra can be compared with those of the accidently formed *O*,*O*-acetal **SI-62**. In the ^{1}H-spectrum of **SI-62** (Fig. S9, top) a signal for an amide proton (no HSQC-correlation, Fig. S9 bottom) instead of enolic proton signal was indicated at 5.84 ppm. The anomeric H-atom (δ = 5.52 ppm) and the anomeric C-atom (δ = 98.3 ppm) of **SI-62** were shifted downfield compared to the *N*,*O*-acetal **62a**.

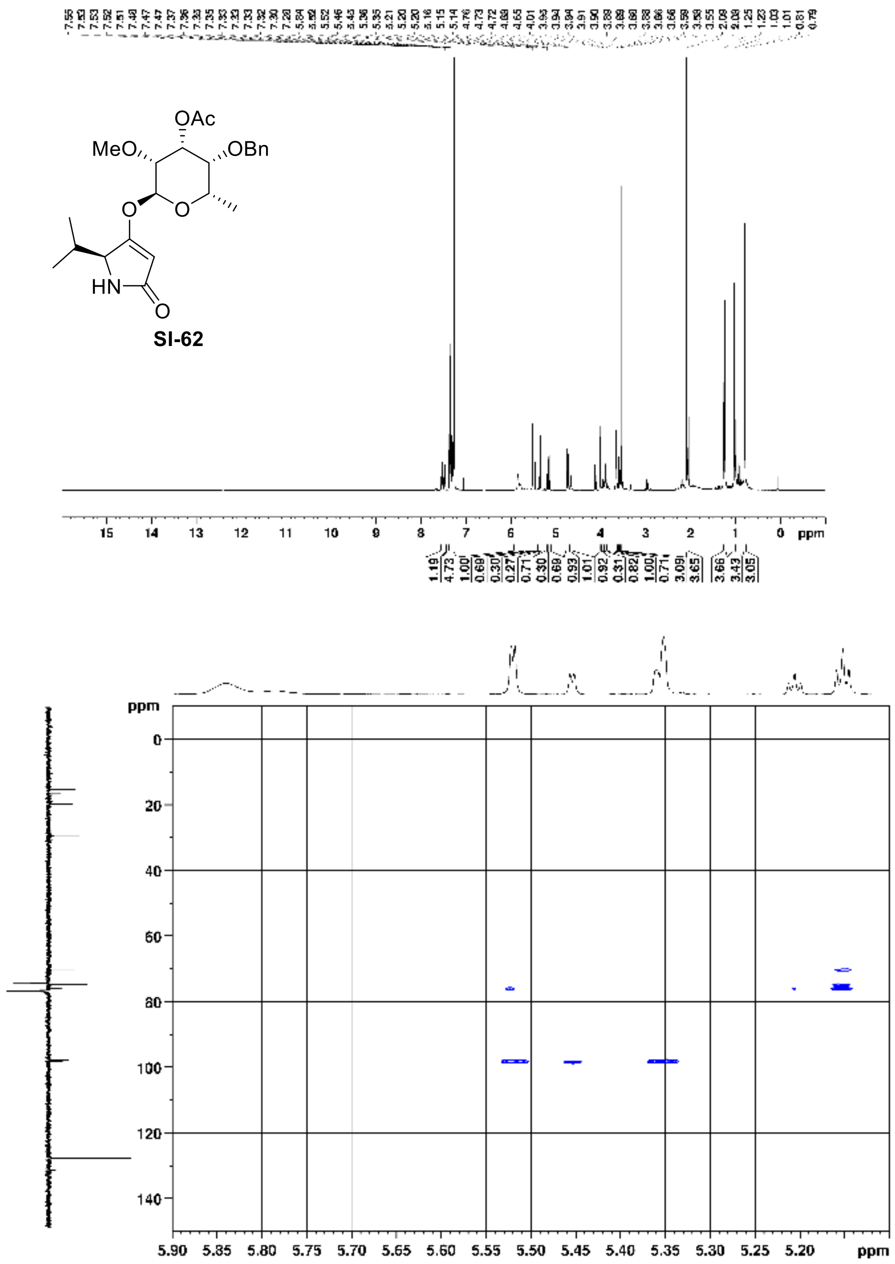

Fig. S9. ^{1}H-NMR-spectrum (top) and ^{1}H-^{13}C-HSQC-spectrum (bottom) of **SI-62** for comparison with spectra of the *N*,*O*-acetal.

(((3a*R*,6*S*,7*R*,7a*S*)-4-(Allyloxy)-2,2,6-trimethyltetrahydro-4*H*-[1,3]dioxolo[4,5-c]pyran-7-yl)oxy)(*tert*-butyl)dimethylsilane (56b)

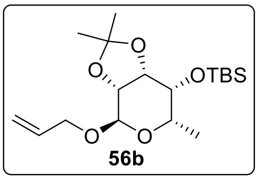

Alcohol **11** (772 mg, 3.16 mmol, 1.00 eq.) in dry CH_2Cl_2 (55 mL) was treated with pyridine (2.55 mL, 31.6 mmol, 10.0 eq.) and TBSOTf (2.18 mL, 9.48 mmol, 3.00 eq.) at 0 °C. The solution was stirred for 5 h and the reaction was quenched by addition of sat. aq. $NaHCO_3$ solution. The aqueous phase was extracted with EtOAc thrice and the combined organic phases were washed with brine as well as dried over Na_2SO_4. After removal of the solvent *in vacuo* the crude product was purified by column chromatography (SiO_2, pentane/EtOAc 6:1) to give TBS-ether **56b** (1.16 g, quant.) as a colourless liquid. $\mathbf{R_f}$ = 0.88 (hexanes/EtOAc 3:2); $[\alpha]_D^{20}$ −58.3° (c 1.0 in $CHCl_3$); **IR** ν_{max}/cm^{-1} 2988 (m), 2933 (m), 2889 (m), 2865 (m), 1473 (w), 1381 (w), 1276 (s), 1260 (s), 1979 (m), 1056 (m), 838 (m), 764 (s), 750 (s); **^{1}H-NMR** (500 MHz, $CDCl_3$) δ 5.92 (dddd, 1H, J = 5.2, 6.3, 10.7, 17.2 Hz), 5.30 (dq, 1H, J = 1.5, 17.2 Hz), 5.19 (dq, 1H, J = 1.5, 10.7 Hz), 4.85 (d, 1H, J = 4.1 Hz), 4.29 (dd, 1H, J = 3.6, 7.5 Hz), 4.26 (ddt, 1H, J = 1.3, 5.2, 12.8 Hz), 4.13 (dd, 1H, J = 3.6, 4.1 Hz), 4.08 (ddt, J = 1.3, 6.3, n.d. Hz), 4.07 (m, 1H), 3.95 (m, 1H), 1.52 (s, 3H), 1.33 (s, 3H), 1.33 (d, 3H, J = 6.5 Hz), 0.92 (s, 9H), 0.11 (s, 3H), 0.07 (s, 3H) ppm; **^{13}C-NMR** (125 MHz, $CDCl_3$) δ 134.2, 117.6, 110.2, 97.2, 76.2, 75.5, 69.8, 69.2, 67.5, 26.7, 26.2, 24.8, 18.5, 17.2, −4.01, −4.58 ppm; **HRMS** ESI *m/z* [M + Na]$^+$ calcd. for $C_{18}H_{34}O_5SiNa$ 381.20677, found 381.20547.

(3*R*,4*S*,5*S*,6*S*)-2-(Allyloxy)-5-((*tert*-butyldimethylsilyl)oxy)-6-methyltetrahydro-2*H*-pyran-3,4-diol (SI-60b)

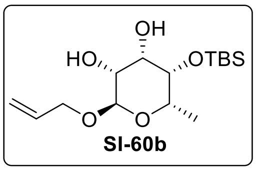

Fully protected carbohydrate **56b** (310 mg, 865 µmol, 1.00 eq.) was dissolved in EtOH *p.a.* (1.3 mL) and formic acid (1.3 mL). The solution was stirred at room temperature for 2.5 h. After addition of sat. aq. $NaHCO_3$ solution, the aqueous phase was extracted with EtOAc thrice. The combined organic phases were dried over Na_2SO_4, and the volatiles were removed *in vacuo*. The crude product was purified by column chromatography (SiO_2, pentane/EtOAc 9:1) to yield deprotected diol **SI-60b** (116 mg, 42%) as a colourless solid. $\mathbf{R_f}$ = 0.58 (hexanes/EtOAc 3:1); **mp** 69 °C; $[\alpha]_D^{20}$ −91.6° (c 1.0 in $CHCl_3$); **IR** ν_{max}/cm^{-1} 3405 (m), 3359 (m), 2945 (m), 2929 (m), 2882 (w), 2858 (m), 1471 (w), 1425 (w), 1351 (w), 1276 (m), 1260 (m), 1167 (w), 1143 (w), 1104 (m), 1067 (m), 1044 (w), 1014 (m), 996 (m), 916 (w), 837 (m), 765 (s), 749 (s),

678 (w); **^{1}H-NMR** (500 MHz, $CDCl_3$) δ 5.89 (dddd, 1H, J = 5.2, 6.2, 10.7, 17.0 Hz), 5.28 (dq, 1H, J = 1.6, 17.0 Hz), 5.19 (dq, 1H, J = 1.6, 10.7 Hz), 4.92 (d, 1H, J = 1.3 Hz), 4.16 (ddt, 1H, J = 1.4, 5.2, 13.0 Hz), 4.00 (ddt, J = 1.4, 6.0, 13.0 Hz), 3.89 (q, 1H, J = 6.6 Hz), 3.80 (m, 1H), 3.75 (dt, 1H, J = 3.1, 10.7 Hz), 3.68 (m, 1H), 3.43 (d, 1H, J = 12.0 Hz), 2.61 (d, 1H, J = 10.7 Hz), 1.23 (d, 3H, J = 6.6 Hz), 0.95 (s, 9H), 0.19 (s, 3H), 0.12 (s, 3H) ppm; **^{13}C-NMR** (125 MHz, $CDCl_3$) δ 134.0, 117.5, 100.2, 75.1, 71.4, 68.3, 66.6, 66.4, 26.1, 18.4, 17.6, −3.88, −4.50 ppm; **HRMS** ESI *m/z* $[M + Na]^+$ calcd. for $C_{15}H_{30}O_5SiNa$ 341.17547, found 341.17505.

(3*R*,4*S*,5*R*,6*S*)-2-(Allyloxy)-5-((*tert*-butyldimethylsilyl)oxy)-3-hydroxy-6-methyltetra-hydro-2*H*-pyran-4-yl acetate (57b)

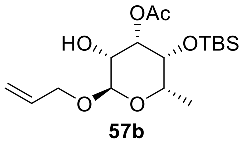
57b

To a solution of diol **SI-60b** (210 mg, 659 µmol, 1.00 eq.) in dry toluene (33 mL) was added Bu_2SnO (197 mg, 791 µmol, 1.20 eq.). The suspension was stirred under reflux for 3 h. AcCl (49.4 µL, 692 µmol, 1.05 eq.) was added at room temperature and stirring was continued for 1 h. All volatiles were removed under reduced pressure. Purification of the crude product (SiO_2, pentane/EtOAc 9:1) resulted in acetylated carbohydrate **57b** (220 mg, 93%) as a colourless liquid. **R_f** = 0.35 (hexanes/EtOAc 5:1); $[\alpha]_D^{20}$ −93.8° (c 1.0 in $CHCl_3$); **IR** ν_{max}/cm^{-1} 3504 (m), 2956 (m), 2932 (m), 2900 (m), 2860 (m), 1741 (m), 1473 (w), 1432 (w), 1374 (w), 1276 (m), 1260 (s), 1235 (m), 1180 (w), 1118 (m), 1070 (m), 1001 (s), 938 (w), 839 (m), 765 (s), 750 (s), 680 (w); **^{1}H-NMR** (500 MHz, $CDCl_3$) δ 5.89 (dddd, 1H, J = 5.1, 6.2, 10.5, 17.1 Hz), 5.29 (dq, 1H, J = 1.6, 17.1 Hz), 5.19 (dq, 1H, J = 1.6, 10.5 Hz), 5.00 (t, 1H, J = 2.9 Hz), 4.90 (d, 1H, J = 1.5 Hz), 4.17 (ddt, 1H, J = 1.5, 5.1, 13.0 Hz), 4.11 (d, 1H, J = 11.1 Hz), 4.01 (ddt, J = 1.5, 6.2, 13.0 Hz), 3.99 (q, 1H, J = 6.6 Hz), 3.92 (m, 1H), 3.80 (m, 1H), 2.15 (s, 3H), 1.23 (d, 3H, J = 6.6 Hz), 0.96 (s, 9H), 0.15 (s, 3H), 0.10 (s, 3H) ppm; **^{13}C-NMR** (125 MHz, $CDCl_3$) δ 170.6, 134.0, 117.6, 110.7, 73.2, 69.66, 69.65, 68.4, 66.8, 26.0, 21.5, 18.3, 17.5, −4.24, −4.41 ppm; **HRMS** ESI *m/z* $[M + Na]^+$ calcd. for $C_{17}H_{32}O_6SiNa$ 383.18604, found 383.18468.

(3*R*,4*S*,5*R*,6*S*)-2-(Allyloxy)-5-((*tert*-butyldimethylsilyl)oxy)-3-methoxy-6-methyltetra-hydro-2*H*-pyran-4-yl acetate (SI-61)

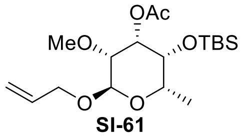
SI-61

Alcohol **57b** (40 mg, 111 µmol, 1.00 eq.) in dry CH_2Cl_2 (1.10 mL) was treated with Me_3OBF_4 (65.6 mg, 444 µmol, 4.00 eq.) and proton sponge (95.1 mg, 444 µmol, 4.00 eq.) at 0 °C and stirred at 40 °C for 21 h. The

reaction was quenched by addition of sat. aq. NH_4Cl solution. The aqueous phase was extracted with EtOAc thrice. Combined organic phases were washed with sat. aq. citric acid solution as well as brine and dried over Na_2SO_4. After removal of the solvent *in vacuo* and purification of the crude product by column chromatography (SiO_2, pentane/EtOAc 9:1) product **SI-61** (40 mg, 96%) was isolated as a colourless liquid. **R_f** = 0.63 (hexanes/EtOAc 4:1); $[\alpha]_D^{20}$ −30.0° (c 1.0 in $CHCl_3$); **IR** ν_{max}/cm^{-1} 2930 (m), 2900 (m), 2857 (m), 1745 (s), 1463 (w), 1374 (w), 1237 (s), 1197 (w), 1130 (m), 1091 (m), 1053 (m), 1004 (m), 859 (m), 838 (m), 765 (s), 750 (s); ^{1}**H-NMR** (500 MHz, $CDCl_3$) δ 5.91 (dddd, 1H, *J* = 5.2, 6.1, 10.5, 17.1 Hz), 5.29 (dq, 1H, *J* = 1.6, 17.1 Hz), 5.18 (dq, 1H, *J* = 1.6, 10.5 Hz), 5.16 (t, 1H, *J* = 3.4 Hz), 4.93 (d, 1H, *J* = 2.9 Hz), 4.18 (ddt, 1H, *J* = 1.4, 5.1, 13.0 Hz), 4.01 (ddt, *J* = 1.4, 6.1, 13.0 Hz), 3.96 (dq, 1H, *J* = 2.6, 6.6 Hz), 3.80 (t, 1H, *J* = 2.8 Hz), 3.43 (s, 3H), 3.36 (m, 1H), 2.13 (s, 3H), 1.25 (d, 3H, *J* = 6.6 Hz), 0.93 (s, 9H), 0.08 (s, 3H), 0.05 (s, 3H) ppm; ^{13}**C-NMR** (125 MHz, $CDCl_3$) δ 170.5, 134.1, 117.5, 97.1, 77.5, 71.3, 70.4, 70.1, 68.4, 68.3, 59.6, 26.0, 21.5, 18.5, 16.6, −4.40, −4.48 ppm; **HRMS** ESI *m/z* $[M + Na]^+$ calcd. for $C_{18}H_{34}O_6SiNa$ 397.20169, found 397.20114.

(2*S*,3*R*,4*S*,5*R*)-3-((*tert*-Butyldimethylsilyl)oxy)-6-hydroxy-5-methoxy-2-methyltetra-hydro-2*H*-pyran-4-yl acetate (58b)

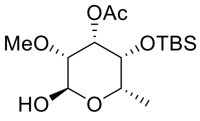

58b

Glycoside **SI-61** (820 mg, 2.19 mmol, 1.00 eq.) dissolved in EtOH *p.a.* (15 mL) was treated with DABCO (128 mg, 1.09 mmol, 0.50 eq.) and Wilkinson catalyst (101 mg, 109 µmol, 0.05 eq.). The reaction mixture was stirred at reflux for 15 h. After cooling down to room temperature, the suspension was filtered off over celite® and solvents were removed under reduced pressure. The crude product was dissolved in EtOAc *p.a.* (226 mL), H_2O (226 mL) and phosphate buffer (22.6 mL). A solution of I_2 (1.67 g, 6.57 mmol, 3.00 eq.) in EtOAc *p.a.* (92 mL) was added dropwise at room temperature. The mixture was stirred vigorously for 10 min. The reaction was quenched by addition of sat. aq. $Na_2S_2O_3$ solution. The aqueous phase was extracted with EtOAc thrice. Combined organic phases were washed with sat. aq. $Na_2S_2O_3$ solution as well as sat. aq. $NaHCO_3$ solution and dried over Na_2SO_4. After removal of the solvents under reduced pressure, purification of the crude product by column chromatography (SiO_2, pentane/EtOAc 2:1) gave semi-acetal **58b** (618 mg, 84%, α:β 4:1) as a colourless liquid. $\mathbf{R_f}$ = 0.86 (hexanes/EtOAc 4:1); $[\alpha]_D^{20}$ −45.9° (c 1.0 in $CHCl_3$); **IR** ν_{max}/cm^{-1} 3402 (m), 2949 (m), 2931 (m), 2886 (w), 2858 (m), 1746 (m), 1464 (w), 1373 (m), 1276 (s), 1260 (s), 1198 (m), 1139 (m), 1090 (m), 1047 (m), 962 (w), 858 (m), 837 (m), 765 (s), 750 (s); α-anomer ^{1}**H-NMR**

(500 MHz, $CDCl_3$) δ 5.32 (t, 1H, J = 3.3 Hz), 5.25 (t, 1H, J = 4.3 Hz), 4.15 (m, 1H), 3.85 (t, 1H, J = 3.4 Hz), 3.44 (s, 3H), 3.28 (m, 1H), 2.57 (d, 1H, J = 4.1 Hz), 2.12 (s, 3H), 1.30 (d, 3H, J = 6.8 Hz), 0.92 (s, 9H), 0.09 (s, 3H), 0.06 (s, 3H) ppm; β-anomer **^{1}H-NMR** (500 MHz, $CDCl_3$) δ 4.84 (t, 1H, J = 3.3 Hz), 4.70 (dd, 1H, J = 1.9, 12.5 Hz), 3.99 (d, 1H, J = 12.5 Hz), 3.72 (dt, 1H, J = 1.3, 3.3 Hz), 3.56 (dd, 1H, J = 1.6, 6.6 Hz), 3.54 (s, 3H), 3.49 (m, 1H), 2.17 (s, 3H), 1.28 (d, 3H, J = 6.6 Hz), 0.96 (s, 9H), 0.10 (s, 3H), 0.06 (s, 3H) ppm; α-anomer **^{13}C-NMR** (125 MHz, $CDCl_3$) δ 170.4, 91.7, 78.3, 70.5, 69.9, 69.6, 59.1, 26.0, 21.4, 18.4, 15.9, −4.54, −4.60 ppm; significant signals β-anomer **^{13}C-NMR** (125 MHz, $CDCl_3$) δ 93.5, 77.9, 74.1, 71.7, 69.5, 61.5, 26.1, 21.5, 17.5, −4.28 ppm; **HRMS** ESI *m/z* $[M + Na]^+$ calcd. for $C_{15}H_{30}O_6SiNa$ 357.17039, found 357.17020.

(3*R*,4*S*,5*R*,6*S*)-4-Acetoxy-5-((*tert*-butyldimethylsilyl)oxy)-3-methoxy-6-methyltetra-hydro-2*H*-pyran-2-yl 2-(2,2-dimethyl-2l6-but-1-yn-1-yl)benzoate (60b)

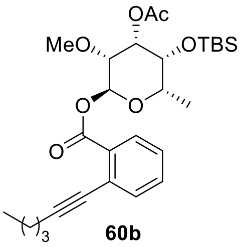

60b

Semi-acetal **58b** (52.8 mg, 158 µmol, 1.00 eq.) in dry CH_2Cl_2 (1.2 mL) was treated with acid **59** (41.0 mg, 190 µmol, 1.20 eq.), DMAP (28.9 mg, 237 µmol, 1.50 eq.) and DCC (48.9 mg, 237 µmol, 1.50 eq.) at room temperature. The reaction mixture was stirred for 3.5 h and quenched by addition of sat. aq. $NaHCO_3$ solution. The aqueous phase was extracted with CH_2Cl_2 thrice and combined organic phases were dried over Na_2SO_4. After removal of all volatiles and purification by column chromatography (SiO_2, pentane/EtOAc 11:1) glycoside **60b** (59.1 mg, 72%, α:β 10:1) was isolated as a light-yellow oil. **R_f** = 0.80 (hexanes/EtOAc 3:1); $[\boldsymbol{\alpha}]_D^{20}$ −60.9° (c 1.0 in $CHCl_3$); **IR** ν_{max}/cm^{-1} 2931 (m), 2854 (m), 1744 (s), 1276 (s), 1260 (s), 1136 (m), 1081 (m), 838 (m), 762 (s), 750 (s); α-anomer **^{1}H-NMR** (500 MHz, $CDCl_3$) δ 7.90 (dd, 1H, J = 1.3, 8.1 Hz), 7.53 (d, 1H, J = 7.8 Hz), 7.44 (dt, 1H, J = 1.3, 7.5 Hz), 7.32 (dt, 1H, J = 1.3 Hz, 7.5 Hz), 6.47 (d, 1H, J = 2.7 Hz), 5.23 (t, 1H, J = 3.5 Hz), 4.20 (dq, 1H, J = 2.2, 6.7 Hz), 3.89 (t, 1H, J = 2.7 Hz), 3.54 (t, 1H, J = 3.2 Hz), 3.45 (s, 3H), 2.46 (dt, 2H, J = 3.3, 7.2 Hz), 2.15 (s, 3H), 1.62 (m, 2H), 1.50 (m, 2H), 1.30 (d, 3H, J = 6.7 Hz), 0.95 (s, 9H), 0.95 (t, 3H, J = 7.0 Hz), 0.10 (s, 3H), 0.06 (s, 3H) ppm; β-anomer significant signals **^{1}H-NMR** (500 MHz, $CDCl_3$) δ 8.23 (d, 1H, J =7.8 Hz), 7.67 (dt, 1H, J = 1.5, 7.8 Hz), 7.45 (m, 1H), 7.34 (m, 1H), 6.23 (s, 1H), 2.55-2.39 (m, 2H), 1.72-1.37 (m, 4H) ppm ; α-anomer **^{13}C-NMR** (125 MHz, $CDCl_3$) δ 170.5, 164.5, 135.0, 132.1, 130.9, 130.6, 127.3, 125.1, 96.7, 92.9, 79.6, 76.3, 70.9, 70.8, 69.9, 59.7, 30.9, 26.0, 22.2, 21.5, 19.7, 18.6, 16.9, 13.8,

−4.34, −4.48 ppm; **HRMS** ESI *m/z* [M + Na]$^+$ calcd. for $C_{28}H_{42}O_7SiNa$ 541.25868, found 541.25920.

(2*S*,3*R*,4*S*,5*R*)-3-((*tert*-Butyldimethylsilyl)oxy)-6-((*S*,*Z*)-3-(cyclohexyl(hydroxy)-methylene)-5-isopropyl-2,4-dioxopyrrolidin-1-yl)-5-methoxy-2-methyltetrahydro-2*H*-pyran-4-yl acetate (62b)

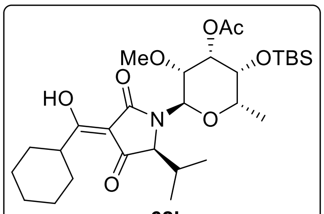
62b

Glycoside **60b** (200 mg, 386 µmol, 1.00 eq.) and tetramic acid **61** (145 mg, 578 µmol, 1.50 eq.) were dissolved in toluene and the solvent was removed on a rotary evaporator. This procedure was repeated twice. The substances were dissolved in dry toluene (1 mL) and treated with $AuPPh_3NTf_2$ (57.0 mg, 77.1 µmol, 0.20 eq.) at room temperature. After stirring for 20 h at 40 °C the volatiles were removed *in vacuo* and crude product was purified by column chromatography (SiO_2 C-18, 40% MeCN in H_2O + 0.1% HCOOH→60% MeCN in H_2O + 0.1% HCOOH→80% MeCN in H_2O + 0.1% HCOOH→90% MeCN in H_2O + 0.1% HCOOH). The product **62b** (110 mg, 50%, α:β >30:1) was isolated as a light-yellow solid. **R$_f$** = 0.40 (hexanes/EtOAc 3:1); **mp** 88 °C; $[\boldsymbol{\alpha}]_D^{20}$ −44.6° (c 1.0 in $CHCl_3$); **IR** ν_{max}/cm^{-1} 2991 (w), 2931 (m), 2858 (m), 1748 (m), 1705 (m), 1652 (m), 1607 (m), 1452 (m), 1361 (w), 1276 (m), 1260 (m), 1231 (m), 1106 (m), 1987 (m), 1007 (w), 963 (m), 863 (m), 838 (m), 764 (s), 751 (s); α-anomer **^{1}H-NMR** (500 MHz, CD_3OD) *δ* 5.72 (t, 1H, *J* = 3.3 Hz), 5.06 (br. s, 1H), 4.26 (br. s, 1H), 4.10 (m, 2H), 3.85 (br. s, 1H), 3.45 (m, 1H), 3.30 (s, 3H, under solvent signal), 2.23 (m, 1H), 2.12 (s, 3H), 1.86-1.69 (m, 5H), 1.50 (m, 2H), 1.41 (d, 3H, *J* = 6.8 Hz), 1.44-1.23 (m, 3H), 1.17 (d, 3H, *J* = 6.9 Hz), 0.89 (s, 9H), 0.89 (d, 3H, *J* = 6.9 Hz), 0.13 (s, 3H), 0.11 (s, 3H) ppm; α-anomer major tautomer **^{13}C-NMR** (125 MHz, $CDCl_3$) *δ* 193.9, 192.6, 175.9, 170.2, 104.9, 101.3, 75.4, 74.0, 73.7, 71.5, 69.8, 67.7, 57.1, 41.0, 30.3, 29.0, 28.5, 25.8, 25.7, 25.6, 18.1, 18.0, 16.0, 13.1, −4.89, −4.98 ppm; significant signals α-anomer minor tautomer **^{13}C-NMR** (125 MHz, $CDCl_3$) *δ* 199.7, 197.6, 170.2, 75.9, 73.5, 73.0, 71.3, 70.1, 67.8, 57.0, 41.8, 30.2, 29.1, 28.4, 25.8, 25.7, 21.4, 18.2, 15.7, 13.1, −4.90, −4.98 ppm; **HRMS** ESI *m/z* [M + H]$^+$ calcd. for $C_{29}H_{50}NO_8Si$ 568.33002, found 568.32990.

2.9 Synthesis of 3-acyltetramic acid **61**

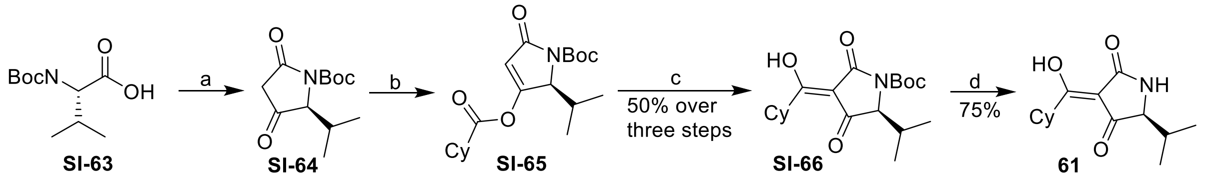

Scheme S12. Synthesis of 3-acyltetramic acid **61**.
Reagents and conditions: a) 1. Meldrum´s acid, DMAP, EDC·HCl, CH_2Cl_2, rt, 3 h, 2. EtOAc, Δ, 2 h; b) 1. cyclohexylcarbonic acid, EDC·HCl, DMAP, CH_2Cl_2, 0 °C, 50 min, 2. tetramic acid **SI-64**, rt, 2.5 h; c) NEt_3, DMAP, CH_2Cl_2, rt, 2 d; d) TFA, CH_2Cl_2, rt, 20 min.

***tert*-Butyl(*S*,*Z*)-3-(cyclohexyl(hydroxy)methylene)-5-isopropyl-2,4-dioxopyrrolidine-1-carboxylate (SI-66)**

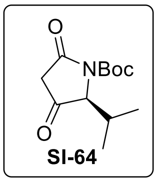

Amino acid **SI-63** (5.00 g, 23.0 mmol, 1.00 eq.) in dry CH_2Cl_2 (74 mL) was treated with Meldrum´s acid (3.65 g, 25.3 mmol, 1.10 eq.), DMAP (3.93 g, 32.2 mmol, 1.40 eq.) and EDC·HCl (5.29 g, 27.6 mmol, 1.20 eq.). at room temperature. The reaction mixture was stirred for 3 h. 0.5M H_2SO_4 and EtOAc were added. The organic phase was separated, and the aqueous phase was extracted thrice with EtOAc. Combined organic phases were washed with H_2O and dried over Na_2SO_4. After filtration, organic phase was stirred under reflux for 2 h. The solvent was removed under reduced pressure. The product **SI-64** was used without further purification.

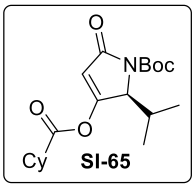

Cyclohexylcarbonic acid (2.58 mL, 20.9 mmol, 1.00 eq.) in dry CH_2Cl_2 (70 mL) was treated with EDC·HCl (4.79 g, 25.0 mmol, 1.20 eq.) and DMAP (511 mg, 4.18 mmol, 0.20 eq.) at 0 °C. After 50 min at room temperature, tetramic acid **SI-64** (5.55 g, 23.0 mmol, 1.10 eq.) in dry CH_2Cl_2 (55 mL) was added. Stirring was continued for 2.5 h. Addition of CH_2Cl_2 and 0.5M H_2SO_4 was followed by separation of organic phase. The aqueous phase was extracted thrice with CH_2Cl_2, combined organic phases were washed with brine, dried over Na_2SO_4 and volatiles were removed under reduced pressure. Purification over a short SiO_2-plug (SiO_2, pentane/EtOAc 20:1 →10:1→7:1→5:1) led to 4-*O*-acyl tetramic acid **SI-65** (6.65 g). It was pure enough for the next step. **R**$_f$ = 0.92 (hexanes/EtOAc 3:1); **^{1}H-NMR** (500 MHz, CD_3OD) δ 6.10 (d, 1H, J = 0.7 Hz), 4.49 (dd, 1H, J = 0.7, 2.4 Hz), 2.49 (m, 2H), 1.99 (m, 2H), 1.79 (m, 2H), 1.67 (m, 1H), 1.54 (s, 9H), 1.51 (m, 1H), 1.32 (m, 4H), 1.12 (d, 3H, J = 6.8 Hz), 0.82 (d, 3H, J = 6.8Hz) ppm; **HRMS** ESI *m/z* $[M + Na]^+$ calcd. for $C_{19}H_{29}NO_5Na$ 374.19375, found 374.19308.

4-*O*-Acyltetramic acid **SI-65** (6.65 g, 18.8 mmol, 1.00 eq.) in dry CH_2Cl_2 (190 mL) was treated with dry NEt_3 (3.20 mL, 22.6 mmol, 1.20 eq.) and DMAP (1.15 g, 9.40 mmol, 0.50 eq.) at room temperature. After stirring for 22 h DMAP (575 mg, 4.70 mmol, 0.25 eq.) was added again and stirring was continued for 24 h. Sat. aq. $NaHCO_3$ solution and CH_2Cl_2 were added. The aqueous phase was extracted thrice with CH_2Cl_2, combined organic phases were washed with brine and dried over Na_2SO_4. Removal of all volatiles under reduced pressure and purification by column chromatography (SiO_2 C-18, 40% MeCN in H_2O + 0.1% HCO_2H→60% MeCN in H_2O + 0.1% HCO_2H→80% MeCN in H_2O + 0.1% HCO_2H→100% MeCN in H_2O + 0.1% HCO_2H) gave 3-acyl tetramic acid **SI-66** as an orange resin (4.04 g, 50% over three steps). $\mathbf{R_f}$ = 0.72 (CH_2Cl_2/MeOH 9:1); $[\alpha]_D^{20}$ +37.2° (c 1.0 in $CHCl_3$); **IR** ν_{max}/cm^{-1} 2970 (m), 2933 (m), 2857 (m), 1771 (m), 1744 (m), 1713 (s), 1652 (m), 1599 (s), 1452 (m), 1393 (m), 1228 (m), 1308 (s), 1277 (s), 1259 (s), 1154 (s), 1022 (w), 931 (m), 913 (m), 857 (w), 764 (s), 751 (s); **^{1}H-NMR** (500 MHz, CD_3OD) δ 4.33 (s, 1H), 3.46 (tt, 1H, J = 3.0, 11.5 Hz), 2.45 (dqn, 1H, J = 3.0, 7.1 Hz), 1.84 (m, 4H), 1.75 (m, 1H), 1.55 (s, 9H), 1.48 (dt, 2H, J = 2.9, 12.1 Hz), 1.40 (m, 2H), 1.28 (m, 1H), 1.17 (d, 3H, J = 7.1Hz), 0.82 (d, 3H, J = 7.1 Hz); mixture of three tautomers **^{13}C-NMR** (125 MHz, $CDCl_3$) δ 201.2, 197.7, 195.4, 192.4, 174.5, 165.8, 165.0, 163.3, 149.7, 149.0, 117.3, 104.5, 101.4, 84.0, 83.5, 83.3, 69.1, 65.6, 61.8, 42.7, 41.3, 30.8, 30.3, 29.2, 28.8, 28.6, 28.4, 28.3, 28.1, 26.0, 25.8, 25.7, 25.6, 25.5, 19.0, 18.6, 18.5, 16.2, 15.7, 15.1 ppm; **HRMS** ESI *m/z* [M + Na]$^+$ calcd. for $C_{19}H_{29}NO_5Na$ 374.19379, found 374.19296.

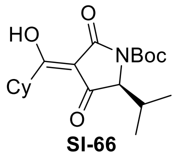

SI-66

(*S*,*Z*)-3-(Cyclohexyl(hydroxy)methylene)-5-isopropylpyrrolidine-2,4-dione (61)

Tetramic acid **SI-66** (606 mg, 1.71 mmol, 1.00 eq.) was dissolved in dry CH_2Cl_2 (32 mL) and treated with TFA (3.20 mL, 10 vol% CH_2Cl_2) at room temperature. The solution was stirred for 20 min. All volatiles were removed at the rotary evaporator. The crude product was purified by column chromatography (SiO_2 C-18, 40% MeCN in H_2O + 0.1% HCO_2H→50% MeCN in H_2O + 0.1% HCO_2H→60% MeCN in H_2O + 0.1% HCO_2H→80% MeCN in H_2O + 0.1% HCO_2H→100% MeCN in H_2O + 0.1% HCO_2H) to afford product **61** as a light orange solid (323 mg, 75%). $\mathbf{R_f}$ = 0.68 (CH_2Cl_2/MeOH 9:1); **mp** 109 °C; $[\alpha]_D^{20}$ −109.3° (c 1.0 in $CHCl_3$); **IR** ν_{max}/cm^{-1} 3219 (m), 2931 (m), 2856 (m), 1653 (s), 1606 (s), 1448 (m), 1352 (m), 1308 (m), 1276 (m), 1261 (m), 1227 (m), 1137 (w), 1024 (w), 920 (m), 817 (m), 765 (s), 750 (s); **^{1}H-NMR** (500 MHz, CD_3OD) δ 3.75 (br. s, 1H), 3.40 (br. s, 1H), 2.17 (m, 1H), 1.86-1.70 (m, 4H), 1.56-1.20 (m,

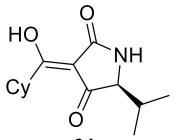

61

6H), 1.03 (d, 3H, J = 7.1 Hz), 0.82 (d, 3H, J = 7.1 Hz) ppm; major tautomer **^{13}C-NMR** (125 MHz, $CDCl_3$) δ 196.6, 192.6, 176.7, 100.4, 67.3, 41.0, 30.26, 28.9, 28.6, 25.74, 25.67, 25.61, 19.6, 16.0 ppm; minor tautomer **^{13}C-NMR** (125 MHz, $CDCl_3$) δ 201.5, 194.9, 169.5, 103.8, 64.0, 41.6, 30.30, 28.9, 28.8, 25.8, 25.61, 25.56, 19.3, 16.3 ppm; **HRMS** ESI *m/z* $[M + H]^+$ calcd. for $C_{14}H_{22}NO_3$ 252.15942, found 252.15883.

2.10 Synthesis of acid **59**

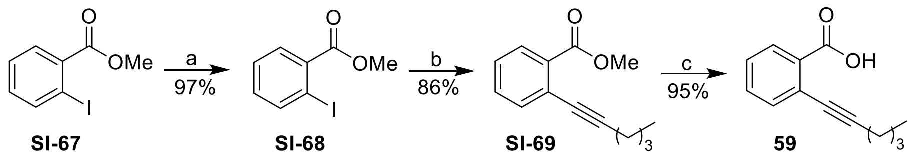

Scheme S13. Synthesis of acid **59**.
Reagents and conditions: a) $SOCl_2$, MeOH, −10 °C→40 °C, 17 h; b) 1. $PdCl_2(PPh_3)_2$, PPh_3, CuI, *i*Pr_2NH, rt, 1 h, 2. 1-hexyne, 0 °C→rt, 18.5 h; c) NaOH, THF, 50 °C, 19 h.

Methyl 2-iodobenzoate (SI-68)

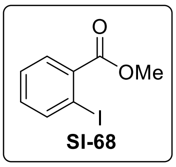

2-Iodobenzoic acid **SI-67** (5.00 g, 20.2 mmol, 1.00 eq.) was dissolved in dry MeOH (35.0 mL) and $SOCl_2$ (2.20 mL, 30.2 mmol, 1.20 eq.) was slowly added at −10 °C. After 15 min the solution was heated to 40 °C and stirred for a further 17 h. The reaction was quenched by addition of sat. aq. $NaHCO_3$ solution and EtOAc. The organic phase was separated, and the aqueous phase was extracted with EtOAc thrice, combined organic phases were washed with H_2O twice and dried over Na_2SO_4. The solvents were removed *in vacuo*. Purification by column chromatography (SiO_2, pentane/EtOAc 6:1) afforded product **SI-68** (5.11 g, 97%) as a colourless liquid. **R_f** = 0.70 (hexanes/EtOAc 4:1); **IR** ν_{max}/cm^{-1} 2950 (m), 1727 (s), 1583 (m), 1562 (w), 1465 (m), 1432 (s), 1289 (s), 1251 (s), 1191 (m),1131 (s), 1104 (s), 1043 (m), 1016 (s), 963 (m), 826 (w), 739 (s), 688 (m); **^{1}H-NMR** (500 MHz, $CDCl_3$) δ 7.99 (d, 1H, J = 7.9 Hz), 7.80 (dd, 1H, J = 1.5, 7.9 Hz), 7.40 (t, 1H, J = 7.7 Hz), 7.15 (t, 1H, J = 7.7 Hz), 3.93 (s, 3H) ppm.

Spectroscopic data corresponded to those reported in the literature.[14]

Methyl 2-(hex-1-yn-1-yl)benzoate (SI-69)

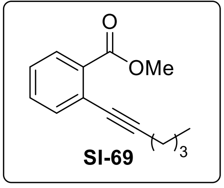

Methyl 2-iodobenzoate (**SI-68**, 100 mg, 382 µmol, 1.00 eq.) was dissolved in *i*Pr_2NH (1.00 mL) and treated with $PdCl_2(PPh_3)_2$ (13.4 mg, 19.1 µmol, 5 mol%), PPh_3 (10.0 mg, 38.2 µmol, 10 mol%) and CuI (3.63 mg, 19.1 µmol, 5 mol%). The mixture was stirred at room temperature for 1h. At 0 °C, hexyne (65.7 µL, 572 µmol, 1.50 eq.) was added, stirring was continued for a further 18.5 h and the mixture was allowed to warm to room temperature. Addition of sat. aq. NH_4Cl solution stopped the reaction. Pentane was added and the organic phase was separated. The aqueous phase was extracted with pentane/EtOAc 100:1 and the combined organic phases were washed with H_2O and brine. They were dried over Na_2SO_4 and all volatiles were removed under reduced pressure. The crude product was purified by column chromatography (SiO_2, pentane/EtOAc 30:1→20:1) to give alkyne **SI-69** as a colourless liquid (76.0 mg, 86%). $\mathbf{R_f}$ = 0.79 (hexanes/EtOAc 9:1); **IR** ν_{max}/cm^{-1} 2956 (m), 2934 (m), 2873 (m), 1733 (s), 1718 (s), 1597 (w), 1577 (w), 1485 (m), 1447 (m), 1433 (m), 1294 (s), 1276 (s), 1249 (s), 1190 (w), 1129 (m), 1083 (s), 1043 (w), 966 (w), 757 (s), 702 (m); **^{1}H-NMR** (500 MHz, $CDCl_3$) δ 7.88 (dd, 1H, J = 1.1, 7.9 Hz), 7.51 (dd, 1H, J = 1.1, 7.9 Hz), 7.42 (dt, 1H, J = 1.4, 7.6 Hz), 7.31 (t, 1H, J = 1.4, 7.6 Hz), 3.91 (s, 3H), 2.48 (t, 2H, J = 7.1 Hz), 1.62 (m, 2H), 1.51 (m, 2H), 0.96 (t, 3H, J = 7.3 Hz) ppm.

Spectroscopic data corresponded to those reported in the literature.[15]

2-(Hex-1-yn-1-yl)benzoic acid (59)

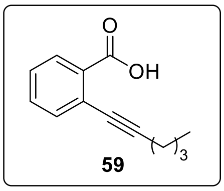

Ester **SI-69** (76.0 mg, 330 µmol, 1.00 eq.) in THF *p.a.* (1.40 mL) and 1M NaOH (1.40 mL) was stirred at 50 °C for 19 h. The solution was treated with conc. HCl until pH value reached 1. The aqueous phase was extracted five times with CH_2Cl_2. The combined organic phases were dried over Na_2SO_4, and the volatiles were removed under reduced pressure. Product **59** (68.2 mg, 95%) was isolated as a colourless resin and used without further purification. $\mathbf{R_f}$ = 0.23 (hexanes/EtOAc 9:1); **IR** ν_{max}/cm^{-1} 3077 (m), 2958 (m), 2932 (m), 2873 (m), 2656 (m), 2229 (w), 1693 (s), 1600 (w), 1568 (w), 1487 (w), 1455 (w), 1409 (m), 1379 (w), 1297 (m), 1274 (m), 1141 (w),1086 (w), 922 (w), 756 (m); **^{1}H-NMR** (500 MHz, $CDCl_3$) δ 8.11 (d, 1H, 7.7 Hz), 7.51 (dd, 1H, J = 1.2, 7.7 Hz), 7.42 (dt, 1H, J = 1.2, 7.7 Hz), 7.31 (t, 1H, J = 1.2, 7.7 Hz), 2.48

(t, 2H, J = 7.1 Hz), 1.62 (m, 2H), 1.51 (m, 2H), 0.96 (t, 3H, J = 7.3 Hz) ppm. COO*H* not detectable.

Spectroscopic data corresponded to those reported in the literature.[15]

2.11 Synthesis of glycoside **65** for formal synthesis

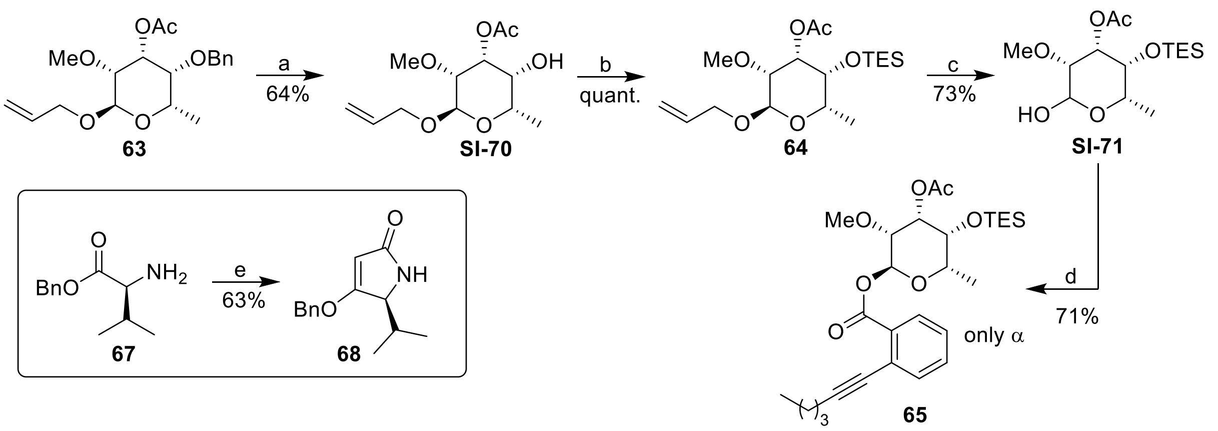

Scheme S14. Synthesis of glycoside **65** for formal synthesis.
Reagents and conditions: a) 1. I_2, CH_2Cl_2, −65 °C, 35 min, 2. Et_3SiH, −65 °C→−20 °C, 2 h; b) TESOTf, pyridine, CH_2Cl_2, 0 °C, 2 h; c) 1. DABCO, Wilkinson´s catalyst, EtOH, Δ, 5 h, 2. I_2, phosphate buffer/H_2O/EtOAc, rt, 25 min; d) DCC, DMAP, CH_2Cl_2, rt, 3 h; e) Ph_3PCCO (**66**), benzoic acid, THF, 60 °C, 22 h.

(3*R*,4*R*,5*R*,6*S*)-2-(Allyloxy)-5-hydroxy-3-methoxy-6-methyltetrahydro-2*H*-pyran-4-yl acetate (SI-70)

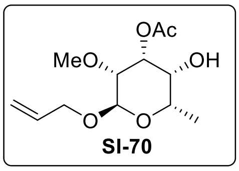

Glycoside **63** (141 mg, 402 µmol, 1.00 eq.) in dry CH_2Cl_2 (10.9 mL) was treated with I_2 (153 mg, 604 µmol, 1.50 eq.) at −65 °C. The mixture was stirred for 35 min and Et_3SiH (96.4 µL, 604 µmol, 1.50 eq.) was added. After 40 min at −65 °C, the solution was allowed to warm to −20 °C. Stirring was continued for 1 h 30 min. Allylic alcohol (136 µL, 2.01 mmol, 5.00 eq.) and $NaHCO_3$ (169 mg, 2.01 mmol, 5.00 eq.) were added. After stirring for 10 min, the mixture was treated with sat. aq. $Na_2S_2O_3$ solution and CH_2Cl_2. The organic phase was separated, and the aqueous phase was extracted with CH_2Cl_2 twice. The combined organic phases were washed with brine and dried over Na_2SO_4. After removal of the volatiles and purification by column chromatography (SiO_2, pentane/EtOAc 3:1) product **SI-70** (67.0 mg, 64%) was obtained as a colourless liquid. **R$_f$** = 0.63 (hexanes/EtOAc 4:1); $[\alpha]_D^{20}$ −110.3° (c 1.0 in $CHCl_3$); **IR** ν_{max}/cm^{-1} 3510 (m), 2987 (m), 2938 (m), 1744 (m), 1429 (m), 1375 (m), 127 (m), 1237 (s), 1178 (w), 1114 (s), 1984 (m), 1045 (s), 981 (m), 933 (w), 764 (s), 750 (s),687 (w); **^{1}H-NMR** (500 MHz, $CDCl_3$) δ 5.90 (dddd, 1H, J = 5.2, 6.1, 10.4, 17.3 Hz), 5.30 (dq, 1H, J = 1.5, 17.3 Hz), 5.21 (dq, 1H, J = 1.5, 10.4 Hz), 5.07 (t, 1H, J = 3.3 Hz), 4.97 (d, 1H, J = 0.8 Hz), 4.20 (ddt, 1H, J = 1.4, 5.2, 12.8 Hz), 4.01 (ddt, 1H, J = 1.4, 6.1, 12.8 Hz), 3.94 (d, 1H, J = 6.6 Hz), 3.70 (m, 1H), 3.55

(m, 1H), 3.52 (s, 3H), 3.38 (d, 1H, J = 11.0 Hz), 2.13 (s, 3H), 1.29 (d, 3H, J = 6.6 Hz) ppm; **^{13}CNMR** (125 MHz, $CDCl_3$) δ 170.5, 133.7, 117.8, 96.7, 78.7, 71.0, 69.9, 68.3, 67.5, 59.8, 21.3, 16.4 ppm; **HRMS** ESI m/z $[M + Na]^+$ calcd. for $C_{12}H_{20}O_6Na$ 283.11521, found 283.11435.

(3*R*,4*S*,5*R*,6*S*)-2-(Allyloxy)-3-methoxy-6-methyl-5-((triethylsilyl)oxy)tetrahydro-2*H*-pyran-4-yl acetate (64)

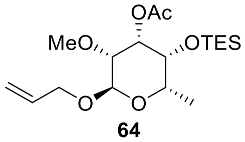

64

Carbohydrate **SI-70** (30.0 mg, 115 µmol, 1.00 eq.) in dry CH_2Cl_2 (2.30 mL) was treated with pyridine (576 µL, 231 µL, 5.00 eq.) and TESOTf (52.1 µL, 231 µL, 2.00 eq.) at 0 °C. After stirring at this temperature for 2 h, sat. aq. $NaHCO_3$ solution and CH_2Cl_2 were added. The aqueous phase was extracted with CH_2Cl_2 thrice and the combined organic phases were dried over Na_2SO_4. After removal of the volatiles under reduced pressure and purification by column chromatography (SiO_2, pentane/EtOAc 4:1) product **64** (43.1 mg, quant.) was isolated as a colourless liquid. **R$_f$** = 0.59 (hexanes/EtOAc 3:1); $[\boldsymbol{\alpha}]_D^{20}$ −77.5° (c 1.0 in $CHCl_3$); **IR** ν_{max}/cm^{-1} 2854 (m), 2878 (m), 1744 (s), 1459 (m), 1413 (w), 1374 (m), 1235 (s), 1197 (m), 1128 (m), 1090 (s), 1052 (s), 1031 (s), 1003 (s), 962 (m), 848 (m), 747 (s), 724 (s), 677 (m); **^{1}H-NMR** (500 MHz, $CDCl_3$) δ 5.90 (dddd, 1H, J = 5.3, 6.0, 10.5, 16.9 Hz), 5.29 (dq, 1H, J = 1.6, 16.9 Hz), 5.18 (dq, 1H, J = 1.6, 10.5 Hz), 5.10 (t, 1H, J = 3.5 Hz), 4.94 (d, 1H, J = 2.5 Hz), 4.17 (ddt, 1H, J = 1.5, 5.3, 12.9 Hz), 4.00 (ddt, J = 1.5, 6.0, 12.9 Hz), 3.95 (dq, 1H, J = 2.1, 6.6 Hz), 3.80 (m, 1H), 3.42 (s, 3H), 3.39 (m, 1H), 2.14 (s, 3H), 1.26 (d, 3H, J = 6.6 Hz), 0.98 (t, 9H, J = 7.9 Hz), 0.65 (q, 6H, J = 7.9 Hz) ppm; **^{13}C-NMR** (125 MHz, $CDCl_3$) δ 170.5, 134.1, 117.4, 97.3, 77.5, 71.2, 70.3, 68.3, 67.9, 59.8, 21.4, 16.5, 7.07, 5.14 ppm; **HRMS** ESI m/z $[M + H]^+$ calcd. for $C_{18}H_{35}O_6Si$ 375.21930, found 375.21974.

(3*R*,4*S*,5*R*,6*S*)-2-Hydroxy-3-methoxy-6-methyl-5-((triethylsilyl)oxy)tetrahydro-2*H*-pyran-4-yl acetate (SI-71)

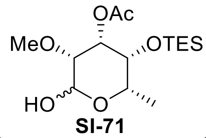

SI-71

Glycoside **64** (168 mg, 449 µmol, 1.00 eq.) was dissolved in EtOH *p.a.* (3.00 mL) and treated with Wilkinson catalyst (4.15 mg, 44.9 µmol, 1 mol%) as well as DABCO (7.55 mg, 67.3 µmol, 15 mol%). The suspension was stirred at 95 °C for 24 h. Rhodium-catalyst (4.15 mg, 44.9 µmol, 1 mol%) and DABCO (7.55 mg, 67.3 µmol, 15 mol%) were added again at room temperature. Stirring was continued for 24 h at 95 °C. A third portion of Wilkinson catalyst (4.15 mg, 44.9 µmol,

1 mol%) and DABCO (7.55 mg, 67.3 µmol, 15 mol%) was added. After stirring for a further 3 days the mixture was filtered off over celite® and the volatiles were removed under reduced pressure. The crude product was dissolved in EtOAc (48 mL) and H_2O (48 mL). A buffer (pH=7, 4.8 mL) was added. The mixture was treated dropwise with a solution of iodine (342 mg, 1.35 mmol, 3.00 eq.) in EtOAc (19 mL). After 25 min, sat. aq. $Na_2S_2O_3$ solution was added. The aqueous phase was extracted with EtOAc thrice, combined organic phases were washed with sat. aq. $NaHCO_3$ solution and dried over Na_2SO_4. Removal of the volatiles *in vacuo* and purification by column chromatography (SiO_2, pentane/EtOAc 2:1→1:1) afforded product **SI-71** (109 mg, 73%) as a colourless resin. $\mathbf{R_f}$ = 0.33 (hexanes/EtOAc 2:1); $[\alpha]_D^{20}$ −64.5° (c 1.0 in $CHCl_3$); **IR** ν_{max}/cm^{-1} 2930 (m), 2900 (m), 2857 (m), 1745 (s), 1463 (w), 1374 (m), 1238 (s), 1130 (m), 1091 (s), 1053 (s), 1004 (m), 940 (w), 859 (m), 838 (m), 765 (s), 750 (s); α-anomer **^{1}H-NMR** (500 MHz, $CDCl_3$) δ 5.27 (t, 1H, J = 2.6 Hz), 5.22 (t, 1H, J = 3.4 Hz), 4.16 (dq, 1H, J = 3.0, 6.7 Hz), 3.82 (t, 1H, J = 3.0 Hz), 3.43 (s, 3H), 3.32 (dt, 1H, J = 0.6, 3.5 Hz), 3.03 (br. s, 1H), 2.13 (s, 3H), 1.69 (br. s, 1H), 1.29 (d, 3H, J = 6.7 Hz), 0.97 (t, 9H, J = 7.9 Hz), 0.66 (q, 6H, J = 7.9 Hz) ppm; β-anomer **^{1}H-NMR** (500 MHz, $CDCl_3$) δ 4.81 (t, 1H, J = 3.2 Hz), 4.67 (dd, 1H, J = 1.6, 12.5 Hz), 4.09 (d, 1H, J = 12.6 Hz), 3.72 (dt, 1H, J = 1.1, 3.2 Hz), 3.55 (s, 3H), 3.55 (dq, 1H, J = 1.4, 6.7 Hz), 3.50 (m, 1H), 2.18 (s, 3H), 1.29 (d, 3H, J = 6.7 Hz), 0.99 (t, 9H, J = 7.9 Hz), 0.66 (q, 6H, J = 7.9 Hz) ppm; α-anomer **^{13}C-NMR** (125 MHz, $CDCl_3$) δ 170.4, 92.2, 78.1, 70.6, 69.9, 69.1, 59.5, 21.3, 16.0, 7.01, 5.05 ppm; β-anomer **^{13}C-NMR** (125 MHz, $CDCl_3$) δ 170.3, 93.8, 73.9, 71.9, 69.7, 61.7, 21.3 17.1, 7.12, 5.22 ppm; **HRMS** ESI *m/z* $[M + Na]^+$ calcd. for $C_{15}H_{30}O_6SiNa$ 357.17039 found 357.16962.

(2*S*,3*R*,4*S*,5*R*,6*S*)-4-Acetoxy-3-methoxy-6-methyl-5-((triethylsilyl)oxy)tetrahydro-2*H*-pyran-2-yl 2-(hex-1-yn-1-yl)benzoate (65)

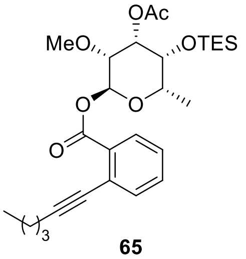

65

Semi-acetal **SI-71** (110 mg, 329 µmol, 1.00 eq.) and acid **59** (85.4 mg, 395 µmol, 1.20 eq.) were dissolved in dry CH_2Cl_2 (1.5 mL) and treated with DCC (102 mg, 493 µmol, 1.50 eq.) as well as DMAP (60.3 mg, 493 µmol, 1.50 eq.) at room temperature. The suspension was stirred for 3 h, before sat. aq. $NaHCO_3$ solution was added. The aqueous phase was extracted with CH_2Cl_2 thrice and the combined organic phases were dried over Na_2SO_4. Removal of the solvent at the rotary evaporator and purification by column chromatography (SiO_2, pentane/EtOAc 6:1→4:1) as well as a second column chromatography (SiO_2, pentane/EtOAc 9:1→8:1) furnished glycoside **65** (122 mg, 71%, single diastereomer) as

a colourless oil. $\mathbf{R_f}$ = 0.80 (hexanes/EtOAc 3:1); $[\alpha]_D^{20}$ −61.7° (c 1.0 in $CHCl_3$); **IR** ν_{max}/cm^{-1} 2956 (m), 2938 (m), 2877 (m), 1744 (m), 1458 (w), 1375 (w), 1276 (s), 1261 (s), 1236 (m), 1136 (m), 1081 (m), 1031 (w), 921 (w), 853 (w), 764 (s), 750 (s); **^{1}H-NMR** (500 MHz, $CDCl_3$) δ 7.90 (dd, 1H, *J* = 1.0, 7.9 Hz), 7.53 (dd, 1H, *J* = 1.0, 7.9 Hz), 7.44 (dt, 1H, 7.8 Hz), 7.32 (dt, 1.2, 7.5 Hz), 6.49 (d, 1H, *J* = 2.3 Hz), 5.18 (t, 1H, *J* = 3.5 Hz), 4.22 (dq, 1H, *J* = 1.7, 6.5 Hz), 3.89 (m, 1H), 3.56 (ddd, 1H, *J* = 0.9, 2.3, 3.5 Hz), 3.50 (s, 3H), 2.46 (dt, 2H, *J* = 3.2, 7.2 Hz), 2.17 (s, 3H), 1.61 (m, 2H), 1.49 (m, 2H), 1.31 (d, 3H, *J* = 6.5 Hz), 1.00 (t, 9H, *J* = 7.9 Hz), 0.95 (t, 3H, *J* = 7.3 Hz), 0.68 (q, 6H, *J* = 7.9 Hz) ppm; **^{13}C-NMR** (125 MHz, $CDCl_3$) δ 170.5, 164.5, 135.0, 132.1, 130.9, 130.7, 127.3, 125.1, 96.7, 93.1, 79.7, 76.3, 70.7, 70.1, 60.0, 30.9, 22.3, 21.4, 19.7, 16.8, 13.8, 7.05, 5.15 ppm; **HRMS** ESI *m/z* [M + Na]$^+$ calcd. for $C_{28}H_{42}O_7SiNa$ 541.25920, found 541.25885.

Spectroscopic data corresponded to those reported in the literature.[2]

(*S*)-4-(Benzyloxy)-5-isopropyl-1,5-dihydro-2*H*-pyrrol-2-one (68)

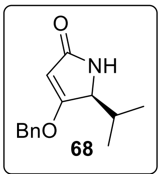

Amino acid **67** (500 mg, 2.41 mmol, 1.00 eq.) in dry THF (8.00 mL) was treated with Ph_3PCCO (**66**, 802 mg, 2.65 mmol, 1.10 eq.) and benzoic acid (58.9 mg, 482 µmol, 0.20 eq.) at room temperature. The mixture was heated to 60 °C and stirred for 22 h. The volatiles were removed under reduced pressure and the crude product was purified by column chromatography (SiO_2, acetone/CH_2Cl_2 19:1→6:1→4:1→3:1 →2:1→1.5:1) to furnish 4-*O*-alkyl tetramic acid **68** (351 mg, 1.52 mmol) as a colourless solid. $\mathbf{R_f}$ = 0.59 (CH_2Cl_2/MeOH 9:1); **mp** 129 °C; **^{1}H-NMR** (500 MHz, $CDCl_3$) δ 7.37 (m, 5H), 6.76 (br. s, 1H), 5.10 (d, 1H, *J* = 1.5 Hz), 4.99 (d, 1H, *J* = 11.6 Hz), 4.94 (d, 1H, *J* = 11.6 Hz), 4.04 (d, 1H, *J* = 3.3 Hz), 2.14 (dqn, 1H, *J* = 3.3, 7.0 Hz), 1.03 (d, 3H, *J* = 7.0 Hz), 0.80 (d, 3H, *J* = 7.0 Hz) ppm; **^{13}C-NMR** (125 MHz, $CDCl_3$) 176.3, 175.2, 135.0, 128.82, 128.78, 127.9, 95.4, 73.2, 63.0, 29.4, 19.6, 15.2 ppm; **HRMS** ESI *m/z* [M + H]$^+$ calcd. for $C_{14}H_{18}NO_2$ 232.13321, found 232.13260.

Spectroscopic data corresponded to those reported in the literature.[2]

2.12 Failed routes to amykitanose

Before the synthesis with an allyl function at the anomeric position was completed, we tried to use a methyl acetal at 1-position. It was introduced with sulfuric acid in MeOH in quantitative yield. Protection of the *syn*-diol furnished carbohydrate **SI-72** in 93% yield. Swern-oxidation in 77% yield and consequent reduction with $NaBH_4$ in 99% yield gave alcohol **SI-73** with inverted stereoconfiguration at 4-position as a single diastereomer. The remaining hydroxyl group was benzylated in 99%. Removal of the acetal with $BiCl_3$ provided a diol, which was regioselectively acetylated at 3-position. This was followed by methylation with $TMSCHN_2$ and HBF_4 (→ **SI-75**). Different acidic conditions were used to cleave the acetal at the anomeric position. However, either the acetyl group was removed too, or no reaction was observed. Therefore, it was switched to the allyl group at the anomeric position. It was also tried, to introduce the carbamate at 4-position. The benzyl group was removed via hydrogenation. The resulting hydroxyl group reacted quickly with trichloroacetylisocyanate to an intermediate, which was converted to carbamate **SI-77** by stirring with SiO_2 in a THF/MeOH mixture.

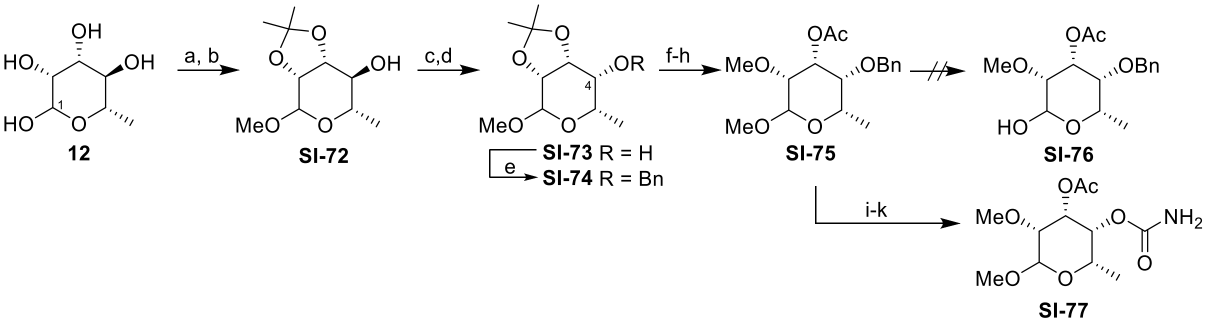

Scheme S15. Performed steps towards methyl-acetal of L-rhamnose **SI-76**.
Reagents and conditions: a) H_2SO_4, MeOH, RT, on, quant.; b) $CuSO_4$, acetone, rt, 21 h, 93%; c) 1. $(ClOC)_2$, DMSO, CH_2Cl_2, −78 °C, 30 min, 2. **SI-72**, 30 min, 3. DIPEA, rt, 18 h, 77%; d) $NaBH_4$, EtOH, 0 C, 21 h, 99%, single diastereomer; e) 1. NaH, imidazole, DMF, 0 °C→rt, 35 min, 2. BnBr, TBAI, rt, 18 h, 99%; f) $BiCl_3$, $MeCN/H_2O$, rt, 1 d, 99%; g) 1. Bu_2SnO, toluene, reflux, 2 h, 2. AcCl, rt, 3 h, 85%; h) $TMSCHN_2$, HBF_4, CH_2Cl_2, 0 °C, 5 h, 77%; i) Pd/C, H_2, MeOH, 20 h, quant.; j) trichloroacetylisocyanate, CH_2Cl_2, 0 °C, 10 min; k) SiO_2, THF/MeOH, 40 °C, 16 h, 65% over two steps.

The main problem of the synthesis of the upper part of kibdelomycin was the coupling of the sugar and tetramic acid. Our first concept was to build *N*-glycosides **SI-79a/b** with L-valine, which we achieved in excellent 99% yield and α:β-ratio of 2:1 by simply adding the amino acid in EtOH or MeOH. However, it was not possible to convert the aminoglycosides **SI-79a/b** into the corresponding β-ketoamides **SI-80a/b**, tetramic acids **SI-81a/b** or 4-*O*-alkyl tetramic acids

SI-82a/b. All of them could be converted to 3-acyltetramic acid in well studied reactions and therefore could have been possible intermediates. For building β-ketoamides **SI-80a/b**, we focused on Ley´s acylation with β-ketothioester **SI-85**. This method was successfully used for acylation of a aminoglycoside by our group in 2016.[16] Different equivalents, reaction time, temperature, different silver salts and additional reagents were tested (Table S1). Most of the times the acetyl group or valine was removed, sometimes complete decomposition was observed or educt was reisolated. Also, an attempt to introduce a β-ketoamide by conversion with adduct **SI-86** under reflux only led to removal of the acetyl group. Likewise, the *in situ* formation of the acid chloride of carboxylic acid **SI-89a** and conversion with aminoglycoside **SI-79a** under basic conditions gave decomposition of starting materials. After multiple attempts, the acetyl group turned out to be instable under different conditions. So instead of the acetyl group, a TBS protecting group was introduced to try some of the reactions already carried out again. Each of them also lead to decomposition or removal of acetyl group or no transformation. Further attempts to convert the aminoglycosides **SI-79a/b** into a tetramic acid via Meldrum´s acid method led to elimination of valine. Also, the conversion with ketenylidentriphenylphosphorane to give 4-*O*-alkyltetramic acids **SI-82a/b** wasn´t successful, only decomposition products were isolated. After trials to convert the aminoglycoside, the β-ketoamide or tetramic acid should be introduced directly. Therefore, a Mitsunobu reaction with β-ketoamide **SI-88** was carried out, but only educt was reisolated. Conversion of semi-acetal **58a** with tetramic acid **SI-87** and *p*TsOH led to decomposition. The experiments with TBS-group instead of acetyl group led to similar results.

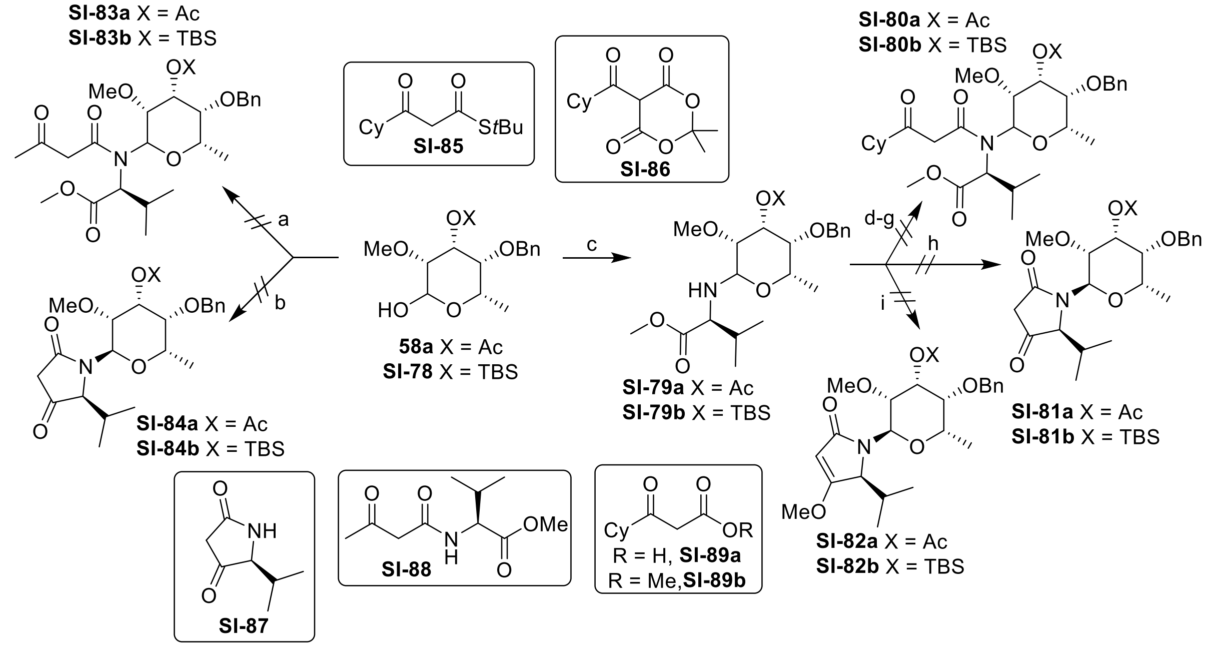

Scheme S16. Failed attempts to attach a tetramic acid or β-ketoamide at the glycoside or aminoglycoside.

Reagents and conditions: a) PPh_3, DIAD, β-ketoamide **SI-88**, THF, −78 °C; b) **SI-87**, *p*TsOH, CH_2Cl_2, reflux, 2 d; c) X = Ac L-valine methyl ester, EtOH/MeOH, rt, 3 d, 99%; X = TBS 86%; d) Table S1 e) X = Ac adduct **SI-86**, toluene, 120 °C, 2 h; f) X = Ac 1. oxalyl chloride, acid **SI-89a**, DMF, 0 °C, 2 h, 2. **SI-79a**, 0 °C, 21 h; g) X = Ac β-ketoester **SI-89b**, toluene, reflux, 22 h; h) X = Ac/TBS 1. Meldrum´s acid, DMAP, EDC·HCl, CH_2Cl_2, rt, 3 h, 2. EtOAc, reflux, 3 h, i) X = Ac/TBS Ph_3PCCO, THF, reflux, 19 h.

Table S1. Reaction conditions for Ley-acylation of aminoglycosides **SI-79a/b**.

**Entry**	**X**	**Reagents and conditions**	**Temperature[°C]**	**Time**	**Result**
1	Ac	Educt (1.00 eq.), **SI-85** (1.25 eq.), AgO_2CCF_3 (1.60 eq.), 4 Å MS, THF, aq. Work-up	0	3 h	Removal of Ac
2	Ac	Educt (1.00 eq.), **SI-85** (1.25 eq.), AgO_2CCF_3 (1.60 eq.), 4 Å MS, THF, without aq. work-up	0	3 h	Removal of Ac/valine
3	Ac	Educt (1.20 eq.), **SI-85** (1.00 eq.), AgO_2CCF_3 (1.20 eq.), NEt_3, THF	0	3 h	Removal of valine
4	Ac	Educt (1.00 eq.), **SI-85** (1.20 eq.), AgO_2CCF_3 (1.50 eq.), NEt_3, THF	0	3 h	Removal of Ac/valine

6	Ac	Educt (1.00 eq.), **SI-85** (1.20 eq.), AgO_2CCF_3 (1.25 eq.), Na_2KHPO_4, THF	0	6 h	Removal of valine
7	Ac	Educt (1.00 eq.), **SI-85** (1.25 eq.), AgO_2CCF_3 (1.25 eq.), 4 Å MS, THF	−78	1 h	Removal of Ac
8	Ac	Educt (1.00 eq.), **SI-85** (1.20 eq.), AgO_2CCF_3 (1.25 eq.), Na_2KHPO_4, THF	−78	1.5 h	Removal of Ac
9	Ac	Educt (1.00 eq.), **SI-85** (1.50 eq.), AgO_3SCF_3 (2.00 eq.), NEt_3, THF	0	6 h	educt
10	Ac	Educt (1.00 eq.), **SI-85** (1.25 eq.), AgO_3SCF_3 (1.60 eq.), 4 Å MS, THF	0	22 h	Removal of Ac/valine
11	TBS	Educt (1.00 eq.), **SI-85** (1.25 eq.), AgO_2CCF_3 (1.25 eq.), 4 Å MS, THF	−78	4 h	Decompo-sition
12	TBS	Educt (1.00 eq.), **SI-85** (1.25 eq. + 1.25 eq.), AgO_2CCF_3 (1.25 eq. + 1.25 eq.), Na_2KHPO_4, THF	−78→rt	1 d	Decompo-sition
13	TBS	Educt (1.20 eq.), **SI-85** (1.00 eq.), AgO_2CCF_3 (1.20 eq.), NEt_3, THF	0→rt	2 d	educt

Some reactions were carried out with activated forms of carbohydrate **58a**. Therefore, it was first acetylated at the anomeric position in 90% yield. The bromide **SI-91** was formed by addition of TMSBr and had to be used directly in the next step because of its instability. On the one hand it was reacted with tetramic acid **SI-87** and KO*t*Bu and on the other hand it was converted with β-ketoamide **SI-88** and KO*t*Bu. Both reactions led to decomposition of starting material. The trichloroacetimidate **SI-92** was easily built by conversion of sugar **58a** with trichloroacetonitrile in 62% yield. Though, the attempts to couple it with tetramic acid **SI-87** or β-ketoamide **SI-88** weren´t successful and led to reisolation of starting material and decomposition, respectively.

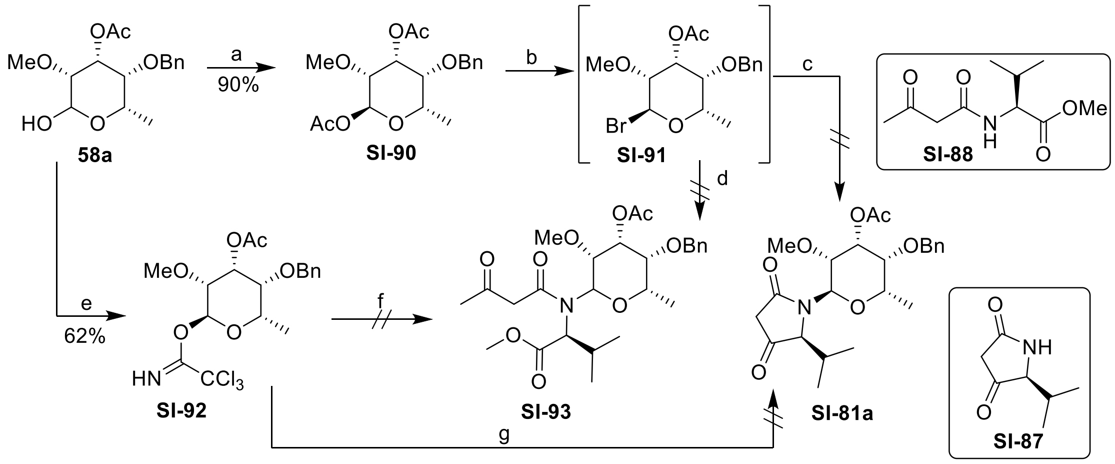

Scheme S17. Failed attempts to attach a tetramic acid or β-ketoamide at activated glycosides **SI-92** and **SI-91**.
Reagents and conditions: a) Ac_2O, pyridine, rt, 2 h; b) TMSBr, CH_2Cl_2, 0 °C, 2 h; c) tetramic acid **SI-87**, KO*t*Bu, THF, 0 °C, 20 h; d) β-ketoamide **SI-88**, KO*t*Bu, THF, 0 °C, 20 h; e) DBU, Cl_3CCN, CH_2Cl_2, 0 °C→rt, 1 d, f) β-ketoamide **SI-88**, TMSOTf, 4 Å MS, CH_3NO_2, rt, 4 d; g) tetramic acid **SI-87**, TMSOTf, 4 Å MS, CH_3NO_2, 0 °C, 1 d.

On the basis of the work of Beretta *et al.*[17] we synthesized the sulfoxide donor **SI-96** in three steps out of L-Rhamnose (**12**) by complete acetylation, *S*-glycosylation and oxidation to the sulfoxide with *m*CPBA. This sugar was used instead of the ready functionalised sugar to try the coupling reactions. Sulfoxide **SI-96** was reacted with tetramic acid **SI-87**, β-ketoamide **SI-88**, 3-acyltetramic acid **61** and 4-*O*-alkyltetramic acid **SI-101**. Before, they were activated by conversion with BSA, which should silylate the nitrogen. Second step is the addition of sugar **SI-96** and a lewis-acid, for which we choose TMSOTf. All the experiments led to decomposition of the starting material.

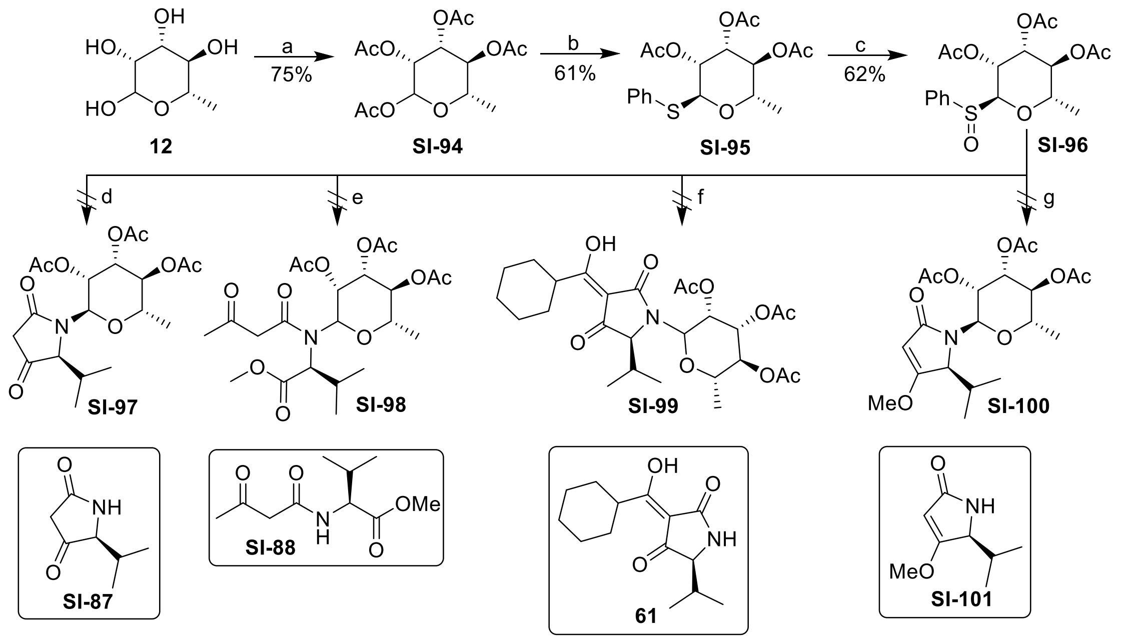

Scheme S18. Failed attempts to attach a tetramic acid or β-ketoamide to sulfoxide **SI-96**.
Reagents and conditions: a) Ac_2O, pyridine, rt, 22 h; b) PhSH, $BF_3{\cdot}OEt_2$, CH_2Cl_2, rt, 22 h; c) *m*CBPA, CH_2Cl_2, −78 °C→0 °C, 7 h; d) 1. tetramic acid **SI-87**, BSA, dichloroethane, 90 °C, 2 h, 2. **SI-96**, TMSOTf, rt, 23 h; e) 1. β-ketoamide **SI-88**, BSA, dichloroethane, 90 °C, 2 h, 2. **SI-96**, TMSOTf, rt, 19 h; f) 1. 3-acyl tetramic acid **61**, BSA, dichloroethane, 90 °C, 1 h, 2. **SI-96**, TMSOTf, rt, 22 h; g) 1. 4-*O*-alkyltetramic acid **SI-101**, BSA, dichloroethane, 90 °C, 2 h, 2. **SI-96**, TMSOTf, rt, 20 h.

Finally, we decided to use the established method of the first total synthesis.[2] Ester **60a** was treated with gold-catalyst and all of the coupling products used before. Conversion with tetramic acid **SI-87** led to a defined product. 2D-NMR-experiments indicated that tetramic acid is bound to the sugar via a *O*-glycosidic linkage. This is possible because of the tautomeric character of tetramic acid **SI-87**. Reaction with β-ketoamide **SI-88** led to a product mixture. Here *O*-, *C*- or *N*-glycosidic linkages are possible. The different products couldn´t be separated. The glycosylation with 4-*O*-alkyltetramic acid **68** as well as 3-acyltetramic acid **61** gave the desired products but with a α:β ratio of 1:1 and 10:1, respectively.

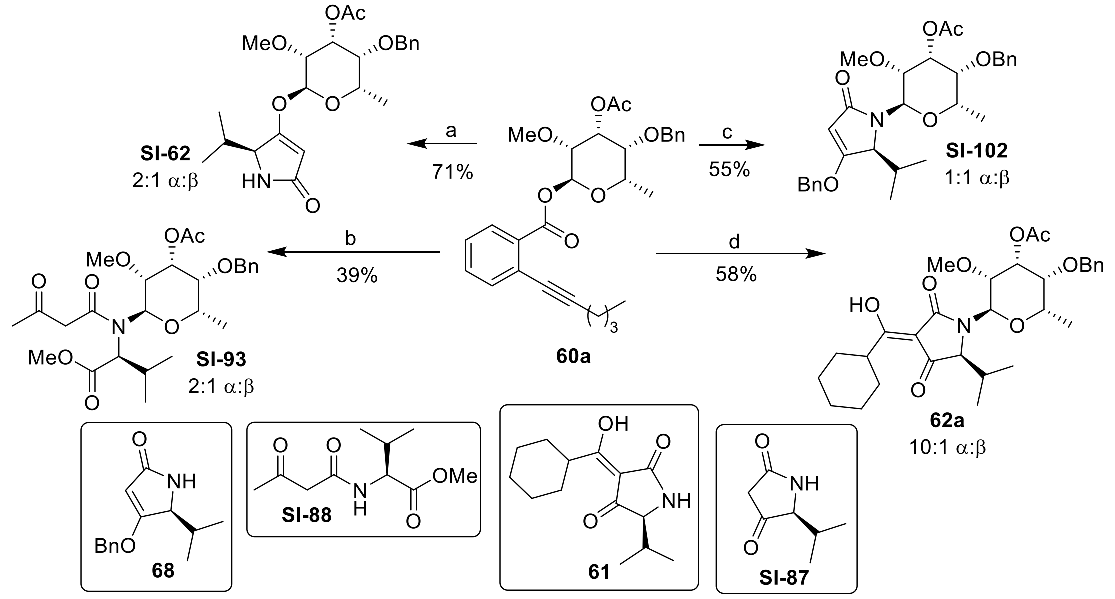

Scheme S19. Investigations on attaching different forms of tetramic acids to a glycoside via an Au-catalysed reaction.
Reagents and conditions: a) tetramic acid **SI-87**, $AuPPh_3NTf_2$, toluene, 40 °C, 20 h; b) β-ketoamide **SI-88**, $AuPPh_3NTf_2$, toluene, 40 °C, 20 h; c) 4-*O*-alkyltetramic acid **68**, $AuPPh_3NTf_2$, toluene, 40 °C, 20 h; d) 3-acyltetramic acid **61**, $AuPPh_3NTf_2$, toluene, 40 °C, 20 h.

2.13 Synthesis of amykitanose (13)

Glycoside **SI-70** was reacted with trichloroacetylisocyanate to give product **SI-103**, which gave the carbamate **SI-104** after stirring with SiO_2 in 91% yield over two steps. Deprotection at the anomeric position in 17% yield gave amykitanose (**13**). The synthesis wasn´t optimised yet but can easily be used to introduce the carbamate function.

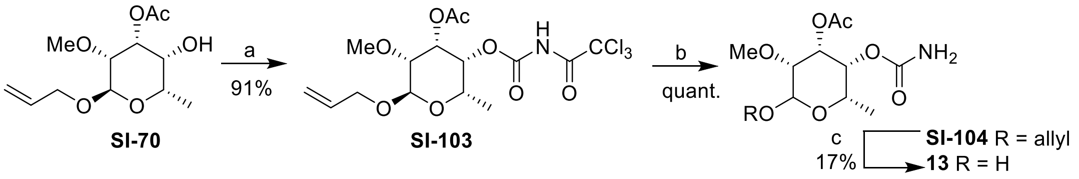

Schema S20. Synthesis of amykitanose (**13**).
Reagents and conditions: a) trichloroacetylisocyanate, CH_2Cl_2, 0°C, 13 min; b) SiO_2, THF/MeOH, 40°C; c) $Pd(PPh_3)_4$, AcOH, rt, 16 h.

2.14 Alternative formal synthesis of kibdelomycin (**1**)

For the completion of an alternative total synthesis exploiting the novel *N*-glycosylation of 3-acyltetramic acids, tetramic acid **SI-87** would have to be attached to the decalin fragment **SI-105** via an established Yoshii-Yoda acylation (Scheme S21).[18] The resulting 3-acyltetramic acid **SI-106** would then be *N*-glycosylated with the sugar fragments **60a/b** via the known Au-catalysed reaction and the 4-position be converted into a carbamic acid to give **SI-107** (analogue to the synthesis of amykitanose (**13**) *cf.* Scheme S20). Finally, building block **SI-107** would be *O*-glycosylated with the amycolose derivative **4** to afford kibdelomycin (**1**).

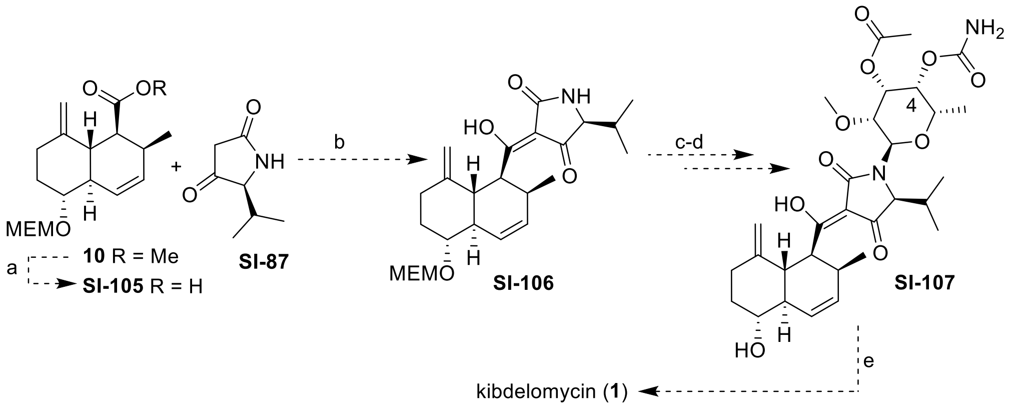

Schema S21. Synthetic plan for an alternative synthesis of kibdelomycin (**1**).
Reagents and conditions: a) LiOH; b) EDC·HCl, DMAP, then NEt_3, DMAP, $CaCl_2$; c) **60a/b**, $AuPPh_3NTf_2$; d) deprotection 4-position, then $Cl_3CCONCO$, then SiO_2, then MEM-deprotection; e) **4**, TfOH.

3. References

1 B. J. L. Royles, *Chem. Rev.*, 1995, **95**, 1981.

2 S. Yang, C. Chen, J. Chen and C. Li, *J. Am. Chem. Soc.*, 2021, **143**, 21258.

3 R. J. Abraham and M. Reid, *J. Chem. Soc., Perkin Trans. 2*, 2002, 1081.

4 A. M. Molins-Pujol, C. Moranta, C. Arroyo, M. T. Rodríguez, M. C. Meca, M. D. Pujol and J. Bonal, *J. Chem. Soc., Perkin Trans. 1*, 1996, 2277.

5 H. Guo and G. A. O'Doherty, *Angew. Chem. Int. Ed.*, 2007, **46**, 5206.

6 T. J. Kucharski, N. Ferralis, A. M. Kolpak, J. O. Zheng, D. G. Nocera and J. C. Grossman, *Nat. Chem.*, 2014, **6**, 441.

7 T. den Hartog, D. van Jan Dijken, A. J. Minnaard and B. L. Feringa, *Tetrahedron Asymmmetry*, 2010, **21**, 1574.

8 H. Sakaguchi, H. Tokuyama and T. Fukuyama, *Org. Lett.*, 2007, **9**, 1635.

9 S. G. Davies, I. A. Hunter, R. L. Nicholson, P. Roberts, E. D. Savory and A. D. Smith, *Tetrahedron*, 2004, **60**, 7553.

10 E. Vedejs, in *Encyclopedia of Reagents for Organic Synthesis*, John Wiley & Sons, Ltd, Chichester, UK, 2001.

11 J. Willwacher and A. Fürstner, *Angew. Chem. Int. Ed.*, 2014, **53**, 4217.

12 T. Hanaya, H. Baba, H. Toyota and H. Yamamoto, *Tetrahedron*, 2009, **65**, 7989.

13 E. Danieli, D. Proietti, G. Brogioni, M. R. Romano, E. Cappelletti, M. Tontini, F. Berti, L. Lay, P. Costantino and R. Adamo, *Bioorg. Med. Chem.*, 2012, **20**, 6403.

14 B. J. Dahl and B. P. Branchaud, *Tetrahedron Lett.*, 2004, **45**, 9599.

15 A. S. K. Hashmi, C. Lothschütz, R. Döpp, M. Ackermann, J. de Buck Becker, M. Rudolph, C. Scholz and F. Rominger, *Adv. Synth. Catal.*, 2012, **354**, 133.

16 M. Petermichl, S. Loscher and R. Schobert, *Angew. Chem. Int. Ed.*, 2016, **55**, 10122.

17 M. Beretta, E. Rouchaud, L. Nicolas, J.-P. Vors, T. Dröge, M. Es-Sayed, J.-M. Beau and S. Norsikian, *Org. Biomol. Chem.*, 2021, **19**, 4285.

18 a) T. Sengoku, J. Wierzejska, M. Takahashi and H. Yoda, *Synlett*, 2010, **2010**, 2944; b) K. Hori, M. Arai, K. Nomura and E. Yoshii, *Chem. Pharm. Bull.*, 1987, **35**, 4368.

4. NMR-Spectra

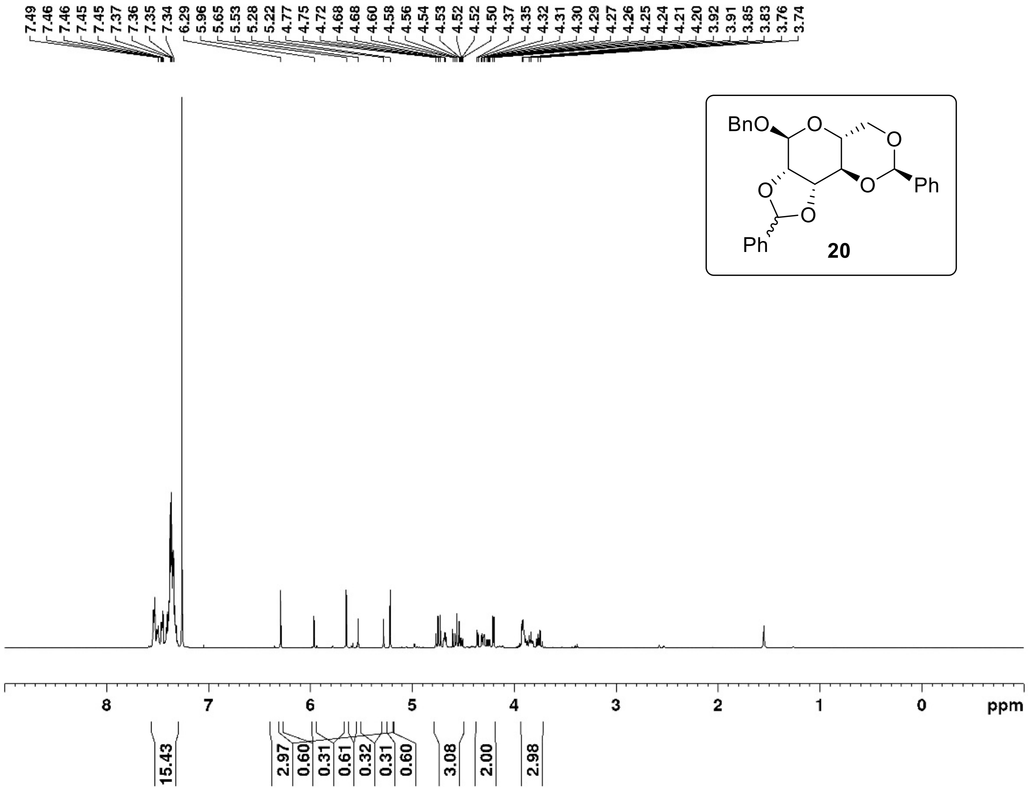

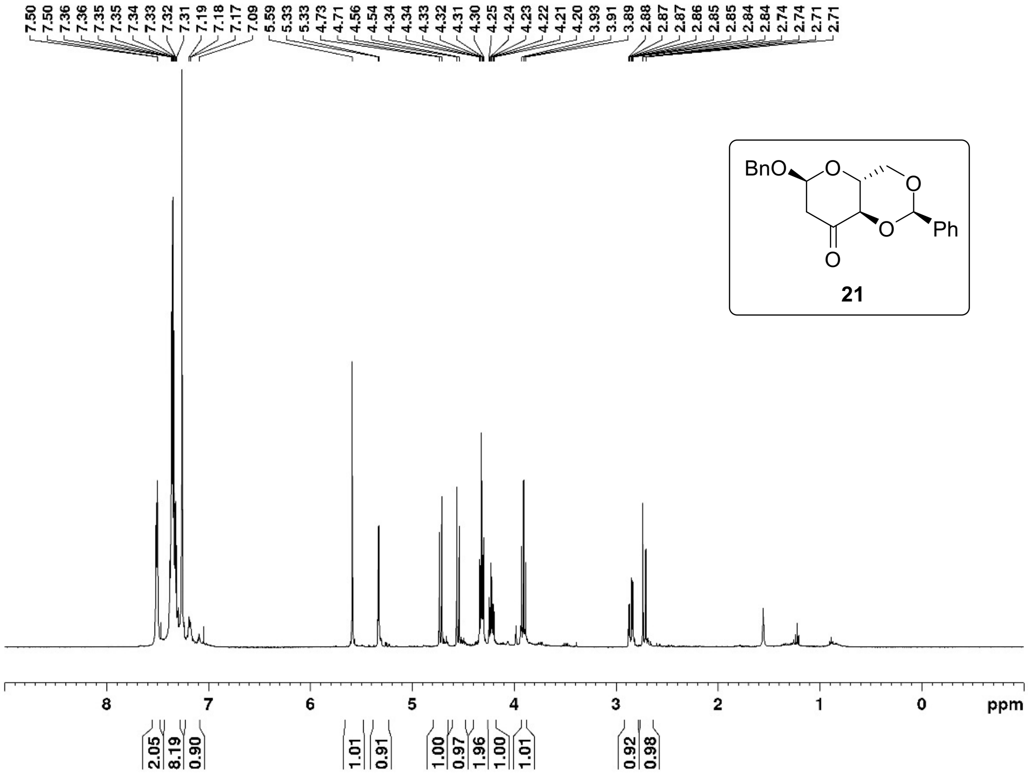

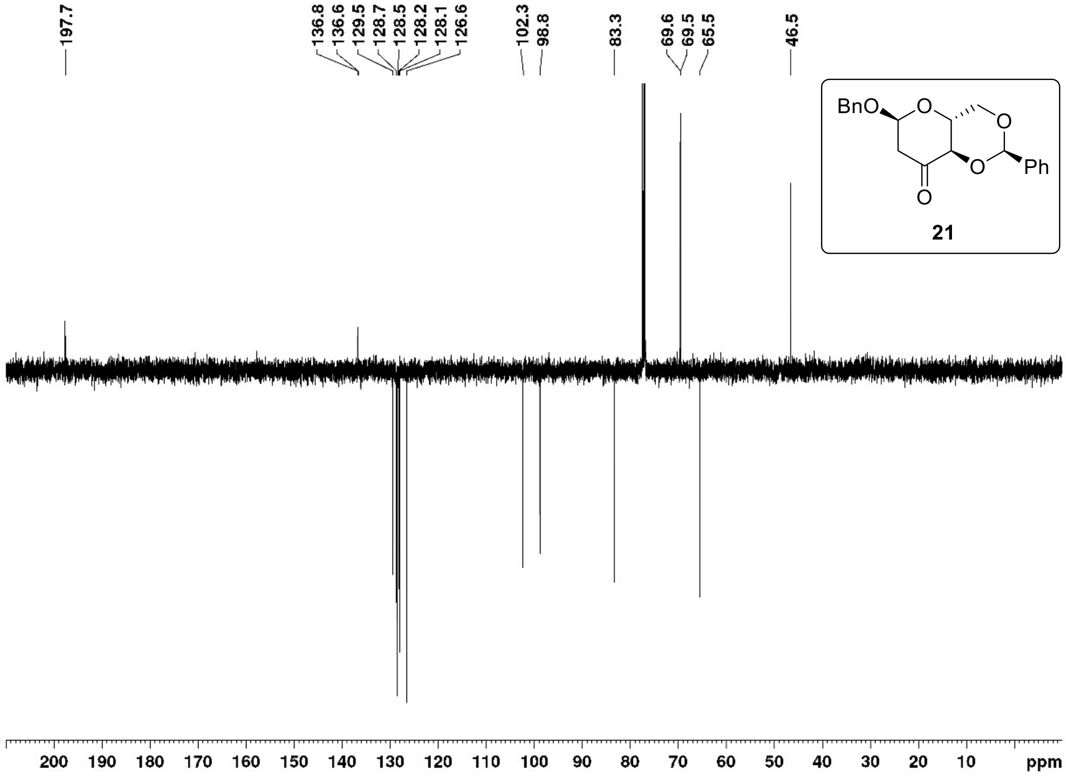
197.7
136.8
136.6
129.5
128.7
128.5
128.2
128.1
126.6
102.3
98.8
83.3
69.6
69.5
65.5
46.5
BnO
O
O
O
Ph
O
21
200 190 180 170 160 150 140 130 120 110 100 90 80 70 60 50 40 30 20 10 ppm

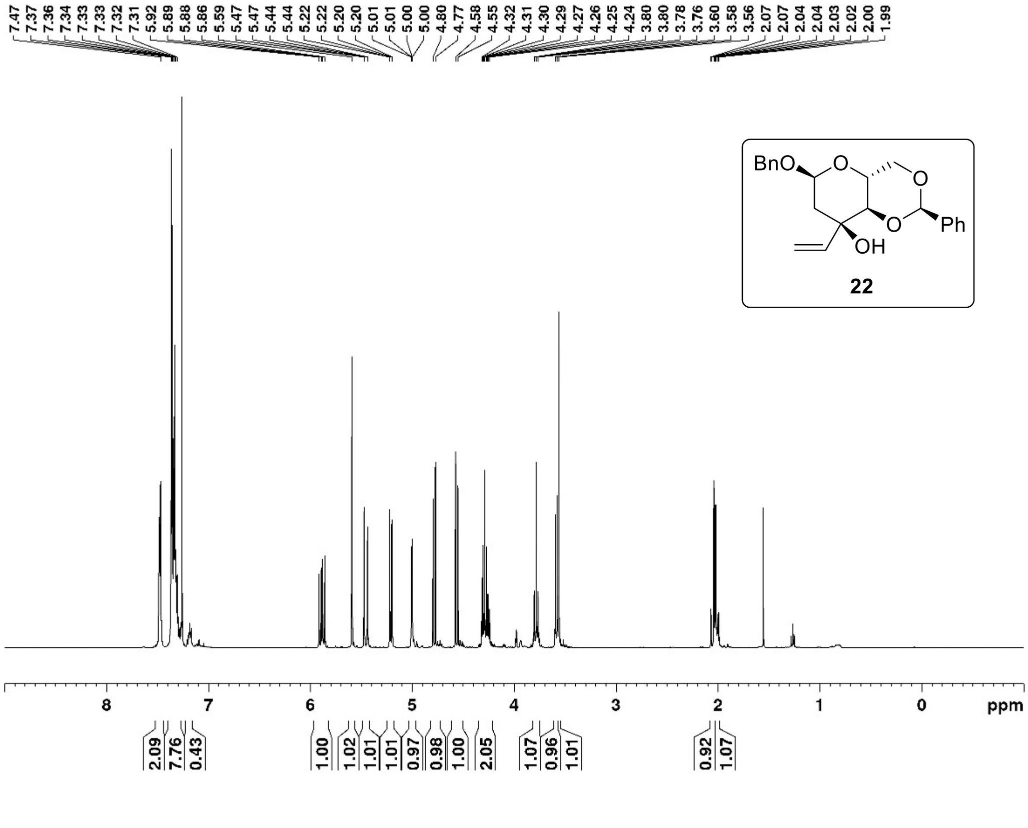
7.47
7.37
7.36
7.34
7.33
7.33
7.32
7.31
5.92
5.89
5.88
5.86
5.59
5.47
5.47
5.44
5.44
5.22
5.22
5.20
5.20
5.01
5.01
5.00
5.00
4.80
4.77
4.58
4.55
4.32
4.31
4.30
4.29
4.27
4.26
4.25
4.24
3.80
3.80
3.78
3.76
3.60
3.58
3.56
2.07
2.07
2.04
2.04
2.03
2.02
2.00
1.99
BnO
O
O
O
Ph
OH
22
8 7 6 5 4 3 2 1 0 ppm
2.09
7.76
0.43
1.00
1.02
1.01
1.01
0.97
0.98
1.00
2.05
1.07
0.96
1.01
0.92
1.07

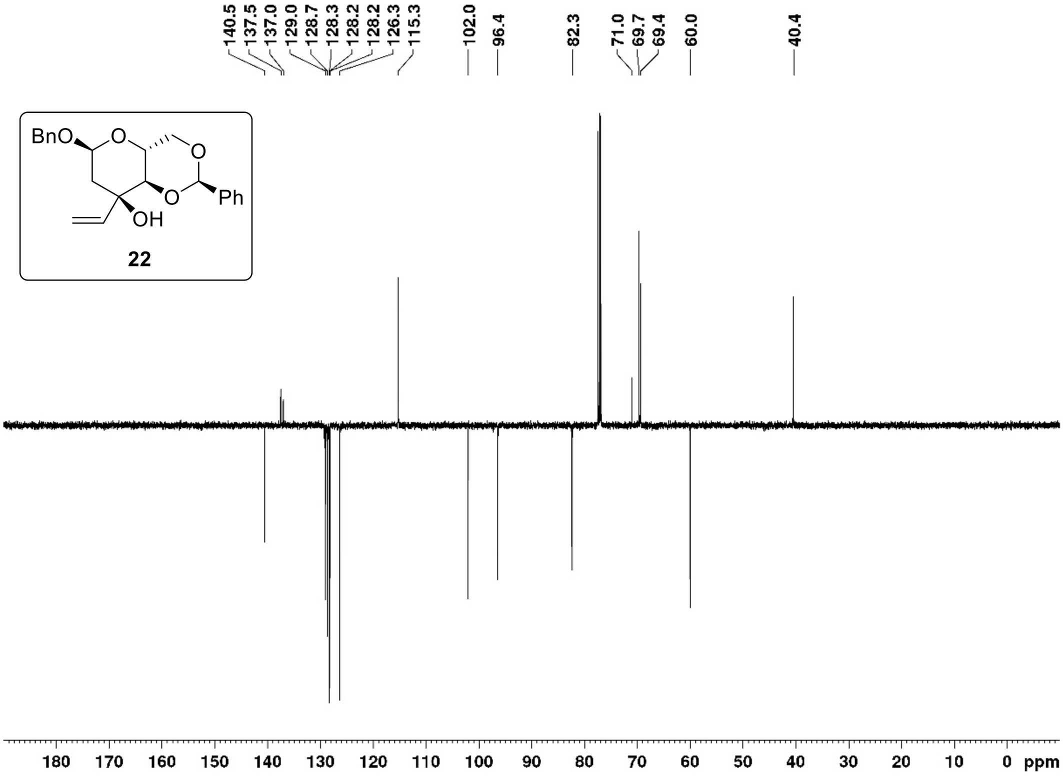
140.5
137.5
137.0
129.0
128.7
128.3
128.2
128.2
126.3
115.3
102.0
96.4
82.3
71.0
69.7
69.4
60.0
40.4
BnO
O
O
O
Ph
OH
22
180 170 160 150 140 130 120 110 100 90 80 70 60 50 40 30 20 10 0 ppm

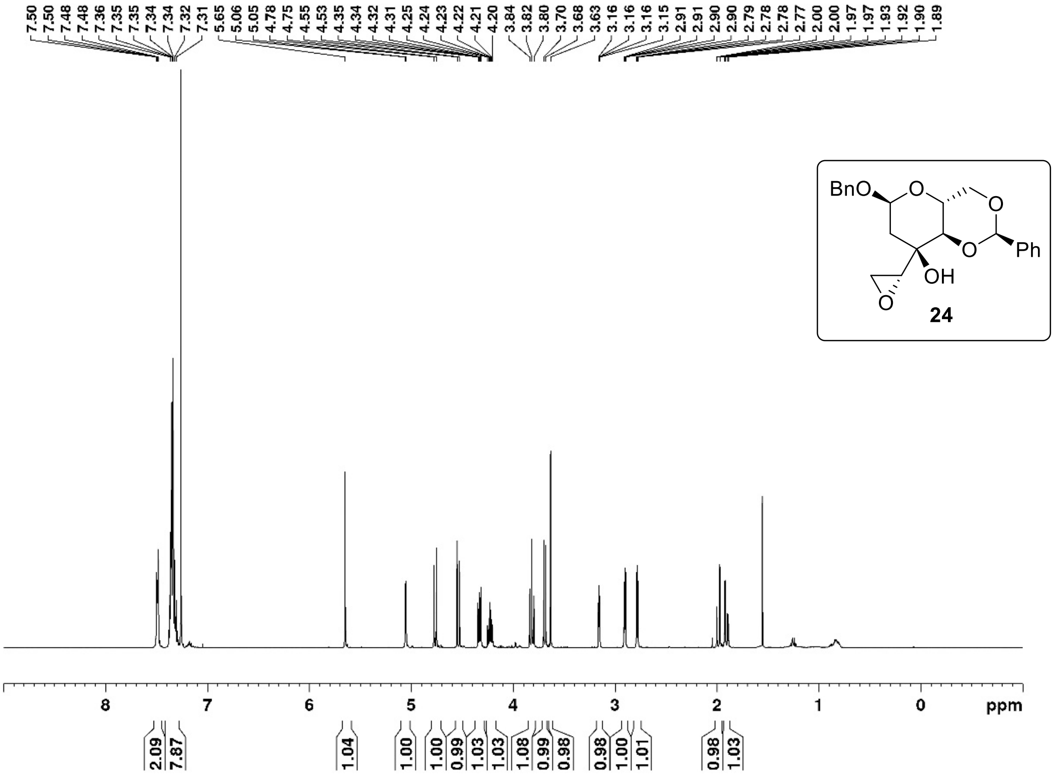
7.50
7.50
7.48
7.36
7.35
7.35
7.34
7.34
7.32
7.31
5.65
5.06
5.05
4.78
4.75
4.55
4.53
4.35
4.34
4.32
4.31
4.25
4.24
4.23
4.22
4.21
4.20
3.84
3.82
3.80
3.70
3.68
3.63
3.16
3.16
3.15
2.91
2.91
2.90
2.90
2.79
2.78
2.78
2.77
2.00
2.00
1.97
1.97
1.93
1.92
1.90
1.89
BnO
O
O
O
Ph
OH
O
24
8 7 6 5 4 3 2 1 0 ppm
2.09
7.87
1.04
1.00
1.00
0.99
1.03
1.03
1.08
0.99
0.98
0.98
1.00
1.01
0.98
1.03

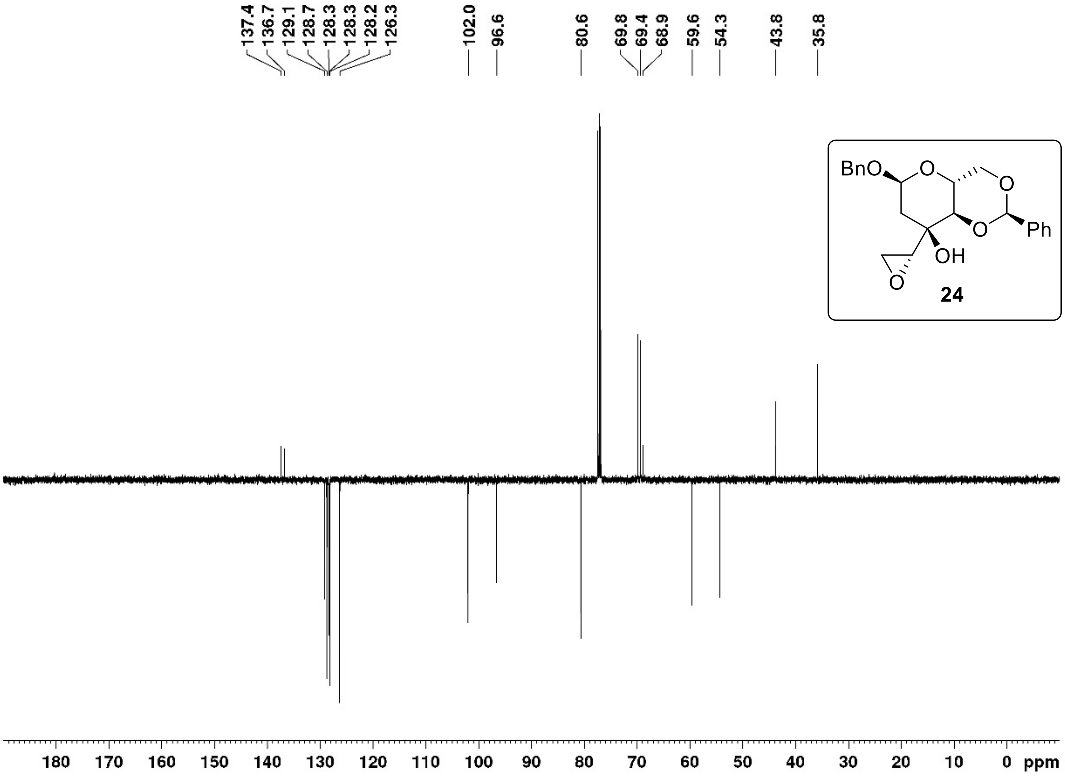
137.4
136.7
129.1
128.7
128.3
128.3
128.2
126.3
102.0
96.6
80.6
69.8
69.4
68.9
59.6
54.3
43.8
35.8
BnO
O
O
O
Ph
OH
O
24
180 170 160 150 140 130 120 110 100 90 80 70 60 50 40 30 20 10 0 ppm

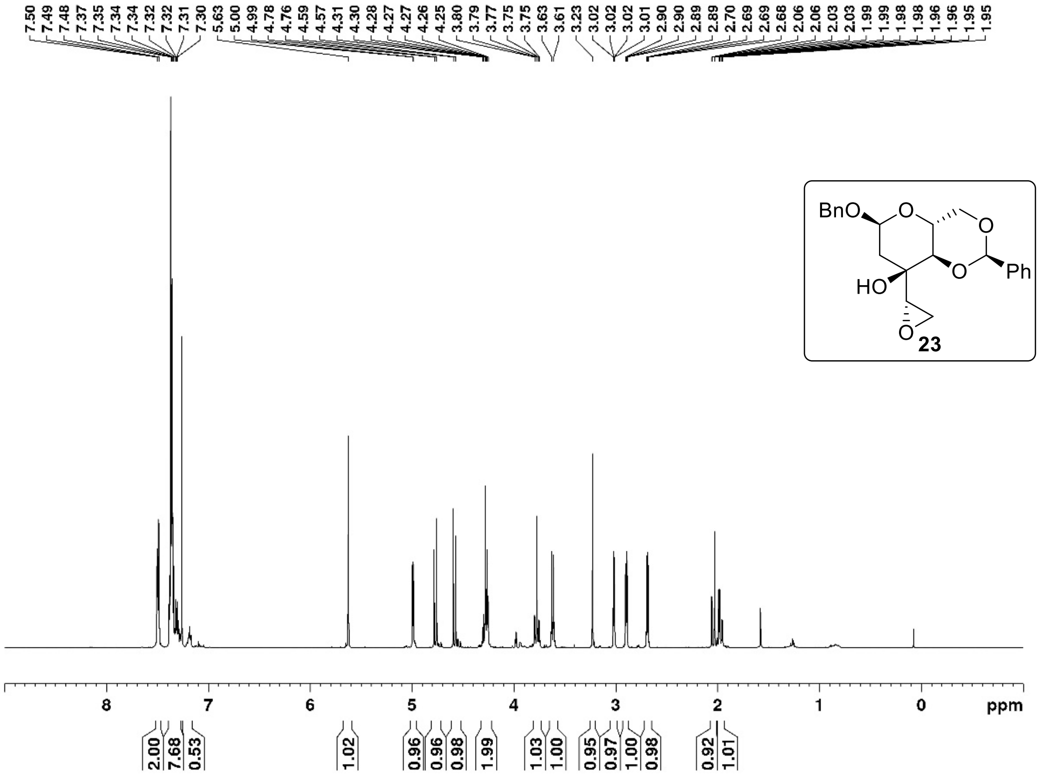
7.50
7.49
7.48
7.37
7.35
7.34
7.34
7.32
7.32
7.31
7.30
5.63
5.00
4.99
4.78
4.76
4.59
4.57
4.31
4.30
4.28
4.27
4.27
4.26
4.25
3.80
3.79
3.77
3.75
3.75
3.63
3.61
3.23
3.02
3.02
3.02
3.01
2.90
2.90
2.89
2.89
2.70
2.69
2.69
2.68
2.06
2.06
2.03
2.03
1.99
1.99
1.98
1.98
1.96
1.96
1.95
1.95
BnO
O
O
O
Ph
HO
O
23
8 7 6 5 4 3 2 1 0 ppm
2.00
7.68
0.53
1.02
0.96
0.96
0.98
1.99
1.03
1.00
0.95
0.97
1.00
0.98
0.92
1.01

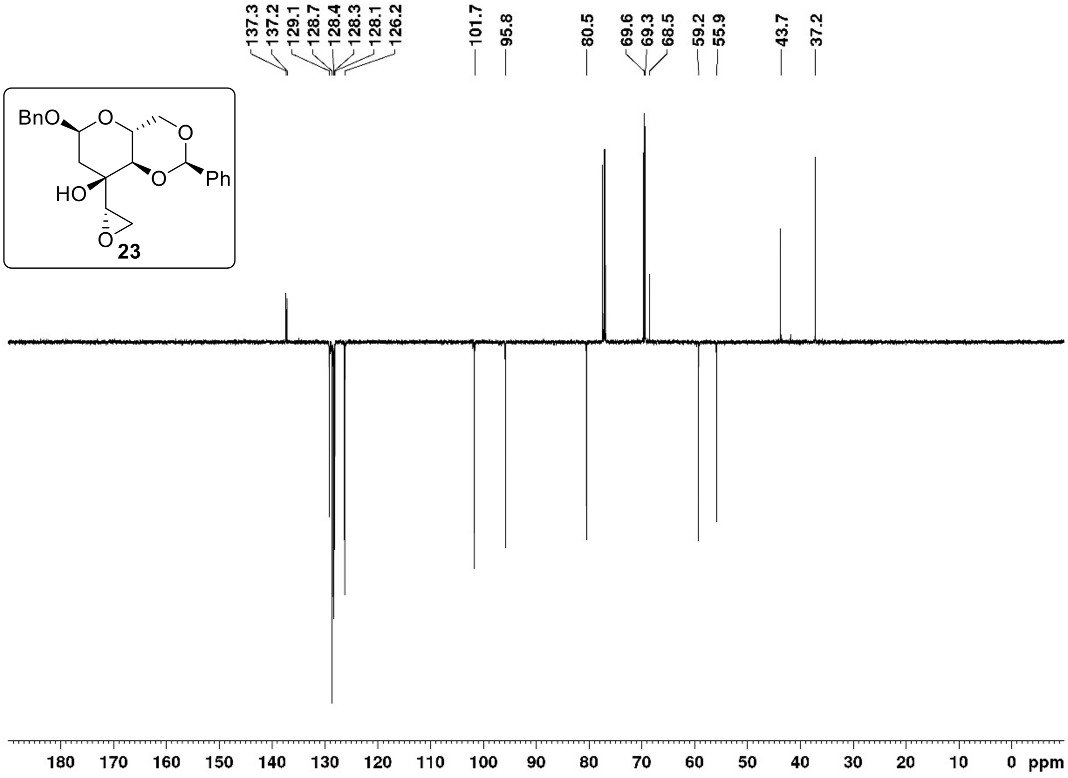
137.3
137.2
129.1
128.7
128.4
128.3
128.1
126.2
101.7
95.8
80.5
69.6
69.3
68.5
59.2
55.9
43.7
37.2
BnO
O
O
O
Ph
HO
O
23
180 170 160 150 140 130 120 110 100 90 80 70 60 50 40 30 20 10 0 ppm

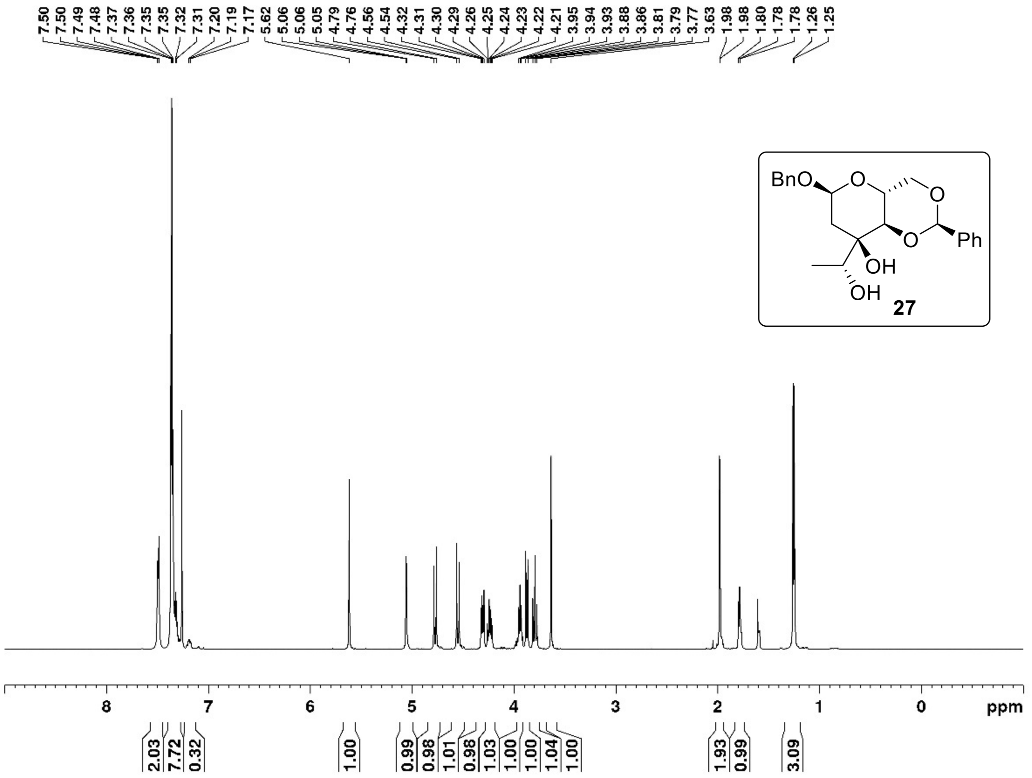
7.50
7.50
7.49
7.48
7.37
7.36
7.35
7.35
7.32
7.31
7.20
7.19
7.17
5.62
5.06
5.06
5.05
4.79
4.76
4.56
4.54
4.32
4.31
4.30
4.29
4.26
4.25
4.24
4.23
4.22
4.21
3.95
3.94
3.93
3.88
3.86
3.81
3.79
3.77
3.63
1.98
1.98
1.80
1.78
1.26
1.25
BnO
O
O
O
Ph
OH
OH
27
8 7 6 5 4 3 2 1 0 ppm
2.03
7.72
0.32
1.00
0.99
0.98
1.01
0.98
1.03
1.00
1.00
1.04
1.00
1.93
0.99
3.09

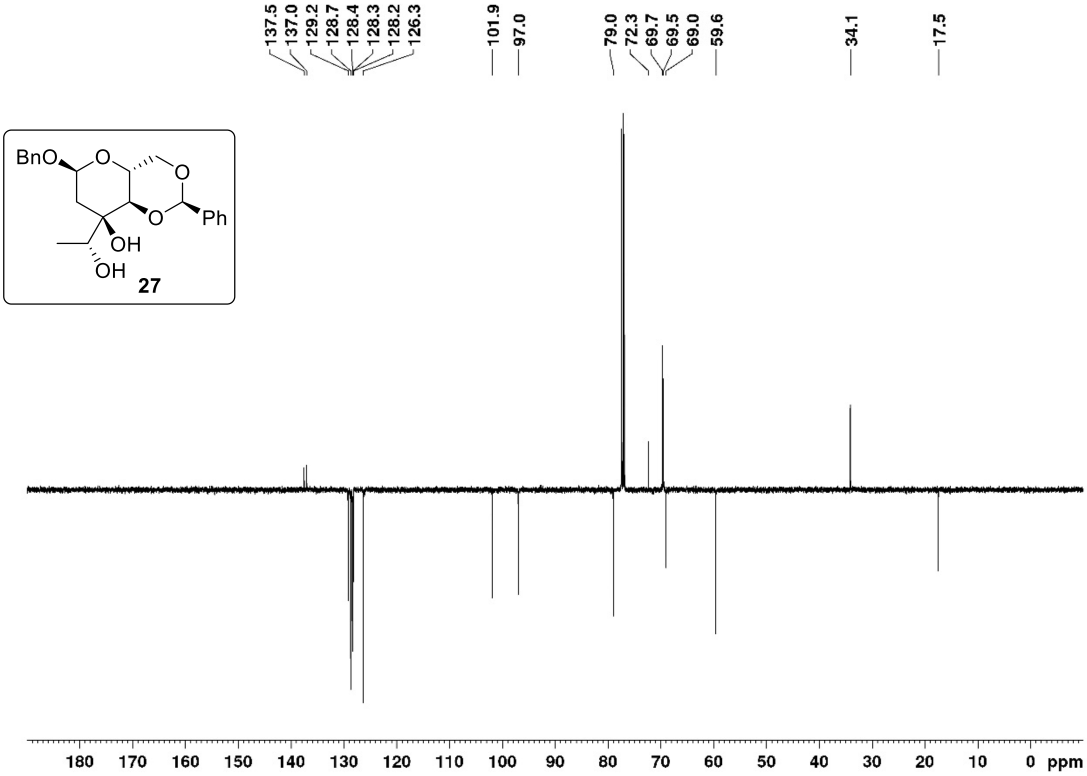
137.5
137.0
129.2
128.7
128.4
128.3
128.2
126.3
101.9
97.0
79.0
72.3
69.7
69.5
69.0
59.6
34.1
17.5
BnO
O
O
O
Ph
OH
OH
27
180 170 160 150 140 130 120 110 100 90 80 70 60 50 40 30 20 10 0 ppm

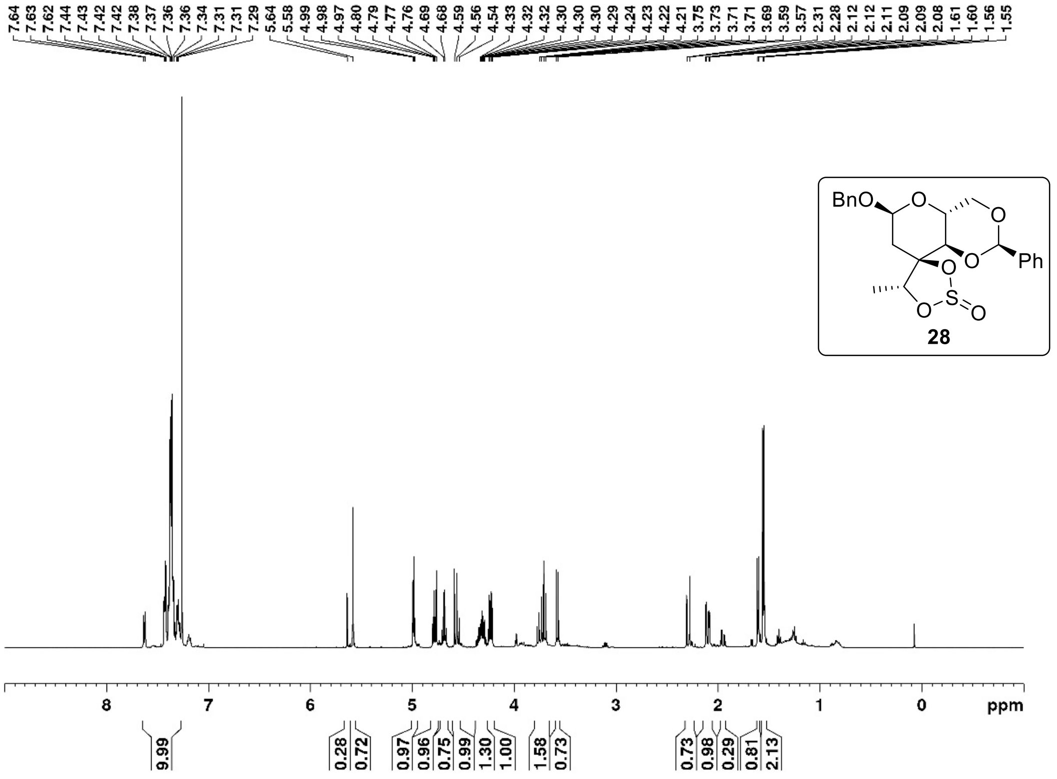
7.64
7.63
7.62
7.44
7.43
7.42
7.38
7.37
7.36
7.36
7.34
7.31
7.29
5.64
5.58
4.99
4.98
4.97
4.80
4.79
4.77
4.76
4.69
4.68
4.59
4.56
4.54
4.33
4.32
4.32
4.30
4.30
4.29
4.24
4.23
4.22
4.21
3.75
3.73
3.71
3.71
3.69
3.59
3.57
2.31
2.28
2.12
2.11
2.09
2.08
1.61
1.60
1.56
1.55
BnO
O
O
O
Ph
O
S
O
O
28
8 7 6 5 4 3 2 1 0 ppm
9.99
0.28
0.72
0.97
0.96
0.75
0.99
1.30
1.00
1.58
0.73
0.73
0.98
0.29
0.81
2.13

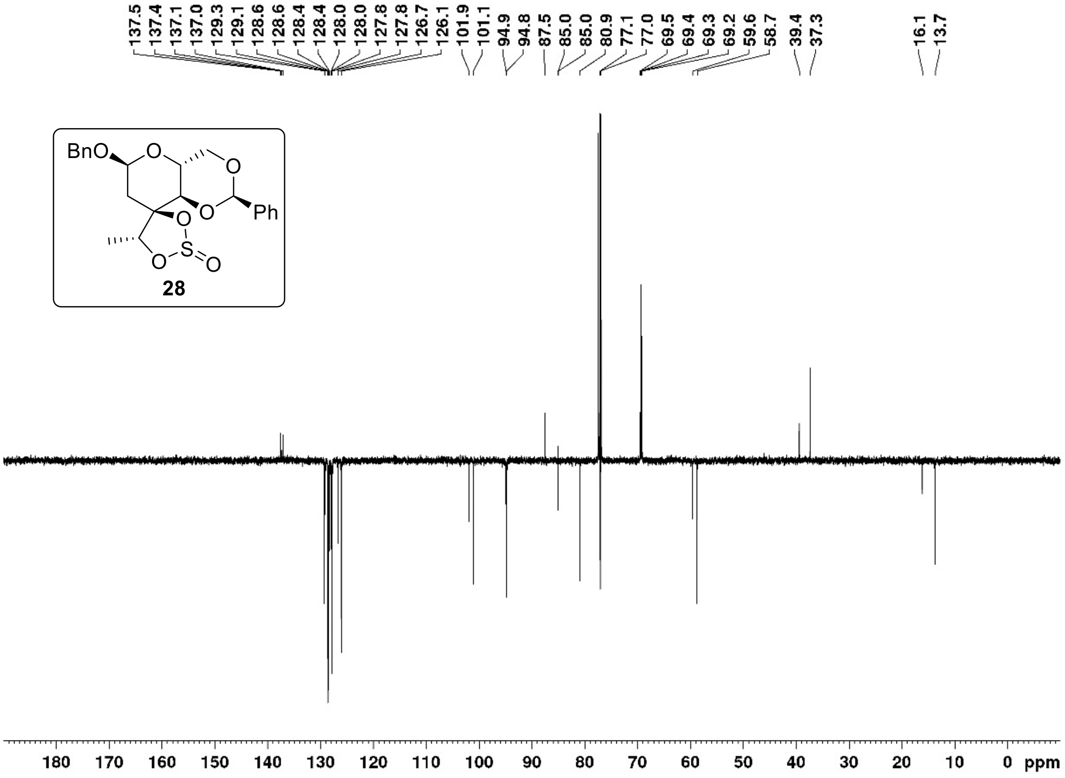
BnO
O
O
O
Ph
O
O
S
O
28
ppm

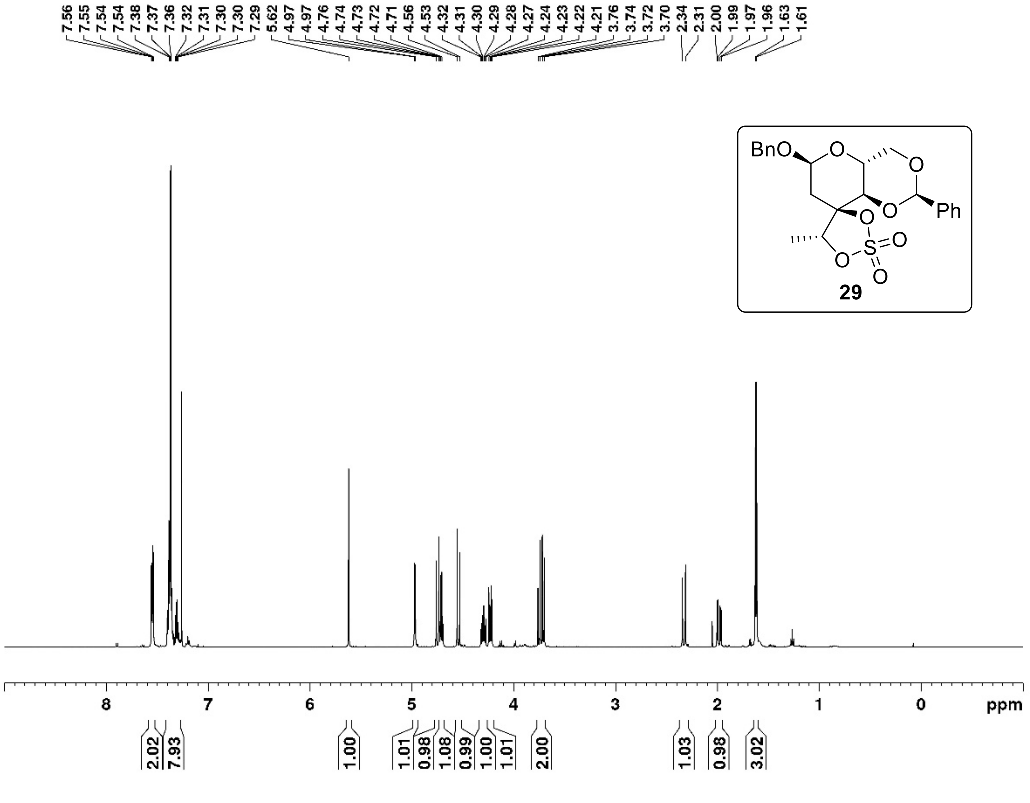
BnO
O
O
O
Ph
O
O
S
O
O
29
ppm

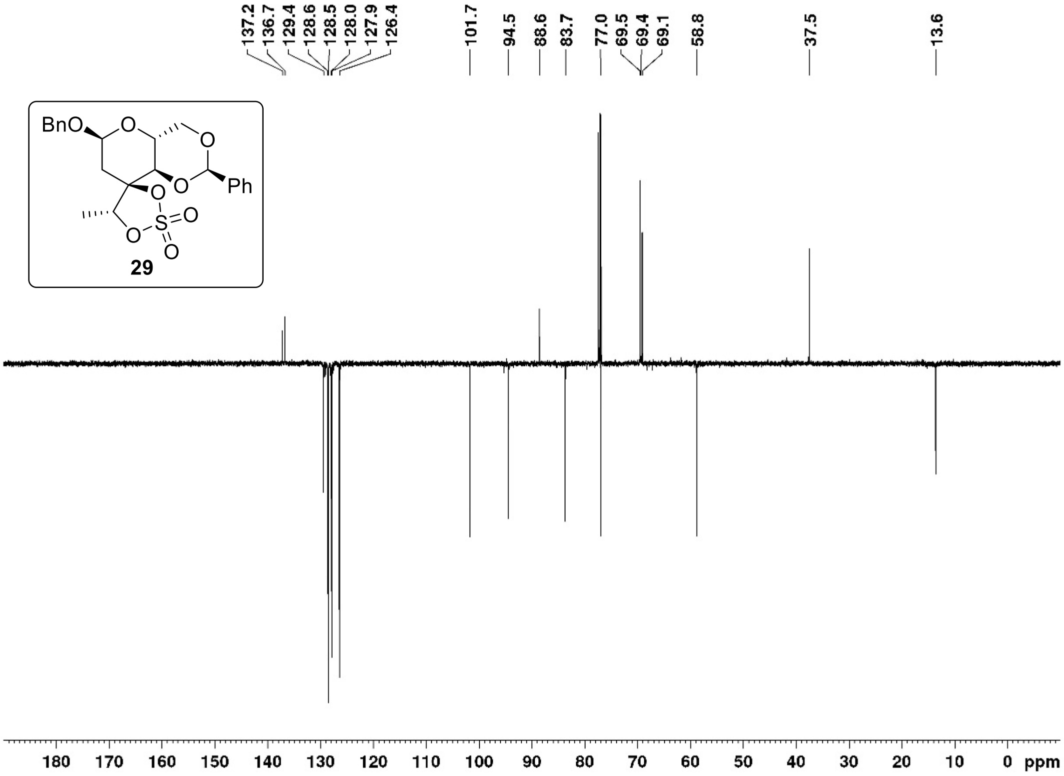
137.2 136.7 129.4 128.6 128.5 128.0 127.9 126.4
101.7
94.5
88.6
83.7
77.0
69.5 69.4 69.1
58.8
37.5
13.6
BnO
O
Ph
O=S=O
29
180 170 160 150 140 130 120 110 100 90 80 70 60 50 40 30 20 10 0 ppm

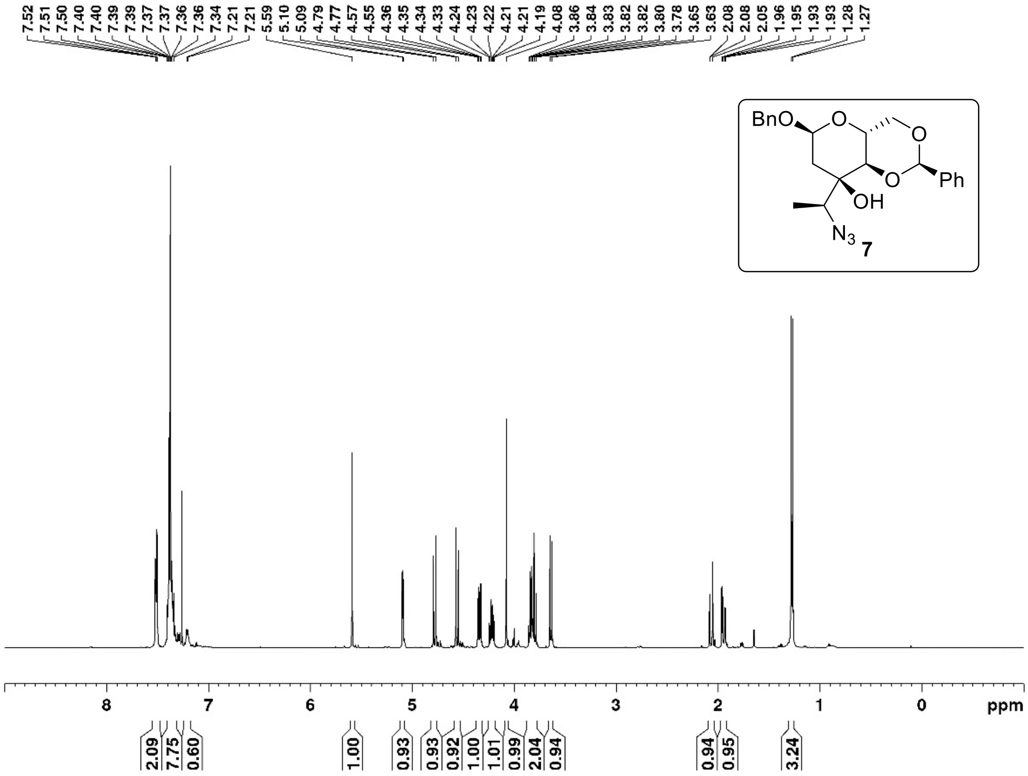
7.52 7.51 7.50 7.40 7.40 7.39 7.39 7.37 7.37 7.36 7.36 7.34 7.21 7.21 5.59 5.10 5.09 4.79 4.77 4.57 4.55 4.36 4.35 4.34 4.33 4.24 4.23 4.22 4.21 4.21 4.19 4.08 3.86 3.84 3.83 3.82 3.82 3.80 3.78 3.65 3.63 2.08 2.08 2.05 1.96 1.95 1.93 1.93 1.28 1.27
BnO
Ph
OH
N_3
7
8 7 6 5 4 3 2 1 0 ppm
2.09 7.75 0.60 1.00 0.93 0.93 0.92 1.00 1.01 0.99 2.04 0.94 0.94 0.95 3.24

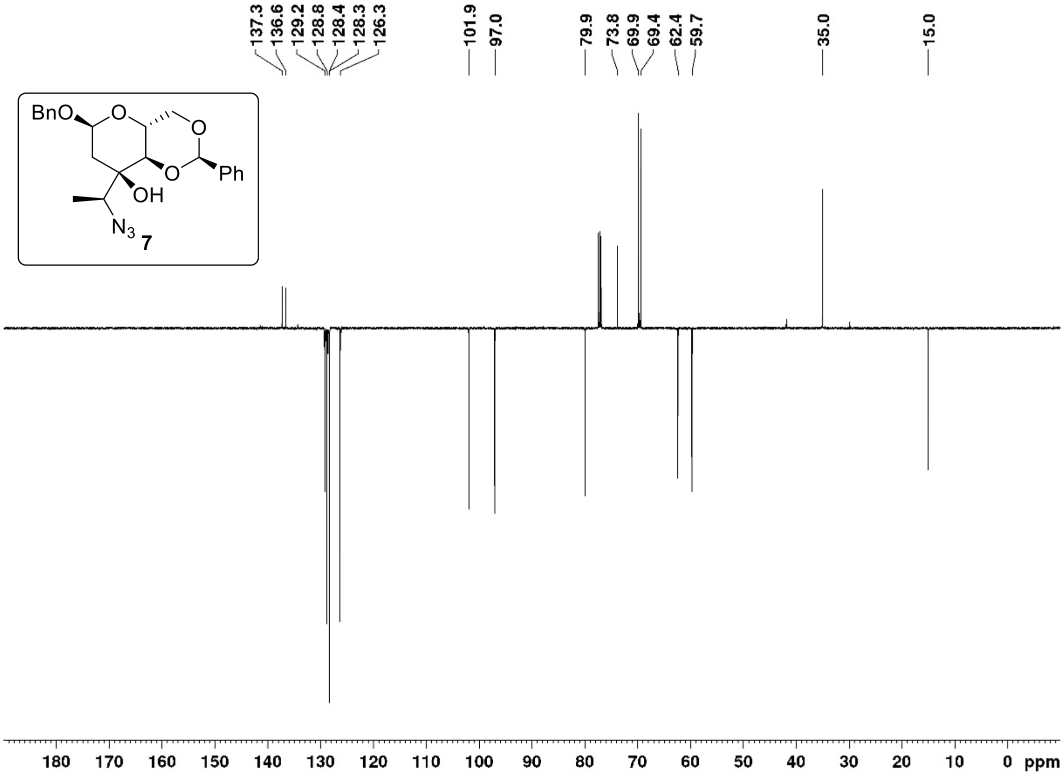
137.3
136.6
129.2
128.8
128.4
128.3
126.3
101.9
97.0
79.9
73.8
69.9
69.4
62.4
59.7
35.0
15.0
BnO
O
O
O
Ph
OH
N3
7
180 170 160 150 140 130 120 110 100 90 80 70 60 50 40 30 20 10 0 ppm

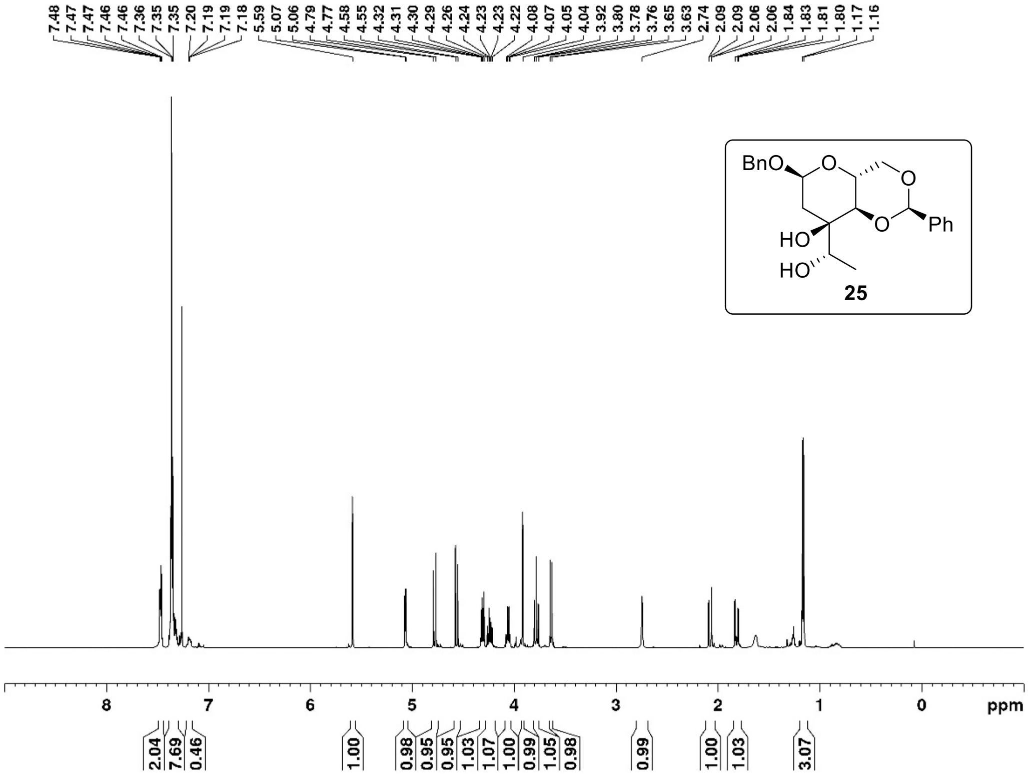
7.48
7.47
7.47
7.46
7.46
7.36
7.35
7.35
7.20
7.19
7.19
7.18
5.59
5.07
5.06
4.79
4.77
4.58
4.55
4.32
4.31
4.30
4.29
4.26
4.24
4.23
4.23
4.22
4.08
4.07
4.05
4.04
3.92
3.80
3.78
3.76
3.65
3.63
2.74
2.09
2.09
2.06
2.06
1.84
1.83
1.81
1.80
1.17
1.16
BnO
O
O
O
Ph
HO
HO
25
8 7 6 5 4 3 2 1 0 ppm
2.04
7.69
0.46
1.00
0.98
0.95
0.95
1.03
1.07
1.00
0.99
1.05
0.98
0.99
1.00
1.03
3.07

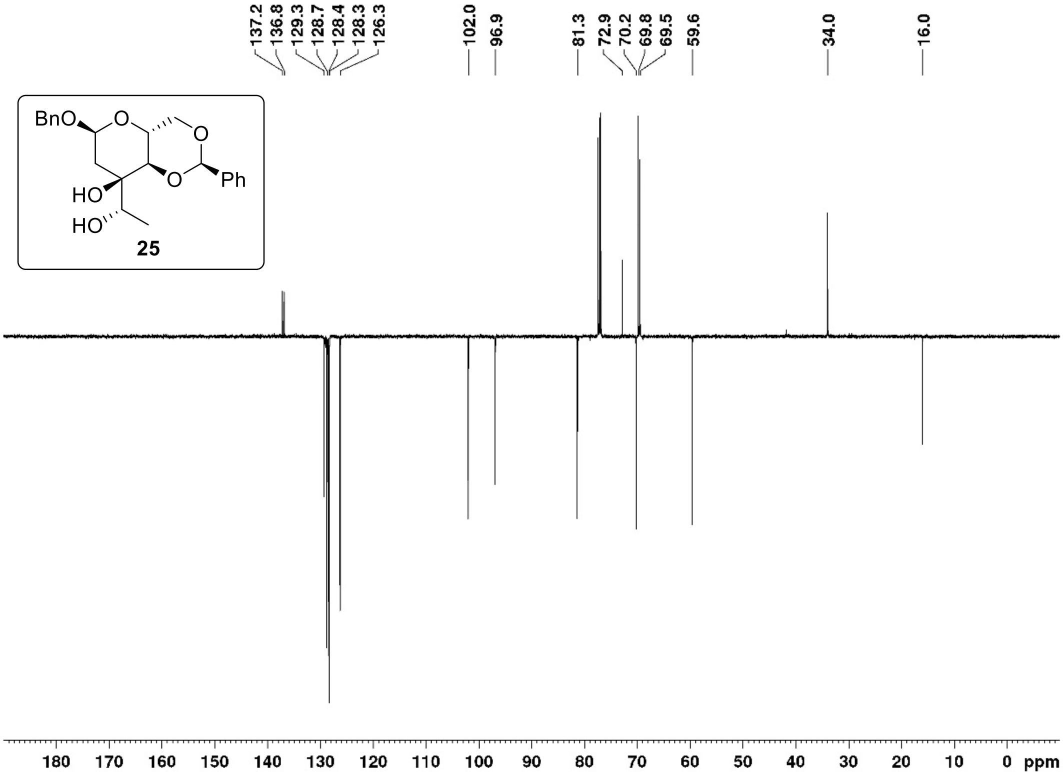
137.2
136.8
129.3
128.7
128.4
128.3
126.3
102.0
96.9
81.3
72.9
70.2
69.8
69.5
59.6
34.0
16.0
BnO
O
O
O
Ph
HO
HO
25
180 170 160 150 140 130 120 110 100 90 80 70 60 50 40 30 20 10 0 ppm

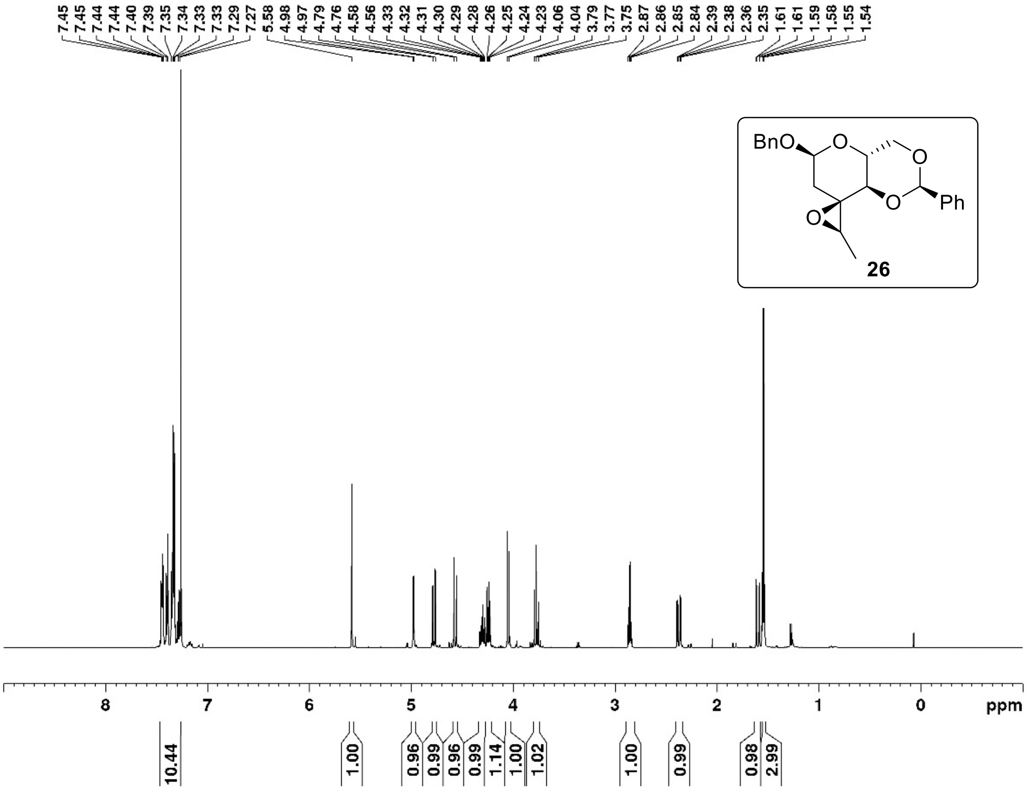
7.45
7.45
7.44
7.44
7.40
7.39
7.35
7.34
7.33
7.33
7.29
7.27
5.58
4.98
4.97
4.79
4.76
4.58
4.56
4.33
4.32
4.31
4.30
4.29
4.28
4.26
4.25
4.24
4.23
4.06
4.04
3.79
3.77
3.75
2.87
2.86
2.85
2.84
2.39
2.38
2.36
2.35
1.61
1.61
1.59
1.58
1.55
1.54
BnO
O
O
O
Ph
O
26
8 7 6 5 4 3 2 1 0 ppm
10.44
1.00
0.96
0.99
0.96
0.99
1.14
1.00
1.02
1.00
0.99
0.98
2.99

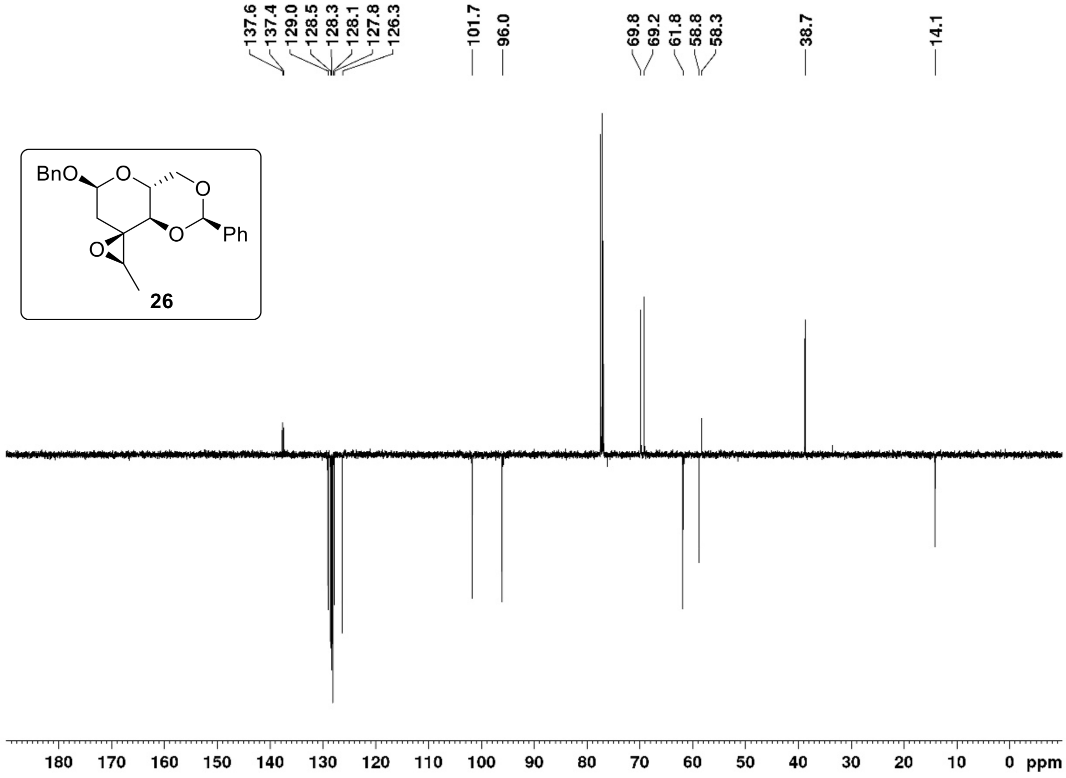
137.6
137.4
129.0
128.5
128.3
128.1
127.8
126.3
101.7
96.0
69.8
69.2
61.8
58.8
58.3
38.7
14.1
BnO
O
O
O
Ph
O
26
180 170 160 150 140 130 120 110 100 90 80 70 60 50 40 30 20 10 0 ppm

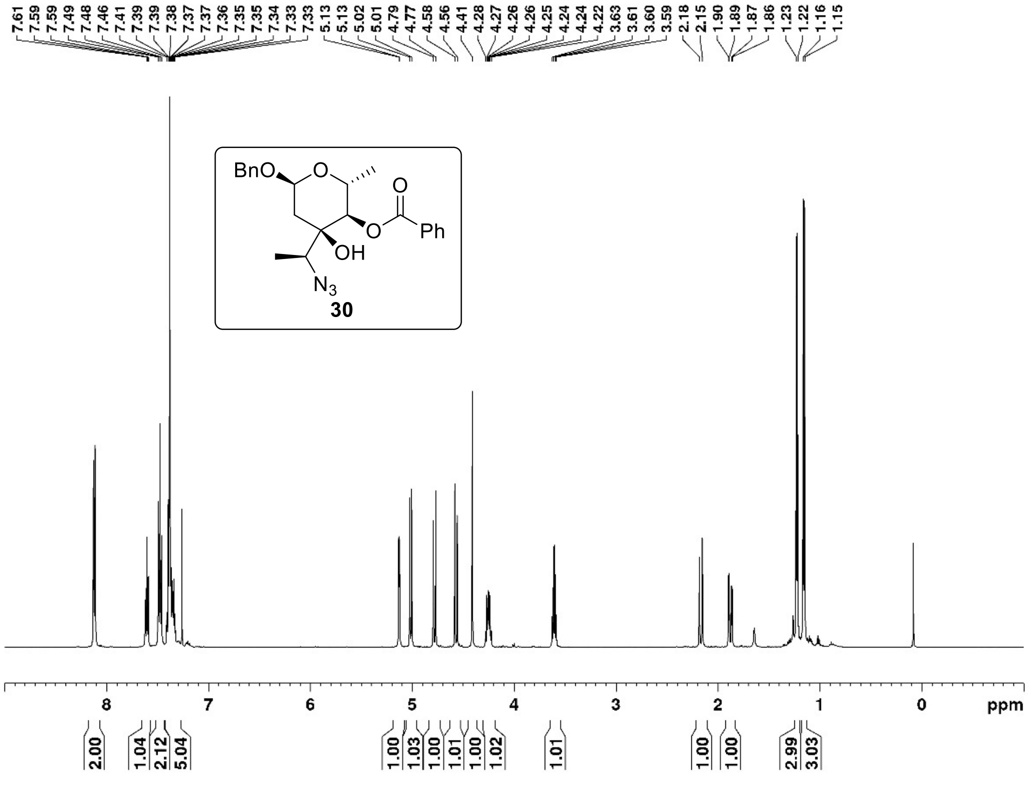
7.61 7.59 7.59 7.49 7.48 7.46 7.41 7.39 7.39 7.38 7.37 7.37 7.36 7.35 7.35 7.34 7.33 7.33 5.13 5.13 5.02 5.01 4.79 4.77 4.58 4.56 4.41 4.28 4.27 4.26 4.26 4.25 4.24 4.24 4.22 4.22 3.63 3.61 3.60 3.59 2.18 2.15 1.90 1.89 1.87 1.86 1.23 1.22 1.16 1.15
BnO
O
O
O
Ph
OH
N_3
30
8 7 6 5 4 3 2 1 0 ppm
2.00 1.04 2.12 5.04 1.00 1.03 1.00 1.01 1.00 1.02 1.01 1.00 1.00 2.99 3.03

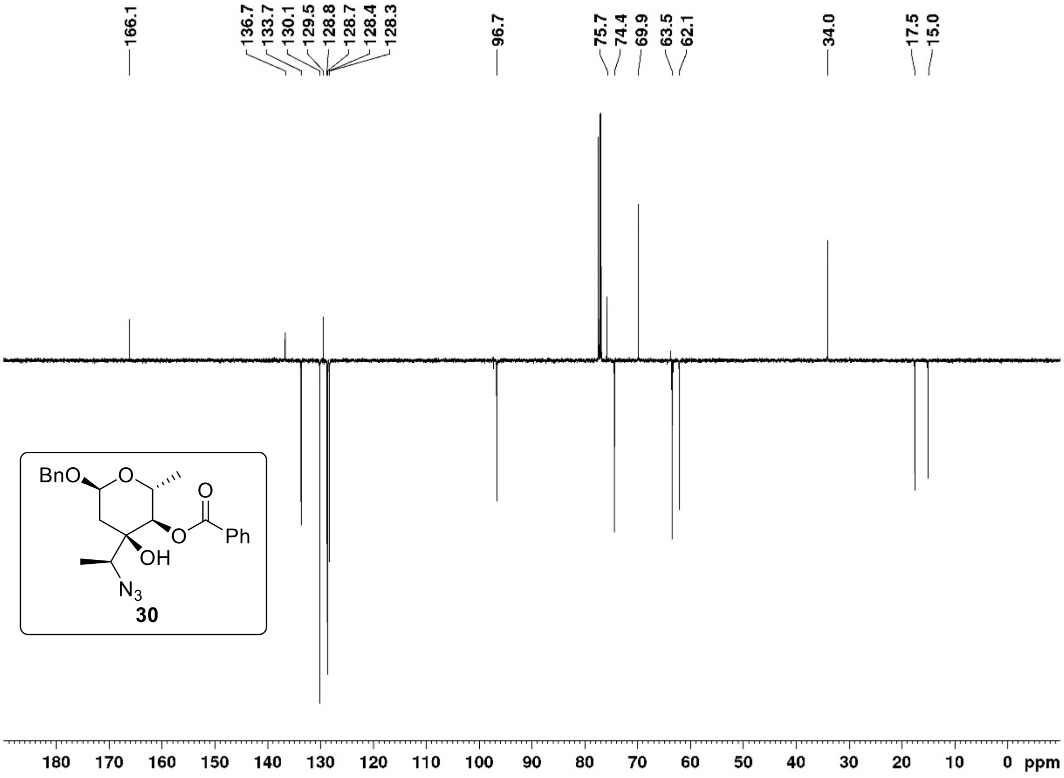
166.1
136.7
133.7
130.1
129.5
128.8
128.7
128.4
128.3
96.7
75.7
74.4
69.9
63.5
62.1
34.0
17.5
15.0
BnO
O
O
Ph
OH
N3
30
180 170 160 150 140 130 120 110 100 90 80 70 60 50 40 30 20 10 0 ppm

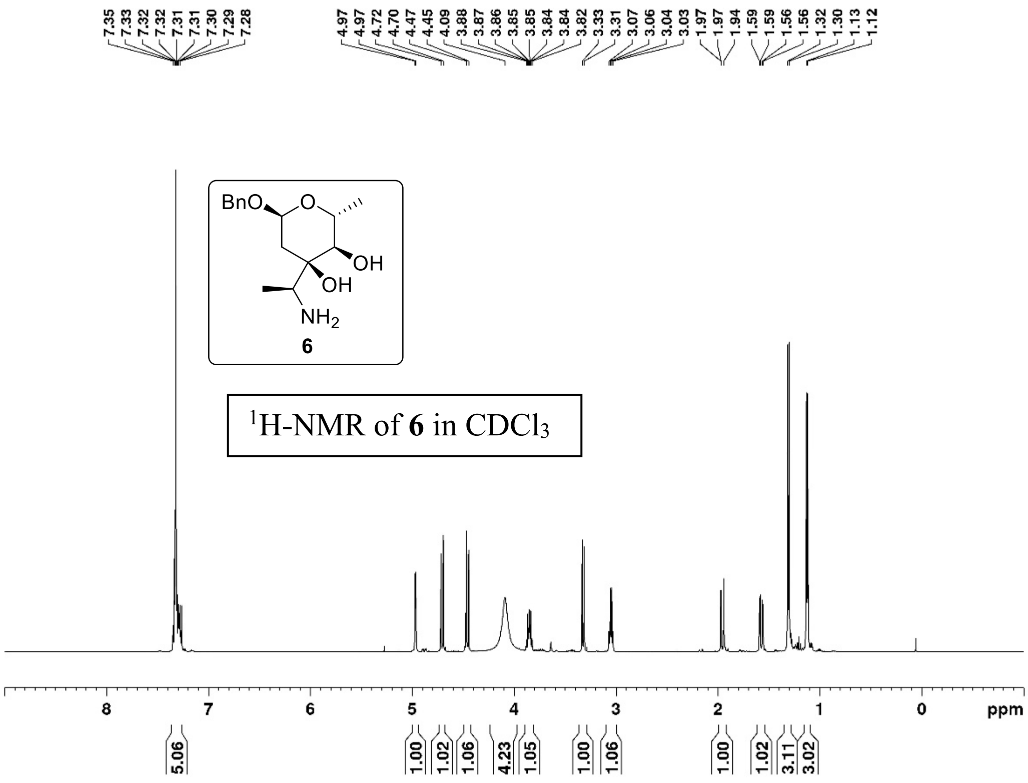
7.35
7.33
7.32
7.32
7.31
7.31
7.30
7.29
7.28
4.97
4.97
4.72
4.70
4.47
4.45
4.09
3.88
3.87
3.86
3.85
3.85
3.84
3.84
3.82
3.33
3.31
3.07
3.06
3.04
3.03
1.97
1.97
1.94
1.59
1.59
1.56
1.56
1.32
1.30
1.13
1.12
BnO
O
OH
OH
NH2
6
1H-NMR of 6 in CDCl3
8 7 6 5 4 3 2 1 0 ppm
5.06
1.00
1.02
1.06
4.23
1.05
1.00
1.06
1.00
1.02
3.11
3.02

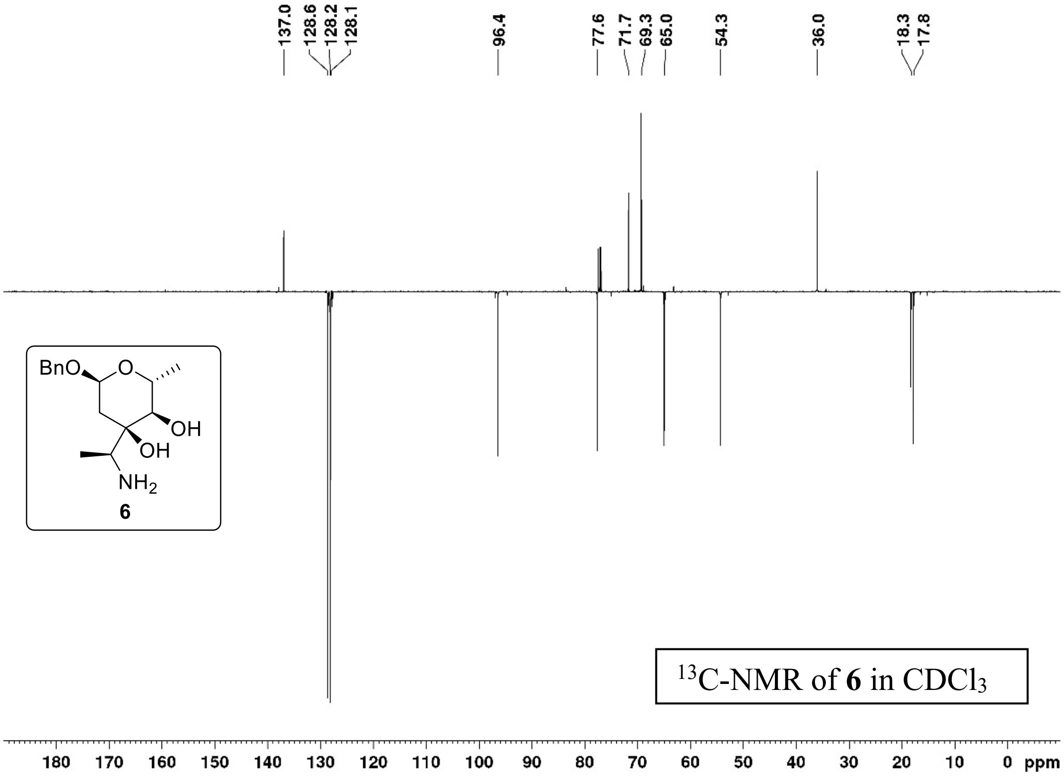
137.0
128.6
128.2
128.1
96.4
77.6
71.7
69.3
65.0
54.3
36.0
18.3
17.8
BnO
O
OH
OH
NH2
6
13C-NMR of 6 in CDCl3
180 170 160 150 140 130 120 110 100 90 80 70 60 50 40 30 20 10 0 ppm

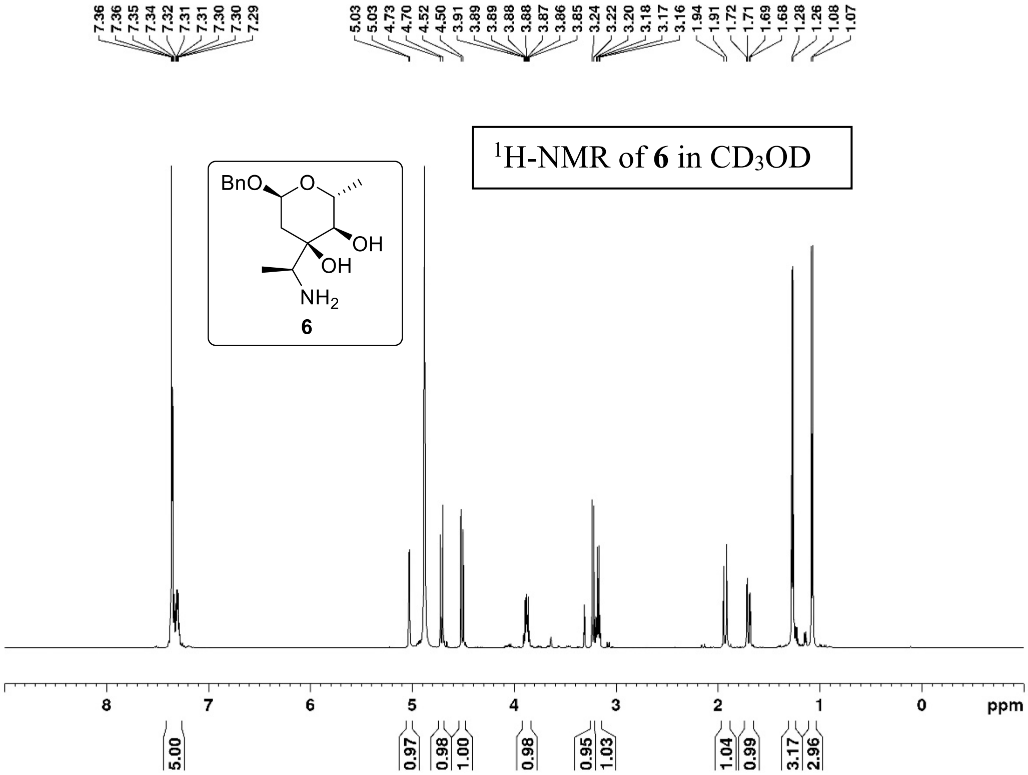
7.36
7.36
7.35
7.34
7.32
7.31
7.31
7.30
7.30
7.29
5.03
5.03
4.73
4.70
4.52
4.50
3.91
3.89
3.89
3.88
3.88
3.87
3.86
3.85
3.24
3.22
3.20
3.18
3.17
3.16
1.94
1.91
1.72
1.71
1.69
1.68
1.28
1.26
1.08
1.07
1H-NMR of 6 in CD3OD
BnO
O
OH
OH
NH2
6
8 7 6 5 4 3 2 1 0 ppm
5.00
0.97
0.98
1.00
0.98
0.95
1.03
1.04
0.99
3.17
2.96

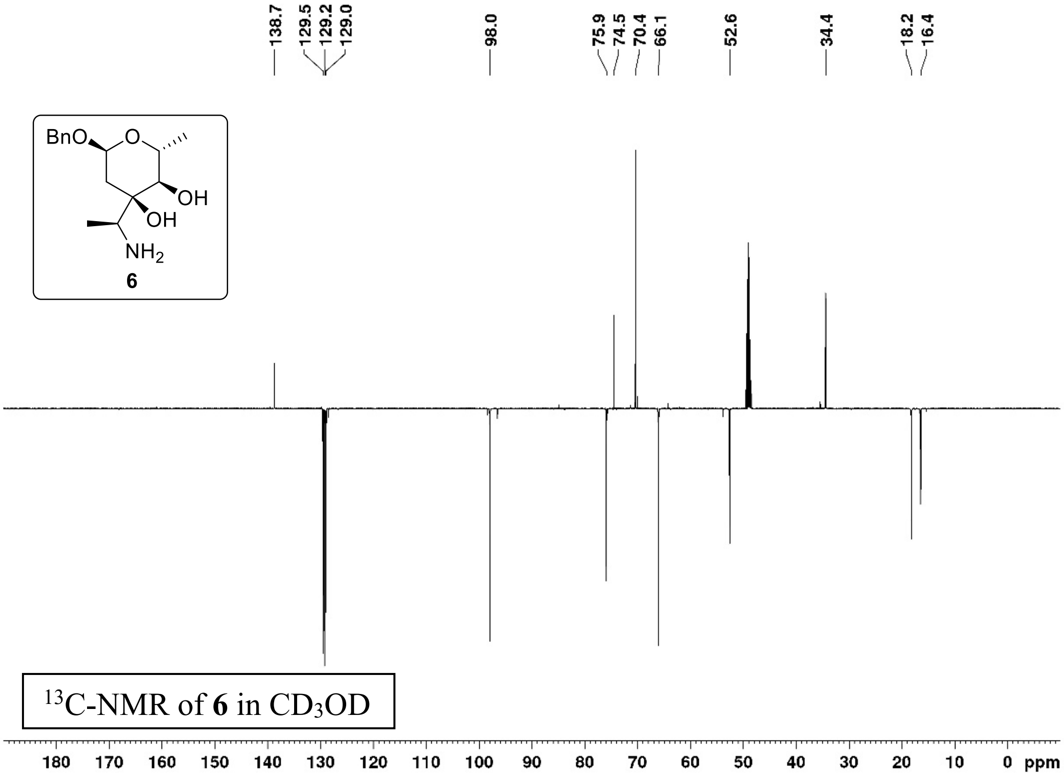
138.7
129.5
129.2
129.0
98.0
75.9
74.5
70.4
66.1
52.6
34.4
18.2
16.4
BnO
O
OH
OH
NH2
6
13C-NMR of 6 in CD3OD
180 170 160 150 140 130 120 110 100 90 80 70 60 50 40 30 20 10 0 ppm

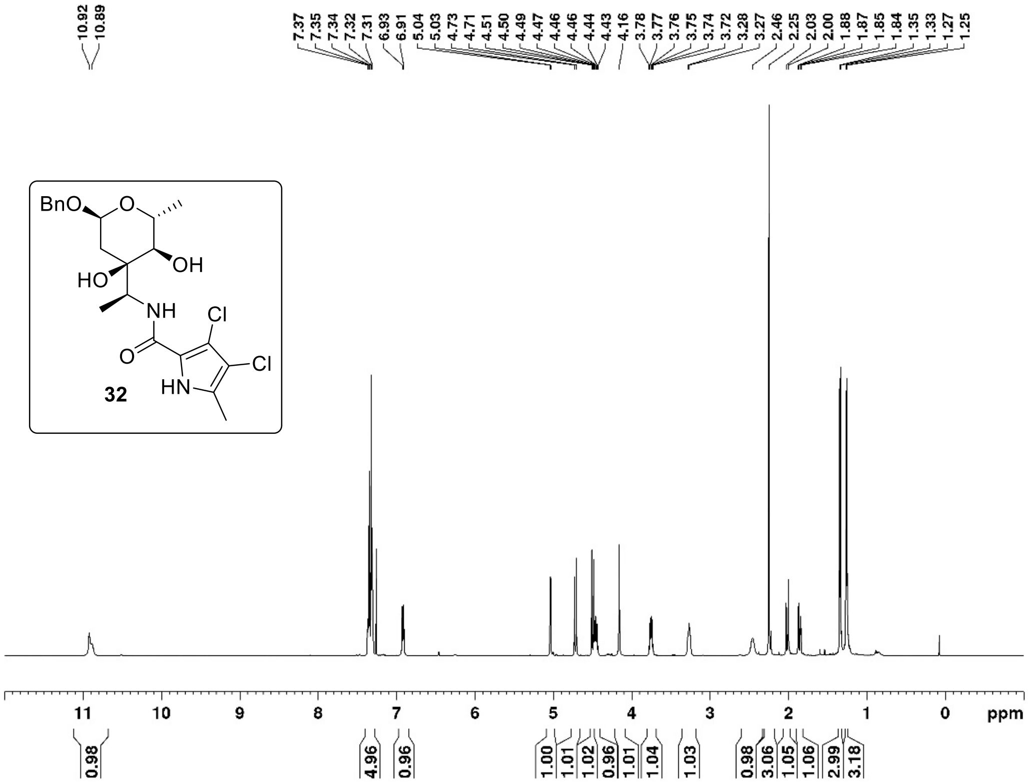
10.92
10.89
7.37
7.35
7.34
7.32
7.31
6.93
6.91
5.04
5.03
4.73
4.71
4.51
4.50
4.49
4.47
4.46
4.46
4.44
4.43
4.16
3.78
3.77
3.76
3.75
3.74
3.72
3.28
3.27
2.46
2.25
2.03
2.00
1.88
1.87
1.85
1.84
1.35
1.33
1.27
1.25
BnO
O
HO
OH
NH
Cl
Cl
O
HN
32
11 10 9 8 7 6 5 4 3 2 1 0 ppm
0.98
4.96
0.96
1.00
1.01
1.02
0.96
1.01
1.04
1.03
0.98
3.06
1.05
1.06
2.99
3.18

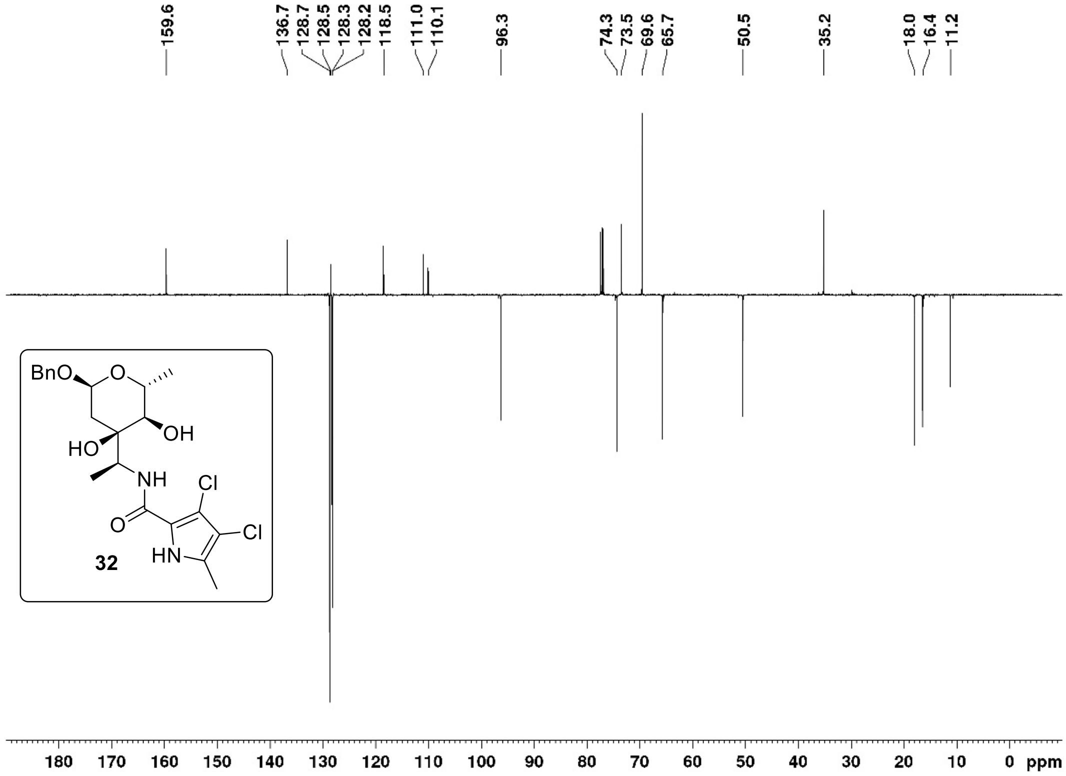
159.6
136.7 128.7 128.5 128.3 128.2 118.5 111.0 110.1
96.3
74.3 73.5 69.6 65.7
50.5
35.2
18.0 16.4 11.2
BnO O OH HO NH Cl Cl O HN
32
180 170 160 150 140 130 120 110 100 90 80 70 60 50 40 30 20 10 0 ppm

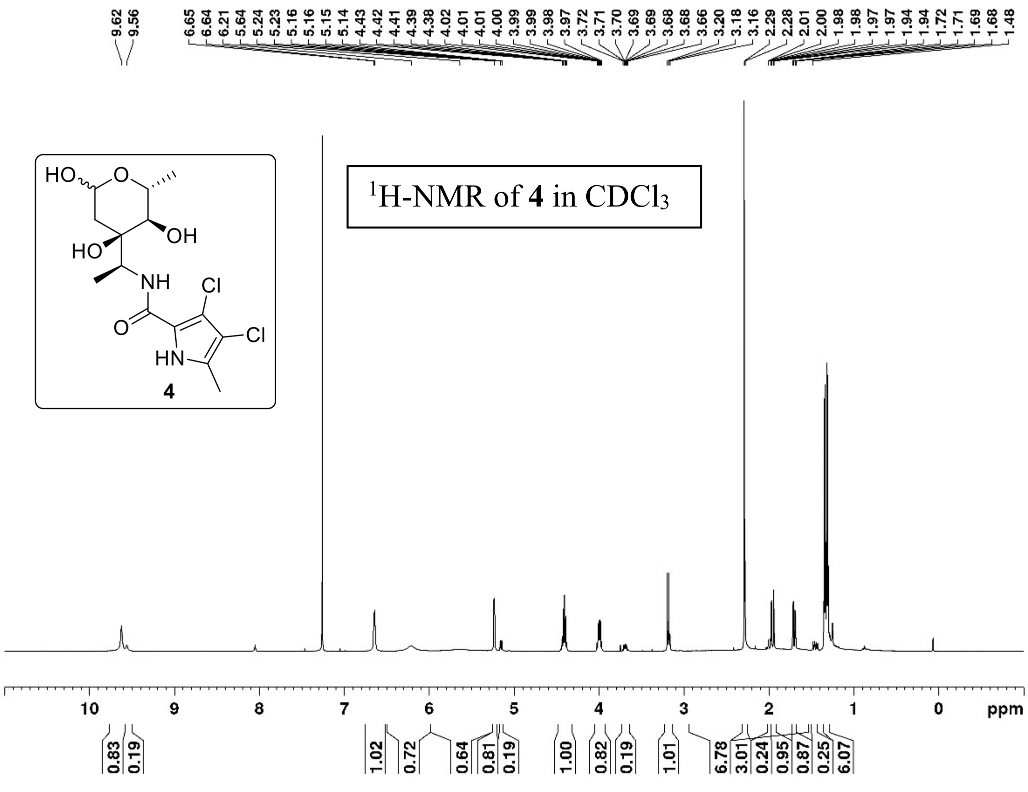
9.62 9.56
6.65 6.64 6.21 5.64 5.24 5.23 5.16 5.16 5.15 5.14 4.43 4.42 4.41 4.39 4.38 4.02 4.01 4.01 4.00 3.99 3.99 3.98 3.97 3.72 3.71 3.70 3.69 3.69 3.68 3.68 3.66 3.20 3.18 3.16 2.29 2.28 2.01 2.00 1.98 1.98 1.97 1.97 1.94 1.94 1.72 1.71 1.69 1.68 1.48
HO O OH HO NH Cl Cl O HN
4
^{1}H-NMR of **4** in CDCl$_3$
10 9 8 7 6 5 4 3 2 1 0 ppm
0.83 0.19 1.02 0.72 0.64 0.81 0.19 1.00 0.82 0.19 1.01 6.78 3.01 0.24 0.95 0.87 0.25 6.07

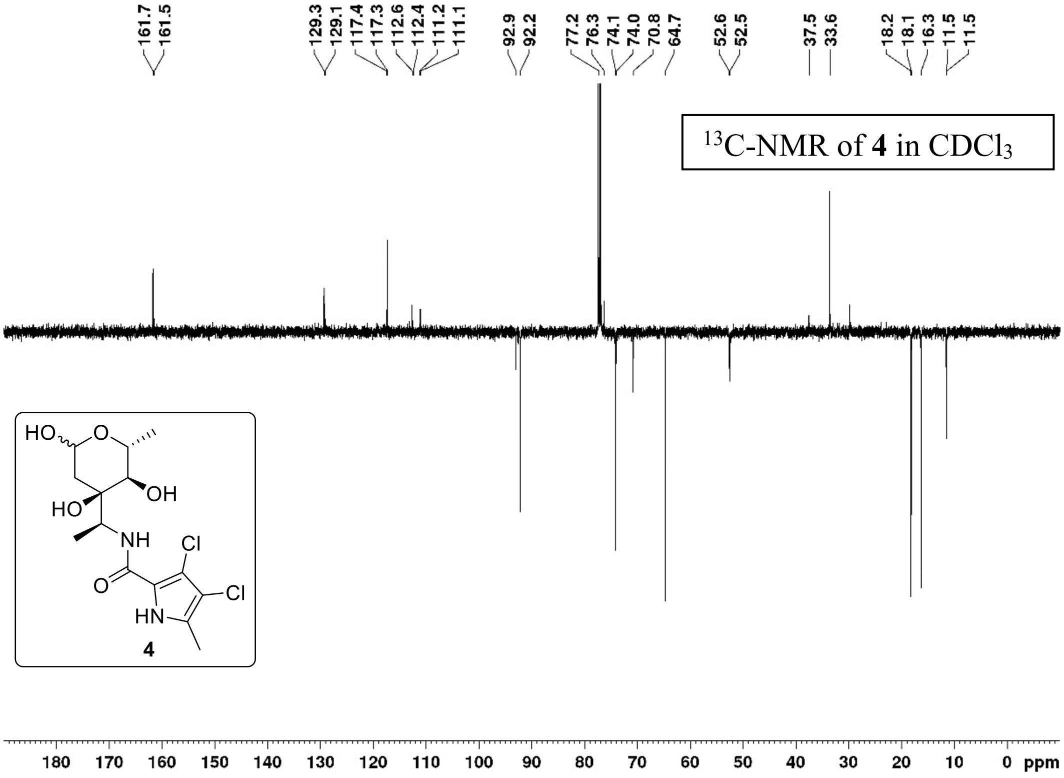
161.7
161.5
129.3
129.1
117.4
117.3
112.6
112.4
111.2
111.1
92.9
92.2
77.2
76.3
74.1
74.0
70.8
64.7
52.6
52.5
37.5
33.6
18.2
18.1
16.3
11.5
11.5
^{13}C-NMR of **4** in $CDCl_3$
HO
O
OH
HO
NH
Cl
Cl
O
HN
4
180 170 160 150 140 130 120 110 100 90 80 70 60 50 40 30 20 10 0 ppm

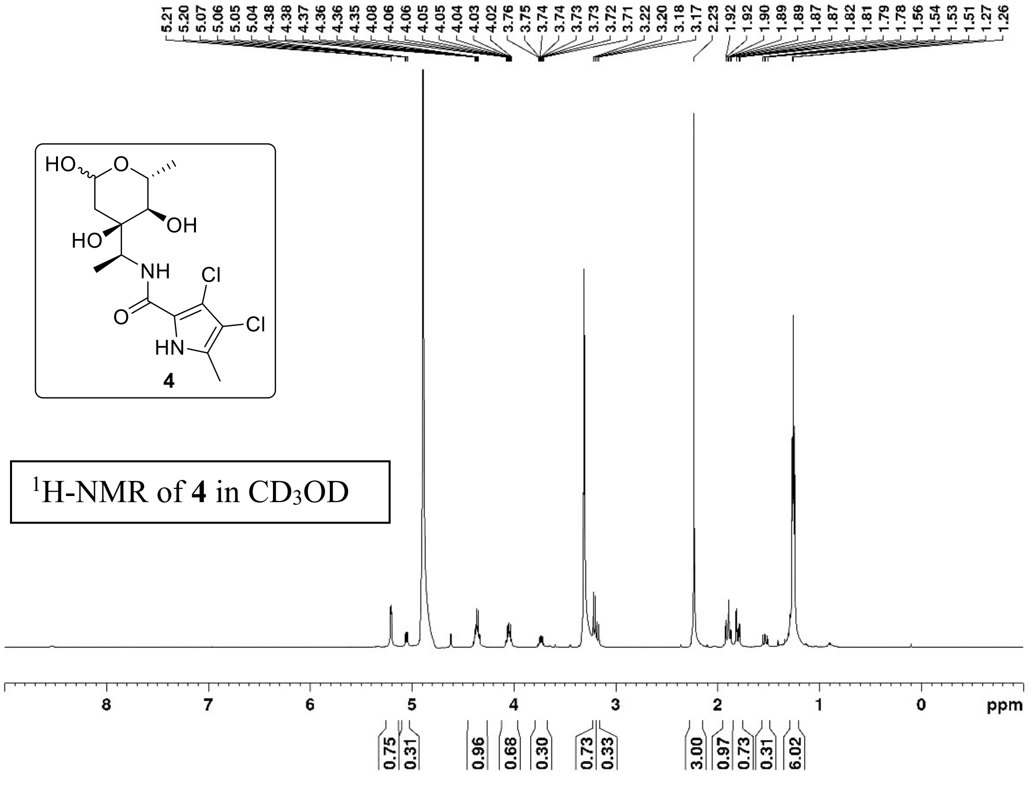
5.21
5.20
5.07
5.06
5.05
5.04
4.38
4.38
4.37
4.36
4.36
4.35
4.08
4.06
4.06
4.05
4.05
4.04
4.03
4.02
3.76
3.75
3.74
3.74
3.73
3.73
3.72
3.71
3.22
3.20
3.18
3.17
2.23
1.92
1.92
1.90
1.89
1.89
1.87
1.87
1.82
1.81
1.79
1.78
1.56
1.54
1.53
1.51
1.27
1.26
HO
O
OH
HO
NH
Cl
Cl
O
HN
4
^{1}H-NMR of **4** in CD_3OD
8 7 6 5 4 3 2 1 0 ppm
0.75
0.31
0.96
0.68
0.30
0.73
0.33
3.00
0.97
0.73
0.31
6.02

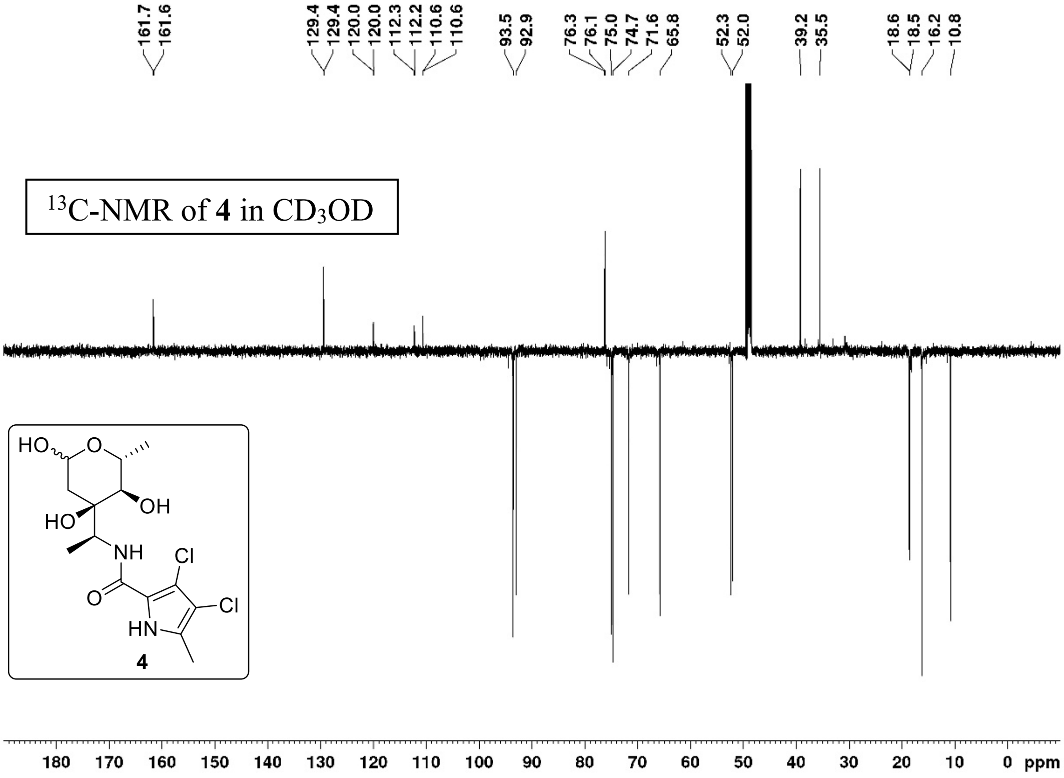
161.7
161.6
129.4
129.4
120.0
120.0
112.3
112.2
110.6
110.6
93.5
92.9
76.3
76.1
75.0
74.7
71.6
65.8
52.3
52.0
39.2
35.5
18.6
18.5
16.2
10.8
^{13}C-NMR of **4** in CD_3OD
HO
O
OH
HO
NH
Cl
Cl
O
HN
4
180 170 160 150 140 130 120 110 100 90 80 70 60 50 40 30 20 10 0 ppm

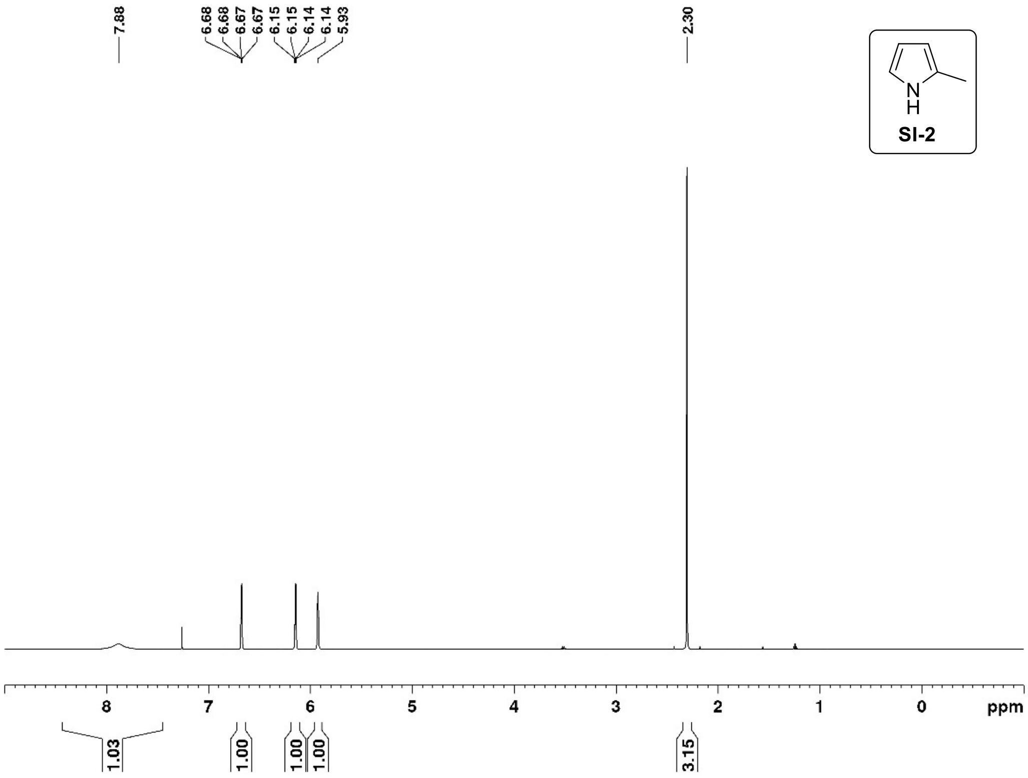
7.88
6.68
6.68
6.67
6.67
6.15
6.15
6.14
6.14
5.93
2.30
N
H
SI-2
8 7 6 5 4 3 2 1 0 ppm
1.03
1.00
1.00
1.00
3.15

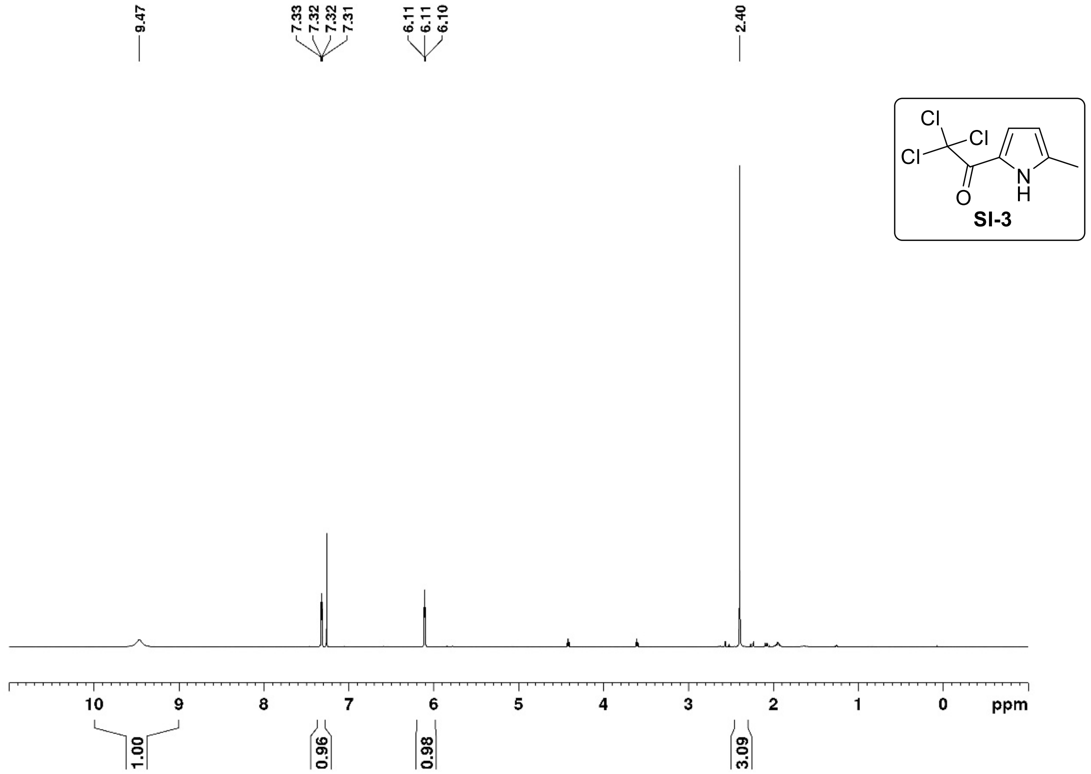
9.47
7.33
7.32
7.32
7.31
6.11
6.11
6.10
2.40
Cl
Cl
Cl
O
N
H
SI-3
10
9
8
7
6
5
4
3
2
1
0
ppm
1.00
0.96
0.98
3.09

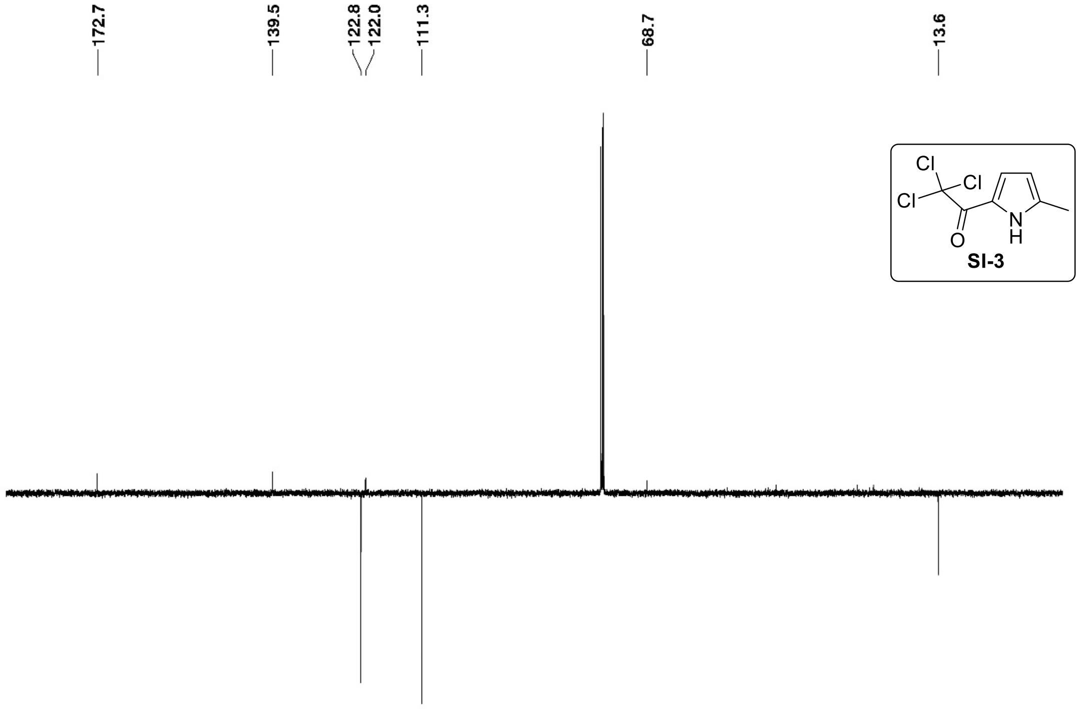
172.7
139.5
122.8
122.0
111.3
68.7
13.6
Cl
Cl
Cl
O
N
H
SI-3
180
170
160
150
140
130
120
110
100
90
80
70
60
50
40
30
20
10
0
ppm

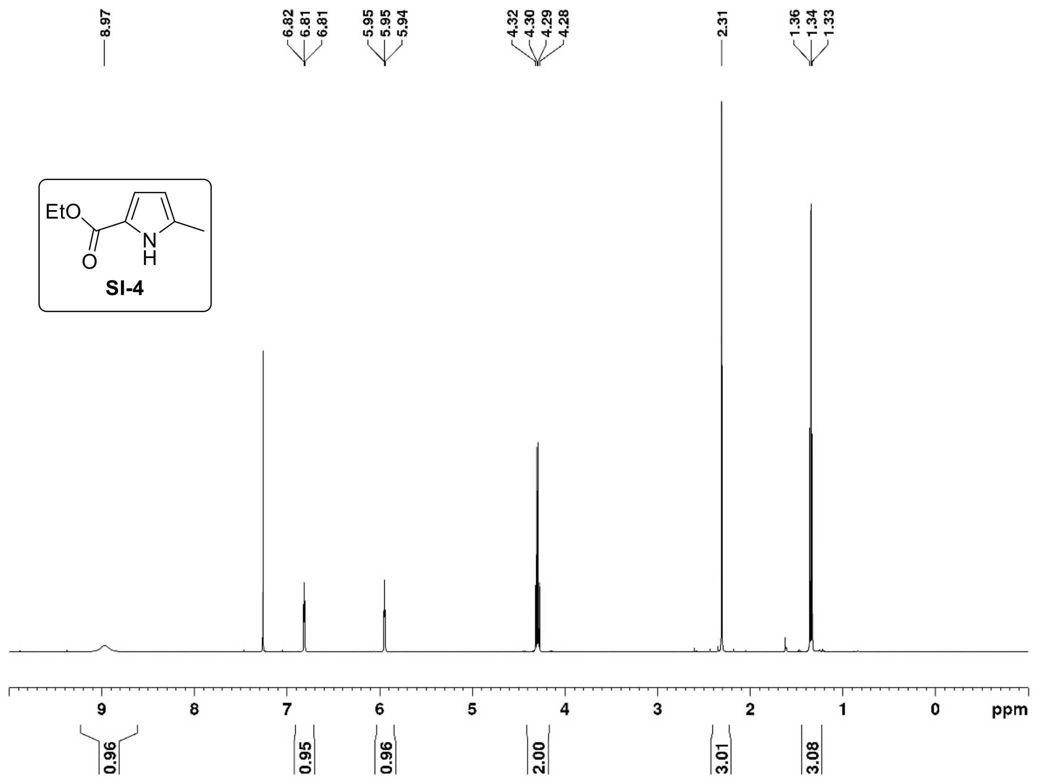
8.97
6.82
6.81
6.81
5.95
5.95
5.94
4.32
4.30
4.29
4.28
2.31
1.36
1.34
1.33
EtO
O
N
H
SI-4
9
8
7
6
5
4
3
2
1
0
ppm
0.96
0.95
0.96
2.00
3.01
3.08

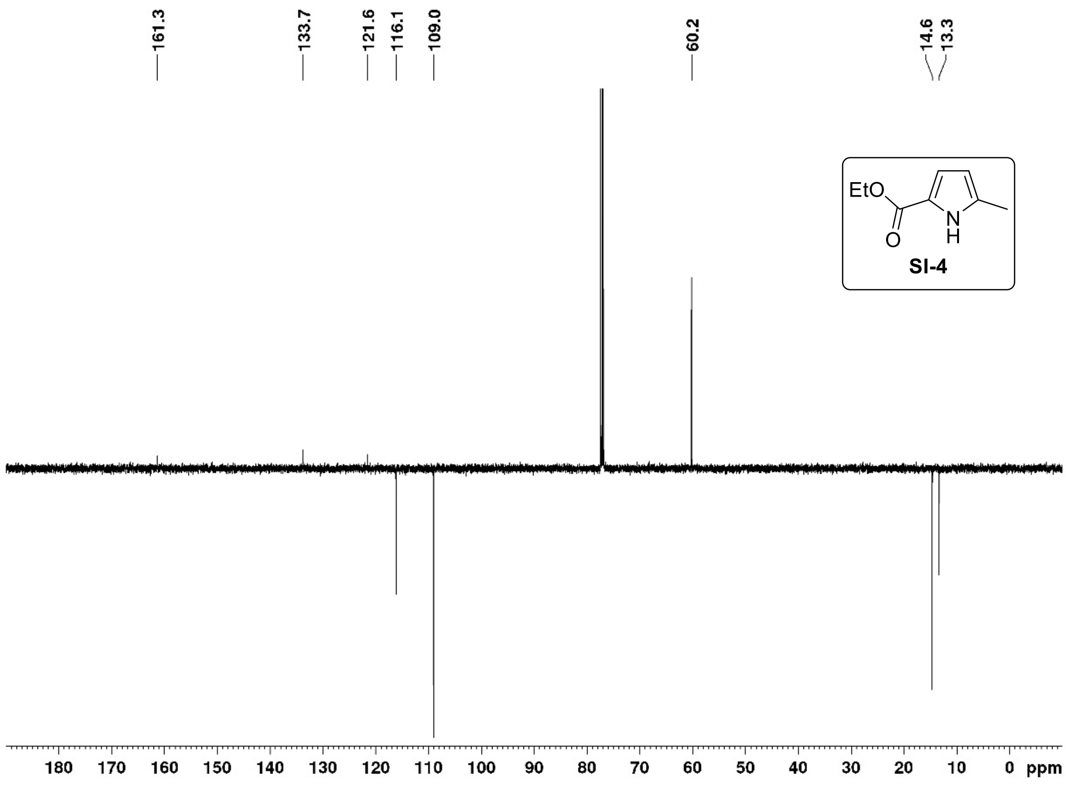
161.3
133.7
121.6
116.1
109.0
60.2
14.6
13.3
EtO
O
N
H
SI-4
180
170
160
150
140
130
120
110
100
90
80
70
60
50
40
30
20
10
0
ppm

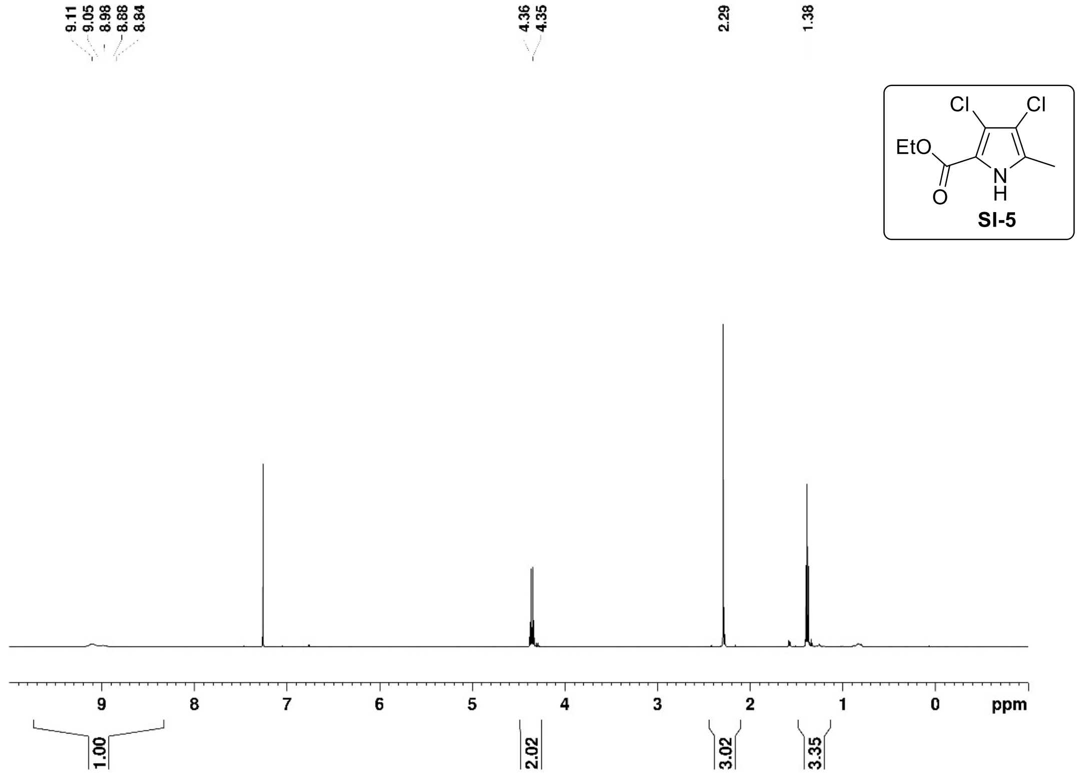
9.11
9.05
8.98
8.88
8.84
4.36
4.35
2.29
1.38
Cl
Cl
EtO
O
N
H
SI-5
9
8
7
6
5
4
3
2
1
0
ppm
1.00
2.02
3.02
3.35

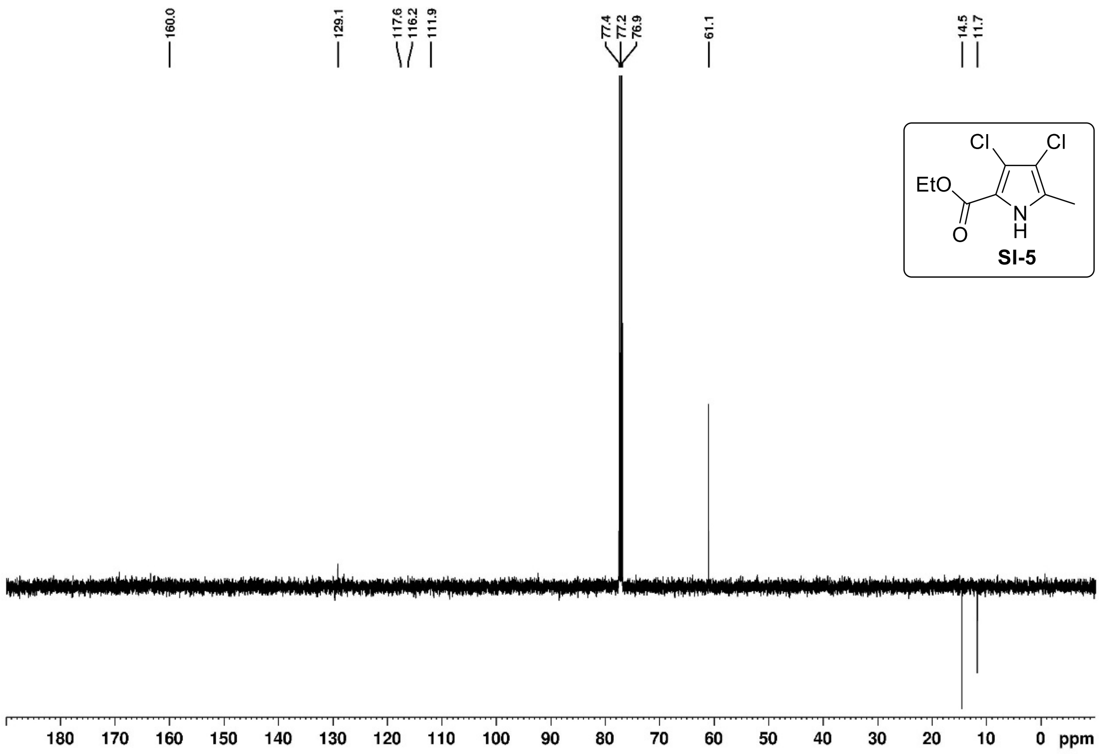
160.0
129.1
117.6
116.2
111.9
77.4
77.2
76.9
61.1
14.5
11.7
Cl
Cl
EtO
O
N
H
SI-5
180
170
160
150
140
130
120
110
100
90
80
70
60
50
40
30
20
10
0
ppm

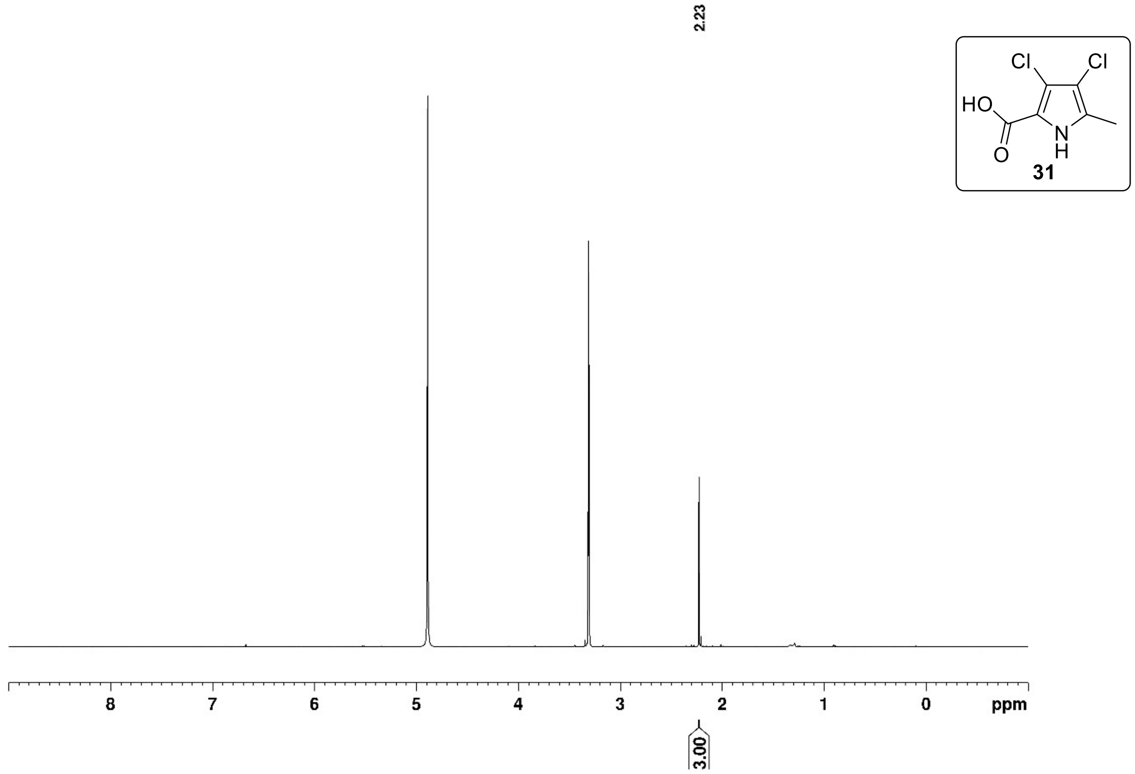
2.23
Cl
Cl
HO
O
N
H
31
8
7
6
5
4
3
2
1
0
ppm
3.00

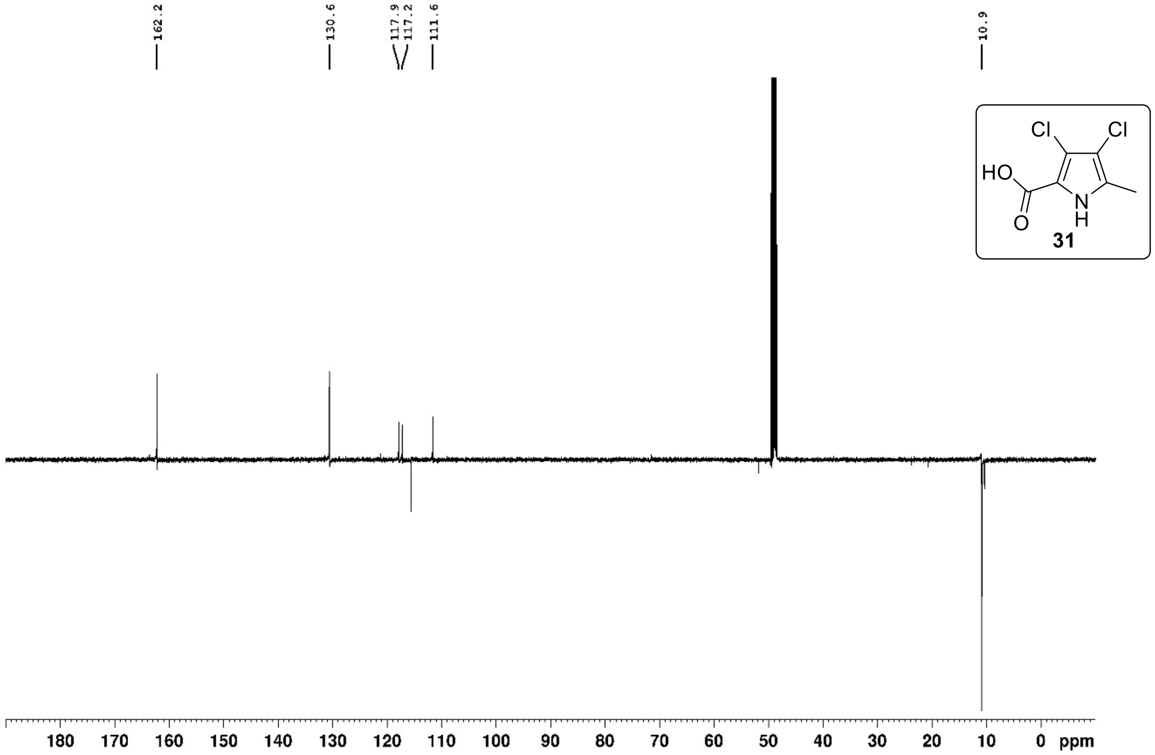
162.2
130.6
117.9
117.2
111.6
10.9
Cl
Cl
HO
O
N
H
31
180
170
160
150
140
130
120
110
100
90
80
70
60
50
40
30
20
10
0
ppm

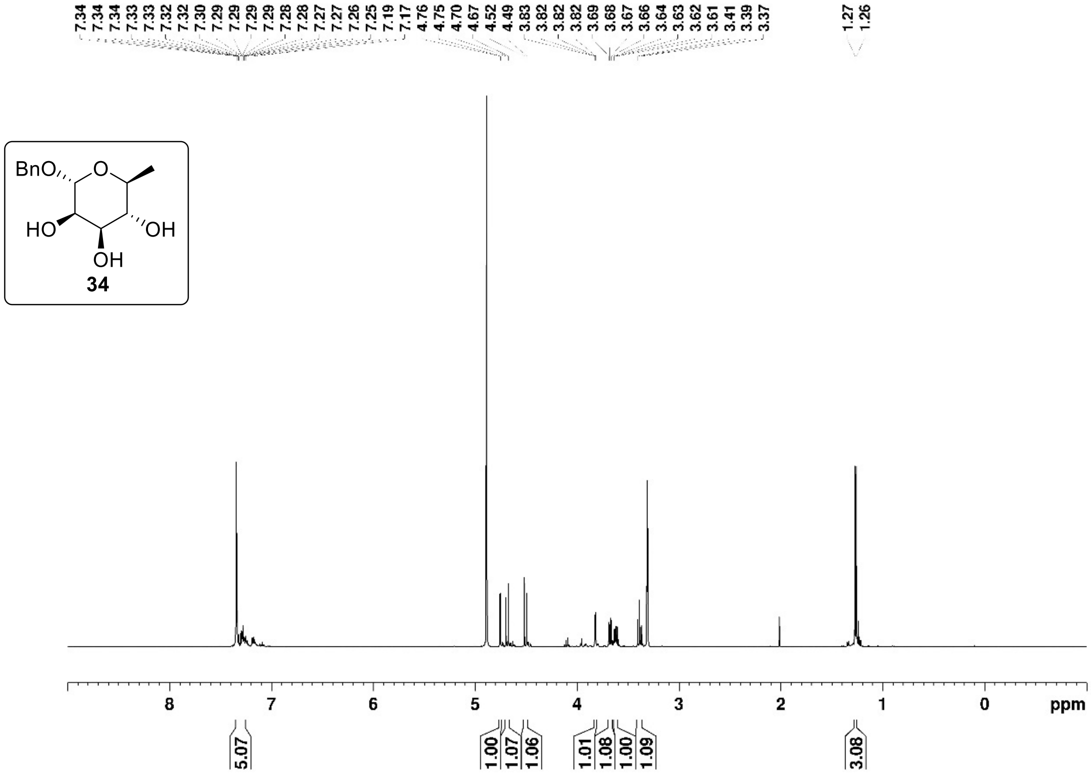
7.34 7.34 7.34 7.33 7.33 7.32 7.32 7.30 7.29 7.29 7.29 7.29 7.28 7.28 7.27 7.27 7.26 7.25 7.19 7.17 4.76 4.75 4.70 4.67 4.52 4.49 3.83 3.82 3.82 3.82 3.69 3.68 3.67 3.66 3.64 3.63 3.62 3.61 3.41 3.39 3.37
1.27 1.26
BnO
O
HO
OH
OH
34
8 7 6 5 4 3 2 1 0 ppm
5.07
1.00 1.07 1.06
1.01 1.08 1.00 1.09
3.08

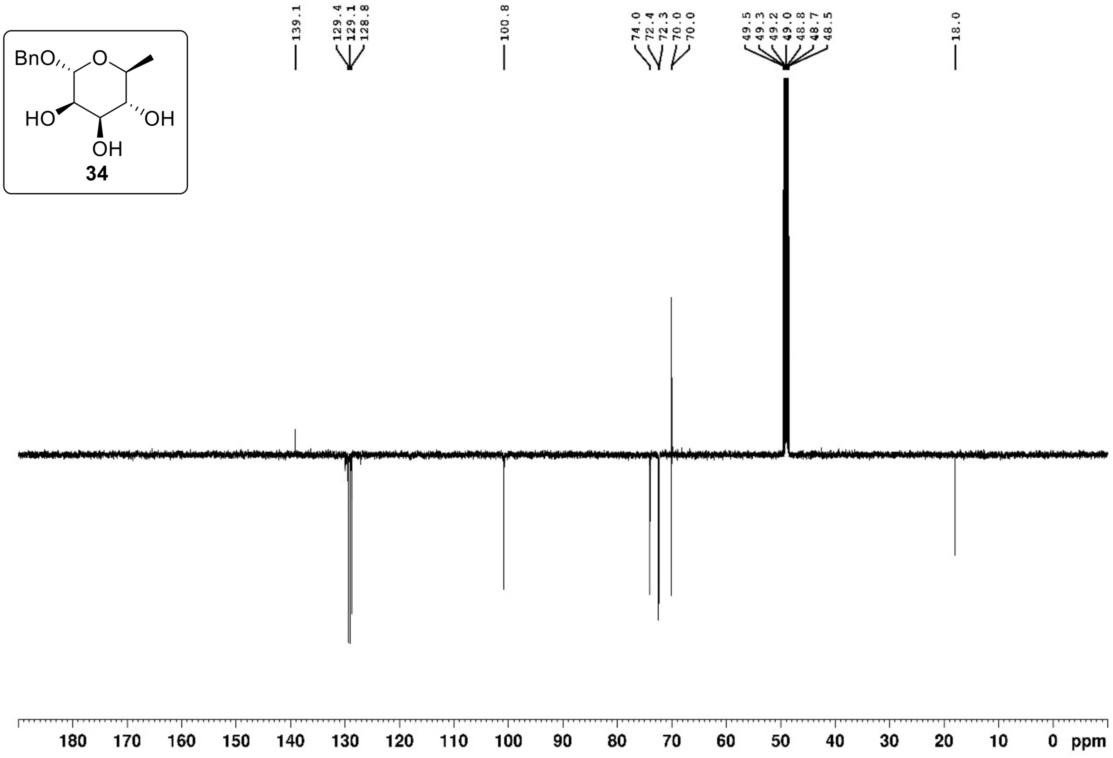
139.1
129.4 129.1 128.8
100.8
74.0 72.4 72.3 70.0 70.0
49.5 49.3 49.2 49.0 48.8 48.7 48.5
18.0
BnO
O
HO
OH
OH
34
180 170 160 150 140 130 120 110 100 90 80 70 60 50 40 30 20 10 0 ppm

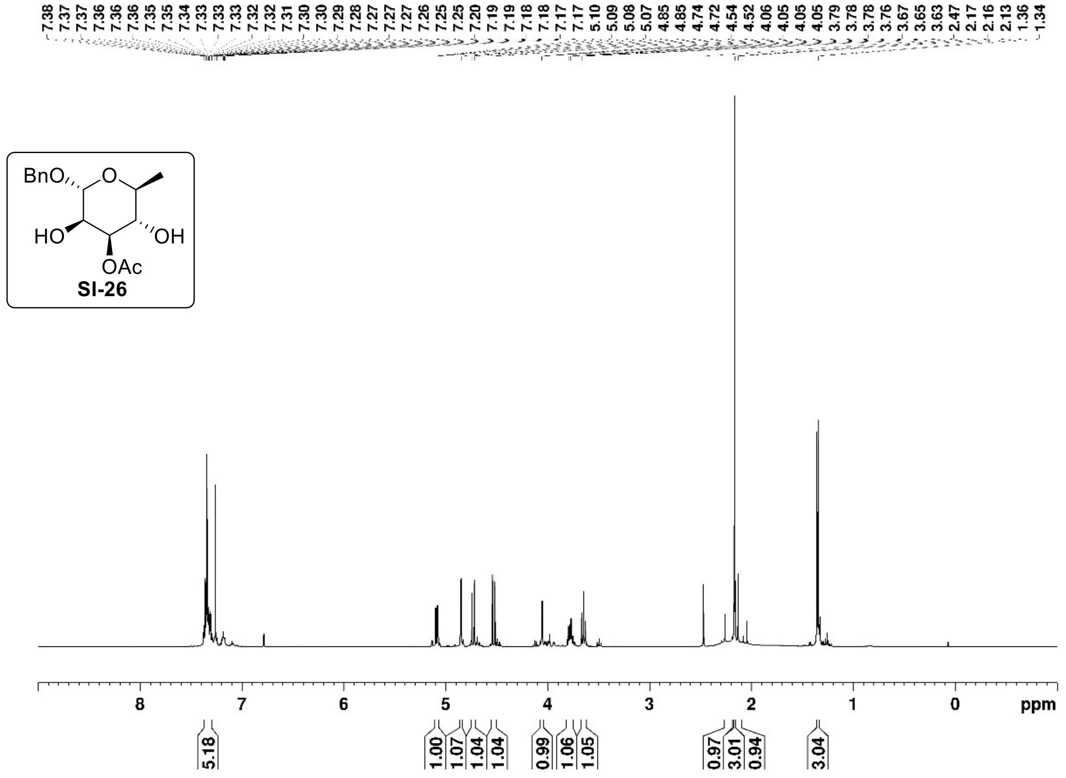
7.38 7.37 7.37 7.36 7.36 7.36 7.35 7.35 7.34 7.33 7.33 7.33 7.32 7.32 7.31 7.30 7.30 7.29 7.28 7.27 7.27 7.27 7.26 7.25 7.25 7.20 7.19 7.19 7.18 7.18 7.17 7.17 5.10 5.09 5.08 5.07 4.85 4.85 4.74 4.72 4.54 4.52 4.06 4.05 4.05 4.05 3.79 3.78 3.78 3.76 3.67 3.65 3.63 2.47 2.17 2.16 2.13 1.36 1.34
BnO
O
HO
OH
OAc
SI-26
ppm
5.18
1.00
1.07
1.04
1.04
0.99
1.06
1.05
0.97
3.01
0.94
3.04

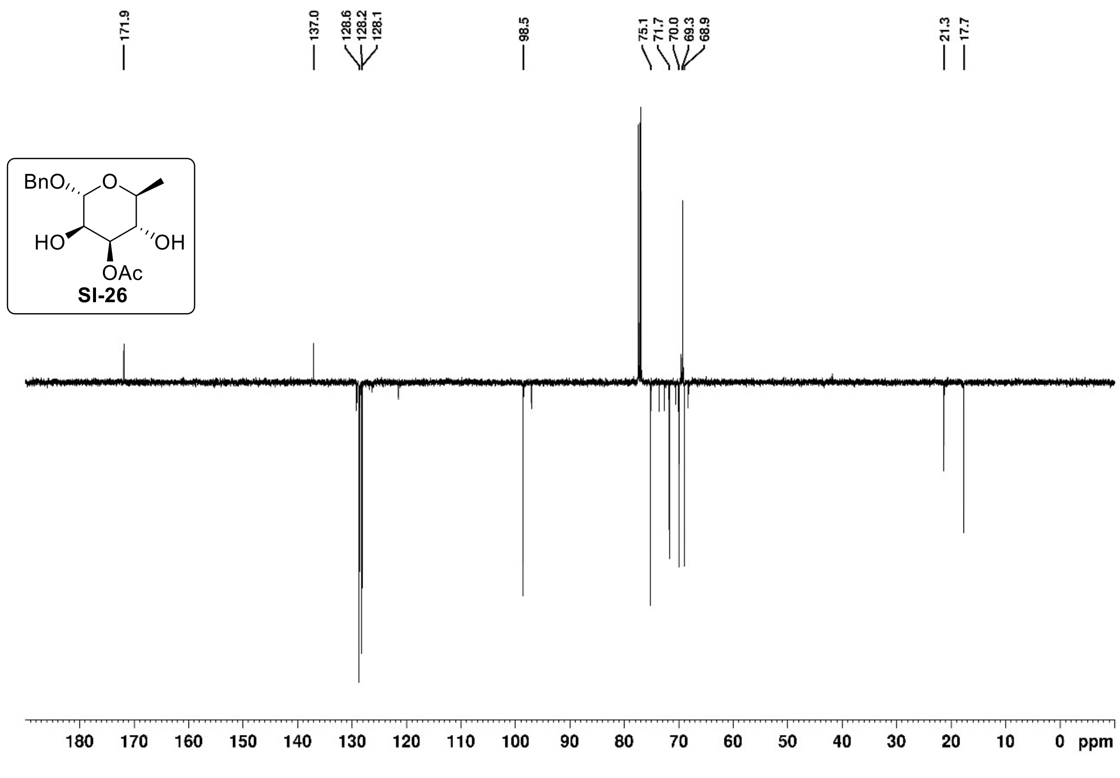
171.9
137.0
128.6
128.2
128.1
98.5
75.1
71.7
70.0
69.3
68.9
21.3
17.7
BnO
O
HO
OH
OAc
SI-26
ppm

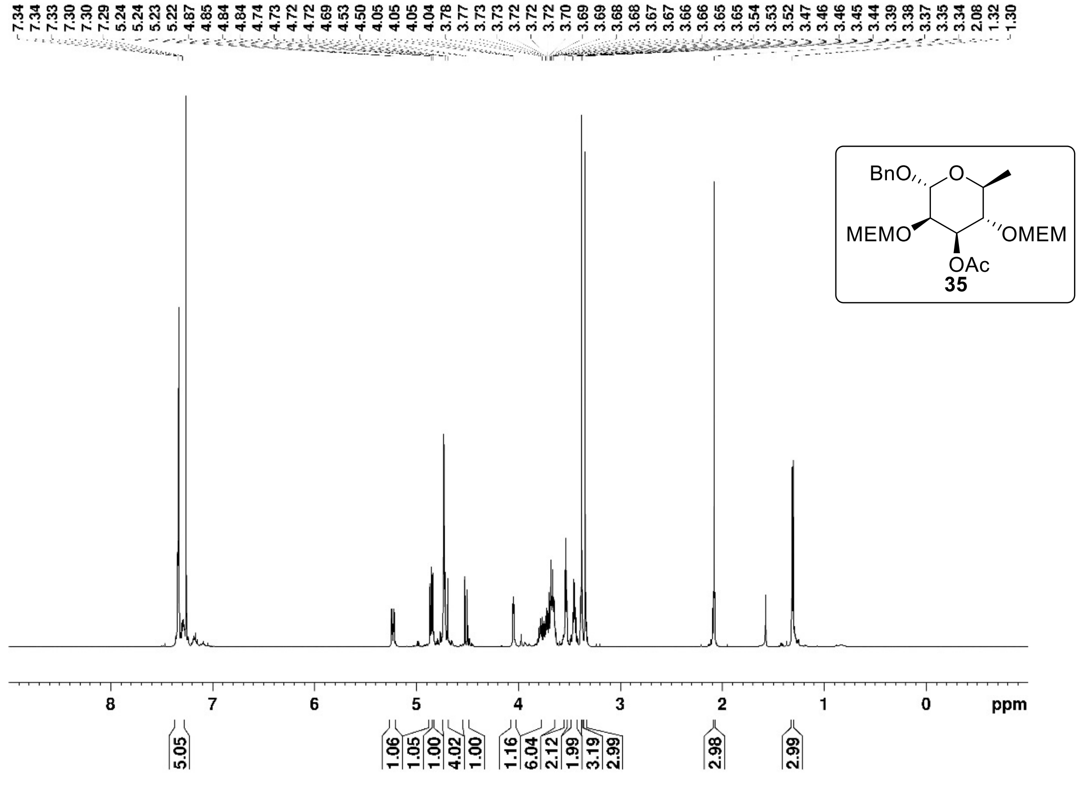

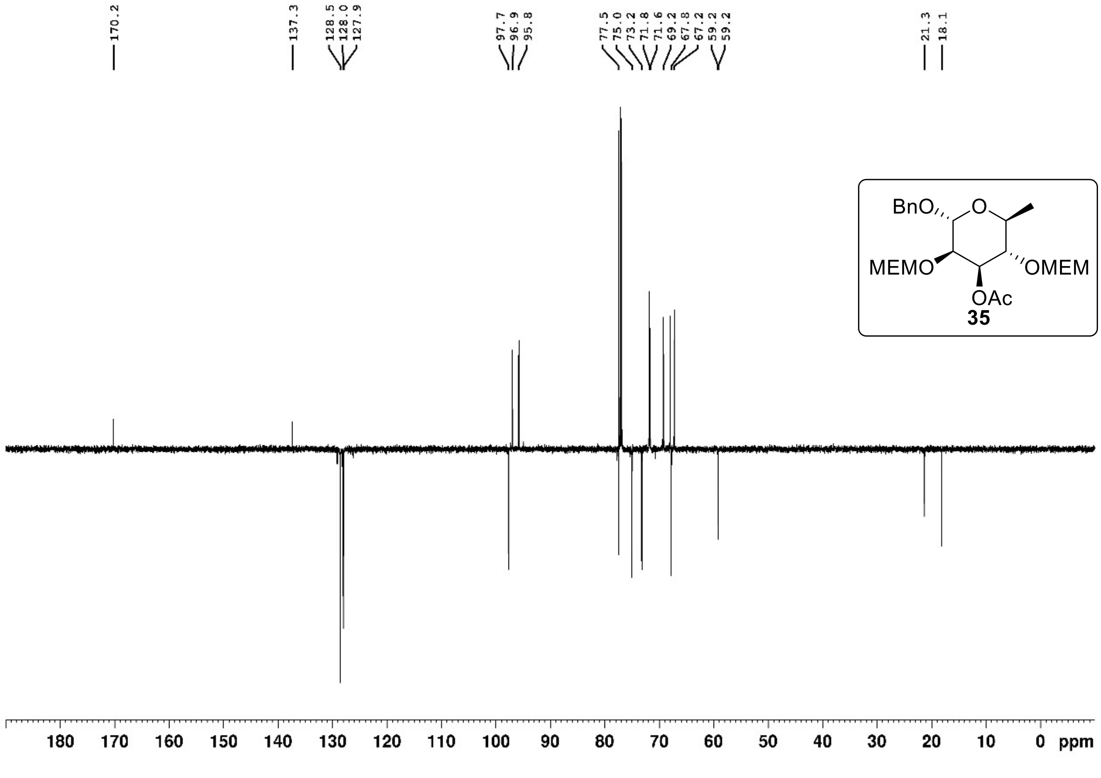

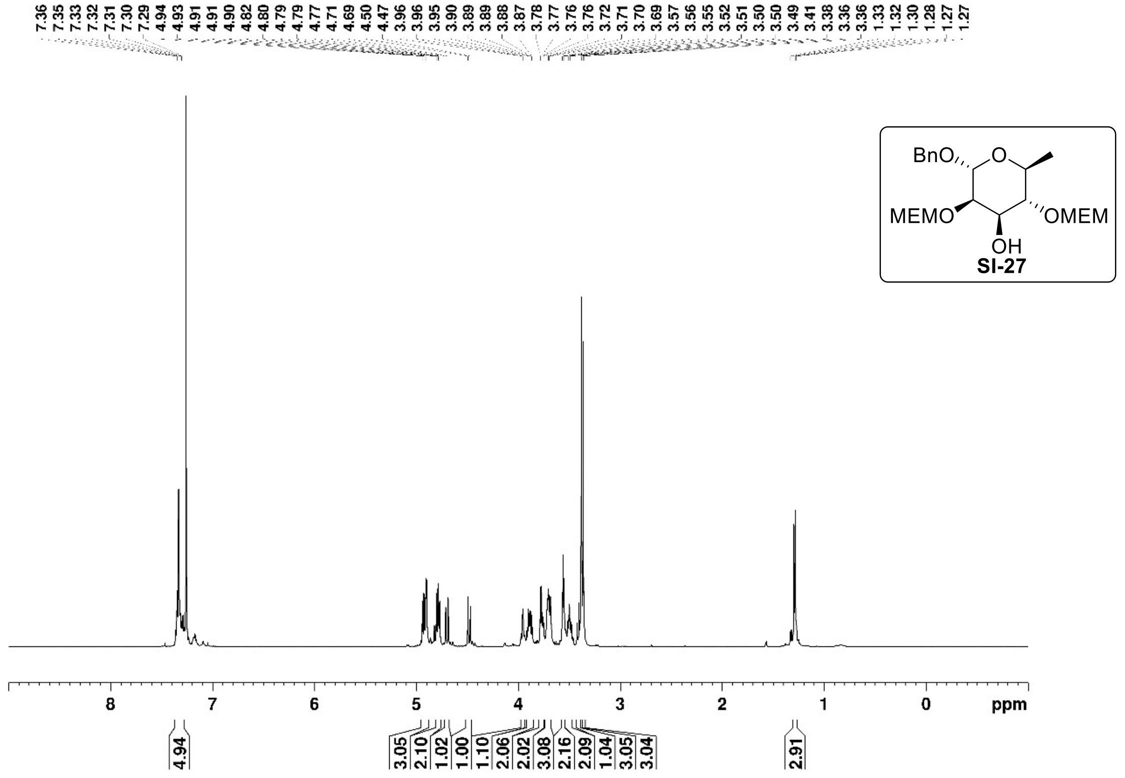
BnO
O
MEMO
OMEM
OH
SI-27
ppm

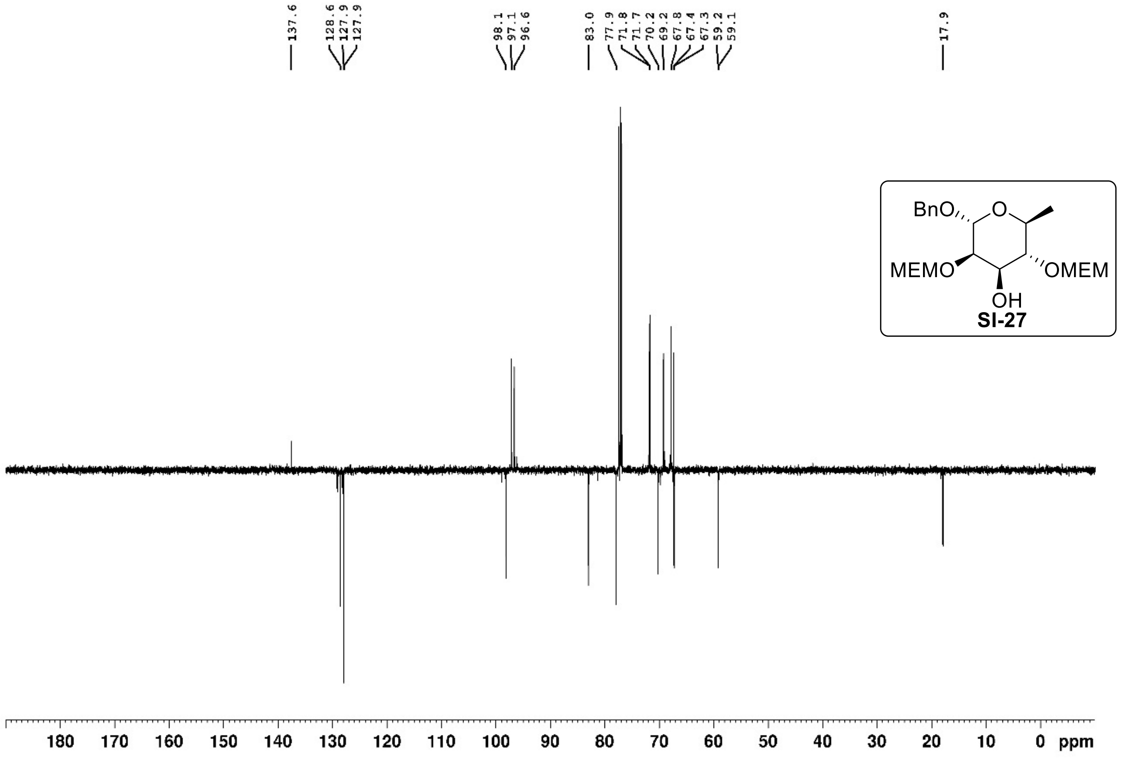
BnO
O
MEMO
OMEM
OH
SI-27
ppm

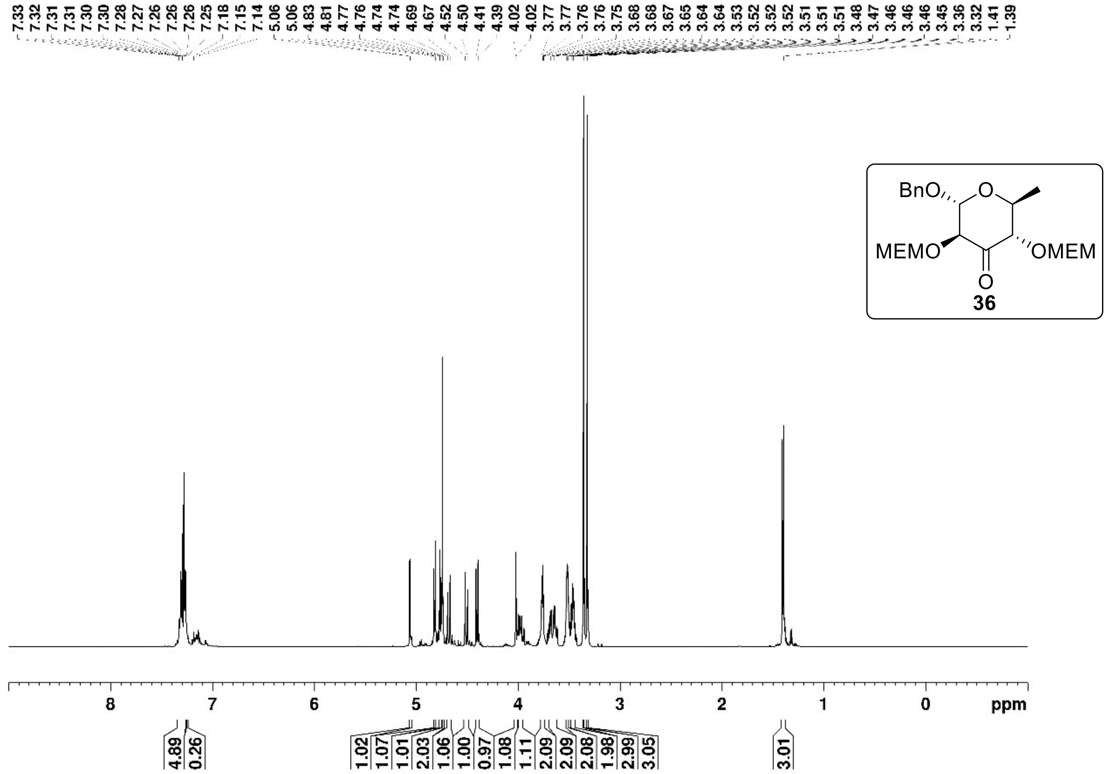
BnO
O
MEMO
OMEM
O
36
ppm

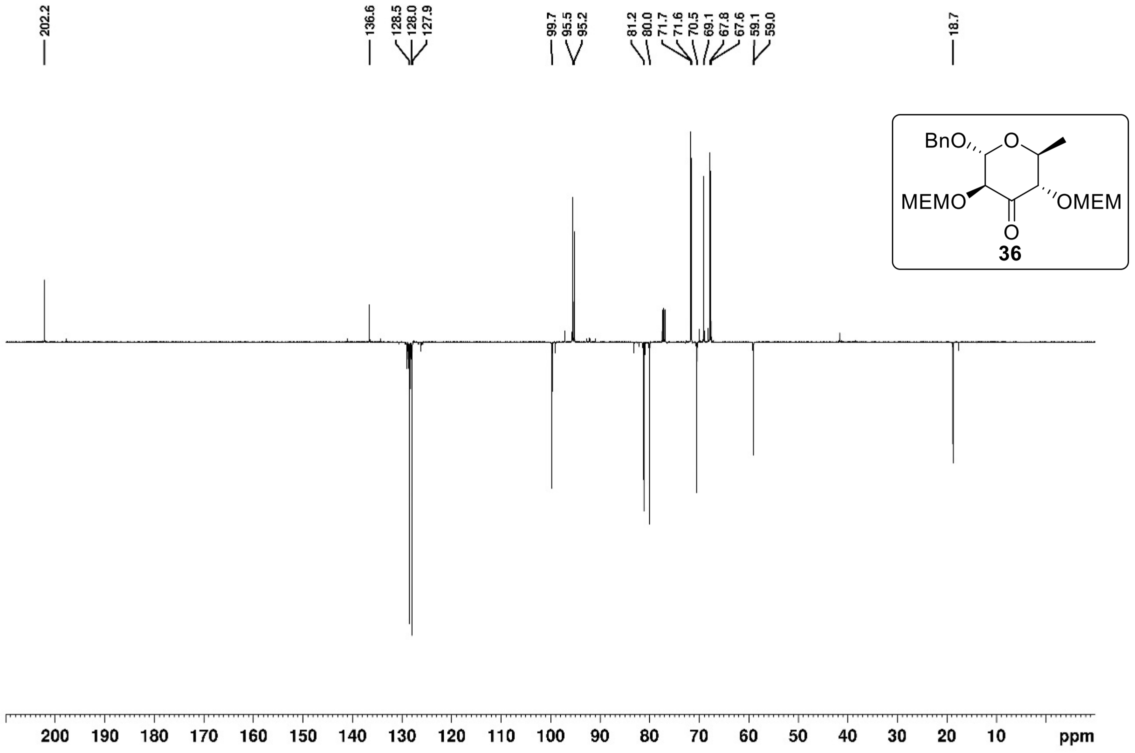
BnO
O
MEMO
OMEM
O
36
ppm

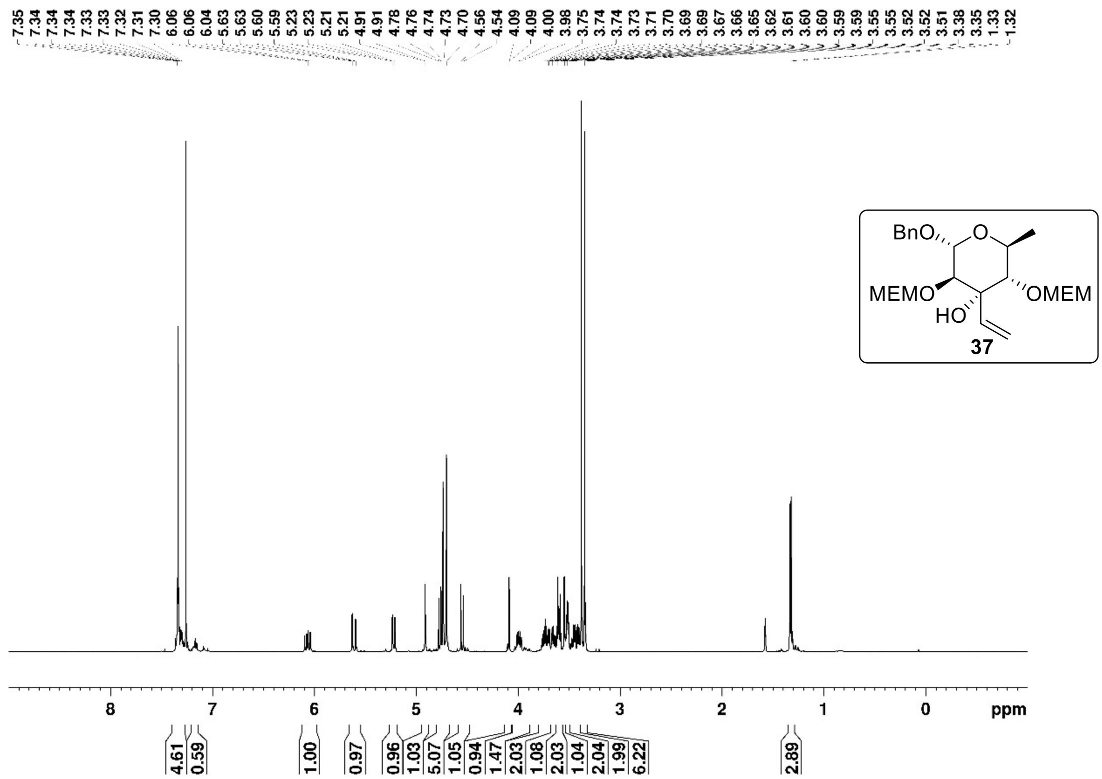
BnO
O
MEMO
OMEM
HO
37
ppm

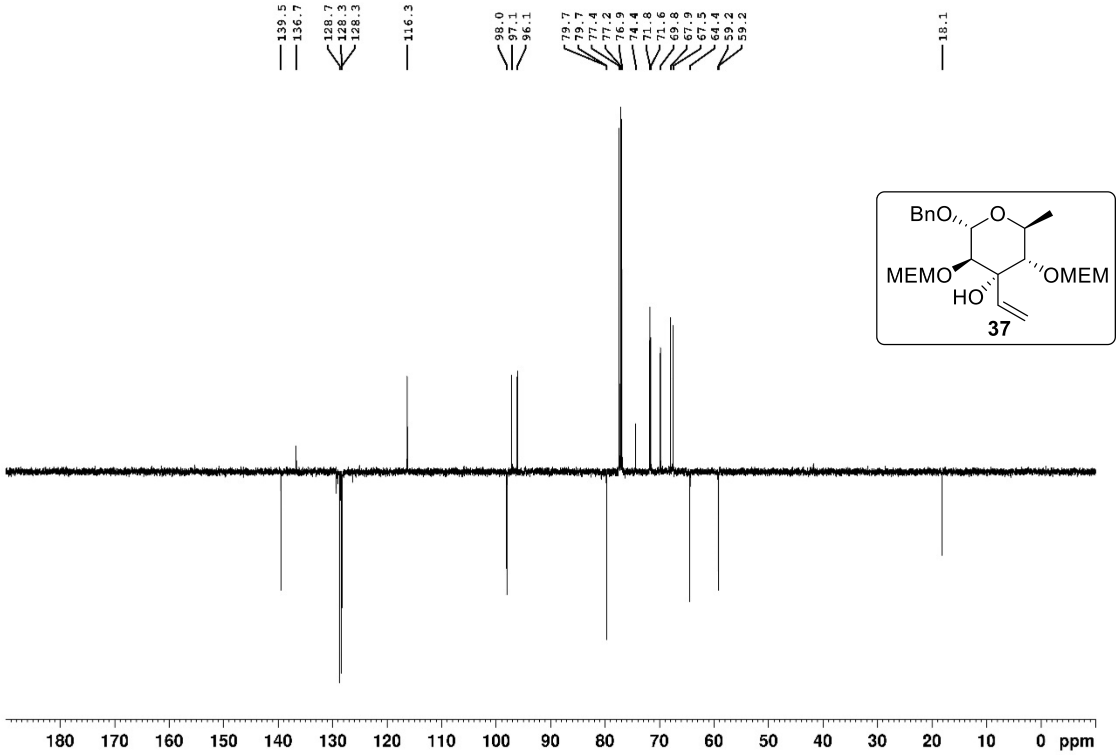
BnO
O
MEMO
OMEM
HO
37
ppm

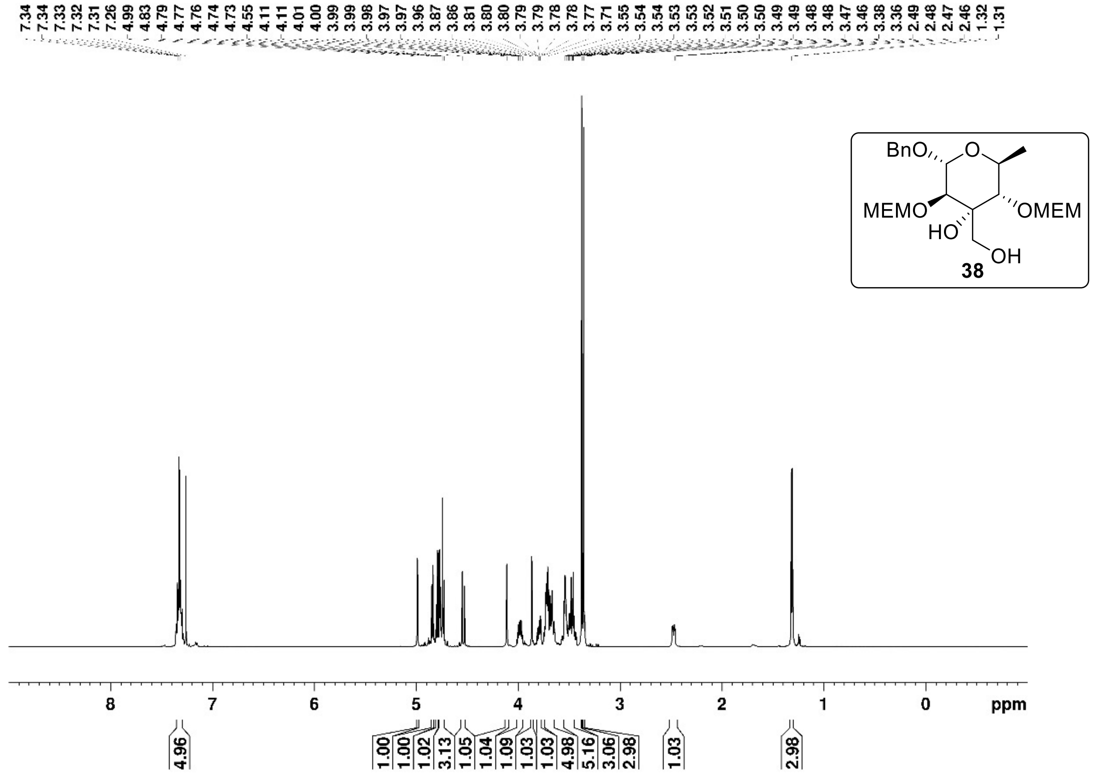
7.34 7.34 7.33 7.32 7.31 7.26 4.99 4.83 4.79 4.77 4.76 4.74 4.73 4.55 4.11 4.11 4.01 4.00 3.99 3.99 3.98 3.97 3.97 3.96 3.87 3.86 3.81 3.80 3.80 3.79 3.79 3.78 3.78 3.77 3.71 3.55 3.54 3.54 3.53 3.53 3.52 3.51 3.50 3.50 3.49 3.49 3.48 3.48 3.47 3.46 3.38 3.36 2.49 2.48 2.47 2.46 1.32 1.31
BnO
O
MEMO
OMEM
HO
OH
38
8 7 6 5 4 3 2 1 0 ppm
4.96
1.00 1.00 1.02 3.13 1.05 1.04 1.09 1.03 1.03 4.98 5.16 3.06 2.98
1.03
2.98

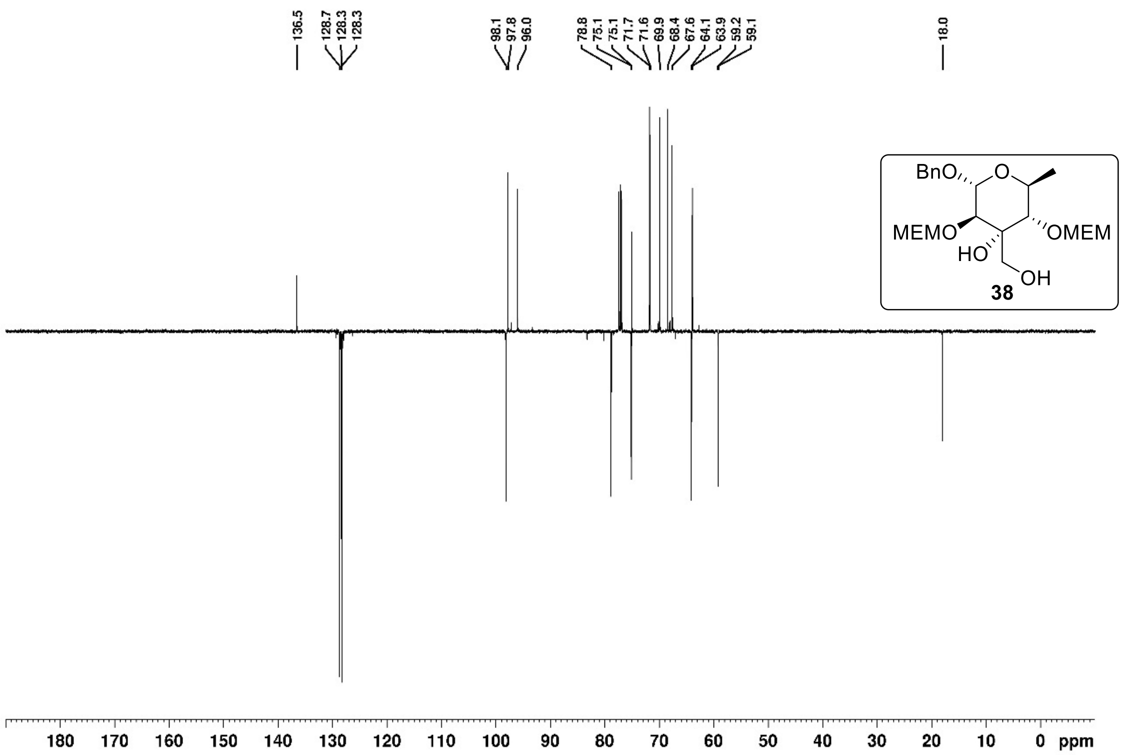
136.5
128.7 128.3 128.3
98.1 97.8 96.0
78.8 75.1 75.1 71.7 71.6 69.9 68.4 67.6 64.1 63.9 59.2 59.1
18.0
BnO
O
MEMO
OMEM
HO
OH
38
180 170 160 150 140 130 120 110 100 90 80 70 60 50 40 30 20 10 0 ppm

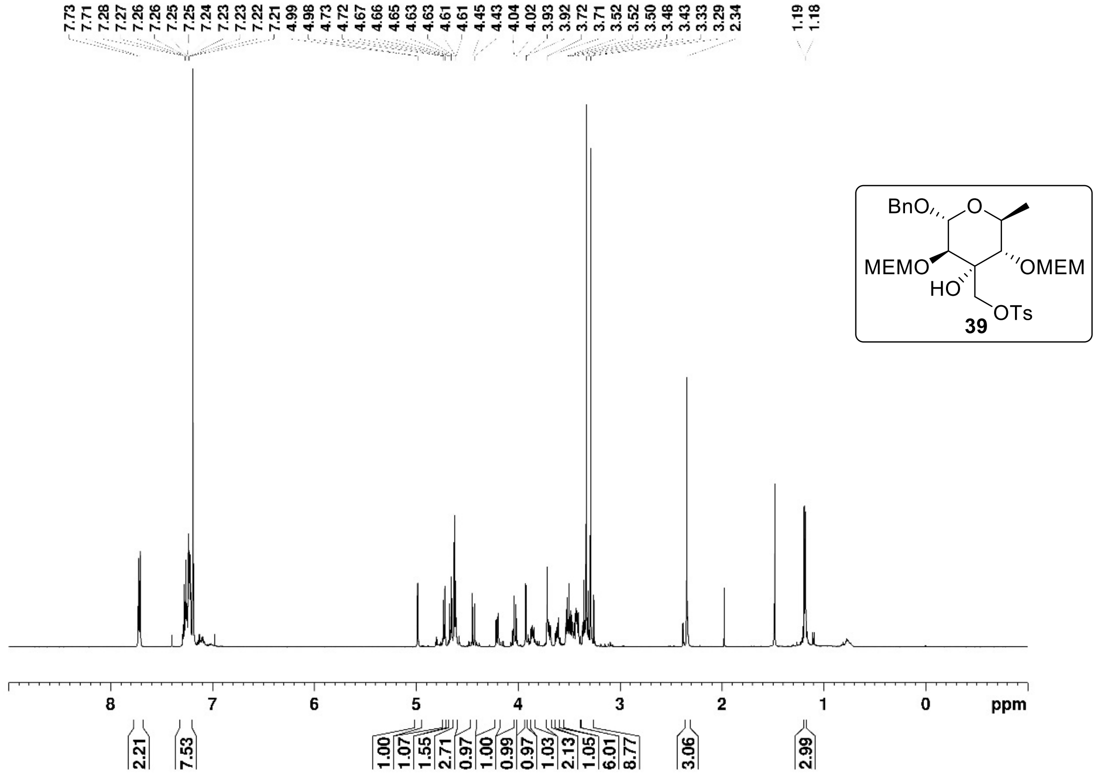

7.73
7.71
7.28
7.27
7.26
7.26
7.25
7.25
7.24
7.23
7.23
7.22
7.21
4.99
4.98
4.73
4.72
4.67
4.66
4.65
4.63
4.63
4.61
4.61
4.45
4.43
4.04
4.02
3.93
3.92
3.72
3.71
3.52
3.52
3.50
3.48
3.43
3.33
3.29
2.34
1.19
1.18
BnO
O
MEMO
OMEM
HO
OTs
39
8
7
6
5
4
3
2
1
0
ppm
2.21
7.53
1.00
1.07
1.55
2.71
0.97
1.00
0.99
0.97
1.03
2.13
1.05
6.01
8.77
3.06
2.99

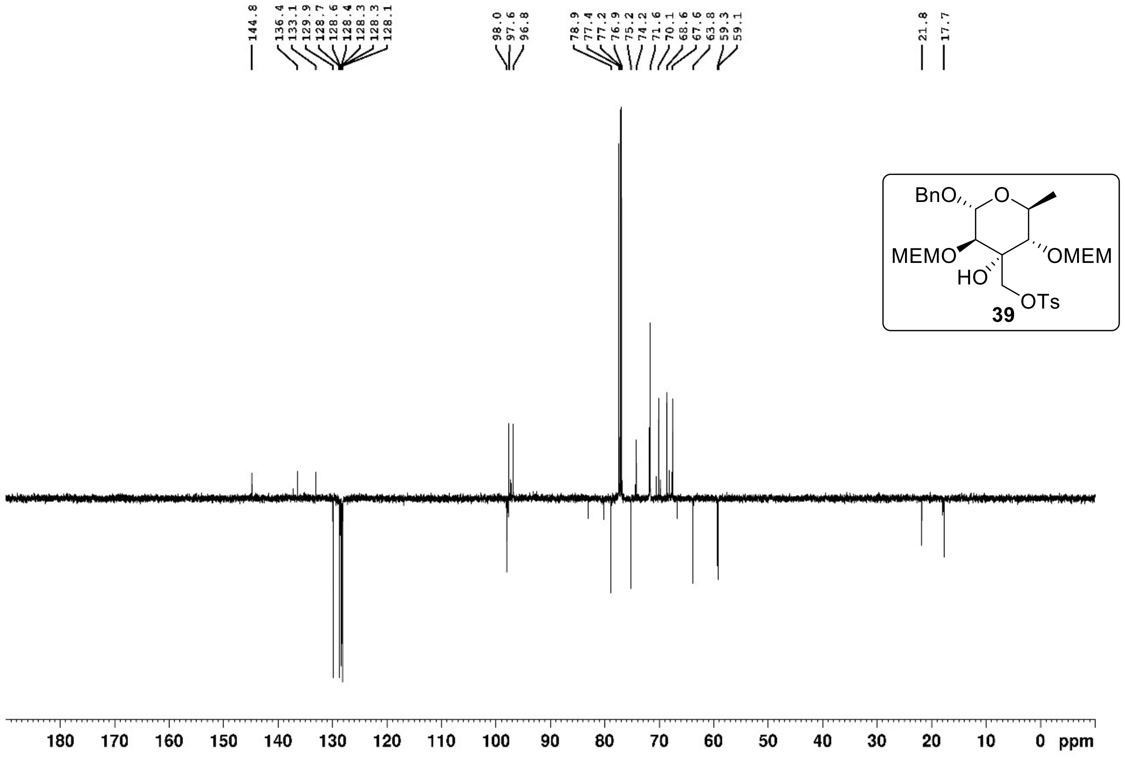

144.8
136.4
133.1
129.9
128.7
128.6
128.4
128.3
128.3
128.1
98.0
97.6
96.8
78.9
77.4
77.2
76.9
75.2
74.2
71.6
70.1
68.6
67.6
63.8
59.3
59.1
21.8
17.7
BnO
O
MEMO
OMEM
HO
OTs
39
180
170
160
150
140
130
120
110
100
90
80
70
60
50
40
30
20
10
0
ppm

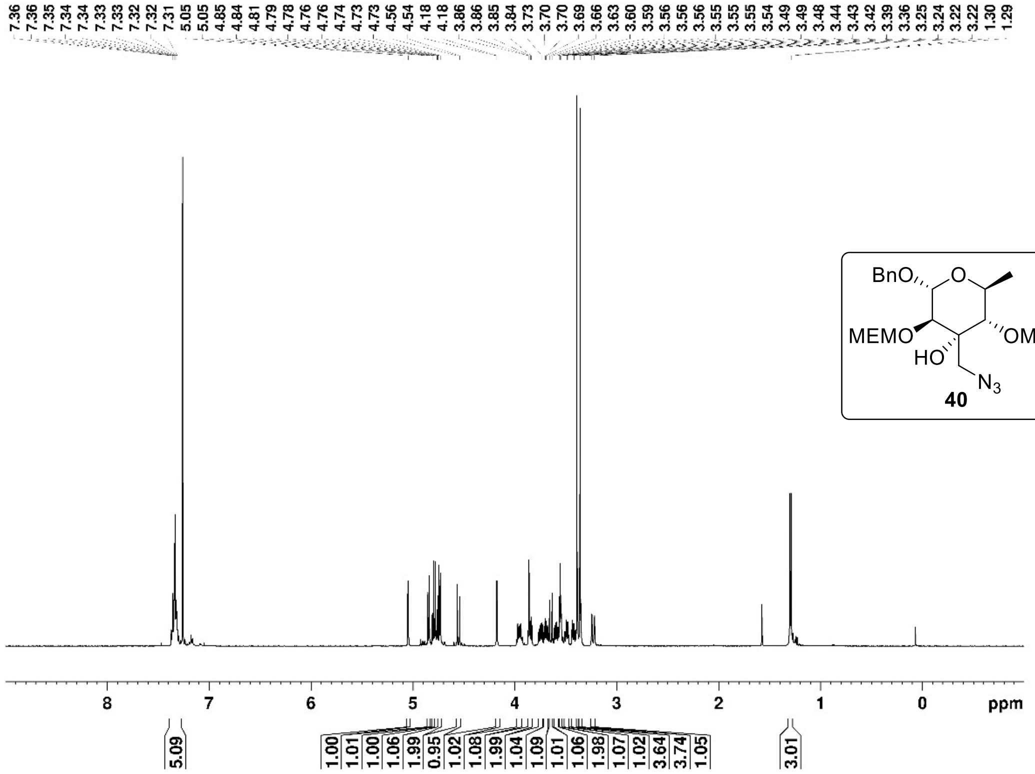
7.36 7.36 7.35 7.34 7.34 7.33 7.33 7.32 7.32 7.31 5.05 5.05 4.85 4.84 4.81 4.79 4.78 4.76 4.76 4.74 4.73 4.73 4.56 4.54 4.18 4.18 3.86 3.86 3.85 3.84 3.73 3.70 3.70 3.69 3.66 3.63 3.60 3.59 3.56 3.56 3.56 3.55 3.55 3.55 3.54 3.49 3.49 3.48 3.44 3.43 3.42 3.39 3.36 3.25 3.24 3.22 3.22 1.30 1.29
BnO
O
MEMO
OMEM
HO
N3
40
8 7 6 5 4 3 2 1 0 ppm
5.09
1.00 1.01 1.00 1.06 1.99 0.95 1.02 1.08 1.99 1.04 1.09 1.01 1.06 1.98 1.07 1.02 3.64 3.74 1.05
3.01

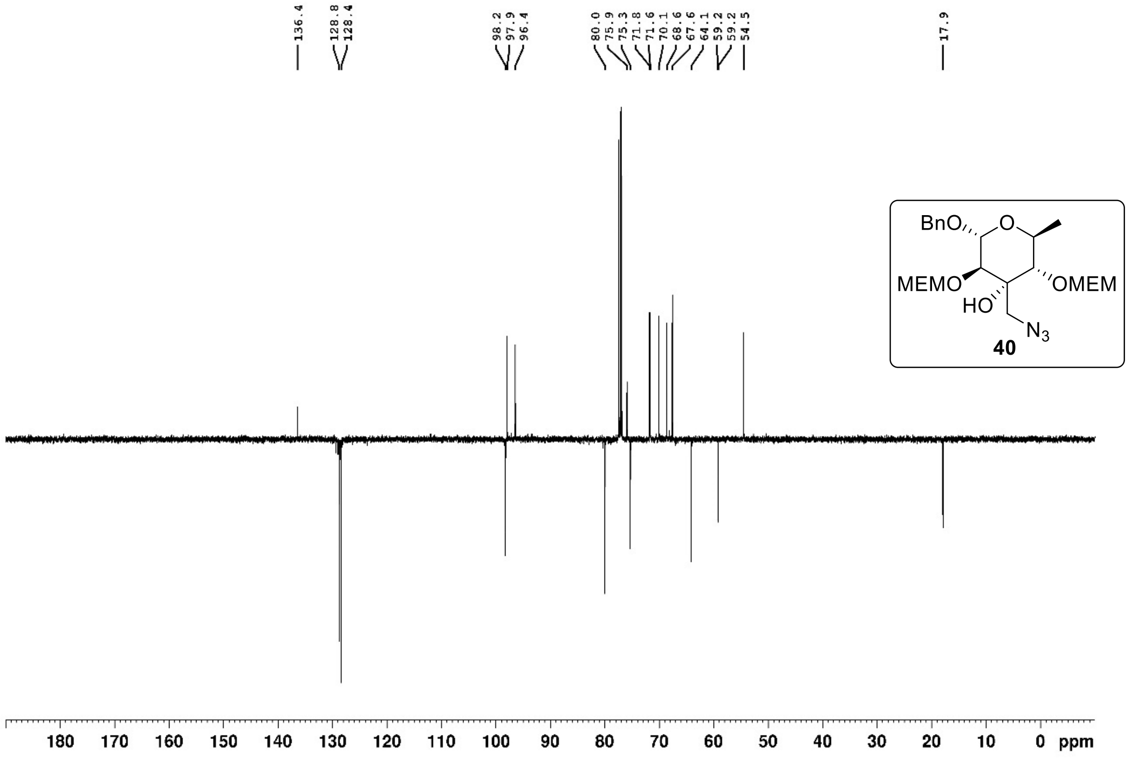
136.4
128.8 128.4
98.2 97.9 96.4
80.0 75.9 75.3 71.8 71.6 70.1 68.6 67.6 64.1 59.2 59.2 54.5
17.9
BnO
O
MEMO
OMEM
HO
N3
40
180 170 160 150 140 130 120 110 100 90 80 70 60 50 40 30 20 10 0 ppm

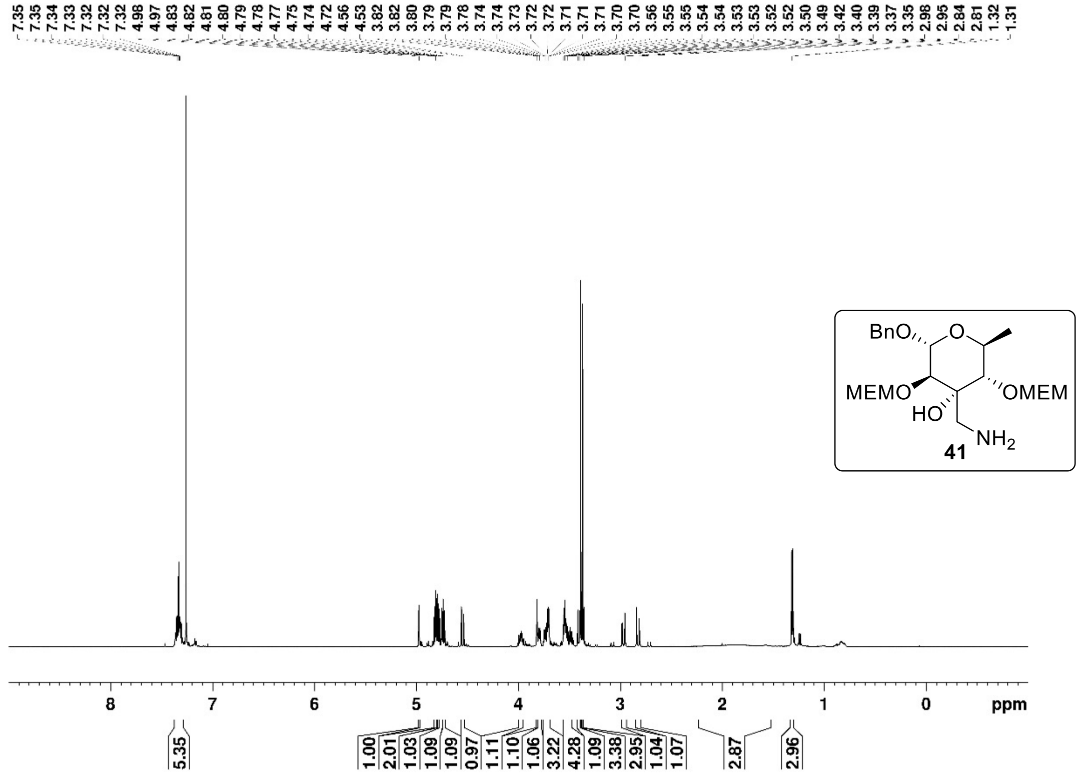
ppm
BnO
O
MEMO
OMEM
HO
NH2
41

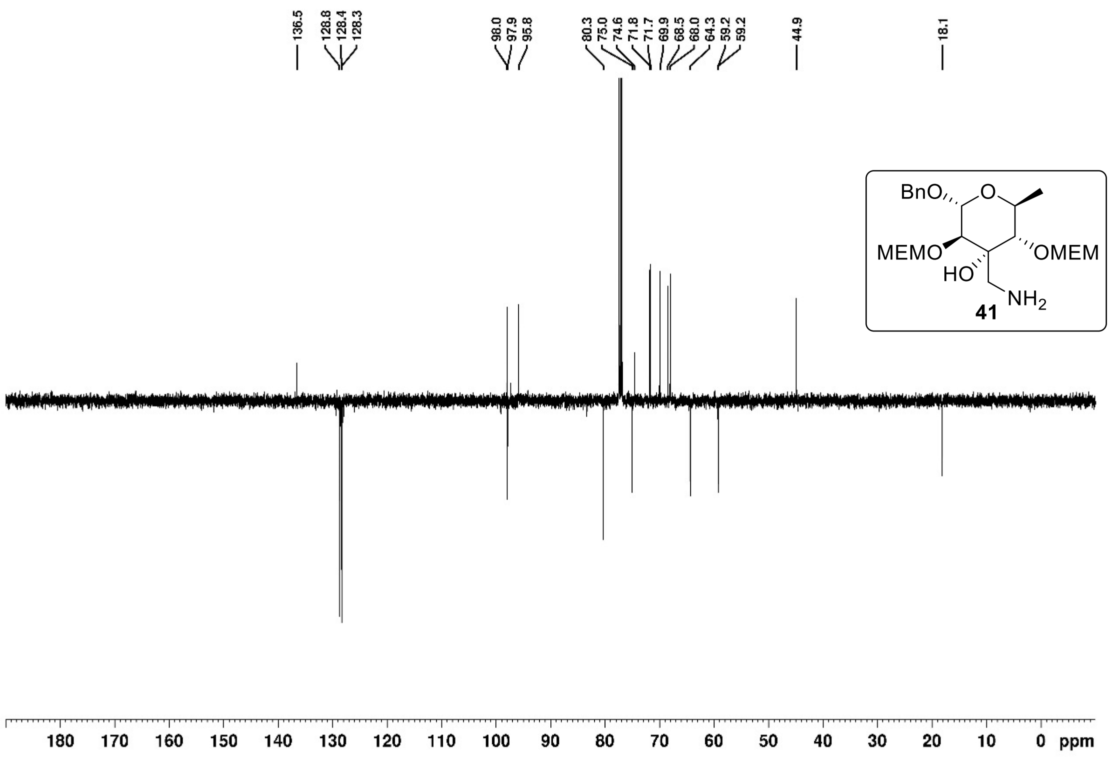
136.5
128.8
128.4
128.3
98.0
97.9
95.8
80.3
75.0
74.6
71.8
71.7
69.9
68.5
68.0
64.3
59.2
59.2
44.9
18.1
ppm
BnO
O
MEMO
OMEM
HO
NH2
41

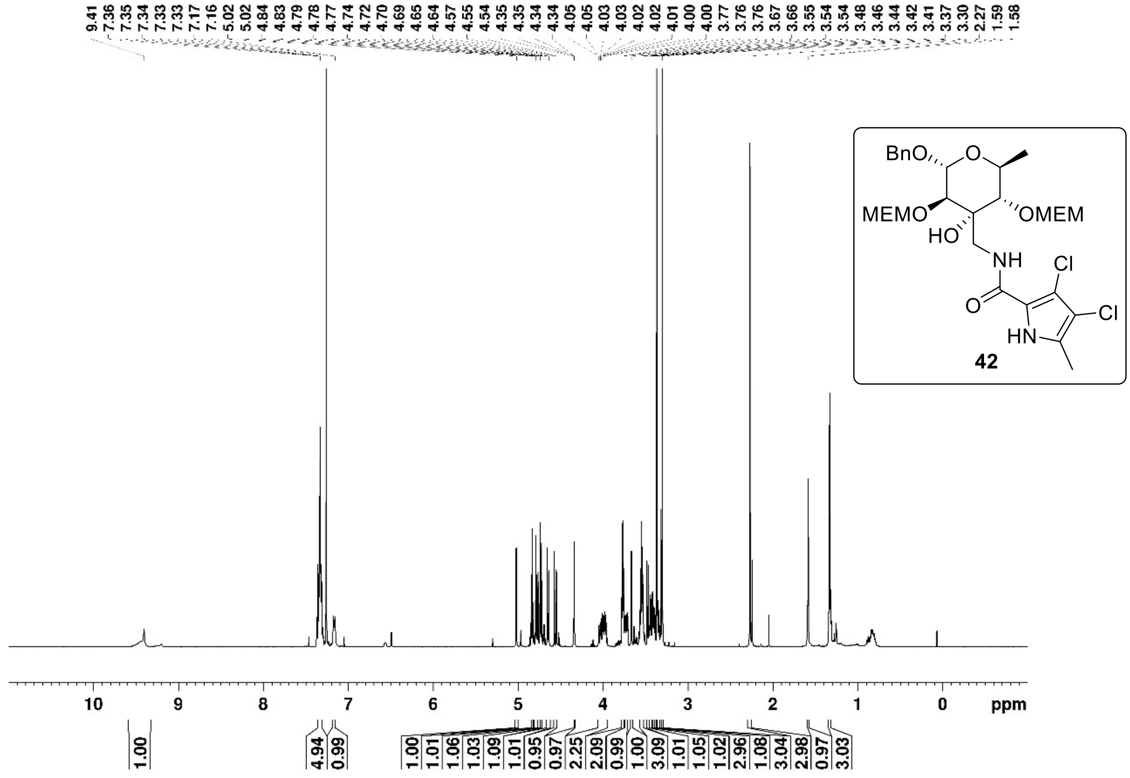

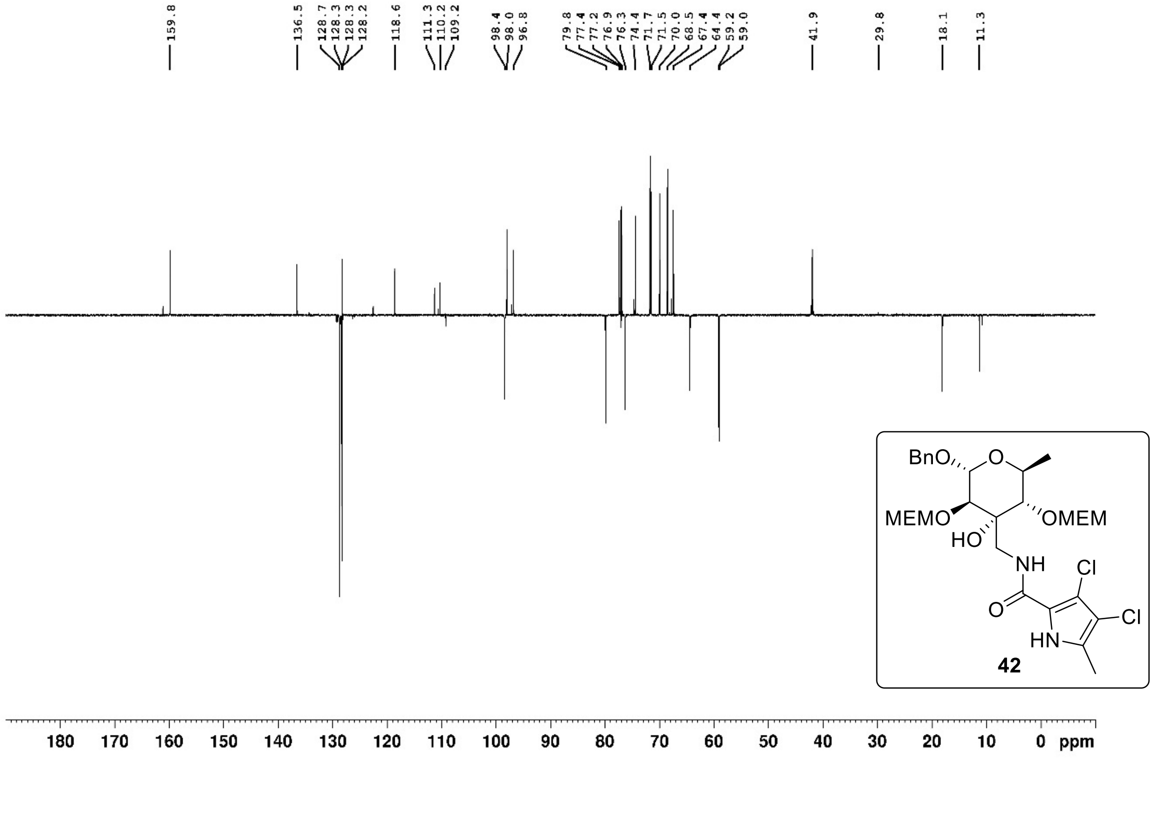

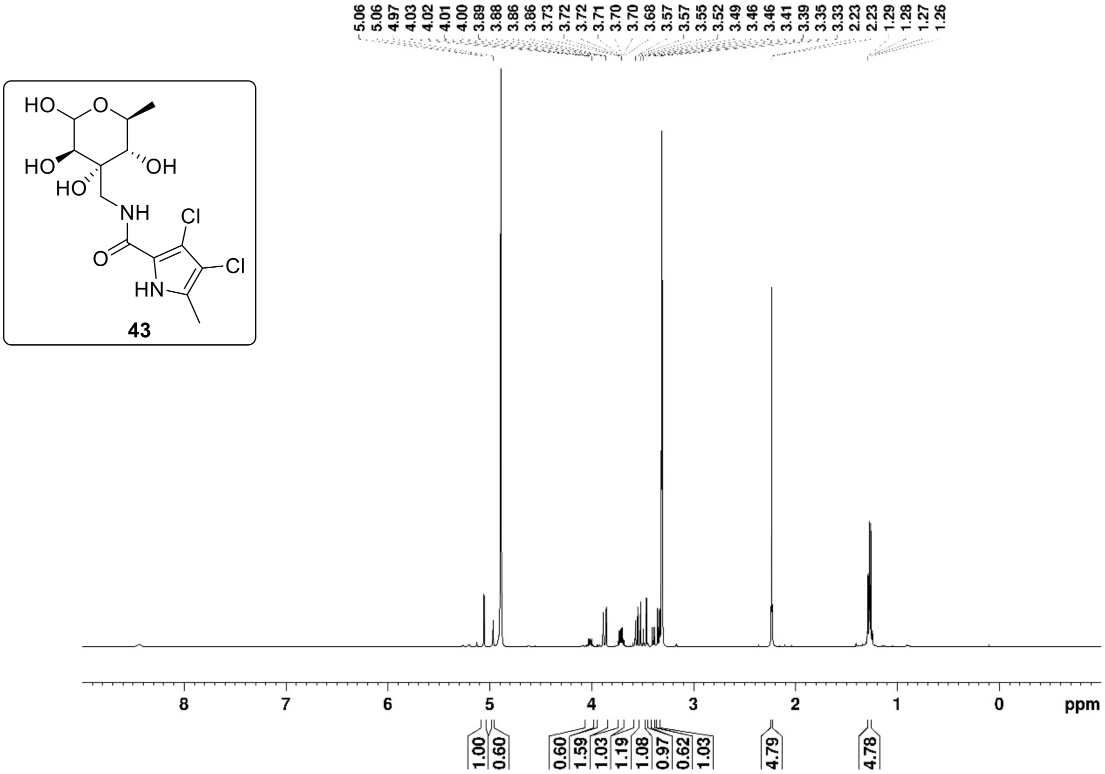
5.06 5.06 4.97 4.03 4.02 4.01 4.00 3.89 3.88 3.86 3.86 3.73 3.72 3.72 3.71 3.70 3.70 3.68 3.57 3.57 3.55 3.52 3.49 3.46 3.46 3.41 3.39 3.35 3.33 2.23 2.23 1.29 1.28 1.27 1.26
HO
O
HO
OH
HO
NH
Cl
O
Cl
HN
43
8 7 6 5 4 3 2 1 0 ppm
1.00 0.60 0.60 1.59 1.03 1.19 1.08 0.97 0.62 1.03 4.79 4.78

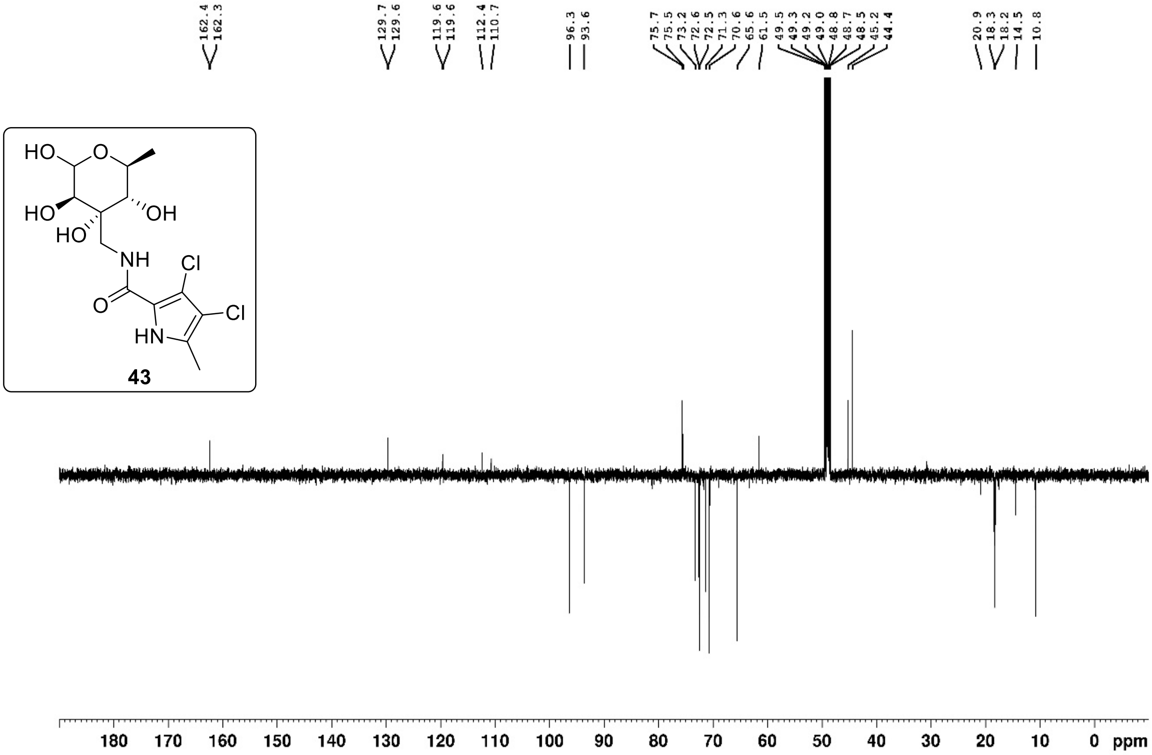
162.4 162.3 129.7 129.6 119.6 119.6 112.4 110.7 96.3 93.6 75.7 75.5 73.2 72.6 72.5 71.3 70.6 65.6 61.5 49.5 49.3 49.2 49.0 48.8 48.7 48.5 45.2 44.4 20.9 18.3 18.2 14.5 10.8
HO
O
HO
OH
HO
NH
Cl
O
Cl
HN
43
180 170 160 150 140 130 120 110 100 90 80 70 60 50 40 30 20 10 0 ppm

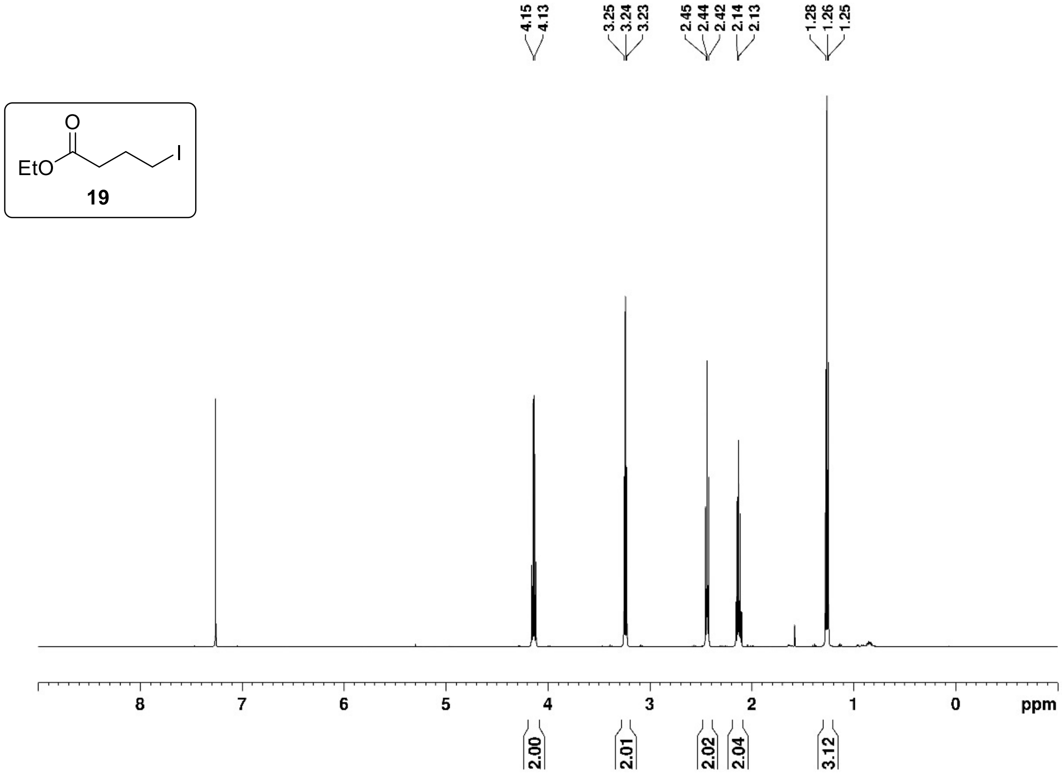
EtO
I
19
4.15
4.13
3.25
3.24
3.23
2.45
2.44
2.42
2.14
2.13
1.28
1.26
1.25
ppm
2.00
2.01
2.02
2.04
3.12

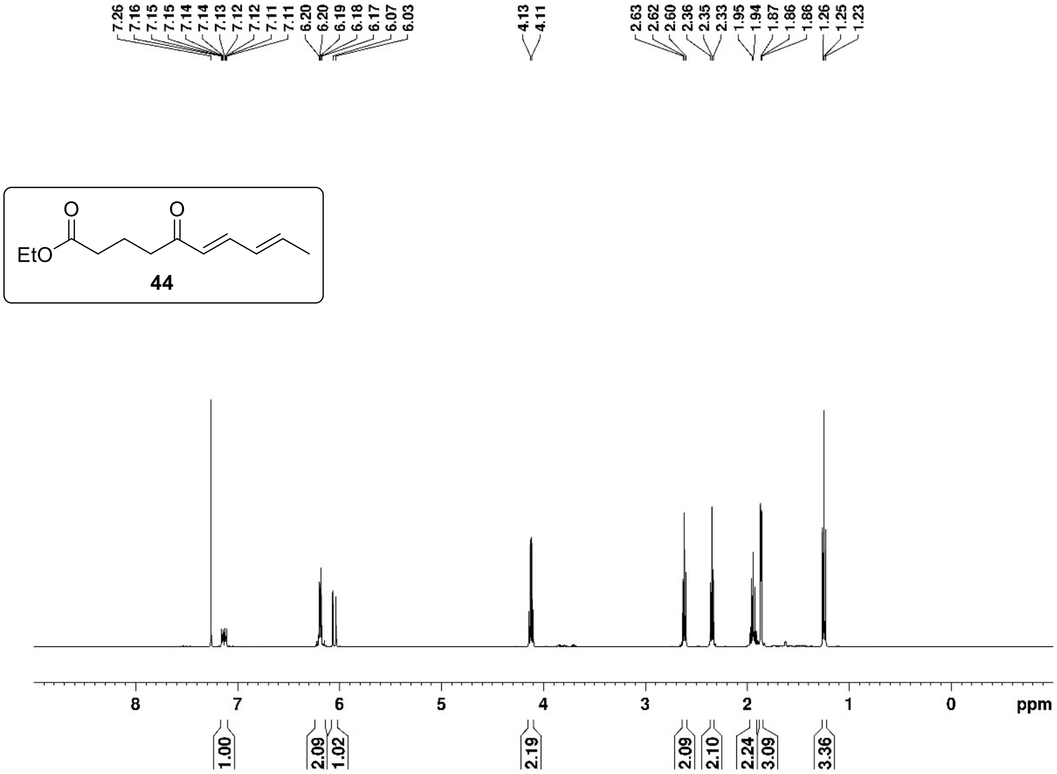
EtO
44
7.26
7.16
7.15
7.15
7.14
7.14
7.13
7.12
7.12
7.11
7.11
6.20
6.20
6.19
6.18
6.17
6.07
6.03
4.13
4.11
2.63
2.62
2.60
2.36
2.35
2.33
1.95
1.94
1.87
1.86
1.86
1.26
1.25
1.23
ppm
1.00
2.09
1.02
2.19
2.09
2.10
2.24
3.09
3.36

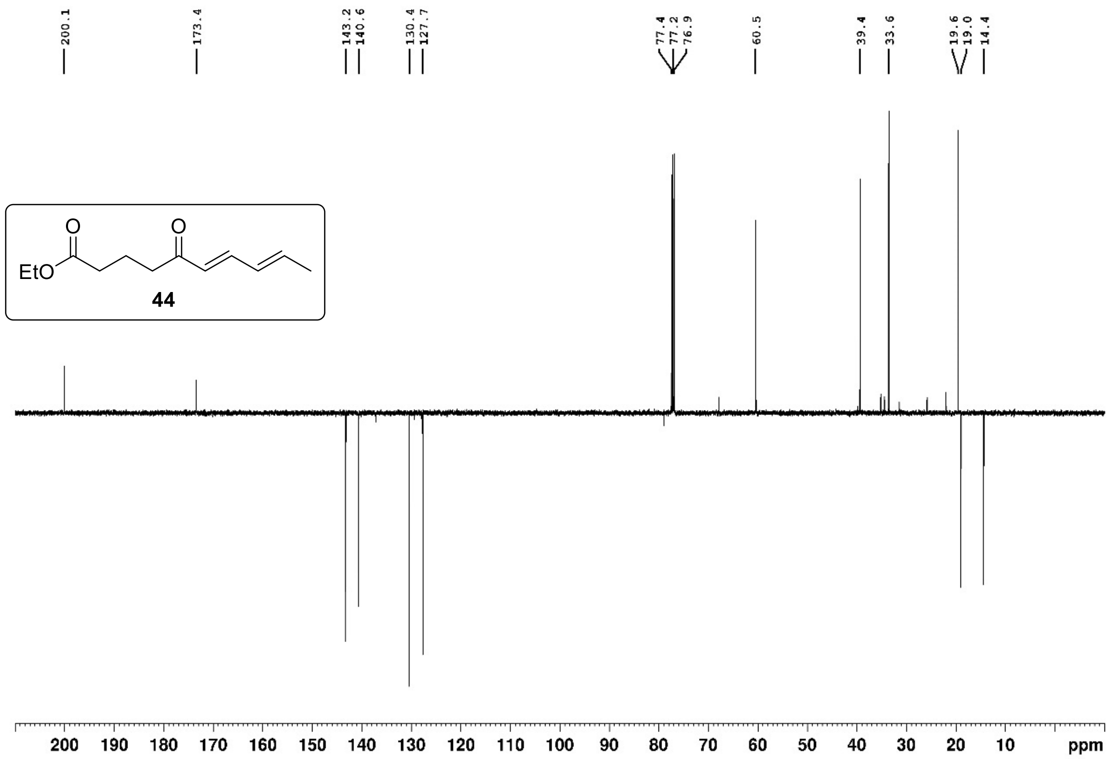
200.1
173.4
143.2
140.6
130.4
127.7
77.4
77.2
76.9
60.5
39.4
33.6
19.6
19.0
14.4
EtO
O
O
44
200 190 180 170 160 150 140 130 120 110 100 90 80 70 60 50 40 30 20 10 ppm

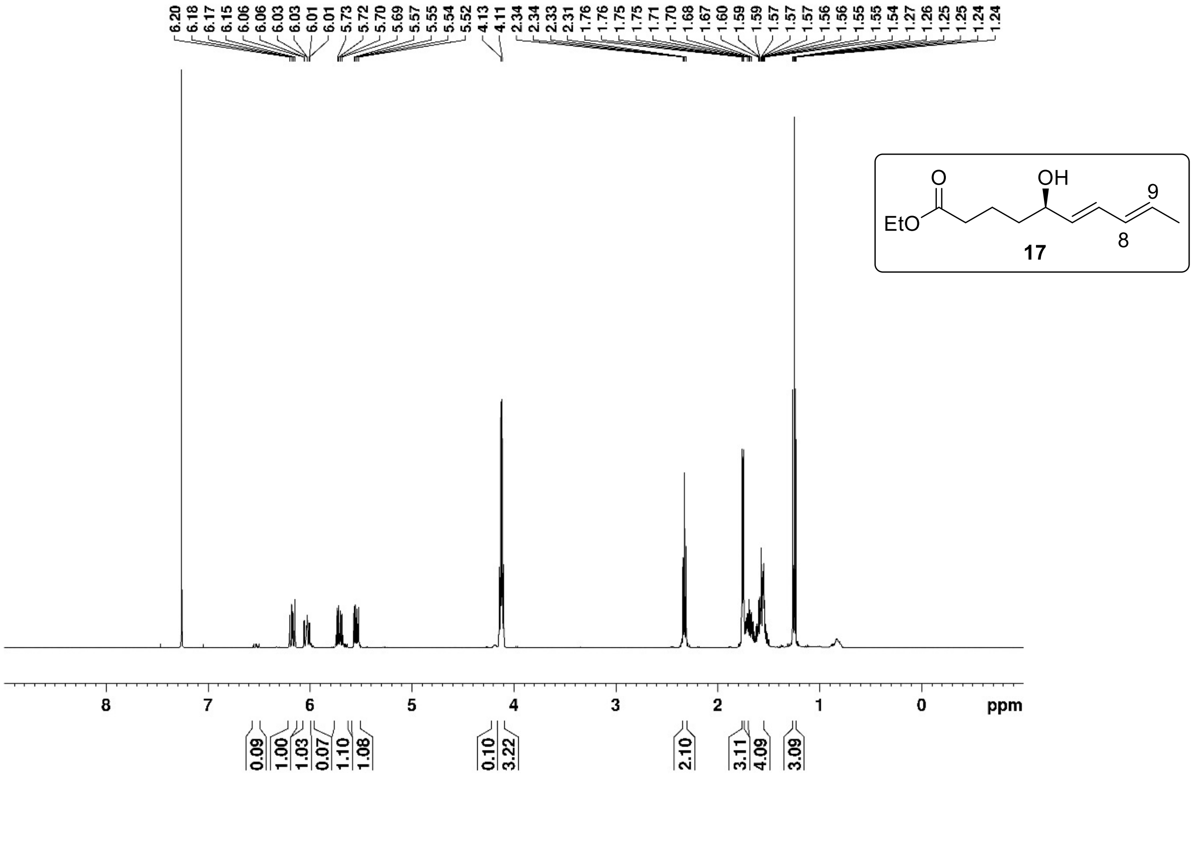
6.20 6.18 6.17 6.15 6.06 6.06 6.03 6.03 6.01 6.01 5.73 5.72 5.70 5.69 5.57 5.55 5.54 5.52 4.13 4.11 2.34 2.34 2.33 2.31 1.76 1.75 1.75 1.71 1.70 1.68 1.67 1.60 1.59 1.59 1.57 1.57 1.57 1.56 1.56 1.55 1.55 1.54 1.27 1.26 1.25 1.25 1.24 1.24
EtO
O
OH
9
8
17
8 7 6 5 4 3 2 1 0 ppm
0.09 1.00 1.03 0.07 1.10 1.08 0.10 3.22 2.10 3.11 4.09 3.09

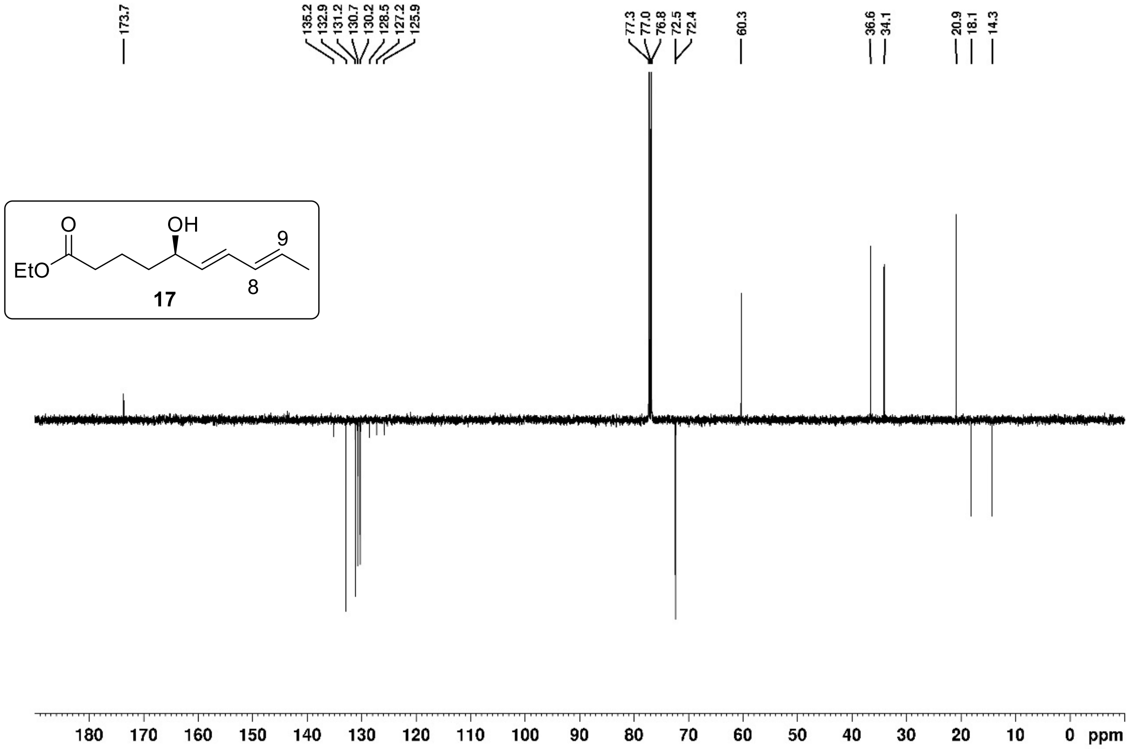
173.7
135.2
132.9
131.2
130.7
130.2
128.5
127.2
125.9
77.3
77.0
76.8
72.5
72.4
60.3
36.6
34.1
20.9
18.1
14.3
OH
EtO
9
8
17
180 170 160 150 140 130 120 110 100 90 80 70 60 50 40 30 20 10 0 ppm

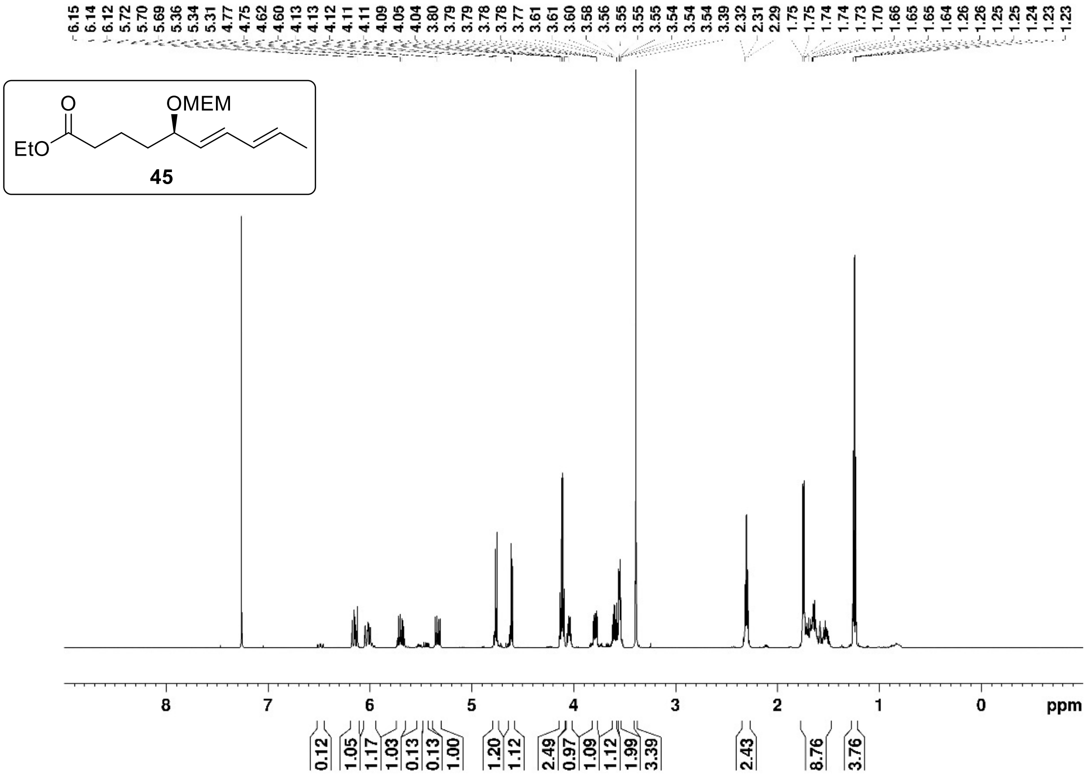
OMEM
EtO
45
8 7 6 5 4 3 2 1 0 ppm
0.12
1.05
1.17
1.03
0.13
0.13
1.00
1.20
1.12
2.49
0.97
1.09
1.12
1.99
3.39
2.43
8.76
3.76

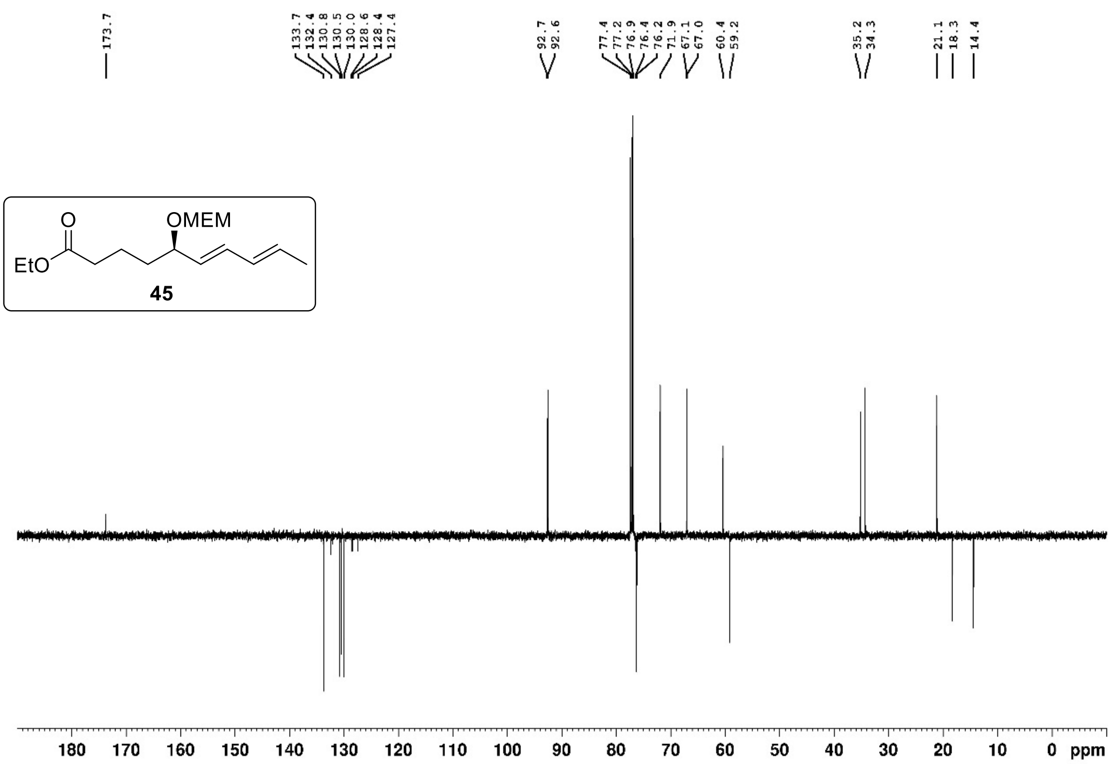
173.7
133.7 132.4 130.8 130.5 130.0 128.6 128.4 127.4
92.7 92.6
77.4 77.2 76.9 76.4 76.2 71.9 67.1 67.0
60.4 59.2
35.2 34.3
21.1 18.3 14.4
O
OMEM
EtO
45
180 170 160 150 140 130 120 110 100 90 80 70 60 50 40 30 20 10 0 ppm

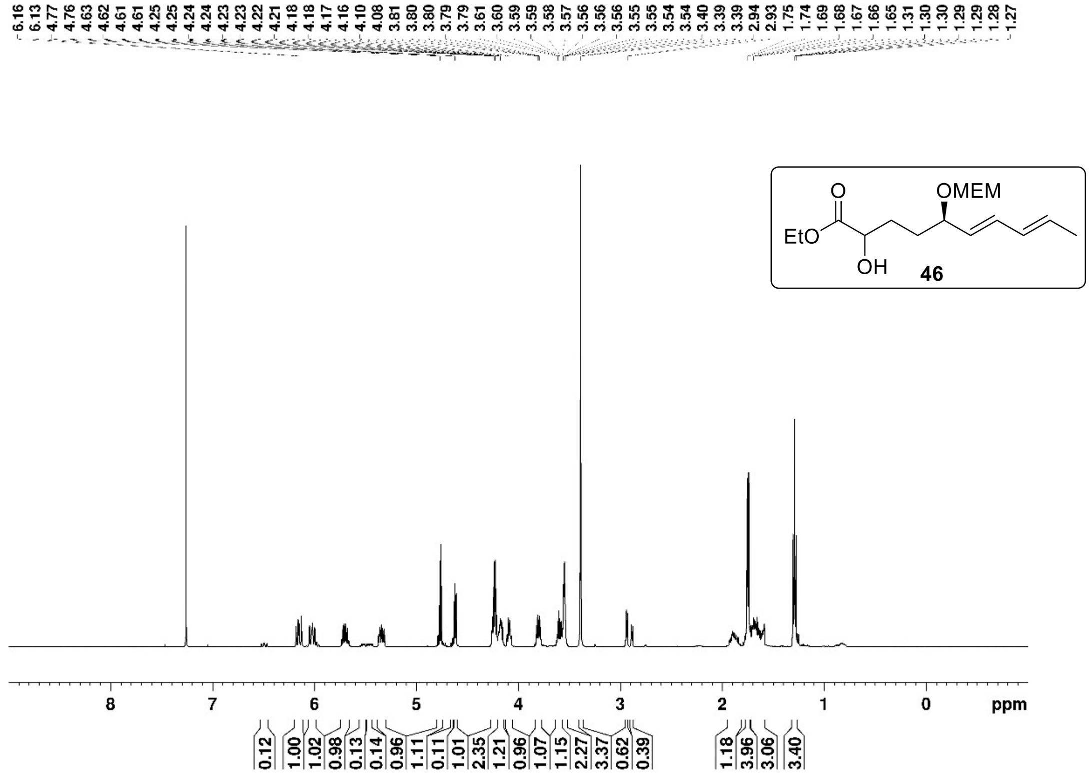
6.16 6.13 4.77 4.76 4.63 4.62 4.61 4.61 4.25 4.25 4.24 4.24 4.23 4.23 4.22 4.21 4.18 4.18 4.17 4.16 4.10 4.08 3.81 3.80 3.80 3.79 3.79 3.61 3.60 3.59 3.59 3.58 3.57 3.56 3.56 3.56 3.55 3.55 3.54 3.54 3.40 3.39 3.39 2.94 2.93 1.75 1.74 1.69 1.68 1.67 1.66 1.65 1.31 1.30 1.30 1.29 1.29 1.28 1.27
O
OMEM
EtO
OH
46
8 7 6 5 4 3 2 1 0 ppm
0.12 1.00 1.02 0.98 0.13 0.14 0.96 1.11 0.11 1.01 2.35 1.21 0.96 1.07 1.15 2.27 3.37 0.62 0.39 1.18 3.96 3.06 3.40

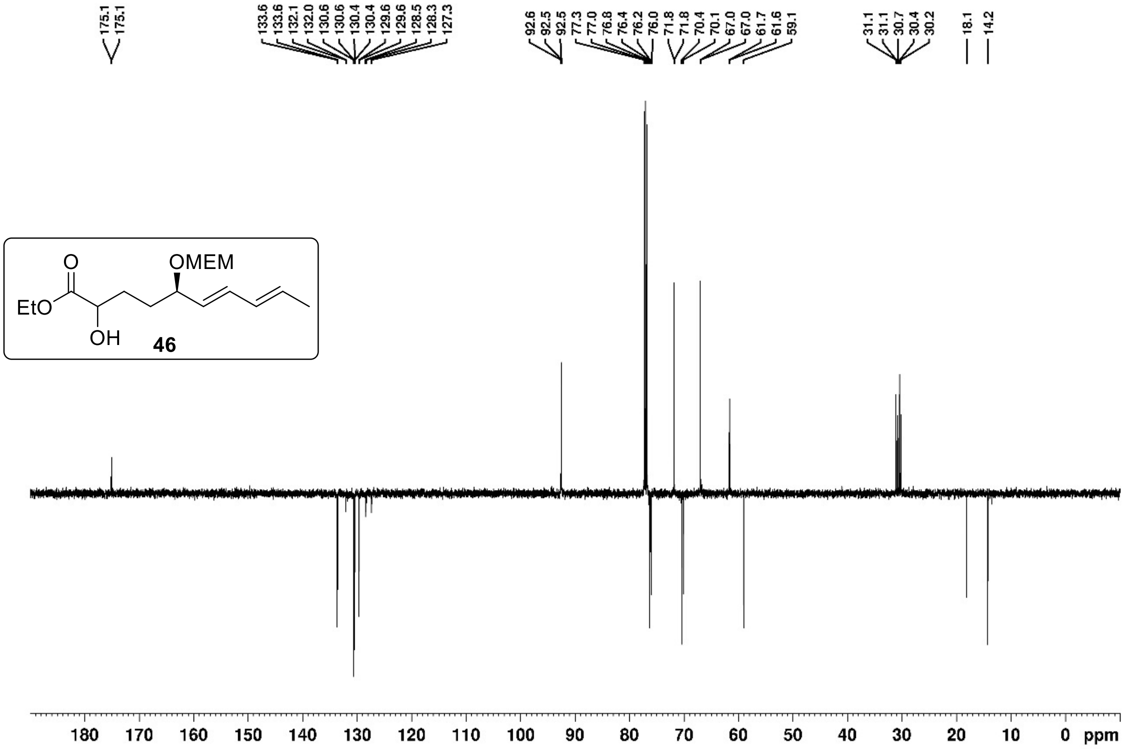
175.1
175.1
133.6
133.6
132.1
132.0
130.6
130.6
130.4
130.4
129.6
129.6
128.5
128.3
127.3
92.6
92.5
92.5
77.3
77.0
76.8
76.4
76.2
76.0
71.8
71.8
70.4
70.1
67.0
67.0
61.7
61.6
59.1
31.1
31.1
30.7
30.4
30.2
18.1
14.2
O
OMEM
EtO
OH
46
180 170 160 150 140 130 120 110 100 90 80 70 60 50 40 30 20 10 0 ppm

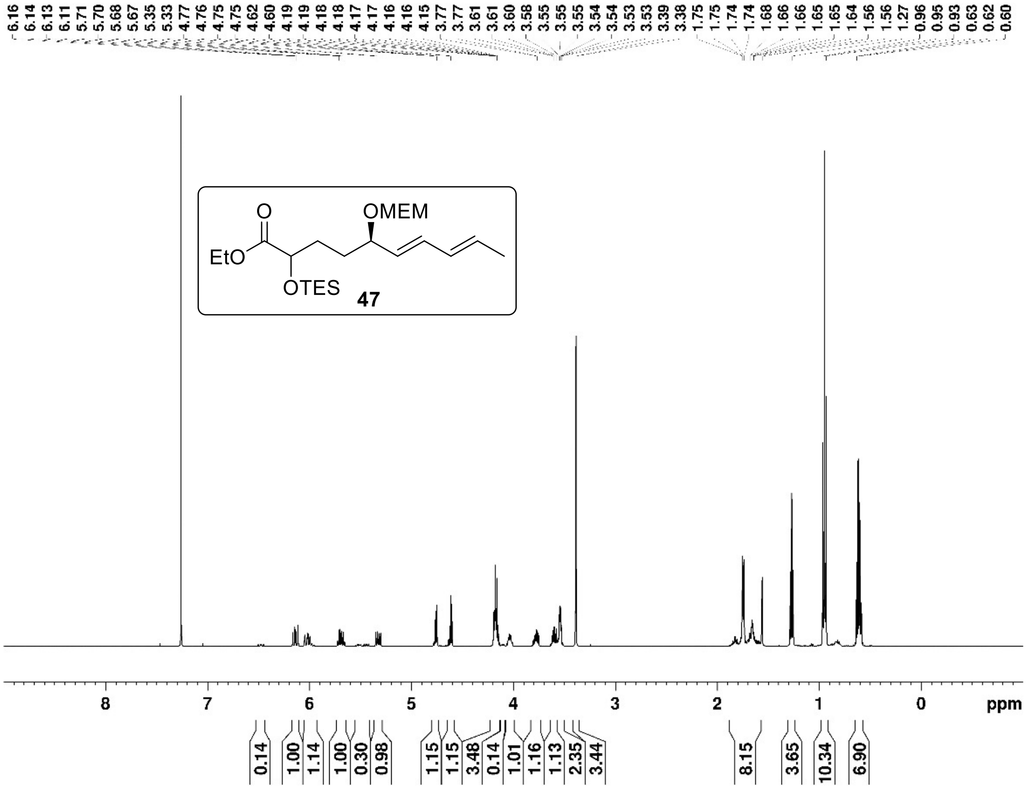
6.16
6.14
6.13
6.11
5.71
5.70
5.68
5.67
5.35
5.33
4.77
4.76
4.75
4.75
4.62
4.60
4.19
4.19
4.18
4.18
4.17
4.17
4.16
4.16
4.15
3.77
3.77
3.61
3.61
3.60
3.58
3.55
3.55
3.54
3.54
3.53
3.53
3.39
3.38
1.75
1.75
1.74
1.74
1.68
1.66
1.66
1.65
1.65
1.64
1.56
1.56
1.27
0.96
0.95
0.93
0.63
0.62
0.60
O
OMEM
EtO
OTES
47
8 7 6 5 4 3 2 1 0 ppm
0.14
1.00
1.14
1.00
0.30
0.98
1.15
1.15
3.48
0.14
1.01
1.16
1.13
2.35
3.44
8.15
3.65
10.34
6.90

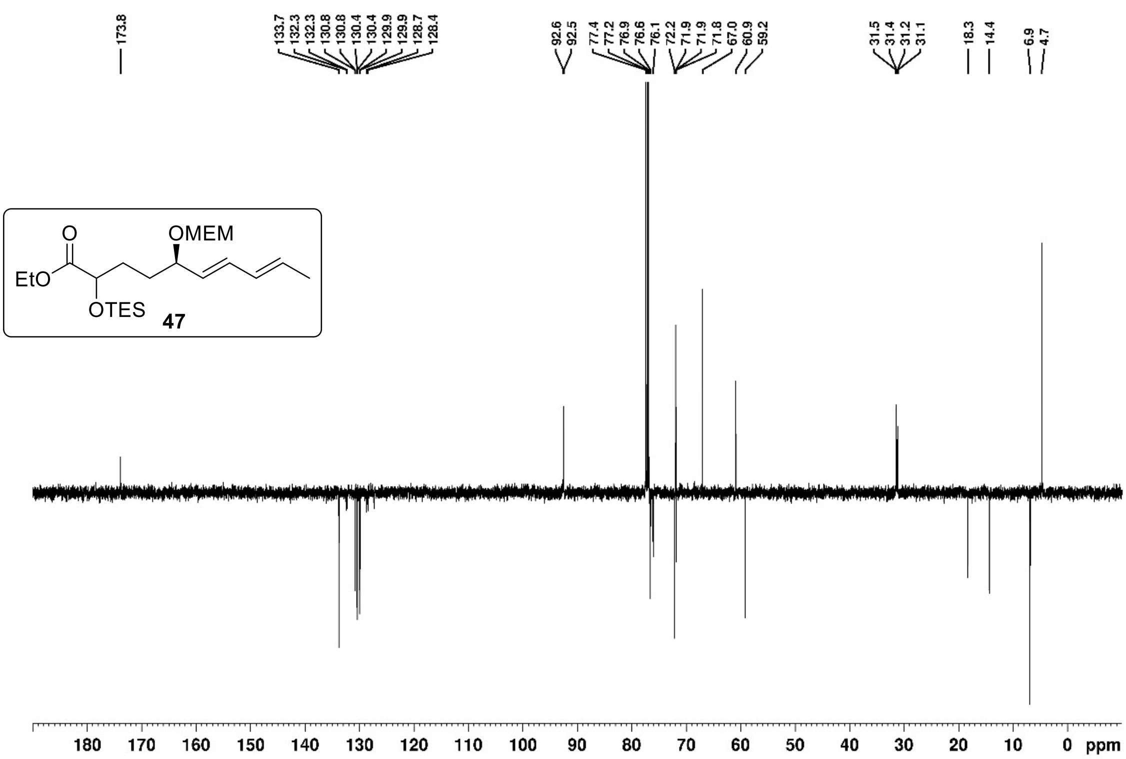
O
OMEM
EtO
OTES
47
ppm

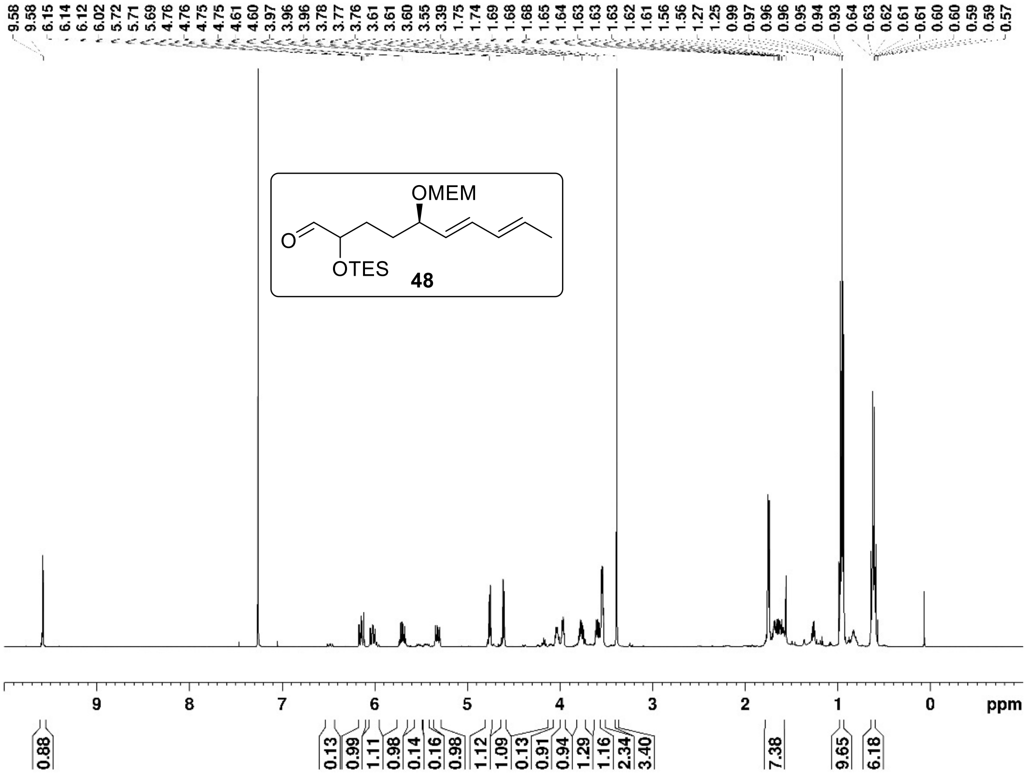
OMEM
O
OTES
48
ppm

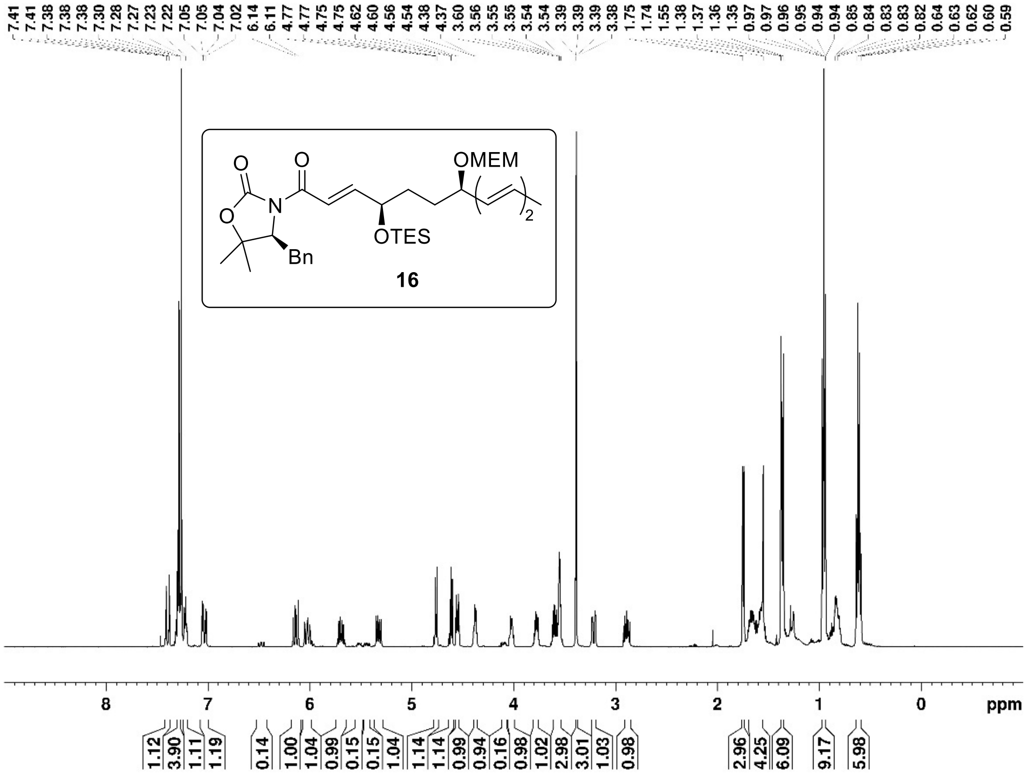

OMEM
OTES
Bn
16
ppm

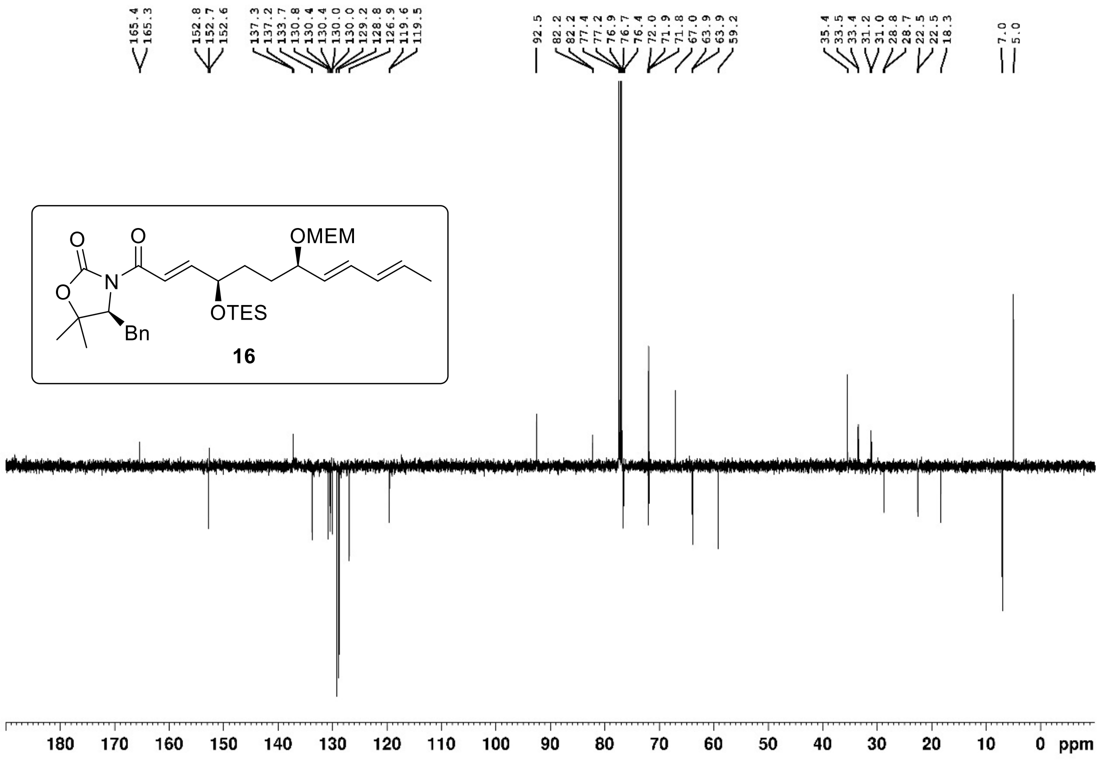

OMEM
OTES
Bn
16
ppm

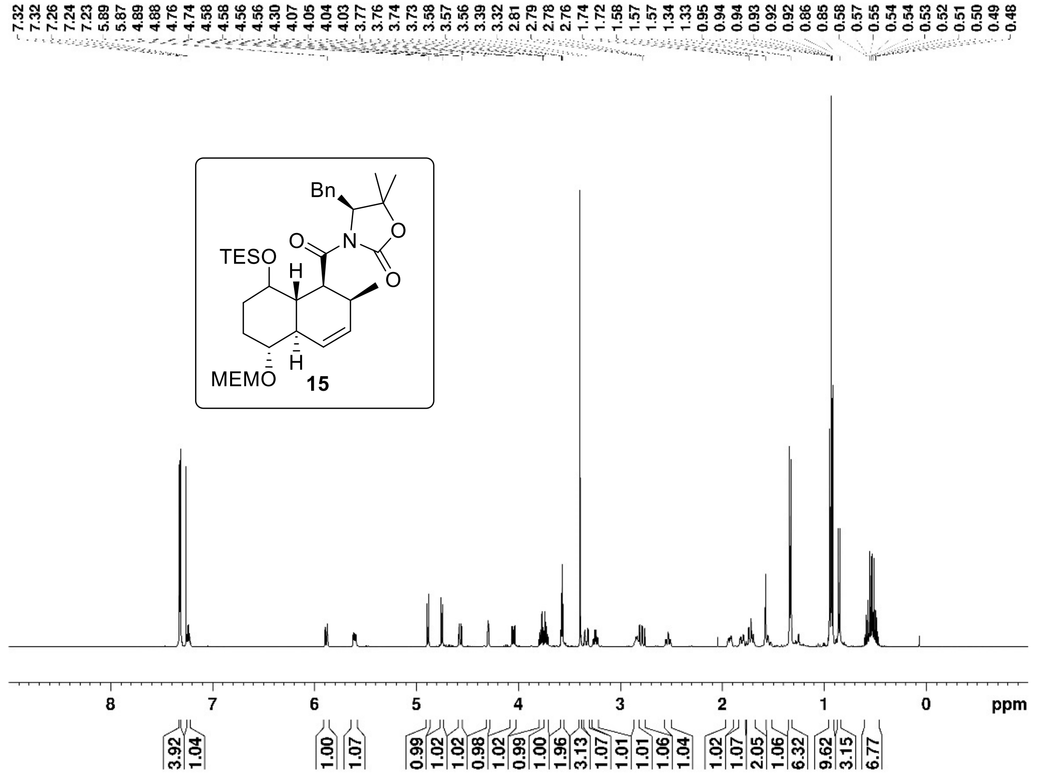

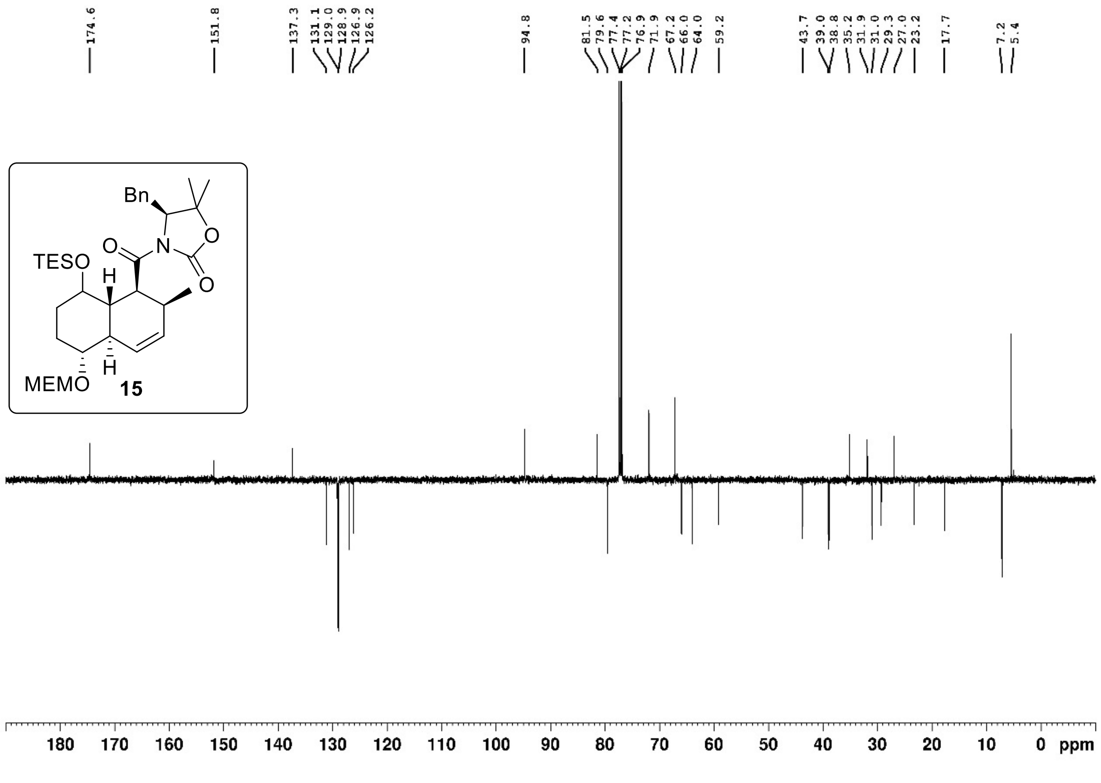

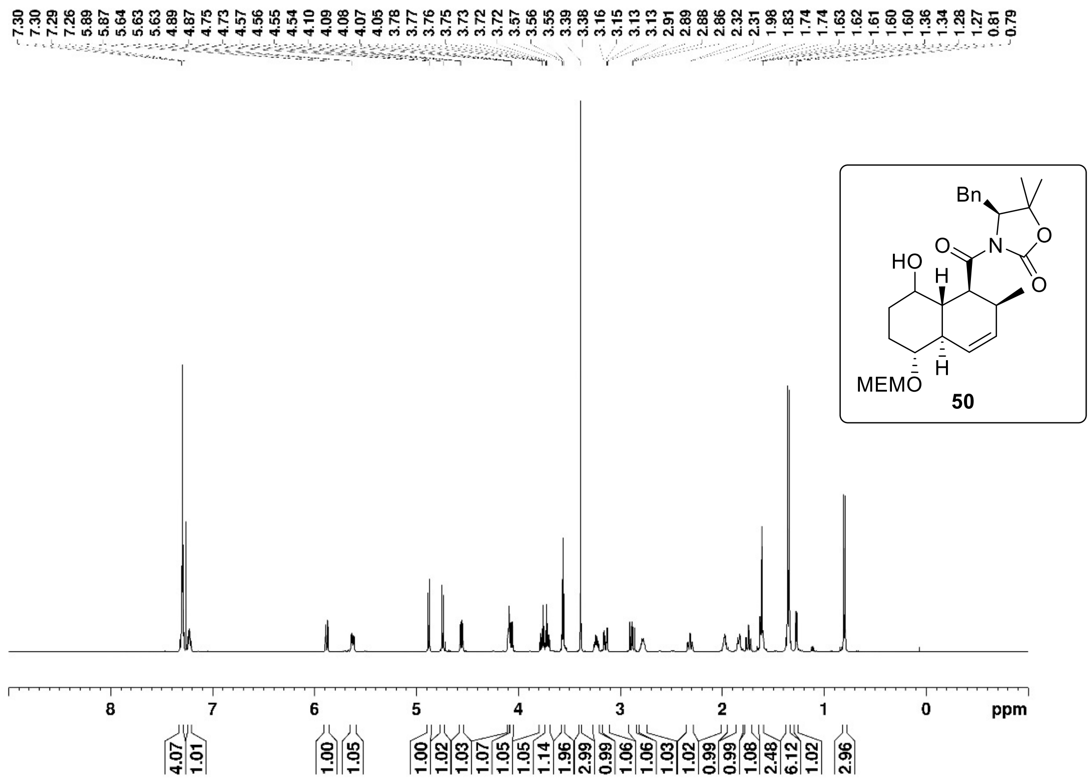
7.30
7.30
7.29
7.26
5.89
5.87
5.64
5.63
5.63
4.89
4.87
4.75
4.73
4.57
4.56
4.55
4.54
4.10
4.09
4.08
4.07
4.05
3.78
3.77
3.76
3.75
3.73
3.72
3.72
3.57
3.56
3.55
3.39
3.38
3.16
3.15
3.13
3.13
2.91
2.89
2.88
2.86
2.32
2.31
1.98
1.83
1.74
1.74
1.63
1.62
1.61
1.60
1.60
1.36
1.34
1.28
1.27
0.81
0.79
Bn
O
N
O
HO
H
O
H
MEMO
50
8
7
6
5
4
3
2
1
0
ppm
4.07
1.01
1.00
1.05
1.00
1.02
1.03
1.07
1.05
1.05
1.14
1.96
2.99
0.99
1.06
1.06
1.03
1.02
0.99
0.99
1.08
2.48
6.12
1.02
2.96

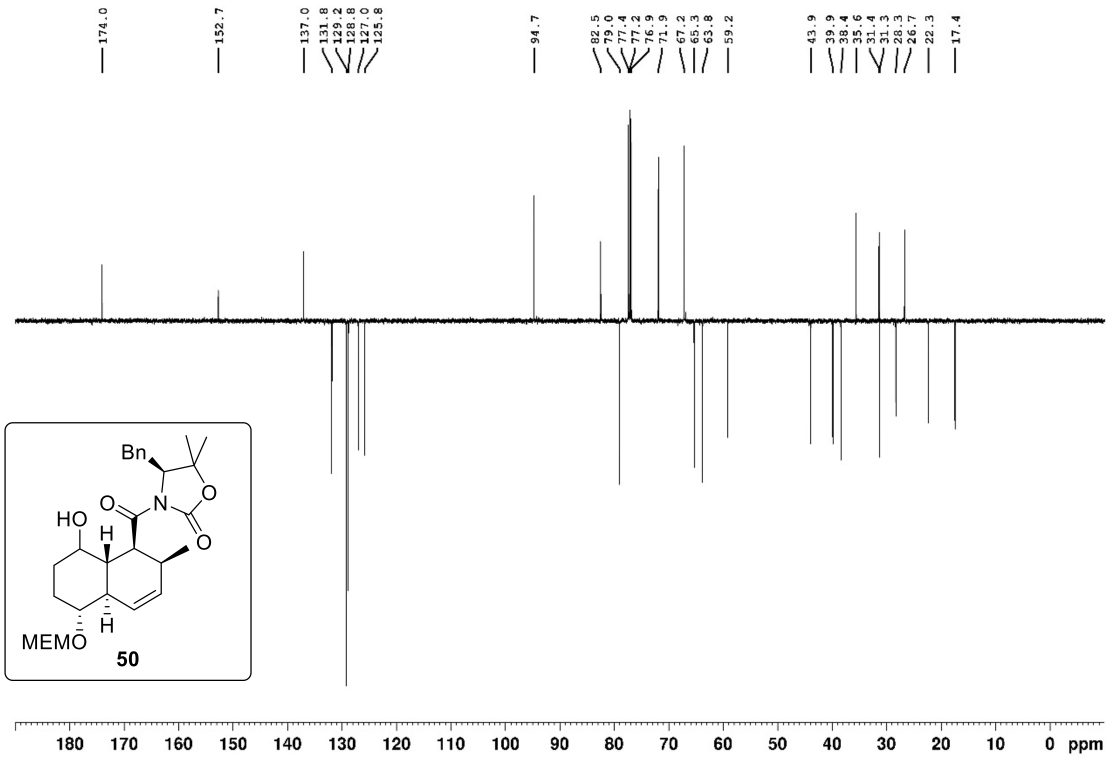
174.0
152.7
137.0
131.8
129.2
128.8
127.0
125.8
94.7
82.5
79.0
77.4
77.2
76.9
71.9
67.2
65.3
63.8
59.2
43.9
39.9
38.4
35.6
31.4
31.3
28.3
26.7
22.3
17.4
Bn
O
N
O
HO
H
O
H
MEMO
50
180
170
160
150
140
130
120
110
100
90
80
70
60
50
40
30
20
10
0
ppm

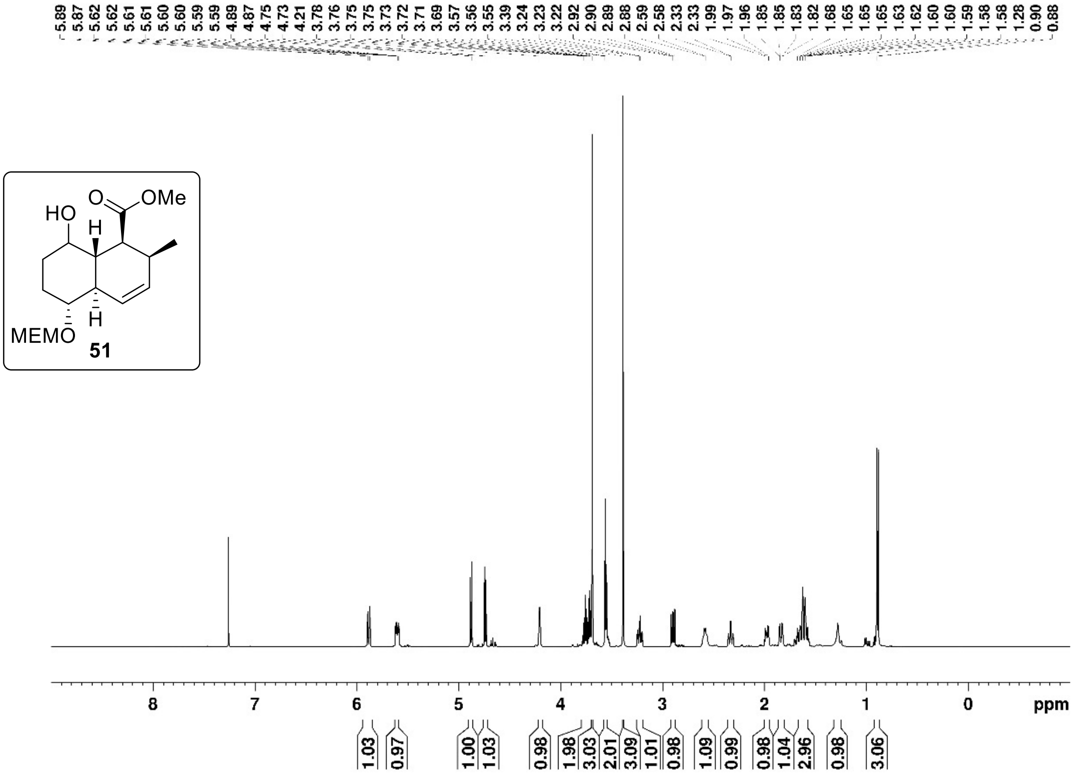
5.89 5.87 5.62 5.62 5.61 5.61 5.60 5.60 5.59 5.59 4.89 4.87 4.75 4.73 4.21 3.78 3.76 3.75 3.75 3.73 3.72 3.71 3.69 3.57 3.56 3.55 3.39 3.24 3.23 3.22 2.92 2.90 2.89 2.88 2.59 2.58 2.33 2.33 1.99 1.97 1.96 1.85 1.85 1.83 1.82 1.68 1.65 1.65 1.65 1.63 1.62 1.60 1.60 1.59 1.58 1.58 1.28 0.90 0.88
HO
O
OMe
H
H
MEMO
51
8 7 6 5 4 3 2 1 0 ppm
1.03 0.97 1.00 1.03 0.98 1.98 3.03 2.01 3.09 1.01 0.98 1.09 0.99 0.98 1.04 2.96 0.98 3.06

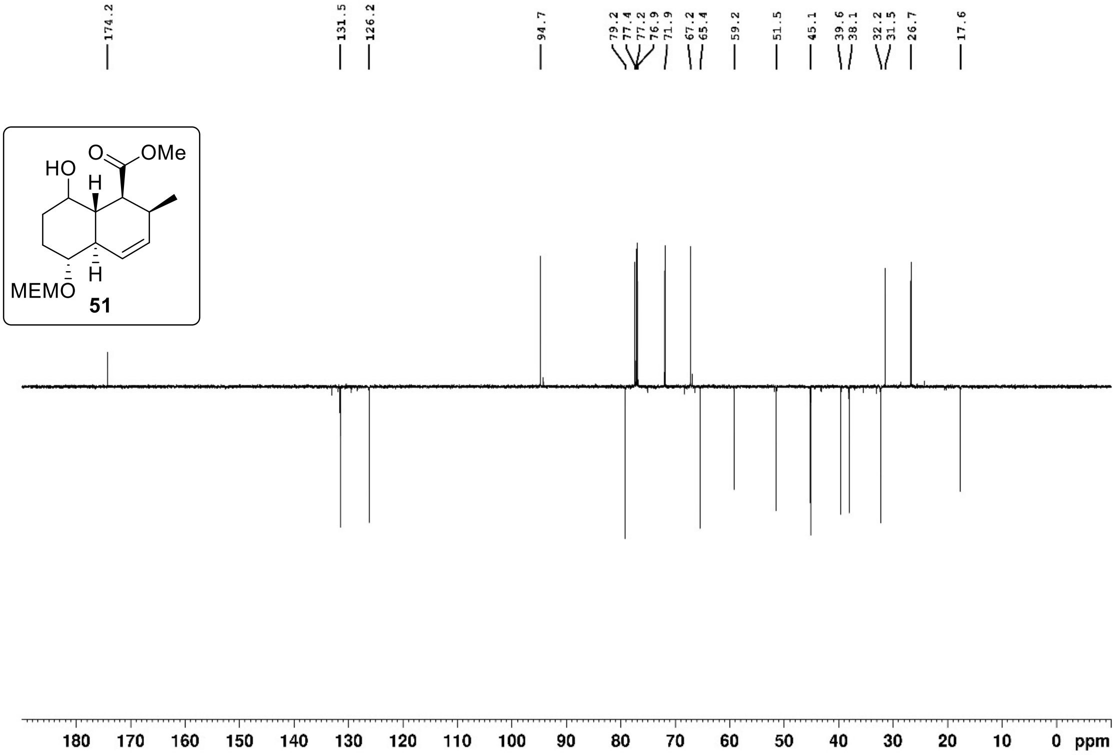
174.2 131.5 126.2 94.7 79.2 77.4 77.2 76.9 71.9 67.2 65.4 59.2 51.5 45.1 39.6 38.1 32.2 31.5 26.7 17.6
HO
O
OMe
H
H
MEMO
51
180 170 160 150 140 130 120 110 100 90 80 70 60 50 40 30 20 10 0 ppm

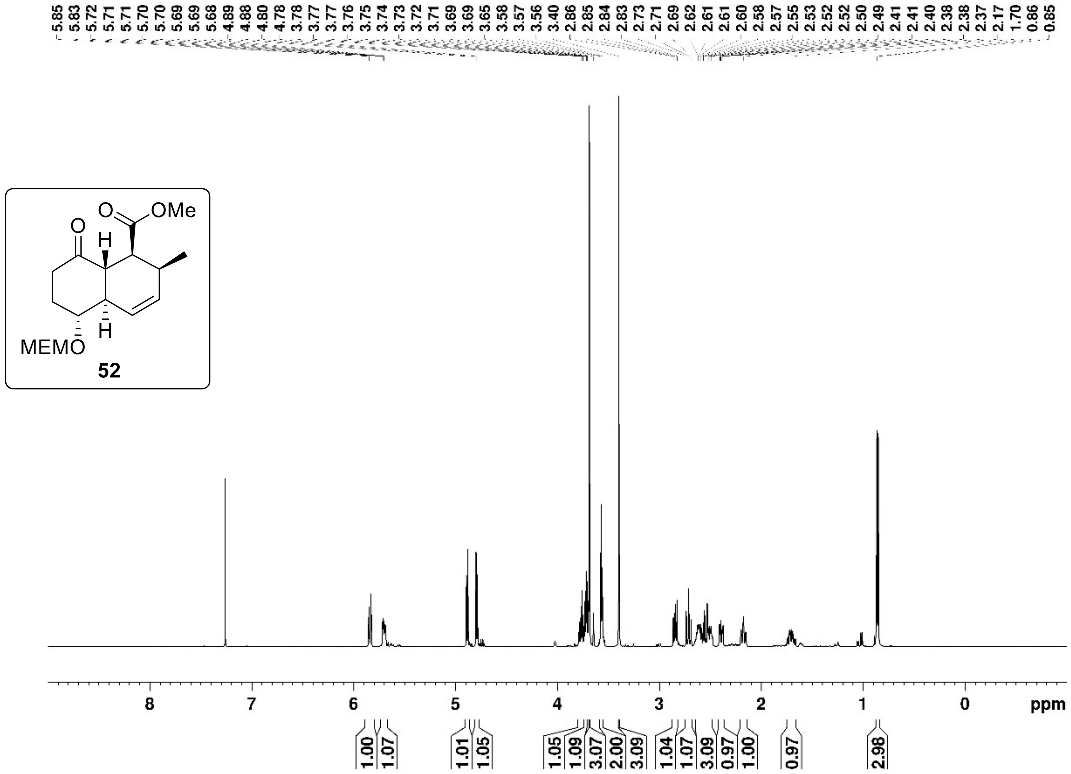
5.85 5.83 5.72 5.71 5.71 5.70 5.70 5.69 5.69 5.68 4.89 4.88 4.80 4.78 3.78 3.78 3.77 3.77 3.76 3.75 3.74 3.73 3.72 3.71 3.69 3.69 3.65 3.58 3.57 3.56 3.40 2.86 2.85 2.84 2.83 2.73 2.71 2.69 2.62 2.61 2.61 2.60 2.58 2.57 2.55 2.53 2.52 2.52 2.50 2.49 2.41 2.41 2.40 2.38 2.38 2.37 2.17 1.70 0.86 0.85
OMe
O
O
H
H
MEMO
52
8 7 6 5 4 3 2 1 0 ppm
1.00 1.07 1.01 1.05 1.05 1.09 3.07 2.00 3.09 1.04 1.07 3.09 0.97 1.00 0.97 2.98

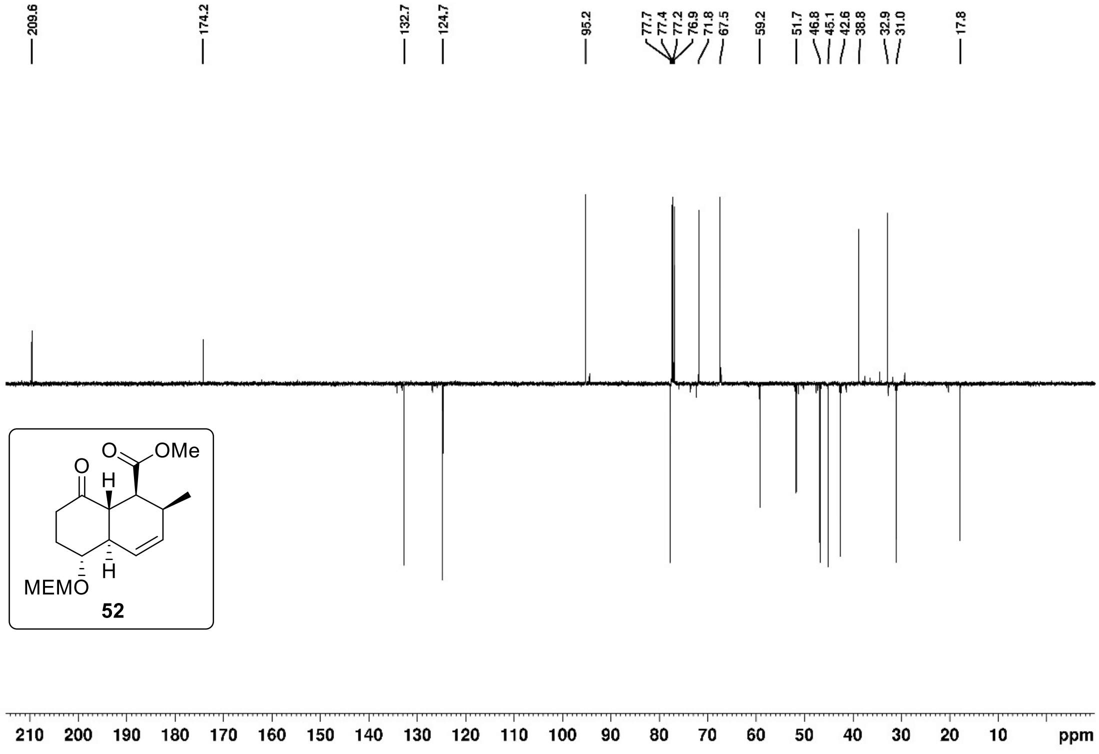
209.6 174.2 132.7 124.7 95.2 77.7 77.4 77.2 76.9 71.8 67.5 59.2 51.7 46.8 45.1 42.6 38.8 32.9 31.0 17.8
OMe
O
O
H
H
MEMO
52
210 200 190 180 170 160 150 140 130 120 110 100 90 80 70 60 50 40 30 20 10 ppm

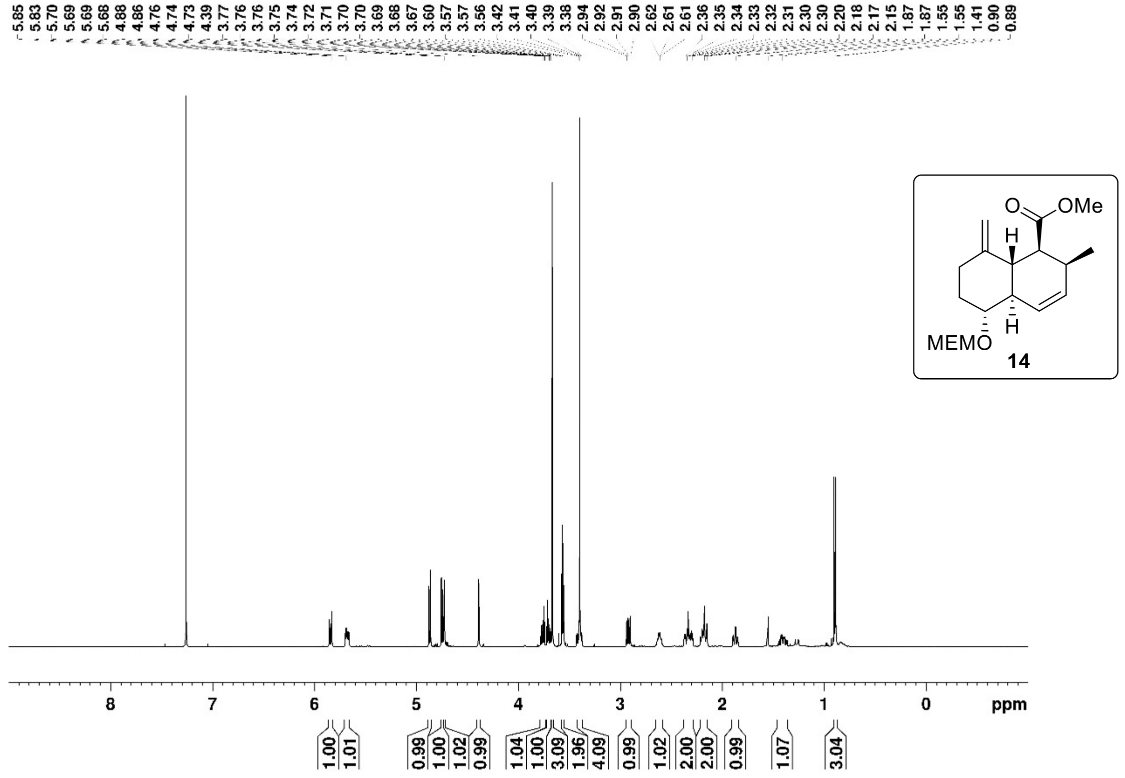

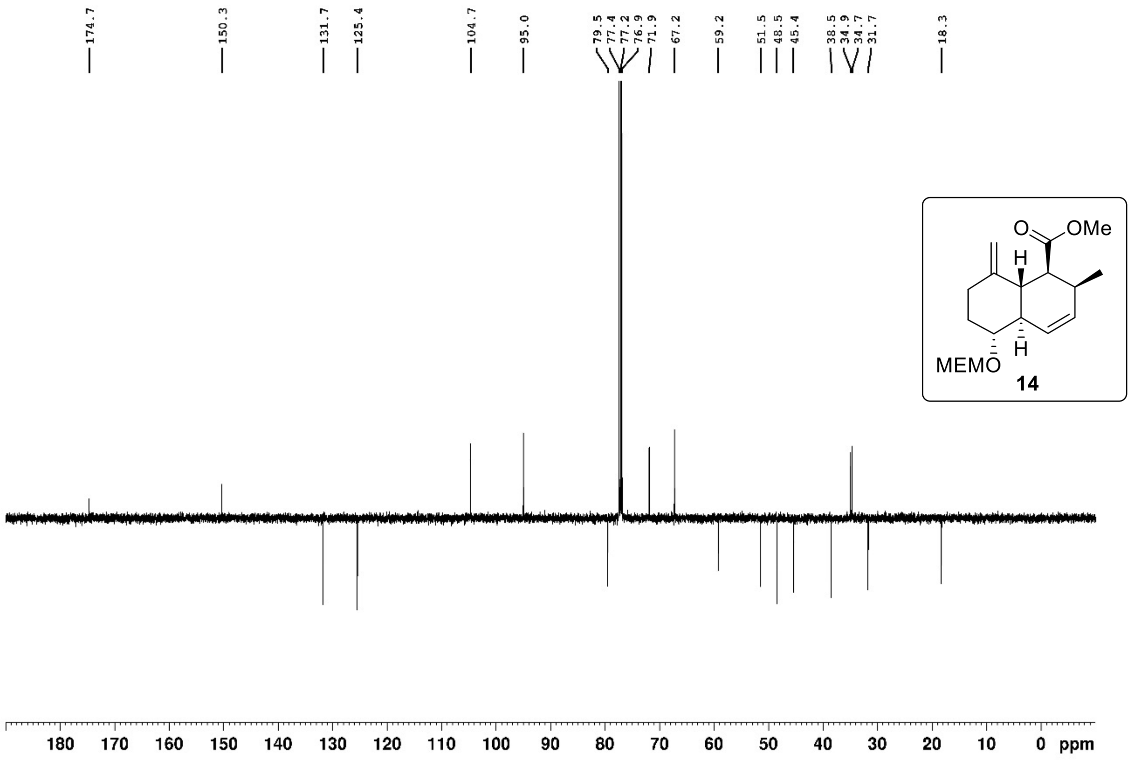

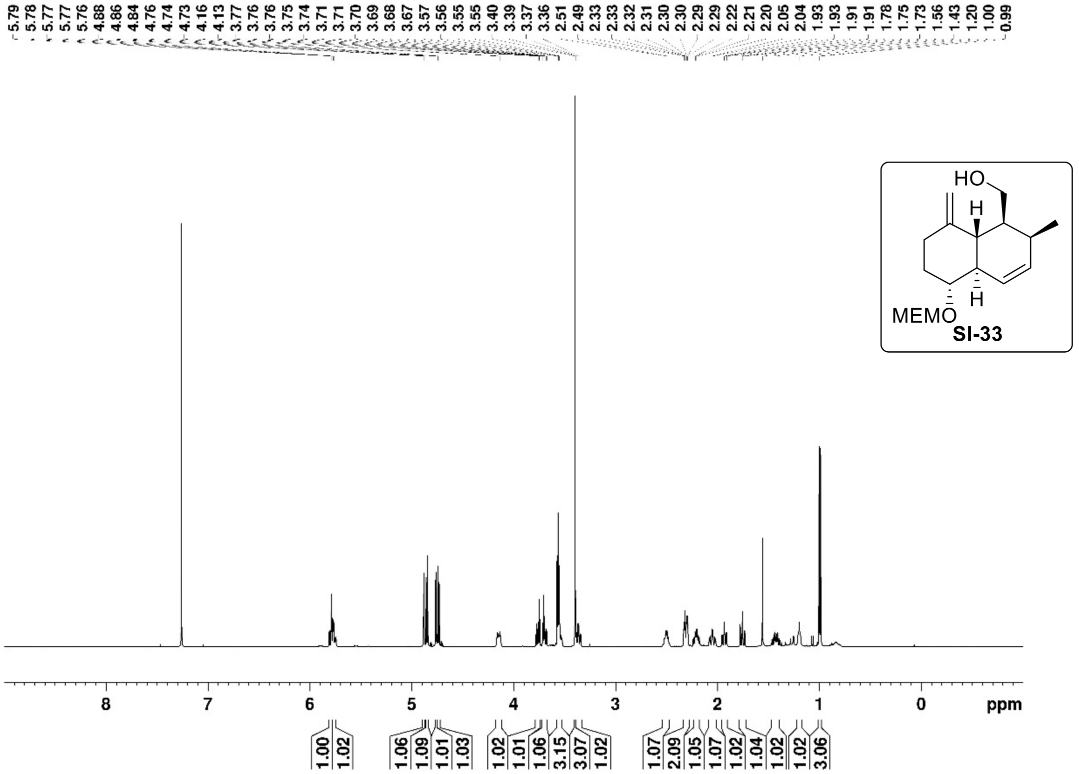
HO
H
H
MEMO
SI-33
8 7 6 5 4 3 2 1 0 ppm
1.00 1.02 1.06 1.09 1.01 1.03 1.02 1.01 1.06 3.15 3.07 1.02 1.07 2.09 1.05 1.07 1.02 1.04 1.02 1.02 3.06

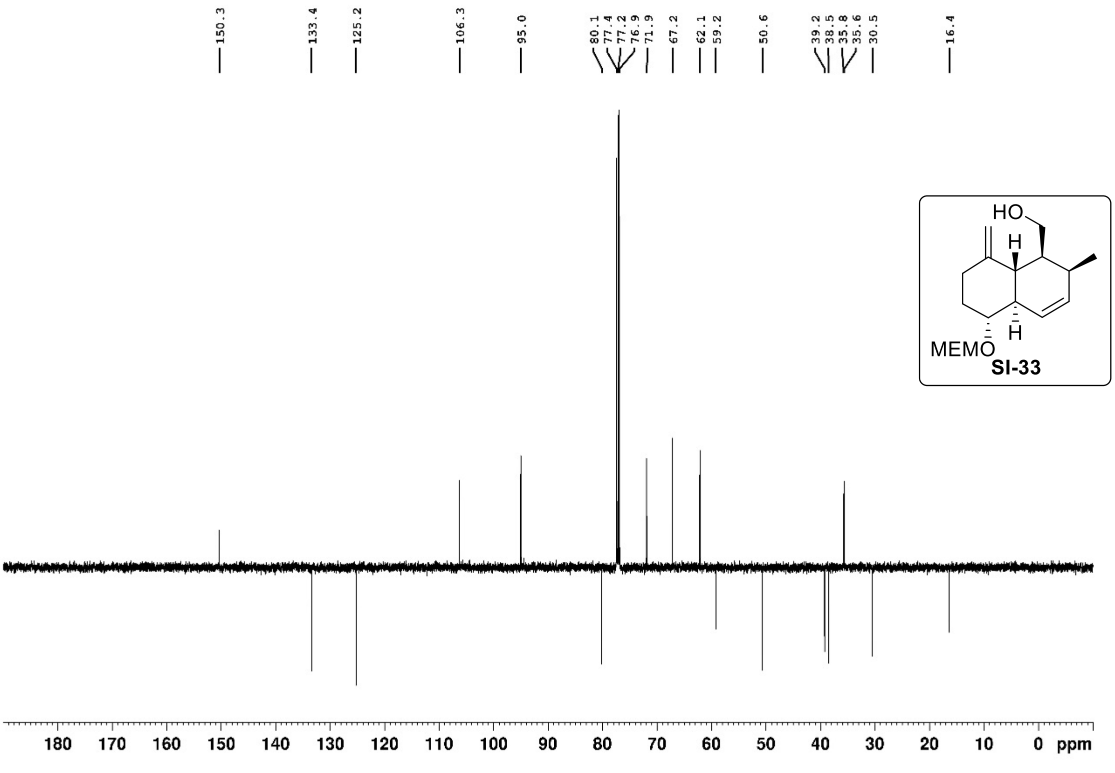
150.3 133.4 125.2 106.3 95.0 80.1 77.4 77.2 76.9 71.9 67.2 62.1 59.2 50.6 39.2 38.5 35.8 35.6 30.5 16.4
HO
H
H
MEMO
SI-33
180 170 160 150 140 130 120 110 100 90 80 70 60 50 40 30 20 10 0 ppm

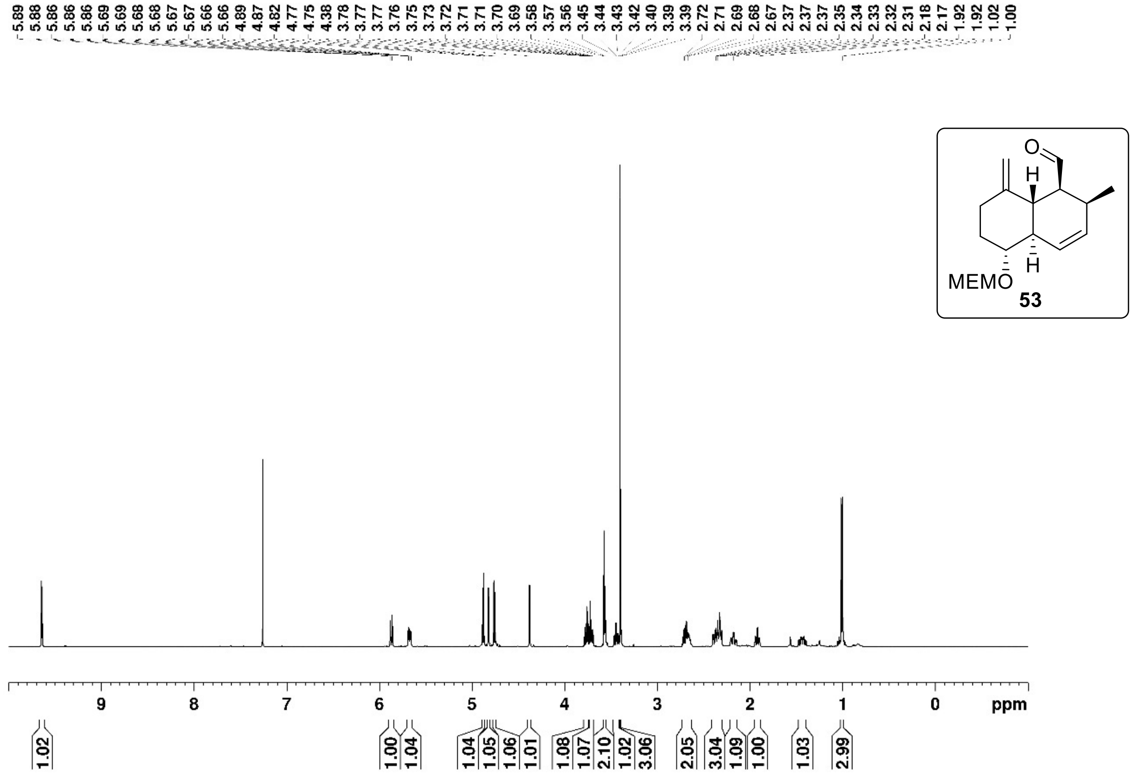

5.89
5.88
5.86
5.86
5.86
5.69
5.69
5.68
5.68
5.67
5.66
5.66
4.89
4.87
4.82
4.77
4.75
4.38
3.78
3.77
3.77
3.76
3.75
3.73
3.72
3.71
3.71
3.70
3.69
3.58
3.57
3.56
3.45
3.44
3.43
3.42
3.40
3.39
3.39
2.72
2.71
2.69
2.68
2.67
2.37
2.37
2.37
2.35
2.34
2.33
2.32
2.31
2.18
2.17
1.92
1.92
1.02
1.00
H
H
MEMO
53
9
8
7
6
5
4
3
2
1
0
ppm
1.02
1.00
1.04
1.04
1.05
1.06
1.01
1.08
1.07
2.10
1.02
3.06
2.05
3.04
1.09
1.00
1.03
2.99

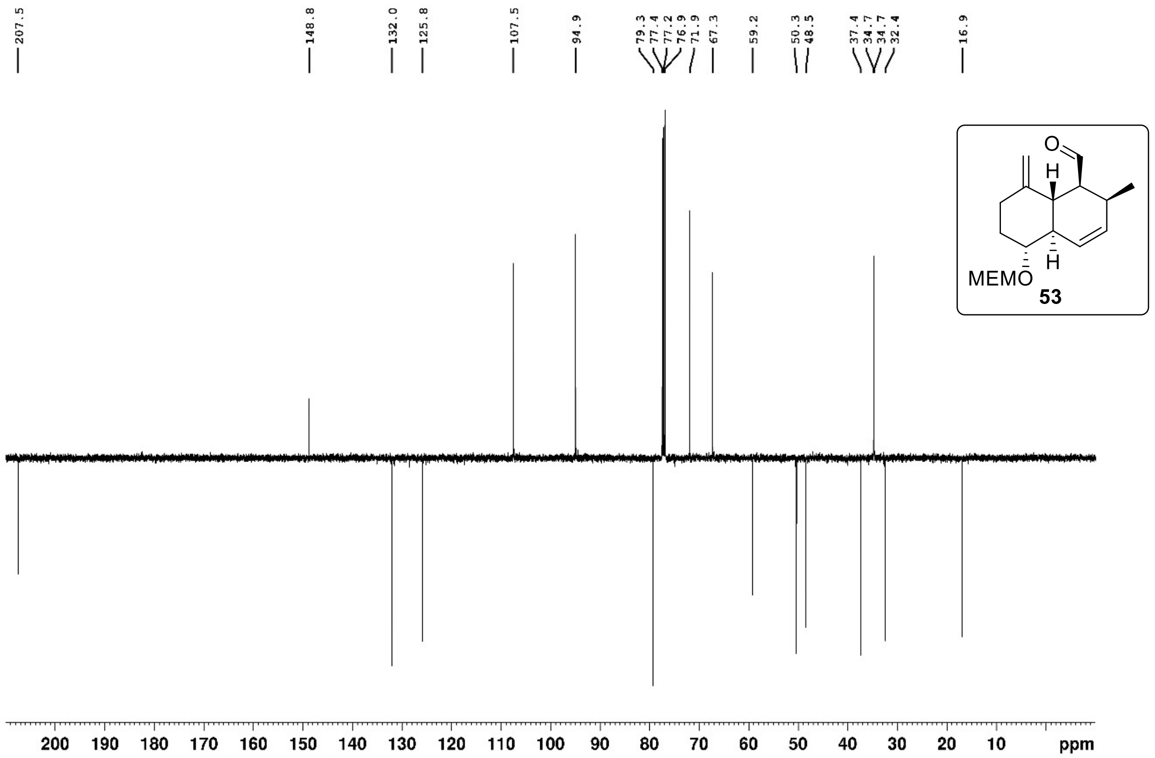

207.5
148.8
132.0
125.8
107.5
94.9
79.3
77.4
77.2
76.9
71.9
67.3
59.2
50.3
48.5
37.4
34.7
34.7
32.4
16.9
H
H
MEMO
53
200
190
180
170
160
150
140
130
120
110
100
90
80
70
60
50
40
30
20
10
ppm

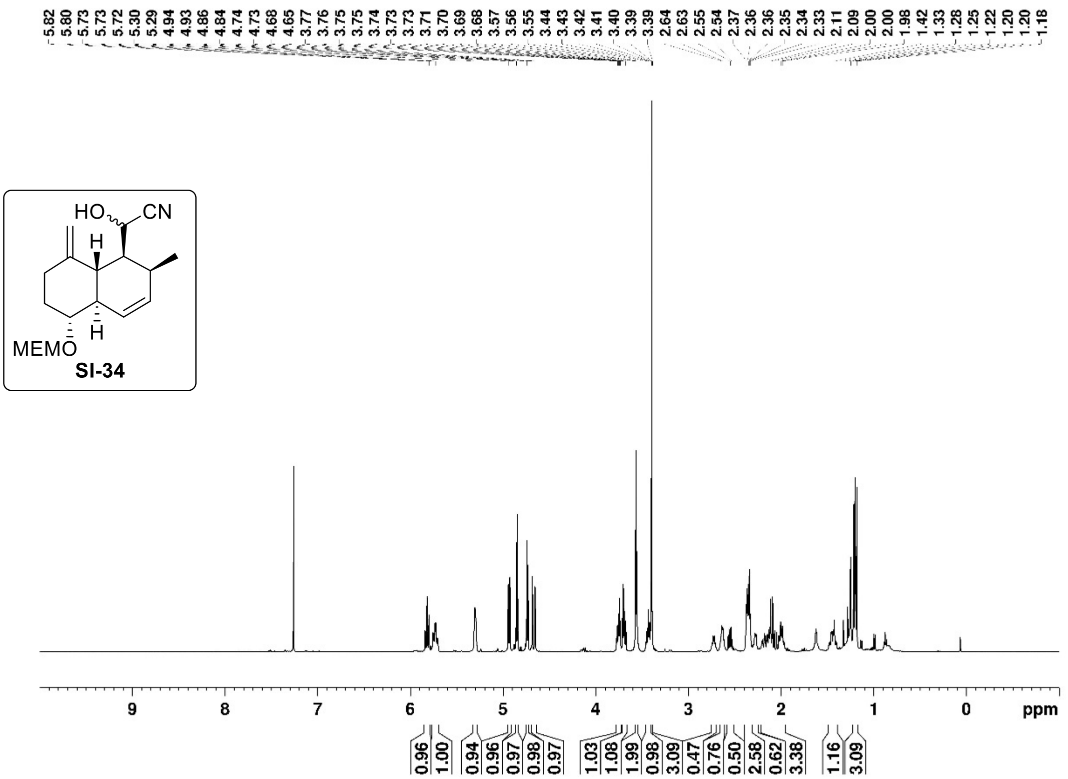

5.82 5.80 5.73 5.73 5.72 5.30 5.29 4.94 4.93 4.86 4.84 4.74 4.73 4.68 4.65 3.77 3.76 3.75 3.75 3.74 3.73 3.73 3.71 3.70 3.69 3.68 3.57 3.56 3.55 3.44 3.43 3.42 3.41 3.40 3.39 3.39 2.64 2.63 2.55 2.54 2.37 2.36 2.36 2.35 2.34 2.33 2.11 2.09 2.00 2.00 1.98 1.42 1.33 1.28 1.25 1.22 1.20 1.20 1.18
HO
CN
H
H
MEMO
SI-34
9 8 7 6 5 4 3 2 1 0 ppm
0.96 1.00 0.94 0.96 0.97 0.98 0.97 1.03 1.08 1.99 0.98 3.09 0.47 0.76 0.50 2.58 0.62 3.38 1.16 3.09

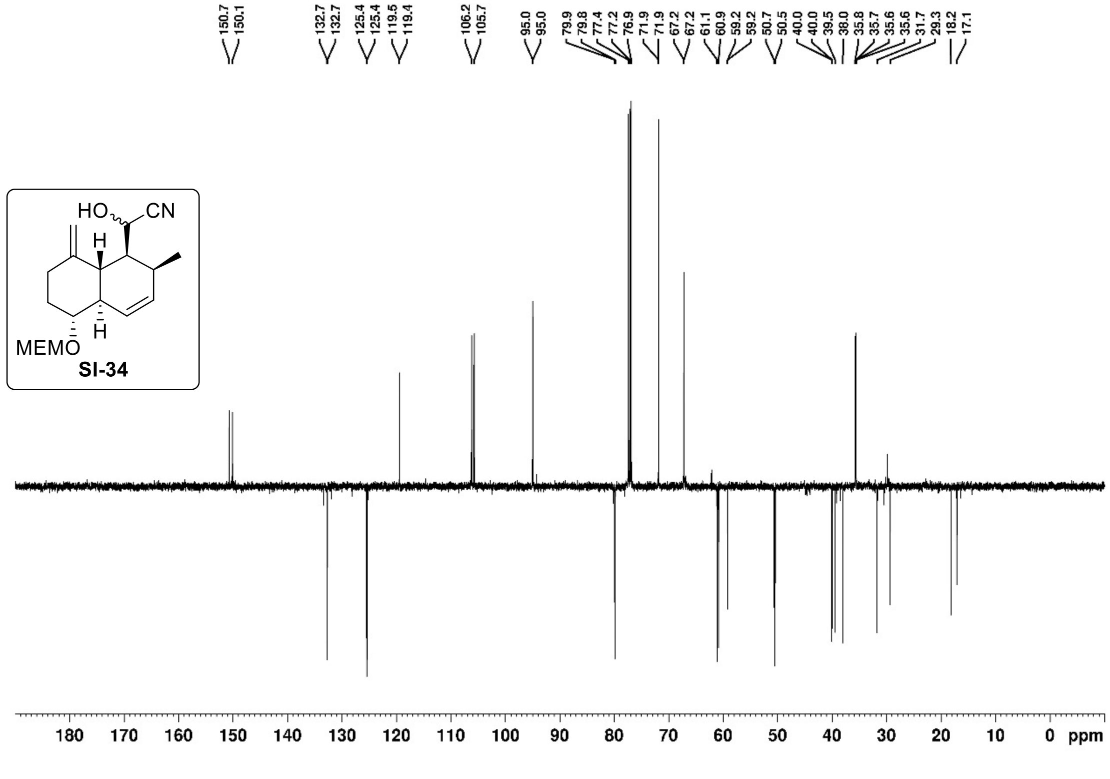

150.7 150.1 132.7 132.7 125.4 125.4 119.5 119.4 106.2 105.7 95.0 95.0 79.9 79.8 77.4 77.2 76.9 71.9 71.9 67.2 67.2 61.1 60.9 59.2 59.2 50.7 50.5 40.0 40.0 39.5 38.0 35.8 35.7 35.6 35.6 31.7 29.3 18.2 17.1
HO
CN
H
H
MEMO
SI-34
180 170 160 150 140 130 120 110 100 90 80 70 60 50 40 30 20 10 0 ppm

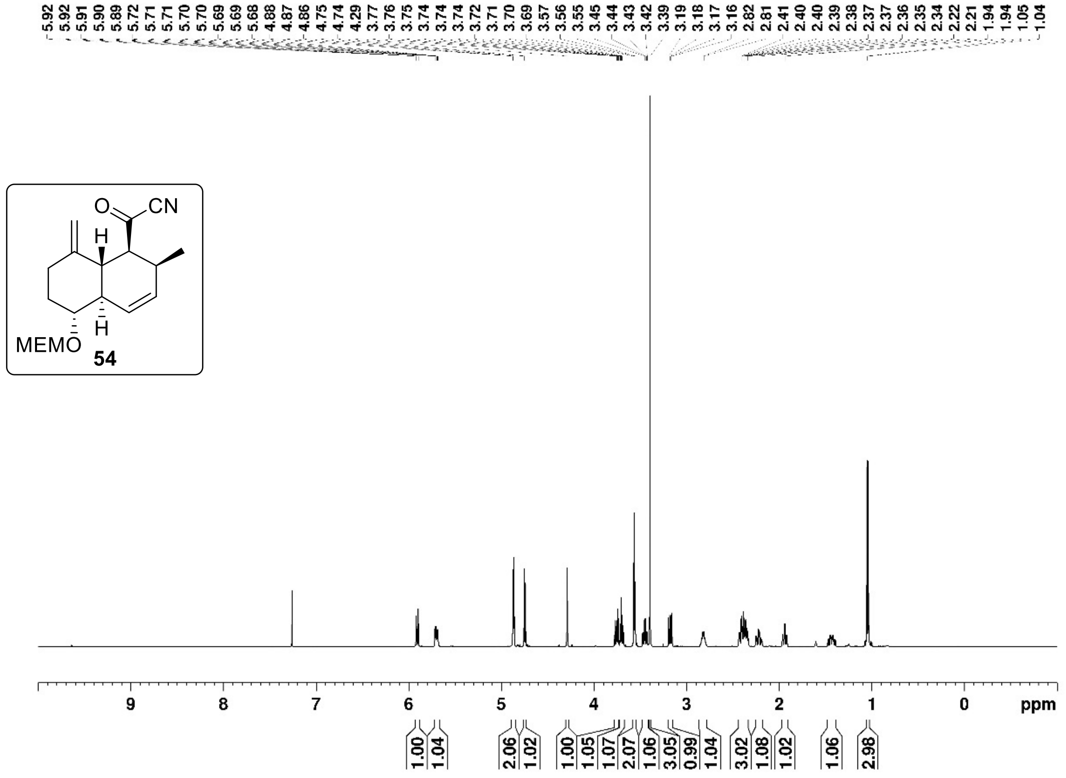
5.92 5.92 5.91 5.90 5.89 5.72 5.71 5.71 5.70 5.69 5.69 5.68 4.88 4.87 4.86 4.75 4.74 4.29 3.77 3.76 3.75 3.74 3.74 3.74 3.72 3.71 3.70 3.69 3.57 3.56 3.55 3.45 3.44 3.43 3.42 3.39 3.19 3.18 3.17 3.16 2.82 2.81 2.41 2.40 2.40 2.39 2.38 2.37 2.37 2.36 2.35 2.34 2.22 2.21 1.94 1.94 1.05 1.04
O
CN
H
H
MEMO
54
9 8 7 6 5 4 3 2 1 0 ppm
1.00 1.04 2.06 1.02 1.00 1.05 1.07 2.07 1.06 3.05 0.99 1.04 3.02 1.08 1.02 1.06 2.98

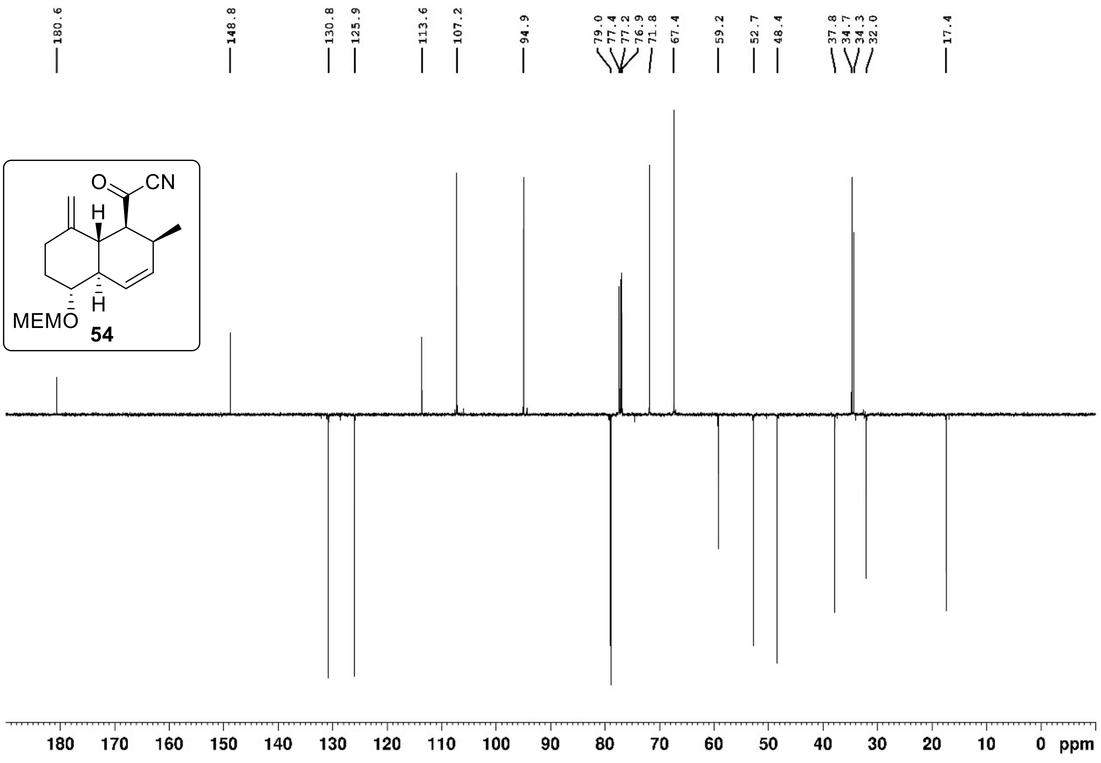
180.6 148.8 130.8 125.9 113.6 107.2 94.9 79.0 77.4 77.2 76.9 71.8 67.4 59.2 52.7 48.4 37.8 34.7 34.3 32.0 17.4
O
CN
H
H
MEMO
54
180 170 160 150 140 130 120 110 100 90 80 70 60 50 40 30 20 10 0 ppm

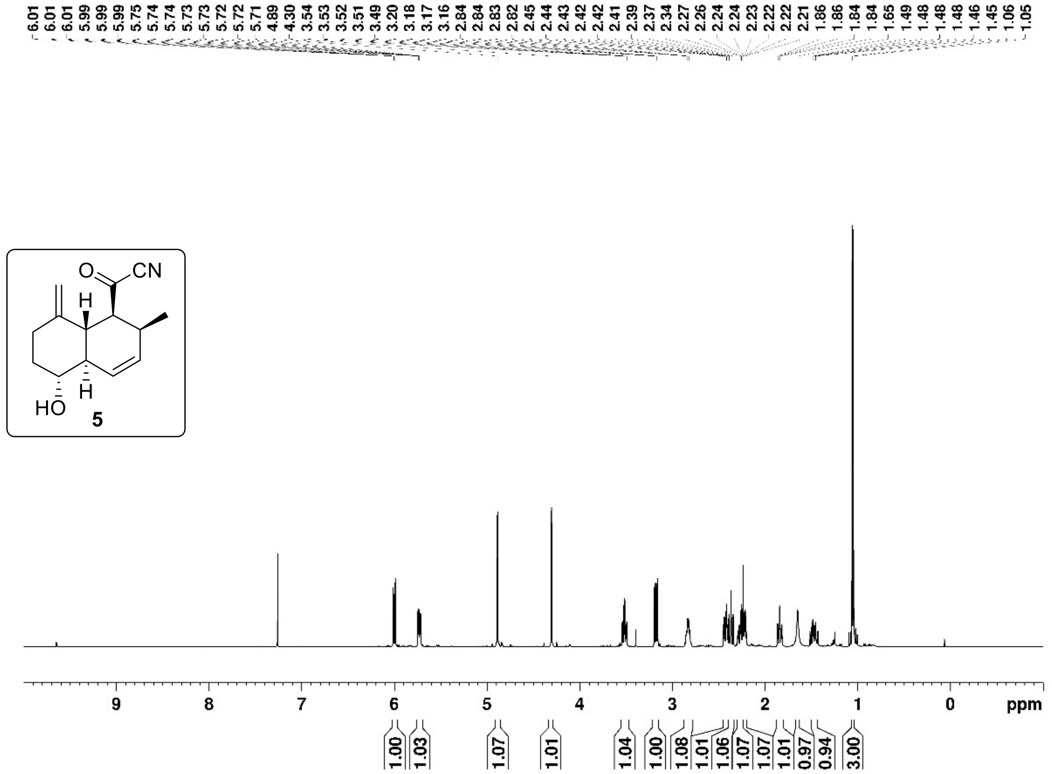
6.01
6.01
6.01
5.99
5.99
5.99
5.75
5.74
5.74
5.73
5.73
5.72
5.72
5.71
4.89
4.30
3.54
3.53
3.52
3.51
3.49
3.20
3.18
3.17
3.16
2.84
2.84
2.83
2.82
2.45
2.44
2.43
2.42
2.42
2.41
2.39
2.37
2.34
2.27
2.26
2.24
2.24
2.23
2.22
2.22
2.21
1.86
1.86
1.84
1.84
1.65
1.49
1.48
1.48
1.48
1.46
1.45
1.06
1.05
O
CN
H
H
HO
5
9
8
7
6
5
4
3
2
1
0
ppm
1.00
1.03
1.07
1.01
1.04
1.00
1.08
1.01
1.06
1.07
1.07
1.01
0.97
0.94
3.00

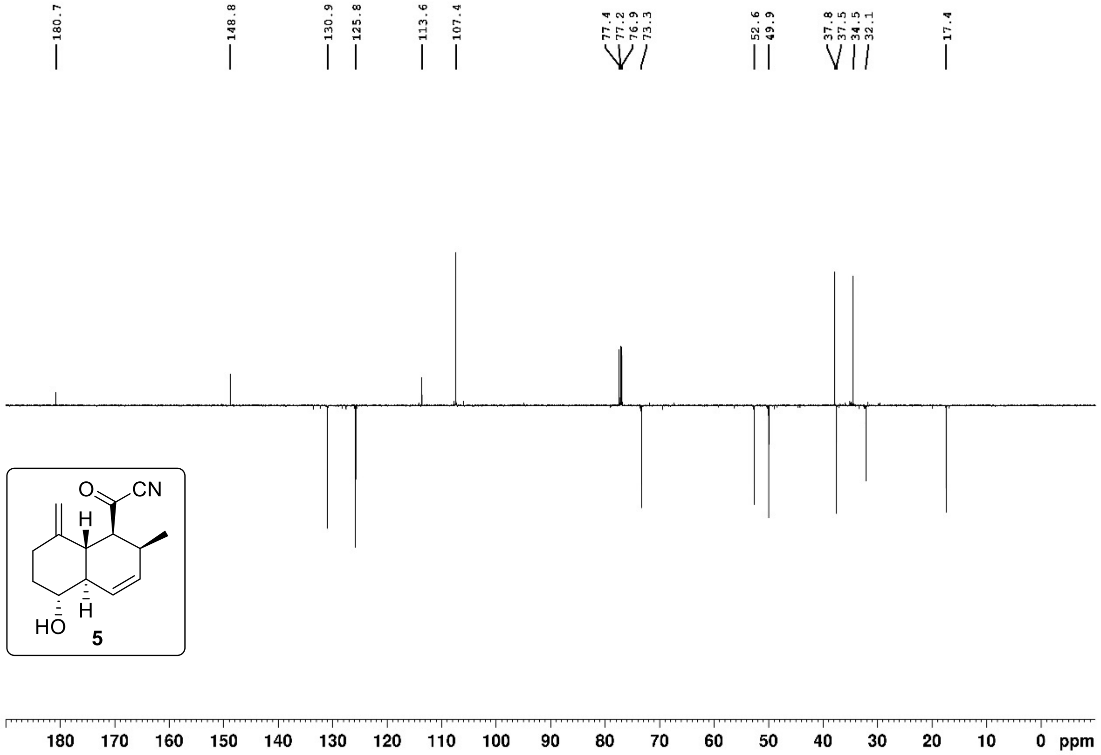
180.7
148.8
130.9
125.8
113.6
107.4
77.4
77.2
76.9
73.3
52.6
49.9
37.8
37.5
34.5
32.1
17.4
O
CN
H
H
HO
5
180
170
160
150
140
130
120
110
100
90
80
70
60
50
40
30
20
10
0
ppm

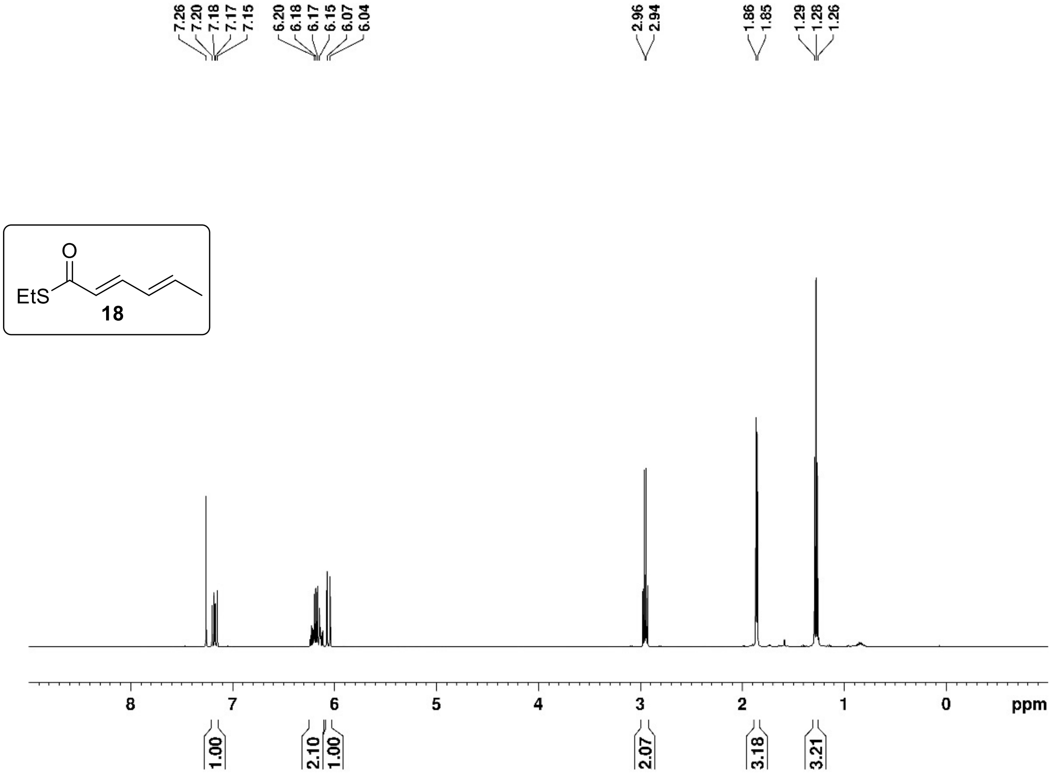
7.26
7.20
7.18
7.17
7.15
6.20
6.18
6.17
6.15
6.07
6.04
2.96
2.94
1.86
1.85
1.29
1.28
1.26
EtS
18
ppm
1.00
2.10
1.00
2.07
3.18
3.21

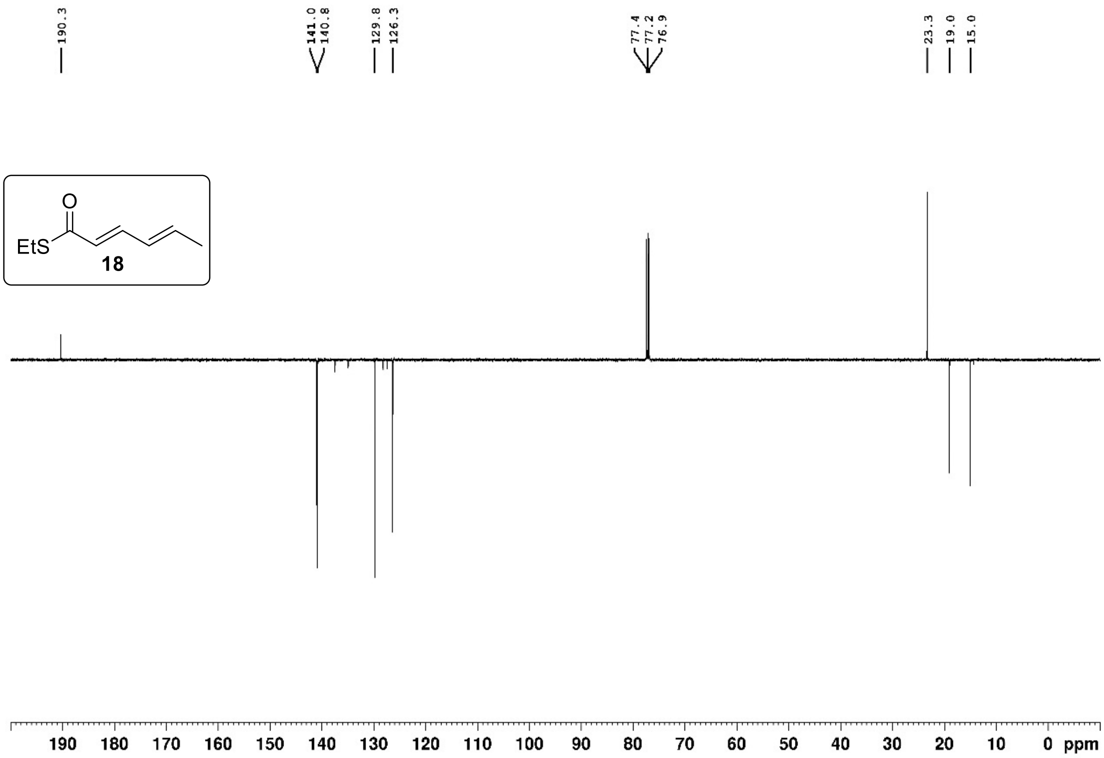
190.3
141.0
140.8
129.8
126.3
77.4
77.2
76.9
23.3
19.0
15.0
EtS
18
ppm

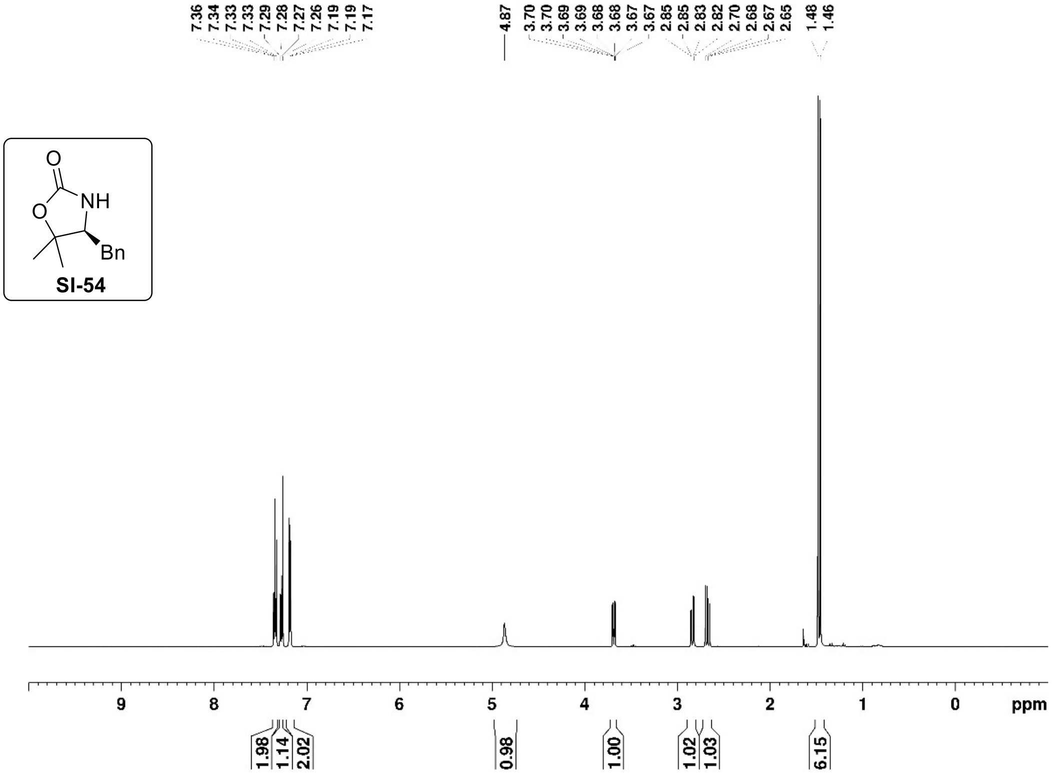
7.36 7.34 7.33 7.33 7.29 7.28 7.27 7.26 7.19 7.19 7.17
4.87
3.70 3.70 3.69 3.69 3.68 3.68 3.67 3.67 2.85 2.85 2.83 2.82 2.70 2.68 2.67 2.65
1.48 1.46
O
NH
O
Bn
SI-54
9 8 7 6 5 4 3 2 1 0 ppm
1.98 1.14 2.02 0.98 1.00 1.02 1.03 6.15

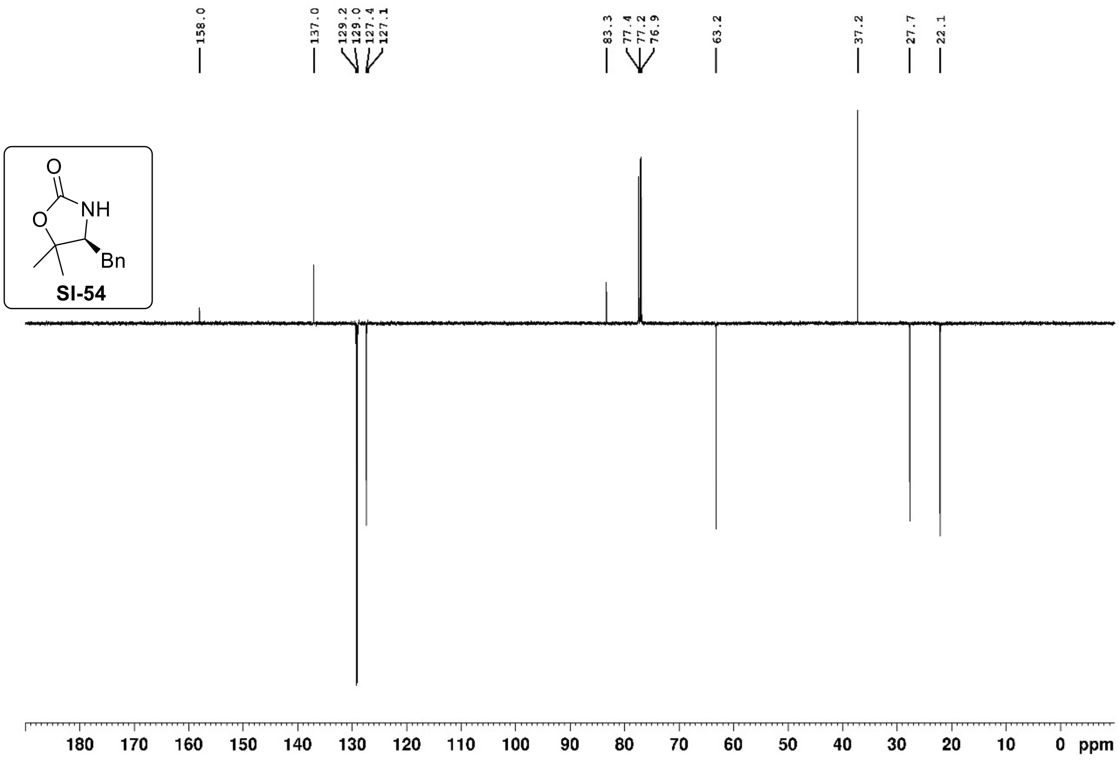
158.0
137.0 129.2 129.0 127.4 127.1
83.3 77.4 77.2 76.9
63.2
37.2 27.7 22.1
O
NH
O
Bn
SI-54
180 170 160 150 140 130 120 110 100 90 80 70 60 50 40 30 20 10 0 ppm

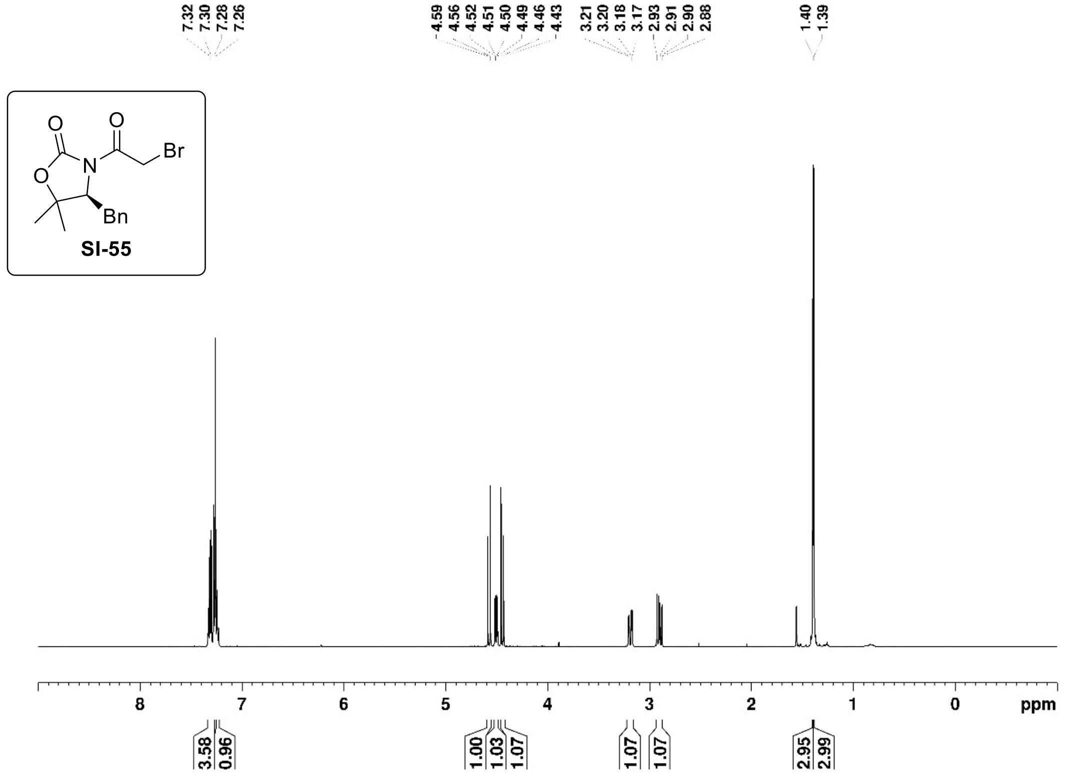
7.32
7.30
7.28
7.26
4.59
4.56
4.52
4.51
4.50
4.49
4.46
4.43
3.21
3.20
3.18
3.17
2.93
2.91
2.90
2.88
1.40
1.39
SI-55
ppm
3.58
0.96
1.00
1.03
1.07
1.07
1.07
2.95
2.99

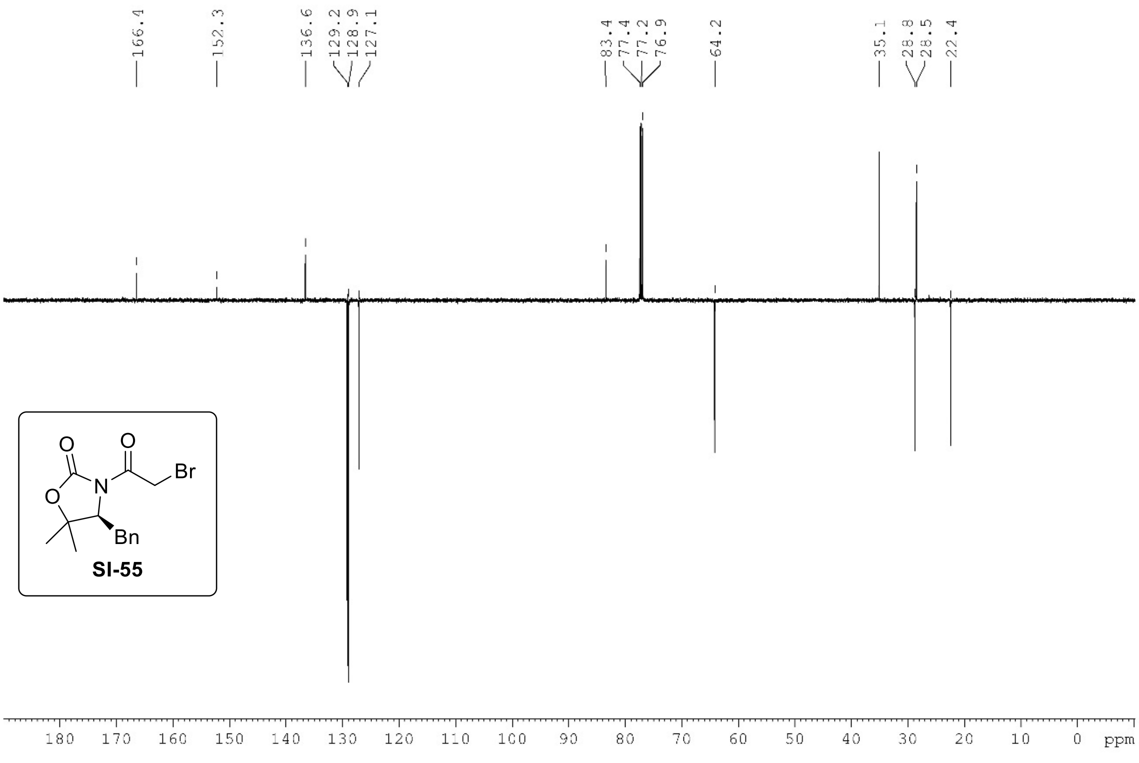
166.4
152.3
136.6
129.2
128.9
127.1
83.4
77.4
77.2
76.9
64.2
35.1
28.8
28.5
22.4
SI-55
ppm

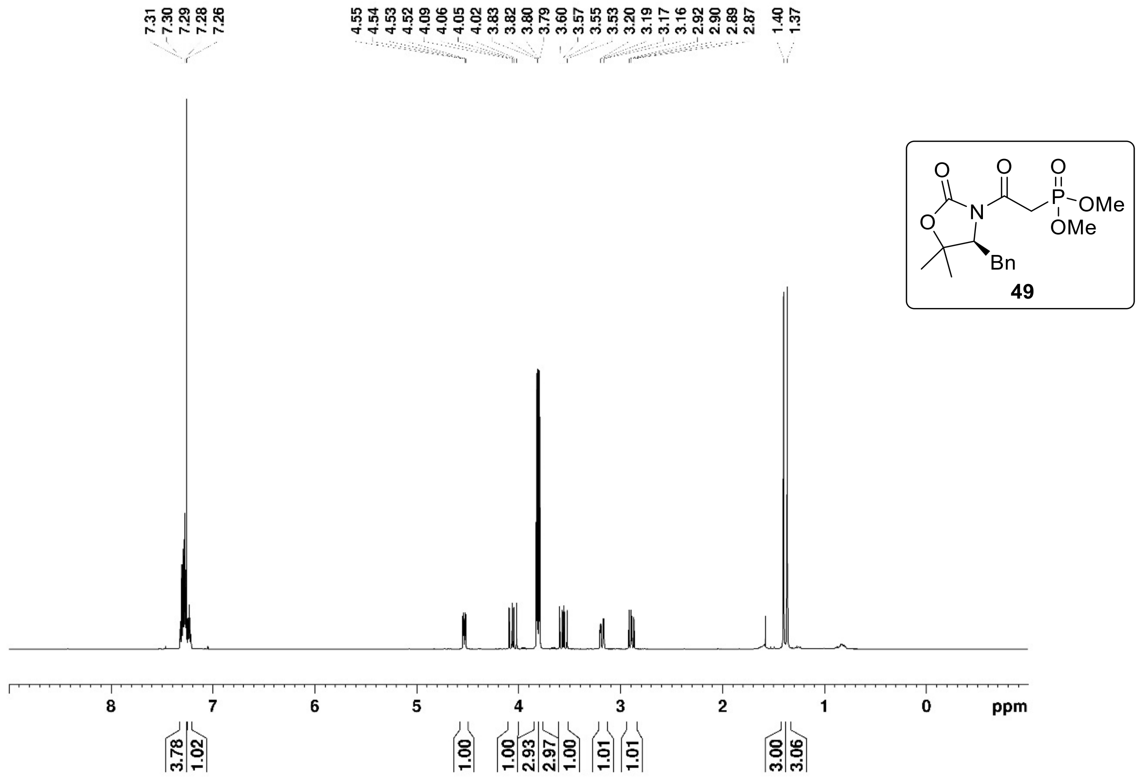
7.31 7.30 7.29 7.28 7.26
4.55 4.54 4.53 4.52 4.09 4.06 4.05 4.02 3.83 3.82 3.80 3.79 3.60 3.57 3.55 3.53 3.20 3.19 3.17 3.16 2.92 2.90 2.89 2.87
1.40 1.37
O
N
O
P
OMe
OMe
O
O
Bn
49
8 7 6 5 4 3 2 1 0 ppm
3.78 1.02 1.00 1.00 2.93 2.97 1.00 1.01 1.01 3.00 3.06

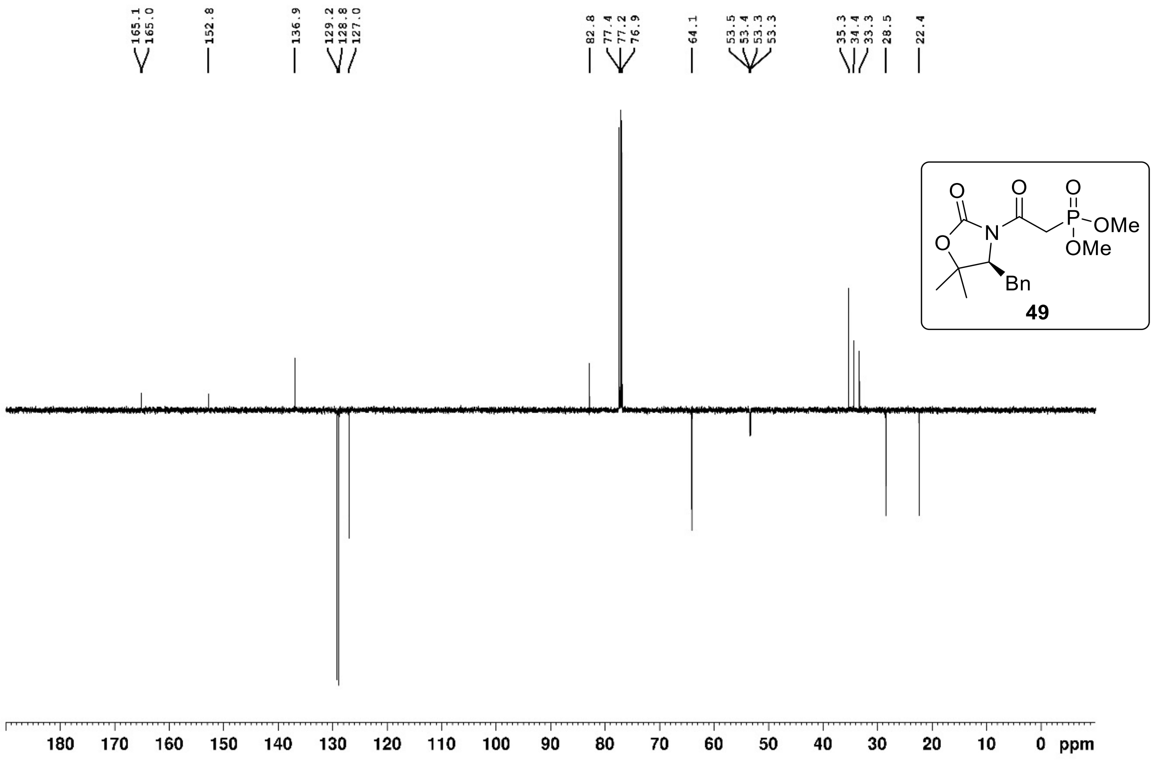
165.1 165.0 152.8 136.9 129.2 128.8 127.0 82.8 77.4 77.2 76.9 64.1 53.5 53.4 53.3 53.3 35.3 34.4 33.3 28.5 22.4
O
N
O
P
OMe
OMe
O
O
Bn
49
180 170 160 150 140 130 120 110 100 90 80 70 60 50 40 30 20 10 0 ppm

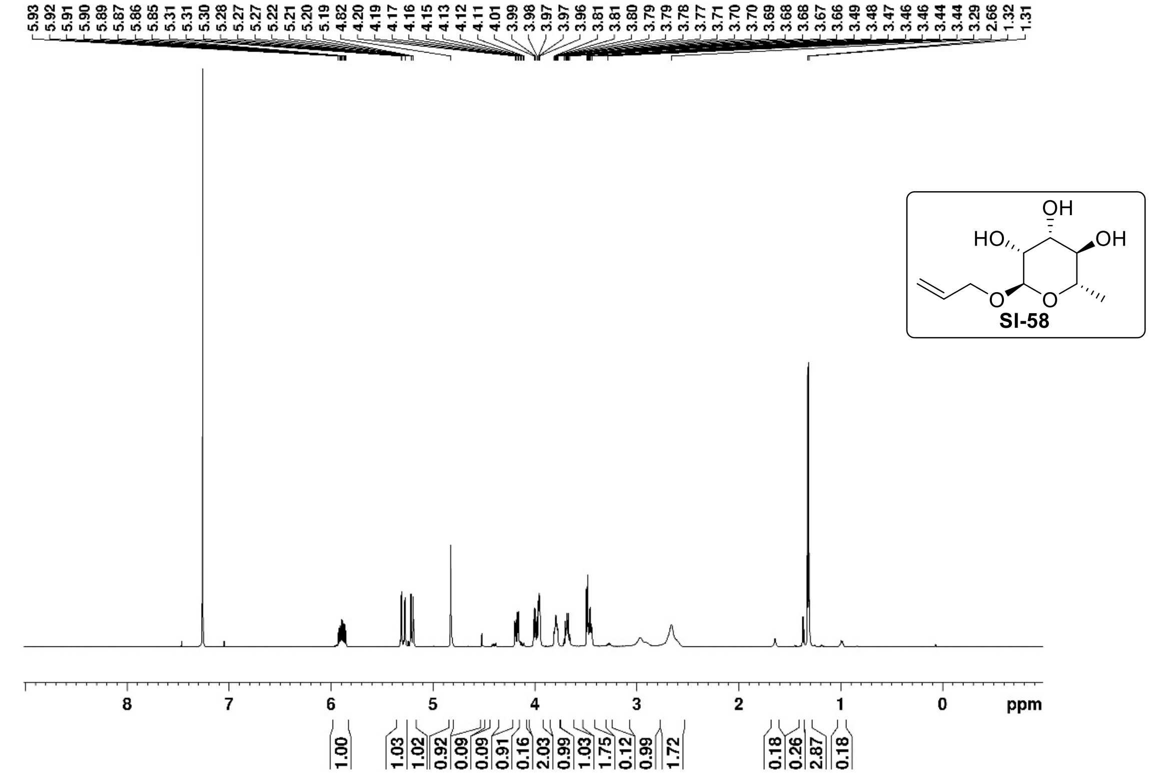
5.93 5.92 5.91 5.90 5.89 5.87 5.86 5.85 5.31 5.31 5.30 5.28 5.27 5.22 5.21 5.20 5.19 4.82 4.20 4.19 4.17 4.16 4.15 4.13 4.12 4.11 4.01 3.99 3.98 3.97 3.97 3.96 3.81 3.81 3.80 3.79 3.78 3.77 3.71 3.70 3.70 3.69 3.68 3.68 3.67 3.66 3.49 3.48 3.47 3.46 3.44 3.44 3.29 2.66 1.32 1.31
OH
HO
OH
O
O
SI-58
8 7 6 5 4 3 2 1 0 ppm
1.00 1.03 1.02 0.92 0.09 0.09 0.91 0.16 2.03 0.99 1.03 1.75 0.12 0.99 1.72 0.18 0.26 2.87 0.18

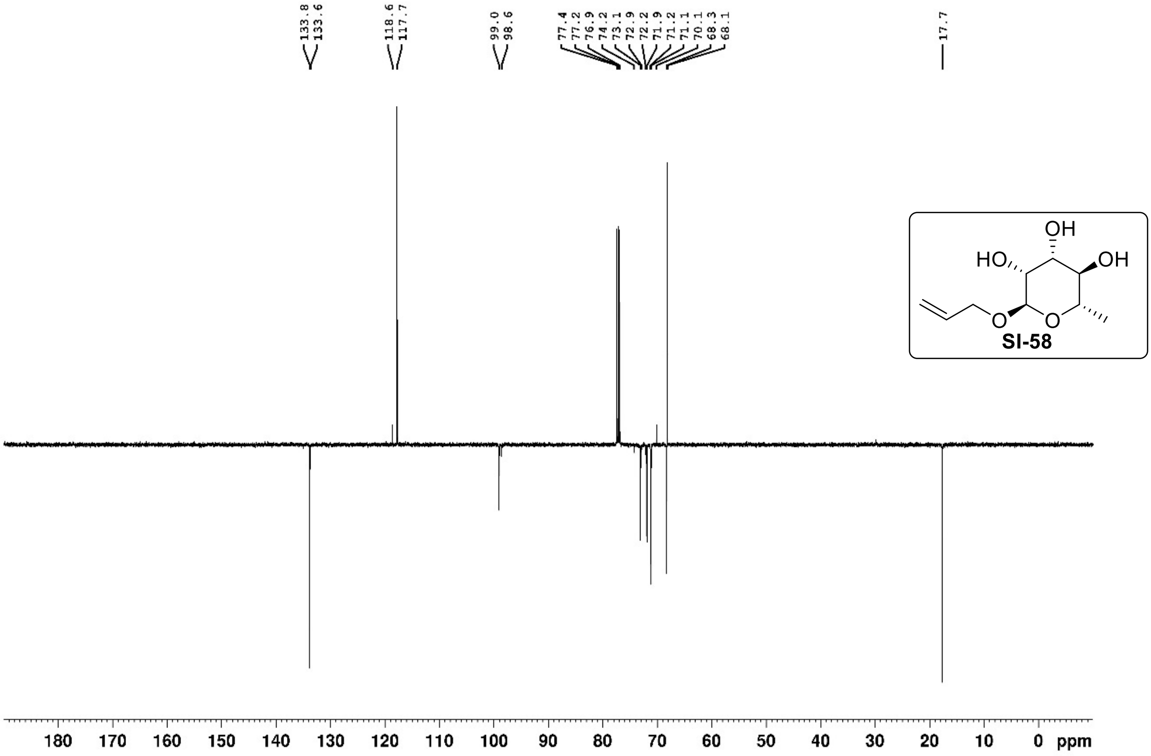
133.8 133.6 118.6 117.7 99.0 98.6 77.4 77.2 76.9 74.2 73.1 72.9 72.2 71.9 71.2 71.1 70.1 68.3 68.1 17.7
OH
HO
OH
O
O
SI-58
180 170 160 150 140 130 120 110 100 90 80 70 60 50 40 30 20 10 0 ppm

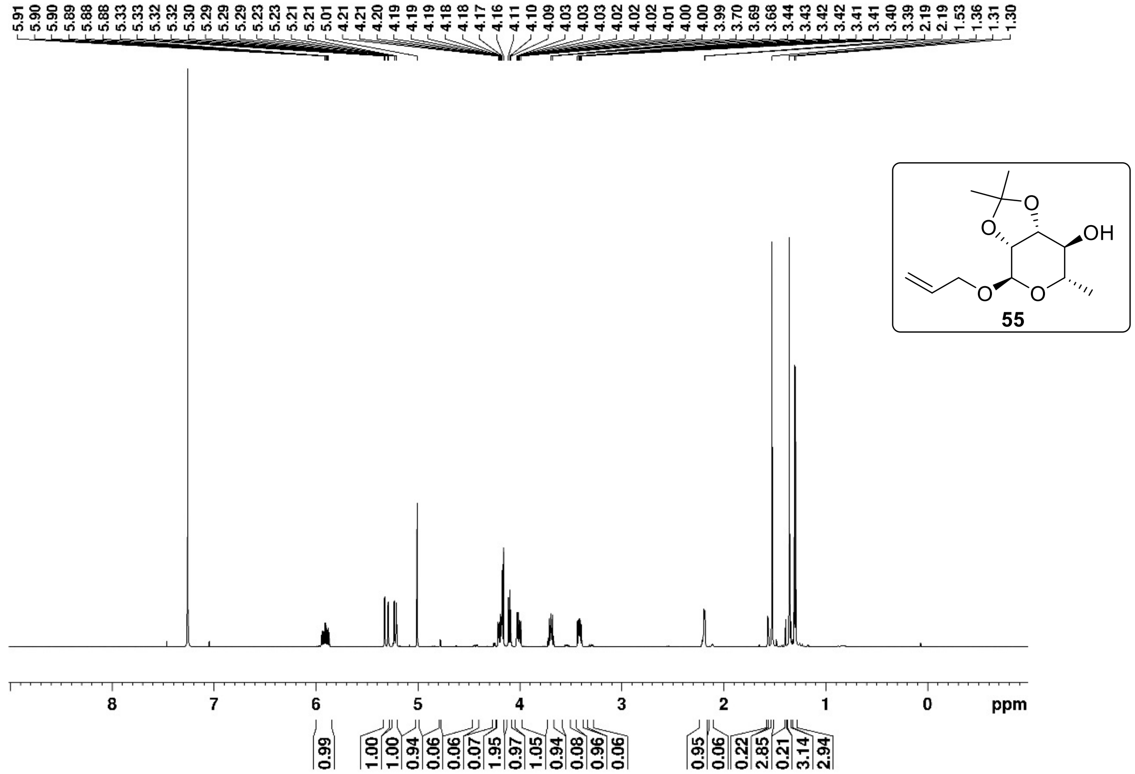

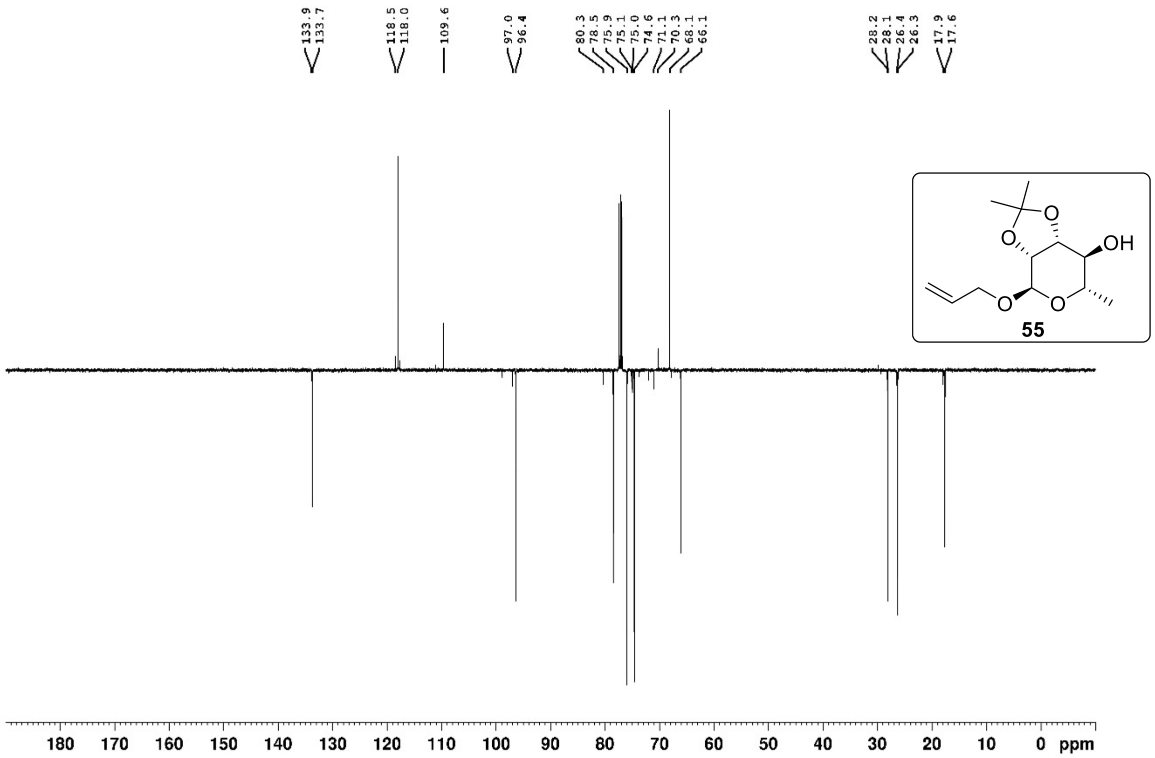

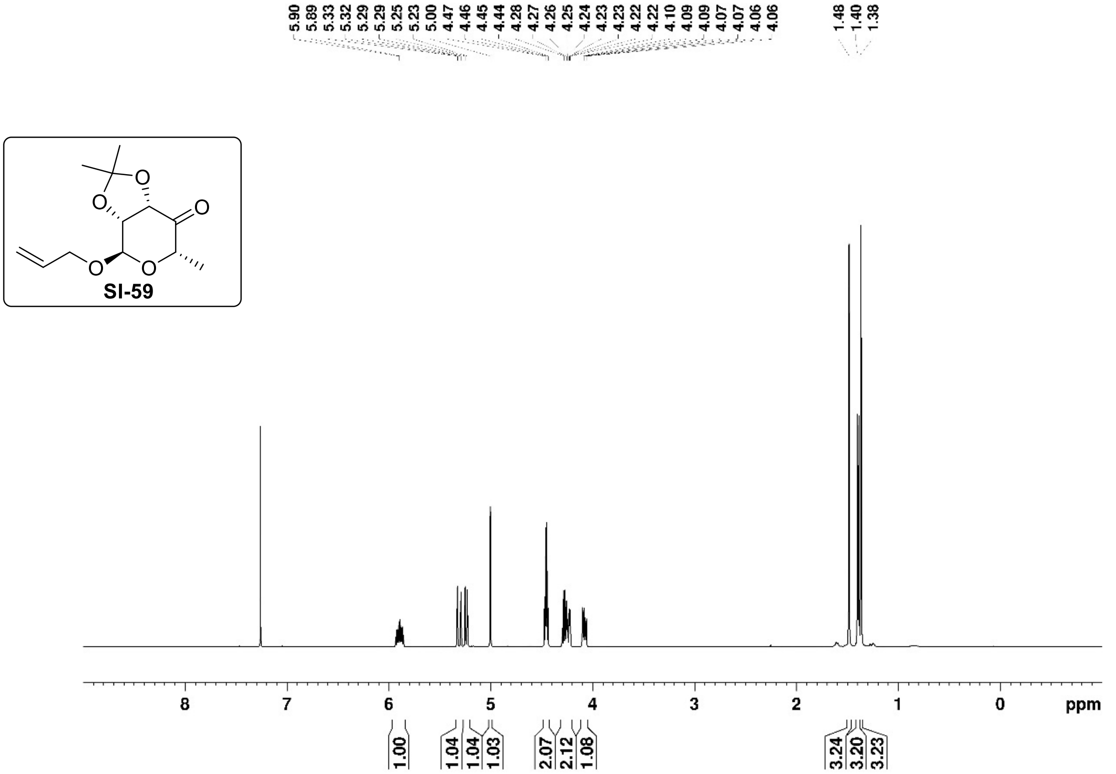
5.90 5.89 5.33 5.32 5.29 5.29 5.25 5.23 5.00 4.47 4.46 4.45 4.44 4.28 4.27 4.26 4.25 4.24 4.23 4.23 4.22 4.22 4.10 4.09 4.09 4.07 4.06 4.06 1.48 1.40 1.38
SI-59
8 7 6 5 4 3 2 1 0 ppm
1.00 1.04 1.04 1.03 2.07 2.12 1.08 3.24 3.20 3.23

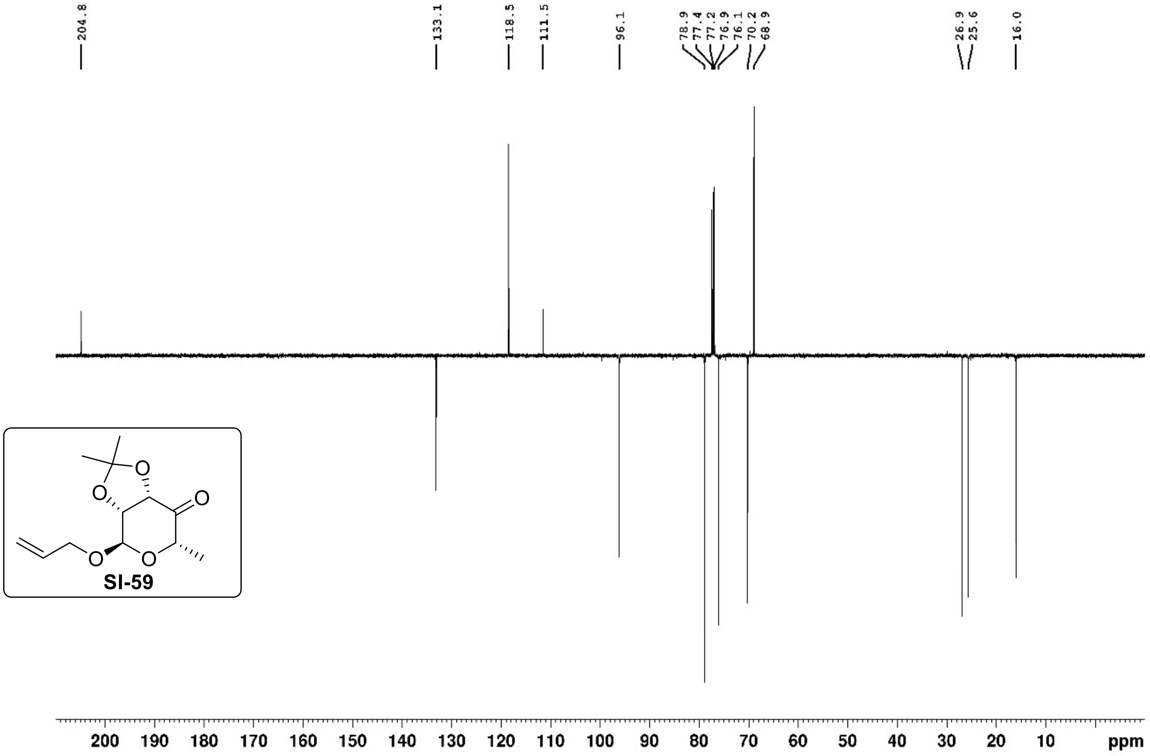
204.8 133.1 118.5 111.5 96.1 78.9 77.4 77.2 76.9 76.1 70.2 68.9 26.9 25.6 16.0
SI-59
200 190 180 170 160 150 140 130 120 110 100 90 80 70 60 50 40 30 20 10 ppm

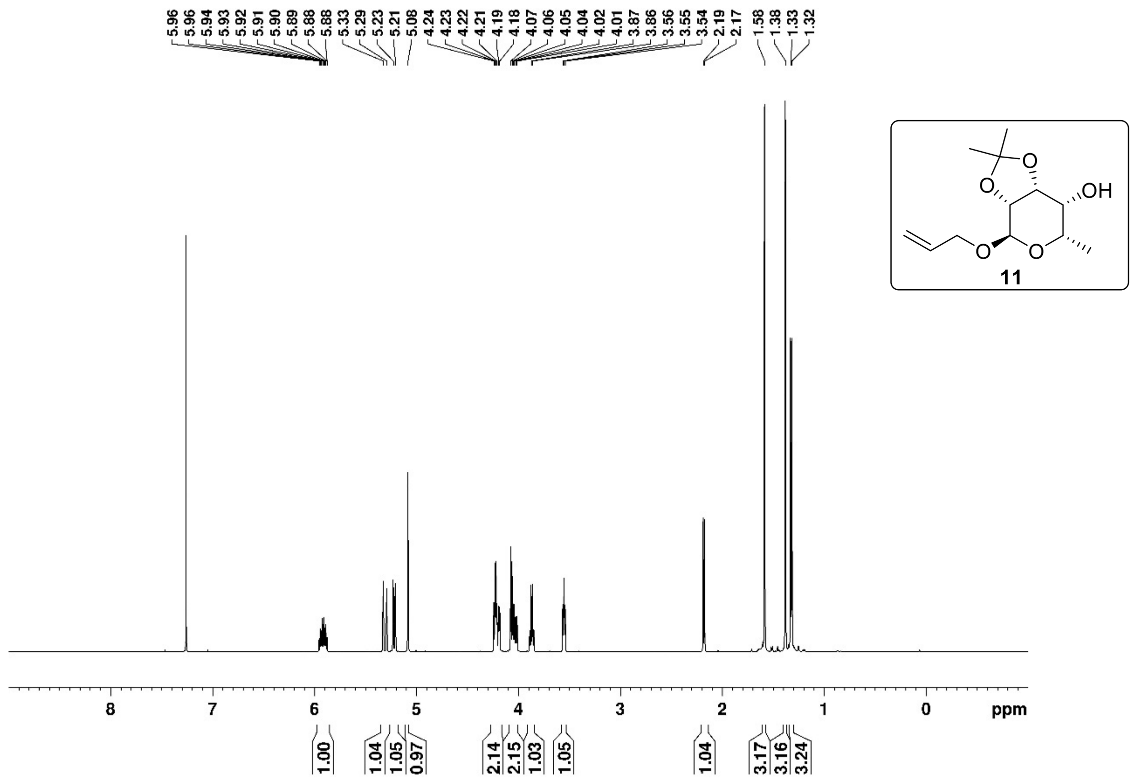
5.96 5.96 5.94 5.93 5.92 5.91 5.90 5.89 5.88 5.88 5.33 5.29 5.23 5.21 5.08 4.24 4.23 4.22 4.21 4.19 4.18 4.07 4.06 4.05 4.04 4.02 4.01 3.87 3.86 3.56 3.55 3.54 2.19 2.17 1.58 1.38 1.33 1.32
8 7 6 5 4 3 2 1 0 ppm
1.00 1.04 1.05 0.97 2.14 2.15 1.03 1.05 1.04 3.17 3.16 3.24
OH
11

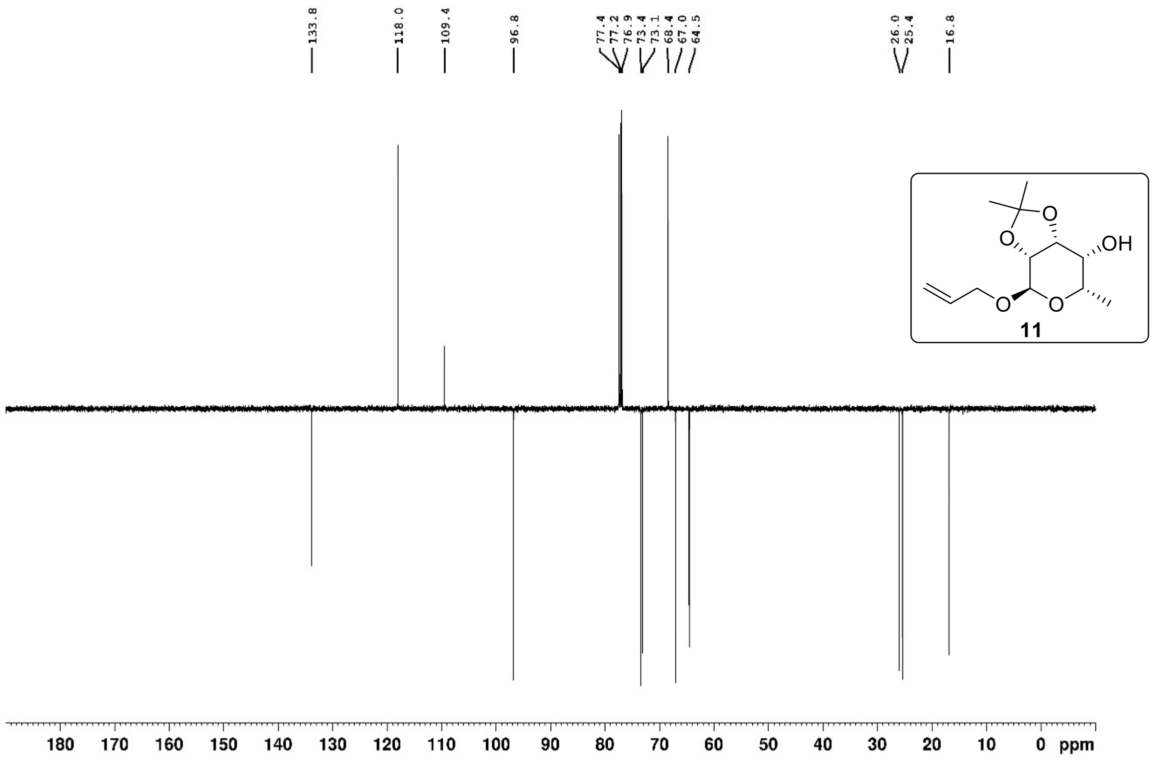
133.8 118.0 109.4 96.8 77.4 77.2 76.9 73.4 73.1 68.4 67.0 64.5 26.0 25.4 16.8
180 170 160 150 140 130 120 110 100 90 80 70 60 50 40 30 20 10 0 ppm
OH
11

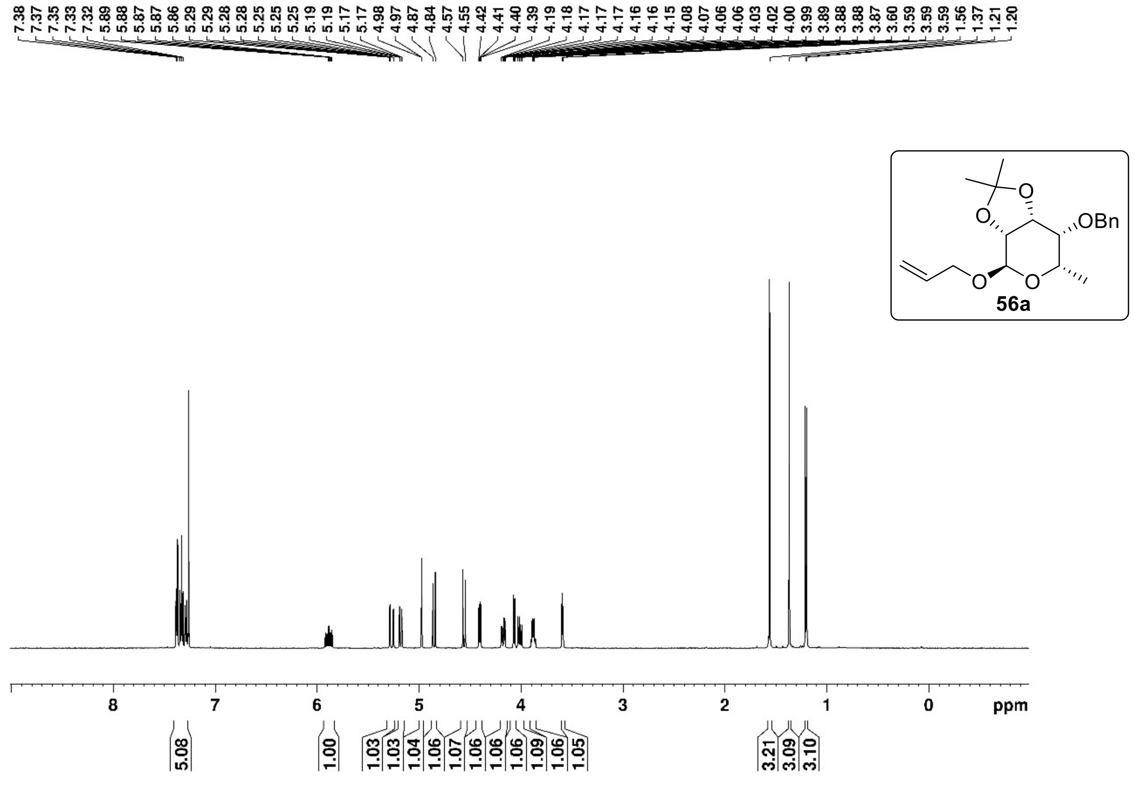
7.38 7.37 7.35 7.33 7.32 5.89 5.88 5.87 5.87 5.86 5.29 5.29 5.28 5.25 5.25 5.25 5.19 5.19 5.17 5.17 4.98 4.97 4.87 4.84 4.57 4.55 4.42 4.41 4.40 4.39 4.19 4.18 4.17 4.17 4.17 4.16 4.16 4.15 4.08 4.07 4.06 4.06 4.03 4.02 4.00 3.99 3.89 3.88 3.88 3.87 3.60 3.59 3.59 3.59 1.56 1.37 1.21 1.20
OBn
56a
8 7 6 5 4 3 2 1 0 ppm
5.08 1.00 1.03 1.03 1.04 1.06 1.07 1.06 1.06 1.06 1.09 1.06 1.05 3.21 3.09 3.10

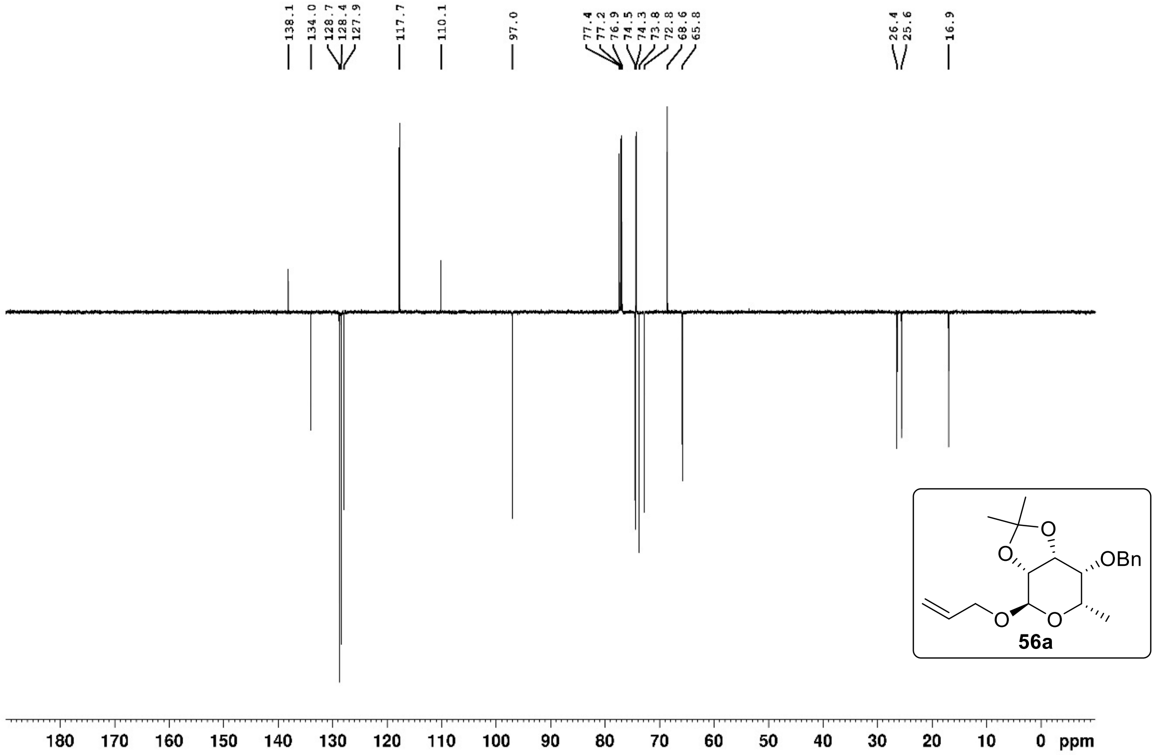
138.1 134.0 128.7 128.4 127.9 117.7 110.1 97.0 77.4 77.2 76.9 74.5 74.3 73.8 72.8 68.6 65.8 26.4 25.6 16.9
OBn
56a
180 170 160 150 140 130 120 110 100 90 80 70 60 50 40 30 20 10 0 ppm

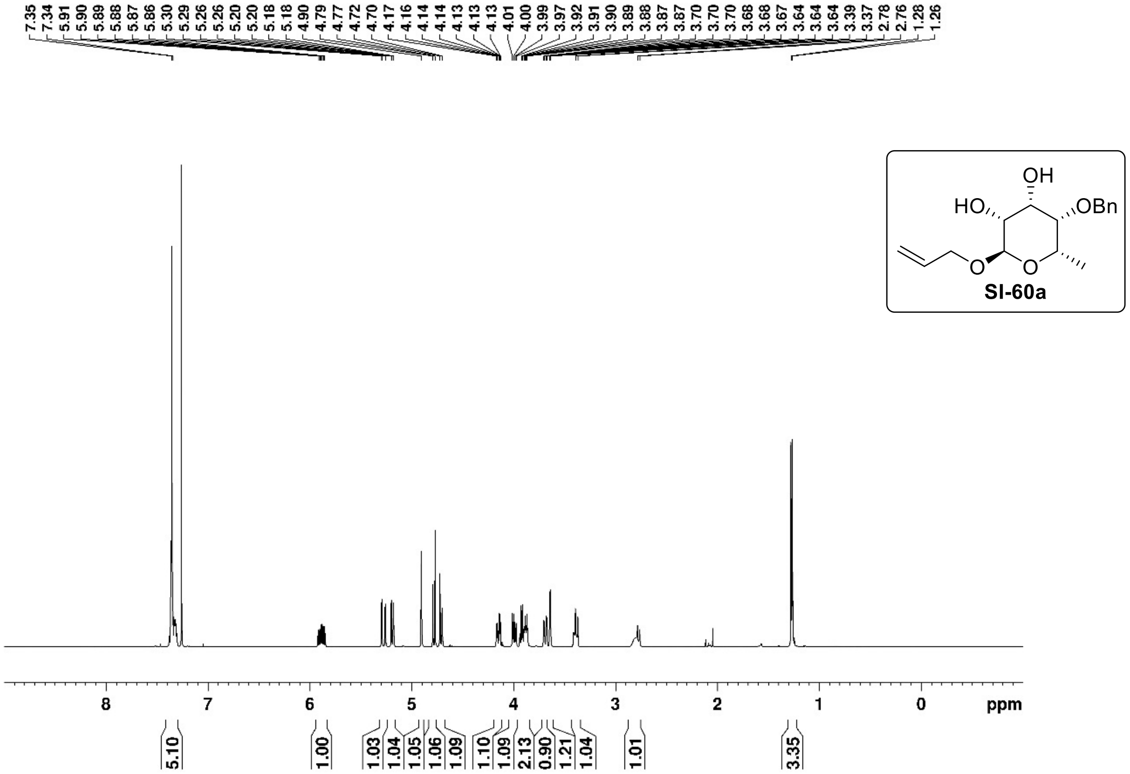
7.35 7.34 5.91 5.90 5.89 5.88 5.87 5.86 5.30 5.29 5.26 5.26 5.20 5.20 5.18 5.18 4.90 4.79 4.77 4.72 4.70 4.17 4.16 4.14 4.14 4.13 4.13 4.13 4.01 4.00 3.99 3.97 3.92 3.91 3.90 3.89 3.88 3.87 3.87 3.70 3.70 3.70 3.68 3.68 3.67 3.64 3.64 3.64 3.39 3.37 2.78 2.76 1.28 1.26
OH
HO
OBn
O
O
SI-60a
8
7
6
5
4
3
2
1
0
ppm
5.10 1.00 1.03 1.04 1.05 1.06 1.09 1.10 1.09 2.13 0.90 1.21 1.04 1.01 3.35

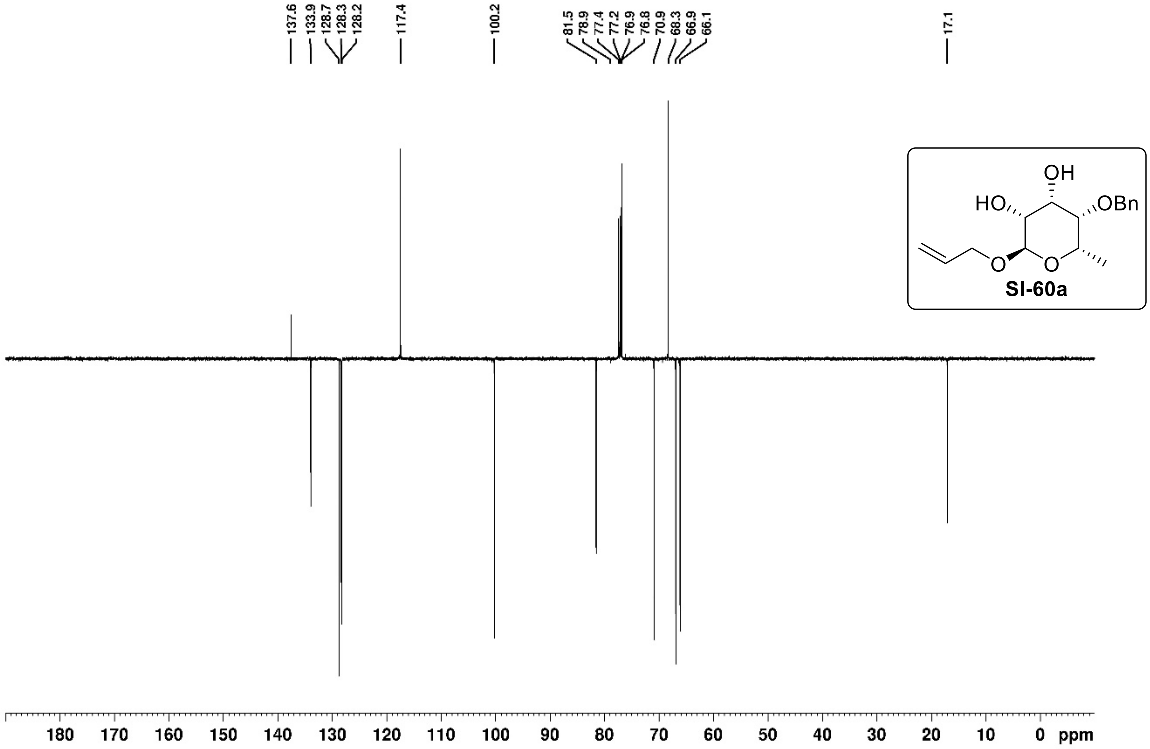
137.6 133.9 128.7 128.3 128.2 117.4 100.2 81.5 78.9 77.4 77.2 76.9 76.8 70.9 68.3 66.9 66.1 17.1
OH
HO
OBn
O
O
SI-60a
180 170 160 150 140 130 120 110 100 90 80 70 60 50 40 30 20 10 0 ppm

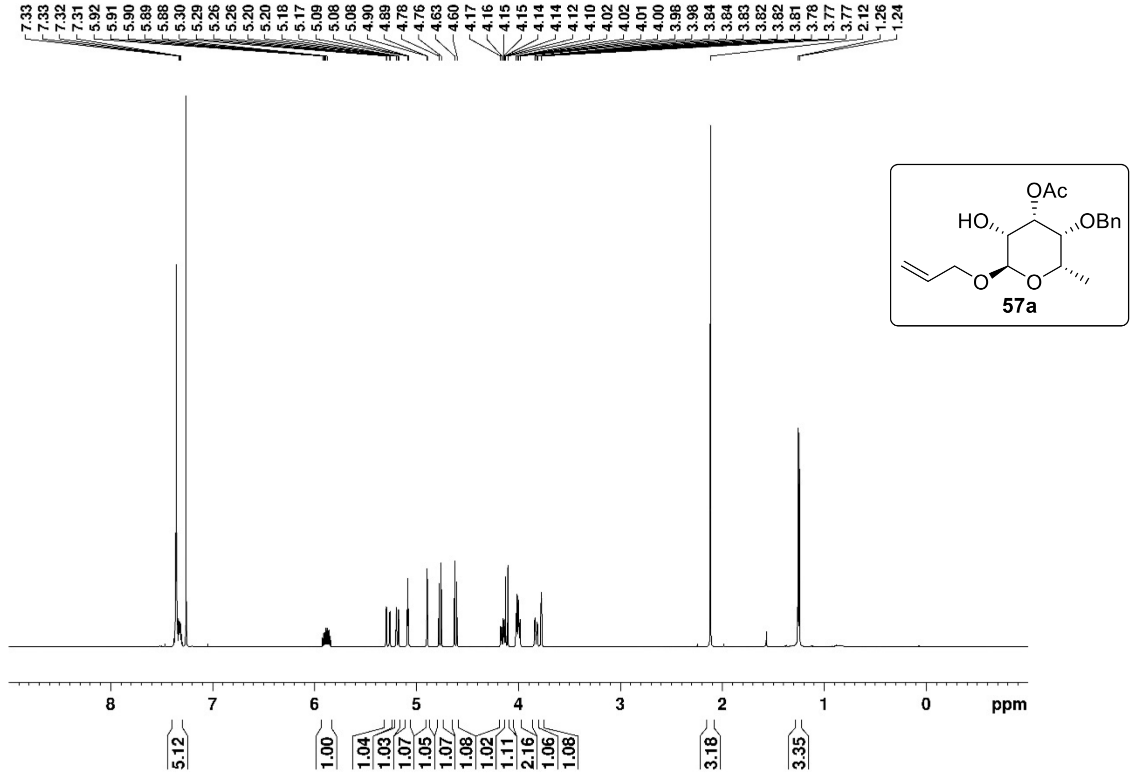

7.33 7.33 7.32 7.31 5.92 5.91 5.90 5.89 5.88 5.30 5.29 5.26 5.26 5.20 5.20 5.18 5.17 5.09 5.08 4.90 4.89 4.78 4.76 4.63 4.60 4.17 4.16 4.15 4.14 4.14 4.12 4.10 4.02 4.02 4.01 4.00 3.98 3.98 3.84 3.84 3.83 3.82 3.82 3.81 3.78 3.77 2.12 1.26 1.24
OAc
HO
OBn
O
O
57a
8 7 6 5 4 3 2 1 0 ppm
5.12 1.00 1.04 1.03 1.07 1.05 1.07 1.08 1.02 1.11 2.16 1.06 1.08 3.18 3.35

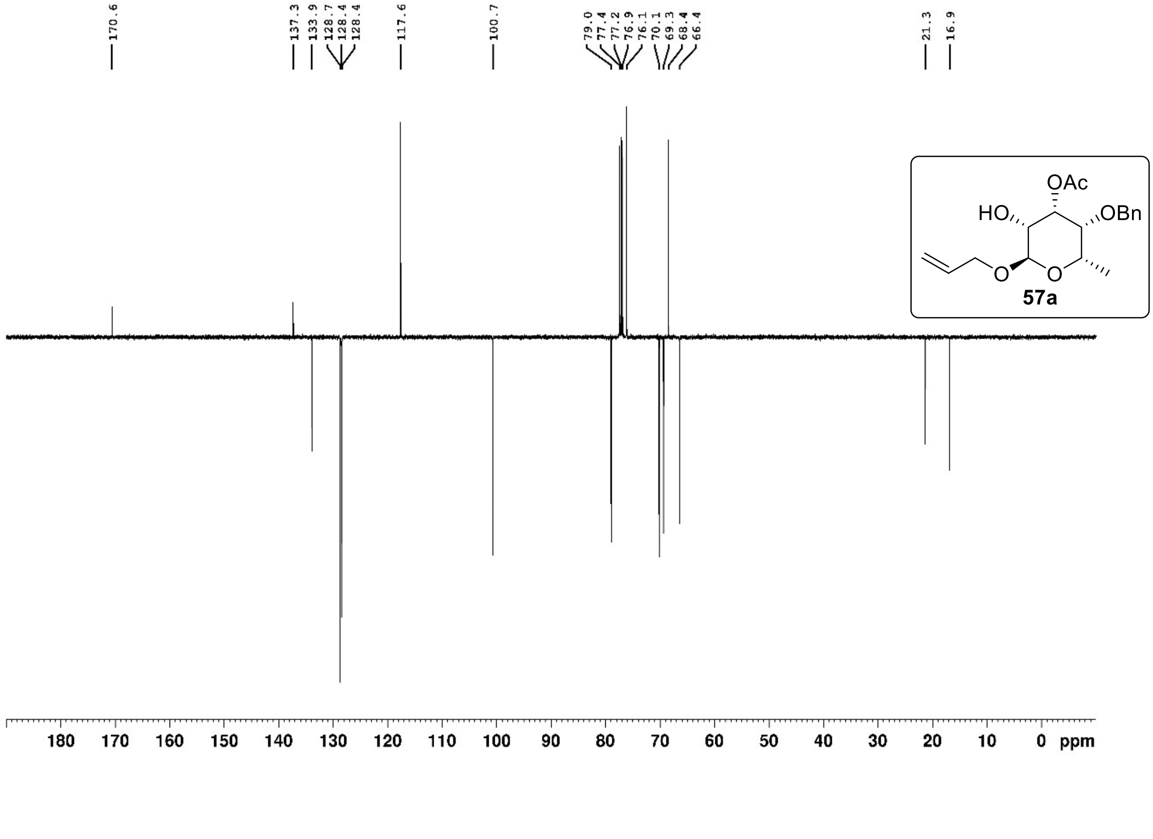

170.6 137.3 133.9 128.7 128.4 128.4 117.6 100.7 79.0 77.4 77.2 76.9 76.1 70.1 69.3 68.4 66.4 21.3 16.9
OAc
HO
OBn
O
O
57a
180 170 160 150 140 130 120 110 100 90 80 70 60 50 40 30 20 10 0 ppm

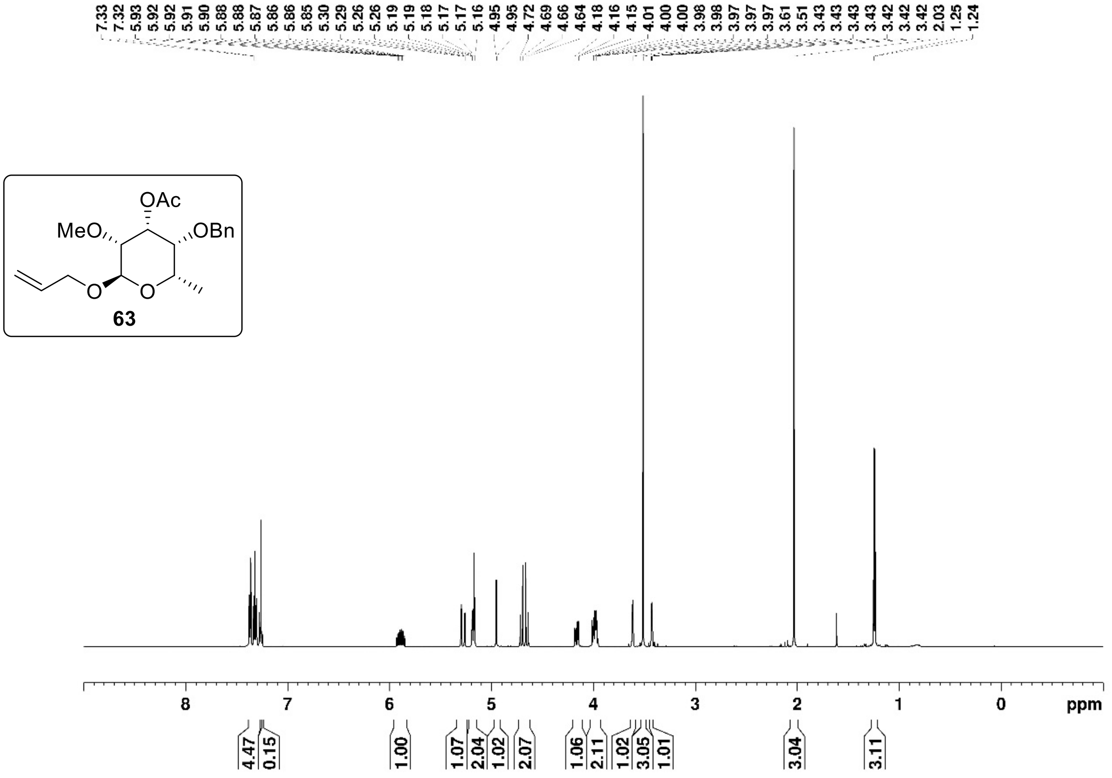

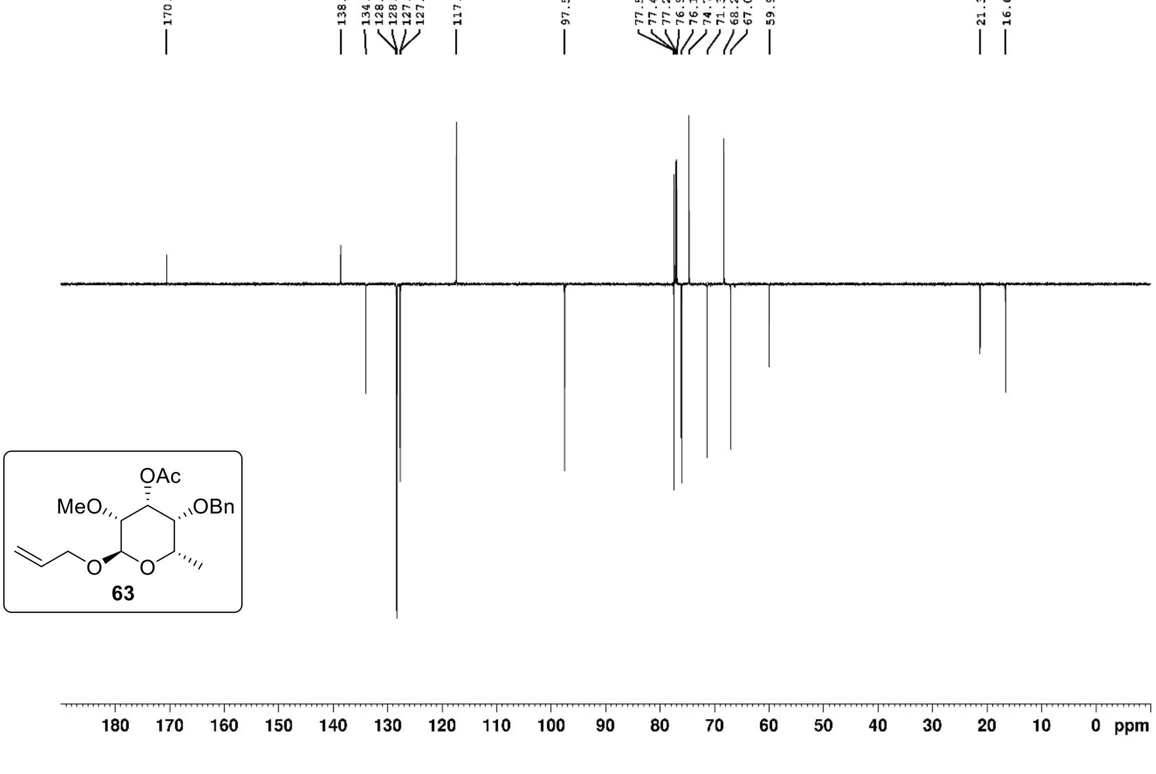

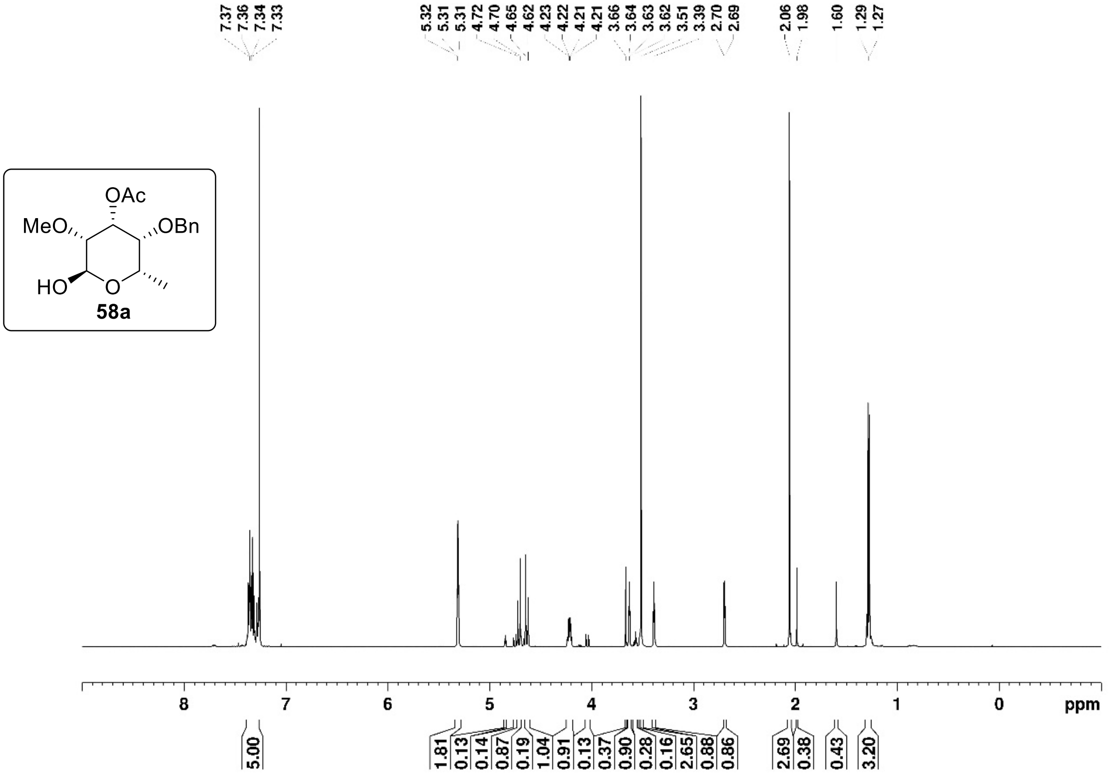
7.37 7.36 7.34 7.33
5.32 5.31 5.31 4.72 4.70 4.65 4.62 4.23 4.22 4.21 4.21 3.66 3.64 3.63 3.62 3.51 3.39 2.70 2.69
2.06 1.98 1.60 1.29 1.27
OAc
MeO
OBn
HO
O
58a
8 7 6 5 4 3 2 1 0 ppm
5.00
1.81 0.13 0.14 0.87 0.19 1.04 0.91 0.13 0.37 0.90 0.28 0.16 2.65 0.88 0.86
2.69 0.38 0.43 3.20

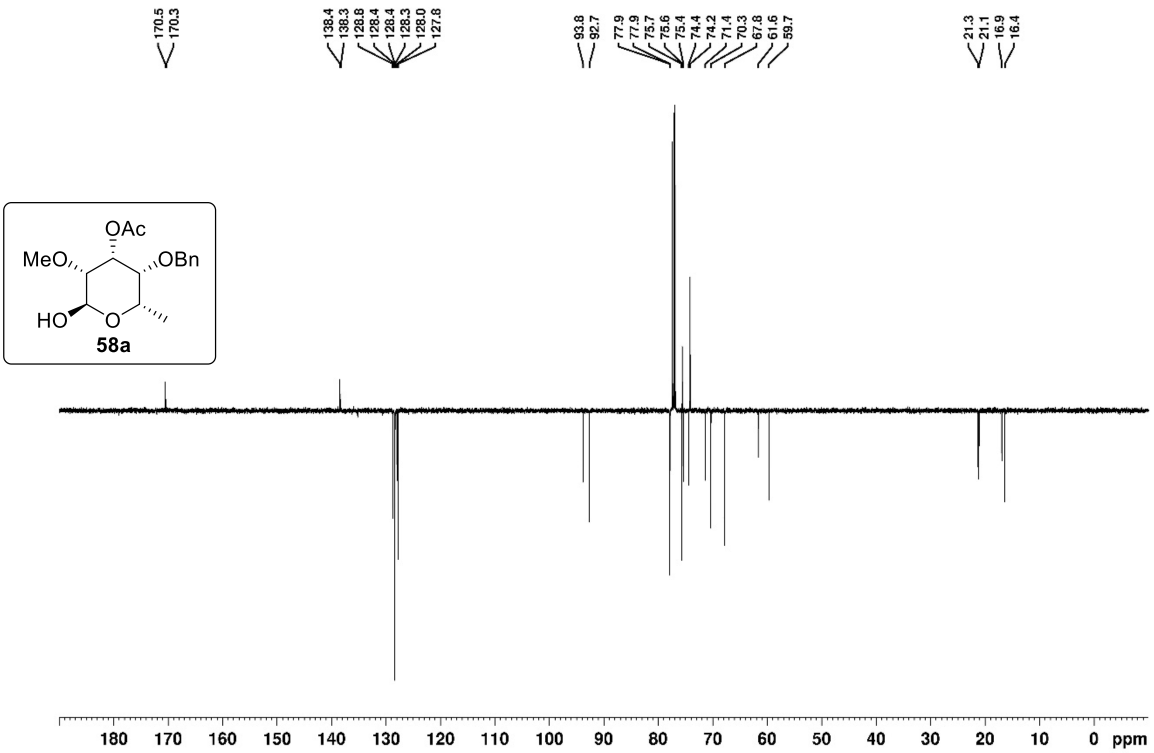
170.5 170.3
138.4 138.3 128.8 128.4 128.4 128.3 128.0 127.8
93.8 92.7
77.9 77.9 75.7 75.6 75.4 74.4 74.2 71.4 70.3 67.8 61.6 59.7
21.3 21.1 16.9 16.4
OAc
MeO
OBn
HO
O
58a
180 170 160 150 140 130 120 110 100 90 80 70 60 50 40 30 20 10 0 ppm

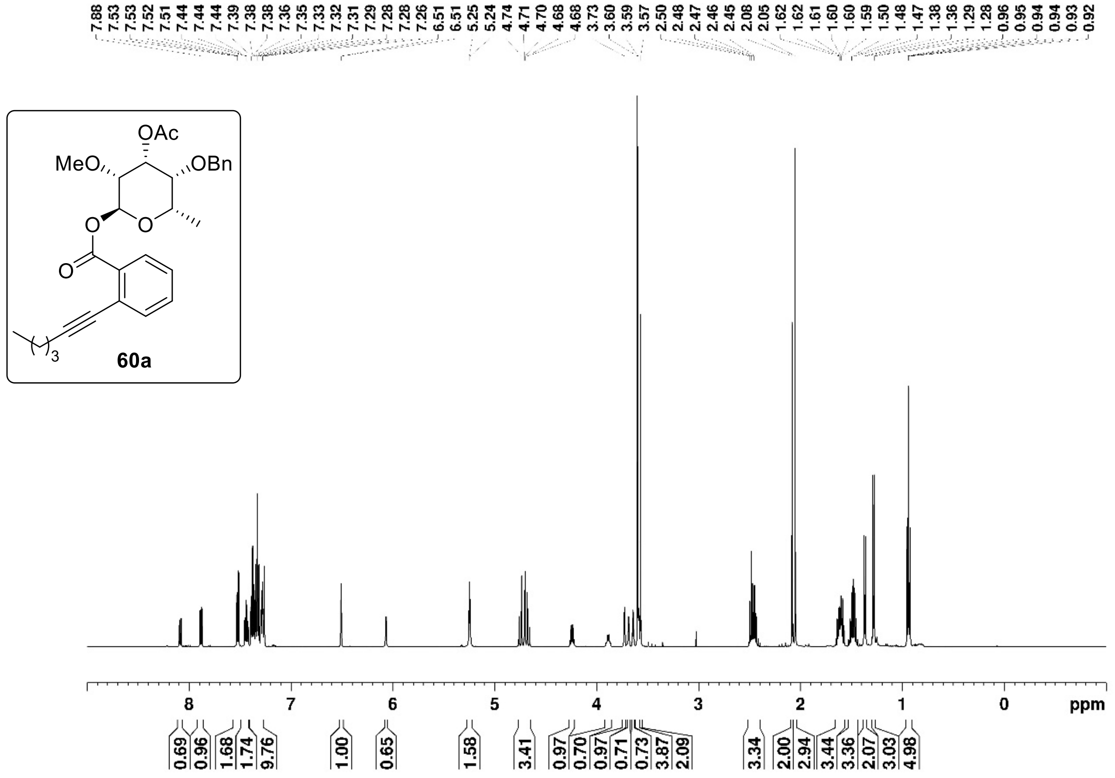
7.88 7.53 7.53 7.52 7.51 7.44 7.44 7.44 7.39 7.38 7.38 7.36 7.35 7.33 7.32 7.31 7.29 7.28 7.28 7.26 6.51 6.51 5.25 5.24 4.74 4.71 4.70 4.68 4.68 3.73 3.60 3.59 3.57 2.50 2.48 2.47 2.46 2.45 2.08 2.05 1.62 1.62 1.61 1.60 1.60 1.59 1.50 1.48 1.47 1.38 1.36 1.29 1.28 0.96 0.95 0.94 0.94 0.93 0.92
OAc
MeO
OBn
O
O
O
3
60a
8
7
6
5
4
3
2
1
0
ppm
0.69 0.96 1.68 1.74 9.76 1.00 0.65 1.58 3.41 0.97 0.70 0.97 0.71 0.73 3.87 2.09 3.34 2.00 2.94 3.44 3.36 2.07 3.03 4.98

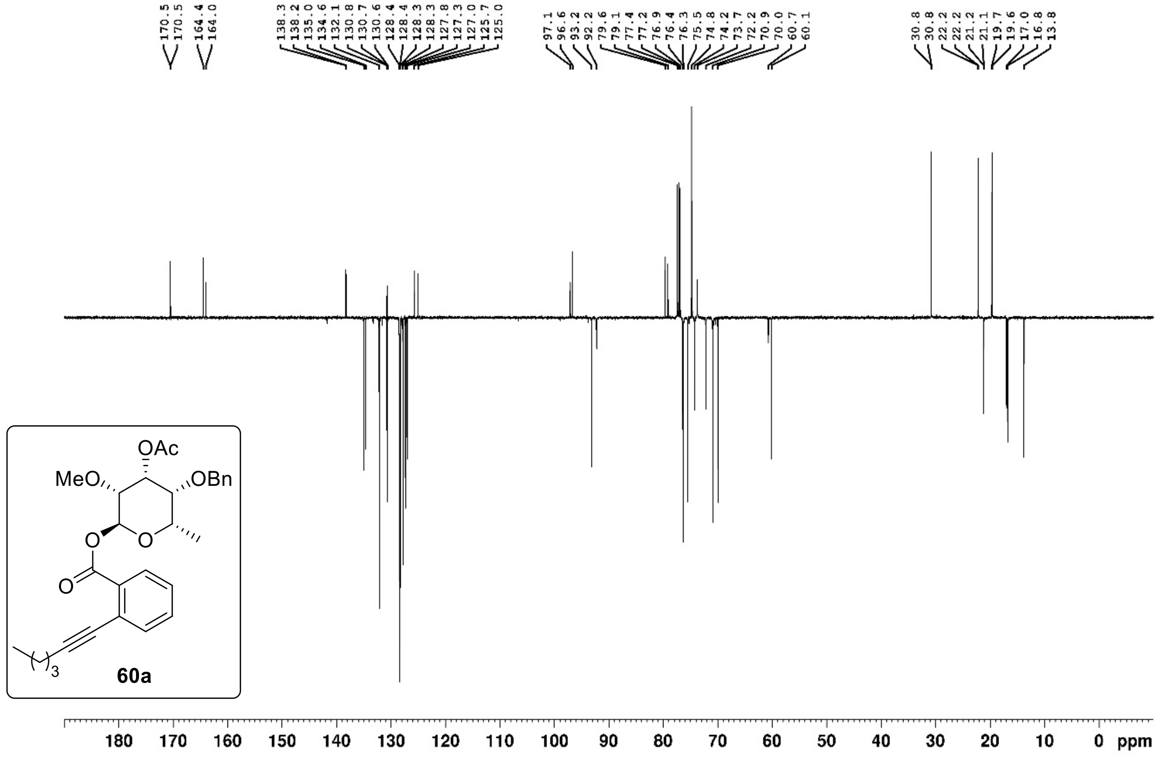
170.5 170.5 164.4 164.0 138.3 138.2 135.0 134.6 132.1 130.8 130.7 130.6 128.4 128.4 128.3 128.3 127.8 127.3 127.0 125.7 125.0 97.1 96.6 93.2 92.2 79.6 79.1 77.4 77.2 76.9 76.4 76.3 75.5 74.8 74.2 73.7 72.2 70.9 70.0 60.7 60.1 30.8 30.8 22.2 21.2 21.1 19.7 19.6 17.0 16.8 13.8
OAc
MeO
OBn
O
O
O
3
60a
180
170
160
150
140
130
120
110
100
90
80
70
60
50
40
30
20
10
0
ppm

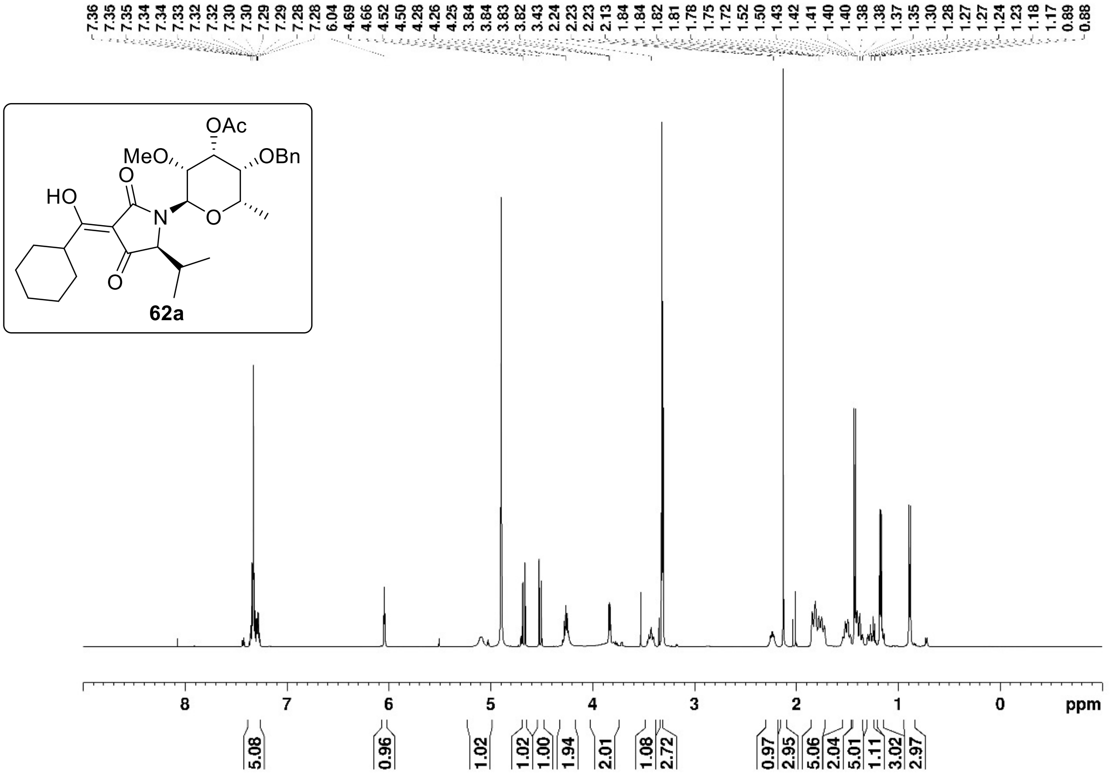
7.36 7.35 7.35 7.34 7.34 7.33 7.32 7.32 7.30 7.30 7.29 7.29 7.28 7.28 6.04 4.69 4.66 4.52 4.50 4.28 4.26 4.25 3.84 3.84 3.83 3.82 3.43 2.24 2.23 2.13 1.84 1.84 1.82 1.81 1.78 1.75 1.72 1.52 1.50 1.43 1.42 1.41 1.40 1.40 1.38 1.38 1.37 1.35 1.30 1.28 1.27 1.27 1.24 1.23 1.18 1.17 0.89 0.88
OAc
MeO
OBn
O
HO
N
O
O
62a
8 7 6 5 4 3 2 1 0 ppm
5.08 0.96 1.02 1.02 1.00 1.94 2.01 1.08 2.72 0.97 2.95 5.06 2.04 5.01 1.11 3.02 2.97

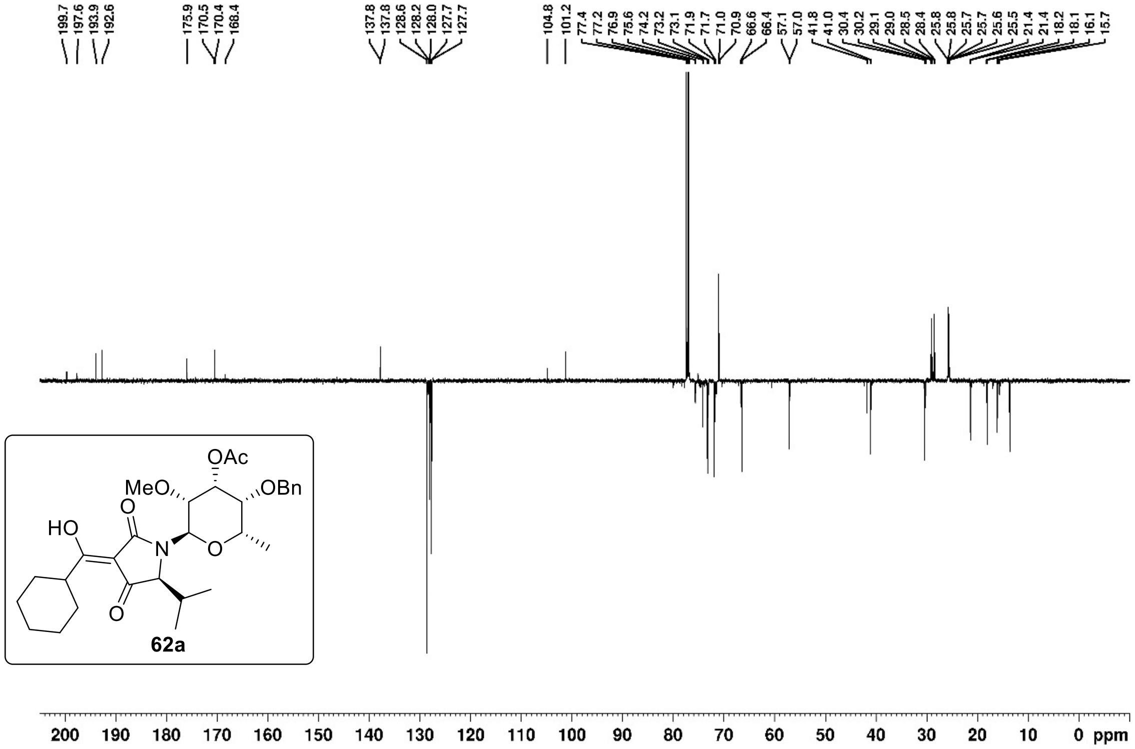
199.7 197.6 193.9 192.6 175.9 170.5 170.4 168.4 137.8 137.8 128.6 128.2 128.0 127.7 127.7 104.8 101.2 77.4 77.2 76.9 75.6 74.2 73.2 73.1 71.9 71.7 71.0 70.9 66.6 66.4 57.1 57.0 41.8 41.0 30.4 30.2 29.1 29.0 28.5 28.4 25.8 25.8 25.7 25.7 25.6 25.5 21.4 21.4 18.2 18.1 16.1 15.7
OAc
MeO
OBn
O
HO
N
O
O
62a
200 190 180 170 160 150 140 130 120 110 100 90 80 70 60 50 40 30 20 10 0 ppm

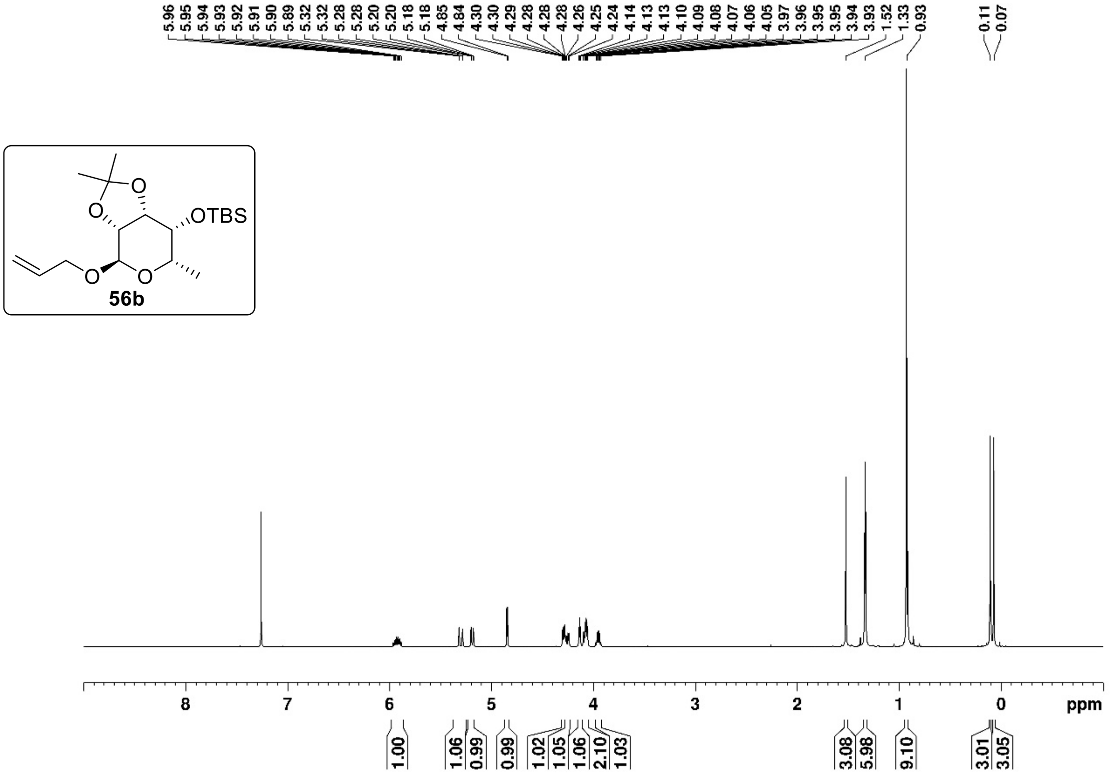
5.96 5.95 5.94 5.93 5.92 5.91 5.90 5.89 5.32 5.32 5.28 5.28 5.20 5.20 5.18 5.18 4.85 4.84 4.30 4.30 4.29 4.28 4.28 4.28 4.26 4.25 4.24 4.14 4.13 4.10 4.09 4.08 4.07 4.06 4.05 3.97 3.96 3.95 3.94 3.93 1.52 1.33 0.93
0.11 0.07
OTBS
56b
8 7 6 5 4 3 2 1 0 ppm
1.00 1.06 0.99 0.99 1.02 1.05 1.06 2.10 1.03 3.08 5.98 9.10 3.01 3.05

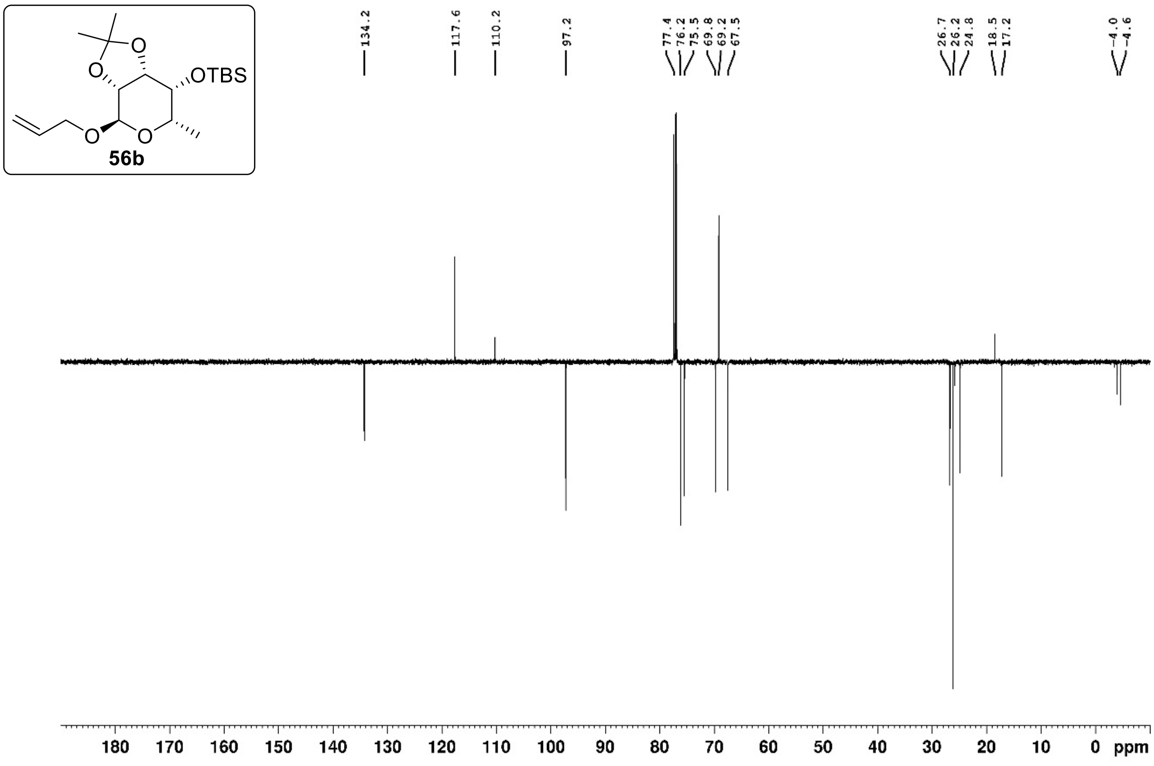
134.2 117.6 110.2 97.2 77.4 76.2 75.5 69.8 69.2 67.5 26.7 26.2 24.8 18.5 17.2 -4.0 -4.6
OTBS
56b
180 170 160 150 140 130 120 110 100 90 80 70 60 50 40 30 20 10 0 ppm

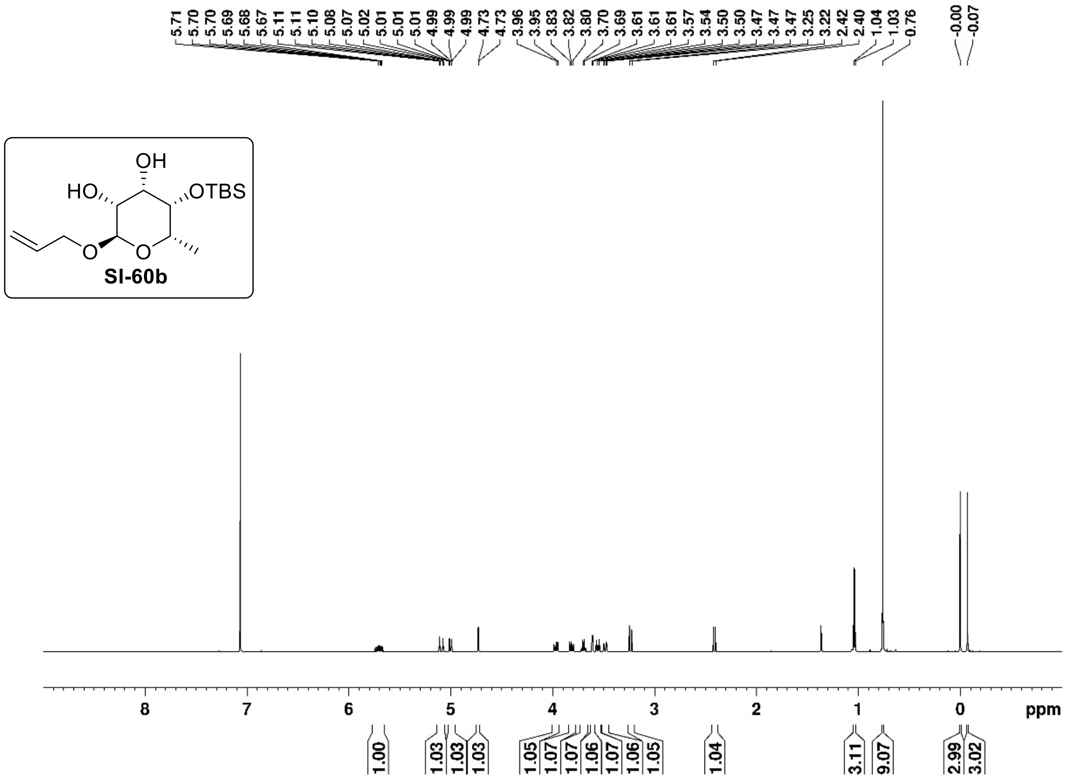
5.71 5.70 5.70 5.69 5.68 5.67 5.11 5.11 5.10 5.08 5.07 5.02 5.01 5.01 5.01 4.99 4.99 4.99 4.73 4.73 3.96 3.95 3.83 3.82 3.80 3.70 3.69 3.61 3.61 3.61 3.57 3.54 3.50 3.50 3.47 3.47 3.47 3.25 3.22 2.42 2.40 1.04 1.03 0.76 -0.00 -0.07
OH
HO
OTBS
O
SI-60b
8 7 6 5 4 3 2 1 0 ppm
1.00 1.03 1.03 1.03 1.05 1.07 1.07 1.06 1.07 1.06 1.05 1.04 3.11 9.07 2.99 3.02

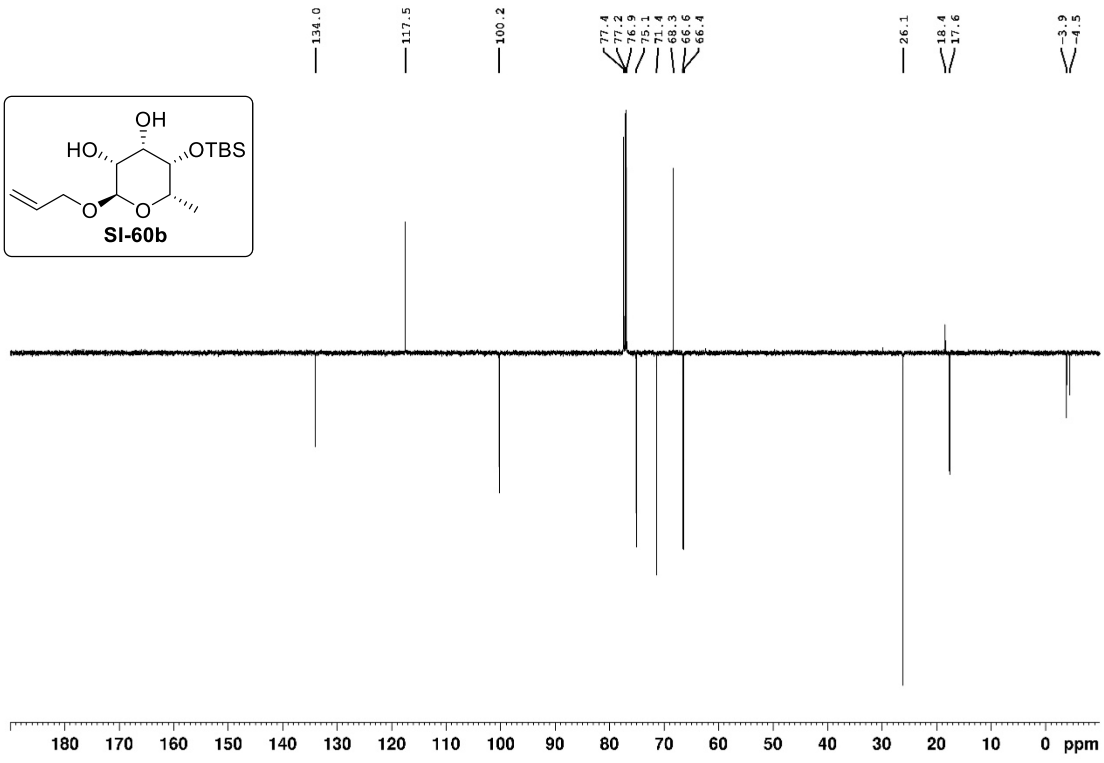
134.0 117.5 100.2 77.4 77.2 76.9 75.1 71.4 68.3 66.6 66.4 26.1 18.4 17.6 -3.9 -4.5
OH
HO
OTBS
O
SI-60b
180 170 160 150 140 130 120 110 100 90 80 70 60 50 40 30 20 10 0 ppm

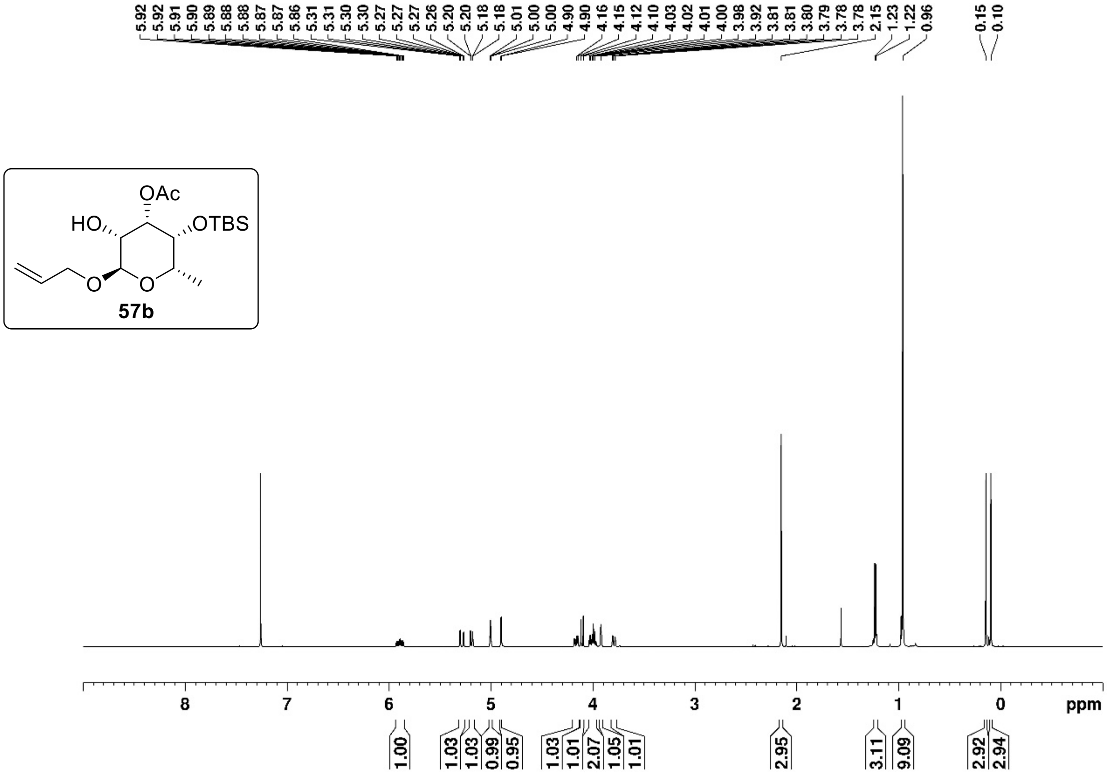
5.92 5.92 5.91 5.90 5.89 5.88 5.88 5.87 5.87 5.86 5.31 5.31 5.30 5.30 5.27 5.27 5.26 5.20 5.20 5.18 5.18 5.01 5.00 5.00 4.90 4.90 4.16 4.15 4.12 4.10 4.03 4.02 4.01 4.00 3.98 3.92 3.81 3.81 3.80 3.79 3.78 2.15 1.23 1.22 0.96 0.15 0.10
OAc
HO
OTBS
O
O
57b
8 7 6 5 4 3 2 1 0 ppm
1.00 1.03 1.03 0.99 0.95 1.03 1.01 2.07 1.05 1.01 2.95 3.11 9.09 2.92 2.94

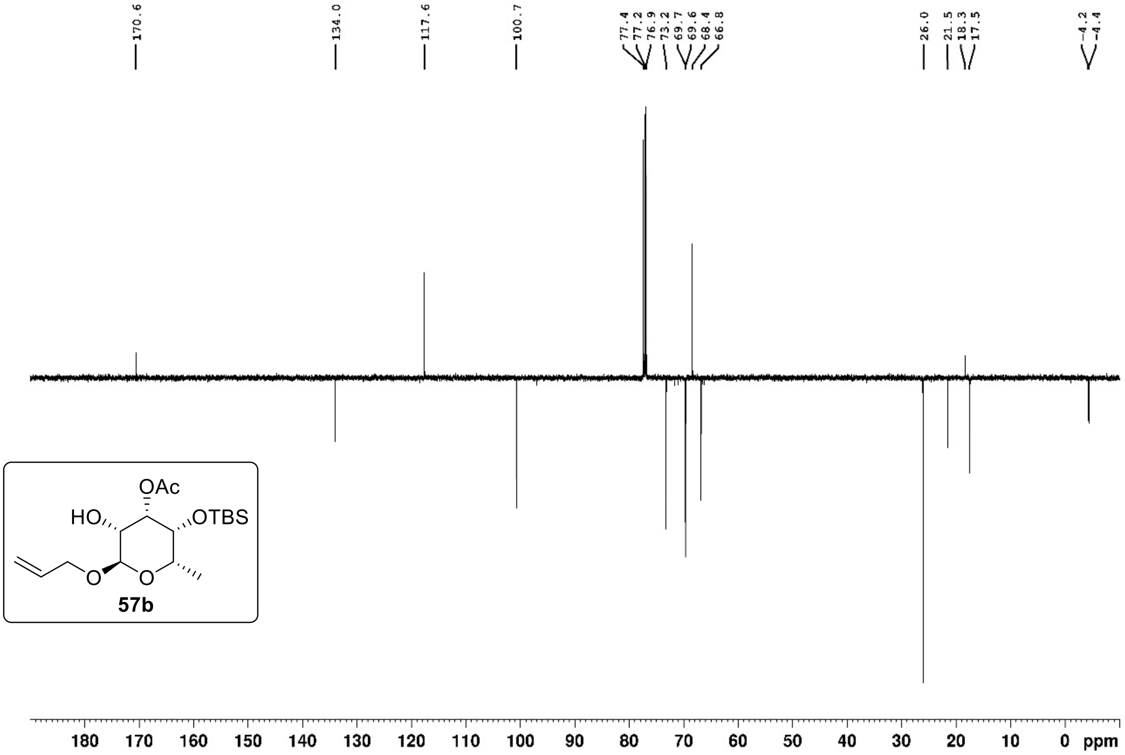
170.6 134.0 117.6 100.7 77.4 77.2 76.9 73.2 69.7 69.6 68.4 66.8 26.0 21.5 18.3 17.5 -4.2 -4.4
OAc
HO
OTBS
O
O
57b
180 170 160 150 140 130 120 110 100 90 80 70 60 50 40 30 20 10 0 ppm

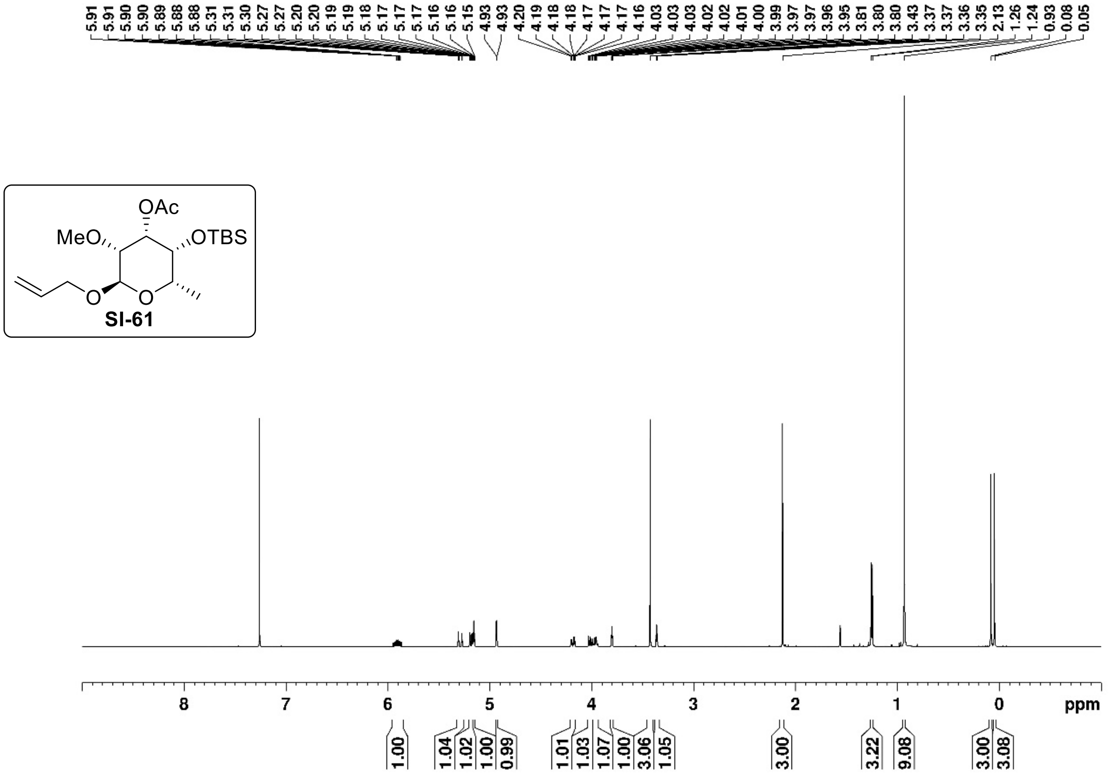
OAc
MeO
OTBS
O
O
SI-61
ppm

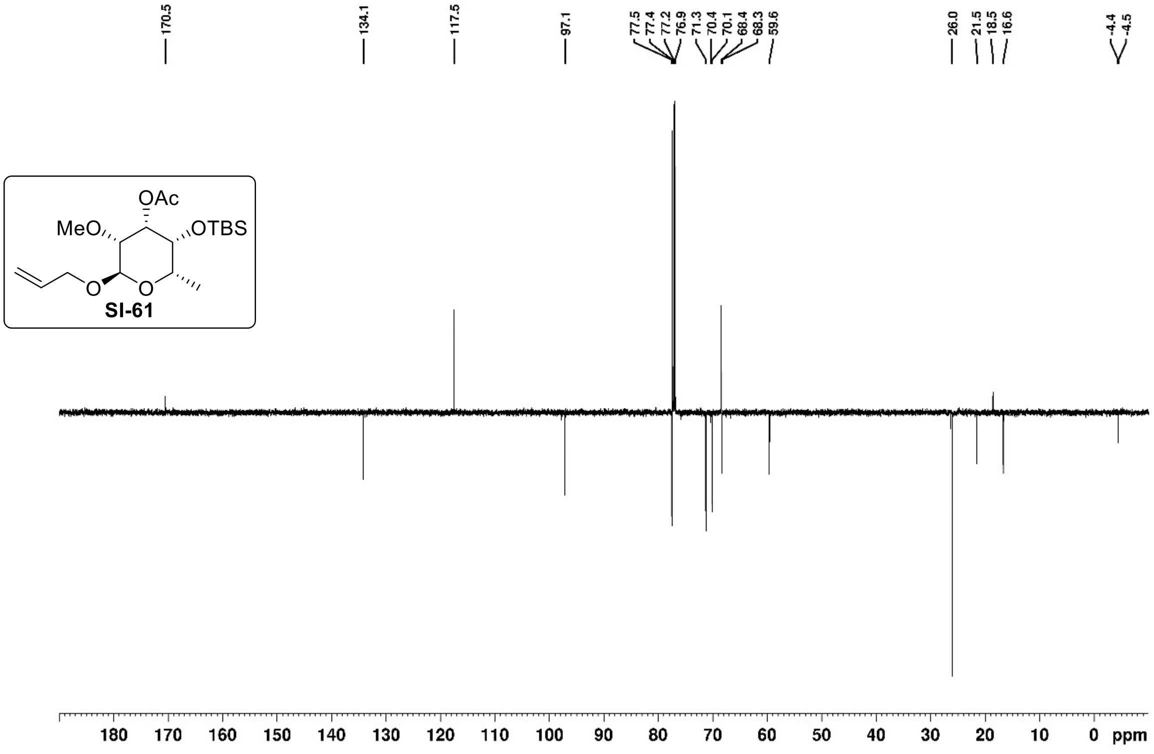
OAc
MeO
OTBS
O
O
SI-61
ppm

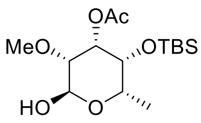
OAc
MeO
OTBS
HO
O

58b

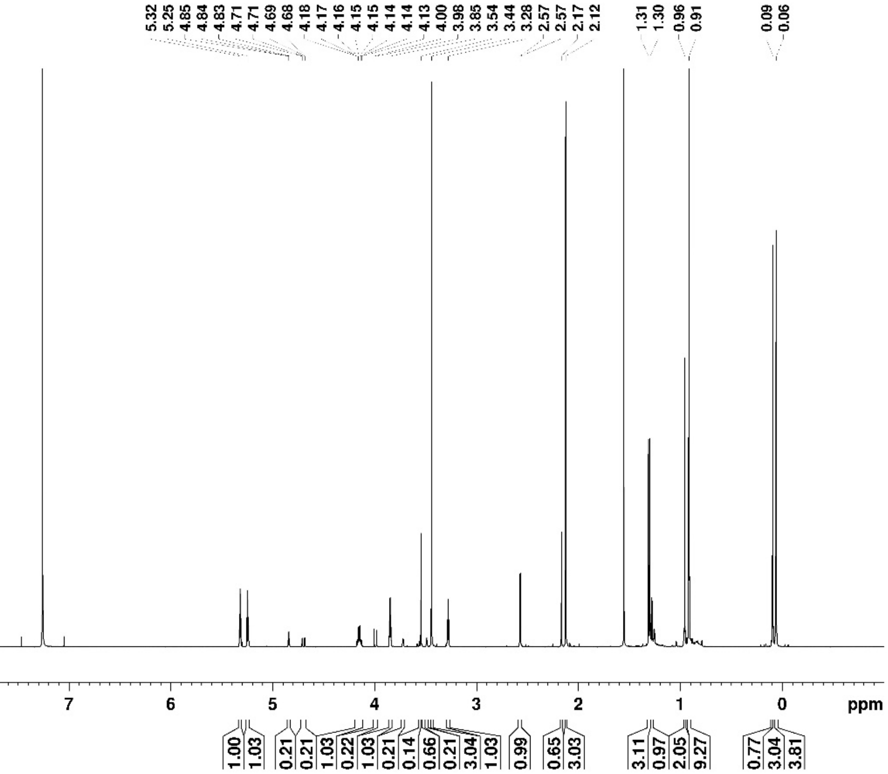
5.32 5.25 4.85 4.84 4.83 4.71 4.71 4.69 4.68 4.18 4.17 4.16 4.15 4.15 4.14 4.14 4.13 4.00 3.98 3.85 3.54 3.44 3.28 2.57 2.57 2.17 2.12 1.31 1.30 0.96 0.91 0.09 0.06
8 7 6 5 4 3 2 1 0 ppm
1.00 1.03 0.21 0.21 1.03 0.22 1.03 0.21 0.14 0.66 0.21 3.04 1.03 0.99 0.65 3.03 3.11 0.97 2.05 9.27 0.77 3.04 3.81

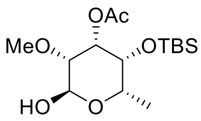
OAc
MeO
OTBS
HO
O

58b

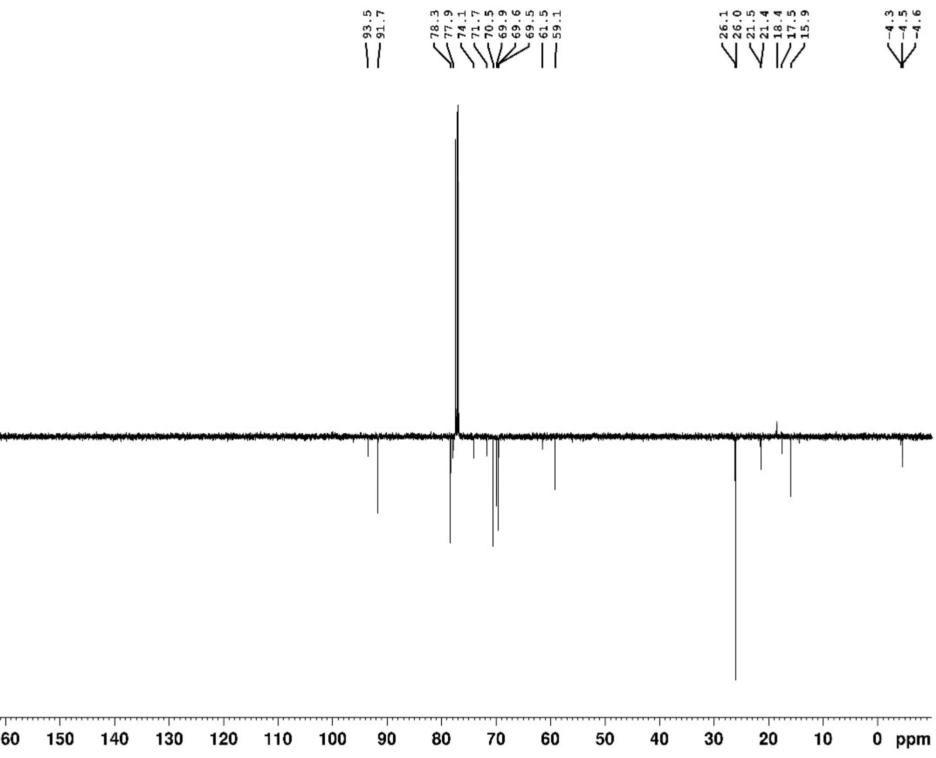
170.4 93.5 91.7 78.3 77.9 74.1 71.7 70.5 69.9 69.6 69.5 61.5 59.1 26.1 26.0 21.5 21.4 18.4 17.5 15.9 -4.3 -4.5 -4.6
180 170 160 150 140 130 120 110 100 90 80 70 60 50 40 30 20 10 0 ppm

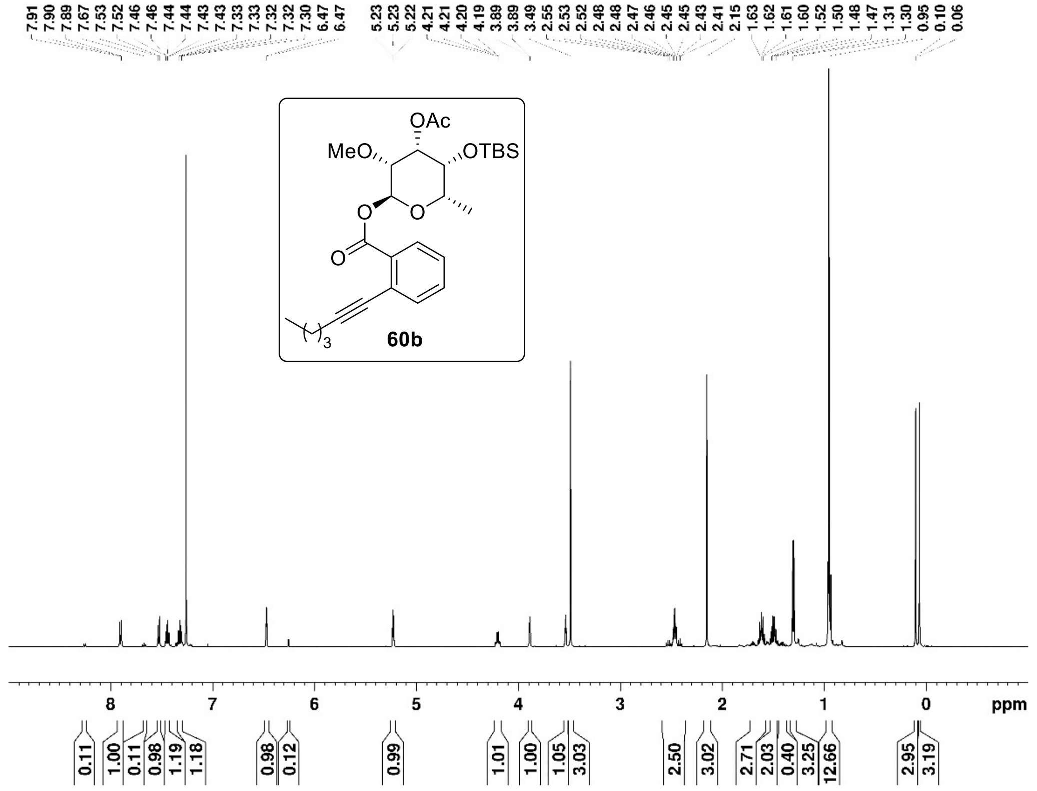
7.91 7.90 7.89 7.67 7.53 7.52 7.46 7.44 7.44 7.43 7.43 7.33 7.33 7.32 7.32 7.30 6.47 6.47
5.23 5.23 5.22 4.21 4.21 4.20 4.19 3.89 3.89 3.49 2.55 2.53 2.52 2.48 2.48 2.47 2.46 2.45 2.45 2.43 2.41 2.15 1.63 1.62 1.61 1.60 1.52 1.50 1.48 1.47 1.31 1.30 0.95 0.10 0.06
OAc
MeO
OTBS
O
O
O
3
60b
8 7 6 5 4 3 2 1 0 ppm
0.11 1.00 0.11 0.98 1.19 1.18 0.98 0.12 0.99 1.01 1.00 1.05 3.03 2.50 3.02 2.71 2.03 0.40 3.25 12.66 2.95 3.19

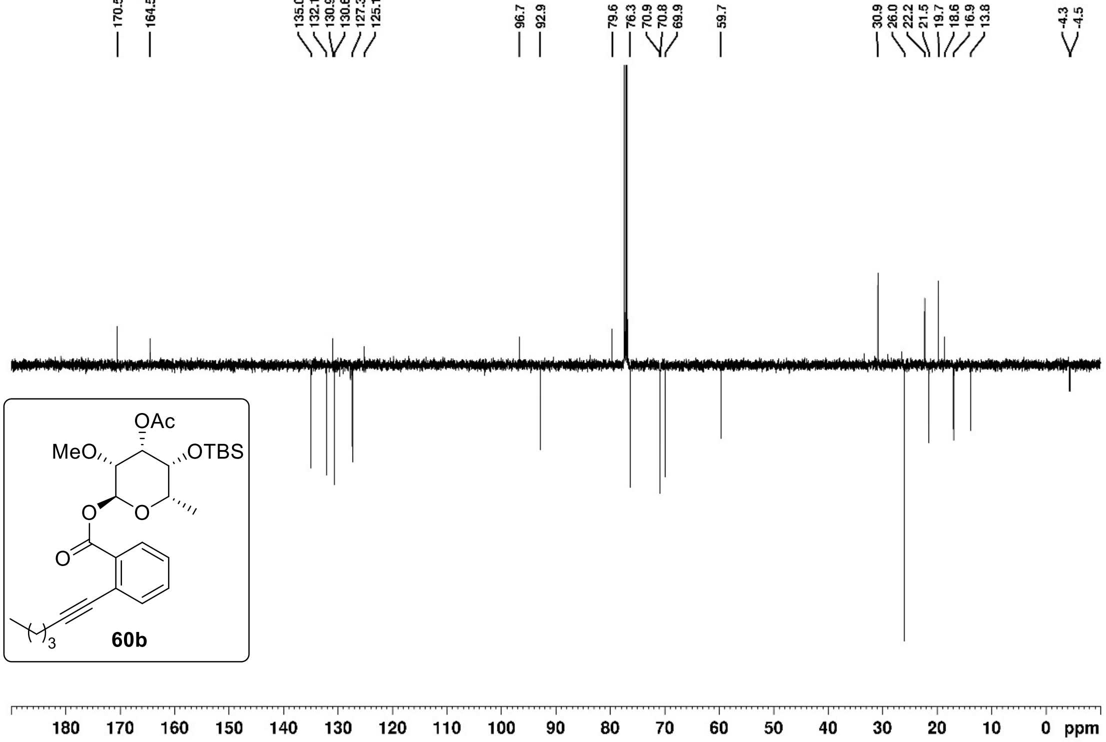
170.5 164.5 135.0 132.1 130.9 130.6 127.3 125.1 96.7 92.9 79.6 76.3 70.9 70.8 69.9 59.7 30.9 26.0 22.2 21.5 19.7 18.6 16.9 13.8 -4.3 -4.5
OAc
MeO
OTBS
O
O
O
3
60b
180 170 160 150 140 130 120 110 100 90 80 70 60 50 40 30 20 10 0 ppm

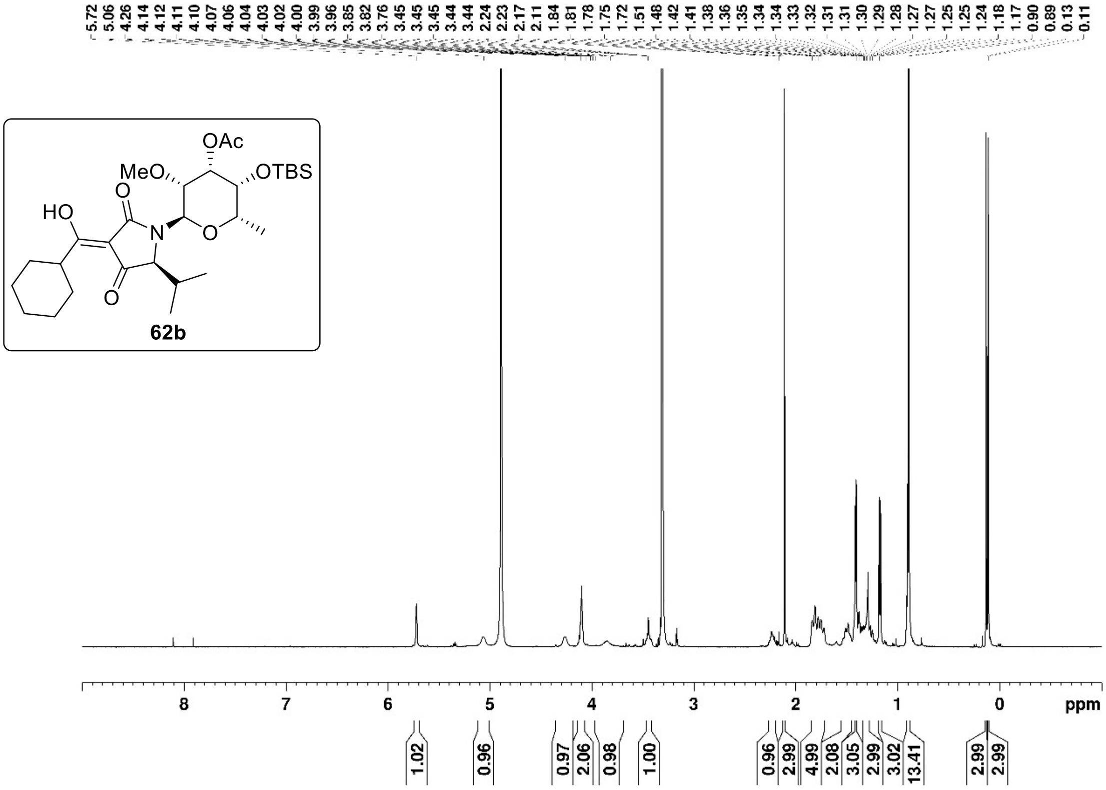
OAc
MeO
OTBS
HO
O
N
62b
ppm

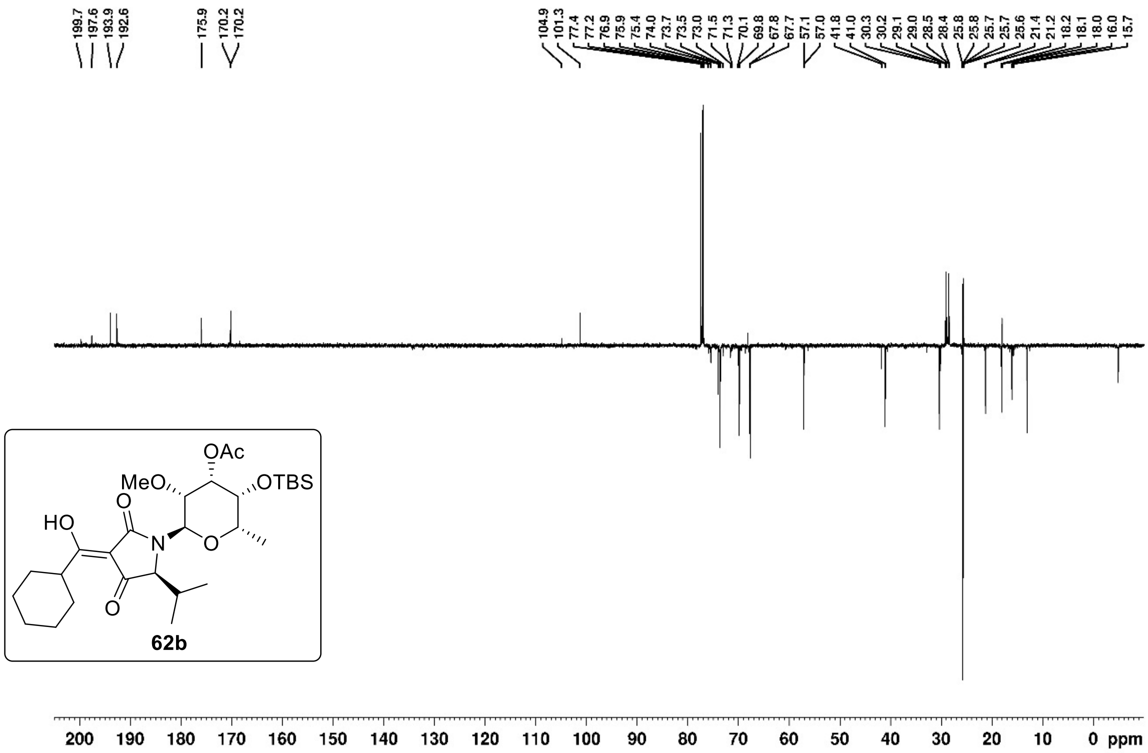
OAc
MeO
OTBS
HO
O
N
62b
ppm

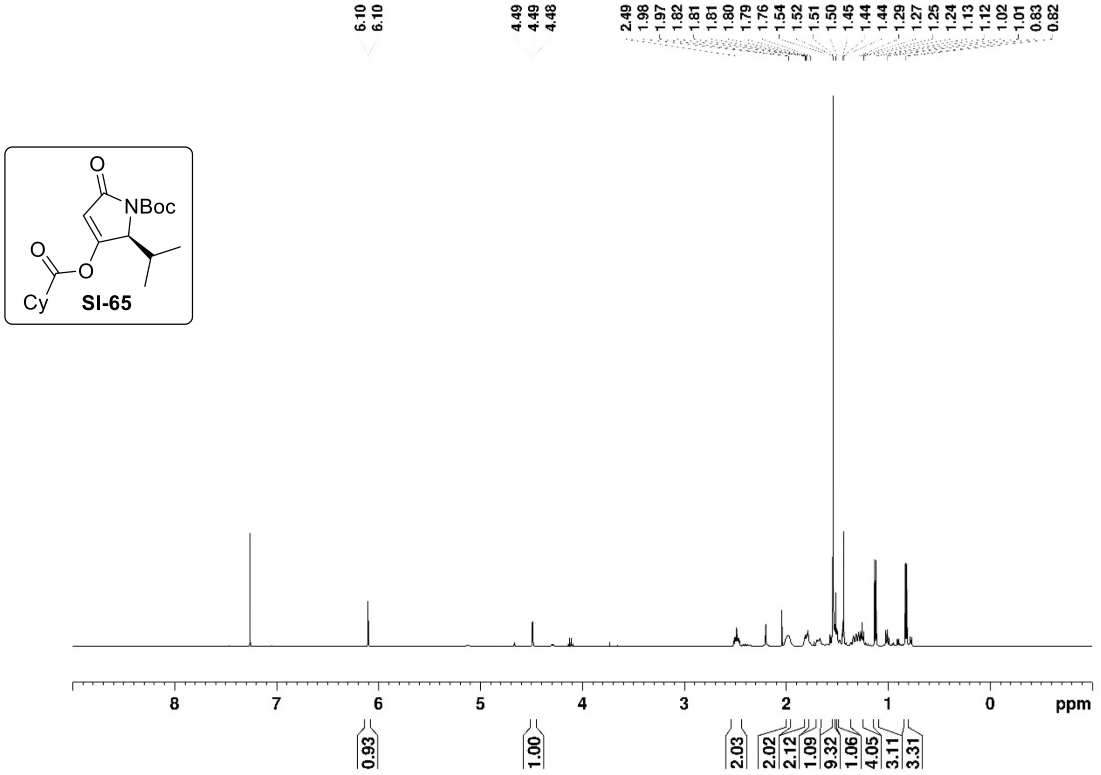
6.10
6.10
4.49
4.49
4.48
2.49
1.98
1.97
1.82
1.81
1.81
1.80
1.79
1.76
1.54
1.52
1.51
1.50
1.45
1.44
1.44
1.29
1.27
1.25
1.24
1.13
1.12
1.02
1.01
0.83
0.82
O
NBoc
O
O
Cy
SI-65
8
7
6
5
4
3
2
1
0
ppm
0.93
1.00
2.03
2.02
2.12
1.09
9.32
1.06
4.05
3.11
3.31

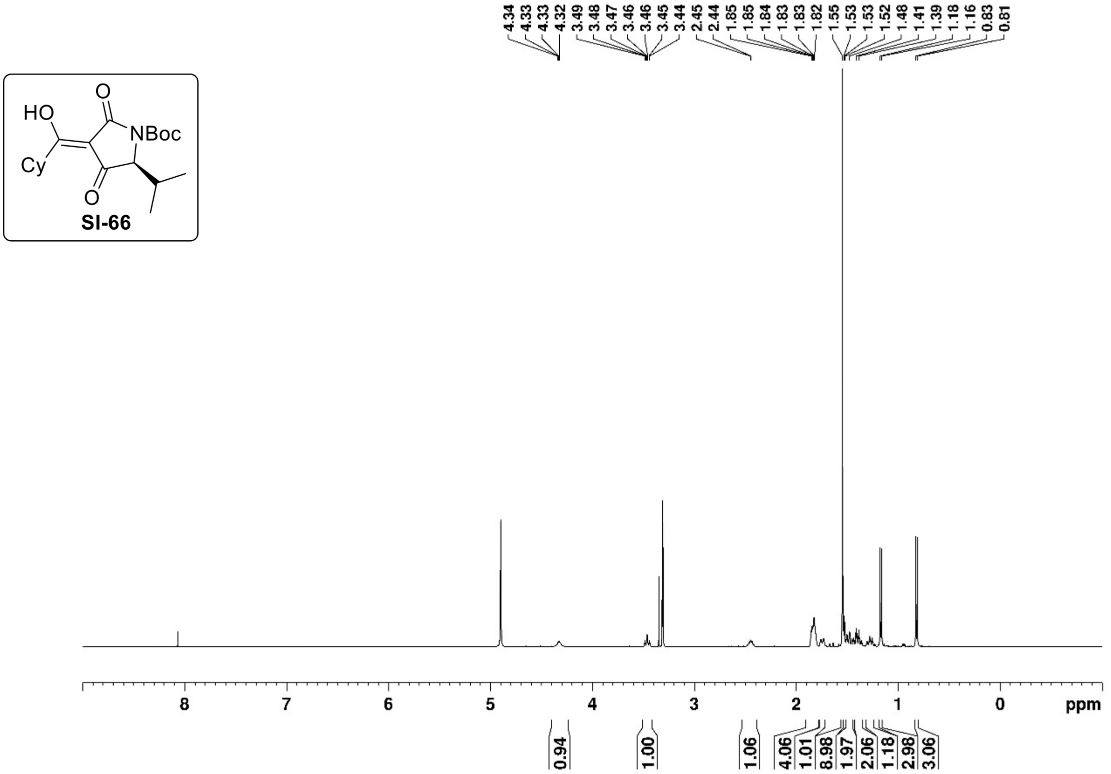
4.34
4.33
4.33
4.32
3.49
3.48
3.47
3.46
3.46
3.45
3.44
2.45
2.44
1.85
1.85
1.84
1.83
1.83
1.82
1.55
1.53
1.53
1.52
1.48
1.41
1.39
1.18
1.16
0.83
0.81
HO
O
NBoc
Cy
O
SI-66
8
7
6
5
4
3
2
1
0
ppm
0.94
1.00
1.06
4.06
1.01
8.98
1.97
2.06
1.18
2.98
3.06

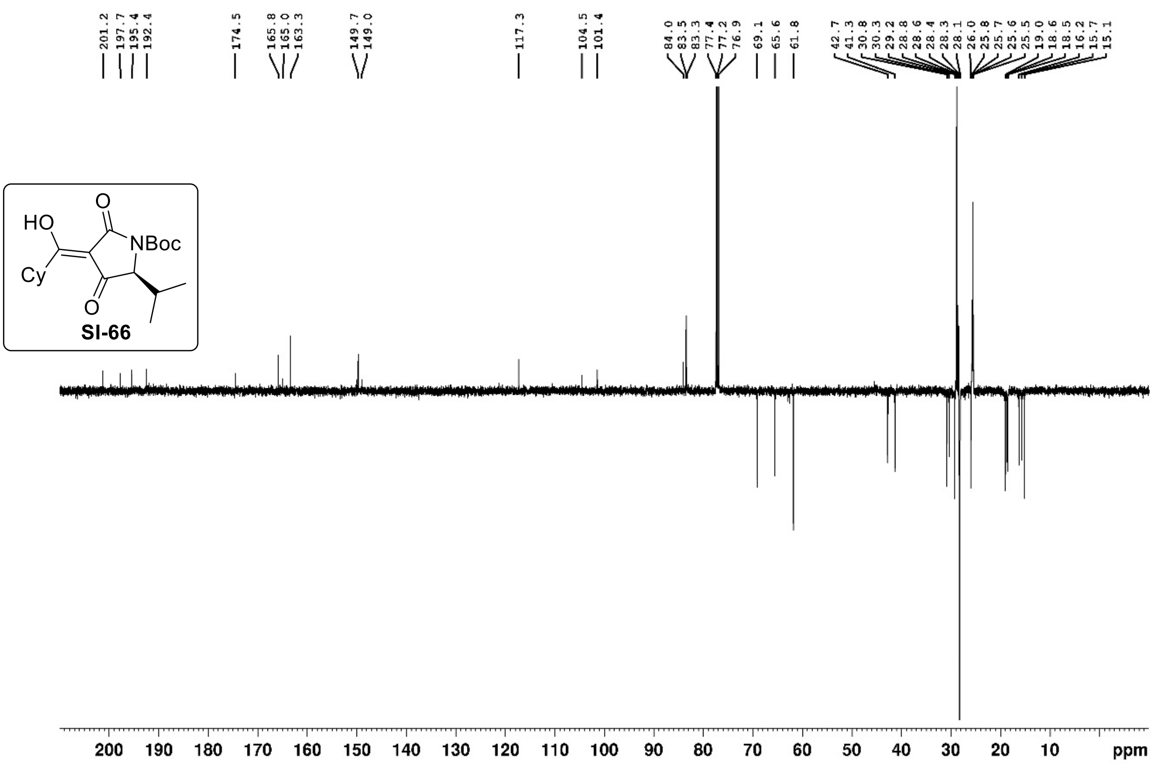
201.2
197.7
195.4
192.4
174.5
165.8
165.0
163.3
149.7
149.0
117.3
104.5
101.4
84.0
83.5
83.3
77.4
77.2
76.9
69.1
65.6
61.8
42.7
41.3
30.8
30.3
29.2
28.8
28.6
28.4
28.3
28.1
26.0
25.8
25.7
25.6
25.5
19.0
18.6
18.5
16.2
15.7
15.1
HO
O
NBoc
Cy
O
SI-66
200 190 180 170 160 150 140 130 120 110 100 90 80 70 60 50 40 30 20 10 ppm

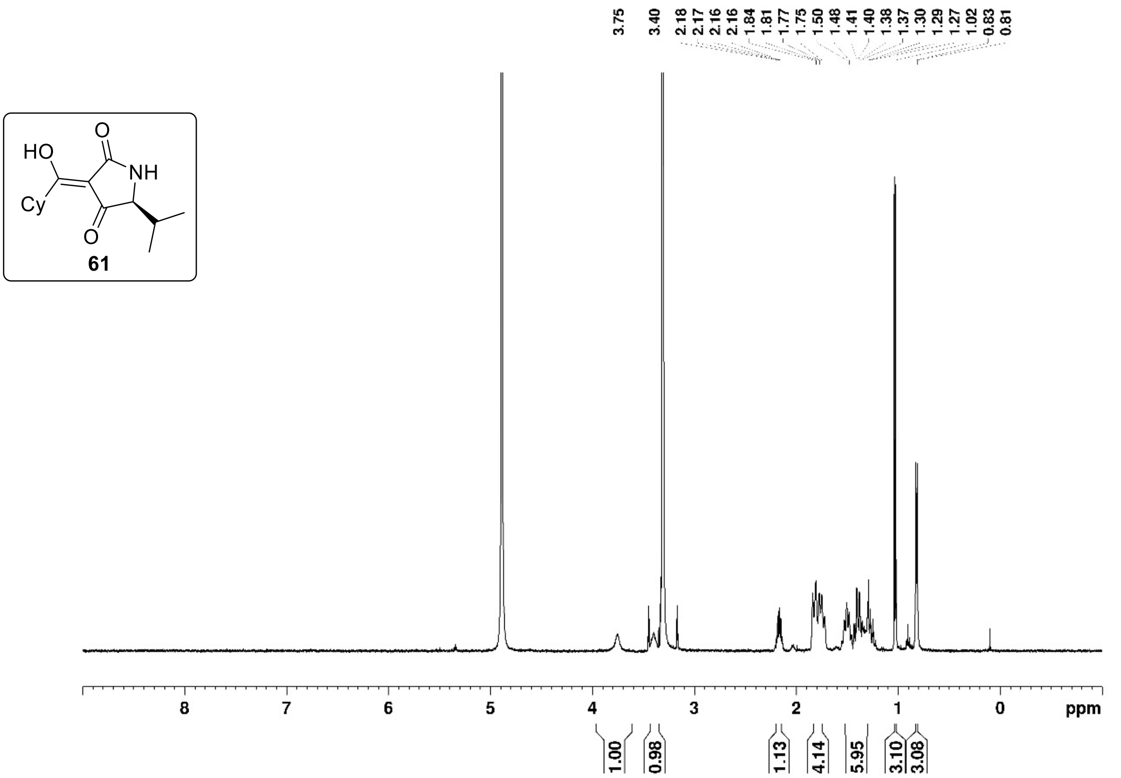
3.75
3.40
2.18
2.17
2.16
2.16
1.84
1.81
1.77
1.75
1.50
1.48
1.41
1.40
1.38
1.37
1.30
1.29
1.27
1.02
0.83
0.81
HO
O
NH
Cy
O
61
8 7 6 5 4 3 2 1 0 ppm
1.00
0.98
1.13
4.14
5.95
3.10
3.08

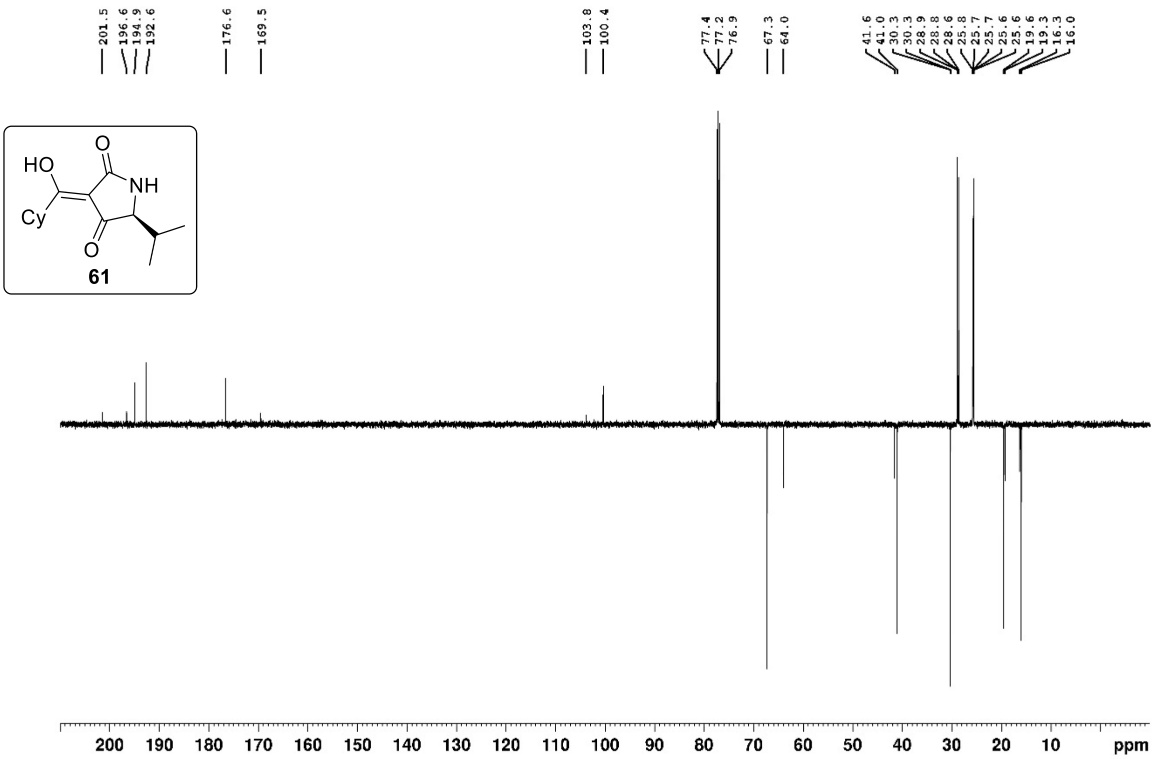
201.5
196.6
194.9
192.6
176.6
169.5
103.8
100.4
77.4
77.2
76.9
67.3
64.0
41.6
41.0
30.3
30.3
28.9
28.8
28.6
25.8
25.7
25.7
25.6
25.6
19.6
19.3
16.3
16.0
HO
O
NH
Cy
O
61
200 190 180 170 160 150 140 130 120 110 100 90 80 70 60 50 40 30 20 10 ppm

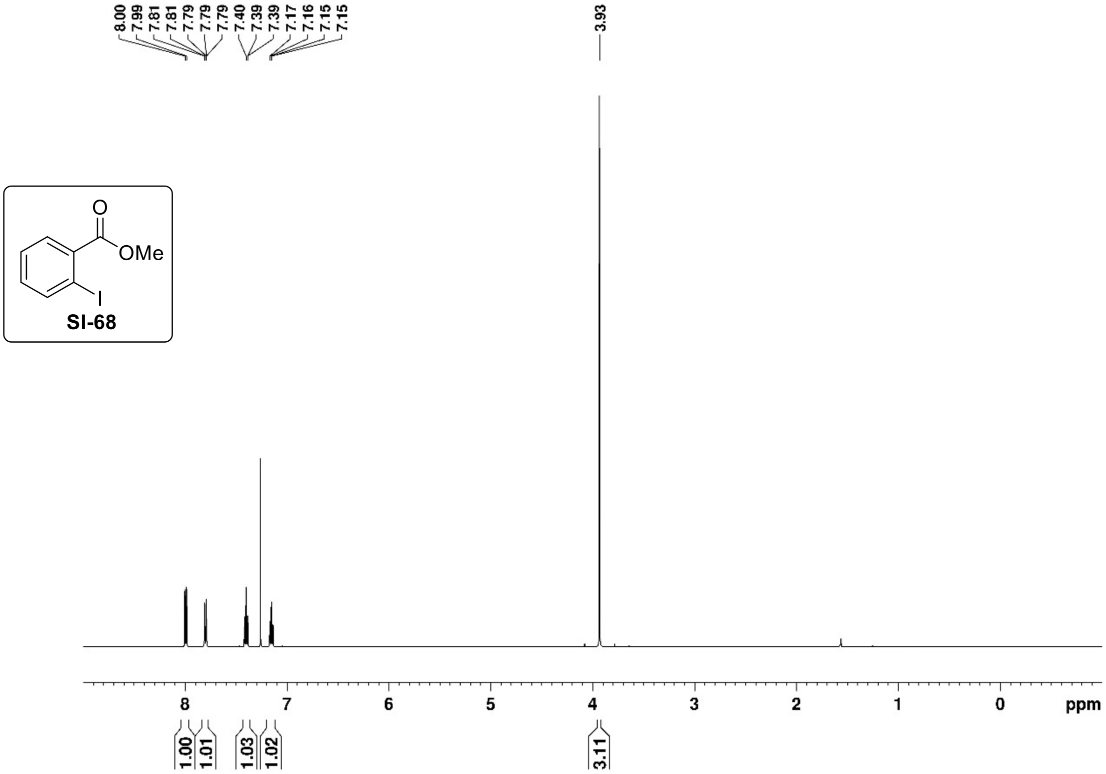
8.00
7.99
7.81
7.81
7.79
7.79
7.79
7.40
7.39
7.39
7.17
7.16
7.15
7.15
3.93
O
OMe
I
SI-68
8 7 6 5 4 3 2 1 0 ppm
1.00
1.01
1.03
1.02
3.11

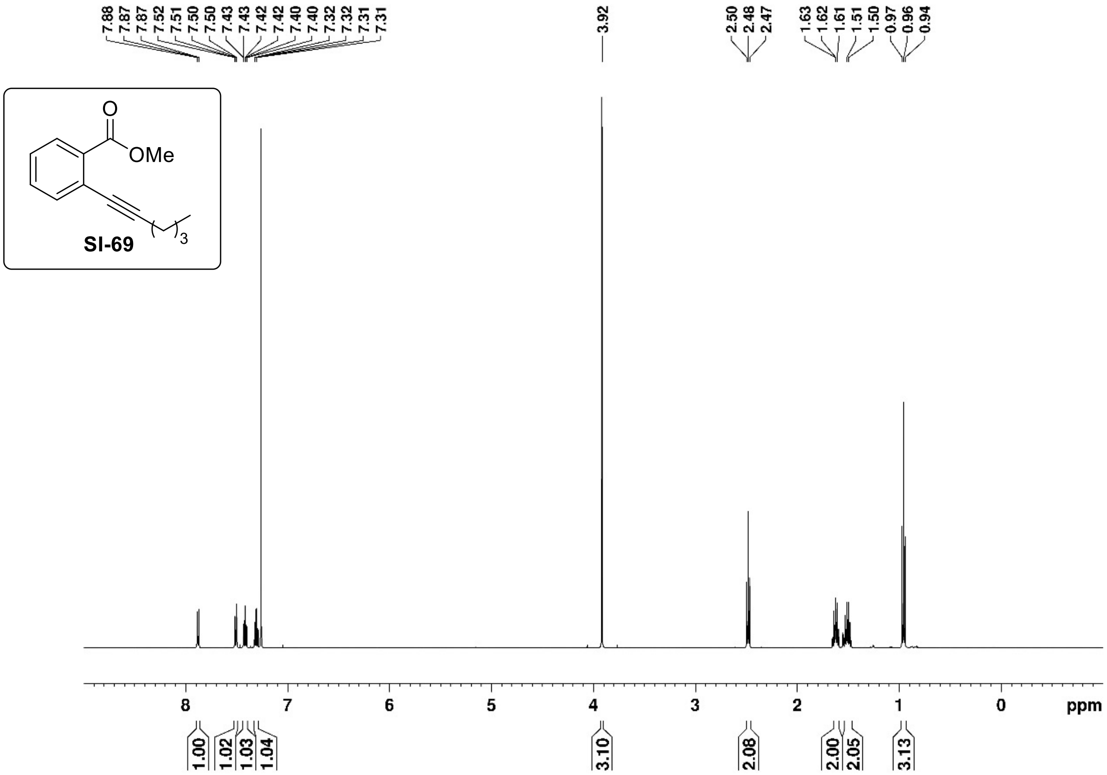

7.88
7.87
7.87
7.52
7.51
7.50
7.50
7.43
7.43
7.42
7.42
7.40
7.40
7.32
7.32
7.31
7.31
3.92
2.50
2.48
2.47
1.63
1.62
1.61
1.51
1.50
0.97
0.96
0.94
O
OMe
3
SI-69
8
7
6
5
4
3
2
1
0
ppm
1.00
1.02
1.03
1.04
3.10
2.08
2.00
2.05
3.13

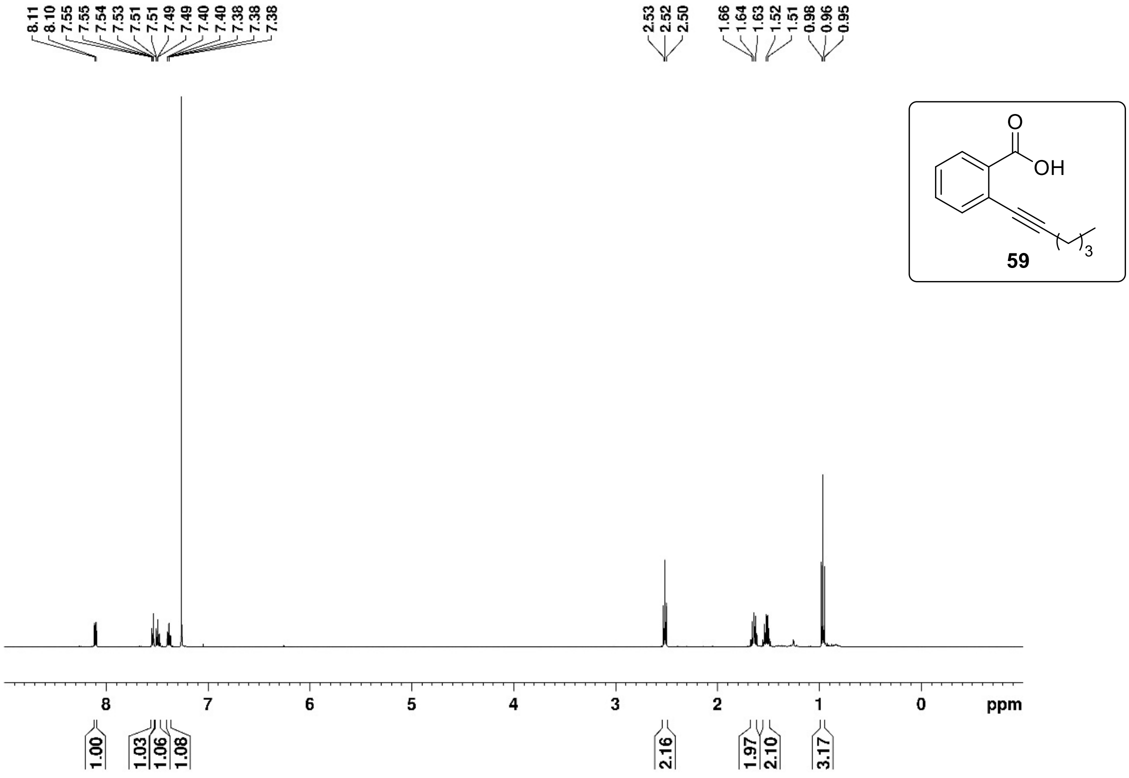

8.11
8.10
7.55
7.54
7.53
7.51
7.51
7.49
7.49
7.40
7.40
7.38
7.38
7.38
2.53
2.52
2.50
1.66
1.64
1.63
1.52
1.51
0.98
0.96
0.95
O
OH
3
59
8
7
6
5
4
3
2
1
0
ppm
1.00
1.03
1.06
1.08
2.16
1.97
2.10
3.17

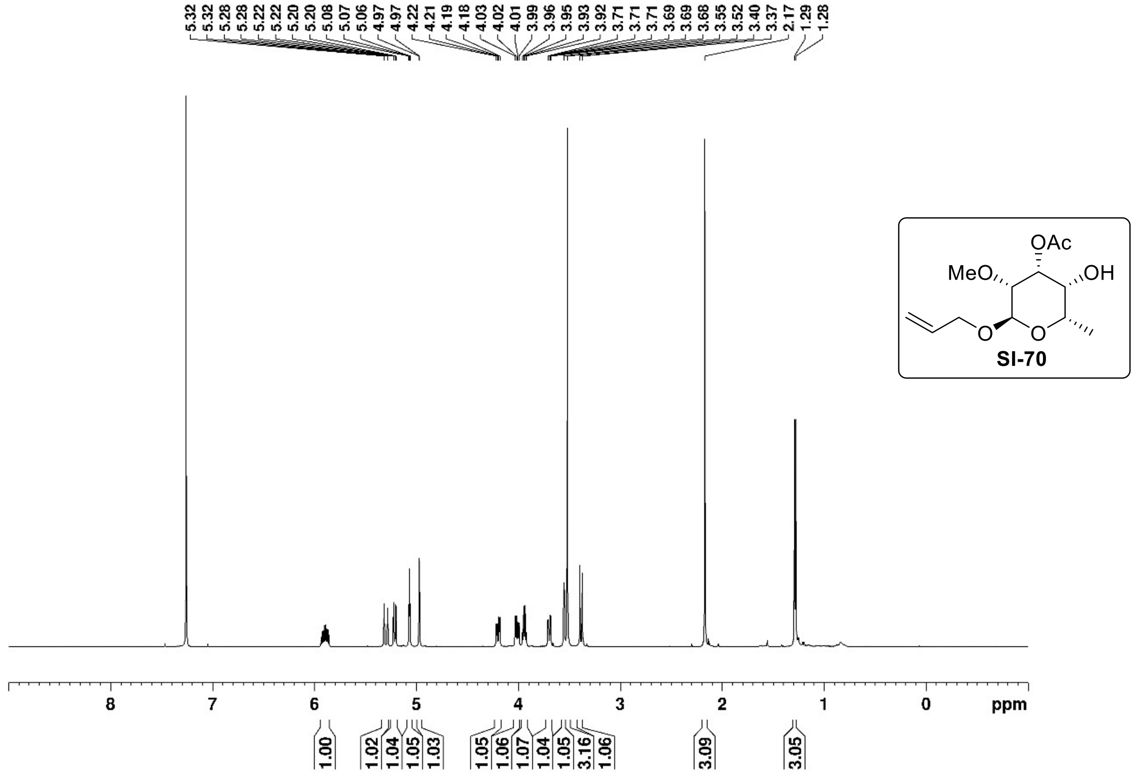
5.32 5.32 5.28 5.28 5.22 5.22 5.20 5.20 5.08 5.07 5.06 4.97 4.97 4.22 4.21 4.19 4.18 4.03 4.02 4.01 3.99 3.96 3.95 3.93 3.92 3.71 3.71 3.71 3.69 3.69 3.68 3.55 3.52 3.40 3.37 2.17 1.29 1.28
8 7 6 5 4 3 2 1 0 ppm
1.00 1.02 1.04 1.05 1.03 1.05 1.06 1.07 1.04 1.05 3.16 1.06 3.09 3.05
OAc
MeO
OH
O
O
SI-70

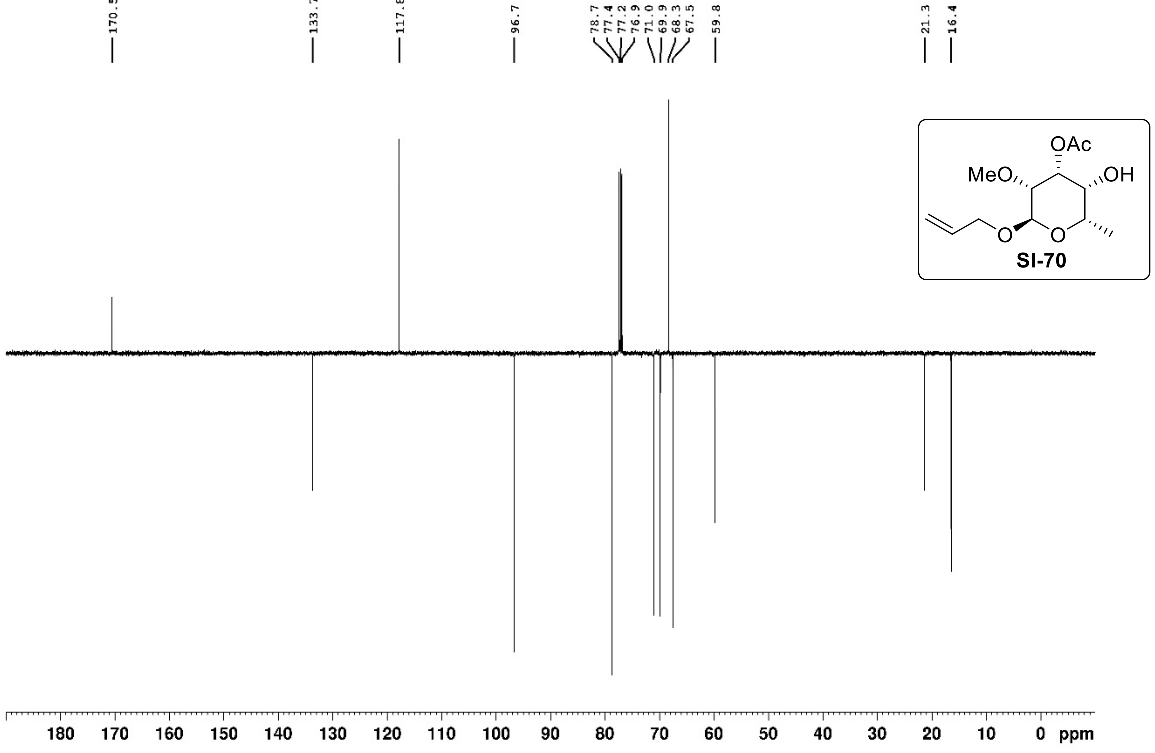
170.5 133.7 117.8 96.7 78.7 77.4 77.2 76.9 71.0 69.9 68.3 67.5 59.8 21.3 16.4
180 170 160 150 140 130 120 110 100 90 80 70 60 50 40 30 20 10 0 ppm
OAc
MeO
OH
O
O
SI-70

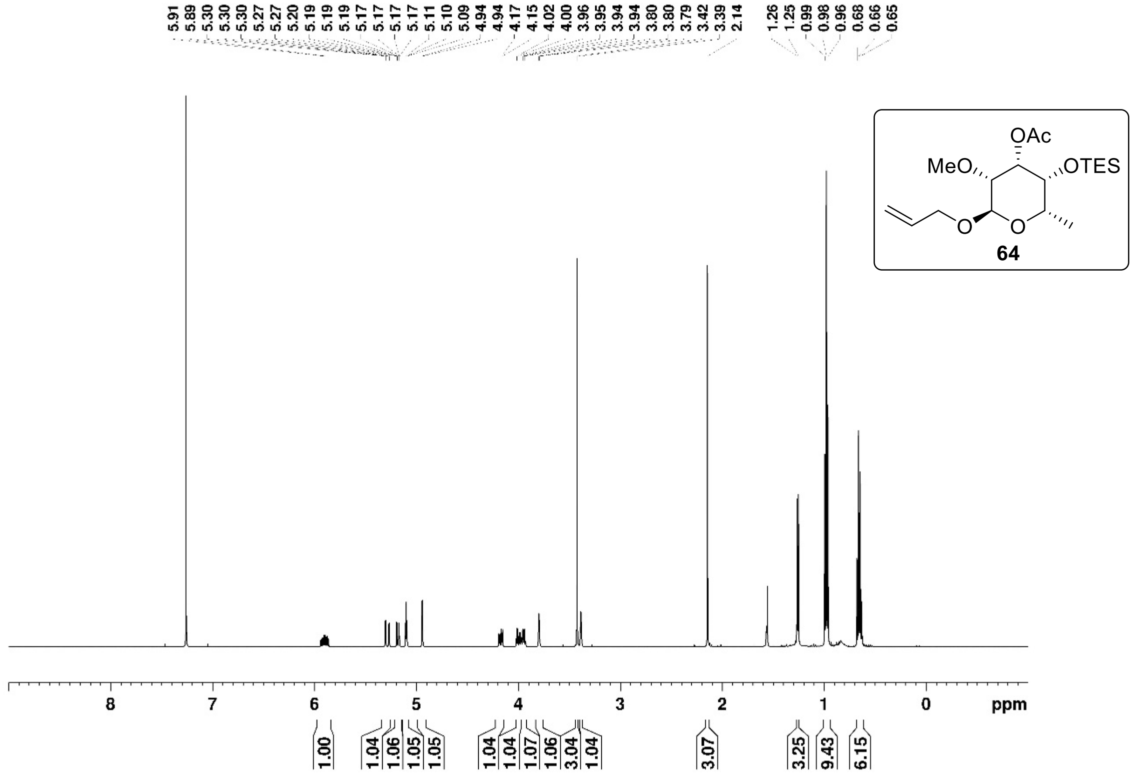
5.91 5.89 5.30 5.30 5.30 5.27 5.20 5.19 5.19 5.19 5.17 5.17 5.17 5.11 5.10 5.09 4.94 4.94 4.17 4.15 4.02 4.00 3.96 3.95 3.94 3.94 3.80 3.80 3.79 3.42 3.39 2.14 1.26 1.25 0.99 0.98 0.96 0.68 0.66 0.65
OAc
MeO
OTES
O
O
64
8 7 6 5 4 3 2 1 0 ppm
1.00 1.04 1.06 1.05 1.05 1.04 1.04 1.07 1.06 3.04 1.04 3.07 3.25 9.43 6.15

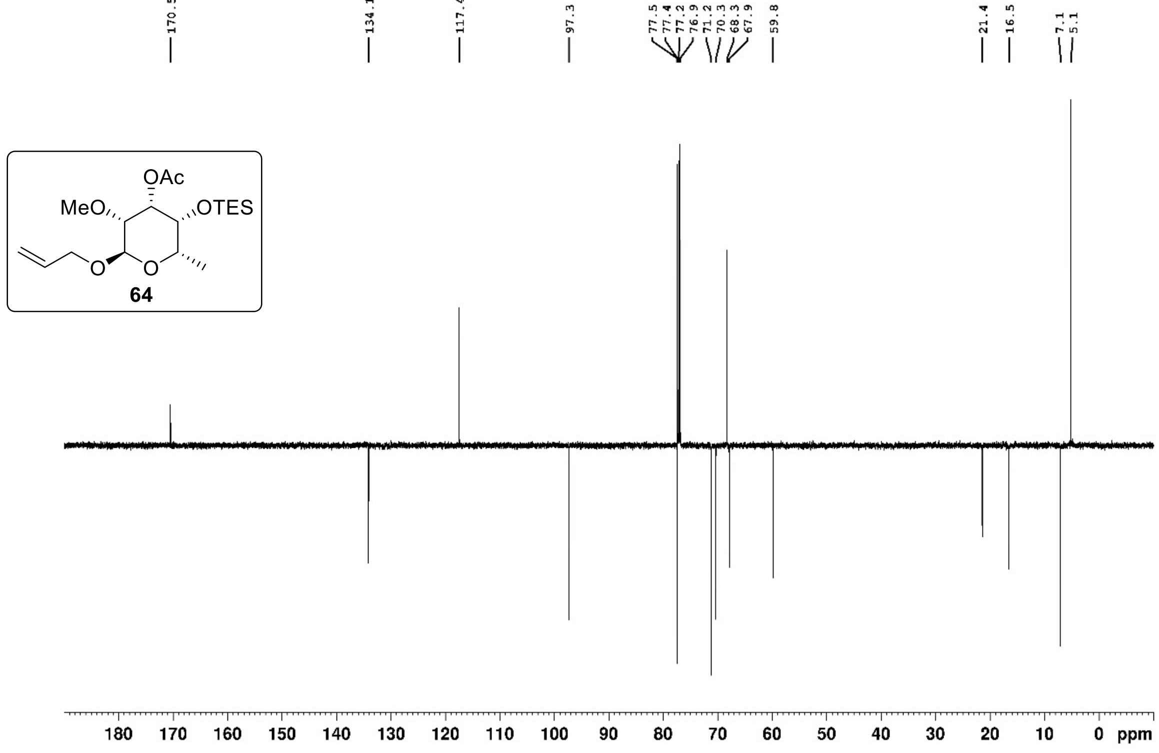
170.5 134.1 117.4 97.3 77.5 77.4 77.2 76.9 71.2 70.3 68.3 67.9 59.8 21.4 16.5 7.1 5.1
OAc
MeO
OTES
O
O
64
180 170 160 150 140 130 120 110 100 90 80 70 60 50 40 30 20 10 0 ppm

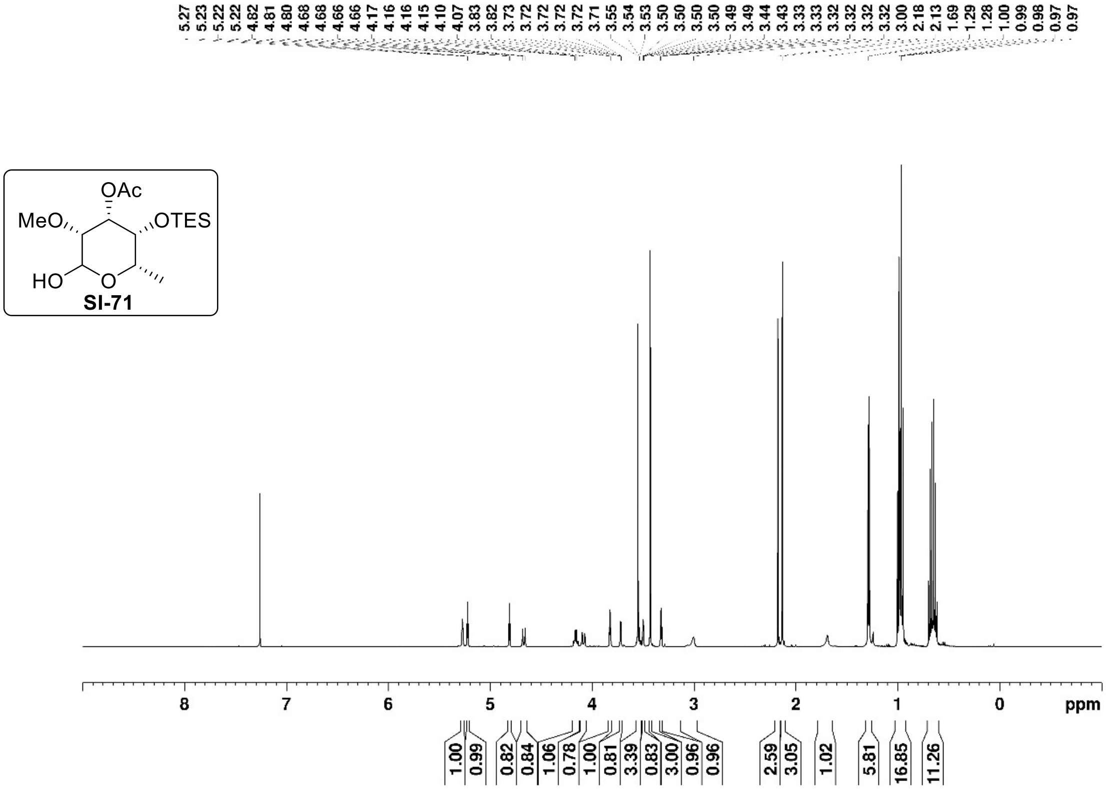
OAc
MeO
OTES
HO
O
SI-71
ppm

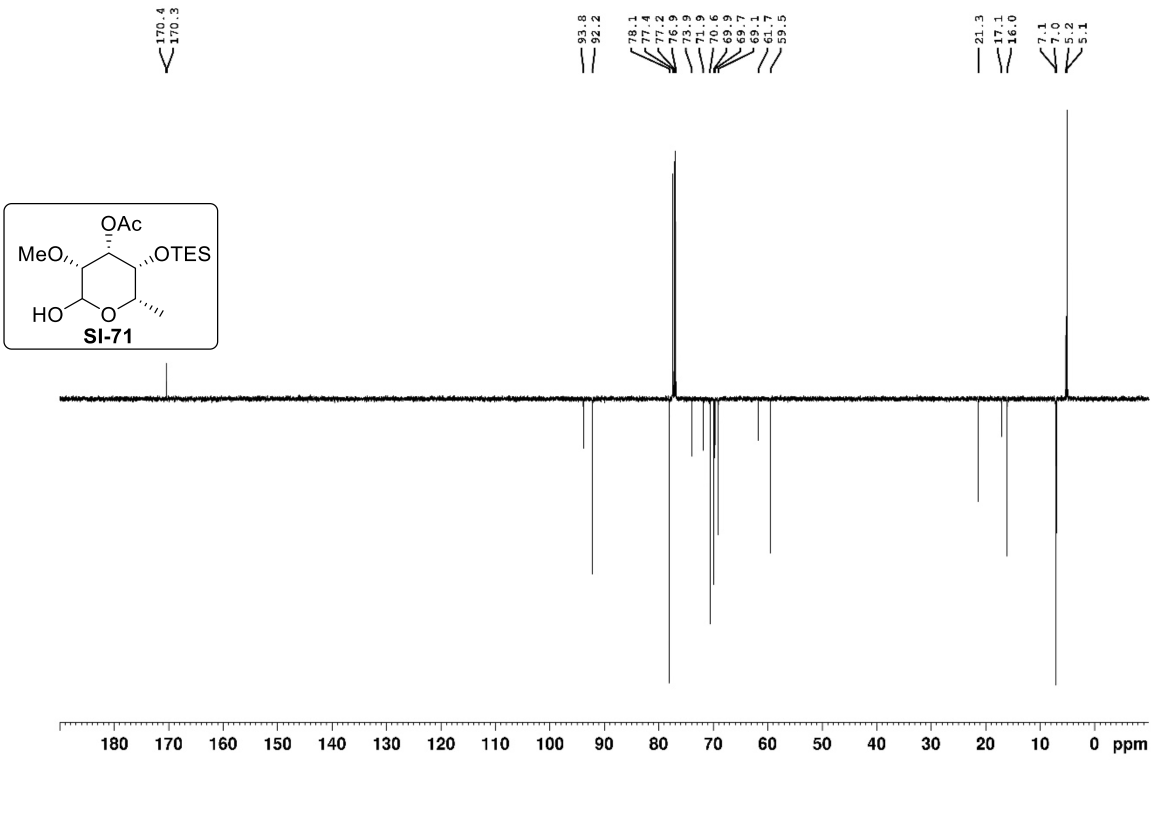
170.4
170.3
93.8
92.2
21.3
17.1
16.0
OAc
MeO
OTES
HO
O
SI-71
ppm

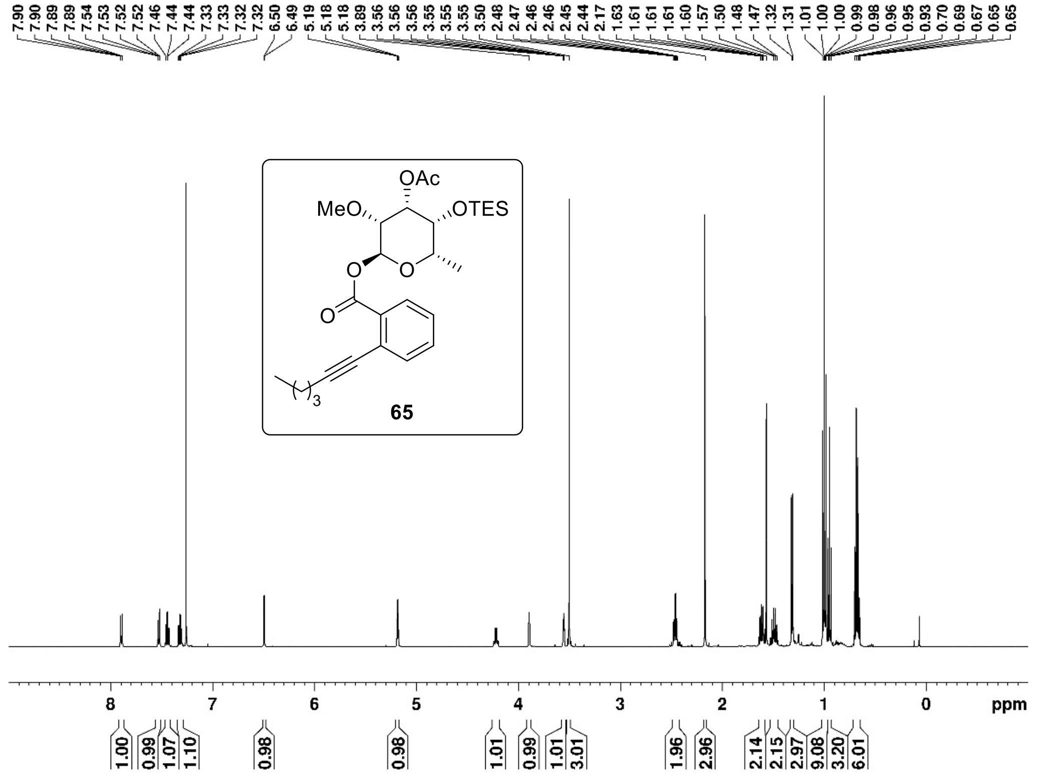

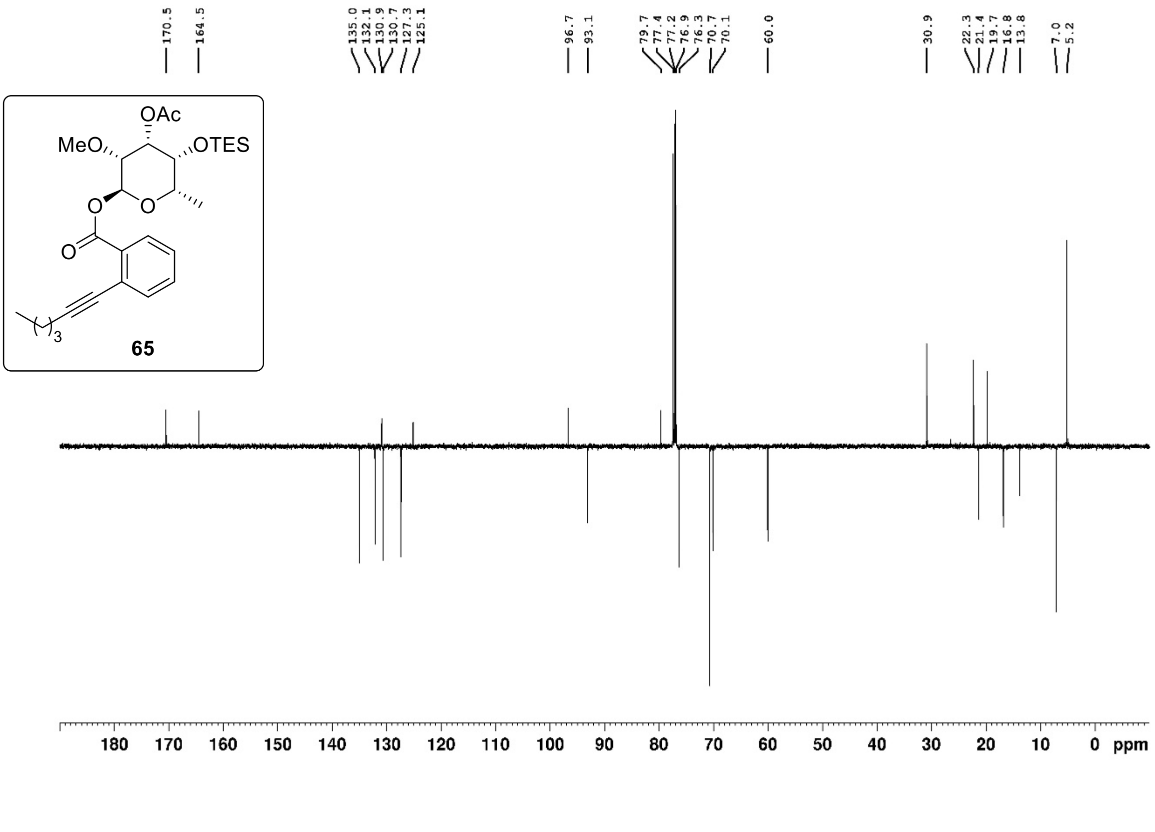

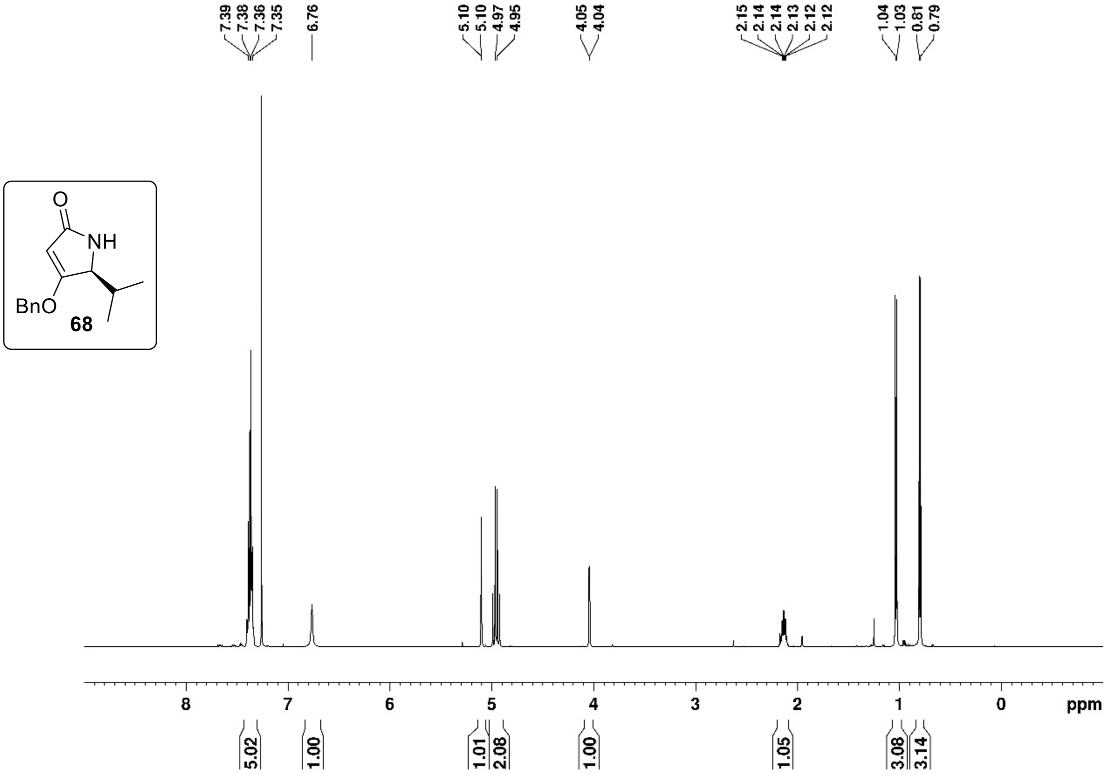
7.39 7.38 7.36 7.35 6.76 5.10 5.10 4.97 4.95 4.05 4.04 2.15 2.14 2.14 2.13 2.12 2.12 1.04 1.03 0.81 0.79
O
NH
BnO
68
8 7 6 5 4 3 2 1 0 ppm
5.02 1.00 1.01 2.08 1.00 1.05 3.08 3.14

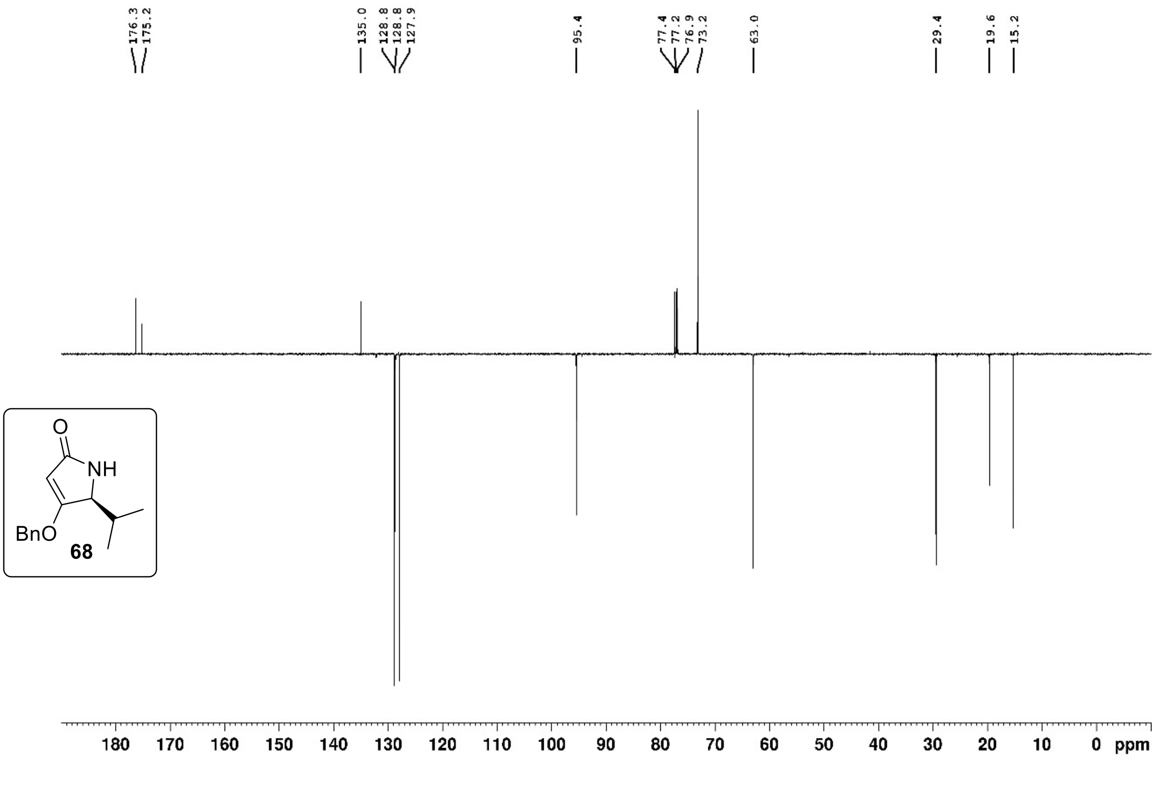
176.3 175.2 135.0 128.8 128.8 127.9 95.4 77.4 77.2 76.9 73.2 63.0 29.4 19.6 15.2
O
NH
BnO
68
180 170 160 150 140 130 120 110 100 90 80 70 60 50 40 30 20 10 0 ppm
